# Integrative analysis of bulk and single-cell RNA sequencing reveals the gene expression profile and the critical signaling pathways of type II CPAM

**DOI:** 10.1186/s13578-024-01276-8

**Published:** 2024-07-18

**Authors:** Fengxia Li, Zheng Tan, Hongyu Chen, Yue Gao, Jie Xia, Ting Huang, Liang Liang, Jian Zhang, Xianghong Zhang, Xucong Shi, Qiang Chen, Qiang Shu, Lan Yu

**Affiliations:** 1 Children’s Hospital, Zhejiang University School of Medicine, National Clinical Research Center for Child Health, Hangzhou, Zhejiang China; 2 Department of Thoracic Surgery, Children’s Hospital, Zhejiang University School of Medicine, National Clinical Research Center for Child Health, Hangzhou, Zhejiang China; 3 Department of Cardiac Intensive Care Unit, Children’s Hospital, Zhejiang University School of Medicine, National Clinical Research Center for Child Health, Hangzhou, Zhejiang China; 4 Department of Cardiac Surgery, Children’s Hospital, Zhejiang University School of Medicine, National Clinical Research Center for Child Health, Hangzhou, Zhejiang China; 5 Department of General Surgery, Jiangxi Provincial Children’s Hospital, Jiangxi, China; 6 Department of Molecular Genetics, University of Toronto, Toronto, ON Canada

**Keywords:** Congenital pulmonary airway malformation, Bulk RNA sequencing, Single-cell RNA sequencing, Cell communication

## Abstract

**Backgroud:**

Type II congenital pulmonary airway malformation (CPAM) is a rare pulmonary microcystic developmental malformation. Surgical excision is the primary treatment for CPAM, although maternal steroids and betamethasone have proven effective in reducing microcystic CPAM. Disturbed intercellular communication may contribute to the development of CPAM. This study aims to investigate the expression profile and analyze intercellular communication networks to identify genes potentially associated with type II CPAM pathogenesis and therapeutic targets.

**Methods:**

RNA sequencing (RNA-seq) was performed on samples extracted from both the cystic area and the adjacent normal tissue post-surgery in CPAM patients. Iterative weighted gene correlation network analysis (iWGCNA) was used to identify genes specifically expressed in type II CPAM. Single-cell RNA-seq (scRNA-seq) was integrated to unveil the heterogeneity in cell populations and analyze the communication and interaction within epithelial cell sub-populations.

**Results:**

A total of 2,618 differentially expressed genes were identified, primarily enriched in cilium-related biological process and inflammatory response process. Key genes such as *EDN1*, *GPR17*, *FPR2*, and *CHRM1*, involved in the G protein-coupled receptor (GPCR) signaling pathway and playing roles in cell differentiation, apoptosis, calcium homeostasis, and the immune response, were highlighted based on the protein-protein interaction network. Type II CPAM-associated modules, including ciliary function-related genes, were identified using iWGCNA. By integrating scRNA-seq data, *AGR3* (related to calcium homeostasis) and *SLC11A1* (immune related) were identified as the only two differently expressed genes in epithelial cells of CPAM. Cell communication analysis revealed that alveolar type 1 (AT1) and alveolar type 2 (AT2) cells were the predominant communication cells for outgoing and incoming signals in epithelial cells. The ligands and receptors between epithelial cell subtypes included COLLAGEN genes enriched in PI3K-AKT singaling and involved in epithelial to mesenchymal transition.

**Conclusions:**

In summary, by integrating bulk RNA-seq data of type II CPAM with scRNA-seq data, the gene expression profile and critical signaling pathways such as GPCR signaling and PI3K-AKT signaling pathways were revealed. Abnormally expressed genes in these pathways may disrupt epithelial-mesenchymal transition and contribute to the development of CPAM. Given the effectiveness of prenatal treatments of microcystic CPAM using maternal steroids and maternal betamethasone administration, targeting the genes and signaling pathways involved in the development of CPAM presents a promising therapeutic strategy.

**Supplementary Information:**

The online version contains supplementary material available at 10.1186/s13578-024-01276-8.

## Introduction

Congenital pulmonary airway malformation (CPAM) was first described by Ch’in and Tang in 1949 as a rare lesion with systemic edema in immature fetuses [1]. It can be diagnosed prenatally through ultrasound screening or postnatally when patients present with pneumonia. While it can be asymptomatic, CPAM carries potential risk of complications, including cyst infection, respiratory distress, failure to thrive, and malignant transformation [2, 3].

According to the Stocker classification, CPAM can be classified into five types based on cyst size and the hamartomatous components of the lesion [4]. Type 0 is incompatible with life due to solid lesions. Type I lesion typically consists of large single or multiple cysts (greater than 2 cm) with pseudostratified columnar epithelium. Type II usually presents with multiple smaller simple and round cysts (less than 1.5–2 cm) lined by ciliated cuboidal to columnar epithelium. Type I and type II lesions are the most frequent forms of CPAM [5]. Type III lesion can expand the entire lung with cuboidal epithelium. Type IV is composed of thin-walled structure lined by alveolar cells and often presents with mediastinal shift, and pneumothorax.

The pathogenic mechanisms of CPAM are not completely understood. Each type originates from a different part of the bronchial tree and may not be related. Type 0, also known as acinar dysplasia (AcDys), is associated with mutations of *TBX4*, *FGFR2*, and *FGF10* [6–8]. Type IV is associated with the inactivation of *DICER1*, caused by germline mutations of *DICER1* [9]. It is suggested to be redesignated as pleuropulmonary blastoma (PPB) type I, as these two entities are indistinguishable based on imaging and share the same pathogenic mechanisms [10, 11]. Type I and some of type III lesions have recently been found to be driven by mosaic *KRAS* mutations that occur in the lung epithelium during early development [12], while type II is negative for *KRAS* mutations and is believed to be the consequence of bronchial atresia [13].

Potential genes and altered expression of factors that control normal lung development have been indicated in CPAM pathogenesis. Fibroblast growth factors (FGFs), including *FGF7* and *FGF10*, are expressed in the embryonic lung mesenchyme [14, 15] and may act on the epithelium through their receptor FGFR2b to play a critical role in early lung morphogenesis [16]. Overexpression of *FGF10* has been implicated in the formation of lung cysts [17, 18]. Furthermore, inactivation of a Ribonuclease Dicer or zinc finger factor Yin Yang I (*YY1*) gene in the mouse lung epithelium resulted in a few large epithelial pouches and increased the *FGF10* expression [18, 19]. *Yy1* impacts on Sonic Hedgehog (*SHH*) expression [19], and *SHH* inhibits the expression of *FGF10* [20]. A previous transcriptome analysis of CPAM microcyst focused on the lung epithelium identified significant differences in gene expression between CPAM and healthy controls, indicating dysregulation of TGF-beta signaling in CPAM at both mRNA and protein levels [21]. In the development of CPAM, thyroid transcription factor 1 (*TTF-1*) was highly expressed in the bronchiolar-like epithelial cells of the cysts in CPAM types I, II, and III [22]. The Hox gene *Hoxb-5* was also highly expressed in human CPAM lung tissues during the saccular and alveolar states of lung development [23]. Sex determining region Y–box 2 (*Sox2*) was reported to be involved in the differentiation of endoderm epithelium cells to basal cells by regulating the expression of *Trp63* [24], resulting in cystic lesions [25]. Interestingly, *Sox2* was exclusively detected in the affected area of type II CPAM but not in type I CPAM [24], suggesting distinct cell types in the affected epithelium and disparate origins for type I and type II CPAM. Therefore, it is imperative to conduct separate investigation into the cellular composition and gene expression profiles of these different CPAM types.

Currently, surgical excision of cysts remains the primary postnatal therapy for CPAM due to the lack of molecular therapeutic methods, attributed to the incomplete understanding of its pathogenesis. Maternal steroids and betamethasone have shown 47-82% effectiveness in prenatal treatment [26, 27], potentially by restoring the balance of cell proliferation and apoptosis during CPAM development [28, 29]. However, there is limited information to identify which patients benefit most from steroid administration and determine optimal timing [29]. Therefore, understanding the genes or signaling pathways involved in different types of CPAM could aid in developing therapeutic targets and tailoring treatment strategies for optimal patient benefit.

Single-cell RNA sequencing (scRNA-seq) has provided new insights into lung development by revealing the heterogeneity in cell populations and the landscape of intercellular interactions [30, 31]. Disturbed communication between epithelial cells and mesenchymal cells is suggested to be involved in the development of CPAM [32, 33]. Using scRNA-seq, Zhang revealed the abnormal proliferation of epithelial cells in CPAM and the psedotime trajectory of subpopulation of epithelial cells [34]. However, a comprehensive evaluation of the interactions among cells in CPAM has not yet been determined.

In this study, the cellular landscape of CPAM was mapped, and intercellular communication networks were further analyzed using scRNA-seq based on the type II-III scRAN-seq data [34]. The underlying cellular and molecular events related to CPAM disease were identified, deepening our understanding of its etiology and cell composition, which may provide new insights for treatment. Additionally, we integrated our RNA-seq data of type II CPAM to investigate the expression profile and described the genes or signaling pathways that potentially associated with CPAM pathogenesis. Understanding the genes or signaling pathways that are involved in the CPAM pathogenesis may help to develop potential molecular therapeutic targets and identify patients who would benefit most from precise treatment.

## Methods

### Subject preparation

Fifteen patients with CPAM from Children’s Hospital, Zhejiang University School of Medicine were included for analysis. The diagnosis of CPAM was confirmed by histopathological analysis following surgical procedures, classified according to the Stocker classification [4]. During surgery, specimens from the cystic area and adjacent normal tissue served as case and control samples, respectively. Tissues were promptly snap-frozen and stored in liquid nitrogen until for further analysis. All patients were carefully screened for infection at the time of surgery, and those with detected infections were excluded from the analysis.

This study was approved by the ethics committee of the Children’s Hospital, Zhejiang University School of Medicine (2022-IRB-001), and written informed consent was obtained from the patient’s legal guardians.

### RNA extraction, cDNA library preparation and RNA sequencing

Total RNA was extracted from frozen tissues of CPAM subjects using TRIzolR Reagent (Invitrogen), as described in the manufacturer’s protocols. RNA concentration and integrity were determined with an Agilent Bioanalyzer (Agilent Technologies, CA, USA). A total of 3 µg RNA per sample was used as input for RNA sample preparation. Sequencing library was generated using VAHTS Universal V6 RNA-seq Library Prep Kit for Illumina ^®^ (NR604-01/02), according to the manufacturer’s recommendations. Index codes were added to attribute sequences to each sample. Eligible libraries were sequenced on the Novaseq 6000 (Illumina) platform, generating 150 base paired-end reads.

### Transcriptome analysis of bulk RNA-seq data

The sequencing quality was evaluated by fastqc software, and low-quality reads were filtered by the Trimmomatic tool [35]. After preprocessing, clean reads were aligned to the human hg38 reference genome with Hisat2 software [36]. Gene expression levels were quantified using StringTie [37], with unexpressed or lowly expressed genes filtered out. Batch effects were corrected using ComBat-seq through the sva R package [38] with default parameters. Principal component analysis (PCA) was performed to visualize clustering between case and control samples based on the transcriptome data. Differentially expressed genes (DEGs) between case and control samples were identified using the DESeq2 package in R [39]. DEGs were selected with adjusted *P*-value < 0.05 and |log2 fold change (log2 FC)| > 1. DEGs were visualized using volcano plot and heatmap created with the R packages ‘ggplot2’ and ‘pheatmap’, respectively.

### Functional annotation and protein-protein interaction networks

To predict the biological processes involved in the pathogenesis of CPAM for those DEGs, Gene Ontology (GO) annotation was performed using the clusterProfiler R package [40]. Adjusted *P*-value of < 0.05 was considered as a significant event. Gene Set Enrichment Analysis (GSEA) was conducted to elucidate molecular mechanism at the whole transcriptome level by comparing the case and controls against the KEGG pathways database. Protein-protein interaction (PPI) based on DEGs enriched in the significant GO terms were predicted by STRING [41]. The PPI network was visualized using Cytoscape software [42].

### Identification of gene modules for distinguishing CPAM from control

To identify genes specific to CPAM, iterative weighted gene correlation network analysis (iWGCNA) (https://github.com/cstoeckert/iterativeWGCNA) was used to construct a co-expression network based on the transcriptome datasets and detect modules or clusters that comprise with highly correlated genes within the network [43]. Random forest analysis with 100 iterations of cross-validation was applied to rank the CPAM-related gene modules in descending order based on the mean decreased Gini index in iWGCNA. For each gene module, an eigengene was calculated and compared between CPAM and control groups, representing a normalized expression value derived from all genes within the module. Correlation coefficients between each module and clinical characteristics were also computed based on the eigengene.

### scRNA-seq analysis of CPAM patient

scRNA-seq data from type II-III CPAM samples was obtained from Zhang’s published paper [34], and was analyzed with Seurat package (https://github.com/satijalab/seurat). Cells expressing fewer than 200 or more than 2500 genes were filtered out, and cells expressing over 20% of genes derived from the mitochondrial genome were removed. Expression data were normalized and scaled by NormalizedData and ScaleData function in Seurat. Clustering was performed using the k-nearest neighbor method with the Findclusters function and visualized using t-distributed stochastic neighbor embedding (t-SNE) with the RunTSNE function. Marker genes for each cell cluster were identified by the FindAllMarkers function. The expression profiles of these markers in epithelial cells were normalized using Z-score as signature score and was compared between CPAM cases and controls in our bulk RNA-seq data by Wilcoxon test. *P*-value threshold was set as 0.05.

### Analysis of cell-cell communication

We conducted quantitative inference and analysis of cellular communication networks using CellChat [44] based on CPAM scRNA-seq data. Seurat data was input into CellChat (https://github.com/sqjin/CellChat/; accessed on 01/07/2022) to identify potential ligand-receptor interactions using the default parameters. Human CellChatDB, including 1,939 validated interactions, served as the interaction database. This analysis encompassed cellular interactions and communication categorized by secreted signaling (61.8%), extracellular matrix-receptor interactions (21.7%), and cell-to-cell contact genes (16.5%). The aggregated cell-cell communication network was established by quantifying interactions and visualized in a circular plot format. Following this, we identified significant cell-cell communication facilitated by interactions. CellChat provided insights into communication patterns among different epithelial cell subtypes, evaluating the number of incoming and outgoing communication patterns using Cophenetic and Silhouette metrics. Prominent ligands involved in outgoing or incoming communication signaling were identified and visualized using heatmaps and bar graphs. Pathway enrichment analysis for these ligands was conducted by EnrichR tool [45] using WikiPathways 2021. Heatmaps displaying the top ten enrichment terms (lowest adjusted *P*-values) were generated with the web tool Morpheus [46]. Finally, the global communication patterns among cell types and signaling pathways were visualized with river plots.

### Quantitative real-time PCR

We performed experimental validation of the expression of *AGR3* (Anterior Gradient 3, Protein Disulphide Isomerase Family Member), *COL4A1* (Collagen Type IV Alpha 1 Chain), *COL4A2* (Collagen Type IV Alpha 2 Chain), *COL4A4* (Collagen Type IV Alpha 4 Chain), *EDN1*, *FPR2*, *CHMR1* in 6 cDNA samples from CPAM and matched controls from lung tissues using quantitative PCR (qPCR). The PCR primers targeting the coding regions of these genes were designed and synthesized by Ykang (Hangzhou, China). All qPCR reactions were conducted in a total of 10 µL volume, comprising 5 µL 2× SYBR Green I Master Mix (Promega), 1 µL 10 nM of each primer, and 2 µL of 1:20 diluted cDNA in 96-well plates with QuantStudio™ 7 Flex (Applied Biosystems). All reactions were performed in triplicate under the following conditions: initial denaturation at 95 °C for 5 min, followed by 40 cycles of 95 °C for 15 s and 60 °C for 30 s. Relative expression levels were calculated using the standard curve method normalized to the GAPDH housekeeping gene. Standard curves for these genes and *GAPDH* were constructed using five-serial 4-fold dilutions of cDNA samples.

## Results

### Gene expression profiles of CPAM reveals deregulated transcriptional control

Six cystic tissues with type II CPAM as cases and fifteen normal tissues as controls were included into final analysis. As shown in Table S1, the average age at surgery for all subjects was 18 months, with type II CPAM patients averaging 17 months at the time of surgery.

After quality control, an average of 21,382,664 reads per sample was generated, with 97.13% of them mapped to the human reference genome. PCA plot was used to investigate the RNA-seq quality and heterogeneity in the transcriptomes across the dataset. The principal component 1 (PC1) revealed a striking separation between CPAM and control samples (Fig. S1A), while PC2 suggested a sex difference among these samples with approximate 14% of variance. Sex differences in gene expression have been documented in lung tissue [47], although the impact of these DEGs may be modest [48]. We investigated DEGs between female and male controls, identifying 52 up-regulated and 69 down-regulated genes in female (Fig. S1B). The GO analysis suggested that those up-regulated genes in females were mainly enriched in homophilic cell adhesion via plasma membrane adhesion molecules and cell-cell adhesion via plasma-membrane adhesion molecules related biological process, while those down-regulated genes were enriched in adaptive immune response, lymphocyte, B cell mediated immunity and immunoglobulin mediated immune response (data not shown), consistent with previous findings of sex-biased genes enrichment [47]. We further investigated these DEGs in the case and control set, identifying 29 differently expressed genes (Table S2). However, these findings may be influenced by the small number of male CPAM cases, with only one male case included in the analysis. GO analysis for these 29 genes did not reveal any significant signaling pathways or biological process. All CPAM and control samples were combined for comprehensive DEG analysis.

**Fig. 1:**
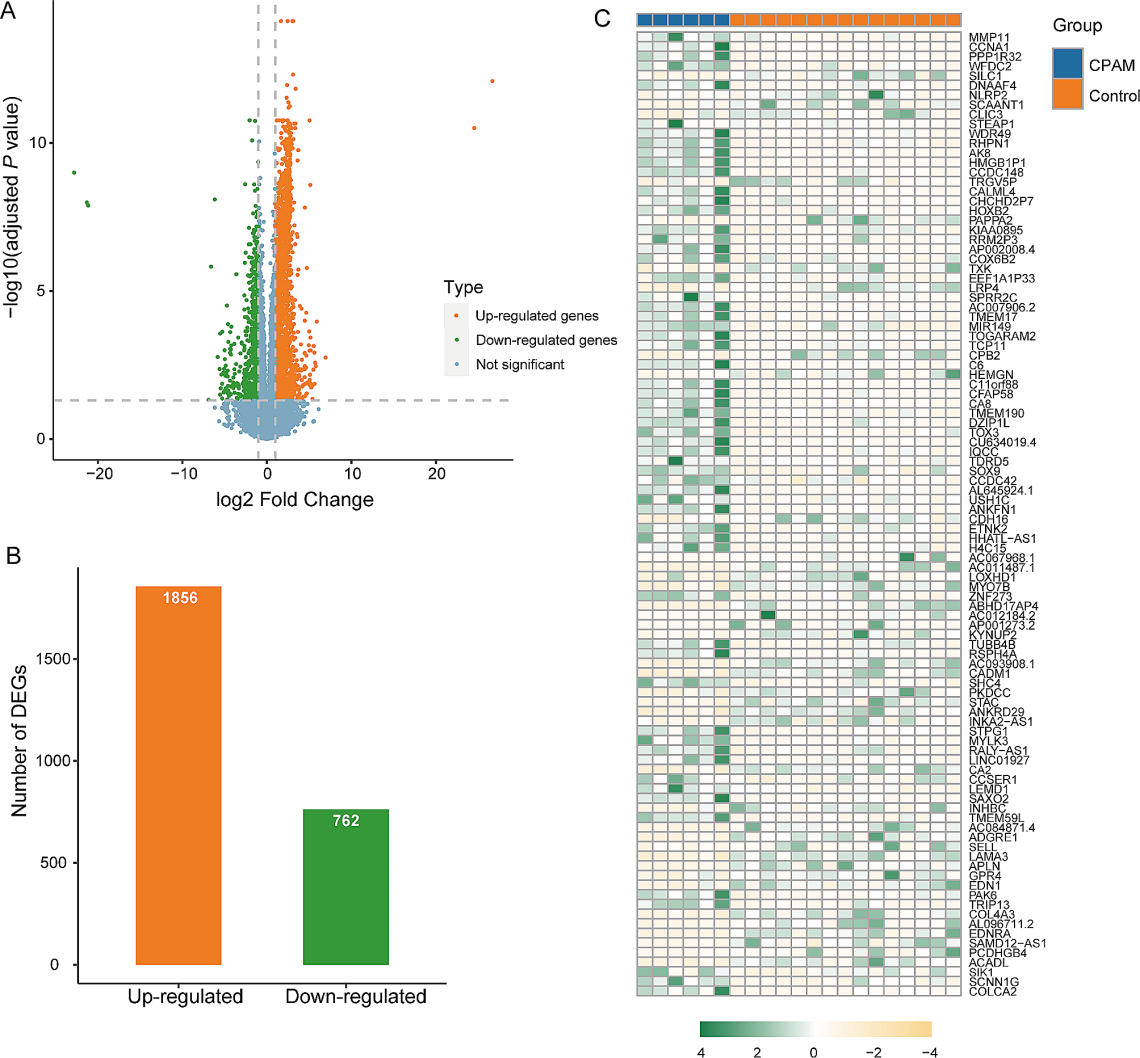
The differential expression of CPAM. (**A**) Volcano plot showed gene expression profiling between the two groups. Each point represents a gene, with vertical lines indicating a 2-fold change and the horizontal line corresponding to an adjusted *P*-value of 0.05. (**B**) The amount of up-regulated and down-regulated genes. (**C**) Heatmap of top 50 up-regulated and top 50 down-regulated genes

A total of 2,618 mRNAs met the criteria for DEGs based upon the adjusted *P*-value threshold of 0.05 and a fold change threshold of 2. Among these, 1,856 genes were up-regulated and 762 genes were down-regulated (Fig. 1A-B, Table S3). The heatmap depicting the top 50 up-regulated and top 50 down-regulated genes effectively visualizes these significant DEGs (Fig. 1C). Notably, among these top expressed genes, genes such as *SOX9* (SRY-Box Transcription Factor 9) and *MMP11*(Matrix Metallopeptidase 11) are involved in epithelial-mesenchymal transition (EMT) [49] which is involved in many different lung diseases [50].

### Functional variation of differentially expressed genes

**Fig. 2:**
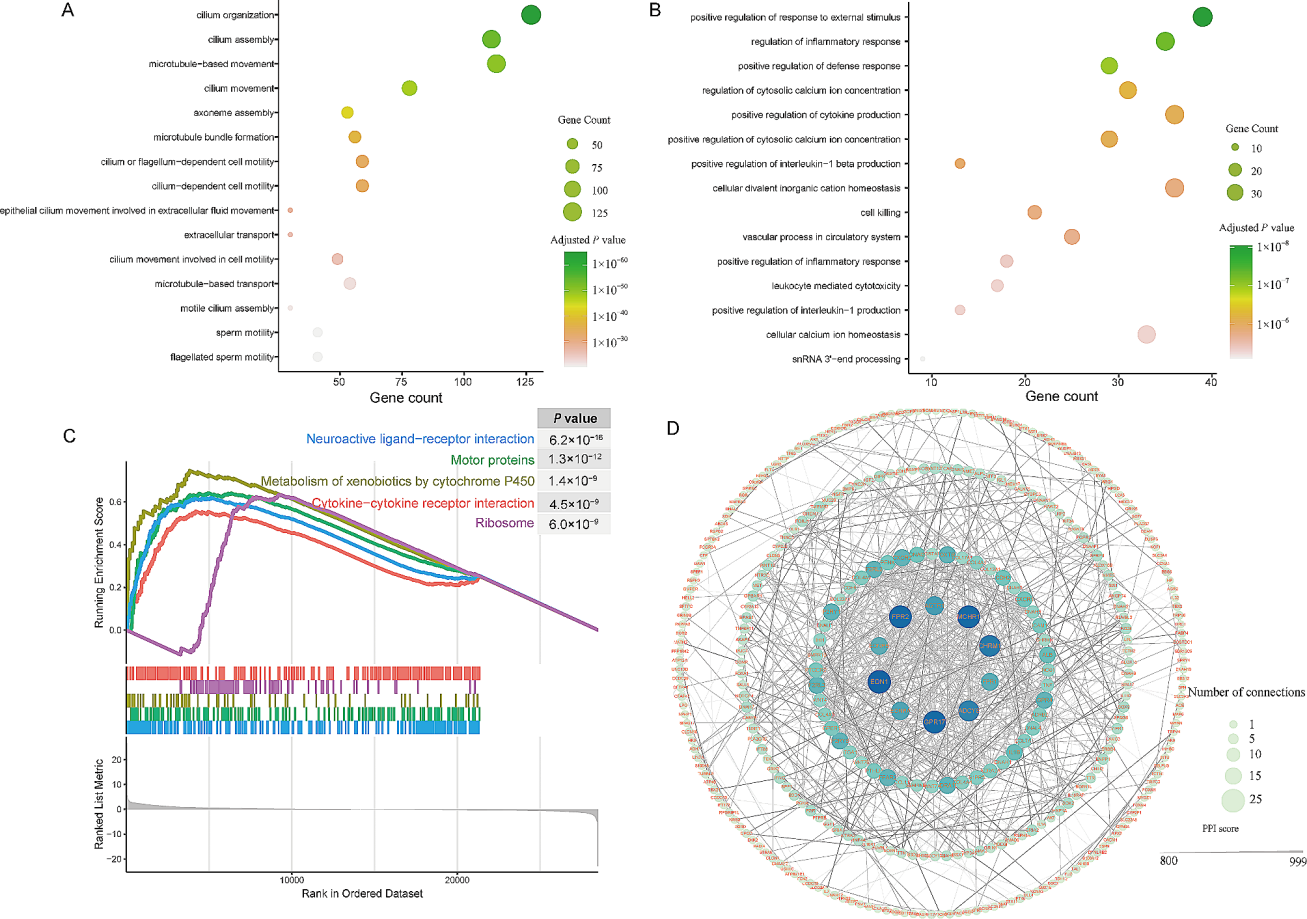
Functional annotation of DEGs. (**A**,** B**) GO enrichments of significantly up-regulated and down-regulated genes were displayed by top 15 significantly adjusted *P*-values, respectively. The size of the bubble indicated the adjusted *P*-value, and the color of the dot indicated the number of genes enriched in the GO term. (**C**) GSEA of whole transcription from RNA-seq, listing the top5 KEGG pathways. (**D**) Constructed PPI network of DEGs. Nodes represent proteins, and the size of nodes indicates the number of interactions. The edges denote the interactions between two proteins, and the width of an edge denotes the PPI score of the interaction

To further investigate the DEGs, we performed GO enrichment analysis. The results showed that the up-regulated genes were markedly enriched in several biological processes related to cilia, including cilium organization, cilium assembly, microtubule-based movement, cilium movement, axoneme assembly, microtubule bundle formation, cilium or flagellum-dependent cell motility, cilium-dependent cell motility, and epithelial cilium movement involved in extracellular fluid movement (Fig. 2A, Table S4). These DEGs also exhibited enrichment in molecular functions such as antigen binding, immunoglobulin receptor binding, and cellular components such as immunoglobulin complex and motile cilium (Table S5). The enrichment indicated the potential roles for cilia in the CPAM, possibly involving signaling transduction or extracellular fluids movement [51], which is consistent with recent transcriptome studies highlighting aberrant ciliary functions in its development [52, 53].

Conversely, down-regulated genes were enriched in processes related to positive regulation of response to external stimulus, regulation of inflammatory response, positive regulation of defense response, regulation of cytosolic calcium ion concentration, and positive regulation of cytokine production (Fig. 2B, Table S4). These down-regulated genes also showed enrichment in molecular functions such as immune receptor activity and carbohydrate binding, as well as cellular components such as integrator complex and secretory granule membrane (Table S5). The dysregulated immune response may contribute to epithelium damage and subsequent airway remodeling [54], which can lead to chronic inflammation and recurrent pulmonary infection in patients with CPAM [55].

Additionally, we utilized GSEA to identify enriched hallmark gene sets based on KEGG pathways. Notably, the genes in type II CPAM were significantly expressed in pathways related to neuroactive ligand-receptor interaction, featuring receptors located on the cell plasma membrances such as *GRIN3B* (Glutamate Ionotropic Receptor NMDA Type Subunit 3B), *LPAR3* (Lysophosphatidic Acid Receptor 3), *GLRB* (Glycine Receptor Beta), and *GPR156* (G Protein-Coupled Receptor 156), which facilitate the signal transduction from extracellular environment into cells [56]. Other enriched pathways included those involving motor proteins, metabolism of xenobiotics by cytochrome P450, and cytokine-cytokine receptor interaction which is associated with inflammation and immune response [57] (Fig. 2C, Table S6).

A total of 867 DEGs that were significantly enriched in GO terms were uploaded to the STRING database to build a PPI network. The cutoff criterion for PPI inclusion was set at a stringent PPI score of > 800. Subsequently, the resulting PPI network, composed of 341 nodes and 704 interactions, was visualized using Cytoscape software (Fig. 2D). The top five DEGs that were determined based on their degree of interaction were listed as follows: *EDN1* (Endothelin 1) (degree = 25), *MCHR1* (Melanin Concentrating Hormone Receptor 1) (degree = 24), *GPR17* (G Protein-Coupled Receptor 17) (degree = 24), *FPR2* (Formyl Peptide Receptor 2) (degree = 24), and *CHRM1* (Cholinergic Receptor Muscarinic 1) (degree = 22).

### Gene modules distinguish CPAMs from controls

**Fig. 3:**
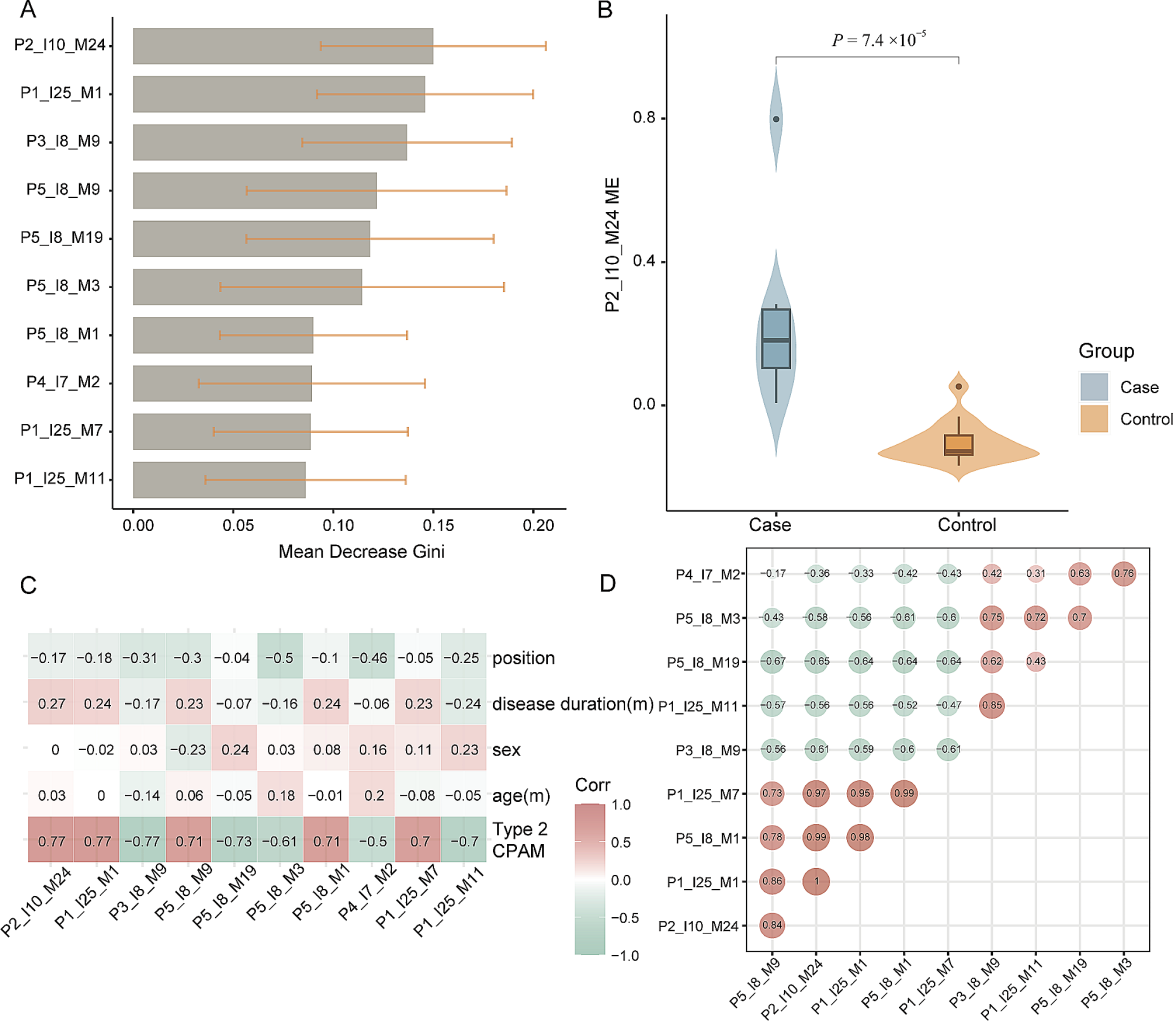
Identification of essential gene modules for CPAM discrimination. (**A**) Top10 modules ordered by the mean decreased Gini for distinguishing CPAM from control. (**B**) P2_I10_M24 ME in cases and controls (Wilcoxon test, *P* = 7.4 × 10 ^− 5^). (**C**) The correlation of top10 module ME and clinical parameters. Numbers in each box represent correlation coefficients, only *P* values that are significant are displayed by *. ‘***’ represented the *P* < 0.001. (**D**) Inter-modular correlation of top10 modules

As a result of iWGCNA, 158 gene modules were identified using the random forest algorithm and ranked based on the mean decreased Gini index. The top 10 gene modules that distinguished CPAMs from controls were presented in Fig. 3A, with module P2_I10_M24 occupying the highest position, followed by P1_I25_M1 and P3_I8_M9. Hub genes in module P2_I10_M24 such as *PIFO* (*CIMAP3*, Ciliary Microtubule Associated Protein 3), *CFAP221* (Cilia And Flagella Associated Protein 221), and *DNAH7* (Dynein Axonemal Heavy Chain 7), along with genes such as *DRC3* (Dynein Regulatory Complex Subunit 3), *RSPH4A* (Radial Spoke Head Component 4 A), *CFAP43* (Cilia And Flagella Associated Protein 43), *DNAH11*(Dynein Axonemal Heavy Chain 11), *DNAH2*, and *CFAP77* (Cilia And Flagella Associated Protein 77) in P1_I25_M1 module (Table S7), were found to be essential for ciliary ultrastructure, ciliary stability, and ciliary beating [58–63].

The module eigengene (ME), representing the expression value of P2_I10_M24, was significantly higher in CPAM cases than in controls (Fig. 3B, *P* = 7.4 × 10^− 5^), suggesting an up-regulation of genes in this module in CPAM. Notably, the top 10 modules were all correlated with type II CPAM but independent of factors such as age, sex, cystic position, and disease duration (Fig. 3C), further confirming that sex and other factors did not affect the analysis when all samples were combined. Among these modules, P2_I10_M24, P1_I25_M1, P5_I8_M9, P5_I8_M1, and P1_I25_M7 showed a positive correlation with CPAM, while the remaining modules were negatively correlated with the disease, indicating that both up-regulated and down-regulated genes could be associated with the development of CPAM (Fig S2) [52, 53, 64]. Furthermore, the CPAM related modules with a positive coefficient were strongly correlated each other (Fig. 3D, coefficient > 0.7), indicating these modules can effectively discriminate CPAM from control groups. We thus defined theses top modules as the ‘CPAM-associated module’ for distinguishing CPAMs from controls.

### Integrated analysis of RNA-seq and scRNA-seq from CPAM patients

**Fig. 4:**
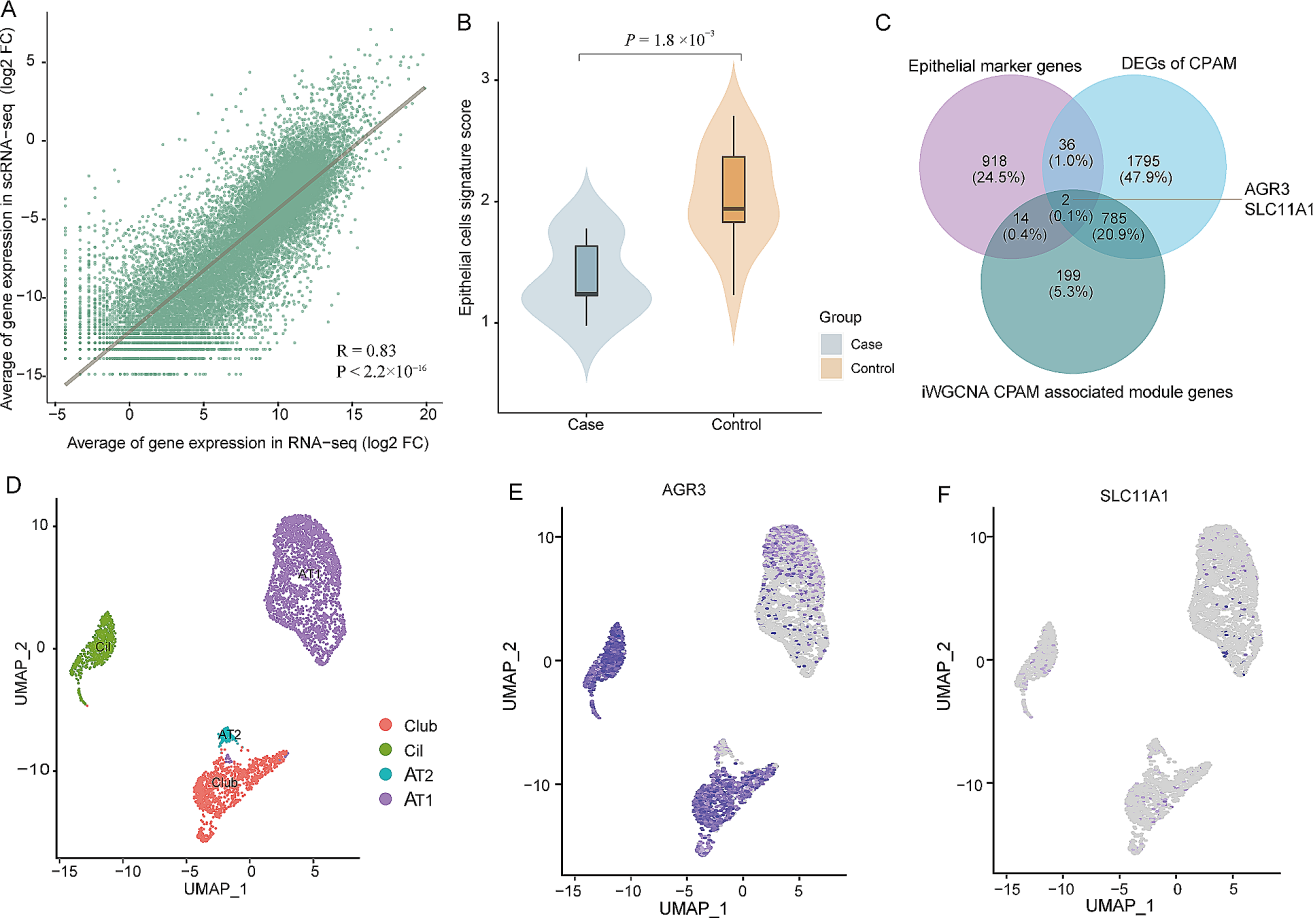
Integration of scRNA-seq and bulk RNA-seq analysis of CPAM. (**A**) Correlation between the RNA-seq and scRNA-seq in gene expression (log2 FC). The correlation coefficient was calculated by Pearson’s test (*R* = 0.83, *P* < 2.2 × 10^− 16^). (**B**) Comparisons of epithelial cell signature scores in CPAM case and control groups (*P* = 1.8 × 10 ^− 3^). *P*-value was obtained from two-sample t-test. (**C**) Venn diagram of DEGs of CPAM, the iWGCNA CPAM-associated module genes and epithelial cell marker genes. (**D**) Umap of subtypes for epithelial cells from CPAM patients. (**E**,** F**) Feature plot of *AGR3* and *SLC11A1* in epithelial cells

We obtained a total of 14, 814 cells for scRNA-seq analysis after quality control. A high consistency between the bulk RNA-seq and scRNA-seq data was observed (Fig. 4A, *P* < 2.2 × 10 ^− 16^). As shown previously, epithelial cells increased and mesenchymal cells decreased in cystic areas of CPAM [34]. The obtained cells could be divided into 9 cell types, including epithelial cells, monocytes, B-cells, fibroblasts, CD8 + T-cells, macrophages, neutrophils, endothelial cells, and NK cells [64]. Given that CPAM is characterized by the proliferation of distal bronchioles leading to the suppression of alveolar formation [65], our primary focus was on the epithelial cells, which are predominant in bronchi and alveoli. We identified 970 marker genes specific to epithelial cells. The signature scores of epithelial cells were calculated based on the normalized expression of each gene using a Z-score. Notably, the signature score of epithelial cells was significantly lower in CPAM cases than in controls (Fig. 4B, *P* = 1.8 × 10 ^− 3^), suggesting a disturbance in the regulation of epithelium-specific genes in CPAM. When we overlapped these 970 genes with DEG dataset from bulk RNA-seq data, 38 genes were differently expressed between CPAM and controls (Table S8). Additionally, we investigated these 970 genes within iWGCNA CPAM-associated modules and found 16 overlapping genes (Table S9). Among these 16 genes, *AGR3* and *SLC11A1* were the only two genes indicated in DEG dataset as well (Fig. 4C).

We further classified the epithelial cells into alveolar type 1 (AT1), alveolar type 2 (AT2), club cells (club), and ciliated cells according to the expression of marker genes (Fig. 4D and Fig. S3A, Table S10). As indicated in Fig. S3, marker genes of AT1 are primarily enriched in signaling pathways such as focal adhesion and ECM-receptor interaction (Fig. S3B). In contrast, marker genes of AT2 are implicated in pathways such as complement and coagulation cascades, phagosome, and pertussis (Fig. S3C). Marker genes of club and ciliated cells were associated with ribosome and motor proteins (Fig. S3D and Fig. S3E). As shown in Fig. 4E, *AGR3* is mainly expressed AT1, club, and ciliated cells, consistent with previous findings that *AGR3* is detected in multiciliated organs such as the colon and lung, specifically in the ciliated cells of the airway epithelium [66]. *SLC11A1* is mainly expressed in AT1 cells (Fig. 4F). *SLC11A1* is a proton/divalent cation antiporter that functions in host resistance to certain pathogens through regulating iron and manganese metabolism [67]. It also modulates proinflammatory cytokines and activation of innate lymphocytes [67]. Downregulated *SLC11A1* in CPAM might affect the immune balance and consequently impact lung epithelial development [68].

### CPAM-associated epithelial cells receive different communication patterns

We calculated the cell-cell communication network among the epithelial sub-cell types and visualized it using circle plots either by number of interactions (Fig. S4A, upper left) or by interaction weights (Fig. S4A, upper right) between two cell types. As shown in figure, signaling from AT1 cells was more active than that from AT2 cells, club cells, and ciliated cells, based on both interaction net number and interaction weight/strength (Fig. S4A). The results showed that cell-cell communication between AT1 cells and other cells was more pronounced.

**Fig. 5:**
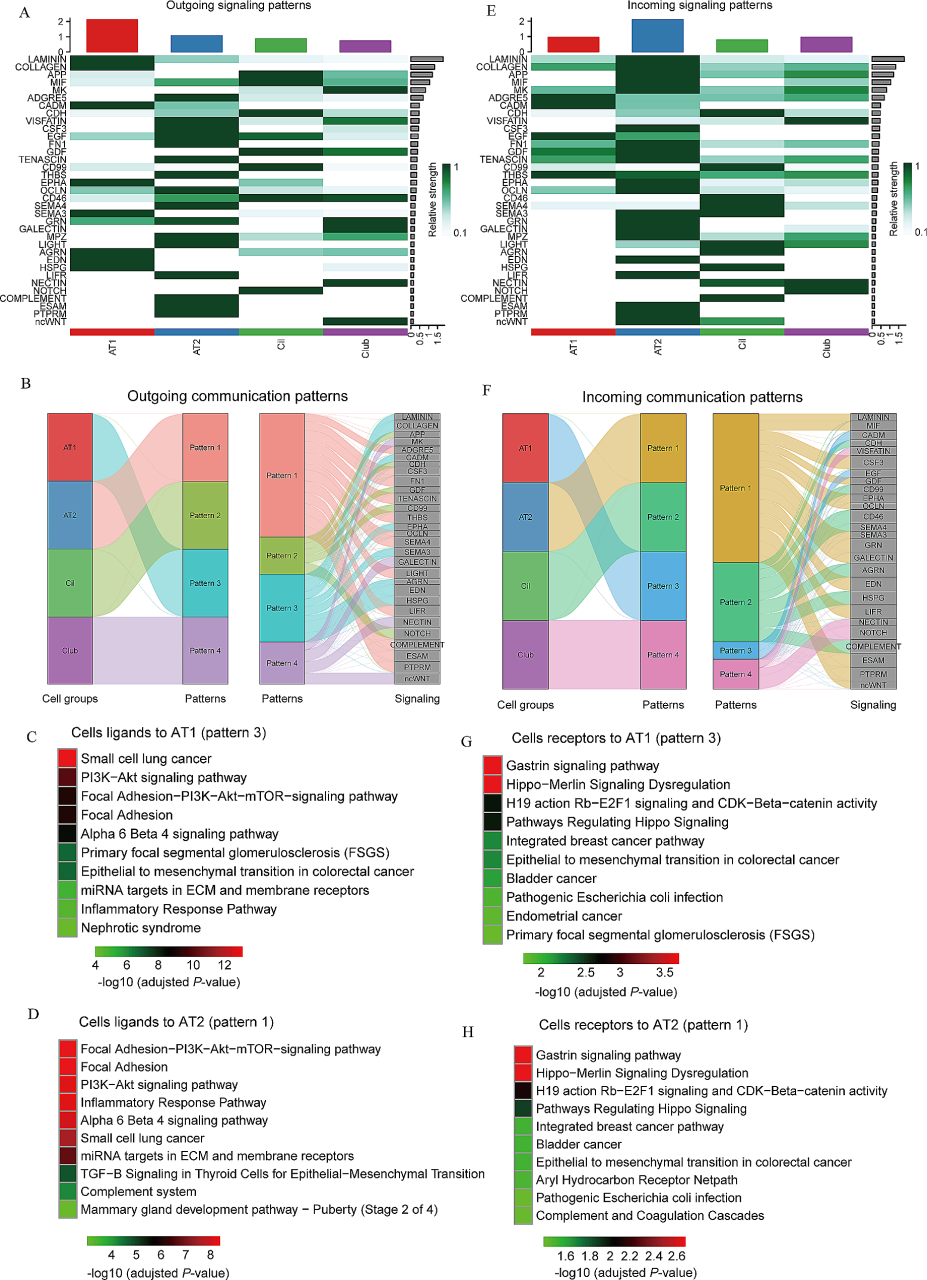
Outgoing and incoming communication patterns between CPAM-associated epithelial cells. (**A**) Outgoing patterns for signaling of AT1, AT2, club and ciliated cells. (**B**) Outgoing communication patterns of epithelial cell subtypes. The flow thickness represents the contribution of a signaling pathway to each mode of communication. (**C**,** D**) Pathways identified by outgoing communication pattern 3 for gene sets of AT1 ligands and receptors. The top 10 pathway terms were selected from WikiPathways 2021. (**E**) Incoming patterns for signaling of AT1, AT2, club and ciliated cells. (**F**) Incoming communication patterns of epithelial cell subtypes, which reveals the correspondence between the inferred patterns and cell groups, as well as signaling pathways. (**G**,** H**) Pathways identified by incoming communication pattern 1 for gene sets of AT2 ligands. The top 10 pathway terms were selected from WikiPathways 2021

We also explored the communication patterns to understand how these epithelial sub-cell types and signaling pathways coordinate. Notably, AT1 cells emerged as the major contributors to outgoing signals (Fig. 5A). Signaling molecules such as LAMININ, COLLAGEN, APP, MIF, and MK exhibited significant involvement in outgoing cellular interactions (Fig. 5A). We identified the correspondence between inferred latent communication patterns to decode outgoing communication patterns. Four distinct signaling patterns were identified (Fig. S4B), each uniquely associated with specific epithelial cell subtypes that predominantly contributed to outgoing communication: pattern #1 (AT2 cells), pattern #2 (Cil cells), pattern #3 (AT1 cells), and pattern #4 (Club cells) (Fig. 5B). In order to identify the pathways specifically associated with the outgoing communication of AT1, we selected a list of ligands (Table S11) that exert the most influence on communication pattern 3 (Fig. 5B). We found that the ligands to AT1 were mostly enriched in pathways such as PI3K-AKT signaling, focal adhesion, and epithelial to mesenchymal transition (Fig. 5C). The receptors of AT1 cells were enriched in pathways such as gastrin signaling pathway and epithelial to mesenchymal transition (Fig. 5D).

We also identified contributors to incoming signals within cell populations. By evaluating ligands overexpressed across all epithelial cells, we confirmed that AT2 cells were the foremost recipients of incoming signaling (Fig. 5E). Notably, LAMININ, COLLAGEN, APP, MIF, and MK emerged as the most significant molecules involved in incoming signaling (Fig. 5E). Our analysis revealed four distinct incoming signaling patterns (Fig. 5F and Fig. S4C), with AT2 cells exhibiting strong associations with signaling pattern #1 (Fig. 5F). Notably, the ligands interacting with AT2 cells, identified within communication pattern #1 (Fig. 5F), were enriched for processes related to focal adhesion, PI3K-AKT signaling pathway, inflammatory response pathway, and epithelial to mesenchymal transition (Fig. 5G). Furthermore, epithelial to mesenchymal transition was also one of the signaling pathways predominantly enriched in AT2-specific receptors (Fig. 5H). These results suggest that the genes involved in PI3K-AKT signaling pathway and epithelial to mesenchymal transition play a crucial role in the development of CPAM [69] (Table S12).

### Top ligand and receptor interactions between epithelial cell subtypes

**Fig. 6:**
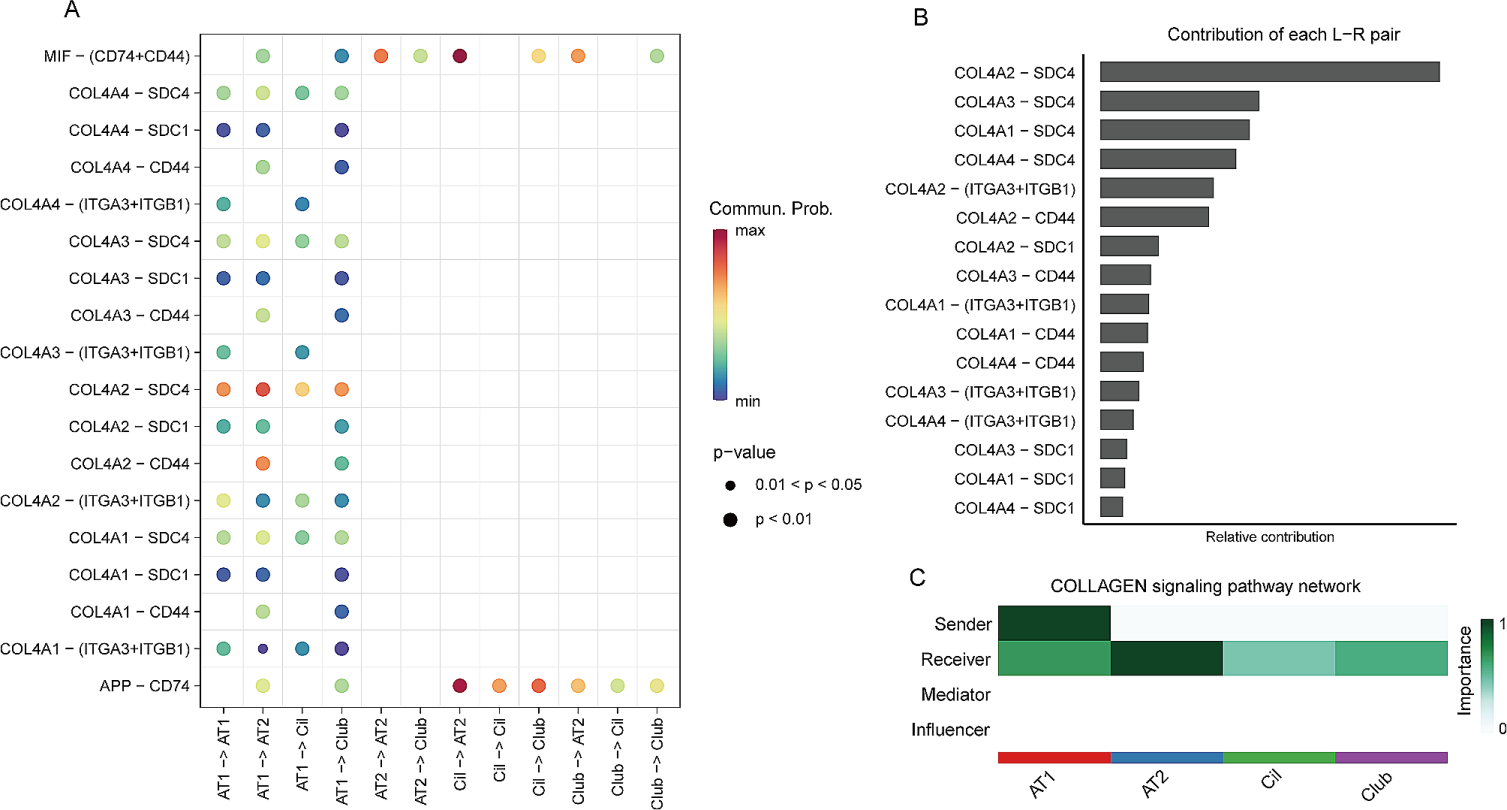
Top interactions between epithelial cells. (**A**) The key ligand-receptor pairs between AT1, AT2, club, and ciliated cells. (**B**) The contribution of each ligand-receptor pair to the COLLAGEN signaling pathway. (**C**) Identification of the COLLAGEN signaling pathway roles of cell groups.

The numerous ligand and receptor interactions among epithelial cells indicated the complexity of both outgoing and incoming signaling (Fig S5). Thus, to explore the top interactions involved in CPAM, we selected the signaling between AT1, AT2, club, and ciliated cells with a communication probability > 0.05. Among these, we found some strong interactions, such as MIF (ciliated cell) and CD74 + CD44 (AT2), APP (ciliated cell) and CD74 (AT2) (Fig. 6A). Notably, collagen-related ligands and their receptors, including *COL4A1*, *COL4A2*, and *COL4A4*, which were activated in the epithelial to mesenchymal transition process and involved in PI3K-AKT signaling pathways [49], were the most predominant ligand-receptor pairs mediating interaction between or within AT1 cells and AT2, cilial or club cells (Fig. 6A and B). AT1 cells serves as the senders in this COLLAGEN signaling network (Fig. 6C). Furthermore, these genes were significantly decreased in our CPAM II cysts samples (Table S3, Fig S6), suggesting that disruption of these genes may impair the PI3K-AKT signaling pathways, resulting in an abnormal EMT process and consequently leading to the formation of CPAM.

### Validation of gene expression

To confirm the RNA-seq data, we analyzed the mRNA expression levels of *AGR3*, *COL4A1*,* COL4A2*, *COL4A4*, *EDN1*, *FPR2*, and *CHMR1* in 10 tissue samples from CPAM and control groups using qPCR (Fig. S6). The mRNA expression level of *AGR3* was significantly higher in CPAM patients than in control tissues. Conversely, *COL4A1*,* COL4A2*, *COL4A4*, *EDN1*, *FPR2*, and *CHMR1* were significantly downregulated in CPAM patients. The qPCR results showed good consistency with the RNA-seq data.

## Discussion

Increasing evidence suggests the distinct mechanisms underlying different type of CPAMs. Type II CPAMs is believed to result from bronchial atresia or obstruction [70], with evidence indicating focal lung development arrest during the the pseudoglandular stage of gestation (5–17 weeks) [5]. Unlike other types of CPAM associated with specific genetic mutations, no genetic alterations have been identified in type II CPAM. Therefore, investigating the pathogenesis of CPAM type-specifically is crucial for developing effective diagnostic and prognostic assessments. In this study, we focus on exploring the transcriptome expression and cell-cell communications of type II CPAM using RNA-seq and single-cell RNA-seq technologies.

Previous research has identified aberrantly expressed genes and proteins in both fetus and postnatal type II CPAM [5, 24, 71]. Consistent with this, our data revealed significant upregulated and downregulated DEGs between postnatal CPAM cystic and control tissues. Notably, *SOX9* and *SOX2* (SRY-Box Transcription Factor 2) are predominantly expressed in CPAM cysts. In human developing lungs, SOX9 + progenitors are enriched in distal epitheliual tips [72], giving rise to alveolar and airway lieages [73]. *SOX2* is localized in the proximal epithelium [72], with proximal-distal patterning regulated by PI3K signaling critical for lung morphpogenesis [72].

It has been demonstrated that reduced expression of *SOX2* is crucial for regulating branching morphogenesis [25]. Ectopic expression of *Sox2* in epithelial cells has been shown to prevent airway branching morphogenesis [24, 25]. Our result corroborate previous studies that exclusively detected *SOX2* expression in type II CPAM but not in control or type I CPAM [24], highlighting the pivotal role of *SOX2* in the development of type II CPAM. This finding also suggests the possibility of distinct origins for type I and type II CPAM.

Previous studies have highlighted the essential role of *FGF10* (Fibroblast Growth Factor 10), its receptor *FGFR2b*, and inhibitor *SHH* (Sonic Hedgehog Signaling Molecule) in the pathogenesis of CPAM [16, 17, 20]. However, our investigation did not reveal any significance in the expression of these genes between the type II CPAM cysts and control samples. This observation is consistent with the findings from studies involving postnatal tissues from type II CPAM and normal controls [16], suggesting that these genes likely exert their essential roles during lung development rather than in the developed CPAM tissues.

Our GO analysis revealed that the top up-regulated transcripts in CPAM were significantly enriched in cilium-related biological process, including cilium assembly and movement. This suggests a potential impairment in cilia function in CPAM patient, which is critical for normal mucociliary clearance in the airway, essential for protecting against various insults. The result is consistent with our previous and other enrichment analysis of RNA expression changes in CPAM [52, 53]. Decreased *SMAD6* in our type II CPAM cysts was observed but TGF-β signaling pathway was not enriched in our dataset, suggesting that expression profiles may vary depending on the CPAM type [53]. This observation aligns with the pathological finding that type II CPAM cysts are typically lined by ciliated cuboidal epithelium. The top down-regulated genes were enriched in inflammatory response process, suggesting disrupted immune response regulation in cysts with CPAM and a potential presence of chronic inflammation, as supported by histological signs of inflammation in CPAM cysts [74]. Such imbalances in immune process may lead to difficulties in effectively mounting a defense against respiratory pathogens and repairing damage caused by infections, potentially contributing to the increased frequency of respiratory infections in children with CPAM. Furthermore, consistent with previous enrichment analysis, our KEGG analysis demonstrated significant enrichment in gene sets related to cytokine–cytokine receptor interaction pathway and neuroactive ligand-receptor interaction [52], indicating that genes involved in cell growth, differentiation, or inflammation may play pivotal roles in the development of CPAM.

The PPI analysis showed that proteins with top connections are predominantly enriched in down-regulated genes. Among them, *EDN1*, *FPR2*, *GPR17*, and *CHRM1* are involved in G protein-coupled receptor (GPCR) signaling pathways, crucial for various cellular processes such as organ and embryonic development, cell differentiation, apoptosis, tissue homeostasis, and the immune. *EDN1*, the predominant isoform in pulmonary endothelial cells, binds to its receptor EDNRA, activating a diverse network of signaling pathways such as MAPK, PI3K-AKT, PKC pathways to promote cell proliferation, cell growth, and survival [75]. *FPR2*, the most promiscuous member of formyl peptide receptors, can modulate both pro- and anti-inflammatory response [76]. Studies on *Fpr2/3* knockout mice suggested increased susceptibility to infection, uncontrolled inflammation, and pulmonary dysfunction compared to wild-type animals [77]. *GPR17* negatively regulated pulmonary inflammation induced by house dust mite [78], while *CHRM1*, widely expressed in lungs, regulates bronchoconstriction, airway epithelial cell proliferation, and inflammation [79]. Moreover, *EDN1*, *GPR17*, *FPR2*, and *CHRM1* are involved in calcium ion homeostasis or transportation. Dysregulation of ion balance in airway epithelial cells may impair electrolyte and PH in airway liquid, which is important to maintain ciliary function and mucociliary clearance to protect the host from inhaled pathogens [80]. Consequently, it damages the ability of innate immunity in CPAM patients, resulting in respiratory distress or recurrent pneumonia.

Our iWGCNA analysis identified 10 modules that associated with CPAM cysts, which are either positively regulated or negatively regulated in CPAM. Specifically, ciliary formation or function related genes are indicated in some of these modules, suggesting their important roles in CPAM pathogenesis. Integration of these module genes with DEGs from bulk and epithelial marker genes highlighted *AGR3* and *SLC11A1*. *AGR3* is up-regulated in CPAM cysts and belongs to protein disulphide isomerase (PDI) family which regulates protein folding and calcium homeostasis. Studies on Agr3 knockout mice have shown reduced calcium-dependent ciliary beat frequency and impaired mucociliary clearance in the airway [66]. Moreover, *AGR3* was overexpressed in many tumor tissues and could promote the stemness of cancer cells by activating Wnt/β-catenin signaling [81]. Wnt signaling plays a crucial role in the developing lung by contributing to branching morphogenesis and airway formation [82]. Additionally, *AGR3* positively regulates proteins involved in airway epithelial junctions in chronic obstructive pulmonary disease [83], suggesting its role in disrupting normal cell-cell communication in CPAM cysts. On the other hand, *SLC11A1* is associated with the infectious and autoimmune diseases such as tuberculosis and type I diabetes mellitus [84, 85]. It modifies macrophage activation by regulating chemokine/cytokine genes such as tumor necrosis factor (TNF) and IL-1β [86]. Recent studies have revealed diversity of immune cells and their impact on epithelial maturation during lung development [68], where IL-1β plays a role in signal transduction in epithelial tip cells [68]. Down-regulation of *SLC11A1* in CPAM may disrupt IL-1β signal transduction, which in turn affects the expression of *SOX9* and *SOX2*, and consequently leading to abnormal immune-epithelial communication, resulting in lung anomalies. Further experimental investigations are warranted to elucidate the immune functions of *SLC11A1* in CPAM.

Increased cell proliferation and decreased apoptosis during lung development were attributable to the disruption of normal mesenchymal-epithelial interactions, leading to the CPAM [33, 87]. Indeed, focal asynchrony proliferation between epithelial and stromal components was indicated in CPAM [87]. Increased epithelial cells and decreased mesenchymal cells in CPAM cystic area were further confirmed by previous single-cell RNA-seq analysis [34]. We observed significant differences in gene signature scores between CPAM and control groups. Furthermore, both identified epithelial cell ligands and receptors were associated with epithelial to mesenchymal transition pathways, suggesting that signals involved in cell communication and interaction may play a role in the abnormal regulation of CPAM development abnormal regulation of developing of CPAM.

We reanalyzed scRNA-seq data [34] to infer and investigate epithelial cell–cell communications and interactions. We found AT1 and AT2 are the predominant communication cells for outgoing and incoming signals with specific signaling communication patterns. Key ligands involved in these interactions included LAMININ, COLLAGEN, SEMA3, and EDN. LAMININ and COLLAGEN are key components of the extracellular matrix (ECM) and actively mediate interactions between epithelium and mesenchyme during the airway branching process [88, 89]. However, we found the COLLAGEN related genes are decreased in our type II CPAM cyst samples, suggesting disrupted epithelium–mesenchyme interactions.

*SEMA3* and its receptor *NRP1* are essential during fetal pulmonary development; disruption of Sema3-Nrp1 signaling in transgenic *Nrp1*^Sema−^ mice has been shown to impair alveolar-capillary interface formation and result in severe parenchymal anomalies at birth [90, 91]. One isoform of *EDN*, frequently connected with other proteins based on our PPI analysis, can activate the PI3K-AKT pathway and is involved in cell migration. We found that ligands from AT1 cells and those targeting AT2 cells are predominantly enriched in the PI3K-AKT-mTOR-singaling pathway, indicating an important role for genes in this pathway in the pathogenesis of CPAM.

The PI3K–AKT–mTOR signaling pathway is essential to cell cycle, cell proliferation, migration, and apoptosis. Genes in this pathway have been extensively studied and are known to be activated in tumorigenesis and progression of disease [92]. Various novel inhibitors targeting this pathway have been identified in preclinical studies and clinical trials in human cancers [93]. In lung development, Khattar et al. observed that PI3K activation and phosphorylated ATK (pAKT) expression levels are higher in the proximal lung epithelium and distal branch tips of early developing embryonic mice than in distal lung epithelium at later stages. Conditional knockout of PI3K singaling in the developing lung epithelium resulted in impaired differentiation of airway epithelial cells [94]. Transcriptome analysis across several subtypes of congenital lung lesions, including CPAM, has indicated reduced gene expression in this pathway [69]. Additionally, three-dimensional cell culture studies using epithelial cells isolated from CPAM lesions have suggested intrinsic defects in epithelial function [69], highlighting the requirement of PI3K signaling for proper epithelial patterning during lung organogenesis. Drugs activating/blocking the ligands of the PI3K-AKT-mTOR signaling may be considered as therapeutic targets.

### Limitation

Although limited by a relatively small sample size, our bulk RNA-seq data has similar enrichment patterns to previous transcriptome results, suggesting a consistent and reliable gene expression profiles. This reinforces the validity and reliability of our findings. However, it remains challenging to conclusively determine from our postnatal CPAM samples whether these genes play a causal role in initial cyst formation or if their altered expression is a consequence of disease progression. Longitudinal studies tracking gene expression from early fetal development through postnatal stages would provide more dynamic insights into the pathogenesis of CPAM. Despite these limitations, the identified genes and signaling pathways are suggestive of association with cell proliferation, apoptosis, and EMT, all of which are relevant to cyst formation and lung development. To firmly establish a causative link between these genes and cyst formation, further functional studies are needed to elucidate the roles of these molecules and signal pathways in CPAM pathophysiology.

## Conclusion

In summary, our study systematically characterized the transcriptome expression pattern and identified gene models associated with type II CPAM. The PPI analysis highlighted several genes in GPCR signaling pathways and the regulation of calcium homeostasis. By integrating scRNA-seq and bulk RNA-seq data, we also identified calcium homeostasis-related gene AGR3 and the immune-related gene *SLC11A1*. We found that the PI3K-AKT-mTOR signaling pathway may play a crucial role in epithelial cell communication and interactions. The genes and signaling pathways highlighted inthis analysis may provide opportunities to explore new molecular mechanisms and develop therapeutic strategies for CPAM.

### Electronic supplementary material

Below is the link to the electronic supplementary material.

Supplementary Material 1: Supplementary Fig. 1 (A) PCA plot of RNA-seq data shows the characteristics of samples according to the gene expression levels. CPAM and control samples are colored in blue and orange, respectively. Each dot indicates a sample. (B) Volcano plot showed the expression profiling between the female and male in control samples. Each point in the plot indicated one gene, vertical lines refer to 2-fold change and the horizontal line corresponds to the adjusted P-value of 0.05.

Supplementary Material 2: Supplementary Fig. 2 Comparison of epithelial cell signature scores in each module between CPAM case and control groups. P-value was obtained from two-sample t-test.

Supplementary Material 3: Supplementary Fig. 3 (A) Feature plot of selected marker genes of AT1, AT2, club, and ciliated cells. (B-E) GSEA of the marker genes for AT1, AT2, club, and ciliated cells, respectively.

Supplementary Material 4: Supplementary Fig. 4 (A) Interaction net count plot and weight plot of epithelial cell subtypes. The thicker the line represented, the more the number of interactions, and the stronger the interaction weights/strength between the two cell types.(B,C) Cophenetic and Silhouette metrics were used to identify the number of outgoing and incoming communication patterns.

Supplementary Material 5: Supplementary Fig. 5 Ligand-receptor interactions between AT1, AT2, club, and ciliated cells inferred by CellChatDB.

Supplementary Material 6: Supplementary Fig. 6 qPCR validation of AGR3, COL4A1, COL4A2, COL4A4, EDN1, FPR2 and CHMR1 at mRNA level.

Supplementary Material 7: Supplemental Table 1 Clinical characteristics of subjects used for RNA-seq and qPCR validation.

Supplementary Material 8: Supplemental Table 2 Overlapped genes between DEGs related to CPAM and DEGs related to sex.

Supplementary Material 9: Supplemental Table 3 DEGs between CPAM cases and controls.

Supplementary Material 10: Supplemental Table 4 GO enrichment analysis (biological process, BP) of up-regulated and down-regulated genes.

Supplementary Material 11: Supplemental Table 5 GO enrichment analysis (molecular function, MF) and (cell component, CC) of up-regulated and down-regulated genes.

Supplementary Material 12: Supplemental Table 6 KEGG analysis of differentially expressed genes.

Supplementary Material 13: Supplemental Table 7 Gene list for the top 10 modules performed by iWGCNA.

Supplementary Material 14: Supplemental Table 8 Overlapped genes between epithelial cell marker genes and DEGs.

Supplementary Material 15: Supplemental Table 9 Overlapped genes between epithelial cell marker genes and the top10 module genes.

Supplementary Material 16: Supplemental Table 10 Marker genes of epithelial subtypes.

Supplementary Material 17: Supplemental Table 11 Inference and analysis of CPAM-associated epithelial cells communication from single-cell data using CellChat.

Supplementary Material 18: Supplemental Table 12 Ligands and receptors enriched in PI3K-AKT signaling pathway and epithelial to mesenchymal transition.

## Data Availability

The data that support the findings of this study are available from the corresponding author upon reasonable request.

## Abbreviations

AT1: Alveolar type 1
AT2: Alveolar type 2
CPAM: Congenital pulmonary airway malformation
DEG: Differentially expressed gene
FC: Fold change
GO: Gene ontology
GSEA: Gene set enrichment analysis
iWGCNA: Iterative weighted gene correlation network analysis
PCA: Principal component analysis
PPI: Protein-protein interaction
RNA: seq RNA sequencing
scRNA: seq Single-cell RNA-seq
