# Supplementary figures and images for: Integrative analysis of bulk and single-cell RNA sequencing reveals the gene expression profile and the critical signaling pathways of type II CPAM
Source: Cell Biosci. 2024 Jul 18;14:94. doi: 10.1186/s13578-024-01276-8 (PMC11264590; doi:10.1186/s13578-024-01276-8)

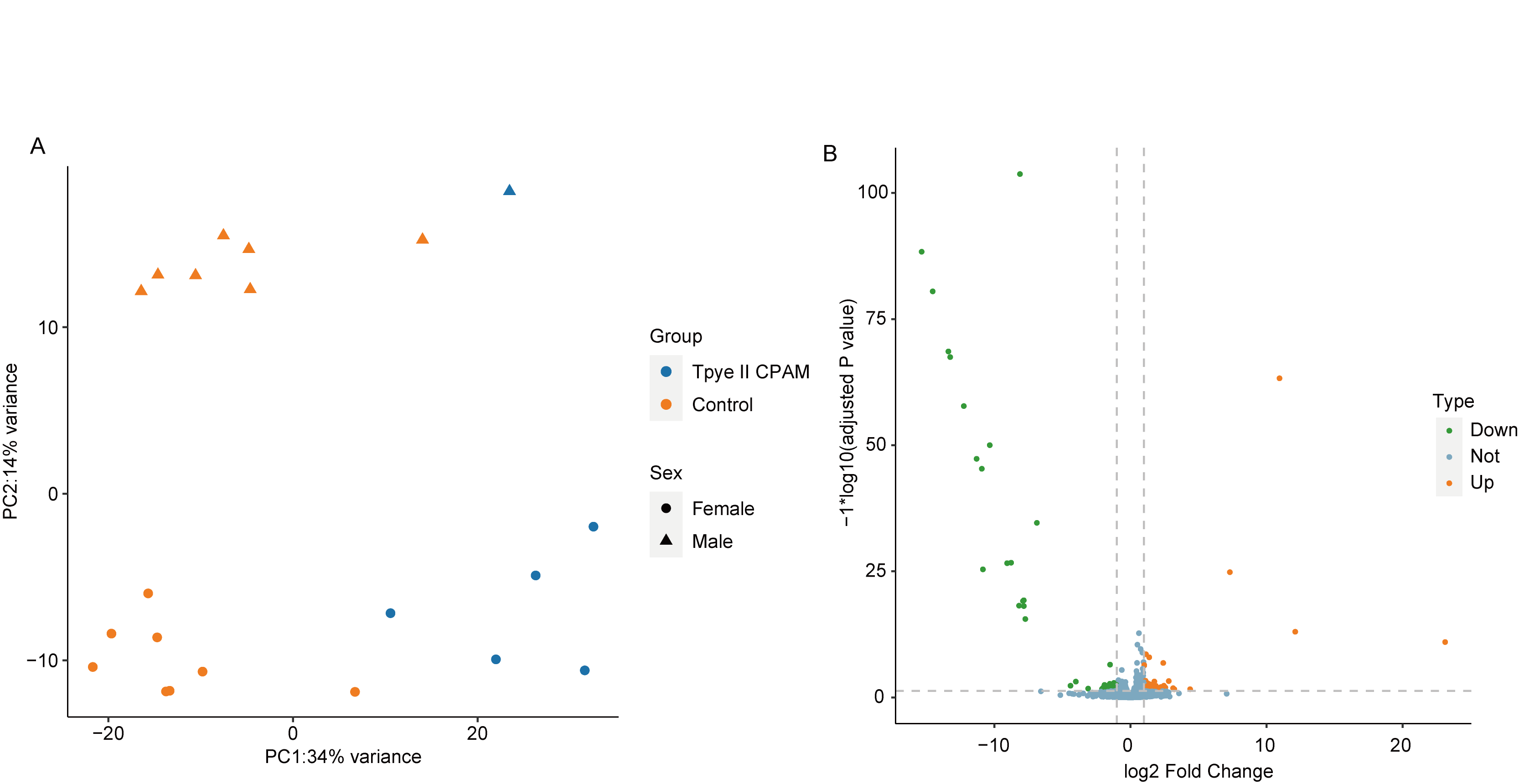

Supplement: Supplementary file 1 — Supplementary Material 1: Supplementary Fig. 1 (A) PCA plot of RNA-seq data shows the characteristics of samples according to the gene expression levels. CPAM and control samples are colored in blue and orange, respectively. Each dot indicates a sample. (B) Volcano plot showed the expression profiling between the female and male in control samples. Each point in the plot indicated one gene, vertical lines refer to 2-fold change and the horizontal line corresponds to the adjusted P-value of 0.05. [file 13578_2024_1276_MOESM1_ESM.png]

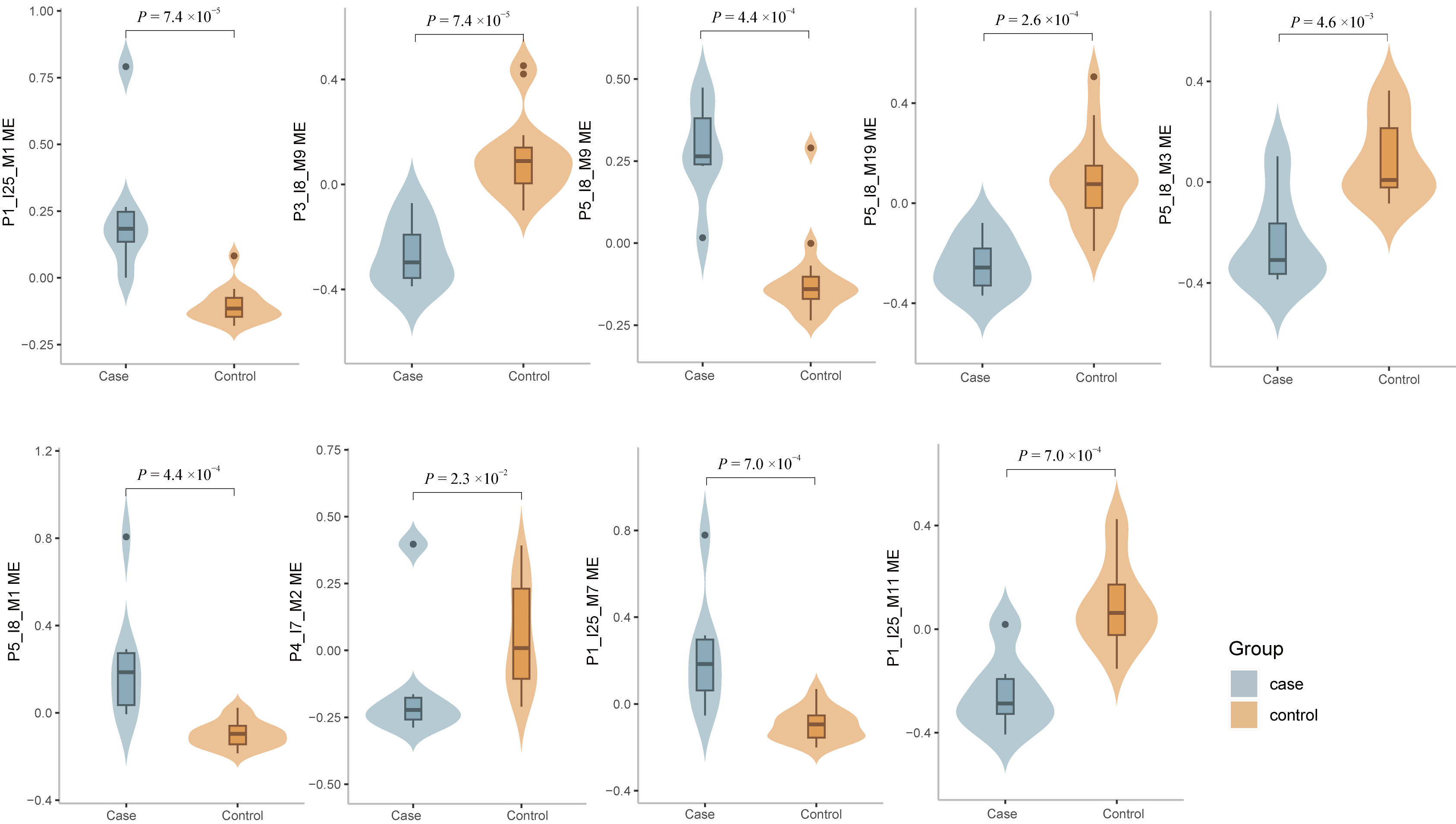

Supplement: Supplementary file 2 — Supplementary Material 2: Supplementary Fig. 2 Comparison of epithelial cell signature scores in each module between CPAM case and control groups. P-value was obtained from two-sample t-test. [file 13578_2024_1276_MOESM2_ESM.png]

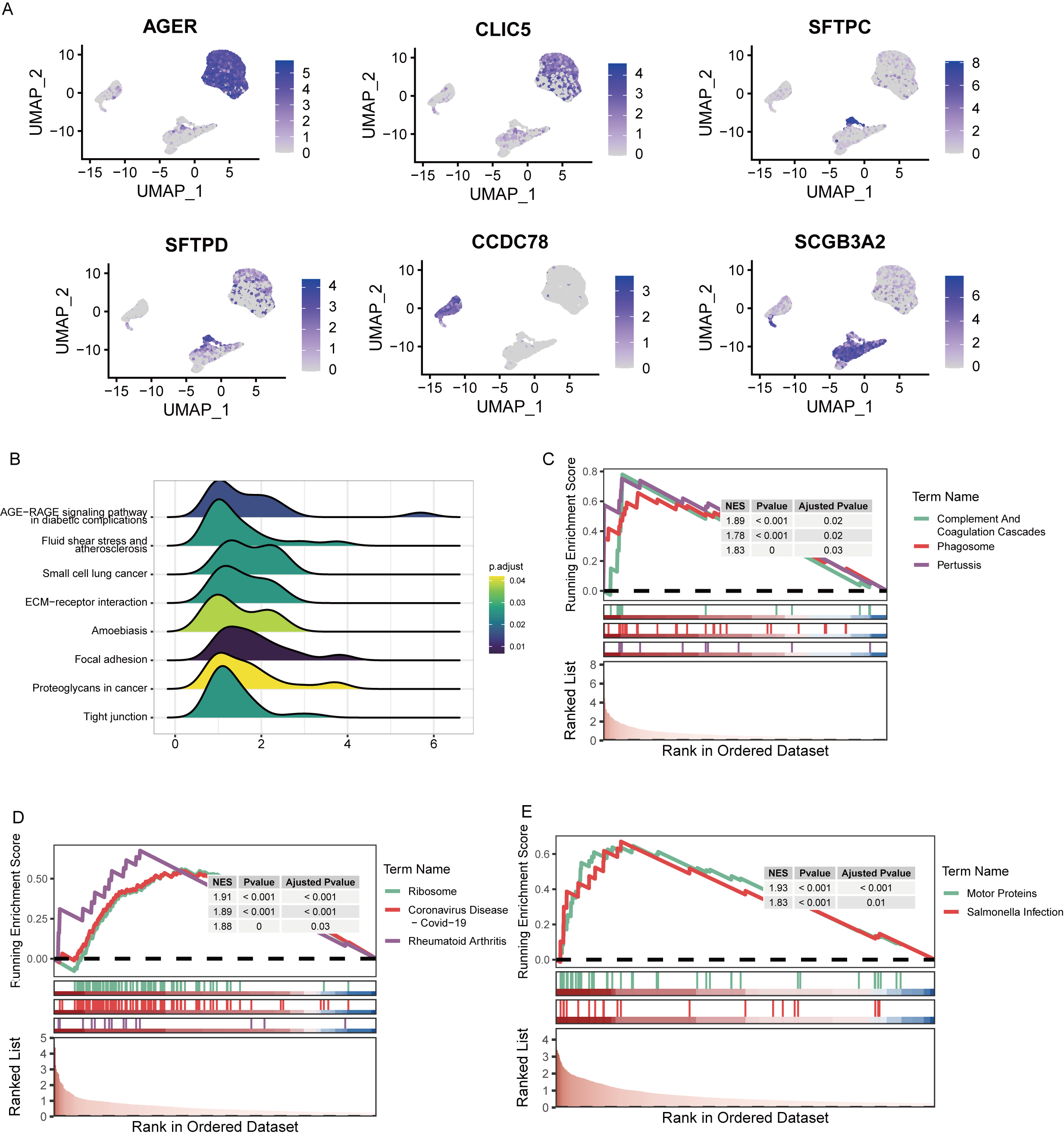

Supplement: Supplementary file 3 — Supplementary Material 3: Supplementary Fig. 3 (A) Feature plot of selected marker genes of AT1, AT2, club, and ciliated cells. (B-E) GSEA of the marker genes for AT1, AT2, club, and ciliated cells, respectively. [file 13578_2024_1276_MOESM3_ESM.png]

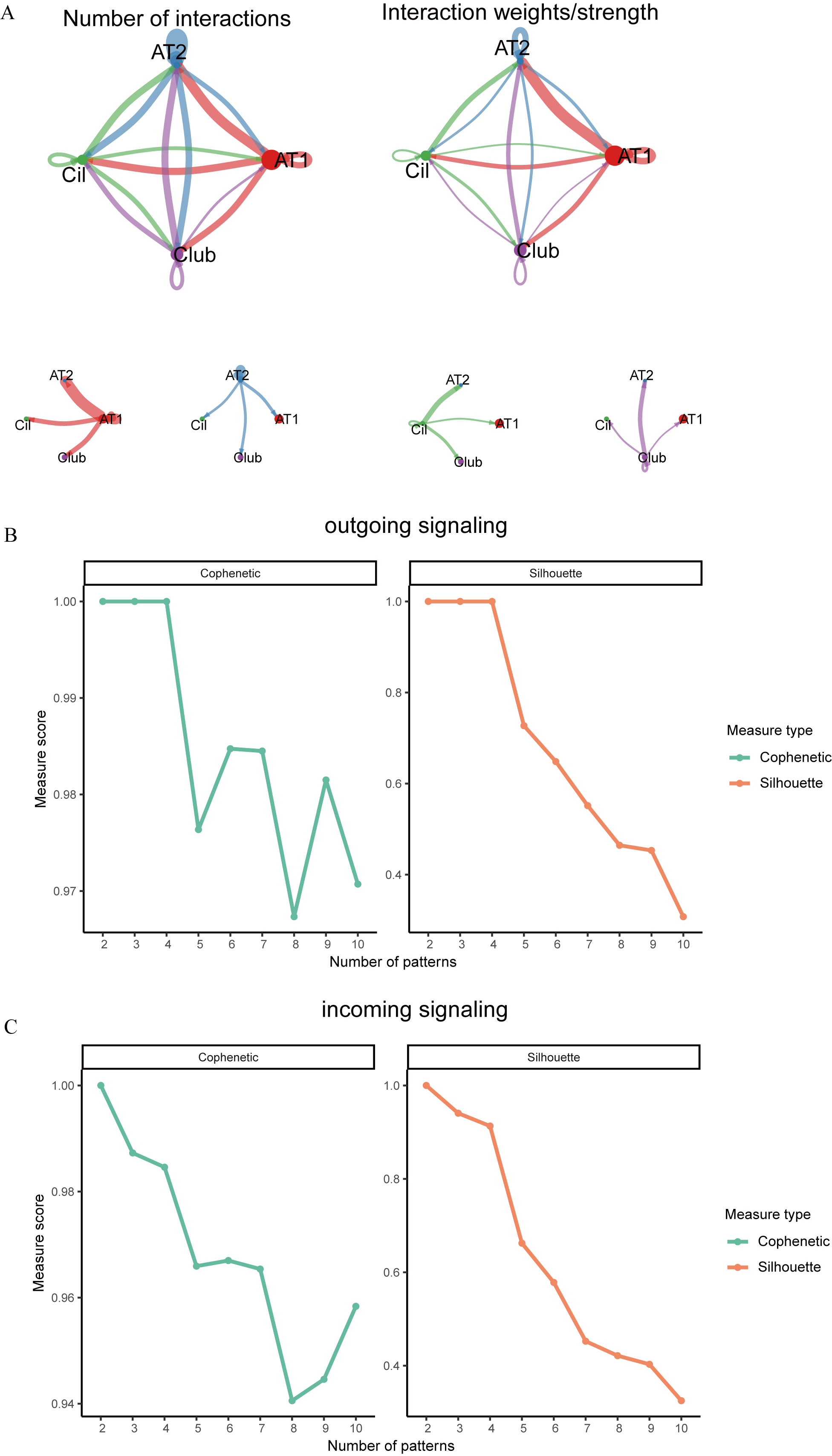

Supplement: Supplementary file 4 — Supplementary Material 4: Supplementary Fig. 4 (A) Interaction net count plot and weight plot of epithelial cell subtypes. The thicker the line represented, the more the number of interactions, and the stronger the interaction weights/strength between the two cell types.(B,C) Cophenetic and Silhouette metrics were used to identify the number of outgoing and incoming communication patterns. [file 13578_2024_1276_MOESM4_ESM.png]

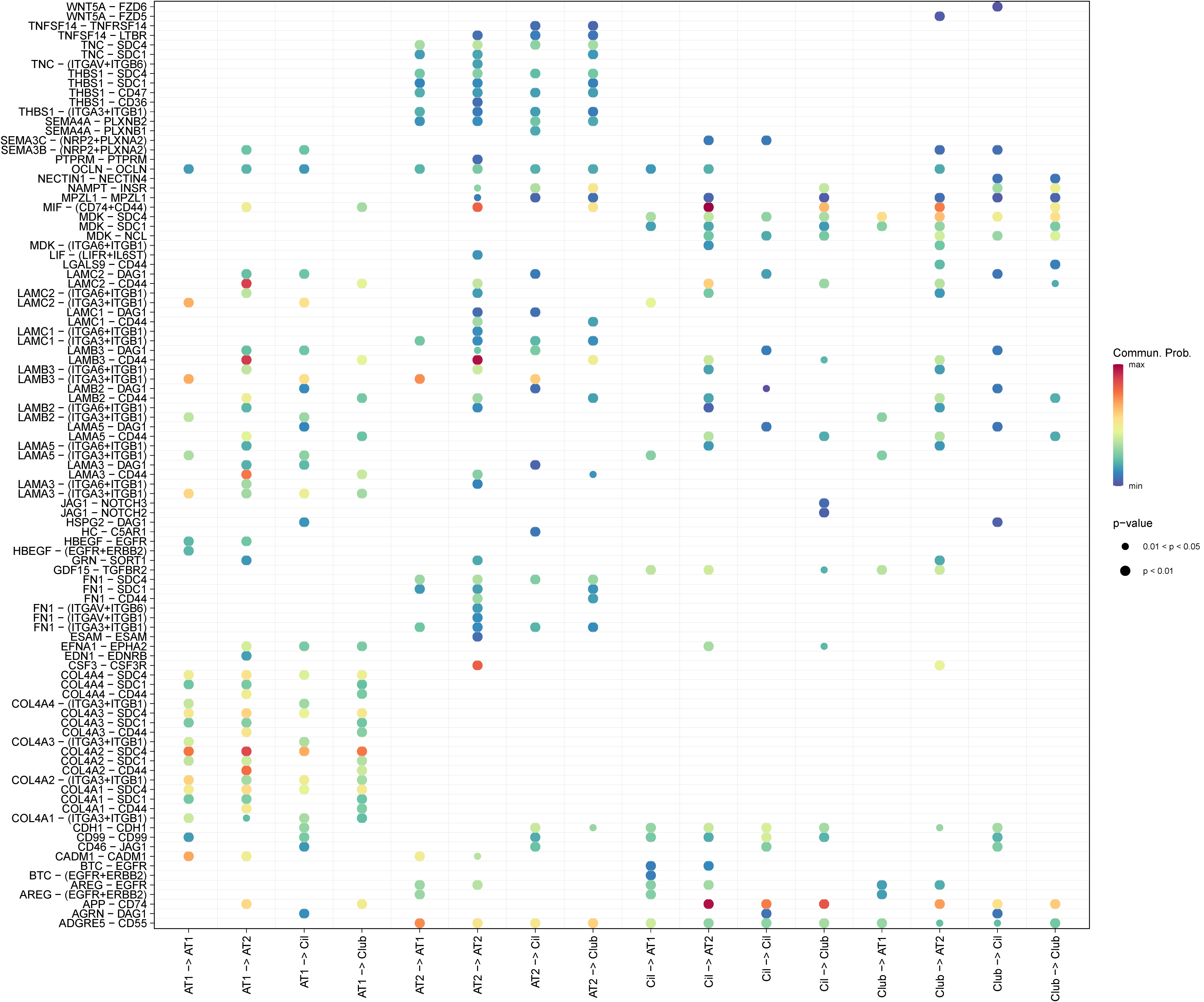

Supplement: Supplementary file 5 — Supplementary Material 5: Supplementary Fig. 5 Ligand-receptor interactions between AT1, AT2, club, and ciliated cells inferred by CellChatDB. [file 13578_2024_1276_MOESM5_ESM.png]

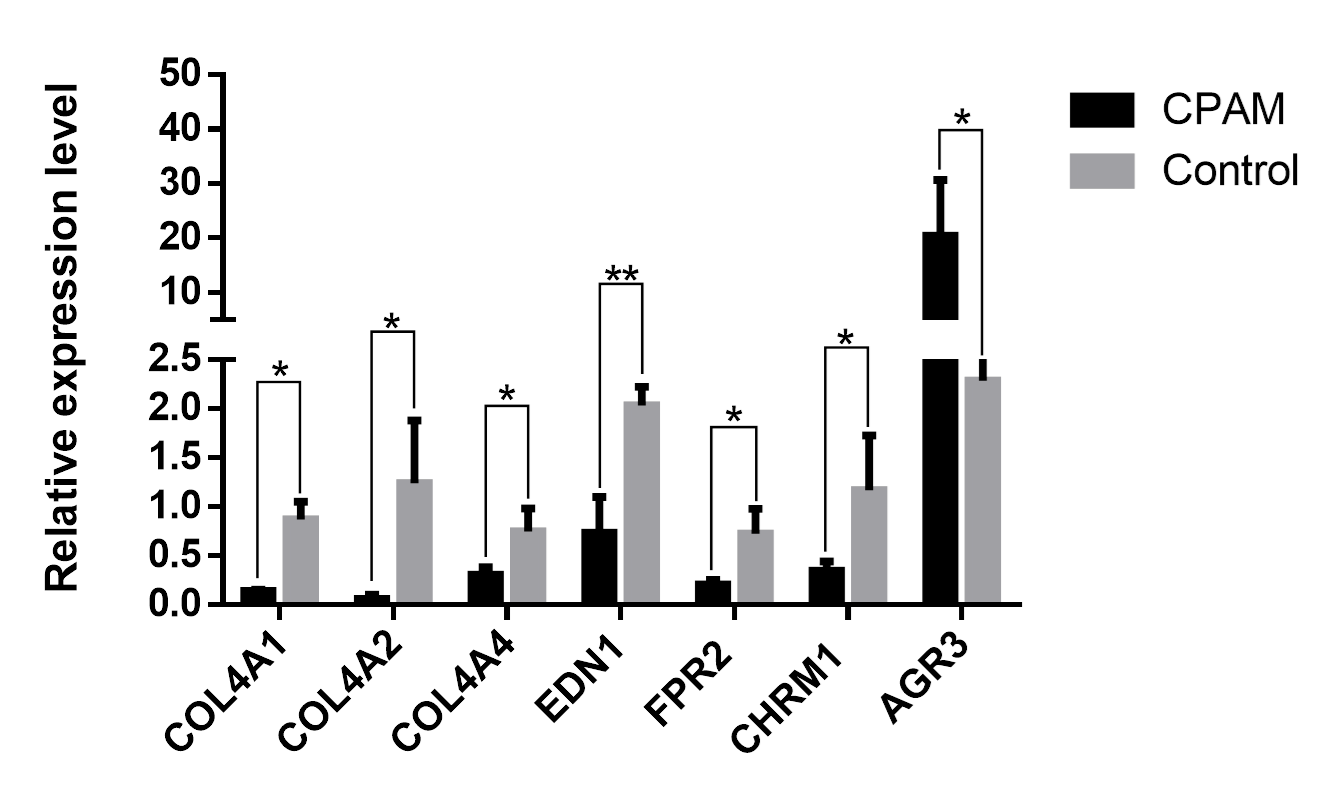

Supplement: Supplementary file 6 — Supplementary Material 6: Supplementary Fig. 6 qPCR validation of AGR3, COL4A1, COL4A2, COL4A4, EDN1, FPR2 and CHMR1 at mRNA level. [file 13578_2024_1276_MOESM6_ESM.png]
