# Supplementary material for: Integrative analysis of bulk and single-cell RNA sequencing reveals the gene expression profile and the critical signaling pathways of type II CPAM
Source: Cell Biosci. 2024 Jul 18;14:94. doi: 10.1186/s13578-024-01276-8 (PMC11264590; doi:10.1186/s13578-024-01276-8)
Supplement: Supplementary file 7 — Supplementary Material 7: Supplemental Table 1 Clinical characteristics of subjects used for RNA-seq and qPCR validation. [file 13578_2024_1276_MOESM7_ESM.docx]

| **Supplemental Table 7 Gene list for the top 10 modules performed by iWGCNA** | |
| --- | --- |
| **Module** | **Gene** |
| P2_I10_M24 | DCDC2B, WDR78, PIFO, AC005041.1, CFAP221, DNAH7, ROPN1L, PITX1, ANKRD66, AL121956.6, NPM2, RGS22, LRRC6, C9orf135, SPAG6, ARMC4, STOX1, MORN3, LRRC43, AL390755.2, ZFHX2, MNS1, HAGHL, HSD17B2, LCA5L, FAM83F |
| P1_I25_M1 | TTLL10-AS1, TTLL10, CFAP74, MORN1, AC242022.2, AC242022.1, TP73, HES2, ESPN, C1orf158, AL031283.1, FHAD1, UBXN10, GRHL3, CD164L2, IQCC, AL138787.1, TEKT2, DNALI1, FAM183A, CCDC30, CFAP57, BEST4, CCDC17, TSPAN1, CYP4X1, AGBL4, LDLRAD1, CYP2J2, C1orf87, AL136985.3, TCTEX1D1, ERICH3, LPAR3, WDR63, LINC02795, ODF2L, AC092802.2, C1orf194, KIAA1324, LINC01765, WDR3, SPAG17, AL589765.7, C1orf189, LRRC71, DCST2, DCST1, CFAP45, IGSF9, CFAP126, NECTIN4, CCDC181, MROH9, ANKRD45, APOBEC4, SLC45A3, AC096637.3, SPATA17, ACBD3-AS1, H2BU1, TRIM17, EFCAB2, GRHL1, C2orf50, DRC1, FAM166C, IFT172, TOGARAM2, SRD5A2, MORN2, ARHGEF33, DYNC2LI1, C2orf73, EML6, FAM161A, WDPCP, WDR92, ALMS1, HMGA1P8, DNAH6, AC097374.1, TEKT4, CNGA3, VWA3B, TSGA10, C2orf15, TBC1D8, CCDC138, NPHP1, RABL2A, STEAP3, INHBB, CCDC74B, CCDC74A, MAP3K19, HNMT, CCDC148, TTC21B, CCDC173, TTC30A, TTC30B, OSBPL6, CERKL, DNAJC10, DUSP19, ANKRD44-AS1, KIAA2012, MDH1B, SPAG16, CFAP65, TUBA4B, DAW1, FBXO36, NGEF, IQCA1, ANKMY1, CROCC2, AC104809.1, IL5RA, CNTN4-AS1, SRGAP3, SRGAP3-AS2, LINC01267, MRPS25, EFHB, NEK10, FBXL2, DLEC1, ULK4, HHATL, CCDC13, LZTFL1, KIF9, CDHR4, AC137630.1, ZMYND10, DNAH12, AC093928.1, C3orf67, SNTN, HHLA2, DZIP3, CFAP44, CCDC191, GPR156, MAATS1, WDR5B, FAM86JP, CFAP100, AC073439.1, KIAA1257, EFCAB12, NEK11, NME9, DZIP1L, PFN2, LINC00886, LEKR1, WDR49, ZBBX, LRRC34, TMEM212, CCDC39, SOX2, LINC02036, TCTEX1D2, IQCG, CFAP99, AL645924.1, CCDC96, AC006230.1, CC2D2A, C4orf19, WDR19, NSUN7, SPATA18, AC034139.1, PRDM8, CFAP299, CDS1, MAPK10, ADH6, CXXC4, PRSS12, INTU, CLGN, TTC29, TIGD4, SPATA4, C4orf47, FAM149A, TRIP13, AC122719.3, C5orf49, DNAH5, TTC23L, AC026801.2, SPEF2, CAPSL, C6, MCIDAS, AC026704.1, AC022107.1, ANKDD1B, MSH3, FAM81B, FAM174A, TMEM232, ZNF474, AC113349.1, LINC01843, NME5, SLC23A1, SPATA24, FABP6, AC008674.1, C6orf52, MAK, AL009177.1, STMND1, KIAA0319, ZNF204P, VWA7, SLC44A4, SCUBE3, KIF6, RSPH9, AL035701.1, SPATS1, TCTE1, EFHC1, AL391840.1, LCA5, CFAP206, KLHL32, GRIK2, ARMC2, PPIL6, AK9, FAM229B, RSPH4A, AL449363.2, MYB, ECT2L, ADGB, CCDC170, AL035530.2, C6orf118, PACRG, IQCE, RSPH10B, COL28A1, RSPH10B2, DNAH11, OSBPL3, C7orf57, PSPH, ZNF273, ELN-AS1, CCDC146, CFAP69, BAIAP2L1, DPY19L2P2, EFCAB10, CDHR3, IQUB, LRRC4, SMKR1, CEP41, LRGUK, TTC26, WDR86, WDR86-AS1, ATG9B, RNF32, NAT1, EFCAB1, RP1, TMEM68, CA8, PPP1R42, AC110998.1, C8orf34, IL7, TMEM67, AC087752.3, ERICH5, SPAG1, AZIN1-AS1, RHPN1, MAPK15, ARHGAP39, IQANK1, RFX3, GLIS3, KIF24, C9orf24, DNAI1, FAM166B, SPAG8, FAM201A, BX005214.1, BX005040.1, AL590491.2, AL353770.4, CDK20, AL161910.1, CCDC180, FSD1L, EPB41L4B, WDR31, WHRN, MORN5, AL445489.1, WDR38, CFAP157, TTC16, ODF2, CFAP77, AK8, SPACA9, PPP1R26-AS1, C9orf116, CCDC187, CR392000.2, SLC34A3, AL590226.2, MEIG1, ACBD7, ARMC3, ENKUR, FAM238C, AGAP12P, FRMPD2, LRRC18, CABCOCO1, CFAP70, DNAJC9-AS1, TMEM254, MAT1A, DYDC1, DYDC2, C10orf95, CFAP43, CFAP58, ENO4, CASC2, FANK1, CFAP46, LRRC56, CNGA4, ZNF214, STK33, C11orf16, AMPD3, E2F8, DCDC1, AGBL2, MS4A8, LRRC10B, SPTBN2, PITPNM1, AC004923.1, DNAJB13, AP003717.1, MAP6, FAM181B, ANKRD42, CCDC89, CCDC81, DEUP1, C11orf97, CNTN5, CFAP300, CEP126, AP001830.1, DYNC2H1, COLCA1, COLCA2, BTG4, C11orf88, PIH1D2, BCO2, TRIM29, USP2-AS1, CCDC153, JHY, SPA17, BARX2, IGSF9B, GLB1L2, AP004608.1, LRRC23, PZP, CASC1, ABCD2, KIF21A, KCNH3, CCDC65, AC125611.4, SLC4A8, KRT80, MDM1, CAPS2, LRRIQ1, CEP83, CFAP54, GOLGA2P5, APPL2, C12orf75, TTC41P, AC007695.1, TRPV4, IFT81, IQCD, CFAP73, HRK, CCDC60, HNF1A-AS1, NRAV, WDR66, HCAR1, DNAH10, AC127070.2, IFT88, MIPEP, HSPH1, CCNA1, STOML3, LINC00598, FAM216B, NRAD1, KCNRG, NEK5, AL353660.1, CFAP97D2, AL121790.2, FOXA1, TTC6, GPR135, SIX1, SIX4, SYT16, LINC00643, DNAL1, BBOF1, SAMD15, TTC8, EFCAB11, AL357093.1, AL121839.2, GON7, FAM181A-AS1, AK7, MOK, LINC02345, ELL3, SLC27A2, DNAAF4, TEX9, FAM81A, AC104590.1, IQCH, CALML4, AC027237.5, DRAIC, NRG4, DNAJA4, ACSBG1, CFAP161, SAXO2, GOLGA2P10, CPEB1, CPEB1-AS1, AC044860.1, WDR93, AC079075.1, C1QTNF8, WDR90, CCDC78, NHLRC4, HS3ST6, BAIAP3, JPT2, C16orf71, DNAH3, VWA3A, KIAA0556, AC009093.8, AC025279.1, CCDC189, LINC01571, AC007906.2, RPGRIP1L, DRC7, CCDC113, AC099521.4, HYDIN, ZNF19, TMEM231, TMEM231P1, DYNLRB2, AC108097.1, AC092718.3, DNAAF1, FAM92B, C17orf97, SPATA33, SPIRE2, GAS8, AC010538.1, LINC02166, MIR132, RTN4RL1, AC087500.1, DNAH2, CYB5D1, TEKT1, CFAP52, DNAH9, AC005703.7, TEKT3, DRC3, FBXW10, GAS2L2, SRCIN1, TTC25, CCDC103, HOXB3, LRRC46, TTLL6, MYCBPAP, EPN3, ANKFN1, MSI2, AC005962.2, TSPOAP1, AC015813.8, PPM1E, AC005821.1, MARCHF10, PRR29-AS1, PRR29, LINC00511, DNAI2, AC103809.1, KIF19, RNF157-AS1, FOXJ1, AC015802.7, CCDC40, KCTD1, AQP4-AS1, ZNF396, FHOD3, CFAP53, FBXO15, PARD6G-AS1, PLPP2, GRIN3B, CATSPERD, CAPS, ZNF440, CCDC151, RHPN2, AC016582.3, CYP2A13, PHLDB3, GIPR, AC074212.1, CCDC114, ZNF473, AC010624.3, AC010624.1, CACNG6, DNAAF3, TMEM190, AL121899.1, AL121899.2, SPEF1, DZANK1, CFAP61, TTLL9, RALY-AS1, ARHGAP40, PTPRT, AL117382.2, WFDC6, HSPD1P21, C20orf85, FNDC11, SMIM34B, KCNE1B, SMIM34A, CLIC6, GET1, B3GALT5, RIPK4, RSPH1, C21orf58, TUBA3FP, LRRC74B, RSPH14, RAB36, C22orf15, AC004832.1, CCDC157, C22orf23, FAM227A, EFCAB6, RIBC2, RABL2B, MAP7D2, CFAP47, PCSK1N, RIBC1, USP51, TSPAN6, NUP62CL, PIH1D3, KLHL13, AKAP14, CCDC160, CETN2, UCN3 |
| P3_I8_M9 | AL591686.2, CHN1, GLS, CFLAR, ACADL, ALS2CL, SEMA3F-AS1, ARHGEF26, ETV5, CTBP1-AS, TRPC3, WWC2-AS2, CAMK4, SYCP2L, GABBR1, NOTCH4, VEGFA, AL391807.1, AC004540.1, GSAP, PON2, OR2A9P, DPYSL2, COL22A1, AGTPBP1, NEBL, MYRF, RASGRP2, CABP4, KLRK1, ADGRD1, USP12, LINC02284, ATXN3, CCDC9B, TMEM266, AC108134.3, LINC01996, ANKRD29, ADAMTS10, MORC4, GAB3 |
| P5_I8_M9 | LURAP1, CCDC190, WDR35, ANKRD36BP2, CLDN1, KIAA0825, MUC21, ARMC2-AS1, SDK1, AC138356.2, CRISPLD1, LINC02605, FRMPD2B, ALDH3B2, NOXRED1, SHC4, CYP11A1, CCDC33, AC009093.10, MEIS3P2, CDH26, MID1, LINC01908 |
| P5_I8_M19 | ADAMTSL4, CD5L, NLRC4, STAC, ADTRP, SNX10, LHFPL3-AS2, MSR1, CA2, AC090559.1, LPXN, MS4A7, PLBD1, PTPRO, GPD1, NCKAP1L, CYSLTR2, SLC7A7, SPN, BTK |
| P5_I8_M3 | FGR, MOB3C, TPM3, STAT4, SLC11A1, PFKFB4, NUDT16, SH3TC1, GLRX, HSD17B4, LCP2, HK3, TMEM170B, PPP1R18, CCDC71L, ZC3HAV1, RIPK2, PIP4K2A, PTPRE, MYBPC3, USP15, TINF2, GPR68, CHP1, LACTB, PLEKHO2, TM6SF1, AC026462.1, MRC2, MKNK2, B3GNT8, VASP, ABCG1 |
| P5_I8_M1 | AL513477.1, ARHGEF19, ANO7L1, STPG1, CCDC24, PRRT3, AC099050.1, AC079848.2, AC055714.1, FAM47E, LINC01091, RPL30P7, AC112204.3, AC010255.1, SLC22A4, REEP2, C6orf201, LRRC73, ADGRF4, CSMD1, PPP1R16A, NCS1, CDNF, AP001085.1, GPR162, RHEBL1, AC073655.2, HNF1A, LRRC63, PPP1R36, FSIP1, SMIM22, SDR42E2, TSNAXIP1, AC004943.2, USP43, CDH2, LINC00683, LINC01927, TJP3, TNFAIP8L1, PSENEN, PNMA8A, DUSP18, WNT7B, NXF2, AL390879.1, AC010273.3 |
| P4_I7_M2 | KCNAB2, THEMIS2, FMO5, RFX5, SLAMF8, CD48, LAX1, FBXO41, PDCD1, TREX1, RAP2B, EMB, HAVCR2, ITK, DEF6, CRYBG1, SCML4, PIK3CG, SEMA4D, C9orf139, MS4A6A, CD6, CCDC88B, RELT, IL10RA, CD3E, CLEC2D, CLEC7A, DGKA, LINC02384, TNFSF13B, PLCB2, CIITA, ITGAX, CARMIL2, PLCG2, FANCA, ZMYND15, ACAP1, PIK3R5, AC008105.3, MILR1, FCHO1, ZBP1, UBASH3A, ITGB2-AS1, PARVG, SEPTIN6 |
| P1_I25_M7 | AL590822.3, UBXN11, ADPRS, OSCP1, MYCBP, RIIAD1, PPOX, UFC1, NME7, MAPRE3, CFAP36, C2orf81, WDR54, ECRG4, BBS5, CATIP, GLB1L, TTC21A, RUVBL1, SERPINI2, LINC01513, GMNN, UBE3D, NT5DC1, AGR3, IFT22, LRWD1, IFT74, AL391987.2, BRD3OS, DLG5-AS1, DPCD, LRRC27, KNDC1, IFTAP, MAPK8IP1, C11orf49, ZNHIT2, FAM86C2P, IFT46, USP2, FAM86FP, GIHCG, TCTN1, NUBPL, ZC2HC1C, SPATA7, DICER1-AS1, CLBA1, CLDN9, AC130456.2, IQCK, ENKD1, ZDHHC1, TPPP3, TMEM107, B9D1, TLCD1, CACNA1G, SMIM5, PCSK4, RFX2, CTXN1, CERS4, AC010422.2, FBXW9, DMKN, B9D2, RUVBL2, SLC6A16, FUZ, Z95115.1, IFT27, CBY1, MCAT, DENND6B, GYG2, FAM104B, AC099521.2 |
| P1_I25_M11 | CTNNBIP1, C1orf216, ZFYVE9, PSRC1, PLEKHO1, GUK1, WNT7A, MYL3, MGLL, GATA2-AS1, TMEM44, SHROOM1, GRPEL2, BTN2A1, KCNK17, PDGFA, RADIL, TECPR1, ARHGEF35-AS1, DENND3, RGP1, PKN3, NPDC1, RBM17, HACD1, PALD1, TAF10, SLC22A10, C1QTNF5, LTBR, IFFO1, HDAC7, RILPL1, DCUN1D2, ANKRD9, DAPK2, SNX22, RNF112, STARD3, PNMT, ITGA3, MPPE1, GATA6-AS1, ARHGAP45, DAPK3, TRIP10, KANK3, ARRDC2, RRAS, FKBP1A, RIN2, ELMO2, TMEM189, CLDN5, EMID1 |
