# Supplementary material for: Integrative analysis of bulk and single-cell RNA sequencing reveals the gene expression profile and the critical signaling pathways of type II CPAM
Source: Cell Biosci. 2024 Jul 18;14:94. doi: 10.1186/s13578-024-01276-8 (PMC11264590; doi:10.1186/s13578-024-01276-8)
Supplement: Supplementary file 8 — Supplementary Material 8: Supplemental Table 2 Overlapped genes between DEGs related to CPAM and DEGs related to sex. [file 13578_2024_1276_MOESM8_ESM.docx]

**Supplemental Table 8 Overlapped genes between epithelial cell marker genes and DEGs**

| **Gene** | **Gene Full Name** | **CPAM (z score)** | **Contrl (z score)** | **log2 fold change (CPAM/control)** | **Overlapped group** |
| --- | --- | --- | --- | --- | --- |
| AGER | ADVANCED GLYCOSYLATION END PRODUCT-SPECIFIC RECEPTOR | 0.412315003 | 0.47218512 | -1.334282891 | DEG |
| AGR3 | ANTERIOR GRADIENT 3,PROTEIN DISULPHIDE ISOMERASE FAMILY MEMBER | 0.486894329 | 0.393415725 | 1.932630821 | DEG, P1_I25_M7 |
| ALOX5AP | ARACHIDONATE 5-LIPOXYGENASE-ACTIVATING PROTEIN | 0.03609998 | 0.045368222 | -1.133044394 | DEG |
| CADM1 | CELL ADHESION MOLECULE 1 | 0.322121766 | 0.342322797 | -1.010621213 | DEG |
| CCL4 | CHEMOKINE, CC MOTIF, LIGAND 4 | -0.085203564 | -0.083729268 | -1.551960532 | DEG |
| CD52 | CAMPATH-1 ANTIGEN | 0.052550678 | 0.209035116 | -1.016883922 | DEG |
| CEMIP2 | CELL MIGRATION-INDUCING HYALURONIDASE 2 | 0.248776867 | 0.275775936 | -1.288146997 | DEG |
| CLDN18 | CLAUDIN 18 | 0.680349569 | 0.750090706 | -1.624717985 | DEG |
| CLIC3 | CHLORIDE INTRACELLULAR CHANNEL 3 | 0.018030964 | 0.022867895 | -1.071436749 | DEG |
| COL4A2 | COLLAGEN, TYPE IV, ALPHA-2 | 4.092393759 | 4.506076058 | -1.105240485 | DEG |
| CST7 | CYSTATIN 7 | -0.065414716 | -0.063094673 | -1.043678669 | DEG |
| DUSP6 | DUAL-SPECIFICITY PHOSPHATASE 6 | 0.203983796 | 0.226550577 | -1.014570666 | DEG |
| EMP2 | EPITHELIAL MEMBRANE PROTEIN 2 | 2.150109888 | 2.269716681 | -1.061455587 | DEG |
| HBEGF | HEPARIN-BINDING EGF-LIKE GROWTH FACTOR | 0.045158589 | 0.058421261 | -1.125170285 | DEG |
| ICAM1 | INTERCELLULAR ADHESION MOLECULE 1 | 0.929326416 | 0.981641702 | -1.067882287 | DEG |
| IL1B | INTERLEUKIN 1-BETA | -0.082552236 | -0.081233016 | -1.706144613 | DEG |
| IL32 | INTERLEUKIN 32 | 0.440739139 | 0.465414538 | -1.133081781 | DEG |
| LAMA3 | LAMININ, ALPHA-3 | 0.401337397 | 0.443211473 | -1.334725191 | DEG |
| MYO1G | MYOSIN IG | 0.097565726 | 0.112141202 | -1.074456873 | DEG |
| NKG7 | NATURAL KILLER CELL GROUP 7 SEQUENCE | -0.038807525 | -0.033037134 | -1.139331183 | DEG |
| NR4A2 | NUCLEAR RECEPTOR SUBFAMILY 4, GROUP A, MEMBER 2 | 0.181738301 | 0.190303899 | 1.531471046 | DEG |
| ODF3B | OUTER DENSE FIBER OF SPERM TAILS 3B | 1.264573575 | 1.068214773 | 1.076119405 | DEG |
| RTKN2 | RHOTEKIN 2 | 0.136770659 | 0.151894514 | -1.636294219 | DEG |
| S100A8 | S100 CALCIUM-BINDING PROTEIN A8 | -0.078353766 | -0.076980168 | -2.151888814 | DEG |
| SCEL | SCIELLIN | 0.071250156 | 0.091023401 | -1.449649045 | DEG |
| SCGB1A1 | SECRETOGLOBIN, FAMILY 1A, MEMBER 1 | 64.36513905 | 65.24545377 | 3.396478257 | DEG |
| SCGB3A1 | SECRETOGLOBIN, FAMILY 3A, MEMBER 1 | 3.867161579 | 4.292353062 | 3.629315401 | DEG |
| SCGB3A2 | SECRETOGLOBIN, FAMILY 3A, MEMBER 2 | 6.117395879 | 4.808583697 | 2.214945734 | DEG |
| SERTAD1 | SERTA DOMAIN DONTAINING 1 | -0.044273341 | -0.041140276 | -1.079126342 | DEG |
| SFTPC | SURFACTANT, PULMONARY-ASSOCIATED PROTEIN C | 51.49234708 | 56.20079266 | -1.102276893 | DEG |
| SLC11A1 | SOLUTE CARRIER FAMILY 11 (PROTON-COUPLED DIVALENT METAL ION TRANSPORTER), MEMBER 1 | 0.32433682 | 0.369543906 | -1.238618666 | DEG, P5_I8_M3 |
| SLC2A3 | SOLUTE CARRIER FAMILY 2 (FACILITATED GLUCOSE TRANSPORTER), MEMBER 3 | 0.167345578 | 0.194486811 | -1.341522764 | DEG |
| SLC39A8 | SOLUTE CARRIER FAMILY 39 (ZINC TRANSPORTER), MEMBER 8 | 0.374085153 | 0.41061048 | -1.07113955 | DEG |
| SPOCK2 | SPARC/OSTEONECTIN, CWCV, AND KAZAL-LIKE DOMAINS PROTEOGLYCAN 2 | 0.924015266 | 1.043531562 | -1.346552418 | DEG |
| STX11 | SYNTAXIN 11 | -0.015730618 | -0.009147301 | -1.157972163 | DEG |
| SYTL3 | SYNAPTOTAGMIN LIKE 3 | 0.152164187 | 0.124452071 | 1.44079421 | DEG |
| TNNC1 | TROPONIN C, SLOW SKELETAL AND CARDIAC TYPE | 0.102132195 | 0.119146703 | -1.291837834 | DEG |
| ZNF331 | ZINC FINGER PROTEIN 331 | 0.105260504 | 0.117649596 | -1.416767681 | DEG |
