# Supplementary material for: Integrative analysis of bulk and single-cell RNA sequencing reveals the gene expression profile and the critical signaling pathways of type II CPAM
Source: Cell Biosci. 2024 Jul 18;14:94. doi: 10.1186/s13578-024-01276-8 (PMC11264590; doi:10.1186/s13578-024-01276-8)
Supplement: Supplementary file 9 — Supplementary Material 9: Supplemental Table 3 DEGs between CPAM cases and controls. [file 13578_2024_1276_MOESM9_ESM.docx]

**Supplemental Table 9 Overlapped genes between epithelial cell marker genes and the top10 module genes**

| **Gene** | **Gene Full Name** | **CPAM (z score)** | **Contrl (z score)** | **log2 fold change (CPAM/control)** | **Overlapped group** |
| --- | --- | --- | --- | --- | --- |
| AGR3 | anterior gradient 3, protein disulphide isomerase family member | 0.486894329 | 0.064170365 | 1.932630821 | P1_I25_M7,DEG |
| CD48 | CD48 molecule | 0.033687745 | 0.039552514 | -0.190366046 | P4_I7_M2 |
| CFLAR | CASP8 and FADD like apoptosis regulator | 2.035659695 | 2.99324032 | -0.490353429 | P3_I8_M9 |
| CHP1 | calcineurin like EF-hand protein 1 | 0.104326163 | 0.182509427 | -0.498804259 | P5_I8_M3 |
| DPYSL2 | dihydropyrimidinase like 2 | 0.629112647 | 1.313315793 | -0.875887675 | P3_I8_M9 |
| GLS | glutaminase | 0.312063472 | 0.497143086 | -0.521849243 | P3_I8_M9 |
| ITGA3 | integrin subunit alpha 3 | 1.709487684 | 2.722246165 | -0.555584138 | P1_I25_M11 |
| MGLL | monoglyceride lipase | 0.546157234 | 0.892294819 | -0.575589447 | P1_I25_M11 |
| MKNK2 | MAPK interacting serine/threonine kinase 2 | 0.899063538 | 1.295488027 | -0.449918986 | P5_I8_M3 |
| RBM17 | RNA binding motif protein 17 | 0.766412984 | 1.002735836 | -0.298959497 | P1_I25_M11 |
| RRAS | RAS related | 0.339293316 | 0.730567334 | -0.859120197 | P1_I25_M11 |
| SLC11A1 | solute carrier family 11 member 1 | 0.32433682 | 0.853620621 | -1.238618666 | P5_I8_M3,DEG |
| TAF10 | TATA-box binding protein associated factor 10 | 0.3905392 | 0.568713989 | -0.388662016 | P1_I25_M11 |
| USP15 | ubiquitin specific peptidase 15 | 0.109271083 | 0.171912556 | -0.356201967 | P5_I8_M3 |
| VASP | vasodilator stimulated phosphoprotein | 0.348172009 | 0.629712702 | -0.677391665 | P5_I8_M3 |
| VEGFA | vascular endothelial growth factor A | 2.407685815 | 4.20968322 | -0.661324542 | P3_I8_M9 |
