# Supplementary material for: Integrative analysis of bulk and single-cell RNA sequencing reveals the gene expression profile and the critical signaling pathways of type II CPAM
Source: Cell Biosci. 2024 Jul 18;14:94. doi: 10.1186/s13578-024-01276-8 (PMC11264590; doi:10.1186/s13578-024-01276-8)
Supplement: Supplementary file 10 — Supplementary Material 10: Supplemental Table 4 GO enrichment analysis (biological process, BP) of up-regulated and down-regulated genes. [file 13578_2024_1276_MOESM10_ESM.docx]

**Supplemental Table 10 Marker genes of epithelial subtypes**

| **Cell type** | **Marker genes** |
| --- | --- |
| Alveolar Type 1 Cells | *AGER, PDPN, CLIC5* |
| Alveolar Type 2 Cells | *SFTPB, SFTPC, SFTPD, MUC1, ETV5* |
| Club Cells | *CYP2F2, SCGB3A2, CCKAR* |
| Ciliated Cells | *FOXJ1, TUBB1, TP73, CCDC78* |
