# Supplementary material for: Integrative analysis of bulk and single-cell RNA sequencing reveals the gene expression profile and the critical signaling pathways of type II CPAM
Source: Cell Biosci. 2024 Jul 18;14:94. doi: 10.1186/s13578-024-01276-8 (PMC11264590; doi:10.1186/s13578-024-01276-8)
Supplement: Supplementary file 11 — Supplementary Material 11: Supplemental Table 5 GO enrichment analysis (molecular function, MF) and (cell component, CC) of up-regulated and down-regulated genes. [file 13578_2024_1276_MOESM11_ESM.docx]

**Supplemental Table 11 Inference and analysis of CPAM-associated epithelial cells communication from single-cell data using CellChat**

| **Source** | **Target** | **Ligand** | **Receptor** | **Prob** | **P-val** | **Interaction name** | **Pathway name** | **Annotation** | **Evidence** |
| --- | --- | --- | --- | --- | --- | --- | --- | --- | --- |
| Cil | AT1 | GDF15 | TGFBR2 | 0.014699 | 0 | GDF15_TGFBR2 | GDF | Secreted Signaling | KEGG: hsa04350 |
| Club | AT1 | GDF15 | TGFBR2 | 0.013962 | 0 | GDF15_TGFBR2 | GDF | Secreted Signaling | KEGG: hsa04350 |
| Cil | AT2 | GDF15 | TGFBR2 | 0.017627 | 0 | GDF15_TGFBR2 | GDF | Secreted Signaling | KEGG: hsa04350 |
| Club | AT2 | GDF15 | TGFBR2 | 0.016746 | 0 | GDF15_TGFBR2 | GDF | Secreted Signaling | KEGG: hsa04350 |
| Cil | Club | GDF15 | TGFBR2 | 0.003705 | 0.03 | GDF15_TGFBR2 | GDF | Secreted Signaling | KEGG: hsa04350 |
| Club | AT2 | WNT5A | FZD5 | 0.000338 | 0 | WNT5A_FZD5 | ncWNT | Secreted Signaling | KEGG: hsa04310 |
| Club | Cil | WNT5A | FZD6 | 0.000245 | 0 | WNT5A_FZD6 | ncWNT | Secreted Signaling | KEGG: hsa04310 |
| AT2 | AT1 | AREG | EGFR | 0.010068 | 0 | AREG_EGFR | EGF | Secreted Signaling | KEGG: hsa04012 |
| Cil | AT1 | AREG | EGFR | 0.007941 | 0 | AREG_EGFR | EGF | Secreted Signaling | KEGG: hsa04012 |
| Club | AT1 | AREG | EGFR | 0.002538 | 0 | AREG_EGFR | EGF | Secreted Signaling | KEGG: hsa04012 |
| AT2 | AT2 | AREG | EGFR | 0.013764 | 0 | AREG_EGFR | EGF | Secreted Signaling | KEGG: hsa04012 |
| Cil | AT2 | AREG | EGFR | 0.010865 | 0 | AREG_EGFR | EGF | Secreted Signaling | KEGG: hsa04012 |
| Club | AT2 | AREG | EGFR | 0.00348 | 0 | AREG_EGFR | EGF | Secreted Signaling | KEGG: hsa04012 |
| AT2 | AT1 | AREG | EGFR_ERBB2 | 0.009857 | 0 | AREG_EGFR_ERBB2 | EGF | Secreted Signaling | KEGG: hsa04012 |
| Cil | AT1 | AREG | EGFR_ERBB2 | 0.007775 | 0 | AREG_EGFR_ERBB2 | EGF | Secreted Signaling | KEGG: hsa04012 |
| Club | AT1 | AREG | EGFR_ERBB2 | 0.002485 | 0 | AREG_EGFR_ERBB2 | EGF | Secreted Signaling | KEGG: hsa04012 |
| Cil | AT1 | BTC | EGFR | 0.00109 | 0 | BTC_EGFR | EGF | Secreted Signaling | KEGG: hsa04012 |
| Cil | AT2 | BTC | EGFR | 0.001495 | 0 | BTC_EGFR | EGF | Secreted Signaling | KEGG: hsa04012 |
| Cil | AT1 | BTC | EGFR_ERBB2 | 0.001067 | 0 | BTC_EGFR_ERBB2 | EGF | Secreted Signaling | KEGG: hsa04012 |
| AT1 | AT1 | HBEGF | EGFR | 0.004299 | 0 | HBEGF_EGFR | EGF | Secreted Signaling | KEGG: hsa04012 |
| AT1 | AT2 | HBEGF | EGFR | 0.005889 | 0 | HBEGF_EGFR | EGF | Secreted Signaling | KEGG: hsa04012 |
| AT1 | AT1 | HBEGF | EGFR_ERBB2 | 0.004208 | 0 | HBEGF_EGFR_ERBB2 | EGF | Secreted Signaling | KEGG: hsa04012 |
| AT1 | AT2 | MIF | CD74_CD44 | 0.025892 | 0 | MIF_CD74_CD44 | MIF | Secreted Signaling | PMID: 29637711; PMID: 26175090 |
| AT2 | AT2 | MIF | CD74_CD44 | 0.072103 | 0 | MIF_CD74_CD44 | MIF | Secreted Signaling | PMID: 29637711; PMID: 26175090 |
| Cil | AT2 | MIF | CD74_CD44 | 0.105755 | 0 | MIF_CD74_CD44 | MIF | Secreted Signaling | PMID: 29637711; PMID: 26175090 |
| Club | AT2 | MIF | CD74_CD44 | 0.064022 | 0 | MIF_CD74_CD44 | MIF | Secreted Signaling | PMID: 29637711; PMID: 26175090 |
| AT1 | Club | MIF | CD74_CD44 | 0.011136 | 0 | MIF_CD74_CD44 | MIF | Secreted Signaling | PMID: 29637711; PMID: 26175090 |
| AT2 | Club | MIF | CD74_CD44 | 0.031873 | 0 | MIF_CD74_CD44 | MIF | Secreted Signaling | PMID: 29637711; PMID: 26175090 |
| Cil | Club | MIF | CD74_CD44 | 0.047714 | 0 | MIF_CD74_CD44 | MIF | Secreted Signaling | PMID: 29637711; PMID: 26175090 |
| Club | Club | MIF | CD74_CD44 | 0.028164 | 0 | MIF_CD74_CD44 | MIF | Secreted Signaling | PMID: 29637711; PMID: 26175090 |
| AT2 | AT2 | LIF | LIFR_IL6ST | 0.002021 | 0 | LIF_LIFR_IL6ST | LIFR | Secreted Signaling | KEGG: hsa04060 |
| AT2 | AT2 | CSF3 | CSF3R | 0.073762 | 0 | CSF3_CSF3R | CSF3 | Secreted Signaling | KEGG: hsa04060 |
| Club | AT2 | CSF3 | CSF3R | 0.023425 | 0 | CSF3_CSF3R | CSF3 | Secreted Signaling | KEGG: hsa04060 |
| AT2 | AT2 | TNFSF14 | LTBR | 0.000725 | 0 | TNFSF14_LTBR | LIGHT | Secreted Signaling | KEGG: hsa04060 |
| AT2 | Cil | TNFSF14 | LTBR | 0.001063 | 0 | TNFSF14_LTBR | LIGHT | Secreted Signaling | KEGG: hsa04060 |
| AT2 | Club | TNFSF14 | LTBR | 0.000809 | 0 | TNFSF14_LTBR | LIGHT | Secreted Signaling | KEGG: hsa04060 |
| AT2 | Cil | TNFSF14 | TNFRSF14 | 0.000618 | 0 | TNFSF14_TNFRSF14 | LIGHT | Secreted Signaling | KEGG: hsa04060 |
| AT2 | Club | TNFSF14 | TNFRSF14 | 0.000466 | 0 | TNFSF14_TNFRSF14 | LIGHT | Secreted Signaling | KEGG: hsa04060 |
| AT2 | AT2 | NAMPT | INSR | 0.009017 | 0.02 | NAMPT_INSR | VISFATIN | Secreted Signaling | PMID: 28490838 |
| AT2 | Cil | NAMPT | INSR | 0.012683 | 0 | NAMPT_INSR | VISFATIN | Secreted Signaling | PMID: 28490838 |
| Club | Cil | NAMPT | INSR | 0.010697 | 0 | NAMPT_INSR | VISFATIN | Secreted Signaling | PMID: 28490838 |
| AT2 | Club | NAMPT | INSR | 0.030527 | 0 | NAMPT_INSR | VISFATIN | Secreted Signaling | PMID: 28490838 |
| Cil | Club | NAMPT | INSR | 0.015957 | 0 | NAMPT_INSR | VISFATIN | Secreted Signaling | PMID: 28490838 |
| Club | Club | NAMPT | INSR | 0.025821 | 0 | NAMPT_INSR | VISFATIN | Secreted Signaling | PMID: 28490838 |
| Cil | AT1 | MDK | SDC1 | 0.002562 | 0 | MDK_SDC1 | MK | Secreted Signaling | PMID: 28356350 |
| Club | AT1 | MDK | SDC1 | 0.007857 | 0 | MDK_SDC1 | MK | Secreted Signaling | PMID: 28356350 |
| Cil | AT2 | MDK | SDC1 | 0.003018 | 0 | MDK_SDC1 | MK | Secreted Signaling | PMID: 28356350 |
| Club | AT2 | MDK | SDC1 | 0.009249 | 0 | MDK_SDC1 | MK | Secreted Signaling | PMID: 28356350 |
| Cil | Club | MDK | SDC1 | 0.002254 | 0 | MDK_SDC1 | MK | Secreted Signaling | PMID: 28356350 |
| Club | Club | MDK | SDC1 | 0.006916 | 0 | MDK_SDC1 | MK | Secreted Signaling | PMID: 28356350 |
| Cil | AT1 | MDK | SDC4 | 0.011939 | 0 | MDK_SDC4 | MK | Secreted Signaling | PMID: 28356350 |
| Club | AT1 | MDK | SDC4 | 0.035919 | 0 | MDK_SDC4 | MK | Secreted Signaling | PMID: 28356350 |
| Cil | AT2 | MDK | SDC4 | 0.015021 | 0 | MDK_SDC4 | MK | Secreted Signaling | PMID: 28356350 |
| Club | AT2 | MDK | SDC4 | 0.044907 | 0 | MDK_SDC4 | MK | Secreted Signaling | PMID: 28356350 |
| Cil | Cil | MDK | SDC4 | 0.00901 | 0 | MDK_SDC4 | MK | Secreted Signaling | PMID: 28356350 |
| Club | Cil | MDK | SDC4 | 0.027269 | 0 | MDK_SDC4 | MK | Secreted Signaling | PMID: 28356350 |
| Cil | Club | MDK | SDC4 | 0.011723 | 0 | MDK_SDC4 | MK | Secreted Signaling | PMID: 28356350 |
| Club | Club | MDK | SDC4 | 0.035283 | 0 | MDK_SDC4 | MK | Secreted Signaling | PMID: 28356350 |
| Cil | AT2 | MDK | ITGA6_ITGB1 | 0.001949 | 0 | MDK_ITGA6_ITGB1 | MK | Secreted Signaling | PMID: 28356350 |
| Club | AT2 | MDK | ITGA6_ITGB1 | 0.005986 | 0 | MDK_ITGA6_ITGB1 | MK | Secreted Signaling | PMID: 28356350 |
| Cil | AT2 | MDK | NCL | 0.005908 | 0 | MDK_NCL | MK | Secreted Signaling | PMID: 28356350 |
| Club | AT2 | MDK | NCL | 0.017995 | 0 | MDK_NCL | MK | Secreted Signaling | PMID: 28356350 |
| Cil | Cil | MDK | NCL | 0.003251 | 0 | MDK_NCL | MK | Secreted Signaling | PMID: 28356350 |
| Club | Cil | MDK | NCL | 0.009957 | 0 | MDK_NCL | MK | Secreted Signaling | PMID: 28356350 |
| Cil | Club | MDK | NCL | 0.006509 | 0 | MDK_NCL | MK | Secreted Signaling | PMID: 28356350 |
| Club | Club | MDK | NCL | 0.0198 | 0 | MDK_NCL | MK | Secreted Signaling | PMID: 28356350 |
| AT2 | Cil | C5 | C5AR1 | 0.000913 | 0 | HC_C5AR1 | COMPLEMENT | Secreted Signaling | KEGG: hsa04080 |
| AT1 | AT2 | EDN1 | EDNRB | 0.002552 | 0 | EDN1_EDNRB | EDN | Secreted Signaling | KEGG: hsa04080 |
| AT1 | AT2 | SEMA3B | NRP2_PLXNA2 | 0.005499 | 0 | SEMA3B_NRP2_PLXNA2 | SEMA3 | Secreted Signaling | PMID: 27533782 |
| Club | AT2 | SEMA3B | NRP2_PLXNA2 | 0.000663 | 0 | SEMA3B_NRP2_PLXNA2 | SEMA3 | Secreted Signaling | PMID: 27533782 |
| AT1 | Cil | SEMA3B | NRP2_PLXNA2 | 0.005479 | 0 | SEMA3B_NRP2_PLXNA2 | SEMA3 | Secreted Signaling | PMID: 27533782 |
| Club | Cil | SEMA3B | NRP2_PLXNA2 | 0.000661 | 0 | SEMA3B_NRP2_PLXNA2 | SEMA3 | Secreted Signaling | PMID: 27533782 |
| Cil | AT2 | SEMA3C | NRP2_PLXNA2 | 0.000979 | 0 | SEMA3C_NRP2_PLXNA2 | SEMA3 | Secreted Signaling | PMID: 27533782 |
| Cil | Cil | SEMA3C | NRP2_PLXNA2 | 0.000975 | 0 | SEMA3C_NRP2_PLXNA2 | SEMA3 | Secreted Signaling | PMID: 27533782 |
| AT1 | AT2 | GRN | SORT1 | 0.002116 | 0 | GRN_SORT1 | GRN | Secreted Signaling | PMID: 29555433 |
| AT2 | AT2 | GRN | SORT1 | 0.003135 | 0 | GRN_SORT1 | GRN | Secreted Signaling | PMID: 29555433 |
| Club | AT2 | GRN | SORT1 | 0.003254 | 0 | GRN_SORT1 | GRN | Secreted Signaling | PMID: 29555433 |
| Club | AT2 | LGALS9 | CD44 | 0.004528 | 0 | LGALS9_CD44 | GALECTIN | Secreted Signaling | PMID: 25065622 |
| Club | Club | LGALS9 | CD44 | 0.001096 | 0 | LGALS9_CD44 | GALECTIN | Secreted Signaling | PMID: 25065622 |
| AT2 | AT1 | FN1 | ITGA3_ITGB1 | 0.005796 | 0 | FN1_ITGA3_ITGB1 | FN1 | ECM-Receptor | KEGG: hsa04512 |
| AT2 | AT2 | FN1 | ITGA3_ITGB1 | 0.001607 | 0.01 | FN1_ITGA3_ITGB1 | FN1 | ECM-Receptor | KEGG: hsa04512 |
| AT2 | Cil | FN1 | ITGA3_ITGB1 | 0.003944 | 0 | FN1_ITGA3_ITGB1 | FN1 | ECM-Receptor | KEGG: hsa04512 |
| AT2 | Club | FN1 | ITGA3_ITGB1 | 0.001637 | 0.01 | FN1_ITGA3_ITGB1 | FN1 | ECM-Receptor | KEGG: hsa04512 |
| AT1 | AT1 | LAMA3 | ITGA3_ITGB1 | 0.037838 | 0 | LAMA3_ITGA3_ITGB1 | LAMININ | ECM-Receptor | KEGG: hsa04512 |
| AT1 | AT2 | LAMA3 | ITGA3_ITGB1 | 0.01074 | 0.01 | LAMA3_ITGA3_ITGB1 | LAMININ | ECM-Receptor | KEGG: hsa04512 |
| AT1 | Cil | LAMA3 | ITGA3_ITGB1 | 0.026017 | 0 | LAMA3_ITGA3_ITGB1 | LAMININ | ECM-Receptor | KEGG: hsa04512 |
| AT1 | Club | LAMA3 | ITGA3_ITGB1 | 0.01094 | 0 | LAMA3_ITGA3_ITGB1 | LAMININ | ECM-Receptor | KEGG: hsa04512 |
| AT1 | AT1 | LAMA5 | ITGA3_ITGB1 | 0.01233 | 0 | LAMA5_ITGA3_ITGB1 | LAMININ | ECM-Receptor | KEGG: hsa04512 |
| Cil | AT1 | LAMA5 | ITGA3_ITGB1 | 0.007908 | 0 | LAMA5_ITGA3_ITGB1 | LAMININ | ECM-Receptor | KEGG: hsa04512 |
| Club | AT1 | LAMA5 | ITGA3_ITGB1 | 0.007502 | 0 | LAMA5_ITGA3_ITGB1 | LAMININ | ECM-Receptor | KEGG: hsa04512 |
| AT1 | Cil | LAMA5 | ITGA3_ITGB1 | 0.008408 | 0 | LAMA5_ITGA3_ITGB1 | LAMININ | ECM-Receptor | KEGG: hsa04512 |
| AT1 | AT1 | LAMB2 | ITGA3_ITGB1 | 0.014684 | 0 | LAMB2_ITGA3_ITGB1 | LAMININ | ECM-Receptor | KEGG: hsa04512 |
| Club | AT1 | LAMB2 | ITGA3_ITGB1 | 0.008346 | 0 | LAMB2_ITGA3_ITGB1 | LAMININ | ECM-Receptor | KEGG: hsa04512 |
| AT1 | Cil | LAMB2 | ITGA3_ITGB1 | 0.010021 | 0 | LAMB2_ITGA3_ITGB1 | LAMININ | ECM-Receptor | KEGG: hsa04512 |
| AT1 | AT1 | LAMB3 | ITGA3_ITGB1 | 0.051096 | 0 | LAMB3_ITGA3_ITGB1 | LAMININ | ECM-Receptor | KEGG: hsa04512 |
| AT2 | AT1 | LAMB3 | ITGA3_ITGB1 | 0.058812 | 0 | LAMB3_ITGA3_ITGB1 | LAMININ | ECM-Receptor | KEGG: hsa04512 |
| AT1 | Cil | LAMB3 | ITGA3_ITGB1 | 0.035284 | 0 | LAMB3_ITGA3_ITGB1 | LAMININ | ECM-Receptor | KEGG: hsa04512 |
| AT2 | Cil | LAMB3 | ITGA3_ITGB1 | 0.040715 | 0 | LAMB3_ITGA3_ITGB1 | LAMININ | ECM-Receptor | KEGG: hsa04512 |
| AT2 | AT1 | LAMC1 | ITGA3_ITGB1 | 0.006205 | 0 | LAMC1_ITGA3_ITGB1 | LAMININ | ECM-Receptor | KEGG: hsa04512 |
| AT2 | AT2 | LAMC1 | ITGA3_ITGB1 | 0.001721 | 0.01 | LAMC1_ITGA3_ITGB1 | LAMININ | ECM-Receptor | KEGG: hsa04512 |
| AT2 | Cil | LAMC1 | ITGA3_ITGB1 | 0.004223 | 0 | LAMC1_ITGA3_ITGB1 | LAMININ | ECM-Receptor | KEGG: hsa04512 |
| AT2 | Club | LAMC1 | ITGA3_ITGB1 | 0.001753 | 0.01 | LAMC1_ITGA3_ITGB1 | LAMININ | ECM-Receptor | KEGG: hsa04512 |
| AT1 | AT1 | LAMC2 | ITGA3_ITGB1 | 0.049855 | 0 | LAMC2_ITGA3_ITGB1 | LAMININ | ECM-Receptor | KEGG: hsa04512 |
| Cil | AT1 | LAMC2 | ITGA3_ITGB1 | 0.022758 | 0 | LAMC2_ITGA3_ITGB1 | LAMININ | ECM-Receptor | KEGG: hsa04512 |
| AT1 | Cil | LAMC2 | ITGA3_ITGB1 | 0.034413 | 0 | LAMC2_ITGA3_ITGB1 | LAMININ | ECM-Receptor | KEGG: hsa04512 |
| AT2 | AT1 | THBS1 | ITGA3_ITGB1 | 0.003611 | 0 | THBS1_ITGA3_ITGB1 | THBS | ECM-Receptor | KEGG: hsa04512 |
| AT2 | AT2 | THBS1 | ITGA3_ITGB1 | 0.000999 | 0 | THBS1_ITGA3_ITGB1 | THBS | ECM-Receptor | KEGG: hsa04512 |
| AT2 | Cil | THBS1 | ITGA3_ITGB1 | 0.002455 | 0 | THBS1_ITGA3_ITGB1 | THBS | ECM-Receptor | KEGG: hsa04512 |
| AT2 | Club | THBS1 | ITGA3_ITGB1 | 0.001018 | 0 | THBS1_ITGA3_ITGB1 | THBS | ECM-Receptor | KEGG: hsa04512 |
| AT1 | AT1 | COL4A1 | ITGA3_ITGB1 | 0.016906 | 0 | COL4A1_ITGA3_ITGB1 | COLLAGEN | ECM-Receptor | KEGG: hsa04512 |
| AT1 | AT2 | COL4A1 | ITGA3_ITGB1 | 0.004725 | 0.03 | COL4A1_ITGA3_ITGB1 | COLLAGEN | ECM-Receptor | KEGG: hsa04512 |
| AT1 | Cil | COL4A1 | ITGA3_ITGB1 | 0.011546 | 0 | COL4A1_ITGA3_ITGB1 | COLLAGEN | ECM-Receptor | KEGG: hsa04512 |
| AT1 | Club | COL4A1 | ITGA3_ITGB1 | 0.004814 | 0.01 | COL4A1_ITGA3_ITGB1 | COLLAGEN | ECM-Receptor | KEGG: hsa04512 |
| AT1 | AT1 | COL4A2 | ITGA3_ITGB1 | 0.039115 | 0 | COL4A2_ITGA3_ITGB1 | COLLAGEN | ECM-Receptor | KEGG: hsa04512 |
| AT1 | AT2 | COL4A2 | ITGA3_ITGB1 | 0.011112 | 0 | COL4A2_ITGA3_ITGB1 | COLLAGEN | ECM-Receptor | KEGG: hsa04512 |
| AT1 | Cil | COL4A2 | ITGA3_ITGB1 | 0.026905 | 0 | COL4A2_ITGA3_ITGB1 | COLLAGEN | ECM-Receptor | KEGG: hsa04512 |
| AT1 | Club | COL4A2 | ITGA3_ITGB1 | 0.01132 | 0 | COL4A2_ITGA3_ITGB1 | COLLAGEN | ECM-Receptor | KEGG: hsa04512 |
| AT1 | AT1 | COL4A3 | ITGA3_ITGB1 | 0.018014 | 0 | COL4A3_ITGA3_ITGB1 | COLLAGEN | ECM-Receptor | KEGG: hsa04512 |
| AT1 | Cil | COL4A3 | ITGA3_ITGB1 | 0.012307 | 0 | COL4A3_ITGA3_ITGB1 | COLLAGEN | ECM-Receptor | KEGG: hsa04512 |
| AT1 | AT1 | COL4A4 | ITGA3_ITGB1 | 0.015366 | 0 | COL4A4_ITGA3_ITGB1 | COLLAGEN | ECM-Receptor | KEGG: hsa04512 |
| AT1 | Cil | COL4A4 | ITGA3_ITGB1 | 0.010489 | 0 | COL4A4_ITGA3_ITGB1 | COLLAGEN | ECM-Receptor | KEGG: hsa04512 |
| AT1 | AT2 | LAMA3 | ITGA6_ITGB1 | 0.010981 | 0 | LAMA3_ITGA6_ITGB1 | LAMININ | ECM-Receptor | KEGG: hsa04512 |
| AT2 | AT2 | LAMA3 | ITGA6_ITGB1 | 0.001265 | 0 | LAMA3_ITGA6_ITGB1 | LAMININ | ECM-Receptor | KEGG: hsa04512 |
| AT1 | AT2 | LAMA5 | ITGA6_ITGB1 | 0.003512 | 0 | LAMA5_ITGA6_ITGB1 | LAMININ | ECM-Receptor | KEGG: hsa04512 |
| Cil | AT2 | LAMA5 | ITGA6_ITGB1 | 0.002246 | 0 | LAMA5_ITGA6_ITGB1 | LAMININ | ECM-Receptor | KEGG: hsa04512 |
| Club | AT2 | LAMA5 | ITGA6_ITGB1 | 0.00213 | 0 | LAMA5_ITGA6_ITGB1 | LAMININ | ECM-Receptor | KEGG: hsa04512 |
| AT1 | AT2 | LAMB2 | ITGA6_ITGB1 | 0.00419 | 0 | LAMB2_ITGA6_ITGB1 | LAMININ | ECM-Receptor | KEGG: hsa04512 |
| AT2 | AT2 | LAMB2 | ITGA6_ITGB1 | 0.00168 | 0 | LAMB2_ITGA6_ITGB1 | LAMININ | ECM-Receptor | KEGG: hsa04512 |
| Cil | AT2 | LAMB2 | ITGA6_ITGB1 | 0.000463 | 0 | LAMB2_ITGA6_ITGB1 | LAMININ | ECM-Receptor | KEGG: hsa04512 |
| Club | AT2 | LAMB2 | ITGA6_ITGB1 | 0.002371 | 0 | LAMB2_ITGA6_ITGB1 | LAMININ | ECM-Receptor | KEGG: hsa04512 |
| AT1 | AT2 | LAMB3 | ITGA6_ITGB1 | 0.014975 | 0 | LAMB3_ITGA6_ITGB1 | LAMININ | ECM-Receptor | KEGG: hsa04512 |
| AT2 | AT2 | LAMB3 | ITGA6_ITGB1 | 0.017336 | 0 | LAMB3_ITGA6_ITGB1 | LAMININ | ECM-Receptor | KEGG: hsa04512 |
| Cil | AT2 | LAMB3 | ITGA6_ITGB1 | 0.002737 | 0 | LAMB3_ITGA6_ITGB1 | LAMININ | ECM-Receptor | KEGG: hsa04512 |
| Club | AT2 | LAMB3 | ITGA6_ITGB1 | 0.002427 | 0 | LAMB3_ITGA6_ITGB1 | LAMININ | ECM-Receptor | KEGG: hsa04512 |
| AT2 | AT2 | LAMC1 | ITGA6_ITGB1 | 0.00176 | 0 | LAMC1_ITGA6_ITGB1 | LAMININ | ECM-Receptor | KEGG: hsa04512 |
| AT1 | AT2 | LAMC2 | ITGA6_ITGB1 | 0.014598 | 0 | LAMC2_ITGA6_ITGB1 | LAMININ | ECM-Receptor | KEGG: hsa04512 |
| AT2 | AT2 | LAMC2 | ITGA6_ITGB1 | 0.002468 | 0 | LAMC2_ITGA6_ITGB1 | LAMININ | ECM-Receptor | KEGG: hsa04512 |
| Cil | AT2 | LAMC2 | ITGA6_ITGB1 | 0.006532 | 0 | LAMC2_ITGA6_ITGB1 | LAMININ | ECM-Receptor | KEGG: hsa04512 |
| Club | AT2 | LAMC2 | ITGA6_ITGB1 | 0.00219 | 0 | LAMC2_ITGA6_ITGB1 | LAMININ | ECM-Receptor | KEGG: hsa04512 |
| AT2 | AT2 | FN1 | ITGAV_ITGB1 | 0.001624 | 0 | FN1_ITGAV_ITGB1 | FN1 | ECM-Receptor | KEGG: hsa04512 |
| AT2 | AT2 | TNC | ITGAV_ITGB6 | 0.002648 | 0 | TNC_ITGAV_ITGB6 | TENASCIN | ECM-Receptor | KEGG: hsa04512 |
| AT2 | AT2 | FN1 | ITGAV_ITGB6 | 0.002315 | 0 | FN1_ITGAV_ITGB6 | FN1 | ECM-Receptor | KEGG: hsa04512 |
| AT2 | AT2 | FN1 | CD44 | 0.010269 | 0 | FN1_CD44 | FN1 | ECM-Receptor | KEGG: hsa04512 |
| AT2 | Club | FN1 | CD44 | 0.002497 | 0 | FN1_CD44 | FN1 | ECM-Receptor | KEGG: hsa04512 |
| AT1 | AT2 | COL4A1 | CD44 | 0.029696 | 0 | COL4A1_CD44 | COLLAGEN | ECM-Receptor | KEGG: hsa04512 |
| AT1 | Club | COL4A1 | CD44 | 0.007329 | 0 | COL4A1_CD44 | COLLAGEN | ECM-Receptor | KEGG: hsa04512 |
| AT1 | AT2 | COL4A2 | CD44 | 0.067552 | 0 | COL4A2_CD44 | COLLAGEN | ECM-Receptor | KEGG: hsa04512 |
| AT1 | Club | COL4A2 | CD44 | 0.017176 | 0 | COL4A2_CD44 | COLLAGEN | ECM-Receptor | KEGG: hsa04512 |
| AT1 | AT2 | COL4A3 | CD44 | 0.031615 | 0 | COL4A3_CD44 | COLLAGEN | ECM-Receptor | KEGG: hsa04512 |
| AT1 | Club | COL4A3 | CD44 | 0.007814 | 0 | COL4A3_CD44 | COLLAGEN | ECM-Receptor | KEGG: hsa04512 |
| AT1 | AT2 | COL4A4 | CD44 | 0.027022 | 0 | COL4A4_CD44 | COLLAGEN | ECM-Receptor | KEGG: hsa04512 |
| AT1 | Club | COL4A4 | CD44 | 0.006655 | 0 | COL4A4_CD44 | COLLAGEN | ECM-Receptor | KEGG: hsa04512 |
| AT1 | AT2 | LAMA3 | CD44 | 0.065411 | 0 | LAMA3_CD44 | LAMININ | ECM-Receptor | KEGG: hsa04512 |
| AT2 | AT2 | LAMA3 | CD44 | 0.007924 | 0 | LAMA3_CD44 | LAMININ | ECM-Receptor | KEGG: hsa04512 |
| AT1 | Club | LAMA3 | CD44 | 0.016603 | 0 | LAMA3_CD44 | LAMININ | ECM-Receptor | KEGG: hsa04512 |
| AT2 | Club | LAMA3 | CD44 | 0.001923 | 0.03 | LAMA3_CD44 | LAMININ | ECM-Receptor | KEGG: hsa04512 |
| AT1 | AT2 | LAMA5 | CD44 | 0.021735 | 0 | LAMA5_CD44 | LAMININ | ECM-Receptor | KEGG: hsa04512 |
| Cil | AT2 | LAMA5 | CD44 | 0.013988 | 0 | LAMA5_CD44 | LAMININ | ECM-Receptor | KEGG: hsa04512 |
| Club | AT2 | LAMA5 | CD44 | 0.013274 | 0 | LAMA5_CD44 | LAMININ | ECM-Receptor | KEGG: hsa04512 |
| AT1 | Club | LAMA5 | CD44 | 0.005331 | 0 | LAMA5_CD44 | LAMININ | ECM-Receptor | KEGG: hsa04512 |
| Cil | Club | LAMA5 | CD44 | 0.003411 | 0 | LAMA5_CD44 | LAMININ | ECM-Receptor | KEGG: hsa04512 |
| Club | Club | LAMA5 | CD44 | 0.003235 | 0 | LAMA5_CD44 | LAMININ | ECM-Receptor | KEGG: hsa04512 |
| AT1 | AT2 | LAMB2 | CD44 | 0.025837 | 0 | LAMB2_CD44 | LAMININ | ECM-Receptor | KEGG: hsa04512 |
| AT2 | AT2 | LAMB2 | CD44 | 0.010494 | 0 | LAMB2_CD44 | LAMININ | ECM-Receptor | KEGG: hsa04512 |
| Cil | AT2 | LAMB2 | CD44 | 0.002913 | 0 | LAMB2_CD44 | LAMININ | ECM-Receptor | KEGG: hsa04512 |
| Club | AT2 | LAMB2 | CD44 | 0.014758 | 0 | LAMB2_CD44 | LAMININ | ECM-Receptor | KEGG: hsa04512 |
| AT1 | Club | LAMB2 | CD44 | 0.006357 | 0 | LAMB2_CD44 | LAMININ | ECM-Receptor | KEGG: hsa04512 |
| AT2 | Club | LAMB2 | CD44 | 0.002552 | 0 | LAMB2_CD44 | LAMININ | ECM-Receptor | KEGG: hsa04512 |
| Club | Club | LAMB2 | CD44 | 0.0036 | 0 | LAMB2_CD44 | LAMININ | ECM-Receptor | KEGG: hsa04512 |
| AT1 | AT2 | LAMB3 | CD44 | 0.087451 | 0 | LAMB3_CD44 | LAMININ | ECM-Receptor | KEGG: hsa04512 |
| AT2 | AT2 | LAMB3 | CD44 | 0.100078 | 0 | LAMB3_CD44 | LAMININ | ECM-Receptor | KEGG: hsa04512 |
| Cil | AT2 | LAMB3 | CD44 | 0.017006 | 0 | LAMB3_CD44 | LAMININ | ECM-Receptor | KEGG: hsa04512 |
| Club | AT2 | LAMB3 | CD44 | 0.015104 | 0 | LAMB3_CD44 | LAMININ | ECM-Receptor | KEGG: hsa04512 |
| AT1 | Club | LAMB3 | CD44 | 0.022595 | 0 | LAMB3_CD44 | LAMININ | ECM-Receptor | KEGG: hsa04512 |
| AT2 | Club | LAMB3 | CD44 | 0.026125 | 0 | LAMB3_CD44 | LAMININ | ECM-Receptor | KEGG: hsa04512 |
| Cil | Club | LAMB3 | CD44 | 0.004156 | 0.02 | LAMB3_CD44 | LAMININ | ECM-Receptor | KEGG: hsa04512 |
| AT2 | AT2 | LAMC1 | CD44 | 0.01099 | 0 | LAMC1_CD44 | LAMININ | ECM-Receptor | KEGG: hsa04512 |
| AT2 | Club | LAMC1 | CD44 | 0.002673 | 0 | LAMC1_CD44 | LAMININ | ECM-Receptor | KEGG: hsa04512 |
| AT1 | AT2 | LAMC2 | CD44 | 0.085406 | 0 | LAMC2_CD44 | LAMININ | ECM-Receptor | KEGG: hsa04512 |
| AT2 | AT2 | LAMC2 | CD44 | 0.015356 | 0 | LAMC2_CD44 | LAMININ | ECM-Receptor | KEGG: hsa04512 |
| Cil | AT2 | LAMC2 | CD44 | 0.039797 | 0 | LAMC2_CD44 | LAMININ | ECM-Receptor | KEGG: hsa04512 |
| Club | AT2 | LAMC2 | CD44 | 0.013646 | 0 | LAMC2_CD44 | LAMININ | ECM-Receptor | KEGG: hsa04512 |
| AT1 | Club | LAMC2 | CD44 | 0.02203 | 0 | LAMC2_CD44 | LAMININ | ECM-Receptor | KEGG: hsa04512 |
| Cil | Club | LAMC2 | CD44 | 0.009899 | 0 | LAMC2_CD44 | LAMININ | ECM-Receptor | KEGG: hsa04512 |
| Club | Club | LAMC2 | CD44 | 0.003326 | 0.02 | LAMC2_CD44 | LAMININ | ECM-Receptor | KEGG: hsa04512 |
| AT1 | AT1 | COL4A1 | SDC1 | 0.006345 | 0 | COL4A1_SDC1 | COLLAGEN | ECM-Receptor | KEGG: hsa04512 |
| AT1 | AT2 | COL4A1 | SDC1 | 0.00747 | 0 | COL4A1_SDC1 | COLLAGEN | ECM-Receptor | KEGG: hsa04512 |
| AT1 | Club | COL4A1 | SDC1 | 0.005584 | 0 | COL4A1_SDC1 | COLLAGEN | ECM-Receptor | KEGG: hsa04512 |
| AT1 | AT1 | COL4A2 | SDC1 | 0.01489 | 0 | COL4A2_SDC1 | COLLAGEN | ECM-Receptor | KEGG: hsa04512 |
| AT1 | AT2 | COL4A2 | SDC1 | 0.017504 | 0 | COL4A2_SDC1 | COLLAGEN | ECM-Receptor | KEGG: hsa04512 |
| AT1 | Club | COL4A2 | SDC1 | 0.013117 | 0 | COL4A2_SDC1 | COLLAGEN | ECM-Receptor | KEGG: hsa04512 |
| AT1 | AT1 | COL4A3 | SDC1 | 0.006765 | 0 | COL4A3_SDC1 | COLLAGEN | ECM-Receptor | KEGG: hsa04512 |
| AT1 | AT2 | COL4A3 | SDC1 | 0.007965 | 0 | COL4A3_SDC1 | COLLAGEN | ECM-Receptor | KEGG: hsa04512 |
| AT1 | Club | COL4A3 | SDC1 | 0.005954 | 0 | COL4A3_SDC1 | COLLAGEN | ECM-Receptor | KEGG: hsa04512 |
| AT1 | AT1 | COL4A4 | SDC1 | 0.005761 | 0 | COL4A4_SDC1 | COLLAGEN | ECM-Receptor | KEGG: hsa04512 |
| AT1 | AT2 | COL4A4 | SDC1 | 0.006784 | 0 | COL4A4_SDC1 | COLLAGEN | ECM-Receptor | KEGG: hsa04512 |
| AT1 | Club | COL4A4 | SDC1 | 0.00507 | 0 | COL4A4_SDC1 | COLLAGEN | ECM-Receptor | KEGG: hsa04512 |
| AT2 | AT1 | FN1 | SDC1 | 0.00216 | 0 | FN1_SDC1 | FN1 | ECM-Receptor | KEGG: hsa04512 |
| AT2 | AT2 | FN1 | SDC1 | 0.002545 | 0 | FN1_SDC1 | FN1 | ECM-Receptor | KEGG: hsa04512 |
| AT2 | Club | FN1 | SDC1 | 0.0019 | 0 | FN1_SDC1 | FN1 | ECM-Receptor | KEGG: hsa04512 |
| AT2 | AT1 | TNC | SDC1 | 0.002471 | 0 | TNC_SDC1 | TENASCIN | ECM-Receptor | KEGG: hsa04512 |
| AT2 | AT2 | TNC | SDC1 | 0.002911 | 0 | TNC_SDC1 | TENASCIN | ECM-Receptor | KEGG: hsa04512 |
| AT2 | Club | TNC | SDC1 | 0.002173 | 0 | TNC_SDC1 | TENASCIN | ECM-Receptor | KEGG: hsa04512 |
| AT2 | AT1 | THBS1 | SDC1 | 0.001344 | 0 | THBS1_SDC1 | THBS | ECM-Receptor | KEGG: hsa04512 |
| AT2 | AT2 | THBS1 | SDC1 | 0.001584 | 0 | THBS1_SDC1 | THBS | ECM-Receptor | KEGG: hsa04512 |
| AT2 | Club | THBS1 | SDC1 | 0.001182 | 0 | THBS1_SDC1 | THBS | ECM-Receptor | KEGG: hsa04512 |
| AT1 | AT1 | COL4A1 | SDC4 | 0.029163 | 0 | COL4A1_SDC4 | COLLAGEN | ECM-Receptor | KEGG: hsa04512 |
| AT1 | AT2 | COL4A1 | SDC4 | 0.036525 | 0 | COL4A1_SDC4 | COLLAGEN | ECM-Receptor | KEGG: hsa04512 |
| AT1 | Cil | COL4A1 | SDC4 | 0.022103 | 0 | COL4A1_SDC4 | COLLAGEN | ECM-Receptor | KEGG: hsa04512 |
| AT1 | Club | COL4A1 | SDC4 | 0.028644 | 0 | COL4A1_SDC4 | COLLAGEN | ECM-Receptor | KEGG: hsa04512 |
| AT1 | AT1 | COL4A2 | SDC4 | 0.066386 | 0 | COL4A2_SDC4 | COLLAGEN | ECM-Receptor | KEGG: hsa04512 |
| AT1 | AT2 | COL4A2 | SDC4 | 0.082348 | 0 | COL4A2_SDC4 | COLLAGEN | ECM-Receptor | KEGG: hsa04512 |
| AT1 | Cil | COL4A2 | SDC4 | 0.050785 | 0 | COL4A2_SDC4 | COLLAGEN | ECM-Receptor | KEGG: hsa04512 |
| AT1 | Club | COL4A2 | SDC4 | 0.065248 | 0 | COL4A2_SDC4 | COLLAGEN | ECM-Receptor | KEGG: hsa04512 |
| AT1 | AT1 | COL4A3 | SDC4 | 0.031049 | 0 | COL4A3_SDC4 | COLLAGEN | ECM-Receptor | KEGG: hsa04512 |
| AT1 | AT2 | COL4A3 | SDC4 | 0.038867 | 0 | COL4A3_SDC4 | COLLAGEN | ECM-Receptor | KEGG: hsa04512 |
| AT1 | Cil | COL4A3 | SDC4 | 0.023543 | 0 | COL4A3_SDC4 | COLLAGEN | ECM-Receptor | KEGG: hsa04512 |
| AT1 | Club | COL4A3 | SDC4 | 0.030496 | 0 | COL4A3_SDC4 | COLLAGEN | ECM-Receptor | KEGG: hsa04512 |
| AT1 | AT1 | COL4A4 | SDC4 | 0.026536 | 0 | COL4A4_SDC4 | COLLAGEN | ECM-Receptor | KEGG: hsa04512 |
| AT1 | AT2 | COL4A4 | SDC4 | 0.033258 | 0 | COL4A4_SDC4 | COLLAGEN | ECM-Receptor | KEGG: hsa04512 |
| AT1 | Cil | COL4A4 | SDC4 | 0.020099 | 0 | COL4A4_SDC4 | COLLAGEN | ECM-Receptor | KEGG: hsa04512 |
| AT1 | Club | COL4A4 | SDC4 | 0.026062 | 0 | COL4A4_SDC4 | COLLAGEN | ECM-Receptor | KEGG: hsa04512 |
| AT2 | AT1 | FN1 | SDC4 | 0.010081 | 0 | FN1_SDC4 | FN1 | ECM-Receptor | KEGG: hsa04512 |
| AT2 | AT2 | FN1 | SDC4 | 0.012689 | 0 | FN1_SDC4 | FN1 | ECM-Receptor | KEGG: hsa04512 |
| AT2 | Cil | FN1 | SDC4 | 0.007604 | 0.01 | FN1_SDC4 | FN1 | ECM-Receptor | KEGG: hsa04512 |
| AT2 | Club | FN1 | SDC4 | 0.009898 | 0 | FN1_SDC4 | FN1 | ECM-Receptor | KEGG: hsa04512 |
| AT2 | AT1 | TNC | SDC4 | 0.011518 | 0 | TNC_SDC4 | TENASCIN | ECM-Receptor | KEGG: hsa04512 |
| AT2 | AT2 | TNC | SDC4 | 0.014492 | 0 | TNC_SDC4 | TENASCIN | ECM-Receptor | KEGG: hsa04512 |
| AT2 | Cil | TNC | SDC4 | 0.008691 | 0 | TNC_SDC4 | TENASCIN | ECM-Receptor | KEGG: hsa04512 |
| AT2 | Club | TNC | SDC4 | 0.011309 | 0 | TNC_SDC4 | TENASCIN | ECM-Receptor | KEGG: hsa04512 |
| AT2 | AT1 | THBS1 | SDC4 | 0.00629 | 0 | THBS1_SDC4 | THBS | ECM-Receptor | KEGG: hsa04512 |
| AT2 | AT2 | THBS1 | SDC4 | 0.007925 | 0 | THBS1_SDC4 | THBS | ECM-Receptor | KEGG: hsa04512 |
| AT2 | Cil | THBS1 | SDC4 | 0.00474 | 0 | THBS1_SDC4 | THBS | ECM-Receptor | KEGG: hsa04512 |
| AT2 | Club | THBS1 | SDC4 | 0.006176 | 0 | THBS1_SDC4 | THBS | ECM-Receptor | KEGG: hsa04512 |
| AT2 | AT2 | THBS1 | CD36 | 0.000637 | 0 | THBS1_CD36 | THBS | ECM-Receptor | KEGG: hsa04512 |
| AT2 | AT1 | THBS1 | CD47 | 0.003554 | 0 | THBS1_CD47 | THBS | ECM-Receptor | KEGG: hsa04512 |
| AT2 | AT2 | THBS1 | CD47 | 0.002332 | 0 | THBS1_CD47 | THBS | ECM-Receptor | KEGG: hsa04512 |
| AT2 | Cil | THBS1 | CD47 | 0.002491 | 0 | THBS1_CD47 | THBS | ECM-Receptor | KEGG: hsa04512 |
| AT2 | Club | THBS1 | CD47 | 0.002531 | 0 | THBS1_CD47 | THBS | ECM-Receptor | KEGG: hsa04512 |
| AT1 | Cil | AGRN | DAG1 | 0.00172 | 0 | AGRN_DAG1 | AGRN | ECM-Receptor | KEGG: hsa04512 |
| Cil | Cil | AGRN | DAG1 | 0.000796 | 0.01 | AGRN_DAG1 | AGRN | ECM-Receptor | KEGG: hsa04512 |
| Club | Cil | AGRN | DAG1 | 0.00087 | 0.01 | AGRN_DAG1 | AGRN | ECM-Receptor | KEGG: hsa04512 |
| AT1 | Cil | HSPG2 | DAG1 | 0.001943 | 0 | HSPG2_DAG1 | HSPG | ECM-Receptor | KEGG: hsa04512 |
| Club | Cil | HSPG2 | DAG1 | 0.000391 | 0.01 | HSPG2_DAG1 | HSPG | ECM-Receptor | KEGG: hsa04512 |
| AT1 | AT2 | LAMA3 | DAG1 | 0.00371 | 0 | LAMA3_DAG1 | LAMININ | ECM-Receptor | KEGG: hsa04512 |
| AT1 | Cil | LAMA3 | DAG1 | 0.004324 | 0 | LAMA3_DAG1 | LAMININ | ECM-Receptor | KEGG: hsa04512 |
| AT2 | Cil | LAMA3 | DAG1 | 0.000495 | 0.01 | LAMA3_DAG1 | LAMININ | ECM-Receptor | KEGG: hsa04512 |
| AT1 | Cil | LAMA5 | DAG1 | 0.001377 | 0 | LAMA5_DAG1 | LAMININ | ECM-Receptor | KEGG: hsa04512 |
| Cil | Cil | LAMA5 | DAG1 | 0.000879 | 0.01 | LAMA5_DAG1 | LAMININ | ECM-Receptor | KEGG: hsa04512 |
| Club | Cil | LAMA5 | DAG1 | 0.000834 | 0.01 | LAMA5_DAG1 | LAMININ | ECM-Receptor | KEGG: hsa04512 |
| AT1 | Cil | LAMB2 | DAG1 | 0.001643 | 0 | LAMB2_DAG1 | LAMININ | ECM-Receptor | KEGG: hsa04512 |
| AT2 | Cil | LAMB2 | DAG1 | 0.000658 | 0.01 | LAMB2_DAG1 | LAMININ | ECM-Receptor | KEGG: hsa04512 |
| Cil | Cil | LAMB2 | DAG1 | 0.000181 | 0.02 | LAMB2_DAG1 | LAMININ | ECM-Receptor | KEGG: hsa04512 |
| Club | Cil | LAMB2 | DAG1 | 0.000929 | 0.01 | LAMB2_DAG1 | LAMININ | ECM-Receptor | KEGG: hsa04512 |
| AT1 | AT2 | LAMB3 | DAG1 | 0.005072 | 0 | LAMB3_DAG1 | LAMININ | ECM-Receptor | KEGG: hsa04512 |
| AT2 | AT2 | LAMB3 | DAG1 | 0.005881 | 0.02 | LAMB3_DAG1 | LAMININ | ECM-Receptor | KEGG: hsa04512 |
| AT1 | Cil | LAMB3 | DAG1 | 0.005911 | 0 | LAMB3_DAG1 | LAMININ | ECM-Receptor | KEGG: hsa04512 |
| AT2 | Cil | LAMB3 | DAG1 | 0.006853 | 0 | LAMB3_DAG1 | LAMININ | ECM-Receptor | KEGG: hsa04512 |
| Cil | Cil | LAMB3 | DAG1 | 0.001072 | 0.01 | LAMB3_DAG1 | LAMININ | ECM-Receptor | KEGG: hsa04512 |
| Club | Cil | LAMB3 | DAG1 | 0.000951 | 0.01 | LAMB3_DAG1 | LAMININ | ECM-Receptor | KEGG: hsa04512 |
| AT2 | AT2 | LAMC1 | DAG1 | 0.000591 | 0.01 | LAMC1_DAG1 | LAMININ | ECM-Receptor | KEGG: hsa04512 |
| AT2 | Cil | LAMC1 | DAG1 | 0.000689 | 0 | LAMC1_DAG1 | LAMININ | ECM-Receptor | KEGG: hsa04512 |
| AT1 | AT2 | LAMC2 | DAG1 | 0.004943 | 0 | LAMC2_DAG1 | LAMININ | ECM-Receptor | KEGG: hsa04512 |
| AT1 | Cil | LAMC2 | DAG1 | 0.00576 | 0 | LAMC2_DAG1 | LAMININ | ECM-Receptor | KEGG: hsa04512 |
| AT2 | Cil | LAMC2 | DAG1 | 0.000967 | 0.01 | LAMC2_DAG1 | LAMININ | ECM-Receptor | KEGG: hsa04512 |
| Cil | Cil | LAMC2 | DAG1 | 0.002565 | 0 | LAMC2_DAG1 | LAMININ | ECM-Receptor | KEGG: hsa04512 |
| Club | Cil | LAMC2 | DAG1 | 0.000858 | 0.01 | LAMC2_DAG1 | LAMININ | ECM-Receptor | KEGG: hsa04512 |
| AT2 | AT1 | ADGRE5 | CD55 | 0.059926 | 0 | ADGRE5_CD55 | ADGRE5 | Cell-Cell Contact | PMID: 11297558 |
| Cil | AT1 | ADGRE5 | CD55 | 0.017456 | 0 | ADGRE5_CD55 | ADGRE5 | Cell-Cell Contact | PMID: 11297558 |
| Club | AT1 | ADGRE5 | CD55 | 0.009628 | 0 | ADGRE5_CD55 | ADGRE5 | Cell-Cell Contact | PMID: 11297558 |
| AT2 | AT2 | ADGRE5 | CD55 | 0.031514 | 0 | ADGRE5_CD55 | ADGRE5 | Cell-Cell Contact | PMID: 11297558 |
| Cil | AT2 | ADGRE5 | CD55 | 0.008987 | 0 | ADGRE5_CD55 | ADGRE5 | Cell-Cell Contact | PMID: 11297558 |
| Club | AT2 | ADGRE5 | CD55 | 0.004938 | 0.02 | ADGRE5_CD55 | ADGRE5 | Cell-Cell Contact | PMID: 11297558 |
| AT2 | Cil | ADGRE5 | CD55 | 0.030739 | 0 | ADGRE5_CD55 | ADGRE5 | Cell-Cell Contact | PMID: 11297558 |
| Cil | Cil | ADGRE5 | CD55 | 0.008762 | 0 | ADGRE5_CD55 | ADGRE5 | Cell-Cell Contact | PMID: 11297558 |
| Club | Cil | ADGRE5 | CD55 | 0.004813 | 0.02 | ADGRE5_CD55 | ADGRE5 | Cell-Cell Contact | PMID: 11297558 |
| AT2 | Club | ADGRE5 | CD55 | 0.039551 | 0 | ADGRE5_CD55 | ADGRE5 | Cell-Cell Contact | PMID: 11297558 |
| Cil | Club | ADGRE5 | CD55 | 0.011347 | 0 | ADGRE5_CD55 | ADGRE5 | Cell-Cell Contact | PMID: 11297558 |
| Club | Club | ADGRE5 | CD55 | 0.006241 | 0.01 | ADGRE5_CD55 | ADGRE5 | Cell-Cell Contact | PMID: 11297558 |
| AT1 | AT2 | APP | CD74 | 0.037239 | 0 | APP_CD74 | APP | Cell-Cell Contact | PMID: 19849849 |
| Cil | AT2 | APP | CD74 | 0.100435 | 0 | APP_CD74 | APP | Cell-Cell Contact | PMID: 19849849 |
| Club | AT2 | APP | CD74 | 0.055044 | 0 | APP_CD74 | APP | Cell-Cell Contact | PMID: 19849849 |
| Cil | Cil | APP | CD74 | 0.063484 | 0 | APP_CD74 | APP | Cell-Cell Contact | PMID: 19849849 |
| Club | Cil | APP | CD74 | 0.034159 | 0 | APP_CD74 | APP | Cell-Cell Contact | PMID: 19849849 |
| AT1 | Club | APP | CD74 | 0.027977 | 0 | APP_CD74 | APP | Cell-Cell Contact | PMID: 19849849 |
| Cil | Club | APP | CD74 | 0.076708 | 0 | APP_CD74 | APP | Cell-Cell Contact | PMID: 19849849 |
| Club | Club | APP | CD74 | 0.041545 | 0 | APP_CD74 | APP | Cell-Cell Contact | PMID: 19849849 |
| AT1 | AT1 | CADM1 | CADM1 | 0.052519 | 0 | CADM1_CADM1 | CADM | Cell-Cell Contact | PMID: 24503895 |
| AT2 | AT1 | CADM1 | CADM1 | 0.027074 | 0 | CADM1_CADM1 | CADM | Cell-Cell Contact | PMID: 24503895 |
| AT1 | AT2 | CADM1 | CADM1 | 0.027074 | 0 | CADM1_CADM1 | CADM | Cell-Cell Contact | PMID: 24503895 |
| AT2 | AT2 | CADM1 | CADM1 | 0.013777 | 0.02 | CADM1_CADM1 | CADM | Cell-Cell Contact | PMID: 24503895 |
| AT1 | Cil | CD46 | JAG1 | 0.002108 | 0 | CD46_JAG1 | CD46 | Cell-Cell Contact | PMID: 23086448 |
| AT2 | Cil | CD46 | JAG1 | 0.005389 | 0 | CD46_JAG1 | CD46 | Cell-Cell Contact | PMID: 23086448 |
| Cil | Cil | CD46 | JAG1 | 0.007539 | 0 | CD46_JAG1 | CD46 | Cell-Cell Contact | PMID: 23086448 |
| Club | Cil | CD46 | JAG1 | 0.00726 | 0 | CD46_JAG1 | CD46 | Cell-Cell Contact | PMID: 23086448 |
| AT1 | AT1 | CD99 | CD99 | 0.002376 | 0 | CD99_CD99 | CD99 | Cell-Cell Contact | KEGG: hsa04514 |
| Cil | AT1 | CD99 | CD99 | 0.006663 | 0 | CD99_CD99 | CD99 | Cell-Cell Contact | KEGG: hsa04514 |
| Cil | AT2 | CD99 | CD99 | 0.003444 | 0 | CD99_CD99 | CD99 | Cell-Cell Contact | KEGG: hsa04514 |
| AT1 | Cil | CD99 | CD99 | 0.006663 | 0 | CD99_CD99 | CD99 | Cell-Cell Contact | KEGG: hsa04514 |
| AT2 | Cil | CD99 | CD99 | 0.003444 | 0 | CD99_CD99 | CD99 | Cell-Cell Contact | KEGG: hsa04514 |
| Cil | Cil | CD99 | CD99 | 0.01854 | 0 | CD99_CD99 | CD99 | Cell-Cell Contact | KEGG: hsa04514 |
| Club | Cil | CD99 | CD99 | 0.003891 | 0 | CD99_CD99 | CD99 | Cell-Cell Contact | KEGG: hsa04514 |
| Cil | Club | CD99 | CD99 | 0.003891 | 0 | CD99_CD99 | CD99 | Cell-Cell Contact | KEGG: hsa04514 |
| Cil | AT1 | CDH1 | CDH1 | 0.009419 | 0 | CDH1_CDH1 | CDH | Cell-Cell Contact | KEGG: hsa04514 |
| Cil | AT2 | CDH1 | CDH1 | 0.016675 | 0 | CDH1_CDH1 | CDH | Cell-Cell Contact | KEGG: hsa04514 |
| Club | AT2 | CDH1 | CDH1 | 0.009708 | 0.04 | CDH1_CDH1 | CDH | Cell-Cell Contact | KEGG: hsa04514 |
| AT1 | Cil | CDH1 | CDH1 | 0.009419 | 0 | CDH1_CDH1 | CDH | Cell-Cell Contact | KEGG: hsa04514 |
| AT2 | Cil | CDH1 | CDH1 | 0.016675 | 0 | CDH1_CDH1 | CDH | Cell-Cell Contact | KEGG: hsa04514 |
| Cil | Cil | CDH1 | CDH1 | 0.019269 | 0 | CDH1_CDH1 | CDH | Cell-Cell Contact | KEGG: hsa04514 |
| Club | Cil | CDH1 | CDH1 | 0.01123 | 0 | CDH1_CDH1 | CDH | Cell-Cell Contact | KEGG: hsa04514 |
| AT2 | Club | CDH1 | CDH1 | 0.009708 | 0.04 | CDH1_CDH1 | CDH | Cell-Cell Contact | KEGG: hsa04514 |
| Cil | Club | CDH1 | CDH1 | 0.01123 | 0 | CDH1_CDH1 | CDH | Cell-Cell Contact | KEGG: hsa04514 |
| AT1 | AT2 | EFNA1 | EPHA2 | 0.018636 | 0 | EFNA1_EPHA2 | EPHA | Cell-Cell Contact | PMID: 15114347 |
| Cil | AT2 | EFNA1 | EPHA2 | 0.011498 | 0 | EFNA1_EPHA2 | EPHA | Cell-Cell Contact | PMID: 15114347 |
| AT1 | Cil | EFNA1 | EPHA2 | 0.006617 | 0 | EFNA1_EPHA2 | EPHA | Cell-Cell Contact | PMID: 15114347 |
| AT1 | Club | EFNA1 | EPHA2 | 0.006954 | 0 | EFNA1_EPHA2 | EPHA | Cell-Cell Contact | PMID: 15114347 |
| Cil | Club | EFNA1 | EPHA2 | 0.004271 | 0.03 | EFNA1_EPHA2 | EPHA | Cell-Cell Contact | PMID: 15114347 |
| AT2 | AT2 | ESAM | ESAM | 0.000685 | 0 | ESAM_ESAM | ESAM | Cell-Cell Contact | KEGG: hsa04514; PMID: 11279107 |
| AT2 | AT2 | MPZL1 | MPZL1 | 0.00104 | 0.03 | MPZL1_MPZL1 | MPZ | Cell-Cell Contact | KEGG: hsa04514 |
| Cil | AT2 | MPZL1 | MPZL1 | 0.000506 | 0.01 | MPZL1_MPZL1 | MPZ | Cell-Cell Contact | KEGG: hsa04514 |
| Club | AT2 | MPZL1 | MPZL1 | 0.000701 | 0 | MPZL1_MPZL1 | MPZ | Cell-Cell Contact | KEGG: hsa04514 |
| AT2 | Cil | MPZL1 | MPZL1 | 0.000506 | 0.01 | MPZL1_MPZL1 | MPZ | Cell-Cell Contact | KEGG: hsa04514 |
| Club | Cil | MPZL1 | MPZL1 | 0.000341 | 0 | MPZL1_MPZL1 | MPZ | Cell-Cell Contact | KEGG: hsa04514 |
| AT2 | Club | MPZL1 | MPZL1 | 0.000701 | 0 | MPZL1_MPZL1 | MPZ | Cell-Cell Contact | KEGG: hsa04514 |
| Cil | Club | MPZL1 | MPZL1 | 0.000341 | 0 | MPZL1_MPZL1 | MPZ | Cell-Cell Contact | KEGG: hsa04514 |
| Club | Club | MPZL1 | MPZL1 | 0.000473 | 0 | MPZL1_MPZL1 | MPZ | Cell-Cell Contact | KEGG: hsa04514 |
| Club | Cil | NECTIN1 | NECTIN4 | 0.00076 | 0 | NECTIN1_NECTIN4 | NECTIN | Cell-Cell Contact | PMID: 23027581 |
| Club | Club | NECTIN1 | NECTIN4 | 0.000814 | 0 | NECTIN1_NECTIN4 | NECTIN | Cell-Cell Contact | PMID: 23027581 |
| Cil | Club | JAG1 | NOTCH2 | 0.000419 | 0 | JAG1_NOTCH2 | NOTCH | Cell-Cell Contact | PMID: 22353464 |
| Cil | Club | JAG1 | NOTCH3 | 0.000573 | 0 | JAG1_NOTCH3 | NOTCH | Cell-Cell Contact | PMID: 22353464 |
| AT1 | AT1 | OCLN | OCLN | 0.002401 | 0 | OCLN_OCLN | OCLN | Cell-Cell Contact | KEGG: hsa04514 |
| AT2 | AT1 | OCLN | OCLN | 0.004042 | 0 | OCLN_OCLN | OCLN | Cell-Cell Contact | KEGG: hsa04514 |
| Cil | AT1 | OCLN | OCLN | 0.002193 | 0 | OCLN_OCLN | OCLN | Cell-Cell Contact | KEGG: hsa04514 |
| AT1 | AT2 | OCLN | OCLN | 0.004042 | 0 | OCLN_OCLN | OCLN | Cell-Cell Contact | KEGG: hsa04514 |
| AT2 | AT2 | OCLN | OCLN | 0.006797 | 0 | OCLN_OCLN | OCLN | Cell-Cell Contact | KEGG: hsa04514 |
| Cil | AT2 | OCLN | OCLN | 0.003693 | 0 | OCLN_OCLN | OCLN | Cell-Cell Contact | KEGG: hsa04514 |
| Club | AT2 | OCLN | OCLN | 0.003207 | 0 | OCLN_OCLN | OCLN | Cell-Cell Contact | KEGG: hsa04514 |
| AT1 | Cil | OCLN | OCLN | 0.002193 | 0 | OCLN_OCLN | OCLN | Cell-Cell Contact | KEGG: hsa04514 |
| AT2 | Cil | OCLN | OCLN | 0.003693 | 0 | OCLN_OCLN | OCLN | Cell-Cell Contact | KEGG: hsa04514 |
| AT2 | Club | OCLN | OCLN | 0.003207 | 0 | OCLN_OCLN | OCLN | Cell-Cell Contact | KEGG: hsa04514 |
| AT2 | AT2 | PTPRM | PTPRM | 0.0006 | 0 | PTPRM_PTPRM | PTPRM | Cell-Cell Contact | KEGG: hsa04514 |
| AT2 | Cil | SEMA4A | PLXNB1 | 0.003069 | 0 | SEMA4A_PLXNB1 | SEMA4 | Cell-Cell Contact | PMID: 27533782 |
| AT2 | AT1 | SEMA4A | PLXNB2 | 0.001863 | 0 | SEMA4A_PLXNB2 | SEMA4 | Cell-Cell Contact | PMID: 27533782 |
| AT2 | AT2 | SEMA4A | PLXNB2 | 0.001706 | 0 | SEMA4A_PLXNB2 | SEMA4 | Cell-Cell Contact | PMID: 27533782 |
| AT2 | Cil | SEMA4A | PLXNB2 | 0.005855 | 0 | SEMA4A_PLXNB2 | SEMA4 | Cell-Cell Contact | PMID: 27533782 |
| AT2 | Club | SEMA4A | PLXNB2 | 0.003105 | 0 | SEMA4A_PLXNB2 | SEMA4 | Cell-Cell Contact | PMID: 27533782 |
