# Supplementary material for: Integrative analysis of bulk and single-cell RNA sequencing reveals the gene expression profile and the critical signaling pathways of type II CPAM
Source: Cell Biosci. 2024 Jul 18;14:94. doi: 10.1186/s13578-024-01276-8 (PMC11264590; doi:10.1186/s13578-024-01276-8)
Supplement: Supplementary file 12 — Supplementary Material 12: Supplemental Table 6 KEGG analysis of differentially expressed genes. [file 13578_2024_1276_MOESM12_ESM.docx]

**Supplemental Table 12 Ligands and receptors enriched in PI3K-AKT signaling pathway and epithelial to mesenchymal transition**

| **Pathway** | **Ligand** | **Receptor** |
| --- | --- | --- |
| PI3K-Akt signaling pathway | LAMA5;LAMB3;COL4A2;LAMB2;COL4A1;COL4A4;LAMA3;COL4A3;FN1;LAMC2;VEGFA | INSR;KDR;EGFR |
| Epithelial to mesenchymal transition | COL4A2;COL4A1;COL4A4;COL4A3;FN1 | TGFBR2 |
