# Supplementary material for: Integrative analysis of bulk and single-cell RNA sequencing reveals the gene expression profile and the critical signaling pathways of type II CPAM
Source: Cell Biosci. 2024 Jul 18;14:94. doi: 10.1186/s13578-024-01276-8 (PMC11264590; doi:10.1186/s13578-024-01276-8)
Supplement: Supplementary file 13 — Supplementary Material 13: Supplemental Table 7 Gene list for the top 10 modules performed by iWGCNA. [file 13578_2024_1276_MOESM13_ESM.docx]

**Supplemental Table 1 Clinical characteristics of subjects**

| **Sample** | **Age(m)** | **Sex** | **Duration(m)** | **Position** | **Group** | **Application** |
| --- | --- | --- | --- | --- | --- | --- |
| Subject1 | 7 | Female | 12 | Right lower lobe | case & control | RNA-seq |
| Subject2 | 7 | Female | 12 | Right lower lobe | case & control | RNA-seq |
| Subject3 | 16 | Female | 12 | Right middle and upper lobe | case & control | RNA-seq |
| Subject4 | 9 | Male | 12 | Right lower lobe | case & control | RNA-seq |
| Subject5 | 57 | Female | 60 | Left lower lobe | case & control | RNA-seq |
| Subject6 | 6 | Female | 12 | Right lower lobe | case & control | RNA-seq |
| Subject7 | 7 | Male | 12 | Right lower lobe | control | RNA-seq |
| Subject8 | 7 | Male | 7 | Right lower lobe | control | RNA-seq |
| Subject9 | 26 | Female | 12 | Left upper and lower lobe | control | RNA-seq |
| Subject10 | 5 | Male | 9 | Right lower lobe | control | RNA-seq |
| Subject11 | 7 | Male | 6 | Left lower lobe | control | RNA-seq |
| Subject12 | 49 | Male | 1 | Right lower lobe | control | RNA-seq |
| Subject13 | 13 | Male | 18 | Right lower lobe | control | RNA-seq |
| Subject14 | 11 | Female | 12 | Right lower lobe | control | RNA-seq |
| Subject15 | 44 | Female | 4 | Right lower lobe | control | RNA-seq |
| Subject16 | 18 | Female | 23 | Left lower lobe | case & control | qPCR |
| Subject17 | 1 | Male | 6 | Right lower lobe | case & control | qPCR |
| Subject18 | 65 | Male | 65 | Left lower lobe | case & control | qPCR |
| Subject19 | 63 | Male | 63 | Left lower lobe | case & control | qPCR |
| Subject20 | 72 | Male | 25 | Left upper lobe | case & control | qPCR |
| Subject21 | 65 | Male | 60 | Left upper lobe | case & control | qPCR |
