# Supplementary material for: Integrative analysis of bulk and single-cell RNA sequencing reveals the gene expression profile and the critical signaling pathways of type II CPAM
Source: Cell Biosci. 2024 Jul 18;14:94. doi: 10.1186/s13578-024-01276-8 (PMC11264590; doi:10.1186/s13578-024-01276-8)
Supplement: Supplementary file 14 — Supplementary Material 14: Supplemental Table 8 Overlapped genes between epithelial cell marker genes and DEGs. [file 13578_2024_1276_MOESM14_ESM.docx]

**Supplemental Table 2 Overlapped genes between DEGs related to CPAM and DEGs related to sex.**

| **Gene symbol** |
| --- |
| C11orf88 |
| CFAP77 |
| FAM81B |
| FBXO15 |
| CATSPERD |
| C9orf24 |
| PLPP2 |
| ROPN1L |
| CTXN1 |
| C16orf71 |
| AL449403.3 |
| FAM166C |
| CACNG6 |
| AQP5 |
| GPC5-AS1 |
| DEGS2 |
| CBLC |
| SMIM6 |
| AL590822.3 |
| PLCXD3 |
| MAFG-DT |
| SCGB3A2 |
| SLC16A12 |
| AC069366.1 |
| AL391987.2 |
| TMEM52 |
| MUC12-AS1 |
| LTK |
| PCDHGB4 |
