# Supplementary material for: Integrative analysis of bulk and single-cell RNA sequencing reveals the gene expression profile and the critical signaling pathways of type II CPAM
Source: Cell Biosci. 2024 Jul 18;14:94. doi: 10.1186/s13578-024-01276-8 (PMC11264590; doi:10.1186/s13578-024-01276-8)
Supplement: Supplementary file 15 — Supplementary Material 15: Supplemental Table 9 Overlapped genes between epithelial cell marker genes and the top10 module genes. [file 13578_2024_1276_MOESM15_ESM.docx]

| **Supplemental Table 3 DEGs between CPAM cases and controls** | | | | | | |
| --- | --- | --- | --- | --- | --- | --- |
| **Gene** | **baseMean** | **log2 Fold**  **Change** | **lfcSE** | **stat** | ***P*-value** | **Adjusted *P*-value** |
| PRDM8 | 282.2713793 | 1.664352646 | 0.18828561 | 8.83951058 | 9.61E-19 | 7.59E-15 |
| CCNO | 355.6982862 | 3.125583804 | 0.351773544 | 8.88521567 | 6.38E-19 | 7.59E-15 |
| WIPF3 | 141.5410292 | 2.451316776 | 0.27767337 | 8.82805859 | 1.07E-18 | 7.59E-15 |
| MMP7 | 844.9486776 | 3.025803684 | 0.337688703 | 8.96033436 | 3.24E-19 | 7.59E-15 |
| SIX1 | 587.6546991 | 3.084765633 | 0.370621425 | 8.32322534 | 8.56E-17 | 4.88E-13 |
| DNAAF4-CCPG1 | 92.03675553 | 26.67714942 | 3.236818031 | 8.24178226 | 1.70E-16 | 8.07E-13 |
| NME5 | 538.0761314 | 2.390487229 | 0.292040104 | 8.1854759 | 2.71E-16 | 1.11E-12 |
| SCGB1A1 | 222248.8625 | 3.396478257 | 0.4175064 | 8.13515256 | 4.11E-16 | 1.47E-12 |
| CLIC6 | 6412.636459 | 2.333531539 | 0.290414246 | 8.03518274 | 9.34E-16 | 2.96E-12 |
| STK33 | 3568.24877 | 2.546142784 | 0.319159999 | 7.97763753 | 1.49E-15 | 4.25E-12 |
| WDR63 | 875.8781839 | 2.706862693 | 0.341366973 | 7.92948031 | 2.20E-15 | 5.71E-12 |
| RIBC1 | 1899.535688 | 2.56209764 | 0.324085207 | 7.90562971 | 2.67E-15 | 6.34E-12 |
| AK8 | 292.686903 | 2.445566453 | 0.315436176 | 7.7529676 | 8.98E-15 | 1.72E-11 |
| CDH19 | 294.3108482 | -2.055497043 | 0.264901919 | -7.7594645 | 8.53E-15 | 1.72E-11 |
| HMGB3 | 2617.9384 | 1.445392666 | 0.18644732 | 7.75228447 | 9.03E-15 | 1.72E-11 |
| CHIT1 | 383.0058798 | 5.043540155 | 0.653721518 | 7.7151203 | 1.21E-14 | 1.72E-11 |
| TRIM17 | 353.3994613 | 1.778284014 | 0.230246221 | 7.72340153 | 1.13E-14 | 1.72E-11 |
| CCDC148 | 240.0651776 | 1.97659012 | 0.255483508 | 7.73666423 | 1.02E-14 | 1.72E-11 |
| CALML4 | 1258.864521 | 2.012826956 | 0.260673006 | 7.72165477 | 1.15E-14 | 1.72E-11 |
| HOXB4 | 1607.089835 | 1.168145424 | 0.15140985 | 7.71512172 | 1.21E-14 | 1.72E-11 |
| ETV5 | 3937.22105 | -1.391836529 | 0.180660656 | -7.7041485 | 1.32E-14 | 1.79E-11 |
| PARD6G-AS1 | 224.6396052 | 1.853734132 | 0.240979763 | 7.69248882 | 1.44E-14 | 1.87E-11 |
| KIAA1324 | 1406.066955 | 2.698507427 | 0.352240725 | 7.6609751 | 1.85E-14 | 2.20E-11 |
| RSPH10B | 1562.560758 | 2.707375326 | 0.353419667 | 7.66051124 | 1.85E-14 | 2.20E-11 |
| CP | 2445.926769 | 3.367961423 | 0.44027441 | 7.64968698 | 2.01E-14 | 2.21E-11 |
| AP002008.4 | 154.2551514 | 3.229792083 | 0.422145177 | 7.65090367 | 2.00E-14 | 2.21E-11 |
| UBXN10 | 2809.056298 | 2.540405244 | 0.33233076 | 7.64420736 | 2.10E-14 | 2.22E-11 |
| EFCAB1 | 3282.122282 | 2.760436048 | 0.36175638 | 7.63064924 | 2.34E-14 | 2.38E-11 |
| LRRC18 | 253.2251605 | 2.31349826 | 0.304205246 | 7.60505708 | 2.85E-14 | 2.79E-11 |
| SPTBN2 | 1059.787583 | 1.925018629 | 0.253253361 | 7.60115728 | 2.93E-14 | 2.79E-11 |
| AC007906.2 | 1855.035243 | 2.658712199 | 0.350474706 | 7.58603162 | 3.30E-14 | 3.03E-11 |
| MIR205HG | 293.2537804 | 3.343736748 | 0.441525088 | 7.57315233 | 3.64E-14 | 3.13E-11 |
| TP63 | 222.6677579 | 2.915947793 | 0.385282638 | 7.56833428 | 3.78E-14 | 3.13E-11 |
| TCTE1 | 774.193224 | 2.708611235 | 0.357980213 | 7.56637136 | 3.84E-14 | 3.13E-11 |
| TMEM189-UBE2V1 | 16.12532218 | 24.53124191 | 3.237612311 | 7.57695473 | 3.54E-14 | 3.13E-11 |
| CPEB1 | 116.5044296 | 2.141502478 | 0.283578028 | 7.55172215 | 4.30E-14 | 3.40E-11 |
| C6 | 1494.737401 | 2.544709273 | 0.337865584 | 7.53172089 | 5.01E-14 | 3.86E-11 |
| HES2 | 215.4650023 | 2.253001065 | 0.299372404 | 7.5257473 | 5.24E-14 | 3.93E-11 |
| CA8 | 506.644683 | 1.697201076 | 0.22644946 | 7.49483385 | 6.64E-14 | 4.62E-11 |
| C11orf88 | 1895.408909 | 2.851481396 | 0.380151748 | 7.50090303 | 6.34E-14 | 4.62E-11 |
| CFAP61 | 551.7843501 | 2.635015541 | 0.351553702 | 7.49534289 | 6.61E-14 | 4.62E-11 |
| TMEM212 | 556.2818698 | 2.776210708 | 0.37204665 | 7.46199625 | 8.52E-14 | 5.79E-11 |
| DZIP3 | 2446.546742 | 1.676144954 | 0.225288523 | 7.43999264 | 1.01E-13 | 6.68E-11 |
| TP73 | 1251.977453 | 2.64116239 | 0.355503571 | 7.42935544 | 1.09E-13 | 7.07E-11 |
| IQCG | 4181.213842 | 2.299184934 | 0.309947151 | 7.41799023 | 1.19E-13 | 7.37E-11 |
| CWH43 | 366.5378202 | 3.14617207 | 0.423988717 | 7.42041461 | 1.17E-13 | 7.37E-11 |
| TEKT2 | 4933.864598 | 2.719778127 | 0.367600136 | 7.39874081 | 1.37E-13 | 8.17E-11 |
| HID1-AS1 | 89.33627451 | -1.745727207 | 0.235898861 | -7.4003206 | 1.36E-13 | 8.17E-11 |
| SPA17 | 1835.616609 | 2.0311415 | 0.275124958 | 7.38261449 | 1.55E-13 | 8.85E-11 |
| AL645924.1 | 124.2018249 | 2.82648662 | 0.383186898 | 7.37626113 | 1.63E-13 | 8.93E-11 |
| CCDC60 | 602.4655905 | 2.74162803 | 0.371623321 | 7.37743805 | 1.61E-13 | 8.93E-11 |
| USP2-AS1 | 238.1210732 | 2.266323472 | 0.307761426 | 7.36389709 | 1.79E-13 | 9.61E-11 |
| ANKFN1 | 241.8394031 | 2.610634605 | 0.354828908 | 7.35744622 | 1.87E-13 | 9.90E-11 |
| OSBPL6 | 2224.891807 | 1.794591118 | 0.244158772 | 7.3500989 | 1.98E-13 | 1.03E-10 |
| DNAH6 | 1212.82387 | 2.438194347 | 0.331888759 | 7.34642039 | 2.04E-13 | 1.04E-10 |
| NEK11 | 2041.103547 | 2.112208226 | 0.287979444 | 7.33457984 | 2.22E-13 | 1.09E-10 |
| BARX2 | 200.8753216 | 2.920819714 | 0.398122318 | 7.33648826 | 2.19E-13 | 1.09E-10 |
| AL121956.6 | 1467.098953 | 2.625909292 | 0.359207055 | 7.31029431 | 2.67E-13 | 1.29E-10 |
| RSPH14 | 209.6055798 | 2.813459355 | 0.384991449 | 7.30784895 | 2.71E-13 | 1.29E-10 |
| CFAP77 | 1082.946976 | 2.809659553 | 0.384804387 | 7.30152682 | 2.85E-13 | 1.31E-10 |
| AC027237.5 | 378.1835706 | 2.170625179 | 0.29736133 | 7.29962158 | 2.89E-13 | 1.31E-10 |
| ZNF473 | 2904.935305 | 2.353831527 | 0.322383198 | 7.3013468 | 2.85E-13 | 1.31E-10 |
| SIX4 | 286.6223409 | 2.639508774 | 0.362130857 | 7.28882591 | 3.13E-13 | 1.39E-10 |
| CASC1 | 745.123823 | 2.476132745 | 0.339999062 | 7.28276346 | 3.27E-13 | 1.44E-10 |
| CDH3 | 541.1976818 | 2.395819971 | 0.329156061 | 7.27867493 | 3.37E-13 | 1.46E-10 |
| H2AW | 213.8699608 | 1.24463077 | 0.171236509 | 7.26848954 | 3.64E-13 | 1.50E-10 |
| ZNF474 | 370.5255918 | 3.03662902 | 0.417884489 | 7.26667081 | 3.68E-13 | 1.50E-10 |
| TMEM130 | 389.6353957 | 2.707487529 | 0.372609725 | 7.26628252 | 3.70E-13 | 1.50E-10 |
| WDR86-AS1 | 1552.009203 | 2.701519633 | 0.371807789 | 7.26590382 | 3.71E-13 | 1.50E-10 |
| DRC3 | 4869.520038 | 2.297473514 | 0.316261916 | 7.26446466 | 3.75E-13 | 1.50E-10 |
| ARMC4 | 1710.126079 | 2.70847273 | 0.37315887 | 7.25822953 | 3.92E-13 | 1.55E-10 |
| SPAG16 | 1152.16057 | 1.537682691 | 0.212060203 | 7.25116109 | 4.13E-13 | 1.59E-10 |
| GMNN | 968.9982137 | 1.362426085 | 0.18787088 | 7.25192794 | 4.11E-13 | 1.59E-10 |
| USP2 | 818.3849093 | 2.22761926 | 0.307365963 | 7.24744938 | 4.25E-13 | 1.62E-10 |
| ADGB | 1423.844573 | 2.77693676 | 0.383385179 | 7.24320322 | 4.38E-13 | 1.64E-10 |
| AP003064.2 | 191.3646459 | 3.706643888 | 0.512610634 | 7.23091493 | 4.80E-13 | 1.75E-10 |
| DNAL1 | 1203.853017 | 1.382043116 | 0.191148517 | 7.23020578 | 4.82E-13 | 1.75E-10 |
| LINC02345 | 529.7491069 | 2.495557894 | 0.345180534 | 7.22971793 | 4.84E-13 | 1.75E-10 |
| FOXJ1 | 8189.316337 | 2.665215731 | 0.368911865 | 7.22453242 | 5.03E-13 | 1.79E-10 |
| CFAP126 | 1767.75607 | 2.477163725 | 0.342970194 | 7.22267931 | 5.10E-13 | 1.79E-10 |
| CCDC65 | 1633.328876 | 2.420241738 | 0.336036998 | 7.20230734 | 5.92E-13 | 2.06E-10 |
| C2orf50 | 785.943948 | 2.559980275 | 0.355647859 | 7.19807587 | 6.11E-13 | 2.10E-10 |
| CFAP53 | 3705.957944 | 2.623411328 | 0.364882961 | 7.18973372 | 6.49E-13 | 2.20E-10 |
| NGEF | 198.1493891 | 2.336228754 | 0.325462945 | 7.17817125 | 7.07E-13 | 2.32E-10 |
| EYA1 | 393.4968586 | 2.752621625 | 0.38348219 | 7.17796471 | 7.08E-13 | 2.32E-10 |
| DPY19L2P1 | 70.79604124 | 2.576160541 | 0.359162381 | 7.17269033 | 7.35E-13 | 2.38E-10 |
| ENKUR | 1414.110027 | 2.760529086 | 0.38500212 | 7.17016595 | 7.49E-13 | 2.40E-10 |
| HOXC4 | 278.6701081 | 2.487757495 | 0.347631407 | 7.15630823 | 8.29E-13 | 2.63E-10 |
| GAS2L2 | 1653.114116 | 2.714777754 | 0.379670308 | 7.15035571 | 8.66E-13 | 2.71E-10 |
| SNTN | 2633.831518 | 2.746977822 | 0.384354923 | 7.14698228 | 8.87E-13 | 2.75E-10 |
| LEKR1 | 128.5803054 | 2.138344877 | 0.300239966 | 7.12211937 | 1.06E-12 | 3.26E-10 |
| INHBB | 1129.733912 | 2.067489632 | 0.290763687 | 7.11054966 | 1.16E-12 | 3.46E-10 |
| TUBA3FP | 211.2639115 | 1.981080422 | 0.278650357 | 7.10955638 | 1.16E-12 | 3.46E-10 |
| CETN2 | 6398.154605 | 1.848882136 | 0.259966565 | 7.11199973 | 1.14E-12 | 3.46E-10 |
| RIBC2 | 882.9707879 | 2.715885739 | 0.382247135 | 7.10505192 | 1.20E-12 | 3.54E-10 |
| SPP1 | 356.6563248 | 3.626110902 | 0.511398761 | 7.09057428 | 1.34E-12 | 3.89E-10 |
| CCDC157 | 1800.60563 | 2.226830724 | 0.314313599 | 7.08474189 | 1.39E-12 | 4.01E-10 |
| EFHC2 | 432.7439498 | 2.500086368 | 0.353158201 | 7.07922501 | 1.45E-12 | 4.13E-10 |
| EFCAB6 | 702.9628522 | 2.466197479 | 0.348497313 | 7.07666139 | 1.48E-12 | 4.17E-10 |
| MYB | 1170.514109 | 2.184799403 | 0.308925603 | 7.07225099 | 1.52E-12 | 4.26E-10 |
| HACD1 | 533.1306309 | -1.025290157 | 0.145084352 | -7.0668555 | 1.58E-12 | 4.39E-10 |
| STMND1 | 1729.531003 | 2.782798947 | 0.394315669 | 7.05728725 | 1.70E-12 | 4.66E-10 |
| FAM81B | 1664.128983 | 2.717431571 | 0.385206021 | 7.05448882 | 1.73E-12 | 4.71E-10 |
| WDR78 | 1748.830697 | 2.390522629 | 0.338929069 | 7.05316495 | 1.75E-12 | 4.71E-10 |
| MDH1B | 800.7391216 | 2.645817386 | 0.37530828 | 7.0497176 | 1.79E-12 | 4.74E-10 |
| FAM104B | 592.7569814 | 1.167870455 | 0.165665171 | 7.04958348 | 1.79E-12 | 4.74E-10 |
| STOX1 | 593.7049755 | 2.509611139 | 0.356226819 | 7.0449809 | 1.85E-12 | 4.85E-10 |
| AL357093.2 | 447.2503593 | 2.521847959 | 0.358049591 | 7.04329238 | 1.88E-12 | 4.86E-10 |
| C21orf58 | 3029.311207 | 2.50396394 | 0.355561376 | 7.04228329 | 1.89E-12 | 4.86E-10 |
| AP004608.1 | 750.8200339 | 2.736240868 | 0.388677832 | 7.0398686 | 1.92E-12 | 4.90E-10 |
| GSTA3 | 500.1506293 | 2.780674256 | 0.395132073 | 7.03732865 | 1.96E-12 | 4.90E-10 |
| CAPS | 14158.30401 | 2.435058126 | 0.346081639 | 7.03608008 | 1.98E-12 | 4.90E-10 |
| ARX | 372.6611467 | 2.807678516 | 0.398987459 | 7.03700945 | 1.96E-12 | 4.90E-10 |
| AC245297.1 | 401.8566719 | 1.772475882 | 0.252174208 | 7.02877544 | 2.08E-12 | 5.12E-10 |
| DRC1 | 5441.233907 | 2.726582586 | 0.38864651 | 7.0155849 | 2.29E-12 | 5.54E-10 |
| C20orf85 | 6655.671813 | 2.707795884 | 0.385960502 | 7.01573314 | 2.29E-12 | 5.54E-10 |
| WDR93 | 538.5363731 | 2.643591757 | 0.376952141 | 7.01307001 | 2.33E-12 | 5.54E-10 |
| SRCIN1 | 711.5190214 | 2.366732622 | 0.337422548 | 7.01415077 | 2.31E-12 | 5.54E-10 |
| ST6GALNAC1 | 1297.25783 | 2.390445664 | 0.341286673 | 7.00421626 | 2.48E-12 | 5.85E-10 |
| DAW1 | 483.8123273 | 2.545244392 | 0.363698153 | 6.99823293 | 2.59E-12 | 6.06E-10 |
| NRAD1 | 121.4588915 | 2.552956232 | 0.36508792 | 6.99271626 | 2.70E-12 | 6.25E-10 |
| ZBBX | 1669.846282 | 2.627216324 | 0.376967641 | 6.96934177 | 3.18E-12 | 7.32E-10 |
| CCDC81 | 1916.150648 | 2.412105037 | 0.346960658 | 6.95209956 | 3.60E-12 | 8.21E-10 |
| VWA3B | 2068.169528 | 2.499609388 | 0.359680534 | 6.94952647 | 3.67E-12 | 8.30E-10 |
| GLB1L2 | 992.57388 | 2.214618207 | 0.318807875 | 6.94656054 | 3.74E-12 | 8.41E-10 |
| RSPH1 | 13715.00395 | 2.690525909 | 0.38782275 | 6.93751439 | 3.99E-12 | 8.89E-10 |
| PACRG | 407.7436315 | 2.53866827 | 0.366307531 | 6.93042882 | 4.20E-12 | 9.14E-10 |
| LRRC23 | 4442.650074 | 2.247900899 | 0.324329415 | 6.93091898 | 4.18E-12 | 9.14E-10 |
| MNS1 | 2338.485293 | 2.204193276 | 0.318035425 | 6.93065332 | 4.19E-12 | 9.14E-10 |
| KCNE1B | 538.5767268 | 2.426490981 | 0.350259718 | 6.92769068 | 4.28E-12 | 9.24E-10 |
| PFN2 | 1993.907725 | 1.463267414 | 0.211269578 | 6.92606775 | 4.33E-12 | 9.28E-10 |
| GRIN3B | 1358.715116 | 2.351545869 | 0.339771011 | 6.92097263 | 4.49E-12 | 9.55E-10 |
| DNAI2 | 2381.376716 | 2.669669768 | 0.386146921 | 6.91361144 | 4.72E-12 | 9.98E-10 |
| AC067968.1 | 50.47997117 | -22.81593359 | 3.300618148 | -6.9126244 | 4.76E-12 | 9.98E-10 |
| LRRC6 | 2130.302696 | 2.017343528 | 0.292071175 | 6.90702713 | 4.95E-12 | 1.03E-09 |
| CFAP57 | 3473.218344 | 2.56691393 | 0.372197541 | 6.89664398 | 5.32E-12 | 1.10E-09 |
| FHOD3 | 874.0272273 | 2.319132661 | 0.336325643 | 6.8954976 | 5.37E-12 | 1.10E-09 |
| C22orf23 | 473.9364223 | 1.894685865 | 0.274872309 | 6.89296739 | 5.46E-12 | 1.11E-09 |
| FBXO15 | 387.4162388 | 2.591827453 | 0.376090335 | 6.89150242 | 5.52E-12 | 1.12E-09 |
| RSPH9 | 1290.570022 | 2.421719115 | 0.35197049 | 6.88046068 | 5.97E-12 | 1.18E-09 |
| FAM229B | 548.5163468 | 1.749427215 | 0.254291527 | 6.87961269 | 6.00E-12 | 1.18E-09 |
| TRIM29 | 929.5925915 | 2.348022044 | 0.341318096 | 6.87927792 | 6.02E-12 | 1.18E-09 |
| CCDC151 | 2399.48772 | 2.621490756 | 0.38092786 | 6.88185621 | 5.91E-12 | 1.18E-09 |
| ZFHX2 | 1112.220492 | 2.095931951 | 0.304928651 | 6.87351596 | 6.26E-12 | 1.22E-09 |
| CNGA4 | 686.7595802 | 2.654080866 | 0.386803173 | 6.86157987 | 6.81E-12 | 1.32E-09 |
| CCDC74B | 1840.377464 | 2.483100462 | 0.362137299 | 6.85679291 | 7.04E-12 | 1.36E-09 |
| IQANK1 | 452.6833654 | 2.07761741 | 0.3030565 | 6.8555448 | 7.10E-12 | 1.36E-09 |
| MAATS1 | 3600.309453 | 2.173647901 | 0.317425607 | 6.8477396 | 7.50E-12 | 1.43E-09 |
| CCDC173 | 1383.431185 | 2.414670861 | 0.352694581 | 6.84635089 | 7.58E-12 | 1.43E-09 |
| MORN2 | 1028.432834 | 1.887085681 | 0.275754323 | 6.84335846 | 7.74E-12 | 1.45E-09 |
| C11orf16 | 1902.042207 | 2.693591963 | 0.393895723 | 6.83833767 | 8.01E-12 | 1.49E-09 |
| HHLA2 | 839.303744 | 2.556035026 | 0.374080293 | 6.83285133 | 8.32E-12 | 1.52E-09 |
| SCUBE3 | 386.0387321 | 1.707555323 | 0.249888042 | 6.83328146 | 8.30E-12 | 1.52E-09 |
| CCDC180 | 3614.594772 | 1.801488634 | 0.263705358 | 6.83144495 | 8.41E-12 | 1.53E-09 |
| LRRC46 | 3262.899391 | 2.609912558 | 0.382208891 | 6.82849776 | 8.58E-12 | 1.55E-09 |
| CCL11 | 154.1105769 | 2.521527188 | 0.369394516 | 6.82610888 | 8.72E-12 | 1.57E-09 |
| IQCH | 194.2807661 | 2.190414103 | 0.321011029 | 6.8234855 | 8.89E-12 | 1.58E-09 |
| COLCA1 | 1748.928482 | 1.777549302 | 0.26058385 | 6.82141008 | 9.02E-12 | 1.60E-09 |
| TUBA4B | 1433.693312 | 2.67592025 | 0.392584096 | 6.8161708 | 9.35E-12 | 1.65E-09 |
| PPIL6 | 1150.358089 | 2.129977247 | 0.312645622 | 6.8127525 | 9.57E-12 | 1.68E-09 |
| TEKT1 | 3337.272877 | 2.717360683 | 0.399039403 | 6.80975528 | 9.78E-12 | 1.70E-09 |
| WNK2 | 523.3032131 | 2.323480327 | 0.341253989 | 6.80865397 | 9.85E-12 | 1.70E-09 |
| CHST9 | 1051.883119 | 2.72953682 | 0.401081744 | 6.80543769 | 1.01E-11 | 1.73E-09 |
| C9orf135 | 451.5685315 | 2.546793617 | 0.374368546 | 6.8029049 | 1.03E-11 | 1.75E-09 |
| NELL2 | 777.8824949 | 2.465664097 | 0.36249193 | 6.80198342 | 1.03E-11 | 1.75E-09 |
| MAPRE3 | 3573.194283 | 1.655074537 | 0.243435417 | 6.79882392 | 1.05E-11 | 1.78E-09 |
| MORN5 | 917.489866 | 2.618483126 | 0.385208273 | 6.7975776 | 1.06E-11 | 1.79E-09 |
| SRGAP3-AS2 | 1998.2513 | 2.839416612 | 0.417796684 | 6.79616838 | 1.07E-11 | 1.79E-09 |
| FBXO36 | 271.3814934 | 1.942519543 | 0.28589238 | 6.79458312 | 1.09E-11 | 1.80E-09 |
| SPATA17 | 942.7306086 | 2.449462071 | 0.360589357 | 6.79294058 | 1.10E-11 | 1.81E-09 |
| FAM238C | 235.6535232 | 2.221305684 | 0.327109003 | 6.7907201 | 1.12E-11 | 1.83E-09 |
| RIIAD1 | 266.1866854 | 2.340564975 | 0.344836922 | 6.78745466 | 1.14E-11 | 1.85E-09 |
| ERICH6-AS1 | 598.6299334 | 2.226121539 | 0.327934245 | 6.78831679 | 1.13E-11 | 1.85E-09 |
| RP1 | 2003.081511 | 2.27363457 | 0.335073715 | 6.78547576 | 1.16E-11 | 1.85E-09 |
| TMEM231 | 3074.453463 | 2.328238696 | 0.343160887 | 6.78468551 | 1.16E-11 | 1.85E-09 |
| CCDC114 | 8688.204797 | 2.371699753 | 0.349587454 | 6.78428166 | 1.17E-11 | 1.85E-09 |
| AC010624.1 | 113.3891868 | 2.58640228 | 0.381152799 | 6.78573602 | 1.15E-11 | 1.85E-09 |
| DNAI1 | 3480.980723 | 2.564267463 | 0.378036306 | 6.78312486 | 1.18E-11 | 1.85E-09 |
| FAM166B | 1600.13868 | 2.551577579 | 0.376419563 | 6.77854668 | 1.21E-11 | 1.90E-09 |
| AC096637.3 | 344.3409892 | 2.531057424 | 0.37353902 | 6.77588495 | 1.24E-11 | 1.93E-09 |
| IL5RA | 903.2613963 | 2.274489349 | 0.335841849 | 6.77250127 | 1.27E-11 | 1.95E-09 |
| CFAP43 | 5246.882129 | 2.578599574 | 0.380721682 | 6.77292546 | 1.26E-11 | 1.95E-09 |
| CFAP52 | 2219.81767 | 2.648895367 | 0.391384835 | 6.76800717 | 1.31E-11 | 2.00E-09 |
| PRR7 | 255.8552338 | 2.074989539 | 0.306716448 | 6.76517204 | 1.33E-11 | 2.02E-09 |
| ARMC3 | 1344.554254 | 2.686782992 | 0.397116383 | 6.76573193 | 1.33E-11 | 2.02E-09 |
| CCDC146 | 3174.164624 | 2.027222519 | 0.299691603 | 6.76436209 | 1.34E-11 | 2.02E-09 |
| KIF24 | 413.1163559 | 2.18552386 | 0.32321321 | 6.76186428 | 1.36E-11 | 2.03E-09 |
| AGBL2 | 817.9384246 | 2.439855514 | 0.360791823 | 6.76250224 | 1.36E-11 | 2.03E-09 |
| PCSK1N | 690.6153112 | 2.304780506 | 0.340866929 | 6.76152571 | 1.37E-11 | 2.03E-09 |
| MAP6 | 1979.823501 | 1.627488182 | 0.240826893 | 6.75791713 | 1.40E-11 | 2.06E-09 |
| CCDC89 | 517.021915 | 2.123459348 | 0.314226699 | 6.75773051 | 1.40E-11 | 2.06E-09 |
| CCDC138 | 438.0440979 | 1.771134414 | 0.26214272 | 6.75637459 | 1.41E-11 | 2.07E-09 |
| C8orf34 | 372.1475221 | 2.216566185 | 0.328271639 | 6.75223175 | 1.46E-11 | 2.12E-09 |
| DRC7 | 2417.155876 | 2.602029863 | 0.385526713 | 6.74928552 | 1.49E-11 | 2.15E-09 |
| AKAP3 | 128.5639525 | 1.467648251 | 0.217527981 | 6.74694006 | 1.51E-11 | 2.18E-09 |
| CCDC189 | 1163.894747 | 2.538716122 | 0.376514954 | 6.74267009 | 1.56E-11 | 2.23E-09 |
| FOXA1 | 1408.294649 | 1.828765765 | 0.27134603 | 6.73960759 | 1.59E-11 | 2.26E-09 |
| HOXB-AS1 | 271.6014277 | 1.336932928 | 0.198534239 | 6.73401692 | 1.65E-11 | 2.34E-09 |
| BMPR1B | 209.7615983 | 2.714389226 | 0.403350051 | 6.72961171 | 1.70E-11 | 2.40E-09 |
| MS4A8 | 2171.16299 | 2.545919586 | 0.378551138 | 6.72543108 | 1.75E-11 | 2.46E-09 |
| AC011487.1 | 77.01590733 | -2.57873705 | 0.383528606 | -6.723715 | 1.77E-11 | 2.48E-09 |
| LPL | 6314.140913 | -1.508934628 | 0.224544195 | -6.7199895 | 1.82E-11 | 2.50E-09 |
| DNAAF3 | 2993.947819 | 2.730257098 | 0.406260328 | 6.720462 | 1.81E-11 | 2.50E-09 |
| EFCAB12 | 1887.120604 | 2.520534621 | 0.375495884 | 6.71254926 | 1.91E-11 | 2.61E-09 |
| TTC6 | 295.447502 | 2.221989172 | 0.330992592 | 6.71310847 | 1.91E-11 | 2.61E-09 |
| CEACAM5 | 798.5967318 | 5.137842677 | 0.765544871 | 6.71135406 | 1.93E-11 | 2.62E-09 |
| RAB36 | 1905.364092 | 2.237870361 | 0.333585684 | 6.70853238 | 1.97E-11 | 2.66E-09 |
| CCDC17 | 7283.397873 | 2.484380767 | 0.37050846 | 6.70532804 | 2.01E-11 | 2.70E-09 |
| LPAR3 | 814.5879206 | 1.760196449 | 0.262569288 | 6.7037408 | 2.03E-11 | 2.72E-09 |
| AK7 | 2078.006342 | 2.437001689 | 0.363620504 | 6.70204694 | 2.06E-11 | 2.74E-09 |
| IQCA1 | 2187.791273 | 1.679515075 | 0.250789675 | 6.69690677 | 2.13E-11 | 2.82E-09 |
| LRRC34 | 430.4244483 | 1.814226421 | 0.270953042 | 6.69572266 | 2.15E-11 | 2.83E-09 |
| TEKT3 | 429.767185 | 2.690417329 | 0.401845958 | 6.69514593 | 2.15E-11 | 2.83E-09 |
| SPAG8 | 1722.53082 | 2.447929104 | 0.366016994 | 6.68802036 | 2.26E-11 | 2.96E-09 |
| FCGBP | 1087.027825 | 2.492902066 | 0.37288677 | 6.68541302 | 2.30E-11 | 3.00E-09 |
| EFHB | 787.0855416 | 2.367968605 | 0.354395992 | 6.68170256 | 2.36E-11 | 3.06E-09 |
| GON7 | 428.9552073 | 1.470133024 | 0.220101922 | 6.67932842 | 2.40E-11 | 3.10E-09 |
| CYP4X1 | 735.0582979 | 1.569409815 | 0.235030584 | 6.67747059 | 2.43E-11 | 3.12E-09 |
| KCNRG | 214.5688667 | 2.949623415 | 0.441942024 | 6.67423158 | 2.49E-11 | 3.16E-09 |
| CATSPERD | 284.7454582 | 2.811240584 | 0.421190293 | 6.67451418 | 2.48E-11 | 3.16E-09 |
| PROM2 | 1057.255099 | 1.292286684 | 0.19379556 | 6.66829873 | 2.59E-11 | 3.28E-09 |
| KCNH3 | 627.3765545 | 2.312667793 | 0.346956681 | 6.66558081 | 2.64E-11 | 3.33E-09 |
| TNFAIP8L1 | 2872.726671 | 1.575599647 | 0.236402666 | 6.66489797 | 2.65E-11 | 3.33E-09 |
| AC089999.1 | 51.49938563 | 2.843305464 | 0.42699507 | 6.65887188 | 2.76E-11 | 3.45E-09 |
| LINC01765 | 276.1377687 | 2.714191992 | 0.407724174 | 6.65693173 | 2.80E-11 | 3.47E-09 |
| CCDC153 | 1198.934874 | 2.20737719 | 0.33159183 | 6.65691067 | 2.80E-11 | 3.47E-09 |
| CCDC78 | 4902.397193 | 2.550719186 | 0.383307427 | 6.65449977 | 2.84E-11 | 3.51E-09 |
| MYRF | 5937.187168 | -1.125288583 | 0.169158593 | -6.6522697 | 2.89E-11 | 3.53E-09 |
| FAM92B | 2049.630452 | 2.658123253 | 0.400067805 | 6.64418187 | 3.05E-11 | 3.72E-09 |
| HCAR1 | 90.04768478 | 2.08796213 | 0.314398154 | 6.64113991 | 3.11E-11 | 3.78E-09 |
| KCTD1 | 898.7522944 | 1.615027323 | 0.243369828 | 6.63610331 | 3.22E-11 | 3.89E-09 |
| AC074212.1 | 663.2918033 | 1.618703139 | 0.244080999 | 6.63182774 | 3.32E-11 | 3.99E-09 |
| TOGARAM2 | 2874.275831 | 2.266556331 | 0.34190662 | 6.62916774 | 3.38E-11 | 4.05E-09 |
| SPACA9 | 1194.128801 | 2.062773269 | 0.31133096 | 6.62566057 | 3.46E-11 | 4.11E-09 |
| ZNF331 | 4220.018165 | -1.416767681 | 0.213836802 | -6.6254624 | 3.46E-11 | 4.11E-09 |
| NSUN7 | 1508.822338 | 2.200704633 | 0.332254721 | 6.62354662 | 3.51E-11 | 4.14E-09 |
| ARHGAP39 | 1958.674543 | 1.859216223 | 0.280708761 | 6.62329246 | 3.51E-11 | 4.14E-09 |
| CCDC113 | 2638.059502 | 2.450491668 | 0.370088314 | 6.62137003 | 3.56E-11 | 4.16E-09 |
| AC010624.3 | 477.6724097 | 2.39965083 | 0.362402456 | 6.62150819 | 3.56E-11 | 4.16E-09 |
| CFAP45 | 7817.72209 | 2.60606388 | 0.394088012 | 6.61289813 | 3.77E-11 | 4.39E-09 |
| CDHR4 | 2836.059623 | 2.632958651 | 0.39831611 | 6.61022385 | 3.84E-11 | 4.42E-09 |
| LRRC10B | 1426.300102 | 2.615728519 | 0.395694263 | 6.61047876 | 3.83E-11 | 4.42E-09 |
| AQP6 | 42.06639394 | 2.324749927 | 0.351701914 | 6.61000078 | 3.84E-11 | 4.42E-09 |
| REEP6 | 331.9205778 | 2.129568017 | 0.322198436 | 6.60949211 | 3.86E-11 | 4.42E-09 |
| RHPN1-AS1 | 47.50932702 | 2.17978349 | 0.329872578 | 6.60795603 | 3.90E-11 | 4.45E-09 |
| KIF19 | 2055.828142 | 2.469643566 | 0.373906693 | 6.60497287 | 3.98E-11 | 4.52E-09 |
| EPB41L4B | 1006.235995 | 1.337774187 | 0.202621833 | 6.60232002 | 4.05E-11 | 4.58E-09 |
| CD164L2 | 1341.288807 | 2.475969639 | 0.375148004 | 6.59998085 | 4.11E-11 | 4.64E-09 |
| KLK11 | 2328.295078 | 1.682310675 | 0.255061261 | 6.59571222 | 4.23E-11 | 4.75E-09 |
| CFAP221 | 1931.145294 | 1.99308226 | 0.302212937 | 6.5949601 | 4.25E-11 | 4.76E-09 |
| TESMIN | 137.431887 | 2.141560085 | 0.324946927 | 6.59049188 | 4.38E-11 | 4.88E-09 |
| E2F8 | 116.2939083 | 1.99646283 | 0.303155426 | 6.58560811 | 4.53E-11 | 5.03E-09 |
| TPTE2P2 | 18.8289367 | 2.552846221 | 0.387715629 | 6.58432632 | 4.57E-11 | 5.05E-09 |
| WNT4 | 309.8234863 | 1.420740883 | 0.215849277 | 6.58209702 | 4.64E-11 | 5.11E-09 |
| RSPH4A | 2683.179041 | 2.58566464 | 0.393059919 | 6.57829638 | 4.76E-11 | 5.22E-09 |
| CFAP58 | 970.430986 | 2.301594672 | 0.35005961 | 6.57486498 | 4.87E-11 | 5.32E-09 |
| CFAP73 | 3526.938492 | 2.509492232 | 0.382269941 | 6.56471243 | 5.21E-11 | 5.67E-09 |
| STOML3 | 543.0263588 | 2.80962625 | 0.428018181 | 6.56426847 | 5.23E-11 | 5.67E-09 |
| TTC25 | 4029.498402 | 2.426140941 | 0.369679741 | 6.56281822 | 5.28E-11 | 5.70E-09 |
| PIFO | 3223.792623 | 2.360357396 | 0.359758431 | 6.56095088 | 5.35E-11 | 5.75E-09 |
| LINC01571 | 73.12322996 | 2.601491251 | 0.396943847 | 6.55380168 | 5.61E-11 | 6.01E-09 |
| TTC29 | 576.4709207 | 2.492878249 | 0.380463494 | 6.55221405 | 5.67E-11 | 6.06E-09 |
| TTC26 | 1153.702718 | 1.835131261 | 0.280371522 | 6.54535542 | 5.94E-11 | 6.32E-09 |
| SPEF1 | 2075.396944 | 2.610092108 | 0.398953612 | 6.54234484 | 6.06E-11 | 6.42E-09 |
| FABP6 | 2505.222049 | 2.494232923 | 0.381681677 | 6.53485109 | 6.37E-11 | 6.70E-09 |
| SAXO2 | 1676.350966 | 2.473358556 | 0.378461301 | 6.5353011 | 6.35E-11 | 6.70E-09 |
| KLHDC7A | 259.777326 | 2.075608258 | 0.317959222 | 6.52790709 | 6.67E-11 | 6.94E-09 |
| C4orf47 | 870.3808993 | 2.533355034 | 0.388051945 | 6.52839153 | 6.65E-11 | 6.94E-09 |
| IFT46 | 2303.103305 | 1.371051752 | 0.210027532 | 6.52796203 | 6.67E-11 | 6.94E-09 |
| APOBEC4 | 243.5735525 | 2.685262032 | 0.411545609 | 6.52482246 | 6.81E-11 | 7.06E-09 |
| C6orf118 | 1257.225391 | 2.609424938 | 0.399973532 | 6.52399403 | 6.85E-11 | 7.07E-09 |
| GLRB | 109.3466885 | 1.947122943 | 0.298756782 | 6.51741838 | 7.15E-11 | 7.37E-09 |
| TEKT4 | 1065.318695 | 2.854492316 | 0.438139763 | 6.51502684 | 7.27E-11 | 7.46E-09 |
| CFAP65 | 4572.564686 | 2.630853618 | 0.404092443 | 6.51052417 | 7.49E-11 | 7.63E-09 |
| WDR66 | 2646.491729 | 2.353796902 | 0.361536896 | 6.51053025 | 7.49E-11 | 7.63E-09 |
| SPEF2 | 2788.084693 | 1.417835211 | 0.217992269 | 6.5040619 | 7.82E-11 | 7.94E-09 |
| C9orf24 | 3225.807397 | 2.717831373 | 0.418050068 | 6.50121022 | 7.97E-11 | 8.03E-09 |
| ABHD17AP4 | 9.617892826 | -6.179864317 | 0.950573097 | -6.5011984 | 7.97E-11 | 8.03E-09 |
| FNDC11 | 190.403721 | 2.356095353 | 0.362514006 | 6.49932227 | 8.07E-11 | 8.10E-09 |
| DNALI1 | 6187.337843 | 1.672607373 | 0.25745252 | 6.49676053 | 8.21E-11 | 8.21E-09 |
| MORN3 | 676.9166503 | 2.284167744 | 0.351787801 | 6.49302715 | 8.41E-11 | 8.39E-09 |
| CAPSL | 1384.209968 | 2.606127644 | 0.40143995 | 6.49194891 | 8.47E-11 | 8.39E-09 |
| CDHR3 | 11040.22469 | 2.546782579 | 0.392276966 | 6.49230723 | 8.45E-11 | 8.39E-09 |
| PLPP2 | 800.7838159 | 2.397312523 | 0.369481608 | 6.48831355 | 8.68E-11 | 8.57E-09 |
| RGS22 | 834.7529975 | 2.318541728 | 0.357388427 | 6.4874561 | 8.73E-11 | 8.59E-09 |
| ZMYND10 | 3076.432314 | 2.456888009 | 0.378940346 | 6.48357462 | 8.96E-11 | 8.78E-09 |
| DPCD | 1150.203424 | 1.638313586 | 0.252734805 | 6.48234256 | 9.03E-11 | 8.82E-09 |
| DCDC2B | 1037.557409 | 2.403759634 | 0.370875459 | 6.48131218 | 9.09E-11 | 8.85E-09 |
| SPAG1 | 936.8280873 | 1.575683147 | 0.243271049 | 6.47706808 | 9.35E-11 | 9.07E-09 |
| GIHCG | 642.8335135 | 1.692670649 | 0.261451613 | 6.47412585 | 9.54E-11 | 9.19E-09 |
| TTLL6 | 1199.069745 | 2.315656689 | 0.357662699 | 6.47441485 | 9.52E-11 | 9.19E-09 |
| HOXB3 | 5010.183285 | 1.373208677 | 0.212174805 | 6.47206286 | 9.67E-11 | 9.28E-09 |
| AL117382.2 | 131.0767296 | 2.780268119 | 0.430080135 | 6.46453508 | 1.02E-10 | 9.73E-09 |
| DOC2A | 2352.121348 | 2.641497383 | 0.408697802 | 6.46320428 | 1.03E-10 | 9.78E-09 |
| AC012184.2 | 17.1561169 | -21.31823147 | 3.301190976 | -6.4577395 | 1.06E-10 | 1.01E-08 |
| TNS4 | 177.9216121 | 3.212679775 | 0.49768894 | 6.45519625 | 1.08E-10 | 1.02E-08 |
| TTLL9 | 1340.044358 | 2.563479724 | 0.397094752 | 6.45558701 | 1.08E-10 | 1.02E-08 |
| IQCC | 736.4491687 | 1.316514681 | 0.204005197 | 6.45333894 | 1.09E-10 | 1.03E-08 |
| TTC23L | 138.0725001 | 2.290143016 | 0.355166999 | 6.44807379 | 1.13E-10 | 1.06E-08 |
| FAM183A | 1474.202208 | 2.610386031 | 0.404991691 | 6.44552985 | 1.15E-10 | 1.08E-08 |
| TJP3 | 2937.42478 | 1.567455185 | 0.243274414 | 6.44315676 | 1.17E-10 | 1.09E-08 |
| ROPN1L | 1378.023429 | 2.519715834 | 0.391119432 | 6.4423182 | 1.18E-10 | 1.09E-08 |
| CCDC74A | 2367.566198 | 2.320885439 | 0.360313971 | 6.44128629 | 1.18E-10 | 1.09E-08 |
| FTO | 5048.411902 | 1.654260269 | 0.256810647 | 6.4415564 | 1.18E-10 | 1.09E-08 |
| PRR29 | 5338.279929 | 2.325485378 | 0.361128592 | 6.43949393 | 1.20E-10 | 1.10E-08 |
| IFT22 | 1515.502422 | 1.555328329 | 0.241610586 | 6.43733519 | 1.22E-10 | 1.12E-08 |
| CYP2J2 | 314.4639711 | 1.982204141 | 0.307977216 | 6.43620385 | 1.22E-10 | 1.12E-08 |
| RUVBL1 | 3463.096409 | 1.305848441 | 0.203038699 | 6.43152485 | 1.26E-10 | 1.15E-08 |
| LRRC43 | 1341.513516 | 2.625334626 | 0.408274872 | 6.43031155 | 1.27E-10 | 1.15E-08 |
| DNAL4 | 1932.579283 | 1.040992002 | 0.161882171 | 6.43055376 | 1.27E-10 | 1.15E-08 |
| SLC45A3 | 691.6212638 | 1.811227235 | 0.281729139 | 6.42896662 | 1.28E-10 | 1.15E-08 |
| IQCD | 1814.629576 | 2.368905871 | 0.368473976 | 6.42896386 | 1.28E-10 | 1.15E-08 |
| DNAH5 | 2295.453849 | 2.587716187 | 0.402579815 | 6.42783391 | 1.29E-10 | 1.16E-08 |
| AL590491.2 | 120.1523093 | 2.495768648 | 0.388404064 | 6.42570168 | 1.31E-10 | 1.17E-08 |
| IGSF9 | 297.8720313 | 2.075375498 | 0.323069541 | 6.42392809 | 1.33E-10 | 1.18E-08 |
| FANK1 | 1810.075556 | 2.349921575 | 0.365791249 | 6.42421486 | 1.33E-10 | 1.18E-08 |
| LINC00882 | 204.2611013 | 1.787939127 | 0.278713777 | 6.41496502 | 1.41E-10 | 1.24E-08 |
| C1orf158 | 1022.183153 | 2.711713884 | 0.422799214 | 6.41371553 | 1.42E-10 | 1.25E-08 |
| SPATS1 | 246.3029115 | 2.790352355 | 0.435052813 | 6.41382441 | 1.42E-10 | 1.25E-08 |
| NRAV | 2107.332068 | 1.794592496 | 0.279899254 | 6.41156583 | 1.44E-10 | 1.26E-08 |
| GRHL1 | 757.6755694 | 2.120126555 | 0.330819116 | 6.4087184 | 1.47E-10 | 1.28E-08 |
| AP001273.2 | 15.32948304 | -21.15425945 | 3.30129443 | -6.4078682 | 1.48E-10 | 1.28E-08 |
| LAMA3 | 9882.125661 | -1.334725191 | 0.208513321 | -6.4011507 | 1.54E-10 | 1.34E-08 |
| CCDC24 | 1814.966406 | 1.911195372 | 0.299064763 | 6.39057359 | 1.65E-10 | 1.43E-08 |
| LRRC71 | 2473.67998 | 2.481655843 | 0.388455952 | 6.38851285 | 1.68E-10 | 1.43E-08 |
| RPL13AP17 | 532.5274018 | 2.720723041 | 0.425875232 | 6.3885449 | 1.67E-10 | 1.43E-08 |
| KRT4 | 1464.110197 | 2.371621464 | 0.371210717 | 6.38888198 | 1.67E-10 | 1.43E-08 |
| VWA5B2 | 247.4971852 | 2.607642795 | 0.408262847 | 6.38716654 | 1.69E-10 | 1.44E-08 |
| TSPAN6 | 1633.503413 | 1.086140627 | 0.170256426 | 6.37943985 | 1.78E-10 | 1.51E-08 |
| VSIG1 | 85.70996594 | 4.489800804 | 0.703887319 | 6.3785789 | 1.79E-10 | 1.52E-08 |
| LINC00689 | 219.4837533 | 2.468413698 | 0.387038292 | 6.37769892 | 1.80E-10 | 1.52E-08 |
| PIH1D2 | 322.6852927 | 2.126198883 | 0.333385447 | 6.37759956 | 1.80E-10 | 1.52E-08 |
| ULK4 | 451.7217012 | 1.310650611 | 0.205621181 | 6.37410312 | 1.84E-10 | 1.55E-08 |
| TSPAN1 | 3174.95496 | 2.53304296 | 0.397599096 | 6.37084689 | 1.88E-10 | 1.57E-08 |
| MOBP | 164.4411547 | 2.609177482 | 0.409608837 | 6.36992478 | 1.89E-10 | 1.58E-08 |
| FHAD1 | 4174.423199 | 2.290282765 | 0.359619006 | 6.3686366 | 1.91E-10 | 1.59E-08 |
| PLPPR3 | 1315.235121 | 2.049229504 | 0.322181038 | 6.36049073 | 2.01E-10 | 1.67E-08 |
| ACBD7 | 113.6710898 | 2.307847967 | 0.362963784 | 6.35834226 | 2.04E-10 | 1.69E-08 |
| CCDC96 | 1607.422727 | 2.226750992 | 0.350262954 | 6.35736943 | 2.05E-10 | 1.69E-08 |
| DNAJB13 | 805.2874443 | 2.443510101 | 0.38444663 | 6.355915 | 2.07E-10 | 1.69E-08 |
| PRMT8 | 884.0230717 | 2.351861423 | 0.36998405 | 6.35665624 | 2.06E-10 | 1.69E-08 |
| AL353660.1 | 187.3332173 | 2.66776652 | 0.419720837 | 6.35604975 | 2.07E-10 | 1.69E-08 |
| DCDC1 | 374.8297232 | 2.60857111 | 0.410509645 | 6.35446972 | 2.09E-10 | 1.70E-08 |
| DNAH11 | 1666.48837 | 2.308968211 | 0.363393341 | 6.35390898 | 2.10E-10 | 1.71E-08 |
| C1orf194 | 987.9840681 | 2.639517108 | 0.415523277 | 6.35227256 | 2.12E-10 | 1.71E-08 |
| WHRN | 2086.911823 | 1.78071962 | 0.280310755 | 6.3526625 | 2.12E-10 | 1.71E-08 |
| CFAP47 | 562.2600035 | 2.434559333 | 0.38322451 | 6.35282783 | 2.11E-10 | 1.71E-08 |
| CYP2F1 | 443.3371946 | 3.013551813 | 0.474466548 | 6.35145265 | 2.13E-10 | 1.71E-08 |
| RTN4RL1 | 515.8628592 | 1.632275263 | 0.257073344 | 6.34945357 | 2.16E-10 | 1.73E-08 |
| SOX2 | 1053.341749 | 2.672347733 | 0.420972473 | 6.34803439 | 2.18E-10 | 1.74E-08 |
| LINC00511 | 125.0838782 | 2.478215177 | 0.390431373 | 6.34737715 | 2.19E-10 | 1.74E-08 |
| CASC2 | 643.3721338 | 1.943927984 | 0.306379427 | 6.34483851 | 2.23E-10 | 1.77E-08 |
| PLEKHG7 | 250.7147239 | 3.034092172 | 0.478547 | 6.34021773 | 2.29E-10 | 1.82E-08 |
| DZANK1 | 1071.394135 | 1.863832892 | 0.293978817 | 6.34002447 | 2.30E-10 | 1.82E-08 |
| GPR156 | 87.72939331 | 2.420734797 | 0.381865444 | 6.33923502 | 2.31E-10 | 1.82E-08 |
| AL031283.1 | 90.61352078 | 2.230534786 | 0.352469571 | 6.32830455 | 2.48E-10 | 1.94E-08 |
| NEK10 | 655.5835986 | 2.507751904 | 0.396260633 | 6.32854162 | 2.47E-10 | 1.94E-08 |
| DCST2 | 322.6931269 | 1.983583652 | 0.313603241 | 6.32513761 | 2.53E-10 | 1.98E-08 |
| AL590226.2 | 135.3847397 | 1.92177052 | 0.303923754 | 6.32319947 | 2.56E-10 | 1.99E-08 |
| LINC00365 | 67.31315469 | 1.716203634 | 0.271410601 | 6.32327414 | 2.56E-10 | 1.99E-08 |
| UBASH3B | 966.2467509 | -1.226607284 | 0.19417356 | -6.3170665 | 2.67E-10 | 2.07E-08 |
| PTPRT | 1557.821343 | 2.405014431 | 0.380760584 | 6.31634295 | 2.68E-10 | 2.07E-08 |
| GLIS3 | 517.2822565 | 1.498086837 | 0.237194343 | 6.31586241 | 2.69E-10 | 2.07E-08 |
| WDR54 | 1614.256982 | 1.874765632 | 0.297016018 | 6.31200176 | 2.75E-10 | 2.11E-08 |
| PRR18 | 183.2972336 | 2.79366232 | 0.442614366 | 6.31172989 | 2.76E-10 | 2.11E-08 |
| TRPV4 | 424.6591649 | 1.811078939 | 0.287111661 | 6.3079254 | 2.83E-10 | 2.16E-08 |
| JPT2 | 3560.759987 | 1.043668106 | 0.16546462 | 6.30750009 | 2.84E-10 | 2.16E-08 |
| FBXO16 | 98.25897368 | 1.868350956 | 0.296287675 | 6.30586795 | 2.87E-10 | 2.17E-08 |
| AKAP14 | 291.9697301 | 2.44706122 | 0.388369072 | 6.30086532 | 2.96E-10 | 2.24E-08 |
| RHOV | 368.3799052 | 2.710448636 | 0.430263495 | 6.29950872 | 2.99E-10 | 2.25E-08 |
| KIF21A | 2086.347387 | 1.982722359 | 0.314983439 | 6.29468764 | 3.08E-10 | 2.31E-08 |
| NHLRC4 | 344.9058797 | 1.832224877 | 0.291060514 | 6.29499636 | 3.07E-10 | 2.31E-08 |
| DNAAF4 | 1091.34316 | 1.692413527 | 0.268896655 | 6.2939181 | 3.10E-10 | 2.32E-08 |
| PPP1R32 | 2910.041222 | 2.08704728 | 0.331720012 | 6.29159293 | 3.14E-10 | 2.35E-08 |
| TEX9 | 519.870752 | 2.230404388 | 0.354614614 | 6.28965729 | 3.18E-10 | 2.37E-08 |
| RTKN2 | 5899.656104 | -1.636294219 | 0.260203322 | -6.2885216 | 3.21E-10 | 2.38E-08 |
| CYSRT1 | 217.9521418 | 2.111072695 | 0.33598494 | 6.28323607 | 3.32E-10 | 2.46E-08 |
| TSNAXIP1 | 1482.842163 | 2.1682917 | 0.345488533 | 6.2760164 | 3.47E-10 | 2.55E-08 |
| ANKMY1 | 1925.805152 | 1.406195414 | 0.224341599 | 6.26809926 | 3.65E-10 | 2.67E-08 |
| GOLM1 | 4398.16974 | 1.02812076 | 0.164017358 | 6.26836556 | 3.65E-10 | 2.67E-08 |
| DEUP1 | 174.3808135 | 2.514730818 | 0.401306806 | 6.26635477 | 3.70E-10 | 2.69E-08 |
| DYNLRB2 | 355.0348172 | 2.400096957 | 0.383011197 | 6.26638849 | 3.70E-10 | 2.69E-08 |
| AC093908.1 | 210.3566203 | -1.10913151 | 0.177015117 | -6.2657446 | 3.71E-10 | 2.69E-08 |
| AQP4-AS1 | 112.2536756 | 2.248071443 | 0.358956012 | 6.26280483 | 3.78E-10 | 2.74E-08 |
| KIAA0319 | 479.4403122 | 2.485820413 | 0.396979247 | 6.26183972 | 3.80E-10 | 2.75E-08 |
| CADM1 | 6712.365276 | -1.010621213 | 0.161629776 | -6.252692 | 4.03E-10 | 2.90E-08 |
| LMNTD1 | 94.26032223 | 2.755500343 | 0.440941026 | 6.24913578 | 4.13E-10 | 2.96E-08 |
| AGR3 | 2783.423615 | 1.932630821 | 0.309315362 | 6.24809195 | 4.15E-10 | 2.97E-08 |
| LCA5L | 449.6334164 | 2.288419189 | 0.366315155 | 6.24713216 | 4.18E-10 | 2.98E-08 |
| IL7 | 160.3331379 | 1.699938036 | 0.272205007 | 6.24506528 | 4.24E-10 | 3.01E-08 |
| WDR86 | 492.4080592 | 1.523842211 | 0.244056668 | 6.24380487 | 4.27E-10 | 3.03E-08 |
| ANKRD36BP2 | 388.8875172 | 2.02837619 | 0.325113731 | 6.23897423 | 4.40E-10 | 3.12E-08 |
| SHROOM1 | 1896.122551 | -1.235620446 | 0.198135135 | -6.2362511 | 4.48E-10 | 3.16E-08 |
| HMGB1P1 | 49.14199182 | 2.377870422 | 0.3813561 | 6.23530192 | 4.51E-10 | 3.18E-08 |
| FAM216B | 2733.979126 | 2.510698421 | 0.402812137 | 6.23292645 | 4.58E-10 | 3.22E-08 |
| ARMH2 | 71.50723884 | 2.716244829 | 0.436117744 | 6.22823737 | 4.72E-10 | 3.31E-08 |
| CDH26 | 356.2763393 | 1.787953423 | 0.287373106 | 6.22171451 | 4.92E-10 | 3.44E-08 |
| AL390879.1 | 325.4331462 | 1.676512234 | 0.269545475 | 6.21977511 | 4.98E-10 | 3.47E-08 |
| KLHL13 | 258.5529262 | 1.718313316 | 0.276351381 | 6.2178568 | 5.04E-10 | 3.51E-08 |
| SNCAIP | 597.3578342 | 1.223798757 | 0.196895042 | 6.21548792 | 5.12E-10 | 3.55E-08 |
| CFAP54 | 484.2408749 | 2.171356947 | 0.34952492 | 6.21230941 | 5.22E-10 | 3.61E-08 |
| RNF224 | 166.6265465 | 2.000930146 | 0.322239288 | 6.20945434 | 5.32E-10 | 3.67E-08 |
| TMEM232 | 789.4342632 | 2.372620734 | 0.382180025 | 6.20812334 | 5.36E-10 | 3.69E-08 |
| BRD3OS | 973.4080326 | 1.144132416 | 0.184395556 | 6.20477217 | 5.48E-10 | 3.76E-08 |
| EFHC1 | 2754.033803 | 1.518343702 | 0.24474561 | 6.20376275 | 5.51E-10 | 3.77E-08 |
| PIH1D3 | 324.2930012 | 2.625192235 | 0.423171731 | 6.20360965 | 5.52E-10 | 3.77E-08 |
| AC242022.1 | 90.83078143 | 2.531879446 | 0.408385915 | 6.19972274 | 5.66E-10 | 3.86E-08 |
| KCNJ16 | 356.8623917 | 2.590036284 | 0.417939704 | 6.19715299 | 5.75E-10 | 3.91E-08 |
| MAPK10 | 1890.722239 | 1.315542903 | 0.212791929 | 6.18229699 | 6.32E-10 | 4.29E-08 |
| KIF6 | 653.1945514 | 1.984307178 | 0.321120133 | 6.17932971 | 6.44E-10 | 4.34E-08 |
| CFAP157 | 16063.37877 | 2.356192326 | 0.381274259 | 6.17978337 | 6.42E-10 | 4.34E-08 |
| GIPR | 3721.985392 | 2.319577296 | 0.375409818 | 6.17878698 | 6.46E-10 | 4.35E-08 |
| DNAH7 | 986.0090701 | 2.5835401 | 0.41824658 | 6.17707406 | 6.53E-10 | 4.37E-08 |
| HSPA4L | 329.1976873 | 2.110494257 | 0.341707967 | 6.17630978 | 6.56E-10 | 4.37E-08 |
| BAIAP3 | 3393.957906 | 1.781925816 | 0.288499305 | 6.17653416 | 6.55E-10 | 4.37E-08 |
| AC025279.1 | 307.3986488 | 1.704846373 | 0.275999052 | 6.17700082 | 6.53E-10 | 4.37E-08 |
| WDR5B | 385.4501406 | 1.021120792 | 0.16538609 | 6.17416369 | 6.65E-10 | 4.41E-08 |
| SPATA33 | 1070.189374 | 1.355162745 | 0.219640999 | 6.16989883 | 6.83E-10 | 4.52E-08 |
| RHPN1 | 2797.175823 | 1.515611065 | 0.245872759 | 6.16420895 | 7.08E-10 | 4.66E-08 |
| SLC27A2 | 470.6537156 | 2.264884931 | 0.367415412 | 6.16437106 | 7.08E-10 | 4.66E-08 |
| FAM81A | 185.091906 | 2.080892478 | 0.337732444 | 6.16136388 | 7.21E-10 | 4.73E-08 |
| CCDC40 | 6198.959293 | 1.956273105 | 0.317556502 | 6.1603938 | 7.26E-10 | 4.75E-08 |
| SPATA18 | 2230.474781 | 2.325489659 | 0.377639475 | 6.15796232 | 7.37E-10 | 4.81E-08 |
| NEK5 | 1811.302545 | 2.18323289 | 0.354692706 | 6.15527991 | 7.49E-10 | 4.88E-08 |
| SMKR1 | 94.13158512 | 2.327360846 | 0.378490901 | 6.14905363 | 7.79E-10 | 5.06E-08 |
| TRIP13 | 375.7570804 | 1.889106303 | 0.307258094 | 6.1482719 | 7.83E-10 | 5.08E-08 |
| CTXN1 | 888.9586058 | 2.44911564 | 0.3990781 | 6.13693319 | 8.41E-10 | 5.44E-08 |
| SRI | 3808.548635 | 1.102932427 | 0.1797495 | 6.13594154 | 8.47E-10 | 5.46E-08 |
| DRAIC | 394.6655668 | 2.196656808 | 0.358236539 | 6.1318614 | 8.69E-10 | 5.58E-08 |
| AL354743.2 | 73.21437743 | 2.545905371 | 0.415616069 | 6.12561823 | 9.03E-10 | 5.79E-08 |
| ESPN | 1022.093622 | 2.442366379 | 0.398858554 | 6.12338975 | 9.16E-10 | 5.85E-08 |
| ECT2L | 409.3585411 | 2.287217842 | 0.373523612 | 6.12335545 | 9.16E-10 | 5.85E-08 |
| ANKDD1B | 244.47262 | 1.672443538 | 0.273217197 | 6.12129674 | 9.28E-10 | 5.91E-08 |
| KRT15 | 2427.158872 | 3.254943531 | 0.532103553 | 6.11712422 | 9.53E-10 | 6.04E-08 |
| VSTM2L | 1355.318622 | 2.005440046 | 0.327858644 | 6.11678259 | 9.55E-10 | 6.04E-08 |
| AL137786.1 | 79.51713549 | 2.595776625 | 0.424416706 | 6.11610379 | 9.59E-10 | 6.05E-08 |
| NPM2 | 163.209923 | 1.408753492 | 0.230531101 | 6.11090429 | 9.91E-10 | 6.24E-08 |
| FMN2 | 110.8793558 | 2.450736935 | 0.401247423 | 6.10779483 | 1.01E-09 | 6.35E-08 |
| FGFR3 | 3406.640538 | 1.024151658 | 0.167694765 | 6.10723689 | 1.01E-09 | 6.35E-08 |
| CDS1 | 1552.845398 | 1.199194884 | 0.196608412 | 6.0994078 | 1.06E-09 | 6.64E-08 |
| PKNOX2 | 1925.812654 | -1.240051162 | 0.203303901 | -6.0994952 | 1.06E-09 | 6.64E-08 |
| GOLGA2P5 | 4781.523709 | 1.730126514 | 0.283751102 | 6.09733847 | 1.08E-09 | 6.70E-08 |
| DZIP1L | 2900.295753 | 1.847419449 | 0.303020898 | 6.09667341 | 1.08E-09 | 6.72E-08 |
| STARD9 | 8512.447292 | -1.006585196 | 0.165150807 | -6.0949457 | 1.09E-09 | 6.77E-08 |
| DPY19L2P4 | 44.67575512 | 2.719595056 | 0.446259805 | 6.09419676 | 1.10E-09 | 6.79E-08 |
| ANKRD29 | 3239.715243 | -1.184525181 | 0.194394461 | -6.0934102 | 1.11E-09 | 6.81E-08 |
| TTLL10 | 1673.908621 | 2.422019024 | 0.397543392 | 6.09246455 | 1.11E-09 | 6.83E-08 |
| LDLRAD1 | 2296.770608 | 2.544030862 | 0.417597523 | 6.09206407 | 1.11E-09 | 6.83E-08 |
| KRT80 | 281.5872346 | 1.724229945 | 0.28304204 | 6.09178037 | 1.12E-09 | 6.83E-08 |
| COLCA2 | 344.3433858 | 1.466960811 | 0.241103203 | 6.08436882 | 1.17E-09 | 7.14E-08 |
| NECTIN4 | 1148.043488 | 1.941707504 | 0.319211441 | 6.08282553 | 1.18E-09 | 7.18E-08 |
| TMEM67 | 1036.215135 | 1.627188523 | 0.26750739 | 6.0827797 | 1.18E-09 | 7.18E-08 |
| C7orf57 | 579.357575 | 2.440526848 | 0.401318158 | 6.08127691 | 1.19E-09 | 7.21E-08 |
| AC005821.1 | 96.70370424 | 2.483165722 | 0.408317541 | 6.08145737 | 1.19E-09 | 7.21E-08 |
| RIPK4 | 1041.568351 | 1.021161362 | 0.167922718 | 6.08113882 | 1.19E-09 | 7.21E-08 |
| C3orf67 | 213.4517897 | 2.413811325 | 0.39711959 | 6.07829829 | 1.21E-09 | 7.31E-08 |
| INSC | 139.3122629 | -2.119679818 | 0.348712535 | -6.078588 | 1.21E-09 | 7.31E-08 |
| CLGN | 376.8243994 | 2.110269885 | 0.347401558 | 6.07443989 | 1.24E-09 | 7.46E-08 |
| FAAH2 | 232.2422983 | 1.221942078 | 0.201160019 | 6.07447784 | 1.24E-09 | 7.46E-08 |
| CEP83 | 1635.265391 | 1.209590887 | 0.199365092 | 6.06721507 | 1.30E-09 | 7.78E-08 |
| AP001830.1 | 139.9622293 | 2.076525512 | 0.342409144 | 6.06445695 | 1.32E-09 | 7.90E-08 |
| CENPM | 132.8203821 | 1.70331206 | 0.281030557 | 6.06094966 | 1.35E-09 | 8.06E-08 |
| C1orf87 | 1070.167653 | 2.58804181 | 0.427125339 | 6.05920926 | 1.37E-09 | 8.13E-08 |
| MARCHF10 | 1304.7782 | 2.490161231 | 0.411014058 | 6.05857922 | 1.37E-09 | 8.14E-08 |
| C9orf116 | 647.1154396 | 2.439972963 | 0.402812851 | 6.05733645 | 1.38E-09 | 8.18E-08 |
| ZNF214 | 317.8028071 | 1.464481657 | 0.241783835 | 6.05698747 | 1.39E-09 | 8.18E-08 |
| GAS8 | 5748.953762 | 1.093875507 | 0.180600538 | 6.05687845 | 1.39E-09 | 8.18E-08 |
| CCDC160 | 167.5582775 | 2.112815099 | 0.348868388 | 6.05619532 | 1.39E-09 | 8.20E-08 |
| CCDC30 | 1779.036751 | 1.807132847 | 0.298583269 | 6.05235804 | 1.43E-09 | 8.34E-08 |
| GDF7 | 233.4495877 | 1.812073682 | 0.299397487 | 6.05240111 | 1.43E-09 | 8.34E-08 |
| STX11 | 1429.576429 | -1.157972163 | 0.191357066 | -6.0513687 | 1.44E-09 | 8.38E-08 |
| CD24 | 2045.458721 | 1.670161561 | 0.276264952 | 6.04550649 | 1.49E-09 | 8.67E-08 |
| MYO7B | 819.4750229 | -1.556439756 | 0.257482506 | -6.0448369 | 1.50E-09 | 8.69E-08 |
| NPHP1 | 2458.042037 | 1.772076226 | 0.293262754 | 6.04262287 | 1.52E-09 | 8.79E-08 |
| AZIN1-AS1 | 704.698467 | 1.523578608 | 0.252173347 | 6.04179079 | 1.52E-09 | 8.80E-08 |
| DNAH9 | 3016.414928 | 2.491819019 | 0.412432615 | 6.04176034 | 1.52E-09 | 8.80E-08 |
| CFAP97D2 | 104.3056146 | 2.429968401 | 0.402320045 | 6.03988897 | 1.54E-09 | 8.87E-08 |
| TMEM107 | 1002.608711 | 1.328459114 | 0.21994226 | 6.04003576 | 1.54E-09 | 8.87E-08 |
| OSCP1 | 1371.654294 | 1.776356642 | 0.294218099 | 6.03755053 | 1.56E-09 | 8.94E-08 |
| KIAA1257 | 588.8530687 | 1.3078057 | 0.216595891 | 6.03799868 | 1.56E-09 | 8.94E-08 |
| C22orf15 | 823.3839231 | 2.461775613 | 0.407723193 | 6.03786013 | 1.56E-09 | 8.94E-08 |
| FAM149A | 834.2764931 | 1.315111014 | 0.217870711 | 6.03619921 | 1.58E-09 | 8.99E-08 |
| ENO4 | 161.7791037 | 2.505319766 | 0.415267851 | 6.03302124 | 1.61E-09 | 9.14E-08 |
| RANBP3L | 114.6274089 | -1.452961973 | 0.24097427 | -6.0295316 | 1.64E-09 | 9.32E-08 |
| C15orf65 | 118.9971017 | 1.914832281 | 0.317633232 | 6.0284381 | 1.66E-09 | 9.37E-08 |
| GRIK5 | 151.0723123 | 1.712695854 | 0.28413381 | 6.02777914 | 1.66E-09 | 9.39E-08 |
| CKMT1A | 111.9360439 | 2.332576516 | 0.387221162 | 6.02388697 | 1.70E-09 | 9.60E-08 |
| BBS5 | 996.0815768 | 1.181533764 | 0.196203748 | 6.02197347 | 1.72E-09 | 9.66E-08 |
| MAK | 343.8699571 | 1.969583348 | 0.327064047 | 6.02201118 | 1.72E-09 | 9.66E-08 |
| ATG9B | 546.3183594 | 2.086327006 | 0.346431669 | 6.0223334 | 1.72E-09 | 9.66E-08 |
| EPN3 | 157.6034176 | 2.457843716 | 0.408263901 | 6.02023276 | 1.74E-09 | 9.74E-08 |
| SLC44A4 | 580.3027275 | 2.132693505 | 0.354467574 | 6.01661101 | 1.78E-09 | 9.94E-08 |
| DYNC2H1 | 1435.949409 | 1.432026576 | 0.238023001 | 6.01633695 | 1.78E-09 | 9.94E-08 |
| IFT88 | 1711.61302 | 1.117317597 | 0.185729445 | 6.01583448 | 1.79E-09 | 9.95E-08 |
| CFAP74 | 4123.742079 | 2.322716027 | 0.386165903 | 6.01481386 | 1.80E-09 | 9.99E-08 |
| SERPINI2 | 281.5184359 | 2.021317966 | 0.336135086 | 6.01340964 | 1.82E-09 | 1.00E-07 |
| SAMD15 | 302.0428108 | 2.283931369 | 0.379789378 | 6.01367891 | 1.81E-09 | 1.00E-07 |
| SPAG6 | 2623.520366 | 2.477615171 | 0.412118576 | 6.01189879 | 1.83E-09 | 1.01E-07 |
| DLEC1 | 10071.09191 | 2.231827138 | 0.371409795 | 6.00906915 | 1.87E-09 | 1.03E-07 |
| AL357093.1 | 89.15403176 | 3.049684738 | 0.507589558 | 6.00817076 | 1.88E-09 | 1.03E-07 |
| NWD1 | 945.0016625 | 2.493591494 | 0.415388884 | 6.00302895 | 1.94E-09 | 1.06E-07 |
| AMPD3 | 1098.845808 | 1.545091048 | 0.257548891 | 5.99921453 | 1.98E-09 | 1.09E-07 |
| RABL2B | 5478.960156 | 1.442157506 | 0.240483064 | 5.9969192 | 2.01E-09 | 1.10E-07 |
| HAGHL | 1253.863046 | 1.902440549 | 0.317294687 | 5.99581597 | 2.02E-09 | 1.10E-07 |
| LINC01996 | 188.9873402 | -1.554796992 | 0.259355716 | -5.9948437 | 2.04E-09 | 1.11E-07 |
| TTC21A | 2824.336091 | 1.683795416 | 0.280935236 | 5.99353588 | 2.05E-09 | 1.11E-07 |
| CA2 | 1516.083132 | -1.660752392 | 0.277081923 | -5.9937234 | 2.05E-09 | 1.11E-07 |
| VTCN1 | 65.45668686 | 3.536601823 | 0.591267829 | 5.9813872 | 2.21E-09 | 1.20E-07 |
| CLUAP1 | 3148.612428 | 1.044736625 | 0.174681931 | 5.98079389 | 2.22E-09 | 1.20E-07 |
| HSD11B1L | 339.7629589 | 1.248422313 | 0.208763813 | 5.98007047 | 2.23E-09 | 1.20E-07 |
| PPP1R16A | 4558.506779 | 1.188621582 | 0.198779975 | 5.97958411 | 2.24E-09 | 1.20E-07 |
| CCNA1 | 456.7821684 | 2.64858521 | 0.442979046 | 5.97903046 | 2.24E-09 | 1.21E-07 |
| LINC02166 | 237.919092 | 2.090735165 | 0.349864315 | 5.97584572 | 2.29E-09 | 1.23E-07 |
| C16orf71 | 734.6884216 | 2.482914816 | 0.41554894 | 5.97502383 | 2.30E-09 | 1.23E-07 |
| C6orf141 | 261.8385691 | 1.563472521 | 0.261828361 | 5.97136428 | 2.35E-09 | 1.26E-07 |
| AC013470.2 | 94.56510253 | 2.858452908 | 0.478931696 | 5.96839368 | 2.40E-09 | 1.28E-07 |
| ZMYND12 | 221.6116105 | 2.419025952 | 0.406256092 | 5.95443613 | 2.61E-09 | 1.38E-07 |
| KNDC1 | 2700.546156 | 1.921292292 | 0.322680829 | 5.95415692 | 2.61E-09 | 1.38E-07 |
| TSGA10 | 936.8912029 | 1.983134574 | 0.333577512 | 5.94504876 | 2.76E-09 | 1.46E-07 |
| CCDC33 | 349.9102012 | 2.21678857 | 0.372990578 | 5.94328303 | 2.79E-09 | 1.47E-07 |
| LRRC73 | 261.5287651 | 2.296475086 | 0.386468825 | 5.94220009 | 2.81E-09 | 1.48E-07 |
| CD101 | 354.0999506 | -1.507715508 | 0.253789448 | -5.9408124 | 2.84E-09 | 1.49E-07 |
| CERKL | 217.4751658 | 2.677640343 | 0.450748885 | 5.94042589 | 2.84E-09 | 1.49E-07 |
| ERN2 | 361.3658736 | 3.607067159 | 0.607263461 | 5.93987188 | 2.85E-09 | 1.49E-07 |
| DCST1 | 120.6503942 | 2.002878314 | 0.337211695 | 5.93952803 | 2.86E-09 | 1.49E-07 |
| LGALSL | 1149.38056 | -1.189041321 | 0.200431015 | -5.9324218 | 2.98E-09 | 1.55E-07 |
| RND2 | 175.198985 | 1.501726721 | 0.253141679 | 5.93235665 | 2.99E-09 | 1.55E-07 |
| CCDC181 | 1204.95711 | 2.411917108 | 0.40666395 | 5.93098333 | 3.01E-09 | 1.56E-07 |
| HHATL | 264.3497773 | 2.485400184 | 0.419689436 | 5.92199843 | 3.18E-09 | 1.64E-07 |
| LRRC4 | 1279.990321 | 1.904541174 | 0.321593246 | 5.92220515 | 3.18E-09 | 1.64E-07 |
| AC105916.2 | 115.3162649 | 2.338112163 | 0.395116966 | 5.91751903 | 3.27E-09 | 1.69E-07 |
| CABCOCO1 | 645.576402 | 2.129851109 | 0.359969171 | 5.91675977 | 3.28E-09 | 1.69E-07 |
| LRGUK | 327.6817386 | 1.87249138 | 0.316714013 | 5.91224671 | 3.37E-09 | 1.73E-07 |
| ZNF19 | 448.44686 | 1.209004871 | 0.204602944 | 5.90902969 | 3.44E-09 | 1.77E-07 |
| BBOF1 | 1694.769962 | 1.947633913 | 0.329721644 | 5.90690344 | 3.49E-09 | 1.78E-07 |
| ACSBG1 | 582.1200839 | 2.158511802 | 0.365432383 | 5.90673379 | 3.49E-09 | 1.78E-07 |
| STPG1 | 829.8811436 | 1.334914782 | 0.226091708 | 5.90430668 | 3.54E-09 | 1.80E-07 |
| IQCK | 691.1861941 | 1.826427863 | 0.309335235 | 5.90436411 | 3.54E-09 | 1.80E-07 |
| GRHL2 | 1640.039365 | 1.032010069 | 0.174798944 | 5.90398342 | 3.55E-09 | 1.80E-07 |
| AC005041.1 | 295.5727387 | 2.898600625 | 0.491103453 | 5.90222001 | 3.59E-09 | 1.82E-07 |
| HOXB2 | 1061.08512 | 1.32515234 | 0.224754074 | 5.89601033 | 3.72E-09 | 1.89E-07 |
| SCEL | 3311.056349 | -1.449649045 | 0.246257609 | -5.8867178 | 3.94E-09 | 1.99E-07 |
| PPP1R42 | 133.6736279 | 2.692915059 | 0.45761175 | 5.88471572 | 3.99E-09 | 2.01E-07 |
| DNAAF1 | 18169.79025 | 2.388487669 | 0.405983919 | 5.88320758 | 4.02E-09 | 2.02E-07 |
| DNAH12 | 2114.157095 | 2.475168435 | 0.420921844 | 5.88035159 | 4.09E-09 | 2.06E-07 |
| FAM86EP | 122.3793081 | 1.301278245 | 0.221359126 | 5.87858413 | 4.14E-09 | 2.07E-07 |
| MIPEP | 696.5637429 | 1.099206008 | 0.187082901 | 5.87550226 | 4.22E-09 | 2.11E-07 |
| WDR38 | 1374.252225 | 2.480868552 | 0.42234586 | 5.87402124 | 4.25E-09 | 2.12E-07 |
| C20orf96 | 2166.222664 | 1.174252926 | 0.199913648 | 5.87380071 | 4.26E-09 | 2.12E-07 |
| MYLK3 | 241.9782128 | 2.115907476 | 0.360276443 | 5.8730109 | 4.28E-09 | 2.13E-07 |
| PWWP3B | 58.61780704 | 2.493287676 | 0.424666207 | 5.87117043 | 4.33E-09 | 2.15E-07 |
| INKA2 | 836.3993722 | -1.152337467 | 0.196296687 | -5.8703867 | 4.35E-09 | 2.16E-07 |
| CFAP69 | 975.580705 | 1.626827337 | 0.277271071 | 5.86728117 | 4.43E-09 | 2.19E-07 |
| AL121820.3 | 136.4842284 | 1.404257679 | 0.239423393 | 5.8651649 | 4.49E-09 | 2.22E-07 |
| KLHL32 | 216.9019954 | 2.343479902 | 0.399706792 | 5.86299745 | 4.55E-09 | 2.24E-07 |
| DNAH10 | 1722.928203 | 2.363133304 | 0.403386338 | 5.85823833 | 4.68E-09 | 2.30E-07 |
| MOK | 3180.317403 | 1.573943601 | 0.268839924 | 5.85457539 | 4.78E-09 | 2.35E-07 |
| HYDIN | 3478.721681 | 2.29614227 | 0.39256965 | 5.84900609 | 4.95E-09 | 2.43E-07 |
| AC110998.1 | 113.3250413 | 2.392609632 | 0.409094402 | 5.84855139 | 4.96E-09 | 2.43E-07 |
| SPAG17 | 2647.582652 | 2.470259794 | 0.422530887 | 5.84634134 | 5.03E-09 | 2.46E-07 |
| LRRIQ1 | 1123.586479 | 2.411271849 | 0.412542127 | 5.84491059 | 5.07E-09 | 2.48E-07 |
| TCTEX1D1 | 773.4968675 | 2.207824376 | 0.378110816 | 5.83909342 | 5.25E-09 | 2.56E-07 |
| WRAP53 | 610.3800753 | 1.172450111 | 0.200829531 | 5.8380364 | 5.28E-09 | 2.57E-07 |
| AC023796.1 | 103.3765165 | 3.344543715 | 0.573086423 | 5.83601981 | 5.35E-09 | 2.60E-07 |
| EFCAB10 | 294.1542996 | 2.323693626 | 0.398185735 | 5.83570284 | 5.36E-09 | 2.60E-07 |
| TTC34 | 195.6392476 | 1.77087766 | 0.303475306 | 5.83532704 | 5.37E-09 | 2.60E-07 |
| PSPH | 408.1792426 | 1.360122516 | 0.233108084 | 5.83472907 | 5.39E-09 | 2.60E-07 |
| TMEM74B | 2524.041 | -1.238399904 | 0.212249141 | -5.8346521 | 5.39E-09 | 2.60E-07 |
| MAP3K19 | 771.9610965 | 2.474489553 | 0.424196328 | 5.8333592 | 5.43E-09 | 2.62E-07 |
| CFAP299 | 330.9024383 | 2.620172301 | 0.449188461 | 5.83312469 | 5.44E-09 | 2.62E-07 |
| AC084871.4 | 51.10902156 | -1.937265562 | 0.332182928 | -5.8319239 | 5.48E-09 | 2.63E-07 |
| ADGRE1 | 390.4477713 | -2.145028906 | 0.36782026 | -5.8317312 | 5.49E-09 | 2.63E-07 |
| AC022107.1 | 261.2197848 | 1.944017269 | 0.333444892 | 5.83010062 | 5.54E-09 | 2.65E-07 |
| ANKRD66 | 469.4911386 | 2.479341706 | 0.42564319 | 5.82492982 | 5.71E-09 | 2.73E-07 |
| RABL2A | 3861.325136 | 1.519982193 | 0.260985524 | 5.82400958 | 5.75E-09 | 2.74E-07 |
| FLACC1 | 567.8634745 | 2.387676018 | 0.410036917 | 5.82307573 | 5.78E-09 | 2.75E-07 |
| CFAP298 | 7067.586852 | 1.12331475 | 0.192903742 | 5.82318798 | 5.77E-09 | 2.75E-07 |
| PITPNM1 | 3973.706018 | 1.311461827 | 0.225228903 | 5.82279544 | 5.79E-09 | 2.75E-07 |
| CFAP99 | 1803.063925 | 2.451669632 | 0.421172747 | 5.82105478 | 5.85E-09 | 2.77E-07 |
| SLC22A4 | 445.1274981 | 2.138368272 | 0.367813778 | 5.81372532 | 6.11E-09 | 2.89E-07 |
| OMG | 753.7214277 | 2.021964682 | 0.34791912 | 5.81159403 | 6.19E-09 | 2.92E-07 |
| CRACR2B | 6004.819292 | 1.384563581 | 0.238366074 | 5.80855974 | 6.30E-09 | 2.96E-07 |
| AL449403.3 | 121.8102528 | 2.876061792 | 0.495595045 | 5.80324969 | 6.50E-09 | 3.05E-07 |
| RPGRIP1L | 588.006469 | 1.715502539 | 0.295614628 | 5.80317203 | 6.51E-09 | 3.05E-07 |
| MLF1 | 1204.302412 | 1.535710007 | 0.264658048 | 5.80261972 | 6.53E-09 | 3.05E-07 |
| TMEM234 | 1572.615908 | 1.085029606 | 0.187053025 | 5.80065255 | 6.61E-09 | 3.08E-07 |
| HNF4A | 83.12442916 | 2.315340385 | 0.399164068 | 5.80047297 | 6.61E-09 | 3.08E-07 |
| MET | 3622.866675 | 1.19892401 | 0.206716745 | 5.79983983 | 6.64E-09 | 3.09E-07 |
| CCDC191 | 3817.44685 | 1.37569475 | 0.237224554 | 5.79912462 | 6.67E-09 | 3.09E-07 |
| RFX3 | 1728.564983 | 1.382337564 | 0.238377582 | 5.79894113 | 6.67E-09 | 3.09E-07 |
| GLB1L | 761.5884416 | 1.582456251 | 0.272918974 | 5.79826396 | 6.70E-09 | 3.10E-07 |
| TTC16 | 969.2355484 | 2.109108835 | 0.364082139 | 5.79294783 | 6.92E-09 | 3.19E-07 |
| GYG2 | 272.2550644 | 1.889862 | 0.326232631 | 5.79298887 | 6.91E-09 | 3.19E-07 |
| MAP1A | 2863.855977 | 1.927863418 | 0.332868672 | 5.79166374 | 6.97E-09 | 3.21E-07 |
| LRRC74B | 1320.228321 | 2.403611653 | 0.415034693 | 5.79135117 | 6.98E-09 | 3.21E-07 |
| MSI2 | 2389.854914 | 1.300196477 | 0.224531739 | 5.7907024 | 7.01E-09 | 3.21E-07 |
| GRIN1 | 63.1820152 | 1.502383587 | 0.259539316 | 5.7886551 | 7.10E-09 | 3.25E-07 |
| DPY19L2 | 480.955624 | 1.281327327 | 0.221443553 | 5.78624805 | 7.20E-09 | 3.29E-07 |
| LINC01267 | 319.5801151 | 2.640833992 | 0.457003827 | 5.77858179 | 7.53E-09 | 3.44E-07 |
| ELN-AS1 | 692.2032148 | 2.064576553 | 0.357296935 | 5.77832147 | 7.54E-09 | 3.44E-07 |
| PLEKHB1 | 1960.015309 | 1.472286153 | 0.254883302 | 5.77631465 | 7.64E-09 | 3.47E-07 |
| C5orf49 | 2930.246093 | 2.368929588 | 0.41017554 | 5.77540433 | 7.68E-09 | 3.49E-07 |
| SEMA5A | 3277.396705 | -1.173478813 | 0.203243594 | -5.7737555 | 7.75E-09 | 3.51E-07 |
| CFAP100 | 4335.956213 | 2.345250852 | 0.406223547 | 5.77330111 | 7.77E-09 | 3.51E-07 |
| RAB3B | 35.49079044 | 2.226266561 | 0.385861259 | 5.76960373 | 7.95E-09 | 3.58E-07 |
| DNPH1 | 1577.032566 | 1.172824582 | 0.203277667 | 5.76956927 | 7.95E-09 | 3.58E-07 |
| OXTR | 417.0224205 | 2.469926216 | 0.428116288 | 5.76928812 | 7.96E-09 | 3.58E-07 |
| AL353622.1 | 116.5521224 | 1.595942592 | 0.276697117 | 5.76783238 | 8.03E-09 | 3.60E-07 |
| CCDC170 | 1845.606924 | 2.200206411 | 0.381445675 | 5.76807277 | 8.02E-09 | 3.60E-07 |
| MDM1 | 1462.187262 | 1.110909417 | 0.192653308 | 5.76636564 | 8.10E-09 | 3.63E-07 |
| AC092058.1 | 97.08163581 | 2.784693707 | 0.483171866 | 5.76336062 | 8.25E-09 | 3.69E-07 |
| DPY19L2P2 | 406.1520804 | 2.376981652 | 0.412479085 | 5.76267194 | 8.28E-09 | 3.70E-07 |
| MEIG1 | 69.16148075 | 2.527902147 | 0.438697702 | 5.76228719 | 8.30E-09 | 3.70E-07 |
| LZTFL1 | 1347.899001 | 1.043224271 | 0.181135002 | 5.75937428 | 8.44E-09 | 3.75E-07 |
| LAMC3 | 5484.712079 | -1.503228924 | 0.261222053 | -5.7546019 | 8.68E-09 | 3.85E-07 |
| DNAJA4 | 5969.586429 | 1.672616426 | 0.290721428 | 5.75333038 | 8.75E-09 | 3.88E-07 |
| PZP | 580.4533345 | 1.879047512 | 0.326723947 | 5.75117781 | 8.86E-09 | 3.91E-07 |
| NRG4 | 234.536762 | 2.413661747 | 0.419673174 | 5.75128909 | 8.86E-09 | 3.91E-07 |
| GJB7 | 165.6308397 | 2.527700932 | 0.439595589 | 5.75005982 | 8.92E-09 | 3.93E-07 |
| MAPK15 | 3397.873445 | 2.211967377 | 0.385043726 | 5.74471736 | 9.21E-09 | 4.05E-07 |
| GET1 | 1123.31792 | 1.062298689 | 0.184938098 | 5.74407708 | 9.24E-09 | 4.06E-07 |
| AL121899.2 | 167.7335751 | 2.373932821 | 0.41343414 | 5.74198546 | 9.36E-09 | 4.10E-07 |
| AC009093.8 | 90.72051849 | 2.052048268 | 0.357613484 | 5.73817364 | 9.57E-09 | 4.19E-07 |
| CSMD1 | 178.8758907 | 2.186137209 | 0.381027842 | 5.73747367 | 9.61E-09 | 4.20E-07 |
| B4GALNT3 | 2662.515656 | 1.064760591 | 0.185854576 | 5.72899852 | 1.01E-08 | 4.41E-07 |
| LINC00683 | 249.9983115 | 1.675013168 | 0.292400934 | 5.72848091 | 1.01E-08 | 4.41E-07 |
| Z95115.1 | 344.7751781 | 1.512652793 | 0.264109503 | 5.72736981 | 1.02E-08 | 4.43E-07 |
| CFAP70 | 2715.37394 | 2.124239331 | 0.371176165 | 5.72299498 | 1.05E-08 | 4.54E-07 |
| B9D1 | 1724.459342 | 1.878754449 | 0.328544215 | 5.71842194 | 1.08E-08 | 4.65E-07 |
| WDR49 | 419.9996039 | 2.095547858 | 0.366754879 | 5.71375591 | 1.11E-08 | 4.76E-07 |
| FAM166C | 219.8774112 | 2.560551042 | 0.448288268 | 5.71184041 | 1.12E-08 | 4.81E-07 |
| RHPN2 | 732.1688945 | 1.628308524 | 0.285185388 | 5.70964921 | 1.13E-08 | 4.86E-07 |
| AC112204.1 | 13.46844418 | 3.034093105 | 0.531666204 | 5.70676315 | 1.15E-08 | 4.94E-07 |
| NME7 | 912.6686062 | 1.161221753 | 0.203553988 | 5.70473596 | 1.17E-08 | 4.99E-07 |
| CFAP300 | 455.6393196 | 2.343678347 | 0.410969315 | 5.70280618 | 1.18E-08 | 5.04E-07 |
| STRBP | 1574.236185 | 1.233772624 | 0.216420934 | 5.70080075 | 1.19E-08 | 5.08E-07 |
| GPRC5D | 386.1856089 | -1.523755483 | 0.26729302 | -5.7006931 | 1.19E-08 | 5.08E-07 |
| APLN | 1726.038832 | -2.507252565 | 0.439811706 | -5.7007409 | 1.19E-08 | 5.08E-07 |
| TNNI3 | 713.3136887 | 2.374336594 | 0.416599971 | 5.69932011 | 1.20E-08 | 5.11E-07 |
| CCDC187 | 4790.259059 | 2.362641603 | 0.414613955 | 5.69841312 | 1.21E-08 | 5.13E-07 |
| TBC1D8 | 3379.668965 | 1.162101814 | 0.203953169 | 5.69788556 | 1.21E-08 | 5.14E-07 |
| EDNRA | 2724.430204 | -1.150227655 | 0.20199924 | -5.6942177 | 1.24E-08 | 5.23E-07 |
| ALDH3B1 | 8566.95067 | 1.07695017 | 0.189205406 | 5.69196301 | 1.26E-08 | 5.29E-07 |
| PALM2AKAP2 | 19054.65389 | -1.14368989 | 0.200952648 | -5.6913402 | 1.26E-08 | 5.30E-07 |
| AMY1C | 566.7326928 | 2.071351377 | 0.36401245 | 5.69033114 | 1.27E-08 | 5.32E-07 |
| TCTEX1D2 | 376.1701621 | 1.571958885 | 0.276258344 | 5.69017703 | 1.27E-08 | 5.32E-07 |
| Z92544.1 | 213.7243814 | 1.440323027 | 0.253195728 | 5.68857554 | 1.28E-08 | 5.36E-07 |
| TOB1 | 2648.815784 | 1.359375454 | 0.238997174 | 5.68783067 | 1.29E-08 | 5.37E-07 |
| LRWD1 | 1451.793493 | 1.386006612 | 0.243891189 | 5.68288923 | 1.32E-08 | 5.52E-07 |
| STEAP3 | 1393.232746 | 1.459096115 | 0.256778947 | 5.68230431 | 1.33E-08 | 5.53E-07 |
| ZSCAN1 | 97.2570001 | 1.805643566 | 0.317960211 | 5.67883498 | 1.36E-08 | 5.64E-07 |
| C4orf19 | 441.428154 | 1.25519381 | 0.221177458 | 5.67505307 | 1.39E-08 | 5.76E-07 |
| CFAP161 | 273.075106 | 1.824295428 | 0.321564921 | 5.67317922 | 1.40E-08 | 5.81E-07 |
| TTC30B | 659.6154591 | 1.690901456 | 0.298679024 | 5.66126618 | 1.50E-08 | 6.22E-07 |
| KLHDC9 | 398.9442165 | 1.861655554 | 0.328882803 | 5.66054393 | 1.51E-08 | 6.24E-07 |
| RUVBL2 | 7655.567501 | 1.211140638 | 0.214043705 | 5.6583801 | 1.53E-08 | 6.31E-07 |
| AP003717.1 | 197.9393655 | 2.255970283 | 0.398963302 | 5.65458094 | 1.56E-08 | 6.44E-07 |
| IFITM10 | 164.4666652 | 1.928106277 | 0.341079994 | 5.65294451 | 1.58E-08 | 6.48E-07 |
| KCNN3 | 549.0896462 | 1.241557041 | 0.219687572 | 5.65146689 | 1.59E-08 | 6.52E-07 |
| WDR31 | 299.5305614 | 1.538856742 | 0.272281657 | 5.65170919 | 1.59E-08 | 6.52E-07 |
| FBXW9 | 590.7038084 | 1.654679126 | 0.292845458 | 5.65034929 | 1.60E-08 | 6.55E-07 |
| TRPV2 | 1748.150983 | -1.043106191 | 0.184722988 | -5.6468672 | 1.63E-08 | 6.68E-07 |
| FAM83F | 348.3111935 | 2.329231784 | 0.412502156 | 5.64659299 | 1.64E-08 | 6.68E-07 |
| MAPK8IP1 | 1150.577351 | 1.264724149 | 0.224065465 | 5.64444035 | 1.66E-08 | 6.75E-07 |
| COL4A4 | 2987.631904 | -1.290505489 | 0.228653928 | -5.6439244 | 1.66E-08 | 6.76E-07 |
| RASSF9 | 683.7021319 | 1.190488945 | 0.211043099 | 5.64097547 | 1.69E-08 | 6.86E-07 |
| ARHGAP40 | 230.3247425 | 2.621420789 | 0.464926192 | 5.63835902 | 1.72E-08 | 6.96E-07 |
| AGR2 | 3802.796162 | 1.781818684 | 0.316067057 | 5.63747041 | 1.73E-08 | 6.98E-07 |
| AL096711.2 | 917.3049141 | -2.098325645 | 0.372574017 | -5.6319699 | 1.78E-08 | 7.20E-07 |
| DNAH2 | 2784.286088 | 2.279212427 | 0.405076057 | 5.62662835 | 1.84E-08 | 7.41E-07 |
| CRISPLD1 | 359.6782806 | 1.222683457 | 0.217359394 | 5.62516961 | 1.85E-08 | 7.47E-07 |
| SRD5A2 | 351.4953059 | 2.777742943 | 0.49385751 | 5.62458379 | 1.86E-08 | 7.48E-07 |
| EDNRB | 8372.229268 | -1.785241678 | 0.31742633 | -5.6241134 | 1.86E-08 | 7.49E-07 |
| IFT172 | 5752.101105 | 1.254134336 | 0.223054624 | 5.62254355 | 1.88E-08 | 7.55E-07 |
| CACNG6 | 712.1611995 | 2.399126834 | 0.426720861 | 5.62223939 | 1.88E-08 | 7.55E-07 |
| CCDC39 | 1090.789196 | 2.311280911 | 0.411313634 | 5.61926648 | 1.92E-08 | 7.67E-07 |
| ZC2HC1C | 632.8266106 | 1.457993003 | 0.259492129 | 5.61864057 | 1.92E-08 | 7.69E-07 |
| CDNF | 138.1473218 | 1.373329696 | 0.244551801 | 5.61570061 | 1.96E-08 | 7.81E-07 |
| CKMT1B | 107.1906508 | 2.047115407 | 0.364634396 | 5.61415881 | 1.98E-08 | 7.87E-07 |
| ANKRD44-AS1 | 328.1804463 | 2.298108187 | 0.409827283 | 5.60750414 | 2.05E-08 | 8.17E-07 |
| PSENEN | 1545.216279 | 1.369743494 | 0.244343093 | 5.60582041 | 2.07E-08 | 8.23E-07 |
| LRRC63 | 47.03535535 | 2.655859207 | 0.47382392 | 5.60516069 | 2.08E-08 | 8.25E-07 |
| SAMD5 | 829.7723406 | -1.224568264 | 0.218539886 | -5.6034085 | 2.10E-08 | 8.33E-07 |
| AQP5 | 663.9864219 | 2.167468872 | 0.386864163 | 5.60266129 | 2.11E-08 | 8.35E-07 |
| TPPP3 | 32837.74033 | 1.693761928 | 0.302398922 | 5.60108454 | 2.13E-08 | 8.42E-07 |
| ARMC2 | 720.6201152 | 1.504957834 | 0.268724672 | 5.6003709 | 2.14E-08 | 8.44E-07 |
| AC127070.2 | 125.7088649 | 2.047683113 | 0.365660104 | 5.59996316 | 2.14E-08 | 8.45E-07 |
| DYDC2 | 998.7352929 | 2.416585029 | 0.432089086 | 5.59279349 | 2.23E-08 | 8.78E-07 |
| NEK2 | 131.4399126 | 1.510915046 | 0.270216257 | 5.59150314 | 2.25E-08 | 8.83E-07 |
| CFAP46 | 3309.865439 | 2.230869701 | 0.399340886 | 5.5863794 | 2.32E-08 | 9.08E-07 |
| ROPN1B | 100.5403997 | 2.756007993 | 0.493390518 | 5.58585521 | 2.33E-08 | 9.10E-07 |
| ANKK1 | 126.0607148 | 1.971989004 | 0.353088795 | 5.58496626 | 2.34E-08 | 9.13E-07 |
| AC079848.2 | 147.8046005 | 2.075988829 | 0.37239173 | 5.57474471 | 2.48E-08 | 9.66E-07 |
| CDK20 | 1111.037427 | 1.189404097 | 0.213359207 | 5.5746556 | 2.48E-08 | 9.66E-07 |
| ZNF837 | 283.8266065 | 1.19887713 | 0.215113916 | 5.57321978 | 2.50E-08 | 9.73E-07 |
| PCOLCE2 | 567.8734449 | -1.473303626 | 0.264405344 | -5.5721401 | 2.52E-08 | 9.78E-07 |
| AC092171.3 | 156.3153344 | 1.348023171 | 0.24198571 | 5.57067262 | 2.54E-08 | 9.83E-07 |
| PCSK4 | 627.1265627 | 1.656122575 | 0.297281879 | 5.57088303 | 2.53E-08 | 9.83E-07 |
| AC004832.1 | 328.8222982 | 2.474376759 | 0.444383924 | 5.56810592 | 2.58E-08 | 9.97E-07 |
| C11orf97 | 742.3823037 | 2.372042462 | 0.426038012 | 5.56767799 | 2.58E-08 | 9.98E-07 |
| CYP2T1P | 263.0914054 | 1.203518484 | 0.216317919 | 5.56365599 | 2.64E-08 | 1.02E-06 |
| ACADL | 1526.350526 | -1.201009705 | 0.215955088 | -5.5613865 | 2.68E-08 | 1.03E-06 |
| AK9 | 1054.776089 | 1.40566072 | 0.252775297 | 5.56091017 | 2.68E-08 | 1.03E-06 |
| SIRPB1 | 795.6326552 | -1.820896231 | 0.327820864 | -5.5545465 | 2.78E-08 | 1.07E-06 |
| SLC23A1 | 239.4274803 | 2.361877327 | 0.425233198 | 5.55431076 | 2.79E-08 | 1.07E-06 |
| SDR16C5 | 1218.453536 | -1.372592334 | 0.247240139 | -5.5516565 | 2.83E-08 | 1.08E-06 |
| IFT81 | 2159.704136 | 1.291008448 | 0.232604902 | 5.55022029 | 2.85E-08 | 1.09E-06 |
| COLEC10 | 232.3558322 | -2.614130309 | 0.471264022 | -5.5470611 | 2.91E-08 | 1.11E-06 |
| SRGAP3 | 903.3113991 | 1.491584825 | 0.268942687 | 5.54610665 | 2.92E-08 | 1.11E-06 |
| SLC4A8 | 799.454107 | 1.586502028 | 0.286318167 | 5.54104564 | 3.01E-08 | 1.14E-06 |
| SOX7 | 889.2541086 | -1.743652518 | 0.314772324 | -5.5394086 | 3.03E-08 | 1.15E-06 |
| FAM86JP | 110.0316947 | 1.920955321 | 0.346965556 | 5.53644385 | 3.09E-08 | 1.17E-06 |
| PITPNM2 | 4202.894283 | -1.017268217 | 0.18380148 | -5.534603 | 3.12E-08 | 1.18E-06 |
| MCOLN3 | 689.6843019 | -1.313820202 | 0.237419889 | -5.5337411 | 3.13E-08 | 1.18E-06 |
| CAPS2 | 479.7564765 | 1.676440013 | 0.303011268 | 5.53259957 | 3.16E-08 | 1.19E-06 |
| TRAF3IP1 | 4596.720573 | 1.219776866 | 0.220483242 | 5.53228833 | 3.16E-08 | 1.19E-06 |
| FSD1L | 370.1308935 | 1.318408063 | 0.238343097 | 5.53155548 | 3.17E-08 | 1.19E-06 |
| DOCK6 | 11076.78752 | -1.001870012 | 0.1813648 | -5.5240599 | 3.31E-08 | 1.24E-06 |
| LRTOMT | 1105.367113 | 1.546690395 | 0.280046608 | 5.52297493 | 3.33E-08 | 1.25E-06 |
| TCTN1 | 1938.643616 | 1.29562072 | 0.234626422 | 5.52205803 | 3.35E-08 | 1.26E-06 |
| ANKRD42 | 1505.202322 | 1.215352634 | 0.220205684 | 5.51917012 | 3.41E-08 | 1.27E-06 |
| AL136985.3 | 42.84444679 | 1.918037518 | 0.347572774 | 5.51837675 | 3.42E-08 | 1.28E-06 |
| P4HTM | 3207.639193 | 1.055832271 | 0.191375673 | 5.51706627 | 3.45E-08 | 1.29E-06 |
| AC108134.3 | 759.2029377 | -1.008109456 | 0.182796618 | -5.514924 | 3.49E-08 | 1.30E-06 |
| LINC00639 | 105.8065 | 2.332514375 | 0.42303879 | 5.51371276 | 3.51E-08 | 1.31E-06 |
| ENPP5 | 561.9918433 | 1.608447952 | 0.291849923 | 5.51121596 | 3.56E-08 | 1.33E-06 |
| CORO6 | 1155.84289 | -1.319018381 | 0.239361326 | -5.5105743 | 3.58E-08 | 1.33E-06 |
| LINC02795 | 113.2695494 | 1.640826858 | 0.297801501 | 5.50980049 | 3.59E-08 | 1.33E-06 |
| PIGR | 13550.82712 | 1.862524993 | 0.338067031 | 5.50933638 | 3.60E-08 | 1.33E-06 |
| REEP2 | 99.16964217 | 1.791715156 | 0.325567919 | 5.50335292 | 3.73E-08 | 1.38E-06 |
| GPC5-AS1 | 46.43175496 | 2.71044009 | 0.492508101 | 5.50334114 | 3.73E-08 | 1.38E-06 |
| ANKRD65 | 1709.950721 | 1.310608343 | 0.238273448 | 5.50043806 | 3.79E-08 | 1.40E-06 |
| IFTAP | 1207.744019 | 1.182932618 | 0.215072207 | 5.50016496 | 3.79E-08 | 1.40E-06 |
| SHC4 | 49.8880418 | 1.54673775 | 0.281247016 | 5.49957036 | 3.81E-08 | 1.40E-06 |
| AC073439.1 | 986.1560521 | 2.354329868 | 0.428315182 | 5.4967229 | 3.87E-08 | 1.42E-06 |
| ADPRS | 3374.611553 | 1.405104359 | 0.25580168 | 5.49294421 | 3.95E-08 | 1.45E-06 |
| LRP4 | 2634.000462 | -1.281027701 | 0.233235934 | -5.4924114 | 3.96E-08 | 1.45E-06 |
| CENPS | 865.5389058 | 1.078170093 | 0.196333722 | 5.49151761 | 3.98E-08 | 1.46E-06 |
| IQUB | 245.3891363 | 2.103213645 | 0.383212055 | 5.4883807 | 4.06E-08 | 1.48E-06 |
| DPEP3 | 12.99755032 | -6.618153643 | 1.206346368 | -5.486114 | 4.11E-08 | 1.49E-06 |
| AFF2 | 492.7579697 | -1.607079813 | 0.292999964 | -5.4849147 | 4.14E-08 | 1.50E-06 |
| AC015813.8 | 190.6036266 | 2.361346505 | 0.430784576 | 5.48150197 | 4.22E-08 | 1.53E-06 |
| FRMPD2 | 674.2423486 | 2.183907192 | 0.398747706 | 5.47691474 | 4.33E-08 | 1.56E-06 |
| FAM27E3 | 423.793136 | 1.728871321 | 0.315932377 | 5.47228283 | 4.44E-08 | 1.60E-06 |
| AFF3 | 1837.641178 | -1.168132015 | 0.213514291 | -5.4709781 | 4.48E-08 | 1.61E-06 |
| FAM86C2P | 372.4476543 | 1.419536788 | 0.259652735 | 5.46705887 | 4.58E-08 | 1.65E-06 |
| UGT1A6 | 81.60191007 | 4.715785556 | 0.86299818 | 5.46442121 | 4.64E-08 | 1.67E-06 |
| FER1L4 | 464.7508614 | 1.702740254 | 0.311950543 | 5.45836605 | 4.81E-08 | 1.72E-06 |
| USP43 | 1346.900639 | 1.772394388 | 0.324805616 | 5.45678493 | 4.85E-08 | 1.74E-06 |
| AC105052.5 | 366.7204447 | 2.432558312 | 0.445812293 | 5.45646307 | 4.86E-08 | 1.74E-06 |
| H2BU1 | 48.09488627 | 2.228604175 | 0.408680835 | 5.45316537 | 4.95E-08 | 1.77E-06 |
| MORN1 | 1601.691345 | 1.577282763 | 0.289365481 | 5.45083248 | 5.01E-08 | 1.79E-06 |
| IFT27 | 2801.224636 | 1.272346357 | 0.23346261 | 5.44989349 | 5.04E-08 | 1.79E-06 |
| ADAMTS7 | 1984.727917 | -1.088082629 | 0.199745418 | -5.4473471 | 5.11E-08 | 1.82E-06 |
| DMKN | 5314.653642 | 1.118108134 | 0.205387308 | 5.44390083 | 5.21E-08 | 1.85E-06 |
| PRR36 | 654.6756915 | 1.505090061 | 0.276586205 | 5.44166713 | 5.28E-08 | 1.87E-06 |
| FAM89A | 501.8016517 | -1.103487406 | 0.202833858 | -5.4403511 | 5.32E-08 | 1.88E-06 |
| FLT4 | 8689.131562 | -1.078312482 | 0.198213001 | -5.4401703 | 5.32E-08 | 1.88E-06 |
| KRT17 | 1944.264768 | 3.716520069 | 0.683243853 | 5.43952215 | 5.34E-08 | 1.88E-06 |
| SMIM34B | 122.4944052 | 2.15683355 | 0.396513311 | 5.43949848 | 5.34E-08 | 1.88E-06 |
| TTC30A | 461.4995028 | 1.29381774 | 0.237963162 | 5.43705055 | 5.42E-08 | 1.90E-06 |
| AMY1B | 665.552886 | 2.115648179 | 0.389223013 | 5.43556806 | 5.46E-08 | 1.92E-06 |
| RIMS4 | 113.6583095 | -1.806380294 | 0.332359746 | -5.4350153 | 5.48E-08 | 1.92E-06 |
| ESRRG | 89.89405445 | 1.894867687 | 0.348723491 | 5.43372538 | 5.52E-08 | 1.93E-06 |
| AC004923.1 | 21.56285147 | 2.601457332 | 0.478753005 | 5.43381933 | 5.52E-08 | 1.93E-06 |
| SPATA4 | 130.7833178 | 2.457236767 | 0.452355512 | 5.43209202 | 5.57E-08 | 1.94E-06 |
| GPD1 | 1227.16451 | -1.815587133 | 0.334294119 | -5.431107 | 5.60E-08 | 1.95E-06 |
| AC005962.2 | 31.45532142 | 2.849165199 | 0.524662467 | 5.43047269 | 5.62E-08 | 1.96E-06 |
| AMY1A | 604.5448305 | 1.995873603 | 0.367615104 | 5.4292481 | 5.66E-08 | 1.97E-06 |
| SOX2-OT | 69.6666767 | 1.566619476 | 0.289730871 | 5.40715414 | 6.40E-08 | 2.22E-06 |
| CEP19 | 314.5775343 | 1.406891466 | 0.260339298 | 5.40406876 | 6.51E-08 | 2.25E-06 |
| SLC25A18 | 159.7615103 | -1.453299124 | 0.268967887 | -5.403244 | 6.54E-08 | 2.26E-06 |
| CC2D2A | 2336.496904 | 1.284381554 | 0.237833417 | 5.400341 | 6.65E-08 | 2.29E-06 |
| CLDN8 | 176.205443 | 2.578847624 | 0.477533294 | 5.40035147 | 6.65E-08 | 2.29E-06 |
| SYCP2L | 426.3047956 | -1.606779228 | 0.298091056 | -5.3902296 | 7.04E-08 | 2.42E-06 |
| VWA3A | 2423.165233 | 1.79973976 | 0.333896661 | 5.39011009 | 7.04E-08 | 2.42E-06 |
| C2orf81 | 943.1805559 | 1.764357383 | 0.327355824 | 5.38972351 | 7.06E-08 | 2.42E-06 |
| CLDN1 | 655.1961559 | 1.498664051 | 0.278092033 | 5.3890938 | 7.08E-08 | 2.43E-06 |
| NUP62CL | 92.92829759 | 1.986020697 | 0.368626137 | 5.38762854 | 7.14E-08 | 2.44E-06 |
| ASTN2 | 166.5590922 | 1.37146884 | 0.254796972 | 5.38259473 | 7.34E-08 | 2.51E-06 |
| CDH2 | 258.4422461 | 1.610165687 | 0.299214055 | 5.38131703 | 7.39E-08 | 2.52E-06 |
| DPEP2 | 1377.10476 | -1.401915173 | 0.260627363 | -5.3790023 | 7.49E-08 | 2.55E-06 |
| AC008674.1 | 134.8775548 | 2.882378822 | 0.535935269 | 5.37822194 | 7.52E-08 | 2.56E-06 |
| PLA2G7 | 202.5947339 | 2.567027665 | 0.477643145 | 5.37436304 | 7.69E-08 | 2.61E-06 |
| PPP1R36 | 176.827808 | 1.863990397 | 0.347177347 | 5.36898624 | 7.92E-08 | 2.69E-06 |
| IL1RL1 | 1827.95913 | -3.620805449 | 0.674672134 | -5.366763 | 8.02E-08 | 2.71E-06 |
| EFNB3 | 733.9534698 | 1.922563557 | 0.358224089 | 5.36692985 | 8.01E-08 | 2.71E-06 |
| BX005040.1 | 50.01398925 | 2.682910894 | 0.50008729 | 5.36488518 | 8.10E-08 | 2.74E-06 |
| ADGRA1 | 153.2454472 | 2.705002172 | 0.504249931 | 5.36440763 | 8.12E-08 | 2.74E-06 |
| BMPER | 1290.215932 | -1.276833266 | 0.238184545 | -5.360689 | 8.29E-08 | 2.79E-06 |
| AC010538.1 | 487.8126077 | 1.465078533 | 0.273322632 | 5.36025326 | 8.31E-08 | 2.79E-06 |
| CEP41 | 629.5977472 | 1.074148685 | 0.200404305 | 5.35990823 | 8.33E-08 | 2.79E-06 |
| DEGS2 | 330.8554322 | 1.753773622 | 0.327278711 | 5.35865476 | 8.38E-08 | 2.81E-06 |
| ILDR1 | 370.4351747 | 1.091241602 | 0.203689687 | 5.35737287 | 8.44E-08 | 2.82E-06 |
| FAM189A1 | 170.3472832 | -1.833234745 | 0.342172699 | -5.3576301 | 8.43E-08 | 2.82E-06 |
| ANKRD54 | 1769.573727 | 1.148019012 | 0.214289707 | 5.35732223 | 8.45E-08 | 2.82E-06 |
| SCGB3A1 | 11774.38525 | 3.629315401 | 0.677775358 | 5.35474676 | 8.57E-08 | 2.85E-06 |
| FAM227A | 1596.867978 | 2.226149086 | 0.415727241 | 5.35483092 | 8.56E-08 | 2.85E-06 |
| ZDHHC13 | 549.2898084 | 1.039023919 | 0.194060577 | 5.35412155 | 8.60E-08 | 2.86E-06 |
| CDKN2B-AS1 | 64.82308588 | 1.829736805 | 0.341826234 | 5.35282733 | 8.66E-08 | 2.88E-06 |
| NCS1 | 631.3937935 | 1.053390159 | 0.196821727 | 5.35200141 | 8.70E-08 | 2.89E-06 |
| FABP6-AS1 | 291.9872456 | 2.677360453 | 0.500524736 | 5.34910717 | 8.84E-08 | 2.93E-06 |
| SPATA24 | 215.2967445 | 1.580480225 | 0.295540053 | 5.34776999 | 8.90E-08 | 2.95E-06 |
| ETNK2 | 273.108156 | 1.058692707 | 0.198023042 | 5.34631069 | 8.98E-08 | 2.97E-06 |
| CD5L | 106.6380488 | -2.237358746 | 0.418525983 | -5.3458061 | 9.00E-08 | 2.97E-06 |
| SIAH3 | 36.05202904 | 1.902498394 | 0.35589444 | 5.3456817 | 9.01E-08 | 2.97E-06 |
| MYCBPAP | 1086.95545 | 2.146262954 | 0.401640909 | 5.34373593 | 9.11E-08 | 3.00E-06 |
| MAT1A | 234.5748615 | 2.369262616 | 0.443489833 | 5.34231552 | 9.18E-08 | 3.02E-06 |
| NAT1 | 307.4890794 | 1.507791092 | 0.282526695 | 5.3368093 | 9.46E-08 | 3.11E-06 |
| RASSF10 | 292.7951313 | 1.807712426 | 0.33885845 | 5.33471255 | 9.57E-08 | 3.14E-06 |
| CBLC | 81.93427825 | 1.740108284 | 0.326276634 | 5.33322985 | 9.65E-08 | 3.16E-06 |
| BIK | 68.56106241 | 1.388085941 | 0.260335531 | 5.33191122 | 9.72E-08 | 3.18E-06 |
| ESM1 | 1301.479535 | -2.023443227 | 0.379519426 | -5.3315933 | 9.74E-08 | 3.18E-06 |
| AC007695.1 | 53.4927722 | 2.066865988 | 0.388027944 | 5.32659057 | 1.00E-07 | 3.27E-06 |
| SMIM22 | 332.9489976 | 1.87689318 | 0.352636086 | 5.32246486 | 1.02E-07 | 3.33E-06 |
| MAMDC2 | 2710.238782 | -1.145940765 | 0.215495762 | -5.3176951 | 1.05E-07 | 3.41E-06 |
| NAP1L3 | 235.5697131 | 1.379717283 | 0.259452055 | 5.31781212 | 1.05E-07 | 3.41E-06 |
| CCDC103 | 604.0618153 | 2.302258227 | 0.432973409 | 5.31732013 | 1.05E-07 | 3.41E-06 |
| SMIM5 | 738.1326834 | 1.534089557 | 0.288551939 | 5.316511 | 1.06E-07 | 3.42E-06 |
| PDLIM4 | 518.2162205 | 1.31925254 | 0.248183388 | 5.31563594 | 1.06E-07 | 3.43E-06 |
| ZNF440 | 1323.832752 | 1.365905383 | 0.257050241 | 5.31376816 | 1.07E-07 | 3.46E-06 |
| TTC41P | 144.5641739 | 1.717909887 | 0.323458168 | 5.31107282 | 1.09E-07 | 3.51E-06 |
| SEPTIN4 | 3685.831639 | -1.054548295 | 0.198688622 | -5.3075424 | 1.11E-07 | 3.57E-06 |
| C6orf132 | 1683.306108 | 1.293046185 | 0.243686552 | 5.30618606 | 1.12E-07 | 3.59E-06 |
| VWA7 | 92.55343713 | 1.690072633 | 0.318817916 | 5.30105916 | 1.15E-07 | 3.69E-06 |
| LINC01091 | 230.0347325 | 1.339032736 | 0.252657305 | 5.29979822 | 1.16E-07 | 3.71E-06 |
| COL4A3 | 4704.385227 | -1.51129028 | 0.285323292 | -5.2967645 | 1.18E-07 | 3.77E-06 |
| PNMA8A | 459.6236265 | 1.887353596 | 0.356583819 | 5.29287504 | 1.20E-07 | 3.84E-06 |
| ZDHHC1 | 1529.550415 | 1.266323318 | 0.239356698 | 5.29052802 | 1.22E-07 | 3.88E-06 |
| BICDL2 | 4859.809895 | 1.231250882 | 0.232791627 | 5.28906859 | 1.23E-07 | 3.90E-06 |
| SORD | 840.7154217 | 1.3168491 | 0.249155589 | 5.2852481 | 1.26E-07 | 3.97E-06 |
| CRNDE | 1498.698389 | 1.095854629 | 0.207594576 | 5.27882109 | 1.30E-07 | 4.10E-06 |
| ADH6 | 70.26435655 | 1.801136357 | 0.341522609 | 5.27384223 | 1.34E-07 | 4.21E-06 |
| KCNK3 | 6494.814582 | -1.459294753 | 0.276764095 | -5.2727026 | 1.34E-07 | 4.23E-06 |
| ZNHIT2 | 449.6105429 | 1.191608334 | 0.226130256 | 5.26956611 | 1.37E-07 | 4.29E-06 |
| TREM1 | 2363.453954 | -1.318031001 | 0.250171282 | -5.2685144 | 1.38E-07 | 4.31E-06 |
| PLA2G4F | 9997.836729 | -1.594060758 | 0.302815423 | -5.2641333 | 1.41E-07 | 4.40E-06 |
| DCDC2 | 1238.318723 | 1.081026399 | 0.205495976 | 5.26057211 | 1.44E-07 | 4.48E-06 |
| GSTA1 | 1274.550197 | 2.426707743 | 0.46164651 | 5.25663617 | 1.47E-07 | 4.57E-06 |
| KLK10 | 2287.219844 | 1.505318804 | 0.286695756 | 5.25057931 | 1.52E-07 | 4.70E-06 |
| SYTL3 | 1320.344793 | 1.44079421 | 0.27448339 | 5.24911257 | 1.53E-07 | 4.73E-06 |
| AC112204.3 | 50.87209283 | 2.793869735 | 0.532766888 | 5.24407541 | 1.57E-07 | 4.85E-06 |
| PAK6 | 396.0484711 | 1.289792944 | 0.245997831 | 5.24310697 | 1.58E-07 | 4.87E-06 |
| CLDN9 | 133.5752279 | 1.67571094 | 0.319707679 | 5.24138471 | 1.59E-07 | 4.91E-06 |
| TCTN2 | 1443.054402 | 1.278746457 | 0.243998182 | 5.24080323 | 1.60E-07 | 4.92E-06 |
| TIGD4 | 79.737981 | 2.18888255 | 0.417737393 | 5.23985305 | 1.61E-07 | 4.94E-06 |
| AC110619.1 | 49.94294307 | 1.671261452 | 0.318977862 | 5.23942772 | 1.61E-07 | 4.95E-06 |
| VIPR1-AS1 | 364.7127405 | -1.932567845 | 0.369179966 | -5.2347582 | 1.65E-07 | 5.06E-06 |
| CXXC4 | 325.4280097 | 1.127151089 | 0.215325535 | 5.23463734 | 1.65E-07 | 5.06E-06 |
| EDARADD | 136.4595644 | 1.664861145 | 0.318338306 | 5.22984861 | 1.70E-07 | 5.19E-06 |
| LBX1-AS1 | 74.29974476 | -2.110300017 | 0.403602401 | -5.2286607 | 1.71E-07 | 5.21E-06 |
| HRK | 91.44818073 | 2.092798507 | 0.40027978 | 5.22833931 | 1.71E-07 | 5.22E-06 |
| FBXL13 | 80.51715858 | 1.432242992 | 0.274198938 | 5.22337177 | 1.76E-07 | 5.35E-06 |
| TMEM200C | 74.67581377 | 2.023892113 | 0.387526775 | 5.22258653 | 1.76E-07 | 5.37E-06 |
| TMEM45A | 451.6161797 | 1.04711836 | 0.20053336 | 5.22166667 | 1.77E-07 | 5.39E-06 |
| ENPP1 | 214.4807279 | -1.255175728 | 0.240890982 | -5.2105551 | 1.88E-07 | 5.69E-06 |
| ECRG4 | 716.7878164 | 1.344736712 | 0.258090882 | 5.21032243 | 1.89E-07 | 5.69E-06 |
| B3GALNT1 | 1345.501501 | -1.293866486 | 0.248342681 | -5.2100045 | 1.89E-07 | 5.69E-06 |
| C1orf189 | 118.2250268 | 2.31293998 | 0.443972013 | 5.20965267 | 1.89E-07 | 5.70E-06 |
| AC097374.1 | 64.76577151 | 2.539446739 | 0.487661054 | 5.20740116 | 1.92E-07 | 5.76E-06 |
| PDZD2 | 3351.32985 | -1.354096506 | 0.260173253 | -5.2045954 | 1.94E-07 | 5.84E-06 |
| C6orf52 | 33.47466463 | 1.876605168 | 0.360614677 | 5.20390679 | 1.95E-07 | 5.86E-06 |
| CLCNKA | 159.2321341 | 1.958150428 | 0.376353952 | 5.20294903 | 1.96E-07 | 5.88E-06 |
| CATIP | 745.6678691 | 1.751796954 | 0.336913921 | 5.19953864 | 2.00E-07 | 5.98E-06 |
| AC044849.1 | 42.0387188 | 1.814222999 | 0.349260787 | 5.19446519 | 2.05E-07 | 6.14E-06 |
| B3GALT5 | 74.65905686 | 2.452007093 | 0.472185634 | 5.19288796 | 2.07E-07 | 6.18E-06 |
| SLC24A4 | 360.5533126 | -1.003643668 | 0.193399865 | -5.1894745 | 2.11E-07 | 6.29E-06 |
| LGR5 | 115.0667212 | 1.741003921 | 0.335912138 | 5.18291459 | 2.18E-07 | 6.51E-06 |
| PPP1R26-AS1 | 295.3905548 | 1.148409298 | 0.221708153 | 5.17982438 | 2.22E-07 | 6.61E-06 |
| C5orf46 | 134.2290033 | 2.737648438 | 0.528717551 | 5.17790347 | 2.24E-07 | 6.67E-06 |
| BEST4 | 239.5645437 | 1.980601037 | 0.382664589 | 5.17581478 | 2.27E-07 | 6.74E-06 |
| PART1 | 91.45787481 | 2.593084181 | 0.50106129 | 5.17518362 | 2.28E-07 | 6.75E-06 |
| NAT14 | 695.0309393 | 1.029547467 | 0.198982718 | 5.17405471 | 2.29E-07 | 6.78E-06 |
| PLXNB3 | 863.8167892 | -1.152244271 | 0.222689708 | -5.1742143 | 2.29E-07 | 6.78E-06 |
| TMEM45B | 394.7786894 | 1.356383337 | 0.262160956 | 5.17385715 | 2.29E-07 | 6.78E-06 |
| KIAA2012 | 1086.996143 | 2.426973667 | 0.469340543 | 5.17102924 | 2.33E-07 | 6.87E-06 |
| TMEM184A | 943.7669692 | 1.44722658 | 0.279881834 | 5.17084856 | 2.33E-07 | 6.87E-06 |
| AL163051.1 | 79.2548407 | 2.370681553 | 0.458650436 | 5.16882002 | 2.36E-07 | 6.93E-06 |
| LANCL1-AS1 | 184.8491593 | -1.635735634 | 0.316514412 | -5.1679657 | 2.37E-07 | 6.94E-06 |
| LMX1B | 51.61237205 | 1.836958432 | 0.355443822 | 5.16806965 | 2.37E-07 | 6.94E-06 |
| EML6 | 270.5717657 | 1.708925213 | 0.330825355 | 5.16564159 | 2.40E-07 | 7.01E-06 |
| HMGA1P8 | 85.12399045 | 2.113662299 | 0.409195668 | 5.16540732 | 2.40E-07 | 7.01E-06 |
| NOTCH4 | 2058.160323 | -1.30848762 | 0.253337064 | -5.1650066 | 2.40E-07 | 7.02E-06 |
| KLK13 | 144.4924079 | 2.951978871 | 0.571658858 | 5.16388197 | 2.42E-07 | 7.05E-06 |
| CEP126 | 2150.021891 | 1.773768145 | 0.3435597 | 5.16291097 | 2.43E-07 | 7.08E-06 |
| PAG1 | 2785.026718 | -1.116352614 | 0.216315903 | -5.1607515 | 2.46E-07 | 7.15E-06 |
| LRRC27 | 2159.99086 | 1.096008771 | 0.21237951 | 5.16061447 | 2.46E-07 | 7.15E-06 |
| STARD4 | 719.8612934 | -1.123729704 | 0.217802899 | -5.1593882 | 2.48E-07 | 7.19E-06 |
| B9D2 | 475.8813289 | 1.128443482 | 0.218779003 | 5.1579149 | 2.50E-07 | 7.24E-06 |
| HCN4 | 162.999772 | 2.342906104 | 0.454839166 | 5.15106499 | 2.59E-07 | 7.49E-06 |
| DCST1-AS1 | 70.67368821 | 2.204981653 | 0.428114762 | 5.15044527 | 2.60E-07 | 7.51E-06 |
| UBE3D | 281.0464411 | 1.460104821 | 0.283523567 | 5.14985345 | 2.61E-07 | 7.53E-06 |
| NXF2 | 104.7993042 | 2.374517494 | 0.461120668 | 5.14944929 | 2.61E-07 | 7.53E-06 |
| FAIM | 1309.322069 | 1.035730159 | 0.201203359 | 5.14767828 | 2.64E-07 | 7.59E-06 |
| C17orf97 | 592.8920346 | 1.842766795 | 0.35800351 | 5.14734282 | 2.64E-07 | 7.60E-06 |
| FENDRR | 8359.174111 | -1.652936576 | 0.321147482 | -5.1469704 | 2.65E-07 | 7.60E-06 |
| UNC119B | 3430.639666 | 1.040241785 | 0.202208818 | 5.14439378 | 2.68E-07 | 7.70E-06 |
| DNAJC9-AS1 | 133.2265027 | 1.808998307 | 0.351668102 | 5.14405003 | 2.69E-07 | 7.71E-06 |
| FA2H | 164.9565115 | 1.10981237 | 0.215803443 | 5.14270003 | 2.71E-07 | 7.76E-06 |
| RCAN3AS | 21.81808652 | 2.343628115 | 0.455876571 | 5.14092687 | 2.73E-07 | 7.82E-06 |
| FAM161A | 643.2560967 | 1.38080352 | 0.26876127 | 5.13765813 | 2.78E-07 | 7.94E-06 |
| RSPH10B2 | 1147.986335 | 2.831482781 | 0.551520208 | 5.13396017 | 2.84E-07 | 8.09E-06 |
| EGLN3 | 203.7830745 | 1.149529218 | 0.224063174 | 5.13037997 | 2.89E-07 | 8.24E-06 |
| HEYL | 2527.817783 | -1.101243725 | 0.214661376 | -5.1301438 | 2.90E-07 | 8.24E-06 |
| TOX3 | 108.5238729 | 1.821901437 | 0.355189623 | 5.12937687 | 2.91E-07 | 8.27E-06 |
| BCO2 | 738.2251634 | 1.350952098 | 0.263400538 | 5.1288889 | 2.91E-07 | 8.28E-06 |
| CFAP44 | 3936.45598 | 1.386954851 | 0.270460093 | 5.12813123 | 2.93E-07 | 8.31E-06 |
| CYB5D1 | 1556.851388 | 1.683705416 | 0.328402923 | 5.12695015 | 2.94E-07 | 8.35E-06 |
| AP003721.4 | 25.81540221 | 2.412533902 | 0.470615079 | 5.12634212 | 2.95E-07 | 8.37E-06 |
| SORCS1 | 21.91396883 | 1.953487664 | 0.381357311 | 5.12246025 | 3.02E-07 | 8.53E-06 |
| ALDH1L1 | 95.42089685 | 1.872697551 | 0.365638617 | 5.12171709 | 3.03E-07 | 8.56E-06 |
| UBXN11 | 10856.64193 | 1.442184386 | 0.281679308 | 5.11995146 | 3.06E-07 | 8.63E-06 |
| VIPR1 | 15867.67202 | -1.987164342 | 0.38817775 | -5.1192124 | 3.07E-07 | 8.66E-06 |
| FPR2 | 471.5815674 | -1.885938855 | 0.368449834 | -5.118577 | 3.08E-07 | 8.68E-06 |
| LINC00475 | 65.6104362 | 2.398612704 | 0.468845639 | 5.11599662 | 3.12E-07 | 8.79E-06 |
| SHROOM4 | 5342.80604 | -1.127682876 | 0.220597999 | -5.1119361 | 3.19E-07 | 8.96E-06 |
| CLHC1 | 687.0720004 | 1.214946161 | 0.237955539 | 5.10576961 | 3.29E-07 | 9.25E-06 |
| RS1 | 239.8581426 | -1.659134527 | 0.324988488 | -5.1052101 | 3.30E-07 | 9.27E-06 |
| C16orf96 | 86.93747164 | 1.635411591 | 0.320382558 | 5.10455875 | 3.32E-07 | 9.29E-06 |
| C9orf43 | 73.12249377 | 1.640459772 | 0.321696248 | 5.09940598 | 3.41E-07 | 9.53E-06 |
| TMPRSS3 | 474.5640424 | 1.528881337 | 0.299921261 | 5.09760906 | 3.44E-07 | 9.61E-06 |
| MUC15 | 1104.732896 | 1.326618418 | 0.260277727 | 5.09693409 | 3.45E-07 | 9.62E-06 |
| AF064858.1 | 419.7058894 | -1.626200827 | 0.319242333 | -5.0939386 | 3.51E-07 | 9.77E-06 |
| TBX5 | 4565.335897 | -1.107068094 | 0.217368376 | -5.0930504 | 3.52E-07 | 9.80E-06 |
| LRP11 | 1583.552418 | 1.204806598 | 0.236978204 | 5.08403972 | 3.69E-07 | 1.03E-05 |
| RADX | 371.8540937 | 1.675171692 | 0.329530567 | 5.08350928 | 3.71E-07 | 1.03E-05 |
| AC026801.2 | 108.112492 | 1.567313745 | 0.308445171 | 5.08133663 | 3.75E-07 | 1.04E-05 |
| INAVA | 514.5746203 | 1.143633573 | 0.225118751 | 5.08013468 | 3.77E-07 | 1.04E-05 |
| TRPC3 | 195.0725344 | -1.392951507 | 0.27439351 | -5.076474 | 3.85E-07 | 1.06E-05 |
| AGBL4 | 87.23511516 | 2.113685315 | 0.416712342 | 5.07228872 | 3.93E-07 | 1.08E-05 |
| AC106820.3 | 24.90638815 | 1.629340259 | 0.321254463 | 5.07180583 | 3.94E-07 | 1.09E-05 |
| AC139100.1 | 34.40447119 | 1.885503669 | 0.37189314 | 5.07001466 | 3.98E-07 | 1.10E-05 |
| AC046134.2 | 204.911247 | 1.036379878 | 0.204459182 | 5.06888401 | 4.00E-07 | 1.10E-05 |
| EFCAB2 | 1974.766234 | 1.447877015 | 0.285937553 | 5.06361267 | 4.11E-07 | 1.13E-05 |
| AL591686.2 | 196.8925013 | -1.370364315 | 0.27089694 | -5.0586187 | 4.22E-07 | 1.16E-05 |
| PAIP2B | 877.2451959 | 1.021389126 | 0.201943084 | 5.05780691 | 4.24E-07 | 1.16E-05 |
| SYT8 | 243.9339384 | 1.484413642 | 0.293517511 | 5.05732566 | 4.25E-07 | 1.16E-05 |
| MROH9 | 152.2716029 | 2.504791781 | 0.495341391 | 5.05669792 | 4.27E-07 | 1.16E-05 |
| FUZ | 1665.944997 | 1.226558546 | 0.242606011 | 5.05576322 | 4.29E-07 | 1.17E-05 |
| PRRT3 | 299.7736111 | 1.163800565 | 0.230589466 | 5.04706735 | 4.49E-07 | 1.22E-05 |
| IL32 | 9380.241106 | -1.133081781 | 0.224528512 | -5.046494 | 4.50E-07 | 1.22E-05 |
| COL7A1 | 3455.558209 | 1.292834281 | 0.256290248 | 5.04441465 | 4.55E-07 | 1.23E-05 |
| PPFIBP1 | 6270.482355 | -1.160049691 | 0.230083449 | -5.041865 | 4.61E-07 | 1.25E-05 |
| TUBB4B | 16330.60939 | 1.135781743 | 0.225435889 | 5.03815851 | 4.70E-07 | 1.27E-05 |
| TTLL13P | 38.67910529 | 1.600654033 | 0.317834197 | 5.03612905 | 4.75E-07 | 1.28E-05 |
| CCDC141 | 282.7858514 | -1.650133387 | 0.327737728 | -5.0349204 | 4.78E-07 | 1.29E-05 |
| AP000944.7 | 155.788152 | -1.305804975 | 0.259427744 | -5.0334053 | 4.82E-07 | 1.30E-05 |
| GPR162 | 2371.434901 | 1.34193729 | 0.266660723 | 5.03237701 | 4.84E-07 | 1.30E-05 |
| SPIRE2 | 1589.803801 | 1.250768369 | 0.248637259 | 5.03049452 | 4.89E-07 | 1.31E-05 |
| AL161618.1 | 30.25524104 | 2.559869905 | 0.509042185 | 5.02879718 | 4.94E-07 | 1.32E-05 |
| AL157700.1 | 38.76332324 | -1.150926074 | 0.228975067 | -5.0264253 | 5.00E-07 | 1.34E-05 |
| FFAR4 | 345.3626592 | -1.692376717 | 0.336907096 | -5.0232742 | 5.08E-07 | 1.36E-05 |
| AP001207.3 | 89.17099133 | 2.489720917 | 0.49574197 | 5.02221128 | 5.11E-07 | 1.36E-05 |
| CYP11A1 | 21.43840799 | 2.109023979 | 0.419938528 | 5.02222073 | 5.11E-07 | 1.36E-05 |
| TMCO1-AS1 | 29.10009798 | 1.287670069 | 0.25643768 | 5.02137623 | 5.13E-07 | 1.37E-05 |
| AL135905.1 | 53.98316291 | 1.209621556 | 0.240971077 | 5.01977902 | 5.17E-07 | 1.38E-05 |
| PTPN5 | 337.4980026 | -2.679592087 | 0.534195689 | -5.0161245 | 5.27E-07 | 1.40E-05 |
| MIR223HG | 592.2389509 | -1.707062166 | 0.340555913 | -5.0125753 | 5.37E-07 | 1.43E-05 |
| LINC00886 | 165.9118331 | 1.294041201 | 0.258262827 | 5.01055927 | 5.43E-07 | 1.44E-05 |
| ERICH3 | 6635.064338 | 2.278953297 | 0.455045763 | 5.00818485 | 5.49E-07 | 1.46E-05 |
| CROCC2 | 4205.658112 | 2.118247475 | 0.423102569 | 5.00646328 | 5.54E-07 | 1.47E-05 |
| PLCH2 | 447.0747746 | 1.569779646 | 0.313780169 | 5.0028007 | 5.65E-07 | 1.49E-05 |
| H4C15 | 197.5683853 | 1.021045353 | 0.204157827 | 5.00125499 | 5.70E-07 | 1.50E-05 |
| EFCAB11 | 392.6719507 | 1.169261884 | 0.233867246 | 4.9996821 | 5.74E-07 | 1.51E-05 |
| DNAH3 | 1912.632193 | 2.300939823 | 0.460202045 | 4.99984702 | 5.74E-07 | 1.51E-05 |
| CLBA1 | 1766.956883 | 1.355066568 | 0.271051609 | 4.99929358 | 5.75E-07 | 1.51E-05 |
| HPGD | 6217.842228 | -1.726730874 | 0.346127022 | -4.9887202 | 6.08E-07 | 1.59E-05 |
| SPN | 2247.424165 | -1.141651007 | 0.228926596 | -4.9869741 | 6.13E-07 | 1.60E-05 |
| AL513477.1 | 321.7331072 | 1.177947963 | 0.236218163 | 4.98669514 | 6.14E-07 | 1.60E-05 |
| AC004943.2 | 235.4656991 | 1.046906915 | 0.20995154 | 4.98642169 | 6.15E-07 | 1.60E-05 |
| KCNJ2-AS1 | 56.37805945 | 1.368756832 | 0.274892141 | 4.9792505 | 6.38E-07 | 1.66E-05 |
| AC125611.4 | 384.646172 | 1.960398155 | 0.393762651 | 4.97862901 | 6.40E-07 | 1.66E-05 |
| DYDC1 | 173.9712408 | 2.436008946 | 0.489382208 | 4.97772274 | 6.43E-07 | 1.67E-05 |
| AC025154.2 | 37.88642132 | 2.914756252 | 0.585648075 | 4.97697572 | 6.46E-07 | 1.67E-05 |
| KATNAL2 | 602.7201684 | 1.33901541 | 0.269047198 | 4.97687922 | 6.46E-07 | 1.67E-05 |
| PKIB | 189.3777096 | 1.343576602 | 0.270144068 | 4.97355581 | 6.57E-07 | 1.70E-05 |
| ENTPD3 | 211.8984193 | 1.176472711 | 0.236686279 | 4.97059955 | 6.67E-07 | 1.72E-05 |
| CNIH2 | 99.1978652 | 1.648102771 | 0.331564021 | 4.97069244 | 6.67E-07 | 1.72E-05 |
| RNF213-AS1 | 626.3144437 | 1.207818076 | 0.243038925 | 4.96964869 | 6.71E-07 | 1.72E-05 |
| DALRD3 | 1579.351724 | 1.15275254 | 0.232240911 | 4.96360668 | 6.92E-07 | 1.77E-05 |
| HSPBP1 | 1760.888538 | 1.011362519 | 0.203796801 | 4.96260251 | 6.96E-07 | 1.78E-05 |
| PALD1 | 5006.227822 | -1.121317193 | 0.225977857 | -4.9620667 | 6.97E-07 | 1.79E-05 |
| CCDC34 | 699.4193406 | 1.094553434 | 0.220763 | 4.95804748 | 7.12E-07 | 1.82E-05 |
| C8orf37 | 186.4107108 | 1.215945618 | 0.245294252 | 4.95708974 | 7.16E-07 | 1.83E-05 |
| DENND6B | 3466.900861 | 1.434689085 | 0.289770466 | 4.95112255 | 7.38E-07 | 1.88E-05 |
| KIF9 | 637.7288407 | 1.167735798 | 0.235894708 | 4.95024161 | 7.41E-07 | 1.88E-05 |
| MCIDAS | 93.52475121 | 2.883860136 | 0.582675425 | 4.94934231 | 7.45E-07 | 1.89E-05 |
| C2orf73 | 175.4509084 | 2.375430377 | 0.480063305 | 4.94816069 | 7.49E-07 | 1.90E-05 |
| SDK1 | 1151.127061 | 1.06452191 | 0.215377097 | 4.94259569 | 7.71E-07 | 1.95E-05 |
| AL133320.2 | 162.4480517 | 2.375588858 | 0.480740359 | 4.94152158 | 7.75E-07 | 1.96E-05 |
| CYP2W1 | 41.3268154 | 2.765980616 | 0.559773597 | 4.9412488 | 7.76E-07 | 1.96E-05 |
| C6orf201 | 109.2250186 | 2.117582159 | 0.428600769 | 4.94068679 | 7.78E-07 | 1.96E-05 |
| TMEM59L | 123.3707217 | 1.949710352 | 0.394706384 | 4.93964737 | 7.83E-07 | 1.97E-05 |
| TLCD1 | 230.7625715 | 1.393094689 | 0.282054408 | 4.93909915 | 7.85E-07 | 1.97E-05 |
| LNC-LBCS | 229.26996 | 1.835061278 | 0.371767917 | 4.93603991 | 7.97E-07 | 2.00E-05 |
| CTSE | 1945.944962 | 1.410241431 | 0.285716607 | 4.93580491 | 7.98E-07 | 2.00E-05 |
| INTU | 1172.772133 | 1.069011705 | 0.216595975 | 4.93551049 | 7.99E-07 | 2.00E-05 |
| HSD17B13 | 430.2026528 | 3.314253242 | 0.67163617 | 4.93459613 | 8.03E-07 | 2.01E-05 |
| AC144831.1 | 359.0503259 | -1.147313327 | 0.232522016 | -4.9342137 | 8.05E-07 | 2.01E-05 |
| NRG2 | 311.8154799 | 1.013750888 | 0.20547616 | 4.93366671 | 8.07E-07 | 2.01E-05 |
| AC010980.2 | 32.98530345 | -2.414173004 | 0.489572959 | -4.9311813 | 8.17E-07 | 2.03E-05 |
| HNF1A-AS1 | 53.63657539 | 2.64835423 | 0.537640214 | 4.92588568 | 8.40E-07 | 2.09E-05 |
| C1GALT1C1L | 31.70497453 | 1.91008413 | 0.387925231 | 4.92384608 | 8.49E-07 | 2.11E-05 |
| FABP4 | 3699.429767 | -1.563493434 | 0.317601989 | -4.9228074 | 8.53E-07 | 2.12E-05 |
| DLG5-AS1 | 38.42937889 | 1.773255814 | 0.360749597 | 4.91547553 | 8.86E-07 | 2.19E-05 |
| AP001453.2 | 74.34739346 | 1.394026892 | 0.283664576 | 4.91434958 | 8.91E-07 | 2.20E-05 |
| AC122719.3 | 90.0516794 | 2.504359741 | 0.51001365 | 4.91037787 | 9.09E-07 | 2.24E-05 |
| FERMT1 | 64.39837549 | 2.401591515 | 0.489078892 | 4.91043788 | 9.09E-07 | 2.24E-05 |
| TMEM231P1 | 44.13613128 | 2.038886411 | 0.415285611 | 4.90960042 | 9.13E-07 | 2.25E-05 |
| AC012313.8 | 93.24141152 | 1.187137178 | 0.241869431 | 4.90817369 | 9.19E-07 | 2.27E-05 |
| GKN2 | 261.9364819 | -1.766922419 | 0.360295173 | -4.9040968 | 9.39E-07 | 2.31E-05 |
| PKIA | 454.1715319 | -1.047127216 | 0.213581932 | -4.9026957 | 9.45E-07 | 2.32E-05 |
| ERICH5 | 281.758122 | 1.837703534 | 0.37517821 | 4.89821499 | 9.67E-07 | 2.37E-05 |
| DIO2 | 120.0314933 | 1.875400086 | 0.382922926 | 4.89759154 | 9.70E-07 | 2.38E-05 |
| PDE1C | 971.38971 | -1.301937762 | 0.265841833 | -4.8974149 | 9.71E-07 | 2.38E-05 |
| AC007743.1 | 502.8379392 | -1.447709354 | 0.295843456 | -4.893498 | 9.91E-07 | 2.43E-05 |
| AC008966.1 | 113.1655922 | 1.069120376 | 0.218627027 | 4.89015649 | 1.01E-06 | 2.46E-05 |
| AC023510.2 | 64.97244618 | 1.728464269 | 0.353489663 | 4.88971659 | 1.01E-06 | 2.46E-05 |
| WNT7B | 630.3342829 | 1.270658209 | 0.26004573 | 4.88628753 | 1.03E-06 | 2.50E-05 |
| SLC39A8 | 8124.870795 | -1.07113955 | 0.219489481 | -4.8801407 | 1.06E-06 | 2.56E-05 |
| ADGRF2 | 36.82494099 | 3.032073604 | 0.621393195 | 4.87947668 | 1.06E-06 | 2.57E-05 |
| RNF32 | 280.6845432 | 1.318360915 | 0.270349082 | 4.87651338 | 1.08E-06 | 2.61E-05 |
| ATP2B3 | 52.99590676 | 3.073087421 | 0.631438709 | 4.86680239 | 1.13E-06 | 2.73E-05 |
| TEX26 | 99.71655926 | 2.021528206 | 0.415498712 | 4.86530559 | 1.14E-06 | 2.75E-05 |
| AL365361.1 | 219.7935743 | -1.024806581 | 0.21065018 | -4.8649689 | 1.14E-06 | 2.75E-05 |
| ZNF273 | 806.0522431 | 1.695737415 | 0.348876736 | 4.86056317 | 1.17E-06 | 2.81E-05 |
| IP6K3 | 169.6372319 | -1.670405614 | 0.343713481 | -4.8598781 | 1.17E-06 | 2.82E-05 |
| AC233728.1 | 18.38831172 | 2.029695107 | 0.418008817 | 4.8556275 | 1.20E-06 | 2.88E-05 |
| GOLGA2P10 | 2453.370338 | 1.39109218 | 0.286586857 | 4.85399852 | 1.21E-06 | 2.90E-05 |
| AC025181.2 | 212.0812942 | 1.071929796 | 0.220942923 | 4.85161408 | 1.22E-06 | 2.93E-05 |
| CEMIP2 | 6123.043494 | -1.288146997 | 0.265671149 | -4.8486522 | 1.24E-06 | 2.97E-05 |
| AXDND1 | 64.70558948 | 2.502863534 | 0.516229158 | 4.84835755 | 1.24E-06 | 2.97E-05 |
| SH3GL3 | 199.657909 | -1.199387036 | 0.247442455 | -4.8471352 | 1.25E-06 | 2.99E-05 |
| FAM221B | 101.5201317 | 1.756982956 | 0.36265182 | 4.84482045 | 1.27E-06 | 3.02E-05 |
| RCAN3 | 1522.532254 | 1.037477065 | 0.214159781 | 4.84440665 | 1.27E-06 | 3.02E-05 |
| TMEM17 | 170.9083675 | 1.224506723 | 0.252791541 | 4.84393869 | 1.27E-06 | 3.02E-05 |
| PLEKHG4B | 1159.943124 | 1.189261514 | 0.245516706 | 4.84391279 | 1.27E-06 | 3.02E-05 |
| RFX2 | 3814.470405 | 1.274025566 | 0.263033121 | 4.84359369 | 1.28E-06 | 3.03E-05 |
| CT45A8 | 85.80926872 | -4.734664858 | 0.978511428 | -4.8386403 | 1.31E-06 | 3.09E-05 |
| CASP12 | 80.23329994 | -1.185958567 | 0.245122439 | -4.8382293 | 1.31E-06 | 3.10E-05 |
| CNGA3 | 115.2209993 | 2.39017544 | 0.49419577 | 4.83649514 | 1.32E-06 | 3.12E-05 |
| LRRC45 | 3602.208235 | 1.003243208 | 0.207479527 | 4.83538411 | 1.33E-06 | 3.14E-05 |
| CCDC68 | 2262.442991 | -1.212858842 | 0.250862207 | -4.8347611 | 1.33E-06 | 3.14E-05 |
| PCP4 | 28.0062879 | 3.895549981 | 0.806296169 | 4.83141323 | 1.36E-06 | 3.19E-05 |
| TBX3 | 3637.023179 | -1.106938226 | 0.229268643 | -4.8281274 | 1.38E-06 | 3.24E-05 |
| AL358113.1 | 85.33643247 | 1.537314931 | 0.318458886 | 4.82735762 | 1.38E-06 | 3.25E-05 |
| RGS9 | 1027.46147 | -1.353773704 | 0.280585624 | -4.8248149 | 1.40E-06 | 3.28E-05 |
| SMIM6 | 192.8044661 | 1.806063597 | 0.374315413 | 4.8249779 | 1.40E-06 | 3.28E-05 |
| SIGLEC11 | 200.9548701 | -1.280951309 | 0.265702551 | -4.8209974 | 1.43E-06 | 3.34E-05 |
| GOLGA2P7 | 6654.156986 | 1.121068355 | 0.232816905 | 4.81523606 | 1.47E-06 | 3.44E-05 |
| DACH2 | 83.32112875 | -1.670387857 | 0.34705749 | -4.8130005 | 1.49E-06 | 3.47E-05 |
| SLITRK6 | 1617.096717 | 1.363945128 | 0.283441629 | 4.8120847 | 1.49E-06 | 3.49E-05 |
| PITX1 | 34.15030486 | 3.138003288 | 0.652400411 | 4.80993457 | 1.51E-06 | 3.52E-05 |
| NCKAP5 | 2831.509715 | -1.27879584 | 0.266059657 | -4.8064252 | 1.54E-06 | 3.58E-05 |
| LINC02185 | 455.125946 | -1.589710746 | 0.330783263 | -4.8058984 | 1.54E-06 | 3.58E-05 |
| LINC01977 | 26.99367148 | 1.637031412 | 0.340729001 | 4.80449685 | 1.55E-06 | 3.60E-05 |
| AC087752.3 | 42.44191353 | 1.590426025 | 0.331114862 | 4.80324566 | 1.56E-06 | 3.61E-05 |
| COL4A2 | 69184.57126 | -1.105240485 | 0.230352015 | -4.79805 | 1.60E-06 | 3.69E-05 |
| MASP1 | 1152.278005 | -1.099139576 | 0.229123445 | -4.7971502 | 1.61E-06 | 3.70E-05 |
| OASL | 465.0811332 | -1.63485919 | 0.340789321 | -4.7972724 | 1.61E-06 | 3.70E-05 |
| ZNF396 | 249.722567 | 1.253448558 | 0.261282782 | 4.79728725 | 1.61E-06 | 3.70E-05 |
| GUCY1A2 | 1398.369211 | -1.381022839 | 0.287898459 | -4.7969094 | 1.61E-06 | 3.70E-05 |
| NOXRED1 | 46.9571285 | 1.639594878 | 0.341889665 | 4.79568424 | 1.62E-06 | 3.72E-05 |
| WWC2-AS2 | 125.1650332 | -1.208145955 | 0.252027481 | -4.7937072 | 1.64E-06 | 3.74E-05 |
| HS3ST6 | 40.76510043 | 2.946751007 | 0.614714949 | 4.79368691 | 1.64E-06 | 3.74E-05 |
| PRODH | 2744.529022 | -1.040827009 | 0.217170721 | -4.7926673 | 1.65E-06 | 3.76E-05 |
| SUSD4 | 441.7698839 | 1.242575162 | 0.25938259 | 4.79051105 | 1.66E-06 | 3.79E-05 |
| PRR29-AS1 | 107.9002312 | 1.756847812 | 0.366749548 | 4.79032032 | 1.67E-06 | 3.79E-05 |
| PRSS22 | 411.8008794 | 1.115807346 | 0.233026688 | 4.78832427 | 1.68E-06 | 3.83E-05 |
| COL12A1 | 8377.860153 | -1.236239435 | 0.258274445 | -4.7865341 | 1.70E-06 | 3.85E-05 |
| AL138828.1 | 83.48451186 | -2.229990322 | 0.465987639 | -4.7855139 | 1.71E-06 | 3.87E-05 |
| BCAS1 | 495.3115645 | 2.372653541 | 0.496004224 | 4.78353496 | 1.72E-06 | 3.90E-05 |
| AC006230.1 | 146.4060383 | 1.66470768 | 0.348020705 | 4.78335816 | 1.72E-06 | 3.90E-05 |
| FAM83A | 41.67321146 | 4.089456147 | 0.854988809 | 4.78305225 | 1.73E-06 | 3.90E-05 |
| SLFN13 | 2354.545421 | 1.281093047 | 0.267949855 | 4.78109251 | 1.74E-06 | 3.93E-05 |
| DTHD1 | 775.5300724 | 2.203158427 | 0.461035311 | 4.77871949 | 1.76E-06 | 3.97E-05 |
| COL9A2 | 2164.122545 | 1.161995195 | 0.243212758 | 4.77769013 | 1.77E-06 | 3.99E-05 |
| ST6GALNAC2 | 890.2942444 | 1.242157054 | 0.260012676 | 4.77729423 | 1.78E-06 | 3.99E-05 |
| AC138028.4 | 545.2877028 | -1.104891591 | 0.231373256 | -4.7753643 | 1.79E-06 | 4.02E-05 |
| MIR205 | 4.597187458 | 2.840730977 | 0.595301882 | 4.77191667 | 1.82E-06 | 4.09E-05 |
| TAL1 | 2333.633556 | -1.346432465 | 0.282380962 | -4.7681418 | 1.86E-06 | 4.16E-05 |
| C12orf75 | 1569.056893 | 1.872513571 | 0.392824056 | 4.76679965 | 1.87E-06 | 4.19E-05 |
| LINC02863 | 9.882507419 | -2.676169575 | 0.561534981 | -4.765811 | 1.88E-06 | 4.20E-05 |
| CATSPERE | 77.3128841 | 1.347798993 | 0.282893908 | 4.76432668 | 1.89E-06 | 4.23E-05 |
| FANCF | 409.026393 | 1.039617686 | 0.218228701 | 4.76389073 | 1.90E-06 | 4.24E-05 |
| RPS3AP16 | 9.696066657 | 2.671362559 | 0.561084293 | 4.76107172 | 1.93E-06 | 4.29E-05 |
| HEG1 | 8060.897626 | -1.236526977 | 0.259880145 | -4.7580664 | 1.95E-06 | 4.36E-05 |
| NME9 | 618.4318689 | 1.853695046 | 0.389703298 | 4.75668299 | 1.97E-06 | 4.38E-05 |
| AC010442.1 | 76.78189215 | 1.221571004 | 0.256976716 | 4.75362525 | 2.00E-06 | 4.45E-05 |
| PPOX | 1989.580358 | 1.082346716 | 0.227752594 | 4.7522915 | 2.01E-06 | 4.47E-05 |
| LIN7A | 612.0767486 | -1.317629839 | 0.277346724 | -4.7508398 | 2.03E-06 | 4.50E-05 |
| AC104809.1 | 632.7253245 | 2.257425914 | 0.475404656 | 4.74843038 | 2.05E-06 | 4.54E-05 |
| GPM6A | 1094.609669 | -1.418406247 | 0.298712335 | -4.748402 | 2.05E-06 | 4.54E-05 |
| OCA2 | 89.63415911 | 1.609903105 | 0.339048804 | 4.74829312 | 2.05E-06 | 4.54E-05 |
| LRRC56 | 527.0634245 | 1.6405962 | 0.345646864 | 4.74645186 | 2.07E-06 | 4.57E-05 |
| KLK12 | 52.97717793 | 2.653133232 | 0.559020525 | 4.74603903 | 2.07E-06 | 4.58E-05 |
| TNXB | 3461.157739 | -1.001189403 | 0.211002246 | -4.744923 | 2.09E-06 | 4.59E-05 |
| LHFPL3-AS2 | 407.4123556 | -1.281205086 | 0.270104691 | -4.7433648 | 2.10E-06 | 4.62E-05 |
| FAM47E | 642.6329652 | 1.474225334 | 0.310822096 | 4.74298755 | 2.11E-06 | 4.62E-05 |
| AL590822.3 | 689.4199146 | 1.607729135 | 0.339389307 | 4.73712371 | 2.17E-06 | 4.74E-05 |
| MZF1-AS1 | 1391.409819 | 1.029822674 | 0.217550506 | 4.73371768 | 2.20E-06 | 4.82E-05 |
| OVCH1 | 132.1898406 | -1.415217816 | 0.299177601 | -4.7303602 | 2.24E-06 | 4.89E-05 |
| ITGA1 | 6738.286111 | -1.156641727 | 0.244590457 | -4.7288915 | 2.26E-06 | 4.93E-05 |
| FZD3 | 211.524012 | 1.026357649 | 0.217098876 | 4.72760462 | 2.27E-06 | 4.95E-05 |
| PENK | 749.898527 | 1.521011901 | 0.321853677 | 4.72578693 | 2.29E-06 | 4.99E-05 |
| PARAL1 | 96.9913948 | -1.792202668 | 0.379244538 | -4.7257178 | 2.29E-06 | 4.99E-05 |
| CLDN18 | 20012.14268 | -1.624717985 | 0.343966481 | -4.7234776 | 2.32E-06 | 5.04E-05 |
| BIN2 | 2151.768603 | -1.07752344 | 0.228188391 | -4.7220783 | 2.33E-06 | 5.07E-05 |
| LINC01783 | 69.73170177 | 2.085926926 | 0.441793912 | 4.72149314 | 2.34E-06 | 5.08E-05 |
| AC062037.4 | 20.75082939 | -2.021675857 | 0.428710214 | -4.7157166 | 2.41E-06 | 5.21E-05 |
| CNTN6 | 454.3779168 | -1.08050379 | 0.229134833 | -4.7155807 | 2.41E-06 | 5.21E-05 |
| RMST | 62.06396758 | -2.07284086 | 0.439561308 | -4.7157036 | 2.41E-06 | 5.21E-05 |
| DUSP18 | 689.2534068 | 1.159238147 | 0.245841175 | 4.71539459 | 2.41E-06 | 5.21E-05 |
| MCEMP1 | 3426.858159 | -1.485507435 | 0.315072837 | -4.7148064 | 2.42E-06 | 5.22E-05 |
| AC244090.1 | 475.5875962 | 1.242974778 | 0.263657099 | 4.71436113 | 2.42E-06 | 5.23E-05 |
| PPM1E | 100.0536656 | 2.207958384 | 0.46852619 | 4.71256129 | 2.45E-06 | 5.27E-05 |
| ADCY2 | 149.9093814 | 1.283077249 | 0.272299443 | 4.71200835 | 2.45E-06 | 5.27E-05 |
| WFDC2 | 1431.575749 | 1.525968773 | 0.323829924 | 4.71225374 | 2.45E-06 | 5.27E-05 |
| SPRY4 | 6407.358113 | -1.401295884 | 0.297454209 | -4.7109634 | 2.47E-06 | 5.30E-05 |
| AC137834.1 | 8.657802119 | 4.651924904 | 0.988384564 | 4.70659405 | 2.52E-06 | 5.39E-05 |
| RALY-AS1 | 148.7607706 | 1.137340146 | 0.241841771 | 4.70282757 | 2.57E-06 | 5.48E-05 |
| CDKN2B | 1667.584126 | -1.231705555 | 0.262049847 | -4.700272 | 2.60E-06 | 5.54E-05 |
| DICER1-AS1 | 720.9571865 | 1.015921044 | 0.216217887 | 4.69859852 | 2.62E-06 | 5.58E-05 |
| PGC | 14696.41247 | -1.472046542 | 0.313347815 | -4.6978038 | 2.63E-06 | 5.60E-05 |
| AL390755.2 | 117.8345999 | 2.239416677 | 0.476820361 | 4.69656261 | 2.65E-06 | 5.63E-05 |
| AC016394.2 | 117.9518127 | 1.118521962 | 0.238242853 | 4.69488149 | 2.67E-06 | 5.67E-05 |
| ELL3 | 275.6239008 | 1.671632175 | 0.356231349 | 4.69254652 | 2.70E-06 | 5.72E-05 |
| AL121899.1 | 64.77559274 | 2.987732464 | 0.636726907 | 4.69232953 | 2.70E-06 | 5.72E-05 |
| CPEB1-AS1 | 55.06679404 | 2.333676278 | 0.497637455 | 4.68951092 | 2.74E-06 | 5.79E-05 |
| SOX9-AS1 | 37.45156336 | 1.889182137 | 0.403052226 | 4.68718944 | 2.77E-06 | 5.85E-05 |
| STARD8 | 4652.376397 | -1.152862622 | 0.246036961 | -4.6857294 | 2.79E-06 | 5.89E-05 |
| NTF3 | 159.0910367 | 1.201133917 | 0.256375523 | 4.68505691 | 2.80E-06 | 5.90E-05 |
| CCDC13-AS1 | 69.19724684 | 2.049586769 | 0.437657797 | 4.68308067 | 2.83E-06 | 5.95E-05 |
| BIRC7 | 33.29114366 | 3.209551389 | 0.685618711 | 4.68124825 | 2.85E-06 | 5.98E-05 |
| GALNT15 | 219.9234737 | 2.006016107 | 0.428594082 | 4.68045685 | 2.86E-06 | 6.00E-05 |
| KLRD1 | 817.1320356 | -1.685838253 | 0.360254246 | -4.6795791 | 2.87E-06 | 6.02E-05 |
| AL391807.1 | 270.5035214 | -1.100847763 | 0.235380328 | -4.6768894 | 2.91E-06 | 6.10E-05 |
| AC130456.2 | 144.903006 | 1.687646369 | 0.361294161 | 4.67111443 | 3.00E-06 | 6.26E-05 |
| CRYM | 249.7976008 | 1.479889403 | 0.316860381 | 4.67047788 | 3.00E-06 | 6.27E-05 |
| FAT2 | 58.30237291 | 2.184829652 | 0.468493687 | 4.66351994 | 3.11E-06 | 6.48E-05 |
| AC004982.1 | 176.7632356 | 1.272800009 | 0.272934778 | 4.66338522 | 3.11E-06 | 6.48E-05 |
| DMRTA1 | 104.8731231 | 1.162201427 | 0.249260885 | 4.66259047 | 3.12E-06 | 6.50E-05 |
| TRPV6 | 119.9412997 | 1.687350298 | 0.361923054 | 4.66217965 | 3.13E-06 | 6.50E-05 |
| AL713852.1 | 59.85504709 | 1.642456756 | 0.352536038 | 4.65897548 | 3.18E-06 | 6.60E-05 |
| PRSS12 | 3625.023521 | 1.386845018 | 0.297847275 | 4.65622866 | 3.22E-06 | 6.67E-05 |
| WDR35 | 1420.825684 | 1.037798684 | 0.222891302 | 4.65607528 | 3.22E-06 | 6.67E-05 |
| HS6ST2 | 105.5561478 | 2.04697065 | 0.439684553 | 4.6555437 | 3.23E-06 | 6.68E-05 |
| AL121768.1 | 22.66673514 | 2.530043622 | 0.543687641 | 4.65348746 | 3.26E-06 | 6.74E-05 |
| SERTAD4-AS1 | 219.4620808 | 1.052129362 | 0.226336177 | 4.64852494 | 3.34E-06 | 6.89E-05 |
| SALL4 | 51.72534842 | 1.667416404 | 0.358995315 | 4.64467455 | 3.41E-06 | 7.01E-05 |
| FOXN4 | 58.02742507 | 2.456074477 | 0.52883258 | 4.64433276 | 3.41E-06 | 7.02E-05 |
| AL121790.2 | 127.2231542 | 3.013232757 | 0.648851737 | 4.64394651 | 3.42E-06 | 7.03E-05 |
| KIAA0556 | 3327.07005 | 1.147880147 | 0.247241457 | 4.64274948 | 3.44E-06 | 7.07E-05 |
| AC109583.1 | 93.91439475 | 1.254642786 | 0.270286953 | 4.64189179 | 3.45E-06 | 7.09E-05 |
| GLDN | 569.333853 | -1.649218783 | 0.355473332 | -4.6395007 | 3.49E-06 | 7.15E-05 |
| AC007996.1 | 53.19352893 | 1.452422461 | 0.313266555 | 4.63637895 | 3.55E-06 | 7.23E-05 |
| SCNN1G | 1098.389436 | 1.107713318 | 0.239036604 | 4.63407402 | 3.59E-06 | 7.30E-05 |
| AC008915.3 | 37.31676556 | 1.730719994 | 0.37351162 | 4.63364431 | 3.59E-06 | 7.30E-05 |
| TPRG1 | 472.2409254 | -1.228131132 | 0.265158816 | -4.6316813 | 3.63E-06 | 7.37E-05 |
| DISP1 | 1709.724852 | -1.174092957 | 0.253828691 | -4.6255329 | 3.74E-06 | 7.54E-05 |
| GPR158 | 86.56902828 | -2.587895064 | 0.559746171 | -4.6233368 | 3.78E-06 | 7.62E-05 |
| CT75 | 54.94498833 | -2.369602779 | 0.512556274 | -4.6231076 | 3.78E-06 | 7.62E-05 |
| GDF15 | 901.64069 | 1.922016131 | 0.415768268 | 4.6228062 | 3.79E-06 | 7.63E-05 |
| EYA2 | 347.2088279 | 1.80508758 | 0.39055925 | 4.62180215 | 3.80E-06 | 7.65E-05 |
| SIGLEC5 | 251.7513026 | -1.154687777 | 0.24989004 | -4.6207835 | 3.82E-06 | 7.67E-05 |
| IL7R | 8769.318917 | -1.601718176 | 0.346958086 | -4.6164601 | 3.90E-06 | 7.81E-05 |
| AC010255.1 | 37.87847622 | 2.695862262 | 0.584437617 | 4.61274597 | 3.97E-06 | 7.95E-05 |
| CCL4 | 170.6411213 | -1.551960532 | 0.336587872 | -4.6108629 | 4.01E-06 | 8.02E-05 |
| GPR135 | 362.1274995 | 1.460999059 | 0.316891618 | 4.61040613 | 4.02E-06 | 8.03E-05 |
| BBS12 | 203.0254444 | 1.075564463 | 0.233343883 | 4.60935358 | 4.04E-06 | 8.06E-05 |
| MIR9-3HG | 71.93961256 | 1.322529812 | 0.286919907 | 4.60940416 | 4.04E-06 | 8.06E-05 |
| OSBPL3 | 2220.362498 | 1.077474446 | 0.23385588 | 4.60742935 | 4.08E-06 | 8.12E-05 |
| PILRA | 938.2598981 | -1.059662127 | 0.230032775 | -4.6065702 | 4.09E-06 | 8.13E-05 |
| HNF1A | 61.71419615 | 3.077150656 | 0.667931568 | 4.60698491 | 4.09E-06 | 8.13E-05 |
| AC018557.3 | 26.65126911 | 1.849962496 | 0.401588892 | 4.60660773 | 4.09E-06 | 8.13E-05 |
| PCYT1B | 553.1167011 | 1.382467018 | 0.300223716 | 4.60478951 | 4.13E-06 | 8.19E-05 |
| AC092802.2 | 171.5239534 | 1.885775092 | 0.409571108 | 4.60426787 | 4.14E-06 | 8.20E-05 |
| KLF7 | 4261.318238 | -1.031323966 | 0.224001516 | -4.6040937 | 4.14E-06 | 8.21E-05 |
| OVOL2 | 409.1139516 | 1.001699142 | 0.217600233 | 4.60339186 | 4.16E-06 | 8.23E-05 |
| SIRPB2 | 328.9131288 | -1.270573476 | 0.276032115 | -4.6029915 | 4.16E-06 | 8.24E-05 |
| AC105206.1 | 91.67193386 | -2.242672439 | 0.48787937 | -4.5967765 | 4.29E-06 | 8.45E-05 |
| SELPLG | 6349.468871 | -1.168368186 | 0.254256734 | -4.5952301 | 4.32E-06 | 8.51E-05 |
| FAM227B | 191.9230582 | 1.152804053 | 0.251000247 | 4.59284031 | 4.37E-06 | 8.60E-05 |
| SYT16 | 23.87177159 | 2.345821996 | 0.511143643 | 4.58935962 | 4.45E-06 | 8.73E-05 |
| DNAJC22 | 94.16044432 | 1.52574005 | 0.332498093 | 4.58871821 | 4.46E-06 | 8.75E-05 |
| AL390778.2 | 44.41407073 | 2.59392852 | 0.565313379 | 4.58847891 | 4.46E-06 | 8.75E-05 |
| B3GNT8 | 478.5311684 | -1.168548807 | 0.254673823 | -4.5884135 | 4.47E-06 | 8.75E-05 |
| SMPD2 | 848.527076 | 1.043912305 | 0.227764567 | 4.58329546 | 4.58E-06 | 8.95E-05 |
| AC092718.3 | 302.0128229 | 1.751513186 | 0.382409581 | 4.58020215 | 4.65E-06 | 9.08E-05 |
| C8orf34-AS1 | 259.560266 | 1.36021681 | 0.297342785 | 4.5745748 | 4.77E-06 | 9.31E-05 |
| RAB39B | 140.8770259 | -1.173004448 | 0.256449345 | -4.5740201 | 4.78E-06 | 9.33E-05 |
| APOBR | 6211.92761 | -1.064370435 | 0.232762564 | -4.5727733 | 4.81E-06 | 9.37E-05 |
| RAMP2-AS1 | 634.3579018 | -1.164407468 | 0.254698486 | -4.5717094 | 4.84E-06 | 9.40E-05 |
| LYPD5 | 143.9300744 | -1.020171765 | 0.223143215 | -4.5718252 | 4.83E-06 | 9.40E-05 |
| HK3 | 2582.75578 | -1.189172564 | 0.26043436 | -4.5661124 | 4.97E-06 | 9.63E-05 |
| AC073257.2 | 22.3049533 | 2.147456882 | 0.470623019 | 4.5630086 | 5.04E-06 | 9.76E-05 |
| ITPRIP | 4379.846947 | -1.19832542 | 0.262685613 | -4.5618236 | 5.07E-06 | 9.80E-05 |
| AC093928.1 | 181.8152308 | 2.072487347 | 0.45454742 | 4.55945245 | 5.13E-06 | 9.90E-05 |
| GAS2L1P2 | 8.87649318 | 4.489942912 | 0.985368242 | 4.55661419 | 5.20E-06 | 0.000100254 |
| HEPACAM2 | 30.04621021 | 2.735630032 | 0.600422784 | 4.55617292 | 5.21E-06 | 0.000100397 |
| AC090246.1 | 15.75580127 | 2.480007928 | 0.544524478 | 4.5544471 | 5.25E-06 | 0.000101157 |
| MAGIX | 548.9561262 | 1.117976373 | 0.245662443 | 4.55086402 | 5.34E-06 | 0.000102618 |
| EHF | 994.2928119 | 1.520500384 | 0.334255598 | 4.54891524 | 5.39E-06 | 0.000103503 |
| ZSCAN4 | 32.07830785 | 2.391980641 | 0.525862593 | 4.54867996 | 5.40E-06 | 0.000103549 |
| RPP38-DT | 55.3848253 | 1.602788417 | 0.352385111 | 4.54839994 | 5.41E-06 | 0.00010356 |
| CNTN5 | 53.90021696 | 2.323361264 | 0.510811314 | 4.54837471 | 5.41E-06 | 0.00010356 |
| HNRNPA1P33 | 32.96049585 | -1.924921997 | 0.423282535 | -4.5476055 | 5.43E-06 | 0.000103799 |
| AC103809.1 | 34.40199949 | 2.519283982 | 0.554624307 | 4.54232523 | 5.56E-06 | 0.000106079 |
| ADGRB2 | 350.1362747 | 1.121627457 | 0.246994133 | 4.54110971 | 5.60E-06 | 0.000106495 |
| DLL4 | 2920.84727 | -1.223757082 | 0.2694861 | -4.5410768 | 5.60E-06 | 0.000106495 |
| AC008549.1 | 4.794442444 | 5.908335504 | 1.301843581 | 4.53843733 | 5.67E-06 | 0.000107693 |
| AC019117.2 | 251.784635 | 1.393386115 | 0.307300907 | 4.53427271 | 5.78E-06 | 0.000109694 |
| C11orf21 | 747.5681341 | -1.434073122 | 0.316478662 | -4.5313422 | 5.86E-06 | 0.000111079 |
| RN7SL8P | 24.84960443 | -2.159294304 | 0.476686135 | -4.529803 | 5.90E-06 | 0.000111817 |
| RGMA | 712.8990091 | 1.215688026 | 0.268527513 | 4.52723824 | 5.98E-06 | 0.000113107 |
| HAP1 | 390.5678251 | -1.535860081 | 0.339553647 | -4.5231736 | 6.09E-06 | 0.000115225 |
| FP236315.2 | 4.990191251 | 2.798515612 | 0.618775635 | 4.52266614 | 6.11E-06 | 0.000115426 |
| TMC3-AS1 | 48.46956005 | 1.566819231 | 0.346467476 | 4.5222693 | 6.12E-06 | 0.000115566 |
| PLCXD3 | 320.0193408 | -1.868592859 | 0.413631194 | -4.5175337 | 6.26E-06 | 0.000117868 |
| SMIM34A | 31.53897507 | 2.175452176 | 0.481630336 | 4.5168504 | 6.28E-06 | 0.000118015 |
| PNMA8C | 120.996019 | 2.214556022 | 0.490691456 | 4.51313345 | 6.39E-06 | 0.000119866 |
| TMEM182 | 53.29121198 | 1.035902699 | 0.229560866 | 4.51254047 | 6.41E-06 | 0.000120122 |
| DLX4 | 113.3222122 | 1.578741828 | 0.350014245 | 4.51050736 | 6.47E-06 | 0.0001212 |
| WASF3 | 1348.217177 | -1.04352571 | 0.23144435 | -4.5087543 | 6.52E-06 | 0.000122045 |
| IGSF9B | 2046.07645 | 1.323382221 | 0.293989059 | 4.5014676 | 6.75E-06 | 0.000125646 |
| LRP2 | 228.1614129 | 1.658739086 | 0.368551658 | 4.5006963 | 6.77E-06 | 0.000125939 |
| AC084880.1 | 253.6583175 | -1.655230336 | 0.367816452 | -4.5001531 | 6.79E-06 | 0.000126179 |
| CHST6 | 425.3069778 | 1.637543339 | 0.363956231 | 4.49928645 | 6.82E-06 | 0.000126612 |
| NLRC4 | 299.9716006 | -1.034928523 | 0.230280882 | -4.4942008 | 6.98E-06 | 0.000129338 |
| AC087521.2 | 86.92951731 | 1.707544951 | 0.380023845 | 4.4932574 | 7.01E-06 | 0.000129744 |
| KCNK17 | 757.6974334 | -1.351409481 | 0.300782962 | -4.4929722 | 7.02E-06 | 0.00012975 |
| MMP11 | 55.92923976 | 1.467430603 | 0.326601432 | 4.49303175 | 7.02E-06 | 0.00012975 |
| AC012178.1 | 41.79272649 | 2.855646606 | 0.635829656 | 4.4912133 | 7.08E-06 | 0.000130488 |
| GPR87 | 28.06692601 | 3.199162546 | 0.712567137 | 4.48962965 | 7.13E-06 | 0.000131339 |
| CT45A7 | 124.4848229 | -4.616750394 | 1.028331566 | -4.4895543 | 7.14E-06 | 0.000131339 |
| AL512274.1 | 177.1957183 | 1.576041235 | 0.351100062 | 4.48886629 | 7.16E-06 | 0.000131594 |
| NRG3 | 142.5946936 | -1.775696175 | 0.395779691 | -4.4865773 | 7.24E-06 | 0.000132844 |
| EPAS1 | 96358.46534 | -1.232353644 | 0.274802204 | -4.4845115 | 7.31E-06 | 0.000133956 |
| C10orf95 | 649.8556993 | 1.85439654 | 0.413522765 | 4.48438804 | 7.31E-06 | 0.000133956 |
| RHEBL1 | 146.5379848 | 1.106025164 | 0.246743681 | 4.48248628 | 7.38E-06 | 0.000134983 |
| FLVCR2 | 1044.956187 | -1.026961367 | 0.229270823 | -4.4792502 | 7.49E-06 | 0.000136957 |
| AL590438.1 | 45.12314879 | 2.593740441 | 0.579404978 | 4.47655878 | 7.59E-06 | 0.000138441 |
| DAAM2-AS1 | 240.8400317 | -1.444252155 | 0.322626983 | -4.4765386 | 7.59E-06 | 0.000138441 |
| AP3B2 | 93.17142156 | 1.353092472 | 0.302322186 | 4.47566382 | 7.62E-06 | 0.000138921 |
| C1QA | 8743.222142 | -1.067035115 | 0.238445143 | -4.4749711 | 7.64E-06 | 0.000139194 |
| AC099050.1 | 43.95793522 | 2.397662401 | 0.535779271 | 4.47509363 | 7.64E-06 | 0.000139194 |
| WNT7A | 448.0056802 | -1.368162824 | 0.305866755 | -4.4730681 | 7.71E-06 | 0.00014017 |
| EMP2 | 40349.70074 | -1.061455587 | 0.237317322 | -4.472727 | 7.72E-06 | 0.000140305 |
| AC006058.1 | 515.66826 | 1.096508433 | 0.245243172 | 4.47110688 | 7.78E-06 | 0.000141282 |
| AL135960.1 | 472.4971362 | -1.32933945 | 0.297334909 | -4.4708489 | 7.79E-06 | 0.000141363 |
| CYP3A5 | 1201.354814 | -1.283119473 | 0.28716814 | -4.4681819 | 7.89E-06 | 0.000142864 |
| ACBD3-AS1 | 43.41977991 | 2.9320519 | 0.656323671 | 4.4673871 | 7.92E-06 | 0.000143123 |
| CAPN13 | 941.2014967 | 1.731897911 | 0.387662345 | 4.46754226 | 7.91E-06 | 0.000143123 |
| FBXL16 | 307.5671555 | 1.325913478 | 0.296833509 | 4.46685916 | 7.94E-06 | 0.000143385 |
| AC007681.1 | 110.3684329 | 1.609111044 | 0.360289323 | 4.46616355 | 7.96E-06 | 0.000143761 |
| P2RY14 | 472.998454 | -1.006704767 | 0.225470438 | -4.464908 | 8.01E-06 | 0.000144424 |
| NIPAL1 | 32.66009094 | 1.362396966 | 0.305490598 | 4.45970179 | 8.21E-06 | 0.000147603 |
| SPOCK2 | 21666.31375 | -1.346552418 | 0.301992124 | -4.4588991 | 8.24E-06 | 0.000148064 |
| ALOX12P2 | 104.248976 | 1.562282575 | 0.350748435 | 4.45413983 | 8.42E-06 | 0.000150816 |
| WDR90 | 3097.905102 | 1.043030284 | 0.23422632 | 4.45308745 | 8.46E-06 | 0.000151462 |
| SLC4A3 | 255.6813622 | 1.023289161 | 0.22994477 | 4.45015194 | 8.58E-06 | 0.000153361 |
| COL28A1 | 1293.438519 | 1.600987882 | 0.359920704 | 4.44816835 | 8.66E-06 | 0.000154681 |
| UNC13D | 5883.742587 | -1.003979166 | 0.225749043 | -4.4473241 | 8.69E-06 | 0.000155193 |
| FP671120.6 | 4.955784506 | 2.906574409 | 0.654016855 | 4.44418884 | 8.82E-06 | 0.000157156 |
| KRT5 | 533.6951143 | 2.994963883 | 0.674317068 | 4.4414772 | 8.93E-06 | 0.000158973 |
| CT45A2 | 80.72266864 | -4.218102029 | 0.950420517 | -4.4381429 | 9.07E-06 | 0.000161254 |
| GRM4 | 36.83063667 | 2.898845375 | 0.653650496 | 4.43485531 | 9.21E-06 | 0.000163429 |
| CCDC13 | 1240.239026 | 2.751469726 | 0.620530162 | 4.43406283 | 9.25E-06 | 0.00016393 |
| PROM1 | 558.1557166 | 2.969228983 | 0.670238971 | 4.43010495 | 9.42E-06 | 0.000166553 |
| LINC02604 | 674.3182392 | 1.210183718 | 0.273594007 | 4.42328299 | 9.72E-06 | 0.000171231 |
| AP001085.1 | 20.96166888 | 2.557682631 | 0.578243506 | 4.42319301 | 9.73E-06 | 0.000171231 |
| AP001189.1 | 224.0813671 | -2.323307381 | 0.525391678 | -4.4220483 | 9.78E-06 | 0.000172035 |
| KLRG2 | 192.4822189 | -1.387525353 | 0.31384374 | -4.4210707 | 9.82E-06 | 0.000172602 |
| SLC34A3 | 170.0438584 | 1.665392382 | 0.377052844 | 4.41686732 | 1.00E-05 | 0.000175343 |
| PKDCC | 3347.708095 | -1.377234211 | 0.311913408 | -4.4154377 | 1.01E-05 | 0.000176398 |
| NEXMIF | 34.65594805 | 1.306845107 | 0.296028067 | 4.41459866 | 1.01E-05 | 0.000176866 |
| ADGRL2 | 7986.960791 | -1.172125308 | 0.26558821 | -4.4133183 | 1.02E-05 | 0.000177698 |
| RAB27B | 257.8646544 | 1.016899929 | 0.230533685 | 4.41106873 | 1.03E-05 | 0.000179335 |
| AL359813.1 | 28.29704623 | -1.507912871 | 0.34190917 | -4.4102733 | 1.03E-05 | 0.000179775 |
| NT5DC3 | 526.1289745 | 1.034926794 | 0.234716189 | 4.40926891 | 1.04E-05 | 0.000180391 |
| ANO7L1 | 174.7972867 | 1.368476016 | 0.310779264 | 4.40336977 | 1.07E-05 | 0.000184918 |
| SLC44A5 | 35.64628147 | 2.246980632 | 0.510432325 | 4.40211272 | 1.07E-05 | 0.00018588 |
| SLC2A6 | 181.477004 | -1.04928677 | 0.238390681 | -4.4015427 | 1.07E-05 | 0.000186142 |
| MIR3945HG | 63.75180439 | -1.833972009 | 0.416843908 | -4.3996613 | 1.08E-05 | 0.000187649 |
| MAFG-DT | 183.3787093 | 1.432175558 | 0.325624702 | 4.3982399 | 1.09E-05 | 0.000188768 |
| AGAP12P | 402.3061229 | 1.60247863 | 0.364777429 | 4.3930312 | 1.12E-05 | 0.000192998 |
| AC131649.2 | 2099.276177 | -1.572798555 | 0.358040559 | -4.3927944 | 1.12E-05 | 0.000193092 |
| AC093159.1 | 26.44741038 | 2.825794737 | 0.643742728 | 4.38963365 | 1.14E-05 | 0.000195329 |
| AGTR1 | 513.8686527 | -1.097567327 | 0.250035355 | -4.3896485 | 1.14E-05 | 0.000195329 |
| SYNM | 3359.955931 | -1.096351356 | 0.249948185 | -4.3863145 | 1.15E-05 | 0.000197929 |
| FOXF1 | 2767.917608 | -1.111045105 | 0.253290803 | -4.3864408 | 1.15E-05 | 0.000197929 |
| REM2 | 186.8730559 | 1.406987002 | 0.320794223 | 4.38594869 | 1.15E-05 | 0.000198068 |
| RBBP8NL | 238.1398772 | 1.486748996 | 0.339015143 | 4.38549436 | 1.16E-05 | 0.000198363 |
| NT5E | 929.5713939 | 1.256380098 | 0.286513555 | 4.38506338 | 1.16E-05 | 0.000198632 |
| TTN | 1903.388725 | -1.12846186 | 0.257422352 | -4.383698 | 1.17E-05 | 0.000199646 |
| KCNA3 | 64.33689972 | -1.825493217 | 0.416472062 | -4.3832309 | 1.17E-05 | 0.000199955 |
| DNER | 115.1270148 | 2.225502449 | 0.508446175 | 4.37706597 | 1.20E-05 | 0.000205324 |
| ARMH1 | 610.570436 | 1.239822432 | 0.283404452 | 4.37474578 | 1.22E-05 | 0.000207272 |
| CYP27C1 | 143.7983196 | 1.326582469 | 0.303332215 | 4.37336493 | 1.22E-05 | 0.000208339 |
| AC010422.2 | 36.0150349 | 1.724792119 | 0.39487977 | 4.36789182 | 1.25E-05 | 0.000213245 |
| PTHLH | 62.56487115 | 1.168187574 | 0.26746735 | 4.36758945 | 1.26E-05 | 0.000213286 |
| LCA5 | 863.5174588 | 1.413854038 | 0.323807124 | 4.36634631 | 1.26E-05 | 0.000214376 |
| SVOPL | 40.24892842 | 1.888753612 | 0.43259772 | 4.3660739 | 1.26E-05 | 0.000214516 |
| ANKRD18A | 88.52602932 | 1.730271879 | 0.396810699 | 4.36044664 | 1.30E-05 | 0.000219585 |
| SLC22A10 | 59.66253702 | -1.529799986 | 0.350857571 | -4.3601738 | 1.30E-05 | 0.000219729 |
| PGM2L1 | 476.7392093 | 1.011755434 | 0.232077215 | 4.35956383 | 1.30E-05 | 0.000219951 |
| TTLL10-AS1 | 388.3556866 | 1.6567252 | 0.380096117 | 4.35870067 | 1.31E-05 | 0.000220429 |
| AC137630.1 | 45.29214116 | 2.166623607 | 0.49730044 | 4.35676994 | 1.32E-05 | 0.00022225 |
| Z97192.4 | 118.9041453 | -2.351420377 | 0.539742503 | -4.3565596 | 1.32E-05 | 0.000222333 |
| MUC4 | 415.0876432 | 2.864798165 | 0.657844811 | 4.35482368 | 1.33E-05 | 0.000223706 |
| IL12A | 97.48713166 | 1.477613927 | 0.339373587 | 4.35394498 | 1.34E-05 | 0.000224473 |
| CDC20B | 143.695183 | 2.157869652 | 0.495796453 | 4.35232975 | 1.35E-05 | 0.000225867 |
| GPA33 | 276.4212597 | -1.780237368 | 0.409255615 | -4.34994 | 1.36E-05 | 0.000227922 |
| AL136369.1 | 18.57907158 | -2.535968566 | 0.583004117 | -4.3498296 | 1.36E-05 | 0.000227922 |
| FAM83D | 155.1315992 | 1.699092414 | 0.390604527 | 4.34990456 | 1.36E-05 | 0.000227922 |
| AL009177.1 | 67.9927561 | 1.862874045 | 0.428510464 | 4.34732452 | 1.38E-05 | 0.000230405 |
| MAP7D2 | 209.0193035 | 1.704538604 | 0.392127459 | 4.34689937 | 1.38E-05 | 0.000230716 |
| AC073464.1 | 3.996073643 | 5.004849891 | 1.151458833 | 4.34652959 | 1.38E-05 | 0.00023097 |
| RNF157-AS1 | 160.1371199 | 2.021265794 | 0.465109715 | 4.34578279 | 1.39E-05 | 0.000231622 |
| SMAD6 | 5206.303327 | -1.626347509 | 0.374630883 | -4.3411998 | 1.42E-05 | 0.000236369 |
| KLK14 | 75.79018206 | 1.774191456 | 0.408764433 | 4.3403763 | 1.42E-05 | 0.000236979 |
| AC080005.1 | 5.156368329 | -5.281515658 | 1.217158595 | -4.3392173 | 1.43E-05 | 0.000238055 |
| RPLP0P2 | 111.477896 | -1.281180433 | 0.295262433 | -4.3391244 | 1.43E-05 | 0.000238055 |
| AC019117.3 | 78.70521734 | 3.667227029 | 0.845462078 | 4.33754171 | 1.44E-05 | 0.000239635 |
| SHISA6 | 54.00761418 | 1.629343991 | 0.375770346 | 4.33601004 | 1.45E-05 | 0.000241169 |
| SLC11A1 | 7272.470212 | -1.238618666 | 0.285839862 | -4.3332608 | 1.47E-05 | 0.000243776 |
| CT45A3 | 240.1879956 | -4.346500216 | 1.003212588 | -4.3325814 | 1.47E-05 | 0.000244388 |
| FIBCD1 | 96.46507263 | -1.117967762 | 0.258170084 | -4.3303536 | 1.49E-05 | 0.000246587 |
| TNNT3 | 196.8057633 | 1.592915913 | 0.367883634 | 4.32994503 | 1.49E-05 | 0.000246901 |
| AC243772.2 | 40.0010139 | 1.425962215 | 0.329406438 | 4.32888387 | 1.50E-05 | 0.00024795 |
| HSPH1 | 6099.938821 | 1.052588941 | 0.243239389 | 4.32737865 | 1.51E-05 | 0.000249505 |
| NDRG4 | 3300.495561 | -1.505970076 | 0.348022831 | -4.3272163 | 1.51E-05 | 0.000249544 |
| GJA5 | 3001.97424 | -1.367327051 | 0.316040954 | -4.3264236 | 1.52E-05 | 0.000250299 |
| TMEM61 | 129.0355468 | 2.015467009 | 0.466007355 | 4.32496824 | 1.53E-05 | 0.000251589 |
| MEFV | 269.4282069 | -1.419644443 | 0.328235134 | -4.3250838 | 1.52E-05 | 0.000251589 |
| TCP11X1 | 18.9883156 | 2.305136952 | 0.532991012 | 4.32490774 | 1.53E-05 | 0.000251589 |
| LINC02577 | 30.40596078 | -1.642699213 | 0.379950202 | -4.3234592 | 1.54E-05 | 0.000252809 |
| SLC2A3 | 5266.523746 | -1.341522764 | 0.310307291 | -4.3232074 | 1.54E-05 | 0.000252952 |
| NAPSA | 23604.3209 | -1.117931847 | 0.25875893 | -4.3203605 | 1.56E-05 | 0.00025609 |
| B4GALNT4 | 223.4237468 | 1.22569988 | 0.283715066 | 4.32017903 | 1.56E-05 | 0.000256153 |
| UCN3 | 56.96395638 | 3.175468552 | 0.735207157 | 4.31914804 | 1.57E-05 | 0.000256908 |
| MFSD2A | 6191.074273 | -1.08476761 | 0.25123151 | -4.3178008 | 1.58E-05 | 0.000258036 |
| CABP4 | 387.8192688 | -1.101234034 | 0.255204529 | -4.3151038 | 1.60E-05 | 0.000260907 |
| AC020763.1 | 23.50728175 | 2.579998887 | 0.598632235 | 4.30982286 | 1.63E-05 | 0.000266299 |
| COL26A1 | 202.5399183 | -1.090034477 | 0.25293773 | -4.3094973 | 1.64E-05 | 0.000266539 |
| LNCTAM34A | 78.72537407 | 1.330004763 | 0.308693261 | 4.30849951 | 1.64E-05 | 0.000267462 |
| MIPOL1 | 610.9887969 | 1.237961669 | 0.287331407 | 4.30848017 | 1.64E-05 | 0.000267462 |
| RBKS | 211.9924147 | 1.482194199 | 0.344033738 | 4.30828153 | 1.65E-05 | 0.00026755 |
| CU634019.4 | 5.0226285 | 2.616638076 | 0.607953886 | 4.30400749 | 1.68E-05 | 0.000272456 |
| SLC22A8 | 6.120384842 | -5.5318825 | 1.285612174 | -4.302917 | 1.69E-05 | 0.000273645 |
| DNAAF2 | 899.0977411 | 1.053295051 | 0.244994494 | 4.2992601 | 1.71E-05 | 0.000278041 |
| FGR | 2787.195699 | -1.014198292 | 0.236062885 | -4.2963056 | 1.74E-05 | 0.000281451 |
| AL606760.1 | 39.59335073 | 1.30408679 | 0.303579926 | 4.29569507 | 1.74E-05 | 0.000282066 |
| FAM86HP | 109.6373986 | 1.11379052 | 0.259491592 | 4.29220273 | 1.77E-05 | 0.000286054 |
| TMEM150B | 228.8660654 | -1.055220523 | 0.245887877 | -4.2914703 | 1.77E-05 | 0.000286557 |
| AC005083.1 | 51.26238032 | 1.052954999 | 0.245511479 | 4.28882186 | 1.80E-05 | 0.000289457 |
| GRIA1 | 1708.714625 | -1.050926523 | 0.245203319 | -4.2859392 | 1.82E-05 | 0.000292411 |
| TRIM2 | 1462.980295 | 1.037903586 | 0.242216788 | 4.28501919 | 1.83E-05 | 0.000293458 |
| HECW2 | 2916.257522 | -1.429995053 | 0.333979649 | -4.2816832 | 1.85E-05 | 0.000297391 |
| AP000866.2 | 135.1579674 | -1.118103658 | 0.261235814 | -4.280055 | 1.87E-05 | 0.000299407 |
| SPDEF | 91.99931049 | 2.368890051 | 0.553686972 | 4.27839226 | 1.88E-05 | 0.000300975 |
| GBP4 | 6169.540787 | -1.341805326 | 0.313648369 | -4.2780561 | 1.89E-05 | 0.000301261 |
| ARC | 1031.725468 | -1.695604881 | 0.396422551 | -4.2772665 | 1.89E-05 | 0.000302162 |
| ADAMTS7P3 | 3278.988758 | -1.381030568 | 0.323048125 | -4.2749995 | 1.91E-05 | 0.000304571 |
| C3orf36 | 176.3003615 | -1.919102318 | 0.449177877 | -4.2724774 | 1.93E-05 | 0.000307351 |
| KIF3A | 1544.838299 | 1.011599174 | 0.23689305 | 4.27027797 | 1.95E-05 | 0.000310052 |
| IL1A | 69.29322751 | -2.45999502 | 0.576336589 | -4.2683305 | 1.97E-05 | 0.000312249 |
| FAM27C | 191.9073251 | 1.373271508 | 0.321781277 | 4.26771726 | 1.97E-05 | 0.000312934 |
| AC044860.1 | 764.6702136 | 1.15445537 | 0.2705324 | 4.26734606 | 1.98E-05 | 0.000313281 |
| ADCY8 | 346.2779094 | -1.609846919 | 0.377331557 | -4.2663989 | 1.99E-05 | 0.000314439 |
| TBX21 | 446.7908123 | -1.467303758 | 0.344131616 | -4.2637866 | 2.01E-05 | 0.000317611 |
| AC099521.4 | 45.23003719 | 2.187457269 | 0.513092389 | 4.26328146 | 2.01E-05 | 0.000317815 |
| AC015802.7 | 29.00341724 | 2.322689708 | 0.544811119 | 4.26329351 | 2.01E-05 | 0.000317815 |
| TPH1 | 106.3770041 | 1.127634916 | 0.264971081 | 4.25569052 | 2.08E-05 | 0.000328054 |
| AC012313.6 | 183.1461559 | 1.342188083 | 0.315992242 | 4.24753492 | 2.16E-05 | 0.000338355 |
| AC000050.3 | 99.55971692 | -1.497548792 | 0.352635934 | -4.2467277 | 2.17E-05 | 0.000339204 |
| CYP2A13 | 34.47602414 | 2.980333061 | 0.702685529 | 4.24134686 | 2.22E-05 | 0.00034706 |
| ERBB4 | 199.0604074 | 1.535145959 | 0.361976393 | 4.24101127 | 2.23E-05 | 0.000347389 |
| FBXW10 | 122.3196761 | 2.289432332 | 0.539944709 | 4.24012365 | 2.23E-05 | 0.000348575 |
| P2RX7 | 584.2631522 | -1.051346982 | 0.248029245 | -4.2388025 | 2.25E-05 | 0.000350249 |
| ABCA13 | 587.1427926 | 2.442461004 | 0.576256293 | 4.23849775 | 2.25E-05 | 0.000350342 |
| SDR42E2 | 75.95852084 | 1.811870942 | 0.427477117 | 4.23852147 | 2.25E-05 | 0.000350342 |
| SPOCK1 | 63.57332121 | 1.534234844 | 0.362019845 | 4.23798548 | 2.26E-05 | 0.00035095 |
| C2orf15 | 102.5712864 | 1.33647869 | 0.315504427 | 4.23600614 | 2.28E-05 | 0.000352901 |
| SLCO2A1 | 8296.132422 | -1.426427752 | 0.337009869 | -4.2325994 | 2.31E-05 | 0.000357514 |
| COX4I2 | 1873.709496 | -1.184329618 | 0.280053219 | -4.2289449 | 2.35E-05 | 0.000363173 |
| ADGRE3 | 114.103335 | -1.937178721 | 0.458425402 | -4.2257229 | 2.38E-05 | 0.000367612 |
| PTPRB | 9424.001603 | -1.166669894 | 0.276154454 | -4.2247006 | 2.39E-05 | 0.000368885 |
| LYPD6B | 229.9465472 | 1.108837191 | 0.262492102 | 4.22426877 | 2.40E-05 | 0.000369345 |
| CCDC125 | 492.4197733 | 1.054801401 | 0.249705785 | 4.22417687 | 2.40E-05 | 0.000369345 |
| DNAH1 | 4859.542682 | 1.309803045 | 0.310097546 | 4.22384201 | 2.40E-05 | 0.000369694 |
| LINC02062 | 95.38346482 | 1.131184972 | 0.267836629 | 4.22341402 | 2.41E-05 | 0.000370032 |
| ABCD2 | 73.21784173 | 1.754325237 | 0.415390704 | 4.22331367 | 2.41E-05 | 0.000370032 |
| LINC00598 | 80.01031663 | 1.289440391 | 0.305317843 | 4.22327231 | 2.41E-05 | 0.000370032 |
| HSD11B1 | 594.136574 | -1.171807624 | 0.277506871 | -4.2226256 | 2.41E-05 | 0.000370895 |
| BMP15 | 3.354649485 | 5.158644788 | 1.221767813 | 4.22227917 | 2.42E-05 | 0.000371266 |
| AC009093.9 | 13.60974585 | -3.392887186 | 0.803707944 | -4.2215424 | 2.43E-05 | 0.000372282 |
| IL17D | 188.4664561 | -1.250803142 | 0.29636994 | -4.2204116 | 2.44E-05 | 0.000373812 |
| ASRGL1 | 812.4089886 | -1.042464037 | 0.247172816 | -4.2175513 | 2.47E-05 | 0.000377424 |
| BAAT | 20.41765412 | 3.351566176 | 0.79489826 | 4.2163461 | 2.48E-05 | 0.000378922 |
| RAMP3 | 4144.013472 | -1.466702449 | 0.347938592 | -4.2154061 | 2.49E-05 | 0.0003803 |
| CILP2 | 64.18901802 | 1.958672259 | 0.465104283 | 4.21125397 | 2.54E-05 | 0.000386739 |
| SFTPC | 921986.0217 | -1.102276893 | 0.261874858 | -4.2091742 | 2.56E-05 | 0.000389691 |
| IL1B | 263.4065297 | -1.706144613 | 0.40543965 | -4.2081346 | 2.57E-05 | 0.000391279 |
| BDH1 | 381.1497766 | 1.053427957 | 0.250346127 | 4.20788598 | 2.58E-05 | 0.000391502 |
| IRX5 | 1416.047194 | 1.021852239 | 0.242973766 | 4.20560727 | 2.60E-05 | 0.000394639 |
| SNX18P3 | 29.84662564 | 2.293061443 | 0.54534773 | 4.20476939 | 2.61E-05 | 0.000395881 |
| RET | 61.31605349 | 1.35893074 | 0.323209317 | 4.20449124 | 2.62E-05 | 0.000396158 |
| ANKRD44 | 2302.616773 | -1.031335645 | 0.245369289 | -4.2031978 | 2.63E-05 | 0.000398218 |
| ACVRL1 | 10251.39723 | -1.271754995 | 0.302780352 | -4.200256 | 2.67E-05 | 0.000402148 |
| ADGRF4 | 45.60679897 | 2.714368199 | 0.646329237 | 4.19966798 | 2.67E-05 | 0.00040298 |
| PTCH2 | 308.2041717 | 1.235563755 | 0.294328019 | 4.19791414 | 2.69E-05 | 0.000405256 |
| EHMT2-AS1 | 21.80197118 | 1.142254566 | 0.272189815 | 4.19653677 | 2.71E-05 | 0.000407513 |
| LGI3 | 1428.843303 | -1.287989732 | 0.307188672 | -4.1928295 | 2.75E-05 | 0.000413578 |
| FAM27B | 92.37009938 | 1.542398805 | 0.367884973 | 4.1926116 | 2.76E-05 | 0.000413758 |
| NPTX1 | 179.348104 | 1.458602731 | 0.34791229 | 4.19244382 | 2.76E-05 | 0.000413847 |
| NPR1 | 5369.154015 | -1.104930608 | 0.263839159 | -4.1878947 | 2.82E-05 | 0.0004209 |
| MYO1G | 3018.559893 | -1.074456873 | 0.256586975 | -4.1874958 | 2.82E-05 | 0.000421419 |
| ZPLD2P | 50.62240635 | 1.629128563 | 0.389255156 | 4.18524595 | 2.85E-05 | 0.000424947 |
| SCGB3A2 | 28300.20258 | 2.214945734 | 0.529570236 | 4.18253441 | 2.88E-05 | 0.000429375 |
| FCGR3A | 5363.402463 | -1.024413264 | 0.244937469 | -4.1823461 | 2.89E-05 | 0.000429507 |
| AC007114.1 | 171.1854523 | 1.162149529 | 0.277906789 | 4.1817961 | 2.89E-05 | 0.000430111 |
| TMEM190 | 1905.214751 | 2.394715708 | 0.572757462 | 4.18102926 | 2.90E-05 | 0.000431327 |
| CYP4F32P | 11.97947024 | 3.156895778 | 0.755269274 | 4.17982816 | 2.92E-05 | 0.00043316 |
| CFC1 | 63.61699347 | 2.553147306 | 0.611513301 | 4.17512964 | 2.98E-05 | 0.000441508 |
| SH2D4B | 60.24268704 | -1.543541984 | 0.36982915 | -4.1736623 | 3.00E-05 | 0.000443482 |
| AL035425.4 | 35.56617815 | 2.187933461 | 0.524204266 | 4.1738185 | 3.00E-05 | 0.000443482 |
| SASH1 | 5848.363532 | -1.027627907 | 0.246265517 | -4.1728453 | 3.01E-05 | 0.000444343 |
| LINC02299 | 51.61141639 | 2.724702732 | 0.65322438 | 4.17115897 | 3.03E-05 | 0.000447182 |
| GRIK2 | 50.05785509 | 2.092823159 | 0.502507479 | 4.16476022 | 3.12E-05 | 0.000458723 |
| CXCR2 | 221.977056 | -2.320072859 | 0.557288443 | -4.1631455 | 3.14E-05 | 0.000461266 |
| KIAA0895 | 287.6252108 | 1.044509254 | 0.251204838 | 4.15799816 | 3.21E-05 | 0.000471053 |
| CLDN5 | 19183.32937 | -1.1644985 | 0.280221241 | -4.1556397 | 3.24E-05 | 0.000474707 |
| TLR8 | 217.9862467 | -1.375641472 | 0.331038923 | -4.1555279 | 3.25E-05 | 0.000474707 |
| CIT | 1754.591498 | 1.183574327 | 0.284929416 | 4.15392115 | 3.27E-05 | 0.000477461 |
| SEC14L3 | 551.2616632 | 1.780389318 | 0.428826015 | 4.15177545 | 3.30E-05 | 0.000481571 |
| C9orf163 | 61.03348138 | 2.265587609 | 0.545744984 | 4.15136681 | 3.30E-05 | 0.000481999 |
| PDZRN4 | 92.20806766 | -2.119342548 | 0.510758093 | -4.1494057 | 3.33E-05 | 0.000485342 |
| SLC16A12 | 193.7108037 | 1.574776521 | 0.379624614 | 4.14824662 | 3.35E-05 | 0.000487059 |
| NLRP12 | 69.68520779 | -1.704671922 | 0.411115579 | -4.1464542 | 3.38E-05 | 0.000490584 |
| FBXO2 | 409.6997639 | 1.255646476 | 0.302945707 | 4.14479046 | 3.40E-05 | 0.000493242 |
| CHST5 | 93.1321925 | 2.127187181 | 0.513203523 | 4.14491929 | 3.40E-05 | 0.000493242 |
| ADRA1D | 352.2534185 | -1.360989966 | 0.328682741 | -4.1407406 | 3.46E-05 | 0.000500978 |
| HIPK1-AS1 | 35.19430487 | 1.175165203 | 0.283967078 | 4.13838537 | 3.50E-05 | 0.000505891 |
| GALNT5 | 1470.92092 | -1.080499661 | 0.26124303 | -4.1359942 | 3.53E-05 | 0.000510626 |
| CSF2RB | 1337.85608 | -1.123184577 | 0.271644135 | -4.1347647 | 3.55E-05 | 0.000512633 |
| PLEKHS1 | 1117.592014 | 2.720509479 | 0.658049741 | 4.13420037 | 3.56E-05 | 0.000513634 |
| TGFBR3 | 4316.122064 | -1.00501761 | 0.243358654 | -4.1297796 | 3.63E-05 | 0.000521501 |
| SEMA6A | 7204.475765 | -1.294951157 | 0.31355602 | -4.1298877 | 3.63E-05 | 0.000521501 |
| AL161772.1 | 328.4419553 | -1.055189969 | 0.255500786 | -4.1298893 | 3.63E-05 | 0.000521501 |
| GATA2-AS1 | 869.8411974 | -1.249115325 | 0.302518276 | -4.1290574 | 3.64E-05 | 0.000522879 |
| AL096706.1 | 11.22301124 | -4.578130926 | 1.108801001 | -4.1289022 | 3.64E-05 | 0.000522969 |
| MSR1 | 3763.495262 | -1.156600661 | 0.280202154 | -4.1277365 | 3.66E-05 | 0.000525362 |
| AC078795.1 | 98.03372737 | 1.342056761 | 0.325199978 | 4.12686608 | 3.68E-05 | 0.000526824 |
| LRRC61 | 402.343542 | 1.077568237 | 0.261104127 | 4.12696747 | 3.68E-05 | 0.000526824 |
| SNORA7B | 23.47235087 | 1.546308155 | 0.374866319 | 4.12495889 | 3.71E-05 | 0.000530675 |
| NOS2 | 333.7440384 | -1.293558999 | 0.313631095 | -4.1244603 | 3.72E-05 | 0.000531559 |
| MATN3 | 711.89295 | -1.211540246 | 0.293764894 | -4.1241832 | 3.72E-05 | 0.000531933 |
| PADI4 | 68.00726167 | -2.98397213 | 0.724714594 | -4.1174445 | 3.83E-05 | 0.000545535 |
| MYOZ1 | 282.3548373 | -1.427769713 | 0.346792342 | -4.1170739 | 3.84E-05 | 0.00054614 |
| LINC01018 | 56.8968122 | 1.120727203 | 0.272246132 | 4.1165955 | 3.85E-05 | 0.000547002 |
| Z95114.3 | 161.870776 | -1.174838746 | 0.285489443 | -4.115174 | 3.87E-05 | 0.000550047 |
| KIF27 | 929.1349037 | 1.13107755 | 0.274914747 | 4.11428474 | 3.88E-05 | 0.000551409 |
| FUT2 | 123.8286597 | 1.508096884 | 0.366678103 | 4.11286322 | 3.91E-05 | 0.000554265 |
| C1QTNF8 | 69.02787529 | 1.908817696 | 0.464255629 | 4.11156608 | 3.93E-05 | 0.000557112 |
| HSPD1P21 | 32.8548115 | 2.348760905 | 0.571680205 | 4.10852236 | 3.98E-05 | 0.000563943 |
| MCOLN2 | 338.8535278 | -1.06331653 | 0.258903612 | -4.1069977 | 4.01E-05 | 0.000567114 |
| CFAP206 | 676.2364444 | 2.663426658 | 0.648793905 | 4.1051968 | 4.04E-05 | 0.000571267 |
| PTPRQ | 408.2297193 | -1.183392782 | 0.288352666 | -4.1039772 | 4.06E-05 | 0.000574004 |
| ABCC13 | 54.28968553 | -1.322512684 | 0.322392635 | -4.1021802 | 4.09E-05 | 0.000578195 |
| LY6H | 48.00862666 | 2.149524209 | 0.524027114 | 4.10193319 | 4.10E-05 | 0.000578526 |
| S100A12 | 70.61990652 | -2.984937054 | 0.72831801 | -4.098398 | 4.16E-05 | 0.00058678 |
| CPNE7 | 125.1413944 | 1.631472676 | 0.398608002 | 4.09292505 | 4.26E-05 | 0.000599103 |
| AC010273.3 | 26.81700093 | 1.60412038 | 0.391951844 | 4.09264659 | 4.26E-05 | 0.000599528 |
| FAM181A-AS1 | 26.6266045 | 2.290126362 | 0.559632248 | 4.09219871 | 4.27E-05 | 0.000600096 |
| ADH1C | 528.5891223 | 1.087614114 | 0.265860195 | 4.09092498 | 4.30E-05 | 0.000602511 |
| CT45A9 | 85.96917843 | -3.868777111 | 0.946075957 | -4.0892881 | 4.33E-05 | 0.000606481 |
| NOS1 | 480.8590648 | -1.687324414 | 0.413138839 | -4.0841583 | 4.42E-05 | 0.000618045 |
| LINC00643 | 27.29336505 | 2.398862351 | 0.58741703 | 4.08374669 | 4.43E-05 | 0.000618701 |
| AC093635.1 | 38.75050293 | 1.831365217 | 0.448874488 | 4.07990488 | 4.51E-05 | 0.000627174 |
| AC113349.1 | 138.8490375 | 2.193193595 | 0.537671262 | 4.07906048 | 4.52E-05 | 0.000629149 |
| PLEKHH2 | 9879.571059 | -1.019698272 | 0.250044251 | -4.0780712 | 4.54E-05 | 0.000631014 |
| TNNC1 | 4023.149171 | -1.291837834 | 0.316782706 | -4.0779936 | 4.54E-05 | 0.000631014 |
| PTP4A1P2 | 5.555942015 | 4.021181978 | 0.986114219 | 4.07780549 | 4.55E-05 | 0.000631014 |
| ZEB2-AS1 | 139.4353606 | -1.002354623 | 0.245887143 | -4.0764825 | 4.57E-05 | 0.000634305 |
| MOXD1 | 1238.851864 | 1.137911546 | 0.279259509 | 4.07474593 | 4.61E-05 | 0.000638746 |
| CFAP298-TCP10L | 755.5607073 | 1.278587675 | 0.313877817 | 4.07352035 | 4.63E-05 | 0.000641806 |
| CACNA1G | 158.9072975 | 1.508491926 | 0.370413057 | 4.07245883 | 4.65E-05 | 0.000644113 |
| AC091182.1 | 95.84126769 | -1.391015125 | 0.341648222 | -4.0714836 | 4.67E-05 | 0.000645913 |
| SLC6A16 | 618.4658309 | 1.001547673 | 0.245991638 | 4.0714704 | 4.67E-05 | 0.000645913 |
| XDH | 113.8118799 | 1.834655971 | 0.451194898 | 4.06621613 | 4.78E-05 | 0.000658408 |
| ALOX15B | 4161.558723 | -1.290581098 | 0.317697208 | -4.0622992 | 4.86E-05 | 0.000668588 |
| SAMD14 | 945.3706327 | -1.015105021 | 0.249982285 | -4.0607078 | 4.89E-05 | 0.000672513 |
| ROBO4 | 10448.7065 | -1.106049052 | 0.272422558 | -4.0600494 | 4.91E-05 | 0.000674087 |
| ERP27 | 1716.956719 | 1.341868353 | 0.330525217 | 4.05980628 | 4.91E-05 | 0.000674465 |
| SECTM1 | 1584.070136 | -1.131339522 | 0.278794977 | -4.0579624 | 4.95E-05 | 0.000679157 |
| SERPINB5 | 12.22919902 | 4.95416242 | 1.220845788 | 4.0579756 | 4.95E-05 | 0.000679157 |
| CT45A6 | 109.5124583 | -4.227359083 | 1.042513173 | -4.0549695 | 5.01E-05 | 0.00068692 |
| AC007877.1 | 80.08004266 | -1.261423054 | 0.311111159 | -4.0545735 | 5.02E-05 | 0.000687754 |
| GCOM1 | 1314.65802 | -1.319097402 | 0.325544702 | -4.0519701 | 5.08E-05 | 0.000694787 |
| AC242022.2 | 19.44889757 | 2.160439233 | 0.533401426 | 4.05030645 | 5.12E-05 | 0.00069941 |
| C16orf46 | 132.5546353 | 1.362865045 | 0.33673508 | 4.04729155 | 5.18E-05 | 0.00070712 |
| LINC01513 | 58.44060512 | 2.05425305 | 0.507593182 | 4.04704618 | 5.19E-05 | 0.000707523 |
| RNU5B-3P | 6.553557351 | -2.674820969 | 0.661003698 | -4.0466052 | 5.20E-05 | 0.00070818 |
| ADARB2 | 319.2705249 | -1.518302526 | 0.375487704 | -4.0435479 | 5.26E-05 | 0.000715772 |
| CSRP3 | 4.300470642 | 5.53870128 | 1.370394743 | 4.04168311 | 5.31E-05 | 0.000720116 |
| SULT1A2 | 1145.343809 | -1.067297589 | 0.264125372 | -4.0408749 | 5.33E-05 | 0.000722259 |
| ADRB2 | 931.7849987 | -1.084133913 | 0.268396799 | -4.0392952 | 5.36E-05 | 0.000725416 |
| BMP6 | 2313.397253 | -1.341533231 | 0.332291365 | -4.0372197 | 5.41E-05 | 0.000730821 |
| AC092171.5 | 54.39716996 | 1.588436127 | 0.393762544 | 4.03399499 | 5.48E-05 | 0.000740228 |
| TMC5 | 2232.198616 | 1.001605763 | 0.248316334 | 4.0335879 | 5.49E-05 | 0.000741161 |
| TSPAN19 | 353.3121407 | 2.50750676 | 0.621729045 | 4.03311826 | 5.50E-05 | 0.000742293 |
| AC004593.1 | 31.04377918 | 2.038038688 | 0.505480841 | 4.03188117 | 5.53E-05 | 0.000745155 |
| HYDIN2 | 481.9486653 | 1.575878877 | 0.391575534 | 4.02445695 | 5.71E-05 | 0.000766162 |
| SH2D1B | 107.6707069 | -1.497453339 | 0.372108915 | -4.024234 | 5.72E-05 | 0.000766528 |
| AL035420.3 | 7.173212158 | 3.078965378 | 0.765728839 | 4.02096045 | 5.80E-05 | 0.000776549 |
| PCDH19 | 65.31792628 | 1.227250498 | 0.305606123 | 4.01579159 | 5.92E-05 | 0.000792632 |
| LILRA6 | 98.03502323 | -1.202639506 | 0.299693538 | -4.0128977 | 6.00E-05 | 0.000800605 |
| LINC01132 | 62.80245971 | 1.783744674 | 0.444800125 | 4.01021621 | 6.07E-05 | 0.000808549 |
| ZNF296 | 190.4909866 | 1.075874012 | 0.268349388 | 4.00922849 | 6.09E-05 | 0.00081118 |
| H19 | 7788.332632 | 1.456033698 | 0.363376129 | 4.00696023 | 6.15E-05 | 0.000818243 |
| BHLHA15 | 39.51934591 | 1.55553663 | 0.3882248 | 4.00679356 | 6.15E-05 | 0.000818439 |
| JCAD | 11106.10575 | -1.17648119 | 0.293636137 | -4.0065954 | 6.16E-05 | 0.000818744 |
| ALOX5AP | 2348.042794 | -1.133044394 | 0.282955762 | -4.0043164 | 6.22E-05 | 0.000824755 |
| MUC20 | 191.2165046 | 1.249194254 | 0.312127014 | 4.00219845 | 6.28E-05 | 0.000831401 |
| AC004130.2 | 35.37564246 | 1.725267536 | 0.431252238 | 4.00059961 | 6.32E-05 | 0.000836262 |
| PRF1 | 2204.940622 | -1.452140087 | 0.363313151 | -3.9969379 | 6.42E-05 | 0.000847333 |
| SMC2-AS1 | 56.32872458 | 1.747195113 | 0.437384247 | 3.99464572 | 6.48E-05 | 0.000853991 |
| AL033519.4 | 70.72945936 | -1.878451823 | 0.47036214 | -3.9936289 | 6.51E-05 | 0.000857267 |
| ARHGEF10 | 1826.203947 | -1.011250051 | 0.25338162 | -3.9910158 | 6.58E-05 | 0.000865569 |
| AC104984.2 | 59.96839115 | -2.658015717 | 0.666254945 | -3.9894874 | 6.62E-05 | 0.000870362 |
| SFN | 520.2248996 | 1.663239816 | 0.416976933 | 3.98880533 | 6.64E-05 | 0.000872064 |
| LINC00920 | 129.8223809 | -1.415102518 | 0.354955179 | -3.9867076 | 6.70E-05 | 0.000878998 |
| HYAL1 | 1616.059974 | -1.251135538 | 0.313879507 | -3.9860377 | 6.72E-05 | 0.000881077 |
| LINC02265 | 42.62469483 | 2.531354988 | 0.635835084 | 3.98115023 | 6.86E-05 | 0.000897331 |
| SLC19A3 | 923.6280719 | -1.207821857 | 0.303433479 | -3.9805161 | 6.88E-05 | 0.000898903 |
| CT45A5 | 125.366668 | -4.346381095 | 1.092153829 | -3.9796419 | 6.90E-05 | 0.000901802 |
| ABCA3 | 22246.21743 | -1.005830311 | 0.252784584 | -3.9790018 | 6.92E-05 | 0.000903405 |
| AC026412.3 | 42.09624539 | 1.047464205 | 0.263438334 | 3.97612674 | 7.00E-05 | 0.00091272 |
| TCTEX1D4 | 102.0316798 | 2.096642602 | 0.527465724 | 3.9749362 | 7.04E-05 | 0.000916879 |
| SILC1 | 38.65236446 | -1.644173077 | 0.413659847 | -3.9746983 | 7.05E-05 | 0.000917378 |
| PREX1 | 7154.801223 | -1.039130106 | 0.261498897 | -3.9737457 | 7.08E-05 | 0.000920636 |
| STC2 | 468.4386355 | -1.689430646 | 0.42529713 | -3.9723537 | 7.12E-05 | 0.00092519 |
| ADGRG2 | 218.5258421 | -1.097459519 | 0.276308266 | -3.9718664 | 7.13E-05 | 0.000926662 |
| MPP3 | 1810.471906 | -1.343192417 | 0.338498864 | -3.9680855 | 7.25E-05 | 0.0009402 |
| FPR1 | 993.127639 | -1.591270008 | 0.401129239 | -3.9669759 | 7.28E-05 | 0.000943727 |
| CT45A1 | 179.7202655 | -4.273746357 | 1.077449155 | -3.9665411 | 7.29E-05 | 0.00094502 |
| SDS | 60.51514111 | 2.790203067 | 0.703518799 | 3.96606753 | 7.31E-05 | 0.000946468 |
| SLC16A11 | 402.8018897 | 1.046806788 | 0.264260689 | 3.96126565 | 7.46E-05 | 0.000963521 |
| AC140479.3 | 17.62567233 | 1.727135794 | 0.436130589 | 3.9601345 | 7.49E-05 | 0.000966345 |
| EPHA7 | 63.22775603 | 1.663485258 | 0.420431265 | 3.95661645 | 7.60E-05 | 0.000980238 |
| AC100788.1 | 6.017688927 | -5.012457312 | 1.268383611 | -3.9518465 | 7.76E-05 | 0.000997732 |
| CTRL | 29.0923669 | -1.396647707 | 0.353465981 | -3.9512931 | 7.77E-05 | 0.000999592 |
| AL023806.3 | 11.16913602 | -2.706954687 | 0.685194846 | -3.9506349 | 7.79E-05 | 0.001001442 |
| PLCXD2 | 111.7543495 | -1.092583011 | 0.276706932 | -3.9485206 | 7.86E-05 | 0.001009417 |
| CCL15 | 6.617013331 | 3.425418716 | 0.86764022 | 3.94797133 | 7.88E-05 | 0.00101128 |
| MAP3K15 | 194.5432732 | -1.240391024 | 0.314258348 | -3.9470424 | 7.91E-05 | 0.001013842 |
| USH1G | 4.478183094 | 5.173716965 | 1.310730757 | 3.9472004 | 7.91E-05 | 0.001013842 |
| CCDC85A | 841.9787754 | -1.33395324 | 0.338177049 | -3.944541 | 8.00E-05 | 0.001023104 |
| GOLGA2P9 | 3.563184876 | 5.060659722 | 1.283247513 | 3.94363493 | 8.03E-05 | 0.001026058 |
| LINC00551 | 326.0450359 | -2.078029142 | 0.527169271 | -3.9418632 | 8.09E-05 | 0.001032743 |
| ADRA2A | 149.3381434 | 2.046446535 | 0.519372793 | 3.94022668 | 8.14E-05 | 0.001038419 |
| MRC1 | 6858.1481 | -1.201949444 | 0.305117113 | -3.9393052 | 8.17E-05 | 0.001041483 |
| AP003393.1 | 54.2786281 | 2.240490921 | 0.568900431 | 3.93828304 | 8.21E-05 | 0.001044529 |
| PLA1A | 283.6140407 | -1.379752192 | 0.350365736 | -3.938034 | 8.22E-05 | 0.001045147 |
| ARHGEF33 | 163.6075034 | 1.137528527 | 0.288964061 | 3.93657441 | 8.27E-05 | 0.001050116 |
| MKX | 89.71807198 | 1.867643631 | 0.47442771 | 3.93662426 | 8.26E-05 | 0.001050116 |
| ADGRF1 | 124.455106 | 3.241323707 | 0.823761293 | 3.93478516 | 8.33E-05 | 0.001057028 |
| AC069366.1 | 60.3475578 | 1.717139677 | 0.436436384 | 3.93445583 | 8.34E-05 | 0.001058008 |
| RPL30P7 | 26.87426713 | 2.020253596 | 0.513807284 | 3.93192868 | 8.43E-05 | 0.00106777 |
| IGFL4 | 13.27002018 | 1.670803897 | 0.424946275 | 3.93180031 | 8.43E-05 | 0.001067866 |
| AL161910.1 | 32.88025754 | 1.837069246 | 0.467356779 | 3.9307641 | 8.47E-05 | 0.001072003 |
| UCKL1-AS1 | 335.8738904 | 1.516687738 | 0.386022625 | 3.92901255 | 8.53E-05 | 0.001078883 |
| ADRA1B | 221.3329587 | 1.324333395 | 0.337219806 | 3.92721119 | 8.59E-05 | 0.001086029 |
| KHDRBS2 | 498.7342629 | -1.035371755 | 0.263674294 | -3.9267072 | 8.61E-05 | 0.001087824 |
| NOL4 | 28.97083885 | 2.610837257 | 0.665230428 | 3.92471112 | 8.68E-05 | 0.001096398 |
| AC009093.10 | 169.2262628 | 1.502851009 | 0.383045157 | 3.92343039 | 8.73E-05 | 0.001101399 |
| AL021392.1 | 134.1188726 | 1.280504907 | 0.326376153 | 3.92340217 | 8.73E-05 | 0.001101399 |
| AC099521.2 | 54.49277113 | 1.902957555 | 0.485040956 | 3.92329252 | 8.73E-05 | 0.001101414 |
| AC254633.1 | 21.72962848 | 1.460605703 | 0.37270277 | 3.91895586 | 8.89E-05 | 0.001119933 |
| AC011473.3 | 18.96157585 | 2.277573728 | 0.581504441 | 3.91669189 | 8.98E-05 | 0.001128505 |
| CDT1 | 140.0002683 | 1.064549116 | 0.271921245 | 3.91491704 | 9.04E-05 | 0.001135837 |
| TUBB1 | 90.21711088 | -1.735485037 | 0.443386543 | -3.9141581 | 9.07E-05 | 0.001138412 |
| SVIL2P | 61.93588495 | -1.489060211 | 0.38100682 | -3.9082246 | 9.30E-05 | 0.001162129 |
| AC104695.2 | 27.97399454 | 1.589556373 | 0.406826215 | 3.90721226 | 9.34E-05 | 0.001165985 |
| AC093909.5 | 20.06042018 | 2.813728144 | 0.720374839 | 3.90592229 | 9.39E-05 | 0.001171198 |
| TMSB15A | 70.75345738 | -1.127801769 | 0.288823345 | -3.9048151 | 9.43E-05 | 0.001176059 |
| S1PR5 | 472.2180171 | -1.727043329 | 0.442311086 | -3.9045897 | 9.44E-05 | 0.001176641 |
| HSPB8 | 1788.356429 | -1.12970468 | 0.2894543 | -3.9028775 | 9.51E-05 | 0.001183529 |
| MEIS3P2 | 46.29786776 | 1.590986784 | 0.408069165 | 3.89881647 | 9.67E-05 | 0.001199854 |
| AL161908.1 | 5.173625386 | 5.612343515 | 1.440787976 | 3.89532923 | 9.81E-05 | 0.001214028 |
| MUC20P1 | 215.1846192 | 1.485238917 | 0.381317804 | 3.89501592 | 9.82E-05 | 0.001215071 |
| AF106564.1 | 3.860012441 | -4.366221266 | 1.121086263 | -3.8946345 | 9.83E-05 | 0.001215929 |
| CDRT4 | 637.3156851 | 2.21813962 | 0.569694007 | 3.8935632 | 9.88E-05 | 0.001220256 |
| AL138787.1 | 56.68299082 | 2.646355761 | 0.679941497 | 3.89203449 | 9.94E-05 | 0.001227441 |
| SLC17A8 | 32.5035188 | 2.052672818 | 0.527965775 | 3.88788993 | 0.000101119 | 0.00124535 |
| AC020663.1 | 4.391994233 | 4.475861437 | 1.151555855 | 3.88679491 | 0.000101577 | 0.001250085 |
| NALCN-AS1 | 13.67418019 | -2.17893535 | 0.561135374 | -3.8830832 | 0.00010314 | 0.001265865 |
| PCDHA4 | 38.64778394 | -3.134239122 | 0.807666696 | -3.8806096 | 0.000104195 | 0.001277488 |
| KIAA0825 | 87.17869906 | 1.021605908 | 0.263342057 | 3.87938759 | 0.00010472 | 0.001283042 |
| CCN4 | 99.32050271 | 1.500750351 | 0.386947401 | 3.87843502 | 0.000105131 | 0.001286418 |
| CR392000.2 | 86.7145704 | 2.341231771 | 0.604005712 | 3.87617489 | 0.000106111 | 0.001296751 |
| CRLF1 | 513.1404894 | 1.156331636 | 0.298365313 | 3.87555652 | 0.000106381 | 0.001299492 |
| BST1 | 262.2946195 | -1.091485652 | 0.281665855 | -3.8751082 | 0.000106577 | 0.00130133 |
| KIF21B | 1465.042474 | -1.041415762 | 0.268865393 | -3.8733723 | 0.00010734 | 0.001308957 |
| PPP1R14C | 1753.011838 | 1.040635556 | 0.268795374 | 3.87147867 | 0.000108177 | 0.001317427 |
| ANKRD45 | 185.3795443 | 2.775478322 | 0.716950565 | 3.87122691 | 0.000108289 | 0.001317715 |
| MPPED2 | 58.71477218 | 1.207690006 | 0.312147552 | 3.86897158 | 0.000109295 | 0.001327697 |
| CHST1 | 649.1875799 | -1.159849272 | 0.299813378 | -3.8685708 | 0.000109475 | 0.001329315 |
| TRGC1 | 172.1811901 | -1.17227977 | 0.303201413 | -3.8663401 | 0.000110481 | 0.001339246 |
| ZNF750 | 285.5857297 | 1.023602503 | 0.265065338 | 3.86169882 | 0.000112601 | 0.001360325 |
| ANGPTL7 | 169.0118399 | -1.624604585 | 0.420784179 | -3.8608975 | 0.000112971 | 0.001364217 |
| AC008543.1 | 113.1924755 | 1.024255318 | 0.265567134 | 3.85686023 | 0.000114853 | 0.001384008 |
| AL121890.4 | 3.590455187 | -4.270304163 | 1.107733047 | -3.8549939 | 0.000115733 | 0.001392843 |
| RGS17 | 58.08614125 | 1.166803069 | 0.302882345 | 3.85233107 | 0.000116999 | 0.001405709 |
| ADAM28 | 530.4457039 | 1.254064202 | 0.325526957 | 3.85241275 | 0.00011696 | 0.001405709 |
| DM1-AS | 307.7759317 | 1.191021592 | 0.309321427 | 3.85043352 | 0.000117909 | 0.001413668 |
| TP53AIP1 | 29.84056663 | 1.832963607 | 0.476076496 | 3.85014514 | 0.000118048 | 0.001414739 |
| USP30-AS1 | 66.36002888 | -1.205657405 | 0.313332548 | -3.8478524 | 0.000119158 | 0.001426243 |
| LRRN4 | 312.8924004 | -1.56684164 | 0.407556226 | -3.8444797 | 0.000120808 | 0.001442973 |
| PDZD4 | 2413.04739 | 1.01546801 | 0.26431413 | 3.84189832 | 0.000122086 | 0.001452762 |
| AL391987.2 | 76.5831915 | 1.629699081 | 0.424386009 | 3.84013386 | 0.000122967 | 0.001460475 |
| AC004923.4 | 71.4822807 | 1.099376261 | 0.286295252 | 3.8400087 | 0.00012303 | 0.001460475 |
| P2RY1 | 633.2679031 | -1.009530853 | 0.262931068 | -3.8395267 | 0.000123272 | 0.00146138 |
| AC108134.4 | 19.42468103 | 1.386537469 | 0.361173112 | 3.83898308 | 0.000123545 | 0.00146401 |
| OR7E36P | 58.88889489 | 2.060787036 | 0.537615081 | 3.83320169 | 0.000126486 | 0.001497619 |
| BDKRB2 | 363.0675042 | 1.511037688 | 0.394213923 | 3.83303989 | 0.000126569 | 0.001497983 |
| XG | 52.12296758 | 1.312893134 | 0.34258493 | 3.83231433 | 0.000126943 | 0.001501786 |
| LINC01708 | 5.241026999 | 4.659500142 | 1.216574794 | 3.83001536 | 0.000128135 | 0.001513376 |
| AL353770.4 | 57.71869587 | 2.49869614 | 0.652436601 | 3.82979149 | 0.000128252 | 0.0015135 |
| MCHR1 | 42.42711458 | 2.308981594 | 0.602944045 | 3.82951223 | 0.000128398 | 0.001514591 |
| GNLY | 1520.12213 | -1.548878544 | 0.40452581 | -3.8288745 | 0.000128731 | 0.001517893 |
| AL031595.3 | 124.7616978 | -1.149060815 | 0.300191265 | -3.8277623 | 0.000129314 | 0.001523347 |
| IVL | 119.8396839 | 2.938448369 | 0.768009369 | 3.82605797 | 0.000130212 | 0.001532822 |
| URAHP | 136.6601377 | 1.516421316 | 0.396523262 | 3.82429346 | 0.000131148 | 0.001541931 |
| PACRG-AS1 | 26.80863173 | 2.274282621 | 0.594785655 | 3.8237012 | 0.000131463 | 0.001544368 |
| AL031663.1 | 7.029216886 | 3.181472347 | 0.832107642 | 3.82339037 | 0.000131629 | 0.00154568 |
| EPPIN-WFDC6 | 20.48793165 | 2.944807369 | 0.77080545 | 3.82042884 | 0.00013322 | 0.001559864 |
| UPK2 | 13.43986644 | 2.55862415 | 0.670327119 | 3.81697843 | 0.000135096 | 0.001576654 |
| PTPRZ1 | 86.70719335 | 2.525408179 | 0.661644444 | 3.81686599 | 0.000135158 | 0.001576727 |
| CFP | 724.6463532 | -1.083275939 | 0.283832454 | -3.8166035 | 0.000135301 | 0.00157776 |
| SOX21 | 25.95497186 | 2.319601173 | 0.607868738 | 3.81595734 | 0.000135656 | 0.001581248 |
| AC016590.3 | 33.85732653 | 1.48712562 | 0.389980524 | 3.81333305 | 0.000137105 | 0.001595533 |
| AC025265.1 | 22.91729984 | 1.575723948 | 0.413636781 | 3.80943866 | 0.000139283 | 0.001618232 |
| GLS2 | 44.04130783 | 1.00499875 | 0.264045896 | 3.80615175 | 0.000141146 | 0.001636545 |
| NGFR | 304.575185 | 1.102534363 | 0.289681074 | 3.80602829 | 0.000141216 | 0.001636697 |
| TRPM5 | 68.6595545 | 2.73714378 | 0.719369045 | 3.80492294 | 0.000141848 | 0.001642606 |
| DNAJC12 | 130.0287165 | 1.134118941 | 0.298110333 | 3.80435972 | 0.000142171 | 0.00164376 |
| GHRL | 320.2789248 | 1.55351248 | 0.408382094 | 3.8040661 | 0.00014234 | 0.001644547 |
| LINC00648 | 20.08944273 | -1.851790112 | 0.48680196 | -3.8039907 | 0.000142384 | 0.001644547 |
| ANXA13 | 148.4581703 | 2.760703142 | 0.725939353 | 3.80293909 | 0.00014299 | 0.001650542 |
| FUT1 | 482.8719375 | -1.165162284 | 0.306474516 | -3.8018244 | 0.000143635 | 0.001655977 |
| DSCAML1 | 399.257572 | 1.056715312 | 0.278086659 | 3.79994968 | 0.000144725 | 0.001667207 |
| LINC02785 | 40.1314745 | -1.288779338 | 0.339249455 | -3.7989135 | 0.000145332 | 0.001672586 |
| MGAT3-AS1 | 7.569951073 | -5.835571415 | 1.537121965 | -3.7964271 | 0.000146797 | 0.001686977 |
| AL035701.1 | 40.52219054 | 2.008509689 | 0.529432488 | 3.79370313 | 0.000148417 | 0.001703542 |
| KIR3DX1 | 16.85171279 | -2.492691311 | 0.657099948 | -3.7934736 | 0.000148554 | 0.001704432 |
| OLR1 | 2666.773331 | -1.172448983 | 0.309183719 | -3.7920787 | 0.000149392 | 0.001711284 |
| AGER | 10982.90933 | -1.334282891 | 0.352516483 | -3.7850227 | 0.000153695 | 0.001750731 |
| AL391840.3 | 40.1517045 | 1.2341011 | 0.326074184 | 3.78472495 | 0.000153879 | 0.001752128 |
| AL391840.1 | 25.84299466 | 2.282369927 | 0.603313664 | 3.78305691 | 0.000154914 | 0.001761105 |
| GRM5-AS1 | 23.58081522 | 6.942443083 | 1.83562379 | 3.78206205 | 0.000155535 | 0.001766048 |
| TMEM204 | 4531.029794 | -1.05830807 | 0.279821867 | -3.7820778 | 0.000155525 | 0.001766048 |
| RPL39P40 | 7.928524922 | 1.971754653 | 0.521545437 | 3.78059995 | 0.000156451 | 0.001775039 |
| UROC1 | 16.45144307 | 2.486274796 | 0.657736477 | 3.780047 | 0.000156799 | 0.001777217 |
| DIO1 | 73.96448129 | 1.746373982 | 0.462220688 | 3.77822549 | 0.00015795 | 0.001788489 |
| LILRA1 | 38.07317838 | -1.517087805 | 0.402247004 | -3.7715329 | 0.000162248 | 0.00182989 |
| AUXG01000058.1 | 2169.10626 | -1.07143318 | 0.284245434 | -3.7693945 | 0.000163644 | 0.001842365 |
| CDH5 | 14862.93617 | -1.101470728 | 0.29259433 | -3.7644979 | 0.000166884 | 0.001876986 |
| AL137139.2 | 131.7664354 | -1.476071601 | 0.39211478 | -3.7643865 | 0.000166958 | 0.001877082 |
| TSPEAR-AS1 | 116.3890606 | -1.287359444 | 0.342101316 | -3.7630941 | 0.000167824 | 0.001885714 |
| HEY2 | 392.1082218 | 1.119619188 | 0.297579326 | 3.76242262 | 0.000168275 | 0.001889653 |
| AP001972.5 | 48.62463956 | 1.589776936 | 0.422652024 | 3.76143221 | 0.000168943 | 0.001896406 |
| KIF26B | 167.8796281 | 1.113947571 | 0.296592527 | 3.75581807 | 0.000172776 | 0.001931072 |
| AC108097.1 | 23.42403962 | 2.575577526 | 0.685891952 | 3.75507763 | 0.000173288 | 0.00193578 |
| AADACP1 | 66.61280968 | 1.452373312 | 0.386806088 | 3.75478401 | 0.000173491 | 0.001936785 |
| AL390719.2 | 476.3155819 | 1.192525572 | 0.317778572 | 3.75269348 | 0.000174945 | 0.001950723 |
| LAMP3 | 17112.68413 | -1.048772667 | 0.279490884 | -3.7524396 | 0.000175122 | 0.001951174 |
| SEC14L5 | 42.39372204 | 1.363059541 | 0.363275053 | 3.75214188 | 0.00017533 | 0.001952731 |
| ALOX15 | 3589.321507 | 2.546778462 | 0.679775484 | 3.74649943 | 0.000179319 | 0.001992492 |
| AL137139.3 | 8.28702836 | -4.9848985 | 1.331126701 | -3.7448715 | 0.000180486 | 0.002003115 |
| AL137847.2 | 44.30105608 | 1.803988609 | 0.482119218 | 3.74178946 | 0.000182715 | 0.00202391 |
| MUCL3 | 114.1544444 | -1.102200853 | 0.29481566 | -3.7386103 | 0.00018504 | 0.002047287 |
| HSD17B2 | 52.60645574 | 1.589667586 | 0.425614839 | 3.73499099 | 0.000187722 | 0.002072935 |
| WFDC6 | 66.62027316 | 2.395907222 | 0.641541802 | 3.73460812 | 0.000188008 | 0.002075288 |
| IL18R1 | 1462.047213 | -1.528854261 | 0.409580392 | -3.732733 | 0.000189413 | 0.002085704 |
| AC026704.1 | 40.80416189 | 2.828957842 | 0.757935269 | 3.7324531 | 0.000189624 | 0.002086669 |
| PAX9 | 54.94289352 | 2.301809562 | 0.61692423 | 3.73110579 | 0.000190641 | 0.002094484 |
| CABYR | 56.19478755 | 1.150353164 | 0.30832085 | 3.73102619 | 0.000190701 | 0.002094484 |
| AC005487.1 | 15.37232106 | 4.142883844 | 1.110431739 | 3.73087665 | 0.000190815 | 0.002094921 |
| NBEA | 648.8964719 | 1.076599625 | 0.288624597 | 3.73010351 | 0.000191401 | 0.002100494 |
| AC022164.1 | 180.9048516 | -1.127400763 | 0.302255688 | -3.7299571 | 0.000191512 | 0.002100494 |
| GZMB | 874.0476652 | -1.430838811 | 0.383982062 | -3.7263168 | 0.000194298 | 0.002127434 |
| AC118755.2 | 6.053226408 | 3.062776028 | 0.822841894 | 3.72219262 | 0.0001975 | 0.002158352 |
| AC004863.1 | 47.03243998 | 2.009959871 | 0.540057902 | 3.72174884 | 0.000197848 | 0.002159668 |
| SPAAR | 517.6659744 | -1.071379015 | 0.287863509 | -3.7218299 | 0.000197784 | 0.002159668 |
| AC124319.2 | 33.56961957 | 1.57405531 | 0.422957726 | 3.72154287 | 0.000198009 | 0.002160603 |
| AC004556.4 | 126.69552 | 1.474176625 | 0.396241385 | 3.72040044 | 0.000198907 | 0.002167913 |
| SCD | 7170.338982 | -1.140930044 | 0.306804413 | -3.7187537 | 0.000200208 | 0.002179249 |
| NDUFA5P8 | 3.03855177 | 3.518443537 | 0.946120324 | 3.71881192 | 0.000200162 | 0.002179249 |
| ANGPT4 | 104.214939 | -1.805405969 | 0.485627422 | -3.7176771 | 0.000201063 | 0.002187231 |
| STAC | 602.5176063 | -1.005492015 | 0.270539298 | -3.7166209 | 0.000201905 | 0.002195554 |
| SMIM25 | 1264.069142 | -1.043611626 | 0.280987183 | -3.7140898 | 0.000203936 | 0.002213424 |
| FFAR2 | 82.96326151 | -2.017182226 | 0.543230428 | -3.7133086 | 0.000204567 | 0.002219426 |
| SOWAHA | 29.52188956 | 2.279498645 | 0.61398449 | 3.7126323 | 0.000205115 | 0.00222283 |
| AC099518.4 | 20.08520297 | 2.367111453 | 0.638139348 | 3.70939586 | 0.000207754 | 0.002248872 |
| AC104035.1 | 20.51337424 | 2.789017706 | 0.752114275 | 3.70823663 | 0.000208708 | 0.002256621 |
| LINC02605 | 129.4076426 | 1.749083024 | 0.47201605 | 3.70555837 | 0.000210926 | 0.002278012 |
| TRIM58 | 63.76571185 | -1.391850495 | 0.376109838 | -3.700649 | 0.000215049 | 0.002319031 |
| CEACAM3 | 41.06977562 | -1.773080329 | 0.479650469 | -3.6966092 | 0.000218498 | 0.002352672 |
| AC005264.1 | 61.40178365 | -1.47156656 | 0.398199773 | -3.6955485 | 0.000219413 | 0.002360734 |
| TMEM100 | 10942.20871 | -1.66192521 | 0.449724269 | -3.6954315 | 0.000219514 | 0.002360931 |
| F2RL3 | 1753.964751 | -1.638593979 | 0.443484463 | -3.6948171 | 0.000220045 | 0.002365755 |
| S100A8 | 479.1410655 | -2.151888814 | 0.582809897 | -3.6922654 | 0.000222265 | 0.002386926 |
| LINC02018 | 246.6182703 | 1.320603993 | 0.357717201 | 3.69175424 | 0.000222713 | 0.002389929 |
| HSD3BP5 | 36.19702267 | -1.380721089 | 0.374037371 | -3.6913988 | 0.000223024 | 0.002391822 |
| AL161431.1 | 43.29560124 | -1.492552918 | 0.404419008 | -3.6906102 | 0.000223717 | 0.002397097 |
| CYP4F29P | 161.7942018 | 1.59338888 | 0.431904005 | 3.68921997 | 0.000224943 | 0.00240752 |
| CRISPLD2 | 4547.283034 | -1.04569389 | 0.283623244 | -3.6869118 | 0.000226992 | 0.002425812 |
| PRLR | 93.97104144 | -1.754732594 | 0.47620469 | -3.6848285 | 0.000228857 | 0.00244208 |
| AL391280.2 | 43.06502708 | -1.235719732 | 0.335454293 | -3.6837201 | 0.000229855 | 0.002449979 |
| FSIP1 | 93.02778914 | 1.745009557 | 0.473914297 | 3.68212052 | 0.000231302 | 0.002463565 |
| CAMP | 108.5205359 | -1.954189753 | 0.531200844 | -3.6788152 | 0.00023432 | 0.002491987 |
| NFE2 | 174.7926805 | -1.223088324 | 0.332617979 | -3.6771564 | 0.000235848 | 0.002506373 |
| ZNF663P | 57.37137589 | 1.68967833 | 0.459843409 | 3.67446461 | 0.000238349 | 0.002529174 |
| SULT1B1 | 125.9044502 | -1.031645402 | 0.280892261 | -3.6727441 | 0.00023996 | 0.002544376 |
| CSPG4P10 | 1288.069102 | 1.370293004 | 0.37331056 | 3.6706516 | 0.000241933 | 0.002564345 |
| SUGCT | 61.30575089 | 1.33684397 | 0.364377292 | 3.66884545 | 0.000243648 | 0.002581568 |
| AC036214.4 | 26.63061548 | 2.082848633 | 0.567958478 | 3.66725512 | 0.000245168 | 0.00259478 |
| AC008163.1 | 7.365160568 | 5.703596069 | 1.557634397 | 3.66170398 | 0.000250543 | 0.002646758 |
| AC092384.2 | 113.4480282 | -1.176210596 | 0.321336481 | -3.6603706 | 0.000251851 | 0.002659585 |
| HABP2 | 49.90092759 | 2.459269603 | 0.671985973 | 3.65970378 | 0.000252507 | 0.002663556 |
| AC055714.1 | 85.07606526 | 2.44967888 | 0.669414344 | 3.6594359 | 0.000252771 | 0.002665356 |
| RBP2 | 51.96377784 | -1.625407076 | 0.444484521 | -3.6568362 | 0.000255347 | 0.002691528 |
| GREM1 | 17.24112388 | 2.138952832 | 0.585465419 | 3.65342301 | 0.000258767 | 0.002721695 |
| CTHRC1 | 183.9287124 | 1.66790202 | 0.45689266 | 3.65053363 | 0.000261696 | 0.002746268 |
| AC073655.2 | 186.7494798 | 1.223641743 | 0.33531618 | 3.64921772 | 0.00026304 | 0.002757496 |
| MGAT3 | 2347.603864 | -1.015746647 | 0.278373757 | -3.6488592 | 0.000263407 | 0.002760165 |
| PCP4L1 | 189.4026537 | 1.143984306 | 0.31357575 | 3.64819124 | 0.000264093 | 0.002766333 |
| NAT8L | 38.7926589 | 1.3777995 | 0.377782886 | 3.64706701 | 0.000265251 | 0.00277744 |
| AL663070.2 | 244.6995233 | -1.395330392 | 0.382613797 | -3.6468376 | 0.000265488 | 0.0027789 |
| ILDR2 | 135.5271042 | -1.231408263 | 0.337721226 | -3.646227 | 0.000266119 | 0.00278331 |
| PRRT1B | 287.3907126 | 1.056581699 | 0.289780331 | 3.64614705 | 0.000266202 | 0.00278331 |
| MMP25 | 612.3057961 | -1.282932763 | 0.351967774 | -3.6450291 | 0.000267362 | 0.002789303 |
| AL606469.1 | 73.03353774 | -1.28043807 | 0.351436402 | -3.6434418 | 0.000269017 | 0.002804517 |
| FRMD5 | 20.00095327 | 1.57075153 | 0.431165953 | 3.64303238 | 0.000269445 | 0.002807957 |
| TNR | 1042.793738 | -2.456767424 | 0.674571112 | -3.6419695 | 0.00027056 | 0.002816473 |
| P3H2 | 4379.668344 | -1.030128723 | 0.282856515 | -3.6418773 | 0.000270657 | 0.002816473 |
| NEFM | 131.1944726 | -2.308289942 | 0.633865904 | -3.6416061 | 0.000270942 | 0.002817687 |
| SFTPA1 | 297363.4447 | -1.031362218 | 0.28335426 | -3.6398331 | 0.000272815 | 0.002834789 |
| AP001372.1 | 9.35105892 | 2.166370689 | 0.595267122 | 3.63932529 | 0.000273353 | 0.002839351 |
| AP000553.7 | 7.397313387 | 2.169428769 | 0.596231207 | 3.63856964 | 0.000274156 | 0.002846657 |
| KRTAP5-AS1 | 19.72674388 | -2.042296616 | 0.561394196 | -3.6379012 | 0.000274869 | 0.002850939 |
| AC097493.1 | 22.20967767 | 2.609007902 | 0.717914042 | 3.63415082 | 0.000278898 | 0.002885859 |
| AC026992.2 | 367.8112373 | -1.175190848 | 0.323503735 | -3.6326964 | 0.000280475 | 0.002897496 |
| EPPIN | 78.38931459 | 2.54834539 | 0.701667981 | 3.63183936 | 0.000281408 | 0.002903983 |
| FAM237B | 35.84104913 | 2.479266558 | 0.682942169 | 3.63027306 | 0.000283122 | 0.00291955 |
| AC004264.2 | 9.955471018 | -3.645253963 | 1.004346271 | -3.6294793 | 0.000283994 | 0.002926426 |
| LCN12 | 175.135448 | 1.807840962 | 0.498477959 | 3.62672196 | 0.000287042 | 0.002951442 |
| SYT5 | 40.12874815 | 1.93912855 | 0.535199736 | 3.62318667 | 0.000290996 | 0.002984563 |
| ETV5-AS1 | 17.37786208 | -2.521323574 | 0.696064554 | -3.6222554 | 0.000292046 | 0.002990866 |
| AP002008.1 | 6.265092021 | 3.438636585 | 0.949297255 | 3.62229698 | 0.000291999 | 0.002990866 |
| AC023510.1 | 8.774117034 | 2.905670899 | 0.802192568 | 3.62216133 | 0.000292152 | 0.002990866 |
| SLITRK2 | 180.2898569 | -1.432333047 | 0.395400118 | -3.6224902 | 0.000291781 | 0.002990866 |
| AL355499.1 | 46.17362405 | -1.160720957 | 0.320721891 | -3.6190886 | 0.000295642 | 0.00302244 |
| GRASP | 7799.018406 | -1.027473697 | 0.284159364 | -3.6158361 | 0.00029938 | 0.003056266 |
| FAM201A | 94.68736498 | 1.080112949 | 0.298798404 | 3.61485515 | 0.000300516 | 0.003064572 |
| MIR6510 | 6.108568667 | 2.027478873 | 0.561199647 | 3.61275864 | 0.000302957 | 0.003087257 |
| LINC02696 | 51.4170616 | 1.08335456 | 0.30019688 | 3.60881353 | 0.000307601 | 0.003125681 |
| AC009652.1 | 36.72474774 | 2.142154042 | 0.593657293 | 3.6084018 | 0.000308089 | 0.003128382 |
| R3HDML | 12.39775484 | 2.612651526 | 0.724232511 | 3.60747617 | 0.00030919 | 0.003135096 |
| AC012313.2 | 106.7389056 | 1.018256923 | 0.282364238 | 3.60618233 | 0.000310735 | 0.003149641 |
| AC004947.2 | 76.60903172 | -1.291136766 | 0.358146622 | -3.6050508 | 0.000312092 | 0.003160027 |
| GCNT3 | 30.48073522 | 2.77423861 | 0.769911123 | 3.6033232 | 0.000314174 | 0.00317773 |
| AL139142.2 | 60.90016113 | 2.176934538 | 0.604213363 | 3.60292352 | 0.000314658 | 0.003181494 |
| MAPK4 | 685.7473043 | -1.161903178 | 0.32255747 | -3.6021586 | 0.000315586 | 0.003189484 |
| GRHL3 | 14.43263148 | 2.050910068 | 0.569558504 | 3.60087691 | 0.000317146 | 0.003200972 |
| BX005214.1 | 42.98877602 | 2.849587382 | 0.791898971 | 3.59842289 | 0.000320153 | 0.003227896 |
| AC235565.2 | 91.9934875 | 1.926651536 | 0.535476752 | 3.59801155 | 0.000320659 | 0.003231861 |
| AC104590.1 | 28.6706756 | 1.930148972 | 0.536605154 | 3.59696317 | 0.000321954 | 0.003243764 |
| NXF2B | 12.59377077 | 3.61751338 | 1.006512576 | 3.59410649 | 0.000325507 | 0.003276086 |
| MNDA | 2209.786792 | -1.051395077 | 0.292637561 | -3.5928234 | 0.000327114 | 0.003289944 |
| AC009070.1 | 11.20703204 | -4.877648186 | 1.358778711 | -3.5897296 | 0.000331021 | 0.00332104 |
| LRRC26 | 71.04542825 | 1.899331245 | 0.529269563 | 3.5885896 | 0.000332472 | 0.003329737 |
| FCN3 | 4801.492869 | -2.122483087 | 0.592103252 | -3.5846503 | 0.00033753 | 0.003372881 |
| RUNDC3B | 135.5767425 | 2.034138458 | 0.567467439 | 3.58459062 | 0.000337607 | 0.003372881 |
| OSCAR | 181.0146109 | -1.175888505 | 0.328055135 | -3.5844234 | 0.000337824 | 0.003373273 |
| ANKUB1 | 103.1778303 | 1.945244364 | 0.542743768 | 3.58409341 | 0.000338251 | 0.003375764 |
| CD52 | 2470.514989 | -1.016883922 | 0.28407098 | -3.5796825 | 0.000344012 | 0.003426065 |
| NKX2-2 | 4.353910184 | 5.774417071 | 1.613160889 | 3.57956674 | 0.000344164 | 0.003426387 |
| AC002064.2 | 4.41035049 | 4.482500227 | 1.253098456 | 3.57713331 | 0.000347383 | 0.003454814 |
| GGT1 | 2389.090559 | -1.117473024 | 0.312579696 | -3.575002 | 0.000350225 | 0.003475809 |
| AL353747.2 | 8.891624883 | 2.516223008 | 0.704694766 | 3.57065659 | 0.000356088 | 0.003523913 |
| AC008752.3 | 16.18689662 | 3.176470538 | 0.889621763 | 3.57058547 | 0.000356184 | 0.003523913 |
| SMIM31 | 54.38864309 | 2.387258697 | 0.668985242 | 3.56847737 | 0.000359062 | 0.003547462 |
| CRISP2 | 143.5675362 | 2.737793446 | 0.767453479 | 3.56737382 | 0.000360577 | 0.003559964 |
| PNMA6A | 109.6966052 | 1.440701142 | 0.403918286 | 3.56681337 | 0.000361349 | 0.003566349 |
| MIR132 | 4.565264646 | 1.912703207 | 0.536337634 | 3.56622972 | 0.000362154 | 0.00357306 |
| TULP1 | 37.80697928 | 1.324881263 | 0.371532992 | 3.56598551 | 0.000362491 | 0.003573917 |
| RPH3A | 16.8958043 | -3.146058035 | 0.882391585 | -3.5653763 | 0.000363335 | 0.003580992 |
| ISL1 | 6.712833106 | 3.103394161 | 0.870626015 | 3.56455482 | 0.000364474 | 0.00358831 |
| LRRC32 | 2924.507716 | -1.238566191 | 0.347474721 | -3.5644786 | 0.00036458 | 0.00358831 |
| STYK1 | 58.02535427 | 1.462105439 | 0.410168298 | 3.5646476 | 0.000364345 | 0.00358831 |
| SEMA5B | 454.4243884 | -1.034058509 | 0.290123925 | -3.5641959 | 0.000364973 | 0.003590939 |
| HNRNPA1P50 | 9.550745075 | 2.337430111 | 0.655953587 | 3.56340777 | 0.000366071 | 0.00359926 |
| STXBP6 | 2893.853064 | -1.220358617 | 0.342789947 | -3.560077 | 0.000370746 | 0.003640203 |
| AC244197.2 | 275.1346639 | 1.000938687 | 0.281441925 | 3.55646618 | 0.000375877 | 0.003682973 |
| GBP1P1 | 317.3051649 | -1.036116304 | 0.291453016 | -3.5550029 | 0.000377975 | 0.003698721 |
| COL4A1 | 43497.13458 | -1.143146466 | 0.321912899 | -3.5511049 | 0.000383618 | 0.003748518 |
| AC117395.1 | 21.60003651 | 2.031370177 | 0.572272352 | 3.54965633 | 0.000385734 | 0.003767911 |
| FHL5 | 517.5909815 | -1.084932046 | 0.305698173 | -3.5490302 | 0.000386653 | 0.003774297 |
| SOX9 | 421.682888 | 1.28478573 | 0.36217243 | 3.54744211 | 0.000388991 | 0.003794527 |
| GPR146 | 940.2284166 | -1.162926979 | 0.327842138 | -3.5472163 | 0.000389325 | 0.003796482 |
| MTTP | 42.37356981 | 1.617990765 | 0.456165667 | 3.54693674 | 0.000389738 | 0.003799215 |
| AC025271.4 | 106.5933771 | -1.128951118 | 0.318414392 | -3.5455405 | 0.000391809 | 0.003815487 |
| HTR3E | 9.824076274 | 4.637436646 | 1.308690422 | 3.5435704 | 0.000394748 | 0.003838866 |
| MYZAP | 5798.616111 | -1.197583389 | 0.33821072 | -3.5409386 | 0.000398706 | 0.003872081 |
| AC104966.1 | 4.616709862 | 4.51425602 | 1.2750936 | 3.54033305 | 0.000399622 | 0.003878928 |
| AC012588.1 | 5.41515782 | 4.529058464 | 1.279289245 | 3.54029277 | 0.000399683 | 0.003878928 |
| EDN1 | 7955.439712 | -1.346932561 | 0.380714898 | -3.5379035 | 0.000403317 | 0.003910205 |
| WNT16 | 18.22790757 | 2.309718751 | 0.652846905 | 3.5379179 | 0.000403295 | 0.003910205 |
| LILRB2 | 697.7369209 | -1.190974773 | 0.337228073 | -3.5316596 | 0.000412961 | 0.003991485 |
| AC009244.2 | 165.7117456 | -1.492427582 | 0.42271026 | -3.5306159 | 0.000414593 | 0.004003197 |
| AL356608.3 | 22.27156295 | 1.853065346 | 0.525787069 | 3.52436462 | 0.0004245 | 0.00408916 |
| C2CD4D | 74.12148111 | 1.003285884 | 0.284969495 | 3.52067818 | 0.000430445 | 0.004138043 |
| RAB26 | 42.11407657 | 1.544046958 | 0.438623324 | 3.5202117 | 0.000431202 | 0.004142536 |
| SLC6A4 | 6509.636553 | -2.293365363 | 0.651582808 | -3.5196837 | 0.000432062 | 0.004146401 |
| CST7 | 479.1729406 | -1.043678669 | 0.296606569 | -3.5187308 | 0.000433616 | 0.004157325 |
| AL512506.3 | 26.09498641 | 2.920327041 | 0.830386741 | 3.51682764 | 0.000436737 | 0.004181624 |
| HOXB1 | 23.1650901 | 3.406964745 | 0.968837685 | 3.51654854 | 0.000437197 | 0.004184618 |
| AP000925.1 | 4.445179542 | -5.066338893 | 1.443156804 | -3.5105949 | 0.000447105 | 0.004263718 |
| AC136297.1 | 11.05559653 | -2.351159829 | 0.669882921 | -3.5098071 | 0.000448432 | 0.004273513 |
| AC104958.1 | 6.433521373 | -5.600679059 | 1.596823811 | -3.507387 | 0.000452531 | 0.004306816 |
| DSC2 | 624.8557717 | -1.156524017 | 0.329947898 | -3.5051716 | 0.000456313 | 0.004337026 |
| THBD | 2406.826837 | -1.001779483 | 0.285852395 | -3.5045342 | 0.000457407 | 0.004345975 |
| GNG13 | 12.42577177 | 4.469486555 | 1.275664821 | 3.50365275 | 0.000458923 | 0.004358932 |
| LINC01602 | 14.39227855 | 3.876292618 | 1.106504731 | 3.50318667 | 0.000459727 | 0.004365113 |
| HHATL-AS1 | 19.06202516 | 2.289106072 | 0.653629863 | 3.50214426 | 0.00046153 | 0.004379312 |
| PCDH12 | 2490.07268 | -1.000239702 | 0.285697464 | -3.5010451 | 0.000463437 | 0.00439303 |
| FAM187A | 346.9147857 | 2.240729824 | 0.64008879 | 3.50065469 | 0.000464117 | 0.004396547 |
| CYCSP10 | 12.35040497 | 1.38075658 | 0.394443223 | 3.50052048 | 0.000464351 | 0.004397301 |
| AP000547.3 | 157.3481377 | -1.415288812 | 0.404330047 | -3.5003305 | 0.000464682 | 0.004398975 |
| AC068594.1 | 3.848969165 | -3.861739687 | 1.103438541 | -3.4997325 | 0.000465725 | 0.004407393 |
| SYN2 | 246.7960433 | -1.682224244 | 0.480836629 | -3.498536 | 0.00046782 | 0.00442428 |
| FO393414.3 | 12.00887441 | -2.354609064 | 0.673019058 | -3.4985771 | 0.000467748 | 0.00442428 |
| SYNDIG1L | 130.7165461 | -1.187118902 | 0.339437482 | -3.4973124 | 0.000469971 | 0.004440208 |
| TFAP2B | 214.818537 | 4.217744472 | 1.206144315 | 3.49688211 | 0.00047073 | 0.004445903 |
| AC117488.1 | 61.13960468 | -1.186974038 | 0.33948309 | -3.4964158 | 0.000471553 | 0.004449179 |
| CGB7 | 65.39068453 | 1.106163834 | 0.316446602 | 3.49557817 | 0.000473036 | 0.004460298 |
| CHRM3-AS2 | 77.7788542 | -1.292955571 | 0.369934396 | -3.4950942 | 0.000473894 | 0.004464342 |
| TENT5B | 1271.886427 | -1.051410044 | 0.300942278 | -3.4937266 | 0.000476329 | 0.004483934 |
| LINC01484 | 57.68283402 | 1.282617718 | 0.367141956 | 3.49351987 | 0.000476698 | 0.004484689 |
| FRMPD2B | 226.4582071 | 1.725986307 | 0.494318743 | 3.4916465 | 0.000480053 | 0.004510064 |
| AC138356.3 | 6.432911367 | 5.503289671 | 1.576362649 | 3.49113174 | 0.000480979 | 0.004517275 |
| SLC5A4 | 149.4493764 | -1.348097403 | 0.386414821 | -3.4887311 | 0.000485319 | 0.004552041 |
| AC025280.3 | 26.73528674 | -1.167712444 | 0.334826471 | -3.4875153 | 0.000487531 | 0.004568281 |
| LINC01146 | 34.29458941 | -1.29640655 | 0.371945751 | -3.4854721 | 0.000491269 | 0.004595761 |
| APOBEC3A | 231.5078457 | -2.65510184 | 0.761832163 | -3.4851533 | 0.000491855 | 0.004599734 |
| GLT1D1 | 104.2458099 | -1.31333471 | 0.377121174 | -3.4825271 | 0.000496705 | 0.004639003 |
| CAPNS2 | 28.14254169 | 2.003108723 | 0.575259571 | 3.48209543 | 0.000497506 | 0.004644967 |
| E2F7 | 50.82539156 | 1.285516365 | 0.369470411 | 3.47934862 | 0.000502634 | 0.004682647 |
| TMEM52 | 113.9519287 | 1.666810748 | 0.479557174 | 3.47572894 | 0.000509467 | 0.004731855 |
| AC111182.1 | 65.52144758 | 1.04233315 | 0.300087256 | 3.47343358 | 0.000513845 | 0.004767855 |
| GPER1 | 696.5279525 | -1.044265302 | 0.30074936 | -3.4722112 | 0.00051619 | 0.004784949 |
| DCAF12L1 | 2.929554924 | 4.069699178 | 1.172923243 | 3.46970631 | 0.000521028 | 0.004823523 |
| CLEC4E | 621.675266 | -1.569381597 | 0.452436223 | -3.4687355 | 0.000522914 | 0.004836994 |
| SP6 | 659.2500876 | -1.353678722 | 0.390266406 | -3.4686017 | 0.000523174 | 0.004837117 |
| AC008870.5 | 6.841859886 | 3.689967211 | 1.064043653 | 3.46787202 | 0.000524597 | 0.004848698 |
| DLK2 | 122.1487097 | 1.169021527 | 0.337128099 | 3.46758852 | 0.000525151 | 0.004852243 |
| AC020651.2 | 3.323887331 | -4.649702645 | 1.341061919 | -3.4671797 | 0.00052595 | 0.004858056 |
| ATP4B | 19.54348013 | 3.478739723 | 1.003527032 | 3.46651322 | 0.000527256 | 0.004865389 |
| ERICH4 | 50.12516857 | -1.31088392 | 0.378186684 | -3.466235 | 0.000527802 | 0.004865704 |
| RGS13 | 104.2826918 | 1.691583032 | 0.488132264 | 3.46541943 | 0.000529405 | 0.004878909 |
| AL133304.2 | 12.78787826 | 3.086087524 | 0.890801064 | 3.46439587 | 0.000531424 | 0.00489435 |
| ARL9 | 34.60094714 | 1.578722901 | 0.456080893 | 3.46149756 | 0.000537179 | 0.004942567 |
| LEMD1 | 13.87284632 | 1.822409628 | 0.527566483 | 3.45436962 | 0.000551581 | 0.005049085 |
| IL18RAP | 286.6438821 | -1.307749815 | 0.378610038 | -3.4540812 | 0.000552171 | 0.0050528 |
| P2RY6 | 95.38939953 | 1.110213441 | 0.321546603 | 3.4527295 | 0.000554945 | 0.005074928 |
| AVPR1A | 133.3795346 | 1.787391889 | 0.517798344 | 3.45190731 | 0.000556639 | 0.005088786 |
| AL121992.1 | 22.92632641 | 1.701854949 | 0.493150706 | 3.4509835 | 0.000558548 | 0.0051046 |
| SLC4A5 | 332.7831821 | 1.070666737 | 0.310401351 | 3.44929793 | 0.000562046 | 0.00513345 |
| S100A2 | 73.43572168 | 1.615878445 | 0.468647609 | 3.4479605 | 0.000564837 | 0.005153818 |
| LINC01488 | 12.91271134 | -3.282016791 | 0.953202937 | -3.4431459 | 0.000574989 | 0.00523306 |
| TRGV9 | 30.60991971 | -1.785153879 | 0.518881298 | -3.4403897 | 0.000580877 | 0.005283278 |
| LINC00656 | 55.96975382 | -1.433408365 | 0.416761838 | -3.4393945 | 0.000583017 | 0.005295985 |
| ODAM | 105.9772286 | -1.379619403 | 0.401416514 | -3.4368775 | 0.000588462 | 0.005333553 |
| F2RL2 | 65.91714946 | 1.474652338 | 0.429055919 | 3.43697004 | 0.000588261 | 0.005333553 |
| TGFBR3L | 60.01299893 | 1.815505202 | 0.528413792 | 3.43576422 | 0.000590885 | 0.005350416 |
| RNF144A-AS1 | 31.19248537 | -1.179907978 | 0.343490696 | -3.4350508 | 0.000592443 | 0.00536282 |
| AC024361.1 | 40.1744116 | 1.126094895 | 0.328076927 | 3.43241113 | 0.00059824 | 0.005405004 |
| ZNF366 | 1374.709715 | -1.214186239 | 0.35385433 | -3.4313166 | 0.000600659 | 0.005416566 |
| RNASEH2B-AS1 | 52.60504562 | 1.016965947 | 0.296363588 | 3.43148075 | 0.000600296 | 0.005416566 |
| AP000941.1 | 87.28723274 | -1.251482416 | 0.36479788 | -3.4306187 | 0.000602207 | 0.005427089 |
| PDE11A | 20.89366446 | 1.710927762 | 0.498747243 | 3.43045056 | 0.00060258 | 0.005428647 |
| CADM2 | 15.87408278 | 1.879896683 | 0.548015814 | 3.43036941 | 0.00060276 | 0.005428647 |
| DPP6 | 430.1390829 | -1.64981526 | 0.481112137 | -3.4291699 | 0.00060543 | 0.005449256 |
| LINC00535 | 58.01865316 | 1.024993247 | 0.299153397 | 3.42631325 | 0.000611834 | 0.005501687 |
| NTRK2 | 234.9880414 | 1.425473147 | 0.416113886 | 3.42568031 | 0.000613262 | 0.005512784 |
| OR2A42 | 39.67494919 | -1.303015843 | 0.380494121 | -3.4245361 | 0.00061585 | 0.00553 |
| COL17A1 | 95.54577523 | 2.749785417 | 0.802976403 | 3.42449094 | 0.000615952 | 0.00553 |
| TFF3 | 370.3941953 | 1.079912297 | 0.31574705 | 3.42018175 | 0.000625793 | 0.005607759 |
| RBM20 | 447.9096802 | 1.136565657 | 0.332523304 | 3.41800302 | 0.000630824 | 0.005644165 |
| SLC5A1 | 383.4655593 | -1.385159731 | 0.40525518 | -3.4179939 | 0.000630845 | 0.005644165 |
| AWAT2 | 53.78353842 | -2.449853108 | 0.717183119 | -3.4159381 | 0.000635627 | 0.005670938 |
| LRRK2 | 4617.043761 | -1.040218527 | 0.304663639 | -3.4143179 | 0.000639419 | 0.005694084 |
| AC020895.2 | 3.715077474 | -4.810339144 | 1.409280435 | -3.41333 | 0.000641742 | 0.005709421 |
| GABRP | 57.41993704 | 2.173764163 | 0.636902018 | 3.4130276 | 0.000642454 | 0.005712249 |
| MYBPC3 | 79.07361676 | -1.141496208 | 0.334452922 | -3.4130251 | 0.00064246 | 0.005712249 |
| AC078905.1 | 4.738021931 | -5.159838864 | 1.511907136 | -3.4128014 | 0.000642988 | 0.005715157 |
| KRT42P | 72.4128261 | 1.124173902 | 0.329414058 | 3.41264702 | 0.000643352 | 0.005716615 |
| CCDC190 | 118.7252765 | 1.760012482 | 0.515965903 | 3.41110231 | 0.000647008 | 0.005741945 |
| GZMH | 574.66645 | -1.411006651 | 0.413909061 | -3.4089774 | 0.000652069 | 0.005783257 |
| PSAPL1 | 34.92760631 | -1.874669743 | 0.550219758 | -3.4071291 | 0.000656501 | 0.005815332 |
| BTNL9 | 14813.51815 | -1.094242079 | 0.321191987 | -3.4068162 | 0.000657254 | 0.005820194 |
| AL160408.3 | 131.6414296 | 1.751495913 | 0.514202983 | 3.40623445 | 0.000658656 | 0.005828993 |
| AC236972.3 | 49.06205574 | -1.360829323 | 0.399521935 | -3.4061442 | 0.000658874 | 0.005829111 |
| ERVMER34-1 | 47.39134721 | -1.51875938 | 0.44600649 | -3.4052405 | 0.000661058 | 0.005846622 |
| SLC15A1 | 31.77744172 | 2.21970553 | 0.651939046 | 3.40477464 | 0.000662187 | 0.005851163 |
| PDE9A-AS1 | 7.219279611 | -2.70710536 | 0.795413407 | -3.4033942 | 0.000665542 | 0.005871714 |
| ODF3B | 8257.507459 | 1.076119405 | 0.316325969 | 3.4019319 | 0.000669113 | 0.005895926 |
| AC124947.2 | 7.144448995 | 2.860090749 | 0.841119688 | 3.40033742 | 0.000673027 | 0.005928586 |
| SLC6A3 | 41.05055836 | 1.445502767 | 0.425139262 | 3.40006887 | 0.000673689 | 0.00593258 |
| LINC00589 | 15.79481952 | 2.887495758 | 0.849304702 | 3.39983489 | 0.000674266 | 0.005935826 |
| ANGPTL6 | 177.1260778 | -1.072289936 | 0.315499401 | -3.3987067 | 0.000677053 | 0.005954849 |
| PHOSPHO1 | 130.4674953 | -1.60487406 | 0.472322111 | -3.3978381 | 0.000679206 | 0.005971946 |
| AC004556.2 | 100.4235828 | 1.399456668 | 0.411885002 | 3.39768785 | 0.000679579 | 0.005973385 |
| CELP | 34.07971382 | 1.001196565 | 0.294731909 | 3.39697378 | 0.000681355 | 0.005985306 |
| AC084024.4 | 39.49607388 | -1.062676332 | 0.312888412 | -3.3963429 | 0.000682928 | 0.005997273 |
| CCDC184 | 135.0304282 | 1.262481191 | 0.371758687 | 3.39596958 | 0.00068386 | 0.00600361 |
| C9orf50 | 44.02979829 | 1.245854249 | 0.366914625 | 3.39548812 | 0.000685063 | 0.006010479 |
| HLA-V | 88.15950167 | -1.936916913 | 0.570531705 | -3.394933 | 0.000686454 | 0.006020826 |
| BICRA-AS1 | 12.51582017 | 2.650412703 | 0.780727007 | 3.39480084 | 0.000686785 | 0.006021142 |
| RASL10A | 91.60964752 | -1.116426009 | 0.329120051 | -3.3921543 | 0.000693454 | 0.006069156 |
| LINC01927 | 41.7310539 | 2.615736132 | 0.77192432 | 3.38859143 | 0.000702526 | 0.006140251 |
| PPIAP74 | 6.95547533 | 2.854459152 | 0.842767325 | 3.38700738 | 0.000706595 | 0.006170619 |
| AL157871.2 | 35.99114423 | -1.538833319 | 0.454358796 | -3.3868241 | 0.000707067 | 0.006171259 |
| ACE | 11222.1749 | -1.083261562 | 0.319858221 | -3.3866929 | 0.000707405 | 0.006172322 |
| TNF | 9.597463109 | -2.310431657 | 0.682553879 | -3.3849806 | 0.000711833 | 0.006205259 |
| LINC01644 | 43.34191244 | 1.689617275 | 0.499375583 | 3.38345993 | 0.000715787 | 0.006232106 |
| AC092620.1 | 3.69434167 | -3.805795065 | 1.125006952 | -3.382908 | 0.000717226 | 0.006242737 |
| LINC00271 | 99.81782455 | 1.342986141 | 0.397002246 | 3.38281749 | 0.000717463 | 0.006242891 |
| AC007998.2 | 4.649288535 | -5.130220309 | 1.516635967 | -3.3826313 | 0.000717949 | 0.006245219 |
| IL13RA2 | 50.91185567 | 2.172949355 | 0.642581136 | 3.38159531 | 0.000720662 | 0.006263087 |
| GPR4 | 683.2480305 | -1.117201457 | 0.330631649 | -3.3789913 | 0.000727523 | 0.006307338 |
| C8orf37-AS1 | 38.89303875 | 1.461489025 | 0.432619161 | 3.37823462 | 0.000729528 | 0.006322799 |
| RAPH1 | 386.6922088 | -1.179840336 | 0.349262106 | -3.3780943 | 0.0007299 | 0.006324104 |
| ZNF295-AS1 | 44.3040611 | 2.300136649 | 0.680978776 | 3.37769212 | 0.000730969 | 0.006331437 |
| AP000438.1 | 26.93919089 | -1.399520462 | 0.414656602 | -3.3751313 | 0.000737805 | 0.006379027 |
| CLTRN | 36.09769485 | 1.119279632 | 0.331853944 | 3.37280799 | 0.000744058 | 0.006421414 |
| ELF5 | 133.7123208 | 1.392769865 | 0.41298493 | 3.37244718 | 0.000745034 | 0.006427888 |
| CLIC3 | 1964.706188 | -1.071436749 | 0.317733689 | -3.372122 | 0.000745914 | 0.006433537 |
| ALMS1P1 | 130.8646977 | 1.011146605 | 0.299889135 | 3.37173471 | 0.000746964 | 0.006440642 |
| LINC01230 | 23.01464091 | -2.641823592 | 0.784003703 | -3.369657 | 0.000752618 | 0.006476286 |
| LINC01700 | 7.524000683 | -4.845116526 | 1.437887166 | -3.3696083 | 0.000752751 | 0.006476286 |
| SOX21-AS1 | 28.63343016 | 2.005854185 | 0.595359342 | 3.36914875 | 0.000754007 | 0.006480698 |
| B3GNT3 | 79.2849814 | 3.276620687 | 0.972706496 | 3.36856051 | 0.000755618 | 0.006489761 |
| ITLN2 | 1849.741335 | -1.227639853 | 0.364843925 | -3.3648357 | 0.000765893 | 0.006566142 |
| AC025048.7 | 5.588040588 | -5.394957817 | 1.605350176 | -3.3606112 | 0.000777702 | 0.006645416 |
| AC007497.1 | 9.272953785 | 2.209841811 | 0.658405498 | 3.35635383 | 0.000789775 | 0.00673848 |
| LINC01050 | 8.496160202 | 5.098351463 | 1.519285024 | 3.35575707 | 0.000791481 | 0.006748999 |
| CNGA1 | 42.41350489 | 1.490066073 | 0.44465029 | 3.3510966 | 0.000804922 | 0.006842672 |
| KCNK2 | 14.40302323 | 3.797265916 | 1.134812011 | 3.34616296 | 0.000819382 | 0.006935086 |
| RSPO2 | 806.0468067 | -1.035841374 | 0.309641424 | -3.3452933 | 0.000821956 | 0.006952744 |
| HNRNPA1P54 | 17.94522426 | 1.486462237 | 0.444456239 | 3.34445128 | 0.000824455 | 0.00696562 |
| AOC1 | 23.9184169 | 2.135574087 | 0.638605997 | 3.34411844 | 0.000825445 | 0.006971917 |
| FOLR3 | 166.9329232 | -2.020837109 | 0.604390021 | -3.3435977 | 0.000826996 | 0.006982947 |
| OR7E47P | 81.6141486 | 1.139107423 | 0.340721003 | 3.34322631 | 0.000828103 | 0.006990231 |
| STEAP2 | 291.786271 | 1.095696074 | 0.327809905 | 3.34247397 | 0.000830351 | 0.00700299 |
| AL445489.1 | 12.49145487 | 2.946403577 | 0.88174235 | 3.34156976 | 0.000833061 | 0.007021688 |
| LILRA2 | 54.17095863 | -1.185733754 | 0.354934791 | -3.3407087 | 0.000835648 | 0.00703726 |
| FOXN1 | 30.40901457 | 1.6456563 | 0.492643658 | 3.34045973 | 0.000836398 | 0.007041494 |
| MRAP2 | 76.53251871 | 1.075375068 | 0.322330815 | 3.33624654 | 0.000849178 | 0.00714487 |
| CLDN3 | 2748.081647 | 1.121980383 | 0.336510221 | 3.33416435 | 0.000855561 | 0.007192209 |
| AC016813.2 | 4.006041584 | -4.91952486 | 1.476676663 | -3.3314841 | 0.000863842 | 0.007249006 |
| LONRF3 | 337.0524892 | -1.193566212 | 0.3582891 | -3.3312937 | 0.000864434 | 0.007251337 |
| COMP | 44.61079801 | 2.62822133 | 0.788994331 | 3.33110293 | 0.000865026 | 0.007252539 |
| AC079610.2 | 5.075733402 | 3.67972955 | 1.104793064 | 3.33069574 | 0.000866292 | 0.007261022 |
| AC104581.3 | 17.03603421 | -1.721762103 | 0.51757106 | -3.3266197 | 0.000879062 | 0.007352932 |
| AC121338.2 | 33.29678431 | 1.221568587 | 0.367428517 | 3.32464284 | 0.000885319 | 0.007398753 |
| IHH | 157.8034262 | 1.176468499 | 0.354051141 | 3.3228773 | 0.000890941 | 0.007432673 |
| CFC1B | 25.10166969 | 1.647488119 | 0.495885532 | 3.32231536 | 0.000892737 | 0.007443305 |
| SLCO4A1 | 505.6389732 | -1.413860923 | 0.425673022 | -3.3214718 | 0.00089544 | 0.007463661 |
| AC011816.2 | 7.059094213 | -2.525263705 | 0.760793434 | -3.3192501 | 0.000902595 | 0.007518905 |
| AC010255.3 | 13.49895726 | 2.79275826 | 0.841439145 | 3.31902584 | 0.000903321 | 0.007522749 |
| ADORA2B | 225.1624019 | 1.086236972 | 0.32758676 | 3.31587568 | 0.000913565 | 0.007592542 |
| AL137847.1 | 25.36128153 | 2.611118738 | 0.787928195 | 3.31390443 | 0.000920029 | 0.007632926 |
| AC112206.2 | 100.5415354 | -1.058695787 | 0.319573212 | -3.3128427 | 0.000923529 | 0.007653056 |
| Z84468.1 | 3.31814666 | -4.650350332 | 1.403699941 | -3.3129234 | 0.000923263 | 0.007653056 |
| PLA2G1B | 266.6598876 | -1.341219882 | 0.404946767 | -3.3120894 | 0.00092602 | 0.007669238 |
| LINC01659 | 11.24844658 | 1.894957427 | 0.572230742 | 3.31152678 | 0.000927884 | 0.007682445 |
| TMEM40 | 41.35708244 | 1.424721429 | 0.430257595 | 3.31132198 | 0.000928563 | 0.00768584 |
| LIF-AS1 | 10.80773174 | 3.8061184 | 1.149788773 | 3.31027619 | 0.00093204 | 0.007707905 |
| SLCO4A1-AS1 | 35.84345774 | -2.125731862 | 0.642228312 | -3.3099317 | 0.000933187 | 0.007712924 |
| GPBAR1 | 105.5278714 | -1.043967185 | 0.315445258 | -3.3095035 | 0.000934616 | 0.007721658 |
| ICAM1 | 17745.77299 | -1.067882287 | 0.322789039 | -3.3082979 | 0.000938649 | 0.007751329 |
| SLC38A4 | 14.9095872 | 1.77458018 | 0.536701895 | 3.3064541 | 0.000944848 | 0.00779575 |
| AC087672.2 | 8.530831575 | 1.820649607 | 0.550831848 | 3.30527295 | 0.000948839 | 0.007826416 |
| LINC02204 | 11.5355295 | -2.465119304 | 0.746411013 | -3.3026299 | 0.000957827 | 0.007889144 |
| RHBDL2 | 135.9341505 | 1.019811701 | 0.308966388 | 3.30072053 | 0.000964369 | 0.007937366 |
| AL589765.7 | 49.38327334 | 2.610585323 | 0.790924079 | 3.30067752 | 0.000964517 | 0.007937366 |
| CDKN2A | 120.4925541 | 2.078351276 | 0.630122743 | 3.29832767 | 0.000972626 | 0.007997175 |
| AC005908.3 | 19.53240725 | 1.889035176 | 0.573218017 | 3.29549163 | 0.000982496 | 0.00806903 |
| LINC02535 | 3.937483975 | 4.459266255 | 1.354168707 | 3.29299166 | 0.000991274 | 0.008115417 |
| GRM3-AS1 | 29.61581592 | -1.303494475 | 0.395834814 | -3.2930264 | 0.000991152 | 0.008115417 |
| DRD1 | 51.38335702 | 1.358856856 | 0.412971753 | 3.29043536 | 0.001000325 | 0.008170752 |
| AL118558.4 | 26.27497777 | 1.332852208 | 0.405105984 | 3.29013211 | 0.001001404 | 0.008175915 |
| DUSP6 | 5067.579865 | -1.014570666 | 0.308402896 | -3.2897573 | 0.001002738 | 0.008178756 |
| HAMP | 11.99662147 | 2.679495017 | 0.814492398 | 3.2897729 | 0.001002683 | 0.008178756 |
| AL596442.2 | 53.0145667 | -1.872025086 | 0.569374796 | -3.2878608 | 0.001009517 | 0.008226388 |
| LRRC37A5P | 46.27247041 | 2.581483326 | 0.785266343 | 3.28739841 | 0.001011177 | 0.008235542 |
| MIR7515HG | 22.24185001 | 3.266734898 | 0.994825061 | 3.28372799 | 0.001024437 | 0.008327167 |
| BX323046.2 | 4.154721234 | -3.970166433 | 1.209898156 | -3.2814055 | 0.001032911 | 0.008386488 |
| MUC12-AS1 | 32.1999885 | 1.282226636 | 0.39092214 | 3.28000516 | 0.001038052 | 0.008425826 |
| LRRC37A11P | 15.99408498 | 1.963668094 | 0.598731168 | 3.27971584 | 0.001039117 | 0.008427469 |
| MGAT5B | 117.9442289 | -1.442933954 | 0.439975114 | -3.2795808 | 0.001039614 | 0.008428913 |
| AC010255.2 | 20.74466823 | 3.56867105 | 1.088503278 | 3.27851199 | 0.001043559 | 0.008452703 |
| AC025580.3 | 4.271718753 | 2.792872436 | 0.852083058 | 3.27769976 | 0.001046566 | 0.008473238 |
| AC011899.2 | 82.10558629 | -1.146648317 | 0.349989571 | -3.2762357 | 0.001052007 | 0.008511063 |
| AL139041.1 | 28.96831901 | -1.760485846 | 0.537507903 | -3.2752743 | 0.001055594 | 0.008534215 |
| AC093904.4 | 16.37196958 | 2.628405682 | 0.802653685 | 3.27464476 | 0.001057949 | 0.008548409 |
| SMIM2-AS1 | 145.7694569 | 1.094115891 | 0.334489263 | 3.27100452 | 0.001071662 | 0.008639631 |
| DLX3 | 51.08196785 | 1.306523167 | 0.399450244 | 3.27080328 | 0.001072425 | 0.008639785 |
| B3GALT5-AS1 | 21.53094959 | 3.097507332 | 0.947509586 | 3.26910395 | 0.001078887 | 0.0086807 |
| AMER2 | 114.7198245 | -2.73719606 | 0.83763012 | -3.2677861 | 0.001083923 | 0.008706505 |
| ALDH3A1 | 171.967314 | 1.330783637 | 0.407261052 | 3.26764278 | 0.001084471 | 0.008708314 |
| COL22A1 | 276.8646971 | -1.35173431 | 0.413879185 | -3.2660118 | 0.001090737 | 0.008751362 |
| DMBX1 | 8.006700504 | 2.967046271 | 0.908545015 | 3.2657119 | 0.001091893 | 0.008755709 |
| AC006116.9 | 17.25192567 | 1.852028913 | 0.567297899 | 3.26464969 | 0.001095995 | 0.008784951 |
| SLC2A3P4 | 6.827325655 | -2.099499059 | 0.643281204 | -3.2637345 | 0.001099541 | 0.008802589 |
| MIR429 | 28.29100906 | 1.091518332 | 0.33465341 | 3.26163816 | 0.001107704 | 0.008855115 |
| DUSP8 | 329.1150344 | -1.288828861 | 0.395462682 | -3.2590404 | 0.001117897 | 0.008924094 |
| UNC79 | 18.74329098 | 1.033374885 | 0.317368088 | 3.25607686 | 0.001129632 | 0.009005166 |
| GPR17 | 610.9186973 | -1.012681121 | 0.311262845 | -3.2534597 | 0.001140089 | 0.009070783 |
| AC079145.1 | 65.68772128 | 1.018771178 | 0.314185108 | 3.24258264 | 0.001184516 | 0.009374982 |
| IGHD | 2362.555729 | 3.05078163 | 0.940852952 | 3.24257008 | 0.001184568 | 0.009374982 |
| FCRL6 | 199.4774084 | -1.222778284 | 0.377161257 | -3.242057 | 0.001186703 | 0.009388693 |
| TEK | 3267.097228 | -1.040014233 | 0.320794468 | -3.2419955 | 0.001186959 | 0.009388693 |
| LINC02212 | 11.52804128 | 2.596421789 | 0.801006703 | 3.24144827 | 0.00118924 | 0.009396315 |
| SLC52A1 | 71.70232591 | 1.451944908 | 0.447916621 | 3.24155175 | 0.001188808 | 0.009396315 |
| CIB4 | 16.36871772 | -1.476513591 | 0.455577098 | -3.2409741 | 0.00119122 | 0.009406746 |
| AC005532.2 | 13.03169323 | 2.330156191 | 0.719300526 | 3.23947517 | 0.001197499 | 0.009443258 |
| GVINP1 | 754.5537624 | -1.084542957 | 0.334892744 | -3.2384785 | 0.001201691 | 0.009473694 |
| AC083837.1 | 72.1270186 | 1.107118758 | 0.342126723 | 3.23599031 | 0.001212215 | 0.009546739 |
| AL021396.1 | 9.993514315 | 2.167708985 | 0.669876812 | 3.23598152 | 0.001212252 | 0.009546739 |
| ARMC4P1 | 12.76084291 | 3.562168933 | 1.101050088 | 3.23524695 | 0.001215376 | 0.009560441 |
| LINC00326 | 6.37528867 | 3.089769538 | 0.955306449 | 3.23432291 | 0.001219315 | 0.009586142 |
| AC079140.6 | 38.45442548 | 1.315120056 | 0.406686642 | 3.23374293 | 0.001221794 | 0.009600337 |
| FCGR1CP | 108.203747 | -1.04206207 | 0.322336916 | -3.232835 | 0.001225683 | 0.009626019 |
| HS3ST2 | 54.59163836 | 2.503718753 | 0.774985773 | 3.23066415 | 0.00123503 | 0.009685019 |
| LINC01290 | 39.12989149 | -1.238869476 | 0.38352997 | -3.2301764 | 0.001237138 | 0.009696867 |
| AC018629.1 | 19.66307113 | 2.255633854 | 0.698416374 | 3.22964057 | 0.001239459 | 0.00971239 |
| USH1C | 52.95283156 | 1.423788223 | 0.441395529 | 3.22565166 | 0.001256862 | 0.009832551 |
| AC147067.2 | 25.37533937 | -1.220806789 | 0.378515073 | -3.2252528 | 0.001258615 | 0.009843563 |
| AL590226.1 | 135.9706353 | -1.323270948 | 0.410496261 | -3.2235883 | 0.001265953 | 0.009890103 |
| AC110285.6 | 28.0397916 | -1.365599018 | 0.423679958 | -3.2231853 | 0.001267735 | 0.009901317 |
| LTK | 716.1062337 | -1.442066367 | 0.447466685 | -3.2227346 | 0.001269731 | 0.00991148 |
| AC068860.1 | 21.59318773 | 1.755286065 | 0.544774619 | 3.22204083 | 0.00127281 | 0.009930076 |
| LRRIQ3 | 54.48491221 | 2.065801163 | 0.641534271 | 3.22009479 | 0.001281482 | 0.009986808 |
| AC074325.1 | 3.151156228 | 5.063821378 | 1.572861167 | 3.21949673 | 0.001284158 | 0.010004929 |
| ALX1 | 8.229108457 | 4.12503826 | 1.282488117 | 3.2164339 | 0.001297944 | 0.010093596 |
| TRGV4 | 57.66282351 | -1.10846257 | 0.344942294 | -3.2134725 | 0.001311404 | 0.010178276 |
| XPNPEP2 | 39.99767191 | -1.284844742 | 0.400072739 | -3.2115278 | 0.001320312 | 0.010236274 |
| RPL21P54 | 9.436887575 | 2.660749075 | 0.828625158 | 3.21104066 | 0.001322552 | 0.010250858 |
| AC005041.4 | 21.18300538 | 1.962024747 | 0.611049029 | 3.21091214 | 0.001323144 | 0.010252657 |
| PTPN22 | 256.8211208 | -1.046712547 | 0.326033762 | -3.2104422 | 0.001325309 | 0.010266647 |
| HECW1 | 26.24106125 | 1.664257819 | 0.518598712 | 3.20914376 | 0.001331309 | 0.010307527 |
| LINC02618 | 32.27590099 | 1.902180461 | 0.592795655 | 3.20882996 | 0.001332763 | 0.010315982 |
| PCAT6 | 54.89478621 | 1.051773359 | 0.327855823 | 3.20803623 | 0.001336447 | 0.010336081 |
| KSR2 | 72.00543036 | 1.098067837 | 0.342583779 | 3.20525344 | 0.001349436 | 0.010413951 |
| PNMA6B | 24.91952007 | 1.407737345 | 0.439187774 | 3.20531997 | 0.001349124 | 0.010413951 |
| NR4A2 | 1525.865444 | 1.531471046 | 0.478128964 | 3.20305014 | 0.001359803 | 0.01046564 |
| LINC02832 | 11.61121018 | 2.625815179 | 0.819756956 | 3.20316304 | 0.00135927 | 0.01046564 |
| COL10A1 | 52.4331765 | 3.224614039 | 1.006778095 | 3.20290445 | 0.001360491 | 0.010468111 |
| AL137786.2 | 19.10275455 | 2.984526155 | 0.931992716 | 3.20230631 | 0.001363319 | 0.010487045 |
| LINC02432 | 91.90267002 | -1.348482641 | 0.421475065 | -3.1994363 | 0.001376966 | 0.010569214 |
| AL354822.1 | 25.05144559 | -2.478970366 | 0.77480993 | -3.1994561 | 0.001376871 | 0.010569214 |
| UTF1 | 23.64548442 | 2.684951023 | 0.839437369 | 3.19851263 | 0.001381385 | 0.01060028 |
| AL160314.2 | 26.95325744 | 1.080367914 | 0.337784151 | 3.19839729 | 0.001381938 | 0.010601669 |
| MEOX1 | 152.5688774 | 1.619734046 | 0.506600548 | 3.19726074 | 0.001387395 | 0.010640671 |
| AP000757.2 | 5.44094074 | -4.372356964 | 1.3676382 | -3.1970129 | 0.001388587 | 0.010644094 |
| RN7SL475P | 17.40934502 | 2.047703093 | 0.641129145 | 3.19390112 | 0.001403642 | 0.010735866 |
| MFSD6L | 62.1119631 | 1.478711444 | 0.463917801 | 3.18744278 | 0.001435369 | 0.010952653 |
| AL121899.4 | 18.75349623 | 2.746590944 | 0.861969622 | 3.18641269 | 0.00144049 | 0.01097998 |
| USP6 | 217.8444294 | 1.40991186 | 0.443148752 | 3.18157696 | 0.001464756 | 0.011138159 |
| MIR6814 | 3.194287513 | 1.801948475 | 0.566735903 | 3.1795206 | 0.001475189 | 0.011205542 |
| IGKV3D-11 | 14.22305903 | 2.973895698 | 0.935368617 | 3.17938366 | 0.001475886 | 0.011207853 |
| AC093484.2 | 6.511373432 | -2.576926135 | 0.811138253 | -3.1769259 | 0.001488451 | 0.011285241 |
| TMC3 | 17.31428108 | 2.307072085 | 0.726843688 | 3.17409661 | 0.001503037 | 0.011374665 |
| AC093866.1 | 15.97675321 | 3.01341936 | 0.949579936 | 3.17342358 | 0.001506526 | 0.011392001 |
| AC007920.2 | 41.07055381 | -1.182927329 | 0.372979183 | -3.1715639 | 0.001516205 | 0.011459118 |
| GHRHR | 6.710238143 | -2.726439347 | 0.860142801 | -3.169752 | 0.001525691 | 0.011515556 |
| FAM86B2 | 76.36568415 | 1.391339466 | 0.439187224 | 3.16798712 | 0.001534983 | 0.011573441 |
| AL356417.3 | 24.38787573 | 1.71865793 | 0.542959553 | 3.16535167 | 0.001548956 | 0.011669539 |
| AC005962.1 | 58.97527208 | 1.050505224 | 0.331884964 | 3.16526911 | 0.001549396 | 0.011669769 |
| AC099063.4 | 25.94760079 | 1.047288599 | 0.331079506 | 3.16325408 | 0.001560161 | 0.011719903 |
| POU2AF1 | 476.4905374 | 1.594922356 | 0.504551164 | 3.1610716 | 0.001571899 | 0.011795649 |
| SH2D6 | 72.2260588 | 1.830061293 | 0.579339611 | 3.15887479 | 0.001583795 | 0.011866191 |
| CXCR1 | 85.42344783 | -2.643114774 | 0.837914297 | -3.1543975 | 0.001608299 | 0.012021357 |
| FXYD6-FXYD2 | 14.7455895 | -2.224925468 | 0.705634214 | -3.1530862 | 0.001615541 | 0.012066007 |
| AL391832.2 | 37.75542492 | 1.185762933 | 0.376422225 | 3.15008746 | 0.001632216 | 0.012174606 |
| ITGBL1 | 989.4769045 | 1.21028868 | 0.384397537 | 3.14853391 | 0.001640917 | 0.012229912 |
| TXK | 205.2528472 | -1.083385954 | 0.344400549 | -3.1457149 | 0.001656814 | 0.012327923 |
| LINC00471 | 33.08440206 | 1.140120041 | 0.36246232 | 3.14548569 | 0.001658113 | 0.012329082 |
| LINC01169 | 21.19613439 | -2.057246074 | 0.654132002 | -3.1450014 | 0.00166086 | 0.012338238 |
| AL662899.4 | 8.83548209 | 4.776805738 | 1.519204568 | 3.14428079 | 0.001664956 | 0.012363848 |
| CT45A10 | 113.6674506 | -3.988499637 | 1.268765087 | -3.1436077 | 0.00166879 | 0.012389095 |
| AC027281.1 | 4.262193093 | 2.59344814 | 0.82532903 | 3.14232027 | 0.001676146 | 0.012437229 |
| MT1L | 82.34215487 | -1.076930494 | 0.3427797 | -3.1417569 | 0.001679374 | 0.012457941 |
| LINC00381 | 14.80818506 | 2.972841726 | 0.946766487 | 3.13999467 | 0.001689509 | 0.012516849 |
| VWA5B1 | 32.84135194 | 1.697384777 | 0.541304879 | 3.13572784 | 0.001714282 | 0.012667479 |
| TCAP | 533.6902033 | -1.065836826 | 0.339964853 | -3.1351383 | 0.001717731 | 0.012689678 |
| AC004528.1 | 17.67958737 | 1.726989673 | 0.551069221 | 3.13388882 | 0.001725062 | 0.012737236 |
| LINC02600 | 95.74880111 | -1.027913885 | 0.328328009 | -3.130753 | 0.001743588 | 0.012840783 |
| LILRA5 | 37.90645933 | -1.11484439 | 0.356267568 | -3.1292334 | 0.00175263 | 0.012890734 |
| KCNIP1 | 77.12028232 | -1.158036337 | 0.370153056 | -3.1285338 | 0.001756808 | 0.012914804 |
| AC055876.4 | 4.506447565 | -3.558294958 | 1.137636696 | -3.1277955 | 0.001761227 | 0.01294395 |
| TRGJP2 | 9.12258264 | -1.461299481 | 0.467416934 | -3.1263298 | 0.001770029 | 0.013001944 |
| SFRP4 | 1298.269669 | 1.243767089 | 0.397868235 | 3.12607788 | 0.001771546 | 0.013009736 |
| STON1-GTF2A1L | 73.91297346 | -1.648121291 | 0.527743104 | -3.1229613 | 0.001790413 | 0.013128007 |
| DKK2 | 940.7795539 | -1.399960716 | 0.448786213 | -3.1194379 | 0.001811965 | 0.013258765 |
| AC010203.1 | 169.1904357 | 1.439892259 | 0.461569655 | 3.11955572 | 0.00181124 | 0.013258765 |
| MMP10 | 54.69997749 | 4.875328197 | 1.562958436 | 3.11929485 | 0.001812845 | 0.013261802 |
| RMDN2-AS1 | 4.620852921 | -3.663066271 | 1.175356694 | -3.1165571 | 0.001829762 | 0.013375272 |
| CLDN16 | 59.64877846 | 2.429645286 | 0.779960553 | 3.11508739 | 0.001838904 | 0.013428332 |
| AC012485.3 | 17.58930866 | 1.930126745 | 0.619829294 | 3.11396503 | 0.001845914 | 0.013465728 |
| PCAT18 | 2.967049263 | 5.189557649 | 1.666610948 | 3.11383869 | 0.001846704 | 0.01346805 |
| TTR | 55.80330701 | 3.293299385 | 1.058123012 | 3.11239747 | 0.001855745 | 0.013527065 |
| C1orf141 | 148.986912 | 1.579131836 | 0.507447793 | 3.11190995 | 0.001858812 | 0.013534705 |
| AC106820.1 | 5.675317745 | -3.425920914 | 1.100866007 | -3.1120235 | 0.001858097 | 0.013534705 |
| AC097493.3 | 16.4825071 | 2.062944396 | 0.663032116 | 3.1113793 | 0.001862156 | 0.013552554 |
| AC005840.3 | 9.941853509 | -2.886578897 | 0.928037858 | -3.1104107 | 0.001868274 | 0.013587142 |
| PTGDR2 | 108.7809562 | 1.209473604 | 0.388936676 | 3.10969286 | 0.00187282 | 0.013609793 |
| AL662899.3 | 7.354662841 | -5.305554042 | 1.706537024 | -3.1089592 | 0.001877476 | 0.013629741 |
| FAM181B | 31.17307459 | 1.601995017 | 0.515346861 | 3.10857626 | 0.001879911 | 0.013640475 |
| HNRNPA1P3 | 3.969273211 | 5.628306445 | 1.810769787 | 3.10823965 | 0.001882054 | 0.013652549 |
| PSPC1P1 | 3.481239729 | 4.538638566 | 1.460623922 | 3.10732866 | 0.001887864 | 0.01367383 |
| AL390816.2 | 17.27001204 | 2.506500962 | 0.806671299 | 3.10721476 | 0.001888591 | 0.013675627 |
| AC124798.1 | 91.38412228 | 1.172950395 | 0.37764699 | 3.10594398 | 0.001896726 | 0.013723796 |
| ARG1 | 56.88671778 | -2.648313753 | 0.853612655 | -3.1024771 | 0.001919084 | 0.013861237 |
| CHRM1 | 195.2955132 | -1.456213914 | 0.469478668 | -3.101768 | 0.001923686 | 0.013887449 |
| BMS1P7 | 37.44024789 | 1.446590316 | 0.466660629 | 3.09987649 | 0.001936014 | 0.013965836 |
| IGF2 | 12742.47284 | 1.091691017 | 0.352190737 | 3.0997153 | 0.001937067 | 0.013969904 |
| SOSTDC1 | 3378.479275 | -1.660157812 | 0.536152982 | -3.0964256 | 0.00195869 | 0.01410801 |
| CHRDL2 | 80.30906793 | 1.347854186 | 0.435739533 | 3.0932566 | 0.001979729 | 0.014237974 |
| GPAA1P2 | 40.6125558 | 1.299821169 | 0.420446324 | 3.09152701 | 0.001991299 | 0.01429595 |
| Z98752.4 | 11.74743977 | 1.813226161 | 0.58667243 | 3.09069605 | 0.001996879 | 0.014328802 |
| AC108053.1 | 100.5915217 | -1.01366484 | 0.328069217 | -3.0897896 | 0.002002984 | 0.014358156 |
| AL132712.1 | 5.201398909 | 2.594616736 | 0.83982906 | 3.08945815 | 0.00200522 | 0.014370572 |
| ACKR4P1 | 35.77176207 | 1.042644412 | 0.337497968 | 3.08933538 | 0.002006048 | 0.014372901 |
| ABAT | 844.5893167 | 1.012770763 | 0.327843602 | 3.08918874 | 0.002007039 | 0.014376386 |
| PPIHP1 | 5.100014712 | 2.672337016 | 0.8653065 | 3.08831266 | 0.002012966 | 0.014414204 |
| OR2S1P | 87.3120992 | -1.394669884 | 0.451838686 | -3.0866544 | 0.002024228 | 0.014477695 |
| KRT18P59 | 6.924454019 | 2.435228862 | 0.789415378 | 3.08485106 | 0.002036541 | 0.014554814 |
| SLC27A6 | 117.6796929 | 1.433303288 | 0.464918306 | 3.08291429 | 0.002049842 | 0.014635204 |
| SCGB2A1 | 43.22965992 | 2.24307224 | 0.727643364 | 3.08265333 | 0.00205164 | 0.014644377 |
| FEZF1-AS1 | 32.21314456 | 5.032728564 | 1.633719041 | 3.08053493 | 0.002066291 | 0.014733865 |
| AC008691.1 | 16.92585338 | 1.600528405 | 0.519648351 | 3.0800221 | 0.002069852 | 0.014752224 |
| DPEP1 | 215.0371752 | -1.261585864 | 0.409620541 | -3.0798892 | 0.002070776 | 0.014753122 |
| AC006058.4 | 106.7747462 | 1.164342972 | 0.378151301 | 3.07903998 | 0.002076688 | 0.014780237 |
| AC113410.5 | 58.79609127 | 1.03609015 | 0.33655516 | 3.07851513 | 0.00208035 | 0.014797001 |
| CR2 | 282.4839287 | 3.032441019 | 0.985863715 | 3.07592315 | 0.002098519 | 0.014900688 |
| VWC2L | 5.502717534 | 3.384656417 | 1.100685292 | 3.07504465 | 0.00210471 | 0.014937212 |
| AL121956.4 | 4.737070007 | 4.310027356 | 1.402294213 | 3.07355426 | 0.002115252 | 0.01500456 |
| C2orf91 | 29.46471809 | -1.156975249 | 0.376518733 | -3.0728225 | 0.002120445 | 0.015033921 |
| LINC02284 | 14.74361149 | -1.569955532 | 0.511029882 | -3.0721404 | 0.002125298 | 0.015049618 |
| FAM86B1 | 270.4790798 | 1.506663808 | 0.491259584 | 3.06694028 | 0.002162621 | 0.015283555 |
| PACRG-AS3 | 54.78462902 | -1.734697056 | 0.565726837 | -3.0663157 | 0.002167144 | 0.015304146 |
| AC024145.1 | 6.877651325 | 3.441854751 | 1.122505565 | 3.06622511 | 0.0021678 | 0.015304993 |
| GZMM | 78.76553236 | -1.75307453 | 0.572047892 | -3.064559 | 0.002179912 | 0.015382884 |
| AC068620.3 | 43.97131659 | 1.666571496 | 0.543858709 | 3.06434643 | 0.002181461 | 0.015386207 |
| LINC02280 | 4.366121019 | -4.046981216 | 1.321275707 | -3.0629347 | 0.002191779 | 0.015443702 |
| AC100793.4 | 9.554327674 | -3.553272117 | 1.160451201 | -3.0619746 | 0.002198821 | 0.015481849 |
| AP001189.3 | 24.52212342 | -1.814241103 | 0.592548488 | -3.0617597 | 0.0022004 | 0.015489143 |
| PCAT14 | 1248.59977 | -2.34457937 | 0.76588362 | -3.0612737 | 0.002203975 | 0.015506658 |
| MPIG6B | 15.82586101 | -1.425520207 | 0.465772635 | -3.0605495 | 0.002209312 | 0.015530813 |
| PEAK3 | 137.2264721 | -1.080506036 | 0.353139452 | -3.0597149 | 0.002215478 | 0.015564551 |
| CST1 | 3.790478115 | 5.346104501 | 1.747715671 | 3.05890975 | 0.002221441 | 0.015594918 |
| MIPEPP3 | 60.12029167 | 1.242989683 | 0.40638637 | 3.05864019 | 0.00222344 | 0.015601276 |
| AL589765.3 | 10.07991912 | 2.075560216 | 0.678640974 | 3.05840687 | 0.002225172 | 0.015609589 |
| LINC00885 | 50.51769475 | 1.089929787 | 0.356464912 | 3.0576075 | 0.002231116 | 0.015632061 |
| AL162586.2 | 168.9803528 | -1.528650388 | 0.500268862 | -3.0556577 | 0.002245675 | 0.01572248 |
| AC097532.3 | 15.9816497 | 2.474795482 | 0.809985484 | 3.05535782 | 0.002247922 | 0.015734348 |
| DDN | 5.053960381 | -3.717745771 | 1.216933271 | -3.055012 | 0.002250515 | 0.015745519 |
| AC003973.2 | 27.88668549 | 1.936085597 | 0.634126657 | 3.05315283 | 0.002264506 | 0.015823248 |
| WNT10A | 52.05186654 | 1.324526912 | 0.433894623 | 3.05264653 | 0.00226833 | 0.015846085 |
| CSMD2 | 29.02778027 | 1.671244724 | 0.547512949 | 3.05242959 | 0.00226997 | 0.015849779 |
| ODF3L1 | 81.45400699 | -1.368549469 | 0.448447959 | -3.0517465 | 0.002275142 | 0.015882005 |
| LINC02858 | 14.83215305 | 2.356738617 | 0.77263568 | 3.05025859 | 0.002286444 | 0.015945293 |
| CPSF1P1 | 34.44601221 | -1.38976446 | 0.455605121 | -3.0503706 | 0.002285592 | 0.015945293 |
| AC087164.2 | 3.231363964 | -4.608805195 | 1.511048196 | -3.0500716 | 0.002287868 | 0.015951323 |
| MIR1180 | 5.843778582 | 1.677848459 | 0.550172691 | 3.04967601 | 0.002290884 | 0.015964542 |
| CEACAM22P | 7.267482228 | -3.31764073 | 1.087918384 | -3.0495309 | 0.002291991 | 0.015968355 |
| AC109322.2 | 18.74394677 | 1.524745215 | 0.500046469 | 3.04920705 | 0.002294463 | 0.015977775 |
| CA4 | 6311.348533 | -1.114686865 | 0.365716803 | -3.0479509 | 0.002304076 | 0.016036883 |
| AL354714.1 | 119.717851 | -1.38857602 | 0.456465338 | -3.0420185 | 0.002349974 | 0.016316532 |
| LINC01747 | 7.566063936 | -3.435817755 | 1.130368384 | -3.0395558 | 0.002369273 | 0.016438526 |
| AL137140.1 | 19.54441944 | -2.210711362 | 0.727527908 | -3.0386619 | 0.002376314 | 0.016467345 |
| VWA8-AS1 | 12.32013323 | 1.999463509 | 0.658056121 | 3.03843919 | 0.002378071 | 0.01647552 |
| ABCB1 | 998.210351 | 1.772160108 | 0.583441736 | 3.0374243 | 0.002386093 | 0.016523073 |
| AC087442.1 | 3.583180984 | -4.254456572 | 1.401662717 | -3.0352927 | 0.002403024 | 0.016612081 |
| AL731567.1 | 13.19115663 | -1.201609308 | 0.395907649 | -3.0350747 | 0.002404761 | 0.016620061 |
| AL451166.1 | 4.140581529 | 4.170934451 | 1.374652415 | 3.03417388 | 0.002411954 | 0.01665488 |
| AC068896.3 | 93.88319757 | 1.490692475 | 0.491305926 | 3.03414308 | 0.0024122 | 0.01665488 |
| AC007496.3 | 6.866459581 | 3.185330163 | 1.050073766 | 3.03343467 | 0.002417871 | 0.016682371 |
| AC084782.3 | 11.23221594 | 2.066238433 | 0.681342926 | 3.03259688 | 0.002424593 | 0.016720661 |
| AC004066.1 | 4.745484995 | 3.696724765 | 1.219337881 | 3.03174766 | 0.002431424 | 0.016759666 |
| AC112229.4 | 8.596946214 | 3.174468128 | 1.047255212 | 3.03122686 | 0.002435622 | 0.016784547 |
| AL671277.2 | 12.26491709 | 1.155387357 | 0.381200515 | 3.03091762 | 0.002438118 | 0.016797688 |
| MRVI1-AS1 | 29.93514491 | 1.708429454 | 0.563934748 | 3.02948074 | 0.002449745 | 0.016861507 |
| NMBR | 8.494953834 | 2.80158717 | 0.924874913 | 3.02915252 | 0.002452408 | 0.016875765 |
| TMEM26 | 110.7386537 | -1.253357456 | 0.414052926 | -3.0270465 | 0.00246956 | 0.016981498 |
| CALCRL | 2897.623189 | -1.1425248 | 0.377458086 | -3.0268918 | 0.002470824 | 0.016982 |
| LRRC9 | 22.34778894 | 2.397552525 | 0.792081766 | 3.02690029 | 0.002470754 | 0.016982 |
| NPTX2 | 45.02757513 | 1.505673329 | 0.497799907 | 3.0246557 | 0.002489163 | 0.01707924 |
| MTND4P23 | 17.799957 | -2.06444443 | 0.682660228 | -3.024117 | 0.002493599 | 0.017102185 |
| BX649601.1 | 19.98369593 | 1.298045433 | 0.42924338 | 3.02403134 | 0.002494306 | 0.017102185 |
| GLIS1 | 36.52016576 | 2.017842694 | 0.667405444 | 3.0234136 | 0.002499404 | 0.017120677 |
| AC023424.2 | 7.542256945 | 2.612638226 | 0.864789502 | 3.02112621 | 0.002518364 | 0.017233994 |
| AC067752.1 | 30.77880672 | -2.722781542 | 0.901938643 | -3.0188102 | 0.002537695 | 0.017357955 |
| CASP5 | 50.31383684 | -1.235654041 | 0.409706214 | -3.0159514 | 0.002561743 | 0.017488893 |
| LRRC31 | 74.53915492 | -1.450970654 | 0.481111764 | -3.0158702 | 0.002562429 | 0.01748939 |
| CNFN | 23.79863374 | 1.781050364 | 0.591024172 | 3.01349835 | 0.002582544 | 0.017605621 |
| AC007193.2 | 68.38138871 | -1.019451879 | 0.338376677 | -3.0127723 | 0.002588731 | 0.017635149 |
| SMPD5 | 23.43417725 | -2.06972055 | 0.687082439 | -3.0123322 | 0.002592487 | 0.017656522 |
| SLC4A11 | 234.8910056 | 1.359413693 | 0.451334444 | 3.01198748 | 0.002595433 | 0.017672365 |
| FAM107A | 5812.27348 | -1.236080345 | 0.410676076 | -3.0098669 | 0.002613622 | 0.017774995 |
| AC124067.3 | 6.62978295 | 2.696614178 | 0.896520383 | 3.00786711 | 0.002630882 | 0.017868027 |
| SEMA3G | 6554.588271 | -1.697956018 | 0.56461202 | -3.007297 | 0.002635821 | 0.017896103 |
| LINC00939 | 70.71695518 | 1.639067732 | 0.54508149 | 3.00701411 | 0.002638275 | 0.017908501 |
| ANKRD20A8P | 12.47356335 | 2.170021638 | 0.722044106 | 3.00538654 | 0.002652435 | 0.017991775 |
| AC010478.1 | 6.020667177 | 3.519853289 | 1.171792341 | 3.00382002 | 0.00266613 | 0.018054612 |
| AC004834.1 | 10.87447959 | 1.84156481 | 0.61306238 | 3.00387835 | 0.002665619 | 0.018054612 |
| SERPINE1 | 4430.969855 | -1.366925587 | 0.455050013 | -3.0039019 | 0.002665413 | 0.018054612 |
| SAXO1 | 16.25014911 | 1.081679322 | 0.360329878 | 3.00191405 | 0.002682879 | 0.018159412 |
| AC007496.1 | 3.061751935 | 5.239208442 | 1.74541655 | 3.00169518 | 0.002684809 | 0.01816449 |
| AL162731.1 | 11.27083257 | 1.30481603 | 0.434762589 | 3.00121506 | 0.002689046 | 0.018179581 |
| AL159156.1 | 13.18400087 | -2.268392641 | 0.755856112 | -3.0010906 | 0.002690145 | 0.018182706 |
| ATP12A | 294.4946702 | 3.100750104 | 1.033367414 | 3.00062694 | 0.002694244 | 0.018197473 |
| MSX1 | 116.79299 | -1.149685539 | 0.383351179 | -2.9990401 | 0.002708317 | 0.018275213 |
| FAM90A2P | 10.96973693 | 1.512222898 | 0.504298118 | 2.99866854 | 0.002711621 | 0.018288857 |
| AC073862.5 | 86.27441804 | -1.036442476 | 0.345652254 | -2.9985121 | 0.002713014 | 0.018293921 |
| CNTN4-AS1 | 16.50680998 | 1.997408002 | 0.666511505 | 2.99680949 | 0.002728211 | 0.018370344 |
| AL449363.2 | 16.0391906 | 2.583246999 | 0.86221612 | 2.99605509 | 0.00273497 | 0.018411507 |
| RASAL2-AS1 | 38.42795138 | -1.334160943 | 0.445367972 | -2.9956374 | 0.002738719 | 0.018428678 |
| TAT-AS1 | 42.27451125 | 1.112013486 | 0.371245082 | 2.9953622 | 0.002741191 | 0.018431636 |
| TEX21P | 111.9733588 | 1.043085265 | 0.34835195 | 2.99434312 | 0.002750365 | 0.018484601 |
| AC096751.1 | 64.20017201 | -1.63458141 | 0.546079132 | -2.993305 | 0.002759738 | 0.018534495 |
| CYP24A1 | 26.36434669 | 3.01860597 | 1.008862921 | 2.99208734 | 0.00277077 | 0.018604206 |
| ALDH3B2 | 55.70015724 | 1.982411685 | 0.66261349 | 2.99180701 | 0.002773315 | 0.018612534 |
| CR559946.2 | 40.29813703 | 1.951598917 | 0.65231017 | 2.9918266 | 0.002773137 | 0.018612534 |
| CPB2 | 192.2020431 | -2.204417983 | 0.73721035 | -2.9902157 | 0.002787805 | 0.018687792 |
| IGHV2-5 | 50.571803 | 1.603485413 | 0.536268478 | 2.99007956 | 0.002789048 | 0.018691733 |
| BTG4 | 32.88974263 | 2.128430437 | 0.712337381 | 2.98795275 | 0.00280853 | 0.018800209 |
| Z82214.2 | 86.93335422 | 1.867925179 | 0.625140722 | 2.98800752 | 0.002808026 | 0.018800209 |
| TMPRSS4 | 251.2467319 | 2.814670136 | 0.942720949 | 2.98568748 | 0.002829416 | 0.018931138 |
| MIA | 4.151803316 | -3.974589637 | 1.331898707 | -2.9841531 | 0.002843645 | 0.019004321 |
| AC104162.1 | 82.09978489 | 1.527772716 | 0.512004027 | 2.98390762 | 0.002845927 | 0.019014812 |
| AC005703.7 | 14.18504788 | 2.376025996 | 0.796416985 | 2.98339443 | 0.002850704 | 0.019037841 |
| STRA6 | 19.41106421 | 1.398261268 | 0.468759504 | 2.98289689 | 0.002855342 | 0.01905989 |
| MAP1LC3C | 873.0977689 | -1.07242551 | 0.359682676 | -2.9815879 | 0.002867577 | 0.019123663 |
| CLEC4GP1 | 63.9769054 | -1.967754725 | 0.660568902 | -2.9788788 | 0.002893051 | 0.019257528 |
| RGR | 8.250643692 | -3.307500325 | 1.110623675 | -2.9780567 | 0.002900823 | 0.019295748 |
| AL034397.2 | 5.883686734 | -5.472316266 | 1.837683718 | -2.9778336 | 0.002902936 | 0.019305301 |
| PCDHA10 | 38.55360973 | -1.549999292 | 0.520757891 | -2.9764298 | 0.002916259 | 0.019371319 |
| RNF7P1 | 8.221280615 | 2.469030826 | 0.829575795 | 2.97625707 | 0.002917902 | 0.01937772 |
| AP002852.1 | 17.72885868 | 1.624743334 | 0.545989059 | 2.97578002 | 0.002922444 | 0.019401642 |
| AC233728.2 | 8.813341485 | 2.954565047 | 0.993126366 | 2.97501421 | 0.00292975 | 0.019442823 |
| AMD1P1 | 3.84607411 | -4.859564299 | 1.633656402 | -2.9746551 | 0.002933182 | 0.019456544 |
| SRSF3P6 | 3.923314891 | -4.889350978 | 1.644075236 | -2.9739217 | 0.002940201 | 0.019489509 |
| AC008555.1 | 61.81745704 | -1.013009117 | 0.340701792 | -2.9733014 | 0.00294615 | 0.019518201 |
| BX005214.2 | 7.817040351 | 2.752541032 | 0.9265355 | 2.97078853 | 0.002970362 | 0.019634681 |
| CDH16 | 23.85888821 | -1.94931821 | 0.656311505 | -2.9701113 | 0.002976919 | 0.019664348 |
| GP5 | 22.16304462 | 2.018319021 | 0.679569865 | 2.96999488 | 0.002978047 | 0.019667246 |
| AL021918.4 | 6.196025151 | 2.767454203 | 0.932297049 | 2.96842536 | 0.002993298 | 0.019751541 |
| AC006033.2 | 42.64763608 | -1.497650626 | 0.505450836 | -2.9629996 | 0.00304657 | 0.020040891 |
| FAM83A-AS1 | 4.172564807 | 3.784893466 | 1.277357106 | 2.96306604 | 0.003045912 | 0.020040891 |
| CCDC73 | 13.38848105 | 1.336317568 | 0.451029338 | 2.9628174 | 0.003048373 | 0.020048134 |
| AC068647.1 | 13.61346671 | 2.291582314 | 0.773629677 | 2.9621179 | 0.003055308 | 0.020079848 |
| AC093567.1 | 12.47868525 | 1.400202685 | 0.472846527 | 2.9612202 | 0.003064228 | 0.020115298 |
| CCL4L2 | 19.29387542 | -1.256286463 | 0.424257545 | -2.9611411 | 0.003065015 | 0.020115834 |
| CERNA1 | 43.85945728 | 1.27773445 | 0.431557817 | 2.96074917 | 0.003068918 | 0.020136816 |
| LRRTM4 | 86.38007724 | -1.154419164 | 0.389957154 | -2.9603744 | 0.003072654 | 0.020151942 |
| TMPRSS11CP | 6.454221697 | -1.954034899 | 0.660078828 | -2.9603054 | 0.003073342 | 0.020151942 |
| FSIP2-AS1 | 9.277393234 | 2.463625865 | 0.832773988 | 2.95833672 | 0.003093041 | 0.020270418 |
| OTOF | 133.5959428 | -2.183481704 | 0.7383838 | -2.9571094 | 0.003105379 | 0.020333958 |
| SEPTIN9-DT | 11.58991838 | -1.403408771 | 0.474634725 | -2.9568186 | 0.003108309 | 0.020343807 |
| CEACAM4 | 58.26410774 | -1.378825072 | 0.46631751 | -2.9568374 | 0.003108119 | 0.020343807 |
| AC005908.2 | 12.97130662 | 2.781793579 | 0.940959254 | 2.95633798 | 0.003113157 | 0.020370863 |
| AL096816.1 | 42.51653283 | -1.145054869 | 0.387571331 | -2.9544365 | 0.003132407 | 0.020478027 |
| AC009119.1 | 50.66525328 | 1.759188677 | 0.596022548 | 2.95154719 | 0.003161862 | 0.020628041 |
| AP002813.1 | 37.55510716 | 3.602969049 | 1.22161081 | 2.94935917 | 0.003184337 | 0.020760419 |
| VN2R17P | 10.51243045 | 2.53369952 | 0.859109054 | 2.94921757 | 0.003185796 | 0.020760444 |
| BTNL8 | 179.2944998 | -1.401204805 | 0.475213225 | -2.9485813 | 0.003192362 | 0.02079373 |
| ARL4AP2 | 19.65817969 | 1.178827931 | 0.399844735 | 2.94821422 | 0.003196155 | 0.020806247 |
| AC009754.2 | 22.99012228 | 2.748148504 | 0.93219192 | 2.94805012 | 0.003197852 | 0.02081048 |
| RNU6-343P | 24.15028129 | -1.079486773 | 0.366266897 | -2.9472682 | 0.00320595 | 0.020858422 |
| ALB | 23.72382441 | 1.043074745 | 0.353988137 | 2.94663757 | 0.003212495 | 0.020896235 |
| DPRX | 13.30194122 | -2.351711535 | 0.798313567 | -2.9458494 | 0.003220692 | 0.020936664 |
| AC097369.2 | 10.10635213 | 1.591901602 | 0.540391865 | 2.94582821 | 0.003220912 | 0.020936664 |
| AC021755.2 | 20.19682209 | 1.429881433 | 0.485585544 | 2.94465404 | 0.00323316 | 0.021011486 |
| AL449106.1 | 16.91390874 | 1.803748122 | 0.6127561 | 2.94366408 | 0.003243518 | 0.021064407 |
| AP001922.6 | 14.70760087 | 1.313043418 | 0.446514729 | 2.94064973 | 0.003275247 | 0.02122214 |
| AL731733.1 | 27.92409621 | 1.264913702 | 0.430306528 | 2.93956429 | 0.003286741 | 0.021286946 |
| NCF1B | 700.4496845 | -1.082567876 | 0.368689366 | -2.9362601 | 0.003321957 | 0.021471146 |
| IGKV3-11 | 283.9659515 | 2.188379609 | 0.745375961 | 2.93594068 | 0.003325379 | 0.021488397 |
| AC084346.1 | 9.026232887 | 2.250284249 | 0.766739168 | 2.93487583 | 0.003336812 | 0.021537879 |
| CCDC175 | 98.84172646 | 2.371066038 | 0.808158854 | 2.93391086 | 0.003347203 | 0.021585412 |
| AC138028.2 | 78.60408784 | -1.032348539 | 0.352064235 | -2.9322732 | 0.003364905 | 0.021679963 |
| GPR141 | 13.23487215 | -1.311511251 | 0.447317387 | -2.9319479 | 0.003368433 | 0.021692891 |
| RNU6-833P | 7.64190294 | 1.9217976 | 0.655667868 | 2.9310535 | 0.003378146 | 0.021740105 |
| AC097468.1 | 138.0500767 | 1.297068525 | 0.44269284 | 2.92995145 | 0.00339015 | 0.021788472 |
| TMEM132E | 43.10289375 | 1.124763196 | 0.38400086 | 2.92906427 | 0.003399841 | 0.021835999 |
| RPS27AP6 | 10.28540575 | 2.386709695 | 0.814909546 | 2.92880321 | 0.003402698 | 0.021844508 |
| AC079075.1 | 21.13302151 | 2.708353487 | 0.924769948 | 2.92867809 | 0.003404068 | 0.021848384 |
| PCDHGB3 | 79.57238796 | -2.243386299 | 0.766475758 | -2.9268849 | 0.003423756 | 0.021925413 |
| TCP11 | 28.90472253 | 2.521538223 | 0.861552291 | 2.92673846 | 0.003425368 | 0.021930813 |
| SNX31 | 10.76255415 | 2.129852569 | 0.727847994 | 2.92623266 | 0.003430942 | 0.021956646 |
| ALPK2 | 41.65906482 | 2.759508385 | 0.94308549 | 2.92604267 | 0.003433038 | 0.021961535 |
| AC013457.1 | 276.4920072 | -2.510126892 | 0.857987 | -2.9256001 | 0.003437925 | 0.021981604 |
| KIF26B-AS1 | 15.24321567 | 3.777384839 | 1.29125457 | 2.92536029 | 0.003440576 | 0.021993626 |
| AL357033.1 | 16.04359689 | -2.145970434 | 0.733666102 | -2.924996 | 0.003444606 | 0.022004592 |
| AL049757.2 | 27.77663364 | 1.397556312 | 0.477903448 | 2.92434867 | 0.003451779 | 0.022040538 |
| PCDHGA11 | 300.3576448 | -1.580438006 | 0.540645068 | -2.923245 | 0.003464038 | 0.022109008 |
| MIR642A | 4.491728346 | 2.103802342 | 0.719680777 | 2.92324376 | 0.003464052 | 0.022109008 |
| AC079035.1 | 13.40003991 | 2.513688791 | 0.860146434 | 2.92239634 | 0.003473492 | 0.022159342 |
| AC013268.3 | 9.352669815 | 1.781246795 | 0.609616229 | 2.92191498 | 0.003478865 | 0.022188654 |
| PTBP1P | 22.25472313 | 2.171472135 | 0.743472768 | 2.92071509 | 0.00349229 | 0.022256996 |
| AC108134.1 | 333.0192181 | 1.054518518 | 0.361052591 | 2.92067844 | 0.003492701 | 0.022256996 |
| ROPN1 | 4.489353358 | 2.728090427 | 0.934506674 | 2.91928405 | 0.003508364 | 0.02234682 |
| AL139353.2 | 43.84564978 | -1.861809776 | 0.637980786 | -2.918285 | 0.003519625 | 0.022408539 |
| LINC02408 | 37.32138759 | -1.494280829 | 0.512347269 | -2.9165391 | 0.003539384 | 0.022504201 |
| ADAM12 | 132.7362635 | 1.05402309 | 0.361407849 | 2.91643664 | 0.003540546 | 0.022506575 |
| AC129492.6 | 6.446844892 | -4.621031334 | 1.58514796 | -2.915205 | 0.003554549 | 0.022570438 |
| IFNG | 10.44174886 | -2.221385495 | 0.762372611 | -2.9137793 | 0.003570823 | 0.022648563 |
| AC005865.1 | 12.16072092 | 1.732042402 | 0.594619884 | 2.91285651 | 0.003581391 | 0.022700449 |
| MIR6757 | 16.65190536 | -1.006826044 | 0.345755376 | -2.9119606 | 0.003591679 | 0.02274544 |
| LINC00443 | 13.94211079 | -2.006755255 | 0.689138146 | -2.9119782 | 0.003591477 | 0.02274544 |
| VNN3 | 557.8404296 | 1.428870329 | 0.49101937 | 2.91000807 | 0.003614194 | 0.022847443 |
| AC024588.1 | 35.01047372 | -1.146464322 | 0.39428049 | -2.9077379 | 0.003640533 | 0.022988473 |
| GASAL1 | 38.96886588 | -1.27424835 | 0.438241342 | -2.9076407 | 0.003641665 | 0.022990219 |
| AC034139.1 | 34.66308533 | 2.153732484 | 0.740752587 | 2.90749235 | 0.003643393 | 0.022991258 |
| AC025774.1 | 168.3775224 | 1.455019491 | 0.500465113 | 2.9073345 | 0.003645232 | 0.02299269 |
| ITPRID1 | 16.91344872 | 2.596524553 | 0.893472024 | 2.90610616 | 0.003659573 | 0.023062746 |
| IGHV1-2 | 68.55652514 | 1.924485827 | 0.662744635 | 2.90381201 | 0.003686494 | 0.023222147 |
| CYP4F62P | 16.93145163 | 1.565353431 | 0.539138572 | 2.90343432 | 0.003690944 | 0.023245041 |
| POTEJ | 7.73459724 | 1.272227682 | 0.438319825 | 2.90251002 | 0.003701853 | 0.023293174 |
| LINC02231 | 13.19578417 | 2.31577483 | 0.797839733 | 2.90255641 | 0.003701305 | 0.023293174 |
| AC116366.3 | 342.756671 | -4.993174313 | 1.720649335 | -2.9019128 | 0.003708918 | 0.023332479 |
| INHBC | 7.469035859 | -2.748612771 | 0.947576822 | -2.9006754 | 0.003723594 | 0.023414477 |
| IGLJ3 | 135.3182309 | 1.701106863 | 0.586606365 | 2.89991204 | 0.003732674 | 0.023461229 |
| HBEGF | 2461.52805 | -1.125170285 | 0.388016983 | -2.8997965 | 0.00373405 | 0.023464708 |
| EDAR | 30.34077111 | 1.265066669 | 0.436614922 | 2.89744259 | 0.003762186 | 0.023625589 |
| LINC02154 | 58.2117053 | -1.883150443 | 0.649977995 | -2.8972526 | 0.003764465 | 0.023625589 |
| MUC2 | 9.299972261 | 3.206562441 | 1.107253988 | 2.89595926 | 0.003780015 | 0.023706543 |
| HTRA4 | 53.0648148 | 1.369844255 | 0.473137975 | 2.89523211 | 0.003788783 | 0.023751088 |
| AC116562.2 | 7.61274476 | 2.200975822 | 0.760571467 | 2.89384485 | 0.003805562 | 0.023840551 |
| AL109947.1 | 5.944488422 | 2.10540608 | 0.72814373 | 2.89147045 | 0.003834436 | 0.023995092 |
| AC010976.2 | 195.9666859 | -1.184782262 | 0.410053387 | -2.8893366 | 0.003860556 | 0.024132069 |
| ARMC2-AS1 | 4.510773681 | 1.678642176 | 0.580940947 | 2.88952291 | 0.003858269 | 0.024132069 |
| RORB | 42.62315055 | 1.27954202 | 0.44288985 | 2.88907506 | 0.003863768 | 0.024141569 |
| AC024909.1 | 409.9407835 | -1.125053747 | 0.389501526 | -2.888445 | 0.003871517 | 0.024174098 |
| AC007406.3 | 39.56184302 | -1.362791865 | 0.471854517 | -2.8881611 | 0.003875013 | 0.024185341 |
| CA12 | 243.9592202 | -1.295821935 | 0.449251558 | -2.8844016 | 0.003921582 | 0.024406566 |
| YBX1P4 | 11.43565856 | -1.769438761 | 0.613700538 | -2.8832283 | 0.00393622 | 0.024484141 |
| AC004556.1 | 62.80999355 | 1.300188538 | 0.451269722 | 2.88117831 | 0.003961914 | 0.024609251 |
| METTL27 | 243.2905332 | 1.066175343 | 0.370223869 | 2.87981255 | 0.003979117 | 0.024708628 |
| AC011487.2 | 13.8965719 | -1.759257149 | 0.610984938 | -2.8793789 | 0.003984593 | 0.024728577 |
| STEAP1 | 125.7983912 | 1.282878062 | 0.445630249 | 2.87879484 | 0.003991979 | 0.024763642 |
| AC091588.3 | 20.26415054 | -1.14518823 | 0.398157758 | -2.8762173 | 0.004024725 | 0.024934808 |
| AL590004.3 | 28.95321556 | 2.298162332 | 0.799076769 | 2.87602196 | 0.004027217 | 0.024944258 |
| RBM43P1 | 3.779604221 | -4.829772909 | 1.679856091 | -2.8751111 | 0.004038853 | 0.025000047 |
| MIR8083 | 17.94455453 | -1.132904703 | 0.394307416 | -2.8731509 | 0.004064 | 0.025128717 |
| LINC01088 | 32.68972746 | -1.144080831 | 0.398960153 | -2.8676569 | 0.004135237 | 0.025522603 |
| C10orf55 | 28.64107938 | -1.397632022 | 0.487519954 | -2.8668201 | 0.004146186 | 0.02558115 |
| GDF9 | 34.16112244 | 1.411748673 | 0.492540108 | 2.86626134 | 0.004153512 | 0.025604768 |
| LINC01447 | 27.83097907 | -1.160770539 | 0.404971014 | -2.8663052 | 0.004152936 | 0.025604768 |
| UVRAG-DT | 12.9195109 | 2.102442456 | 0.733533184 | 2.86618588 | 0.004154502 | 0.025604768 |
| AC124319.3 | 43.92358954 | 1.28136483 | 0.447342873 | 2.86439084 | 0.004178121 | 0.025739213 |
| TNFSF11 | 11.70761078 | 1.928852197 | 0.673515592 | 2.86385678 | 0.004185172 | 0.02577708 |
| OR6N1 | 3.067465811 | -4.530826414 | 1.582221202 | -2.863586 | 0.004188751 | 0.02578799 |
| AC021127.1 | 9.486555603 | 2.098020441 | 0.733746767 | 2.85932495 | 0.004245437 | 0.026086296 |
| AC023055.1 | 144.0995239 | 2.328548891 | 0.814512252 | 2.85882611 | 0.004252118 | 0.026117078 |
| SIK1 | 3241.862958 | 1.122500631 | 0.392803859 | 2.85766193 | 0.004267748 | 0.026178276 |
| AC020907.2 | 41.45309075 | -5.120714843 | 1.793646203 | -2.8549191 | 0.004304779 | 0.026354413 |
| MROH7 | 413.8430403 | 1.094529146 | 0.383576905 | 2.85348031 | 0.004324321 | 0.02645134 |
| AL158168.1 | 20.67880129 | 2.646447784 | 0.928009795 | 2.85174553 | 0.004347989 | 0.026573323 |
| TMED10P2 | 32.0479337 | -1.359809736 | 0.477274563 | -2.8491142 | 0.004384114 | 0.026776892 |
| IRS4 | 12.23838088 | 1.903832896 | 0.668542216 | 2.84773773 | 0.004403119 | 0.026883443 |
| TRIM31 | 8.37926005 | 2.126058119 | 0.747046811 | 2.84595033 | 0.00442791 | 0.027003908 |
| AC131025.1 | 3.776889814 | -3.827012118 | 1.344867514 | -2.8456425 | 0.004432193 | 0.027024248 |
| LINC02575 | 27.49980049 | -1.402447334 | 0.493208857 | -2.8435161 | 0.004461875 | 0.027181988 |
| PAPPA2 | 81.69882985 | -1.412137721 | 0.496731058 | -2.8428617 | 0.004471046 | 0.027226227 |
| SPOCK3 | 17.18822445 | 1.796492513 | 0.632424735 | 2.84064239 | 0.004502277 | 0.02738717 |
| AL135818.1 | 81.67850488 | -1.067120102 | 0.3758624 | -2.8391244 | 0.004523752 | 0.027488492 |
| LINC00958 | 35.91085574 | 1.561448979 | 0.550038481 | 2.83879953 | 0.00452836 | 0.027510629 |
| EPHA10 | 21.78155003 | 1.788845112 | 0.630503956 | 2.83716715 | 0.004551578 | 0.027603392 |
| AC005379.1 | 3.36505214 | -4.668843257 | 1.64581813 | -2.8367917 | 0.004556933 | 0.027619505 |
| AC005821.2 | 19.00630367 | 2.498912089 | 0.881450125 | 2.83500112 | 0.004582553 | 0.02773942 |
| TREML2 | 108.1109188 | -1.195266033 | 0.421952218 | -2.8327047 | 0.0046156 | 0.027898022 |
| AL109615.3 | 73.77284173 | 1.155335121 | 0.408012489 | 2.83161705 | 0.004631328 | 0.027987154 |
| LINC01014 | 19.58046499 | 2.702425904 | 0.954647276 | 2.83081089 | 0.004643016 | 0.02803403 |
| ALPL | 4802.260017 | -1.030161318 | 0.364403839 | -2.8269771 | 0.004698968 | 0.028299974 |
| DIRAS2 | 17.08881693 | 3.19139071 | 1.129207354 | 2.82622204 | 0.00471006 | 0.028354801 |
| AC135012.3 | 65.03549839 | -1.749245264 | 0.619259104 | -2.8247389 | 0.004731916 | 0.028456345 |
| AC012313.3 | 416.7001863 | 1.012180856 | 0.358427229 | 2.82395079 | 0.004743567 | 0.028502371 |
| ROR1-AS1 | 14.84665703 | 1.723724998 | 0.610941985 | 2.82142174 | 0.00478113 | 0.02866911 |
| AC017002.4 | 4.267218385 | 4.71252447 | 1.670385055 | 2.82122045 | 0.004784131 | 0.028679652 |
| IGF2BP2-AS1 | 5.462443723 | 2.225767466 | 0.789058977 | 2.82078721 | 0.004790597 | 0.028712376 |
| AL079303.1 | 7.590563023 | 2.873831827 | 1.018877017 | 2.82058755 | 0.004793579 | 0.028724214 |
| AL451069.1 | 11.22848075 | -2.153109149 | 0.763650124 | -2.8194969 | 0.0048099 | 0.028797811 |
| AC106818.2 | 7.187949857 | 2.20928521 | 0.783840329 | 2.81853986 | 0.004824262 | 0.028850625 |
| HTR2C | 38.6050976 | 3.881928779 | 1.37759092 | 2.81791113 | 0.004833719 | 0.028891903 |
| NSG2 | 43.58444107 | -1.05926486 | 0.375967937 | -2.817434 | 0.004840906 | 0.028928799 |
| MTUS2 | 137.3135964 | 1.126042455 | 0.400362789 | 2.81255523 | 0.004914958 | 0.029302856 |
| AC104051.2 | 3.23009976 | -4.604189506 | 1.637162037 | -2.8122992 | 0.004918872 | 0.029314876 |
| SIK1B | 5713.716908 | 1.153238281 | 0.410269109 | 2.8109313 | 0.004939833 | 0.029415212 |
| TDO2 | 31.37421052 | 1.12958013 | 0.402354586 | 2.80742452 | 0.004993938 | 0.029656902 |
| LINC02405 | 8.606567866 | -3.407403851 | 1.21390269 | -2.8069827 | 0.005000793 | 0.029680349 |
| AC135983.2 | 10.12354945 | -1.088044142 | 0.387821164 | -2.8055306 | 0.005023381 | 0.029767266 |
| AC021491.2 | 13.26950708 | 1.696223862 | 0.604608609 | 2.80549076 | 0.005024002 | 0.029767266 |
| AC104758.1 | 18.32744832 | -1.197447627 | 0.427043354 | -2.8040423 | 0.005046628 | 0.029870302 |
| CD300H | 21.51514585 | -1.570461111 | 0.560200541 | -2.8033909 | 0.005056833 | 0.029918287 |
| AC008763.3 | 166.8513097 | -2.544989787 | 0.908058345 | -2.8026721 | 0.005068117 | 0.029941569 |
| AC112229.1 | 9.270817576 | 1.913922913 | 0.683042235 | 2.80205647 | 0.005077798 | 0.029986345 |
| AP002518.1 | 3.119732137 | 4.653647046 | 1.661643676 | 2.80062875 | 0.005100316 | 0.030100622 |
| MKRN2OS | 30.38902003 | 1.104509523 | 0.394583634 | 2.79917723 | 0.005123301 | 0.030198783 |
| LINC02099 | 6.966307806 | 3.279800643 | 1.172991781 | 2.79609857 | 0.005172362 | 0.030462789 |
| CYP4F30P | 8.525072944 | 2.433621873 | 0.870395728 | 2.79599473 | 0.005174024 | 0.030466287 |
| LINC01506 | 7.410108274 | -4.253626603 | 1.521730788 | -2.7952557 | 0.005185869 | 0.03052648 |
| AP000553.2 | 10.8362828 | 1.799341344 | 0.643720062 | 2.79522334 | 0.005186387 | 0.03052648 |
| SPINK1 | 40.55878346 | 3.225165754 | 1.15482427 | 2.79277622 | 0.005225783 | 0.030707666 |
| AC091825.2 | 3.613885873 | -3.224653996 | 1.155495654 | -2.7907106 | 0.005259247 | 0.030872505 |
| ATP6V1B1 | 262.731757 | 1.395495258 | 0.500318466 | 2.78921398 | 0.005283614 | 0.030990033 |
| CCDC178 | 47.40332365 | 1.857994542 | 0.666268126 | 2.7886589 | 0.005292678 | 0.03103681 |
| IL1R2 | 143.6867902 | -1.602526961 | 0.575024503 | -2.7868846 | 0.005321742 | 0.031188852 |
| IGHV3-66 | 19.91109483 | 2.064070941 | 0.741119518 | 2.78507163 | 0.00535159 | 0.031343614 |
| H3C10 | 30.34673228 | -1.203206795 | 0.432084533 | -2.784656 | 0.005358454 | 0.03137093 |
| LINC01724 | 6.146408907 | -5.535293126 | 1.98941576 | -2.7823712 | 0.005396328 | 0.031547338 |
| CYP2G1P | 49.27998017 | 1.317859574 | 0.473843509 | 2.78121268 | 0.005415625 | 0.031647175 |
| AC092306.1 | 7.535176515 | 1.546056628 | 0.555988908 | 2.78073287 | 0.005423635 | 0.031664687 |
| AP000845.1 | 17.27816814 | 1.450931731 | 0.521997378 | 2.77957666 | 0.00544298 | 0.031754614 |
| AC023300.2 | 10.53622231 | 2.375056507 | 0.85509298 | 2.77754181 | 0.005477179 | 0.03190882 |
| AC239799.1 | 26.32540824 | 1.235878983 | 0.44511464 | 2.77654085 | 0.005494073 | 0.03196683 |
| LRP1B | 11.00786844 | 1.230301132 | 0.443325178 | 2.77516639 | 0.005517347 | 0.032070776 |
| LHFPL3 | 136.1797201 | -1.19680242 | 0.431692907 | -2.7723467 | 0.005565372 | 0.032310423 |
| AC099535.2 | 8.693947126 | 3.091364277 | 1.11522588 | 2.77196246 | 0.005571946 | 0.032342006 |
| AC020911.2 | 11.46670399 | 2.117369617 | 0.764303306 | 2.77032639 | 0.005600015 | 0.032498314 |
| TMEM121 | 199.7839407 | 1.156427684 | 0.417654233 | 2.76886379 | 0.005625215 | 0.032611382 |
| MEP1A | 7.54414694 | 3.757036613 | 1.357256643 | 2.76811068 | 0.00563823 | 0.032680196 |
| ENPP7 | 8.248137553 | -3.082207646 | 1.114196461 | -2.7663054 | 0.005669542 | 0.032841661 |
| ZBTB12BP | 12.66744798 | -2.050954975 | 0.741478636 | -2.7660338 | 0.005674265 | 0.032855677 |
| MB | 28.79342447 | 1.514749292 | 0.547700723 | 2.76565144 | 0.005680922 | 0.032874202 |
| LINC00842 | 48.27431504 | -1.0904255 | 0.394772231 | -2.7621636 | 0.005741969 | 0.033180346 |
| PCARE | 84.25257562 | -1.44094315 | 0.522154791 | -2.7596092 | 0.005787055 | 0.033413798 |
| GBP7 | 21.20117881 | -2.017747959 | 0.731580458 | -2.7580671 | 0.005814427 | 0.033551467 |
| CLEC4D | 32.65406579 | -1.960669366 | 0.711215436 | -2.7567869 | 0.005837239 | 0.033655864 |
| AC020663.2 | 10.4824618 | 3.079603172 | 1.117146046 | 2.75667016 | 0.005839322 | 0.033661072 |
| CX3CR1 | 376.6084658 | -1.16537017 | 0.422917489 | -2.7555497 | 0.00585936 | 0.033756114 |
| CDH12 | 22.16245267 | 1.206931429 | 0.438043078 | 2.75528022 | 0.005864189 | 0.033776214 |
| MYBPHL | 65.56240617 | -1.046259982 | 0.379815196 | -2.7546554 | 0.005875398 | 0.033807541 |
| AC027277.1 | 5.289270384 | -2.219573744 | 0.806123024 | -2.7533933 | 0.005898099 | 0.033904691 |
| AC104984.5 | 24.92698206 | -1.83419103 | 0.666504803 | -2.7519547 | 0.005924071 | 0.034025833 |
| AP000322.2 | 10.06796359 | 1.516487235 | 0.551108674 | 2.75170272 | 0.005928631 | 0.034034396 |
| MOGAT1 | 16.77058506 | -2.016896606 | 0.73316293 | -2.7509528 | 0.005942221 | 0.034075183 |
| DNAH17-AS1 | 28.03283326 | -1.130490718 | 0.410926365 | -2.7510786 | 0.005939939 | 0.034075183 |
| MKRN7P | 28.4160798 | 1.066174893 | 0.387829625 | 2.74908058 | 0.005976269 | 0.034242894 |
| PCDHGB4 | 167.8121657 | -1.269314363 | 0.462160418 | -2.74648 | 0.006023855 | 0.034480919 |
| AL160270.1 | 11.81133069 | 1.909595773 | 0.695539644 | 2.74548804 | 0.006042097 | 0.034578397 |
| TMPRSS7 | 12.58819064 | 2.913352817 | 1.062025484 | 2.74320425 | 0.006084283 | 0.034794752 |
| AL355601.1 | 15.54252009 | 1.920252876 | 0.700213101 | 2.74238353 | 0.006099508 | 0.034857996 |
| MYL7 | 2.981145746 | -4.492910553 | 1.639972588 | -2.7396254 | 0.006150925 | 0.035046132 |
| LINC00840 | 27.1381645 | -2.170941779 | 0.792684213 | -2.7387221 | 0.006167848 | 0.035093863 |
| KRT6A | 16.16940896 | 4.496065952 | 1.641925535 | 2.73828859 | 0.006175985 | 0.035133154 |
| AC011511.5 | 298.9256797 | -1.412526714 | 0.515934525 | -2.7378023 | 0.006185125 | 0.035171117 |
| AC025442.2 | 9.34455132 | -2.232926391 | 0.815661104 | -2.7375663 | 0.006189563 | 0.035189339 |
| AL121694.1 | 7.972365207 | 3.611670928 | 1.319372534 | 2.73741558 | 0.006192401 | 0.035191444 |
| AC020978.4 | 21.35334836 | 1.091112055 | 0.39881719 | 2.73587018 | 0.006221557 | 0.035307891 |
| AC017104.5 | 9.225368084 | 1.199670508 | 0.438731683 | 2.73440591 | 0.006249295 | 0.035415985 |
| AC104389.6 | 63.29130068 | -2.669194801 | 0.976227273 | -2.7341941 | 0.006253318 | 0.035424704 |
| TMPOP2 | 6.282588397 | 2.406879309 | 0.880284579 | 2.73420592 | 0.006253092 | 0.035424704 |
| AC012085.2 | 10.74397297 | -2.021347045 | 0.739607731 | -2.7329988 | 0.006276056 | 0.035539397 |
| AC130456.1 | 96.63949297 | 1.203910423 | 0.440592573 | 2.73248007 | 0.006285948 | 0.035581282 |
| AL445645.1 | 24.39842631 | 1.011081395 | 0.37015521 | 2.73150659 | 0.006304548 | 0.035651194 |
| STAU2-AS1 | 13.17113991 | 1.469843922 | 0.538239748 | 2.73083496 | 0.00631741 | 0.035695618 |
| METTL14-DT | 17.07275358 | 1.17933039 | 0.431937909 | 2.73032389 | 0.006327213 | 0.035743928 |
| EFNA2 | 9.119265598 | 2.912538374 | 1.066880223 | 2.72995816 | 0.006334236 | 0.035762356 |
| GABRB3 | 108.21936 | 1.631564932 | 0.597804611 | 2.72926121 | 0.00634764 | 0.035818192 |
| AL022069.2 | 19.97299775 | 1.211082131 | 0.443977038 | 2.72780353 | 0.006375757 | 0.035939865 |
| CEP295NL | 24.67979636 | -1.400245826 | 0.513314601 | -2.7278512 | 0.006374837 | 0.035939865 |
| BX322234.1 | 82.64598666 | 1.229413193 | 0.450790547 | 2.72723818 | 0.006386692 | 0.03598728 |
| SLC6A12 | 85.42351241 | -1.278459719 | 0.46905336 | -2.7256168 | 0.006418146 | 0.036121623 |
| AL022345.4 | 28.55863663 | 1.092618695 | 0.400945594 | 2.72510463 | 0.006428111 | 0.036163514 |
| MGAT4D | 9.838429725 | 2.646494029 | 0.971265414 | 2.72478973 | 0.006434245 | 0.036183747 |
| BX322557.2 | 6.336951671 | -2.691040247 | 0.989019378 | -2.7209176 | 0.006510098 | 0.036559862 |
| CKS1BP1 | 5.831758533 | 2.128019764 | 0.782276493 | 2.72029108 | 0.006522447 | 0.036607589 |
| AC095050.1 | 13.67487421 | -3.122540937 | 1.148410191 | -2.7190119 | 0.006547726 | 0.036722955 |
| AC008132.1 | 33.07317383 | 1.302180982 | 0.478920786 | 2.71899032 | 0.006548152 | 0.036722955 |
| LRRC2-AS1 | 7.537317397 | 1.739970239 | 0.640303478 | 2.71741494 | 0.006579406 | 0.036883686 |
| FGFBP2 | 415.239786 | -1.283958172 | 0.472714626 | -2.716138 | 0.006604838 | 0.036975423 |
| APOE | 21354.23946 | 1.145222257 | 0.421746326 | 2.71542913 | 0.006618994 | 0.03703287 |
| AP001271.2 | 8.30481261 | 1.646057702 | 0.606214459 | 2.71530591 | 0.006621458 | 0.037039388 |
| AMZ1 | 137.820943 | -1.456845115 | 0.536604875 | -2.7149308 | 0.006628962 | 0.037071215 |
| AC002472.2 | 3.046045956 | 4.33343471 | 1.598164531 | 2.71150725 | 0.006697808 | 0.037393143 |
| RNVU1-3 | 3.474700875 | 1.648744909 | 0.608473122 | 2.70964296 | 0.006735567 | 0.03755984 |
| LINC00862 | 8.222822229 | 2.081423271 | 0.768256633 | 2.70928122 | 0.006742916 | 0.037578779 |
| FAM41C | 41.70338505 | 1.491222416 | 0.550653822 | 2.70809419 | 0.006767081 | 0.037706088 |
| TTC39C-AS1 | 8.246723813 | 2.209625784 | 0.816345538 | 2.70672856 | 0.006794979 | 0.037817207 |
| AC007861.1 | 14.95839321 | -2.267095219 | 0.838513373 | -2.7037079 | 0.006857054 | 0.038066125 |
| AC073862.2 | 34.04269398 | -1.288825092 | 0.476941819 | -2.702269 | 0.006886802 | 0.038194099 |
| AC140479.2 | 19.2097532 | 1.345199914 | 0.497894456 | 2.70177725 | 0.006896995 | 0.038220904 |
| SNX29P2 | 144.091631 | 1.140286855 | 0.422227091 | 2.70064825 | 0.006920449 | 0.038281996 |
| AP001021.1 | 25.82347622 | 1.808074459 | 0.669755125 | 2.69960526 | 0.006942179 | 0.038359507 |
| NXF3 | 646.354016 | -1.856030917 | 0.687702485 | -2.6988864 | 0.006957191 | 0.038427568 |
| AL645728.2 | 21.89056766 | 2.243854378 | 0.831447369 | 2.69873291 | 0.006960401 | 0.038432831 |
| AC020612.4 | 10.08160244 | 3.011379831 | 1.117124068 | 2.69565388 | 0.007025062 | 0.038727454 |
| AL512603.2 | 5.876270541 | 2.992944685 | 1.110351618 | 2.6954927 | 0.007028462 | 0.038731224 |
| ZBTB38 | 2673.541783 | -1.0529179 | 0.390776764 | -2.694423 | 0.007051062 | 0.038807931 |
| NKG7 | 997.6278463 | -1.139331183 | 0.422994419 | -2.6934899 | 0.007070829 | 0.038883796 |
| RNF183 | 34.1138338 | 1.382390357 | 0.51331679 | 2.69305502 | 0.007080058 | 0.03892531 |
| LINC01082 | 41.41330387 | -1.494154892 | 0.555815515 | -2.6882209 | 0.007183385 | 0.039448117 |
| AJAP1 | 33.54386736 | 1.340478229 | 0.498668067 | 2.68811724 | 0.007185616 | 0.039449607 |
| ORM1 | 195.0063226 | -1.080067059 | 0.401946657 | -2.6870905 | 0.007207741 | 0.039551059 |
| AC020658.7 | 12.36047042 | 2.481705665 | 0.923593721 | 2.68701011 | 0.007209477 | 0.039552975 |
| HP | 1868.609968 | -1.527738249 | 0.568703927 | -2.6863508 | 0.00722372 | 0.039608261 |
| CLEC4M | 178.7195077 | -2.299367407 | 0.856320455 | -2.6851716 | 0.007249257 | 0.039694864 |
| HNRNPA1P41 | 2.949122182 | 3.649113507 | 1.359098963 | 2.68495055 | 0.007254054 | 0.039698269 |
| AC092198.1 | 31.43291065 | 1.984210277 | 0.739001319 | 2.68498882 | 0.007253223 | 0.039698269 |
| NCF1 | 1564.775661 | -1.104527633 | 0.411478563 | -2.6842896 | 0.007268411 | 0.039738718 |
| AC096536.2 | 13.16911389 | 1.299654997 | 0.484297116 | 2.68359021 | 0.007283632 | 0.039806677 |
| DOK5 | 52.06885273 | 1.054999585 | 0.393262872 | 2.68268291 | 0.00730342 | 0.039899531 |
| ZNF488 | 7.150798179 | -2.498911283 | 0.931710772 | -2.6820676 | 0.007316867 | 0.039957687 |
| ADAMTS18 | 13.26009671 | 3.000445057 | 1.120401212 | 2.67800947 | 0.007406112 | 0.040336938 |
| LINC01843 | 26.03710147 | 1.945187552 | 0.727105569 | 2.67524777 | 0.007467404 | 0.040609941 |
| AL121949.1 | 21.54837894 | 2.005906695 | 0.750117245 | 2.67412422 | 0.007492469 | 0.040737266 |
| AC022417.1 | 3.173820702 | -3.566047274 | 1.333923434 | -2.6733523 | 0.007509734 | 0.040823354 |
| LINC02478 | 11.71176322 | -1.962499699 | 0.734301358 | -2.672608 | 0.007526414 | 0.040867295 |
| ST18 | 27.82399412 | 1.241366642 | 0.464539655 | 2.67225118 | 0.007534423 | 0.040887431 |
| IGKV1-16 | 54.05693248 | 1.831880098 | 0.687263986 | 2.66546791 | 0.007688127 | 0.041547514 |
| CYP4F26P | 33.68030056 | 2.377522634 | 0.892364812 | 2.66429447 | 0.007714999 | 0.041669178 |
| CR381670.1 | 16.80848731 | -2.594747535 | 0.973977189 | -2.6640742 | 0.007720052 | 0.041680684 |
| AC004938.2 | 39.76487445 | 1.083677239 | 0.406869672 | 2.66345052 | 0.007734379 | 0.041750129 |
| AL137781.1 | 41.91028745 | 2.190142766 | 0.822367758 | 2.66321575 | 0.007739777 | 0.041771365 |
| AL160408.6 | 26.12174022 | 1.584097921 | 0.595659042 | 2.6594038 | 0.007827908 | 0.042175184 |
| C1GALT1P1 | 29.73720447 | 1.027223231 | 0.386453626 | 2.65807632 | 0.007858809 | 0.042317693 |
| HCG14 | 3.05418462 | 2.542902616 | 0.95722062 | 2.6565481 | 0.007894519 | 0.042461883 |
| AADACL2-AS1 | 3.129108731 | 4.018316252 | 1.51273962 | 2.65631719 | 0.007899927 | 0.04248296 |
| AP000640.1 | 14.19456058 | -1.32371466 | 0.498486634 | -2.6554667 | 0.007919875 | 0.04257418 |
| AC018523.2 | 5.203612897 | -5.294690557 | 1.994089374 | -2.6551922 | 0.007926323 | 0.042592788 |
| CD207 | 125.8131646 | 1.092373965 | 0.411504352 | 2.65458667 | 0.007940564 | 0.04265324 |
| AC007319.1 | 30.13382757 | -1.003255826 | 0.378058539 | -2.653705 | 0.00796134 | 0.04272461 |
| CORT | 38.83295764 | 1.049592187 | 0.395561438 | 2.65342394 | 0.007967973 | 0.04275216 |
| LILRB3 | 169.9437618 | -1.086173217 | 0.409973442 | -2.6493746 | 0.00806409 | 0.043129951 |
| AC233263.6 | 7.896239929 | -1.868370663 | 0.705262688 | -2.649184 | 0.008068638 | 0.043138098 |
| SLC28A2 | 72.39842996 | 2.367417219 | 0.893684048 | 2.64905391 | 0.008071746 | 0.043146626 |
| ATP11A-AS1 | 10.79345421 | -1.961714919 | 0.740587962 | -2.6488615 | 0.008076343 | 0.043161877 |
| PTPN20CP | 13.26919741 | 2.30353151 | 0.870053847 | 2.6475735 | 0.008107173 | 0.043303541 |
| AC093772.2 | 11.15339043 | 1.409362735 | 0.532358457 | 2.64739428 | 0.008111472 | 0.043318388 |
| CHST13 | 33.3656711 | 1.351323787 | 0.510556148 | 2.64676822 | 0.008126503 | 0.043382416 |
| EDA2R | 45.48916757 | 1.242417292 | 0.469601413 | 2.64568474 | 0.008152576 | 0.043489044 |
| HPCAL4 | 106.4591593 | -1.310424239 | 0.495488208 | -2.6447133 | 0.008176016 | 0.043581483 |
| AL161756.3 | 45.02475664 | 1.125751328 | 0.425697538 | 2.64448635 | 0.008181501 | 0.043602568 |
| THAP12P3 | 2.938257008 | 5.389794295 | 2.040372948 | 2.6415731 | 0.0082522 | 0.043913711 |
| CHCHD2P7 | 5.296728767 | 2.548628908 | 0.964908822 | 2.64131579 | 0.00825847 | 0.043930688 |
| AC008875.2 | 27.48872453 | 1.492826261 | 0.565383447 | 2.64037843 | 0.00828135 | 0.044035969 |
| AC026369.2 | 40.72124427 | -1.00848406 | 0.382000037 | -2.6400104 | 0.008290349 | 0.044059181 |
| CCDC162P | 164.2950097 | 1.145569223 | 0.434271894 | 2.63790782 | 0.008341925 | 0.044250835 |
| PSAT1 | 57.5987943 | 1.136882065 | 0.43097819 | 2.6379109 | 0.00834185 | 0.044250835 |
| AC092647.5 | 34.53350388 | 1.293625767 | 0.490720344 | 2.63617717 | 0.008384594 | 0.044435332 |
| AL390783.1 | 6.003563443 | -3.457100652 | 1.311881927 | -2.6352224 | 0.008408217 | 0.044544497 |
| TNNT1 | 431.9542143 | -1.544428318 | 0.586539573 | -2.6331187 | 0.008460477 | 0.044796398 |
| AC110597.1 | 14.07351362 | -1.132367667 | 0.430280651 | -2.6316955 | 0.008495996 | 0.044926085 |
| HMGB1P16 | 7.693422171 | 2.598412296 | 0.98802748 | 2.62989881 | 0.008541029 | 0.045139107 |
| AL158198.2 | 3.358210518 | 4.031715068 | 1.533206437 | 2.62959701 | 0.008548614 | 0.045162458 |
| RDH12 | 10.69807805 | 1.37863127 | 0.524530468 | 2.62831495 | 0.008580903 | 0.045307866 |
| AL365475.1 | 12.79614997 | -2.465558695 | 0.938098498 | -2.6282514 | 0.008582506 | 0.045307944 |
| AC011899.1 | 25.61459658 | 1.855143586 | 0.706284738 | 2.62662279 | 0.008623687 | 0.045483253 |
| ENHO | 95.1928076 | -1.240748642 | 0.472646685 | -2.6251081 | 0.008662146 | 0.045627035 |
| LINC02397 | 41.95018917 | 1.373699726 | 0.523311467 | 2.62501362 | 0.00866455 | 0.045627859 |
| AL772337.1 | 14.62219952 | -4.689893902 | 1.786642435 | -2.6249762 | 0.008665502 | 0.045627859 |
| AP003783.1 | 8.273026134 | 2.209230736 | 0.842056646 | 2.62361297 | 0.008700259 | 0.045768624 |
| AC092078.2 | 6.421441719 | -2.699522511 | 1.028923384 | -2.623638 | 0.008699619 | 0.045768624 |
| IGHG4 | 311.1742347 | 1.696102357 | 0.646897114 | 2.62190435 | 0.008743997 | 0.045939403 |
| COX6B2 | 23.55779926 | 1.392134947 | 0.531224776 | 2.62061374 | 0.008777165 | 0.046062753 |
| AL390195.2 | 9.087732883 | 1.543387745 | 0.589436858 | 2.61841065 | 0.008834042 | 0.046293109 |
| DIRAS1 | 42.89642464 | 1.5262869 | 0.58294189 | 2.6182488 | 0.008838234 | 0.046298062 |
| PAX1 | 4.958489213 | 3.937325516 | 1.503793985 | 2.61826125 | 0.008837911 | 0.046298062 |
| GRIA2 | 11.80514419 | 1.883210031 | 0.719424747 | 2.6176609 | 0.008853474 | 0.046369378 |
| AC011416.3 | 28.33133944 | 1.066133273 | 0.407606696 | 2.61559313 | 0.008907263 | 0.046608302 |
| SLC2A14 | 138.2946218 | -1.964317505 | 0.751550853 | -2.6136854 | 0.008957147 | 0.046774935 |
| AL135841.1 | 20.18953398 | -1.167026104 | 0.4467574 | -2.6122144 | 0.008995782 | 0.046942317 |
| LINC02570 | 6.136369841 | -1.980378754 | 0.758427041 | -2.6111658 | 0.009023413 | 0.047069279 |
| AC018450.1 | 20.44079851 | -1.714086614 | 0.656555482 | -2.6107262 | 0.009035019 | 0.047121202 |
| AC019117.4 | 15.93771439 | -6.909891109 | 2.647196386 | -2.6102677 | 0.009047141 | 0.047158551 |
| PCDHB1 | 13.00438538 | 1.953587325 | 0.748632926 | 2.60953968 | 0.009066413 | 0.047241743 |
| AC018816.1 | 18.79705957 | -1.17415087 | 0.450355579 | -2.607164 | 0.009129561 | 0.04751003 |
| LINC02417 | 6.938714937 | 2.979440472 | 1.143170803 | 2.60629511 | 0.009152757 | 0.047596003 |
| ADH7 | 36.97278956 | 3.491102792 | 1.340007367 | 2.60528627 | 0.009179753 | 0.047708614 |
| SERTAD1 | 860.2986332 | -1.079126342 | 0.414272463 | -2.604871 | 0.009190885 | 0.047750747 |
| AC016866.3 | 13.18957838 | -1.404452968 | 0.539768025 | -2.6019566 | 0.009269358 | 0.04808838 |
| FAM3D | 171.6653551 | 1.045257229 | 0.401961798 | 2.60038947 | 0.009311801 | 0.048255905 |
| IQSEC3 | 480.7679893 | -1.106356732 | 0.425586883 | -2.5996025 | 0.00933318 | 0.048353678 |
| AL359979.2 | 5.745372292 | 4.191032335 | 1.612240679 | 2.59950787 | 0.009335754 | 0.048353678 |
| GUCA2A | 26.17254673 | -1.619145386 | 0.622975433 | -2.5990517 | 0.009348168 | 0.048391615 |
| LPO | 8.510317046 | 1.82518385 | 0.702715944 | 2.59732807 | 0.009395214 | 0.048608687 |
| AL845472.2 | 13.85247059 | 1.581681623 | 0.609034714 | 2.59703033 | 0.009403362 | 0.04864202 |
| PRSS57 | 8.72498697 | -2.293837248 | 0.883358345 | -2.5967234 | 0.009411769 | 0.048667856 |
| ZNF98 | 10.81974285 | 2.012009349 | 0.774919535 | 2.59641067 | 0.00942034 | 0.048703344 |
| AC007666.1 | 30.39506518 | -1.213876117 | 0.467636295 | -2.5957697 | 0.009437931 | 0.048776607 |
| AP000553.6 | 3.038550916 | 1.598749976 | 0.616775259 | 2.59211107 | 0.009538897 | 0.049218154 |
| AC009060.2 | 5.814496728 | 1.513179912 | 0.583825773 | 2.59183473 | 0.009546562 | 0.049222089 |
| AC005479.1 | 25.52667107 | -1.225165635 | 0.472764378 | -2.5914931 | 0.009556045 | 0.049236029 |
| ASS1P2 | 11.32133662 | 1.283917285 | 0.495478844 | 2.5912656 | 0.009562366 | 0.049250156 |
| SOX30 | 19.81594472 | 1.676301903 | 0.646957766 | 2.59105307 | 0.009568274 | 0.049271686 |
| AC090617.10 | 54.02551867 | 1.348376315 | 0.520560881 | 2.5902375 | 0.009590974 | 0.049351406 |
| AC092718.5 | 9.609520272 | 2.170515849 | 0.838147974 | 2.5896571 | 0.009607158 | 0.049418387 |
| AC027104.1 | 15.00122919 | 2.041552163 | 0.788620923 | 2.58876236 | 0.009632154 | 0.049520175 |
| AC140912.1 | 21.88805253 | -1.564412393 | 0.604398771 | -2.5883779 | 0.009642914 | 0.049566559 |
| GMNC | 5.663541085 | 3.477737882 | 1.34380177 | 2.5879843 | 0.009653938 | 0.049605346 |
