# Supplementary material for: Integrative analysis of bulk and single-cell RNA sequencing reveals the gene expression profile and the critical signaling pathways of type II CPAM
Source: Cell Biosci. 2024 Jul 18;14:94. doi: 10.1186/s13578-024-01276-8 (PMC11264590; doi:10.1186/s13578-024-01276-8)
Supplement: Supplementary file 16 — Supplementary Material 16: Supplemental Table 10 Marker genes of epithelial subtypes. [file 13578_2024_1276_MOESM16_ESM.docx]

| **Supplemental Table 4a GO enrichment analysis (biological process, BP) of up-regulated genes** | | | | | | |
| --- | --- | --- | --- | --- | --- | --- |
| **ID** | **Description** | **Gene**  **Ratio** | **Bg Ratio** | ***P*-value** | **p.adjust** | **Gene ID** |
| GO:0044782 | cilium organization | 127/951 | 384/18800 | 1.67E-69 | 7.61E-66 | CCNO/NME5/UBXN10/CFAP61/IQCG/TEKT2/SPAG16/DNAL1/FOXJ1/CFAP126/CCDC65/CFAP53/DRC1/DAW1/RSPH1/MNS1/DNAI2/RSPH9/TEKT1/RP1/TMEM231/DNAI1/CFAP43/CFAP52/KIF24/DRC7/DNAAF3/TEKT3/CCDC113/KIF19/CFAP221/RSPH4A/CFAP58/CFAP73/PIFO/TTC29/TTC26/SPEF1/IFT46/TEKT4/CFAP65/SPEF2/ZMYND10/SPAG1/DNAH5/CCDC96/DNAJB13/CFAP47/DNAAF4/LCA5L/CFAP54/CFAP157/DNAH7/CCDC40/DZIP1L/TMEM67/CEP83/GAS8/TMEM107/ENO4/BBS5/MAK/DYNC2H1/IFT88/CFAP74/SPAG6/RABL2B/TTC21A/CLUAP1/LRGUK/BBOF1/DNAAF1/CFAP69/HYDIN/SPAG17/CFAP298/RPGRIP1L/RFX3/CFAP100/MEIG1/MAPK15/CFAP70/B9D1/CFAP161/TTC30B/DNAH2/IFT172/CCDC39/ARMC2/CFAP46/ROPN1B/IFT81/TRAF3IP1/TCTN1/IQUB/IFT27/TTC30A/CEP19/CC2D2A/CEP41/CCDC103/DCDC2/TCTN2/CATIP/CEP126/B9D2/UNC119B/FAM161A/CFAP44/FUZ/MCIDAS/INTU/TMEM17/RFX2/WDR35/BBS12/WDR90/CCDC13/LCA5/DNAAF2/KIF3A/DNAH1/KIF27/CFAP206/SAXO1/ROPN1/GMNC |
| GO:0060271 | cilium assembly | 111/951 | 355/18800 | 9.19E-58 | 2.10E-54 | CCNO/NME5/UBXN10/IQCG/TEKT2/SPAG16/DNAL1/FOXJ1/CCDC65/CFAP53/DRC1/DAW1/RSPH1/MNS1/DNAI2/RSPH9/TEKT1/RP1/TMEM231/DNAI1/CFAP43/CFAP52/KIF24/DRC7/DNAAF3/TEKT3/CCDC113/CFAP221/RSPH4A/CFAP58/CFAP73/TTC26/SPEF1/IFT46/TEKT4/CFAP65/SPEF2/ZMYND10/SPAG1/DNAH5/CCDC96/DNAJB13/CFAP47/DNAAF4/CFAP54/CFAP157/DNAH7/CCDC40/DZIP1L/TMEM67/CEP83/GAS8/TMEM107/BBS5/MAK/DYNC2H1/IFT88/CFAP74/SPAG6/RABL2B/CLUAP1/LRGUK/BBOF1/DNAAF1/CFAP69/HYDIN/SPAG17/CFAP298/RPGRIP1L/RFX3/CFAP100/MEIG1/MAPK15/CFAP70/B9D1/CFAP161/DNAH2/IFT172/CCDC39/ARMC2/CFAP46/IFT81/TRAF3IP1/TCTN1/IQUB/CEP19/CC2D2A/CEP41/CCDC103/DCDC2/TCTN2/CEP126/B9D2/UNC119B/FAM161A/CFAP44/FUZ/MCIDAS/INTU/TMEM17/RFX2/WDR35/WDR90/CCDC13/DNAAF2/KIF3A/DNAH1/KIF27/CFAP206/SAXO1/GMNC |
| GO:0007018 | microtubule-based movement | 113/951 | 395/18800 | 2.61E-54 | 3.97E-51 | NME5/TCTE1/CFAP61/IQCG/TEKT2/SPA17/DNAH6/SPAG16/CCDC65/CFAP53/ENKUR/GAS2L2/DRC1/DAW1/ZBBX/MNS1/DNAI2/RSPH9/TEKT1/DNAI1/CFAP43/CFAP52/KIF24/DRC7/TEKT3/CATSPERD/CFAP45/KIF19/CFAP221/RSPH4A/CFAP58/CFAP73/TTC29/TTC26/SPEF1/IFT46/TEKT4/CFAP65/SPEF2/ZMYND10/DPCD/TTLL6/TTLL9/ROPN1L/DNAL4/DNAH5/DNAH11/CFAP47/NEK10/KIF21A/DNAAF4/DYNLRB2/LCA5L/CFAP54/KIF6/CFAP157/DNAH7/CCDC40/FMN2/GAS8/DNAH9/ENO4/MAK/DYNC2H1/IFT88/SPAG6/RABL2B/TTC21A/DNAAF1/DNAH12/CFAP69/DNAH10/HYDIN/SPAG17/CFAP298/RFX3/MAP1A/CFAP100/MEIG1/CFAP70/TTC30B/DNAH2/IFT172/CCDC39/ARMC2/CFAP46/ROPN1B/IFT81/TRAF3IP1/IFT27/TTC30A/CCDC103/BICDL2/SORD/CFAP44/AGBL4/DNAH3/CNIH2/KIF9/CATSPERE/WDR35/BBS12/AP3B2/RAB27B/LCA5/DNAAF2/KIF3A/DNAH1/KIF27/CFAP206/CABYR/EPPIN/ROPN1 |
| GO:0003341 | cilium movement | 78/951 | 187/18800 | 1.90E-51 | 2.17E-48 | NME5/TCTE1/CFAP61/IQCG/TEKT2/SPA17/SPAG16/CCDC65/CFAP53/ENKUR/GAS2L2/DRC1/DAW1/ZBBX/MNS1/DNAI2/RSPH9/TEKT1/DNAI1/CFAP43/CFAP52/DRC7/TEKT3/CATSPERD/CFAP45/CFAP221/RSPH4A/CFAP58/CFAP73/TTC29/SPEF1/TEKT4/CFAP65/SPEF2/ZMYND10/DPCD/TTLL6/TTLL9/ROPN1L/DNAH5/DNAH11/CFAP47/NEK10/DNAAF4/CFAP54/CFAP157/DNAH7/CCDC40/GAS8/DNAH9/ENO4/SPAG6/TTC21A/DNAAF1/CFAP69/HYDIN/SPAG17/CFAP298/RFX3/CFAP100/MEIG1/CFAP70/CCDC39/ARMC2/CFAP46/ROPN1B/CCDC103/SORD/CFAP44/KIF9/CATSPERE/DNAAF2/DNAH1/KIF27/CFAP206/CABYR/EPPIN/ROPN1 |
| GO:0035082 | axoneme assembly | 53/951 | 87/18800 | 1.64E-46 | 1.50E-43 | IQCG/TEKT2/SPAG16/DNAL1/FOXJ1/CCDC65/DRC1/DAW1/RSPH1/MNS1/DNAI2/RSPH9/RP1/DNAI1/CFAP43/DRC7/DNAAF3/RSPH4A/CFAP58/CFAP73/TTC26/SPEF1/CFAP65/SPEF2/ZMYND10/SPAG1/DNAH5/DNAJB13/CFAP47/DNAAF4/CFAP157/DNAH7/CCDC40/GAS8/CFAP74/SPAG6/LRGUK/DNAAF1/CFAP69/HYDIN/SPAG17/CFAP100/MEIG1/DNAH2/CCDC39/ARMC2/CFAP46/CC2D2A/CCDC103/CFAP44/DNAAF2/DNAH1/CFAP206 |
| GO:0001578 | microtubule bundle formation | 56/951 | 118/18800 | 5.88E-41 | 4.47E-38 | IQCG/TEKT2/SPAG16/DNAL1/FOXJ1/CCDC65/GAS2L2/DRC1/DAW1/RSPH1/MNS1/DNAI2/RSPH9/RP1/DNAI1/CFAP43/DRC7/DNAAF3/RSPH4A/CFAP58/CFAP73/TTC26/SPEF1/CFAP65/SPEF2/ZMYND10/SPAG1/TTLL6/DNAH5/DNAJB13/CFAP47/DNAAF4/CFAP157/DNAH7/CCDC40/GAS8/CFAP74/SPAG6/LRGUK/DNAAF1/CFAP69/HYDIN/SPAG17/CFAP100/MEIG1/DNAH2/CCDC39/TPPP3/ARMC2/CFAP46/CC2D2A/CCDC103/CFAP44/DNAAF2/DNAH1/CFAP206 |
| GO:0001539 | cilium or flagellum-dependent cell motility | 59/951 | 151/18800 | 3.63E-37 | 2.07E-34 | TCTE1/IQCG/TEKT2/DNAH6/SPAG16/CCDC65/ENKUR/GAS2L2/EFHC2/DRC1/MNS1/RSPH9/TEKT1/DNAI1/CFAP43/CFAP52/DRC7/TEKT3/CATSPERD/CFAP45/RSPH4A/CFAP58/TEKT4/CFAP65/SPEF2/DPCD/TTLL6/TTLL9/ROPN1L/DNAH5/DNAH11/CFAP47/CFAP54/EFHC1/CFAP157/DNAH7/CCDC40/GAS8/ENO4/SPAG6/TTC21A/CFAP69/RFX3/MEIG1/DNAH2/CCDC39/ARMC2/CFAP46/ROPN1B/SORD/CFAP44/DNAH3/KIF9/CATSPERE/DNAAF2/DNAH1/CFAP206/EPPIN/ROPN1 |
| GO:0060285 | cilium-dependent cell motility | 59/951 | 151/18800 | 3.63E-37 | 2.07E-34 | TCTE1/IQCG/TEKT2/DNAH6/SPAG16/CCDC65/ENKUR/GAS2L2/EFHC2/DRC1/MNS1/RSPH9/TEKT1/DNAI1/CFAP43/CFAP52/DRC7/TEKT3/CATSPERD/CFAP45/RSPH4A/CFAP58/TEKT4/CFAP65/SPEF2/DPCD/TTLL6/TTLL9/ROPN1L/DNAH5/DNAH11/CFAP47/CFAP54/EFHC1/CFAP157/DNAH7/CCDC40/GAS8/ENO4/SPAG6/TTC21A/CFAP69/RFX3/MEIG1/DNAH2/CCDC39/ARMC2/CFAP46/ROPN1B/SORD/CFAP44/DNAH3/KIF9/CATSPERE/DNAAF2/DNAH1/CFAP206/EPPIN/ROPN1 |
| GO:0003351 | epithelial cilium movement involved in extracellular fluid movement | 30/951 | 41/18800 | 1.57E-30 | 7.99E-28 | NME5/SPA17/SPAG16/CFAP53/DAW1/DNAI1/CFAP43/CFAP45/CFAP221/SPEF2/DPCD/ROPN1L/DNAH5/DNAH11/NEK10/DNAAF4/CFAP54/CCDC40/GAS8/DNAH9/SPAG6/DNAAF1/SPAG17/RFX3/CCDC39/CCDC103/DNAAF2/DNAH1/KIF27/CABYR |
| GO:0006858 | extracellular transport | 30/951 | 44/18800 | 4.95E-29 | 2.26E-26 | NME5/SPA17/SPAG16/CFAP53/DAW1/DNAI1/CFAP43/CFAP45/CFAP221/SPEF2/DPCD/ROPN1L/DNAH5/DNAH11/NEK10/DNAAF4/CFAP54/CCDC40/GAS8/DNAH9/SPAG6/DNAAF1/SPAG17/RFX3/CCDC39/CCDC103/DNAAF2/DNAH1/KIF27/CABYR |
| GO:0060294 | cilium movement involved in cell motility | 49/951 | 138/18800 | 8.85E-29 | 3.67E-26 | TCTE1/IQCG/TEKT2/SPAG16/ENKUR/GAS2L2/MNS1/RSPH9/TEKT1/DNAI1/CFAP43/CFAP52/DRC7/TEKT3/CATSPERD/CFAP45/RSPH4A/CFAP58/TEKT4/CFAP65/SPEF2/DPCD/TTLL6/TTLL9/ROPN1L/DNAH5/DNAH11/CFAP47/CFAP54/CFAP157/CCDC40/GAS8/ENO4/SPAG6/TTC21A/CFAP69/MEIG1/CCDC39/ARMC2/CFAP46/ROPN1B/SORD/CFAP44/KIF9/CATSPERE/DNAH1/CFAP206/EPPIN/ROPN1 |
| GO:0099111 | microtubule-based transport | 54/951 | 198/18800 | 3.60E-25 | 1.37E-22 | NME5/SPA17/SPAG16/CFAP53/DAW1/DNAI1/CFAP43/CFAP45/CFAP221/TTC26/IFT46/SPEF2/DPCD/ROPN1L/DNAH5/DNAH11/NEK10/DNAAF4/LCA5L/CFAP54/CCDC40/GAS8/DNAH9/MAK/DYNC2H1/IFT88/SPAG6/RABL2B/TTC21A/DNAAF1/SPAG17/RFX3/MAP1A/TTC30B/IFT172/CCDC39/IFT81/TRAF3IP1/IFT27/TTC30A/CCDC103/BICDL2/AGBL4/CNIH2/WDR35/BBS12/AP3B2/RAB27B/LCA5/DNAAF2/KIF3A/DNAH1/KIF27/CABYR |
| GO:0044458 | motile cilium assembly | 30/951 | 57/18800 | 3.21E-24 | 1.13E-21 | IQCG/SPAG16/FOXJ1/MNS1/RSPH9/CFAP43/DRC7/DNAAF3/CFAP221/CFAP58/CFAP65/SPEF2/ZMYND10/CFAP47/CFAP157/CCDC40/BBS5/SPAG6/BBOF1/DNAAF1/CFAP69/MEIG1/CCDC39/ARMC2/CC2D2A/CFAP44/MCIDAS/INTU/DNAH1/CFAP206 |
| GO:0030317 | flagellated sperm motility | 41/951 | 122/18800 | 3.18E-23 | 9.69E-21 | TCTE1/IQCG/TEKT2/SPAG16/ENKUR/MNS1/DNAI1/CFAP43/CFAP52/DRC7/TEKT3/CATSPERD/CFAP45/CFAP58/CFAP65/SPEF2/DPCD/TTLL9/ROPN1L/DNAH5/DNAH11/CFAP47/CFAP157/CCDC40/GAS8/ENO4/SPAG6/TTC21A/CFAP69/MEIG1/CCDC39/ARMC2/ROPN1B/SORD/CFAP44/KIF9/CATSPERE/DNAH1/CFAP206/EPPIN/ROPN1 |
| GO:0097722 | sperm motility | 41/951 | 122/18800 | 3.18E-23 | 9.69E-21 | TCTE1/IQCG/TEKT2/SPAG16/ENKUR/MNS1/DNAI1/CFAP43/CFAP52/DRC7/TEKT3/CATSPERD/CFAP45/CFAP58/CFAP65/SPEF2/DPCD/TTLL9/ROPN1L/DNAH5/DNAH11/CFAP47/CFAP157/CCDC40/GAS8/ENO4/SPAG6/TTC21A/CFAP69/MEIG1/CCDC39/ARMC2/ROPN1B/SORD/CFAP44/KIF9/CATSPERE/DNAH1/CFAP206/EPPIN/ROPN1 |
| GO:0070286 | axonemal dynein complex assembly | 22/951 | 38/18800 | 2.50E-19 | 7.14E-17 | TEKT2/DNAL1/CCDC65/DRC1/DAW1/DNAI2/DNAI1/DNAAF3/CFAP73/ZMYND10/SPAG1/DNAH5/DNAAF4/DNAH7/CCDC40/DNAAF1/CFAP100/DNAH2/CCDC39/CCDC103/DNAAF2/DNAH1 |
| GO:0048515 | spermatid differentiation | 41/951 | 183/18800 | 4.32E-16 | 1.16E-13 | NME5/IQCG/SPAG16/DPY19L2P1/RSPH1/PACRG/MNS1/CFAP43/DRC7/CATSPERD/CFAP58/TTC26/CFAP65/SPEF2/ROPN1L/CFAP47/CFAP157/TRIP13/NPHP1/SPAG6/TTC21A/CFAP69/DPY19L2/DPY19L2P2/MEIG1/STRBP/ARMC2/ROPN1B/PCSK4/CFAP44/RFX2/CATSPERE/OCA2/TCP11X1/DNAH1/CFAP206/CABYR/TCP11/ROPN1/SPINK1/SOX30 |
| GO:0007286 | spermatid development | 40/951 | 177/18800 | 7.23E-16 | 1.83E-13 | NME5/IQCG/SPAG16/DPY19L2P1/RSPH1/PACRG/MNS1/CFAP43/DRC7/CATSPERD/CFAP58/TTC26/CFAP65/SPEF2/ROPN1L/CFAP47/CFAP157/TRIP13/SPAG6/TTC21A/CFAP69/DPY19L2/DPY19L2P2/MEIG1/STRBP/ARMC2/ROPN1B/PCSK4/CFAP44/RFX2/CATSPERE/OCA2/TCP11X1/DNAH1/CFAP206/CABYR/TCP11/ROPN1/SPINK1/SOX30 |
| GO:0007288 | sperm axoneme assembly | 17/951 | 28/18800 | 1.03E-15 | 2.47E-13 | IQCG/SPAG16/MNS1/CFAP43/DRC7/CFAP58/CFAP65/SPEF2/CFAP47/CFAP157/SPAG6/CFAP69/MEIG1/ARMC2/CFAP44/DNAH1/CFAP206 |
| GO:0120316 | sperm flagellum assembly | 17/951 | 32/18800 | 2.23E-14 | 5.10E-12 | IQCG/SPAG16/MNS1/CFAP43/DRC7/CFAP58/CFAP65/SPEF2/CFAP47/CFAP157/SPAG6/CFAP69/MEIG1/ARMC2/CFAP44/DNAH1/CFAP206 |
| GO:0036159 | inner dynein arm assembly | 13/951 | 17/18800 | 2.58E-14 | 5.62E-12 | TEKT2/CFAP73/ZMYND10/DNAAF4/DNAH7/CCDC40/DNAAF1/CFAP100/DNAH2/CCDC39/CCDC103/DNAAF2/DNAH1 |
| GO:0007368 | determination of left/right symmetry | 31/951 | 128/18800 | 1.82E-13 | 3.77E-11 | FOXJ1/CFAP53/ENKUR/DRC1/DAW1/DNAI2/DNAI1/CFAP52/CFAP45/DPCD/DNAH5/DNAH11/DNAAF4/CCDC40/GAS8/TMEM107/BBS5/DYNC2H1/DNAAF1/RPGRIP1L/RFX3/IFT172/CCDC39/CC2D2A/CCDC103/FOXN4/OVOL2/DNAAF2/CFC1/IHH/CFC1B |
| GO:0009855 | determination of bilateral symmetry | 32/951 | 138/18800 | 2.70E-13 | 5.37E-11 | FOXJ1/CFAP53/ENKUR/DRC1/DAW1/DNAI2/DNAI1/CFAP52/CFAP45/DPCD/DNAH5/DNAH11/DNAAF4/CCDC40/GAS8/TMEM107/BBS5/DYNC2H1/DNAAF1/RPGRIP1L/RFX3/IFT172/CCDC39/CC2D2A/CCDC103/FOXN4/OVOL2/DNAAF2/CFC1/GREM1/IHH/CFC1B |
| GO:0009799 | specification of symmetry | 32/951 | 139/18800 | 3.35E-13 | 6.37E-11 | FOXJ1/CFAP53/ENKUR/DRC1/DAW1/DNAI2/DNAI1/CFAP52/CFAP45/DPCD/DNAH5/DNAH11/DNAAF4/CCDC40/GAS8/TMEM107/BBS5/DYNC2H1/DNAAF1/RPGRIP1L/RFX3/IFT172/CCDC39/CC2D2A/CCDC103/FOXN4/OVOL2/DNAAF2/CFC1/GREM1/IHH/CFC1B |
| GO:0007281 | germ cell development | 48/951 | 299/18800 | 1.03E-12 | 1.89E-10 | NME5/IQCG/SPAG16/DPY19L2P1/INHBB/RSPH1/PACRG/MNS1/CFAP43/DRC7/BMPR1B/CATSPERD/WNT4/CFAP58/TTC26/CFAP65/SPEF2/ROPN1L/CFAP47/CFAP157/TRIP13/NPM2/FMN2/SPAG6/TTC21A/CFAP69/DPY19L2/DPY19L2P2/MEIG1/STRBP/ARMC2/ROPN1B/PCSK4/LGR5/CFAP44/RFX2/CATSPERE/OCA2/DMRTA1/TCP11X1/DNAH1/CFAP206/CABYR/IHH/TCP11/ROPN1/SPINK1/SOX30 |
| GO:0042073 | intraciliary transport | 17/951 | 39/18800 | 1.44E-12 | 2.53E-10 | TTC26/IFT46/LCA5L/MAK/DYNC2H1/IFT88/RABL2B/TTC21A/TTC30B/IFT172/IFT81/TRAF3IP1/IFT27/TTC30A/WDR35/BBS12/LCA5 |
| GO:0007389 | pattern specification process | 61/951 | 463/18800 | 6.53E-12 | 1.10E-09 | SIX1/HOXB4/TP63/HES2/FOXJ1/CFAP53/EYA1/ENKUR/HOXC4/DRC1/DAW1/MNS1/DNAI2/DNAI1/CFAP52/FOXA1/BMPR1B/CFAP45/PIFO/DPCD/HOXB3/DNAH5/DNAH11/DNAAF4/CCDC40/DZIP1L/GAS8/TMEM107/BBS5/DYNC2H1/HOXB2/DNAAF1/RPGRIP1L/RFX3/IFT172/CCDC39/CDK20/TCTN1/CC2D2A/CCDC103/CXXC4/LMX1B/FUZ/INTU/WNT7B/FOXN4/OVOL2/LRP2/DNAAF2/ERBB4/CFC1/DSCAML1/HEY2/GREM1/GRHL3/NKX2-2/ISL1/HOXB1/IHH/CFC1B/MEOX1 |
| GO:0003352 | regulation of cilium movement | 16/951 | 39/18800 | 2.04E-11 | 3.33E-09 | CCDC65/GAS2L2/DRC1/CFAP43/CFAP45/RSPH4A/TTLL6/DNAH11/CCDC40/DNAAF1/CFAP69/CFAP298/CCDC39/KIF9/CFAP206/EPPIN |
| GO:0022412 | cellular process involved in reproduction in multicellular organism | 53/951 | 406/18800 | 2.29E-10 | 3.60E-08 | NME5/IQCG/SPAG16/DPY19L2P1/INHBB/RSPH1/PACRG/MNS1/CFAP43/DRC7/BMPR1B/CATSPERD/WNT4/CFAP58/TTC26/CFAP65/SPEF2/ROPN1L/CFAP47/CFAP157/TRIP13/NPM2/FMN2/NPHP1/SPAG6/TTC21A/CCNA1/CFAP69/DPY19L2/DPY19L2P2/MEIG1/STRBP/ARMC2/ROPN1B/PCSK4/LGR5/CFAP44/SPIRE2/RFX2/CATSPERE/OCA2/DMRTA1/TCP11X1/DNAH1/CFAP206/CABYR/IHH/PTGDR2/TCP11/ROPN1/GDF9/SPINK1/SOX30 |
| GO:0061512 | protein localization to cilium | 20/951 | 75/18800 | 5.61E-10 | 8.55E-08 | CFAP58/TTC26/ZMYND10/DNAH11/DZIP1L/GAS8/TMEM107/DYNC2H1/TTC21A/LZTFL1/MAPK15/CCDC39/ROPN1B/CDK20/TCTN1/CC2D2A/TCTN2/WDR35/TULP1/ROPN1 |
| GO:0060632 | regulation of microtubule-based movement | 17/951 | 54/18800 | 6.47E-10 | 9.54E-08 | CCDC65/GAS2L2/DRC1/CFAP43/CFAP45/RSPH4A/TTLL6/DNAH11/CCDC40/DNAAF1/CFAP69/CFAP298/CCDC39/CNIH2/KIF9/CFAP206/EPPIN |
| GO:0060287 | epithelial cilium movement involved in determination of left/right asymmetry | 8/951 | 12/18800 | 1.72E-08 | 2.40E-06 | CFAP53/CFAP45/DNAH11/CCDC40/DNAAF1/RFX3/CCDC39/CCDC103 |
| GO:0003356 | regulation of cilium beat frequency | 9/951 | 16/18800 | 1.73E-08 | 2.40E-06 | GAS2L2/CFAP43/CFAP45/TTLL6/DNAH11/CCDC40/DNAAF1/CCDC39/CFAP206 |
| GO:0008544 | epidermis development | 44/951 | 355/18800 | 3.75E-08 | 5.04E-06 | TP63/CDH3/GRHL1/KRT4/WHRN/KRT15/KRT80/SLC44A4/GRHL2/IFT172/LRTOMT/KRT17/LGR5/FA2H/FUZ/COL7A1/INTU/FERMT1/SLITRK6/FZD3/OVOL2/KRT5/PTHLH/KLK14/PTCH2/SFN/ZNF750/IVL/SOX21/NGFR/HEY2/GRHL3/SOX9/WNT16/COL17A1/FOXN1/USH1C/SFRP4/WNT10A/CNFN/TMEM132E/EDAR/KRT6A/EDA2R |
| GO:0036158 | outer dynein arm assembly | 10/951 | 22/18800 | 3.88E-08 | 5.06E-06 | DNAL1/DAW1/DNAI2/DNAI1/ZMYND10/DNAH5/DNAAF4/DNAAF1/CCDC103/DNAAF2 |
| GO:0090660 | cerebrospinal fluid circulation | 8/951 | 14/18800 | 9.52E-08 | 1.21E-05 | SPAG16/DAW1/CFAP43/CFAP45/CFAP221/CFAP54/DNAH9/CCDC39 |
| GO:0007224 | smoothened signaling pathway | 23/951 | 140/18800 | 5.52E-07 | 6.81E-05 | TMEM231/FOXA1/TTC26/DZIP1L/GAS8/DYNC2H1/RPGRIP1L/B9D1/IFT172/CDK20/IFT81/TCTN1/IQUB/IFT27/CC2D2A/DCDC2/TCTN2/FUZ/INTU/TMEM17/PTCH2/NKX2-2/IHH |
| GO:0010970 | transport along microtubule | 24/951 | 157/18800 | 1.21E-06 | 0.000146 | TTC26/IFT46/LCA5L/MAK/DYNC2H1/IFT88/RABL2B/TTC21A/MAP1A/TTC30B/IFT172/IFT81/TRAF3IP1/IFT27/TTC30A/BICDL2/AGBL4/CNIH2/WDR35/BBS12/AP3B2/RAB27B/LCA5/KIF3A |
| GO:0048240 | sperm capacitation | 10/951 | 31/18800 | 1.75E-06 | 0.000205 | CATSPERD/ROPN1L/ROPN1B/PCSK4/CATSPERE/TCP11X1/CABYR/TCP11/ROPN1/SPINK1 |
| GO:0016331 | morphogenesis of embryonic epithelium | 23/951 | 150/18800 | 1.91E-06 | 0.000218 | SIX1/TP63/SIX4/WNT4/GDF7/GRHL2/IFT172/CDK20/TCTN1/CC2D2A/FUZ/WNT7B/FZD3/SALL4/OVOL2/RGMA/LRP2/RET/GREM1/CTHRC1/GRHL3/SOX9/WNT16 |
| GO:0048562 | embryonic organ morphogenesis | 35/951 | 294/18800 | 2.35E-06 | 0.000262 | SIX1/HOXB4/SIX4/EYA1/HOXC4/STOX1/HOXB3/WHRN/CCDC40/BBS5/SLC44A4/GRHL2/HOXB2/DNAAF1/IFT172/CCDC39/CCDC103/FUZ/SLITRK6/FZD3/FOXN4/OVOL2/IRX5/USH1G/DSCAML1/CTHRC1/GRHL3/SOX9/WNT16/HOXB1/IHH/USH1C/ALX1/STRA6/ATP6V1B1 |
| GO:0001838 | embryonic epithelial tube formation | 20/951 | 124/18800 | 3.99E-06 | 0.000434 | SIX1/SIX4/WNT4/GDF7/GRHL2/IFT172/CDK20/TCTN1/CC2D2A/FUZ/FZD3/SALL4/OVOL2/RGMA/LRP2/RET/GREM1/CTHRC1/GRHL3/SOX9 |
| GO:0001895 | retina homeostasis | 15/951 | 80/18800 | 9.85E-06 | 0.001046 | CDH3/RP1/WHRN/MAK/PIGR/BBS12/PROM1/LCA5/USH1G/TULP1/USH1C/ALB/POTEJ/SLC28A2/RDH12 |
| GO:0003002 | regionalization | 38/951 | 354/18800 | 1.04E-05 | 0.001084 | SIX1/HOXB4/TP63/HES2/FOXJ1/HOXC4/MNS1/FOXA1/BMPR1B/PIFO/DPCD/HOXB3/DZIP1L/TMEM107/DYNC2H1/HOXB2/DNAAF1/RPGRIP1L/IFT172/CDK20/TCTN1/CXXC4/LMX1B/FUZ/INTU/WNT7B/FOXN4/OVOL2/LRP2/CFC1/DSCAML1/HEY2/GREM1/NKX2-2/ISL1/HOXB1/CFC1B/MEOX1 |
| GO:0060562 | epithelial tube morphogenesis | 36/951 | 328/18800 | 1.09E-05 | 0.001104 | SIX1/SIX4/EYA1/STOX1/CCL11/FOXA1/WNT4/CCDC40/GDF7/BBS5/GRHL2/DNAAF1/MET/CSMD1/IFT172/CCDC39/CDK20/TCTN1/CC2D2A/CCDC103/LGR5/FUZ/FZD3/SALL4/FOXN4/OVOL2/RGMA/LRP2/RET/EPHA7/KIF26B/GREM1/CTHRC1/GRHL3/SOX9/IHH |
| GO:1905515 | non-motile cilium assembly | 13/951 | 63/18800 | 1.30E-05 | 0.001288 | RP1/TMEM107/MAK/DYNC2H1/IFT88/RPGRIP1L/IFT172/CC2D2A/CEP126/FUZ/INTU/TMEM17/CCDC13 |
| GO:0072175 | epithelial tube formation | 20/951 | 136/18800 | 1.66E-05 | 0.001611 | SIX1/SIX4/WNT4/GDF7/GRHL2/IFT172/CDK20/TCTN1/CC2D2A/FUZ/FZD3/SALL4/OVOL2/RGMA/LRP2/RET/GREM1/CTHRC1/GRHL3/SOX9 |
| GO:0060219 | camera-type eye photoreceptor cell differentiation | 8/951 | 25/18800 | 2.08E-05 | 0.001955 | RP1/RPGRIP1L/PROM1/SOX9/NTRK2/IHH/USH1C/RORB |
| GO:0001894 | tissue homeostasis | 31/951 | 272/18800 | 2.10E-05 | 0.001955 | CDH3/SPP1/RP1/TJP3/VSIG1/WHRN/IL7/MAK/PIGR/CLDN1/ILDR1/INAVA/BBS12/PROM1/LCA5/MUC4/USH1G/TULP1/SOX9/TFF3/CLDN3/COMP/IHH/HAMP/USH1C/ALB/POTEJ/MUC2/TNFSF11/SLC28A2/RDH12 |
| GO:0030705 | cytoskeleton-dependent intracellular transport | 25/951 | 197/18800 | 2.16E-05 | 0.00197 | MAP6/TTC26/IFT46/LCA5L/MAK/DYNC2H1/IFT88/RABL2B/TTC21A/MAP1A/TTC30B/IFT172/IFT81/TRAF3IP1/IFT27/TTC30A/BICDL2/AGBL4/CNIH2/WDR35/BBS12/AP3B2/RAB27B/LCA5/KIF3A |
| GO:0090102 | cochlea development | 11/951 | 49/18800 | 2.60E-05 | 0.002326 | SIX1/EYA1/RPGRIP1L/IFT27/SLITRK6/SLC17A8/HEY2/CTHRC1/GRHL3/SOX9/KCNK2 |
| GO:0035148 | tube formation | 21/951 | 152/18800 | 2.71E-05 | 0.002379 | SIX1/SIX4/WNT4/GDF7/GRHL2/IFT172/CDK20/TCTN1/CC2D2A/FUZ/FZD3/SALL4/OVOL2/RGMA/LRP2/RET/GREM1/CTHRC1/GRHL3/SOX9/EDAR |
| GO:0090596 | sensory organ morphogenesis | 30/951 | 266/18800 | 3.46E-05 | 0.00294 | SIX1/SIX4/EYA1/STOX1/RP1/WHRN/DZANK1/SLC44A4/RPGRIP1L/IFT172/AQP5/SDK1/INTU/SLITRK6/FZD3/FOXN4/PROM1/USH1G/CTHRC1/GRHL3/TULP1/SOX9/WNT16/TFAP2B/NTRK2/IHH/USH1C/STRA6/RORB/ATP6V1B1 |
| GO:0060249 | anatomical structure homeostasis | 34/951 | 319/18800 | 3.48E-05 | 0.00294 | CDH3/SPP1/NELL2/RP1/TJP3/VSIG1/WHRN/IL7/MAK/MAP1A/PIGR/CLDN1/ILDR1/INAVA/ATP2B3/BBS12/PROM1/LCA5/MUC4/USH1G/TULP1/SOX9/TFF3/CLDN3/COMP/IHH/HAMP/USH1C/ALB/POTEJ/MUC2/TNFSF11/SLC28A2/RDH12 |
| GO:0043588 | skin development | 32/951 | 296/18800 | 4.44E-05 | 0.003688 | TP63/CDH3/GRHL1/KRT4/KRT80/GRHL2/MET/KRT17/CLDN1/LGR5/FA2H/FUZ/INTU/FERMT1/FZD3/OVOL2/KRT5/PTCH2/SFN/IVL/SOX21/NGFR/GRHL3/SOX9/WNT16/TFAP2B/FOXN1/COMP/WNT10A/CNFN/EDAR/KRT6A |
| GO:0048568 | embryonic organ development | 43/951 | 449/18800 | 4.64E-05 | 0.003785 | SIX1/HOXB4/SIX4/EYA1/HOXC4/STOX1/E2F8/PIFO/HOXB3/WHRN/CCDC40/BBS5/SLC44A4/GRHL2/HOXB2/DNAAF1/IFT172/CCDC39/CDK20/CC2D2A/CCDC103/FUZ/WNT7B/SLITRK6/FZD3/FOXN4/OVOL2/IRX5/USH1G/DSCAML1/HEY2/CTHRC1/GRHL3/SOX9/WNT16/HOXB1/E2F7/IHH/USH1C/ALX1/IGF2/STRA6/ATP6V1B1 |
| GO:0031016 | pancreas development | 14/951 | 81/18800 | 5.01E-05 | 0.004013 | GIPR/CCDC40/DNAAF1/HNF4A/MET/RFX3/CCDC39/CDH2/HNF1A/NKX2-2/ISL1/SOX9/IHH/IGF2 |
| GO:0009953 | dorsal/ventral pattern formation | 15/951 | 92/18800 | 5.52E-05 | 0.004345 | FOXA1/BMPR1B/DYNC2H1/HOXB2/IFT172/CDK20/TCTN1/CXXC4/LMX1B/INTU/FOXN4/OVOL2/DSCAML1/GREM1/NKX2-2 |
| GO:0032886 | regulation of microtubule-based process | 28/951 | 249/18800 | 6.57E-05 | 0.005039 | CCDC65/GAS2L2/STMND1/DRC1/MAPRE3/CFAP43/MAP6/CFAP45/RSPH4A/SPEF1/TTLL6/DNAH11/TRPV4/CCDC40/TMEM67/DNAAF1/CFAP69/CFAP298/MET/MAP1A/MDM1/MAPK15/CCDC39/TRAF3IP1/CNIH2/KIF9/CFAP206/EPPIN |
| GO:0001754 | eye photoreceptor cell differentiation | 10/951 | 45/18800 | 6.62E-05 | 0.005039 | RP1/DZANK1/RPGRIP1L/PROM1/TULP1/SOX9/NTRK2/IHH/USH1C/RORB |
| GO:0048732 | gland development | 41/951 | 431/18800 | 8.03E-05 | 0.006013 | SIX1/TP63/SIX4/INHBB/CCL11/FOXA1/E2F8/WNT4/HOXB3/SOX2/CCDC40/GDF7/DNAAF1/RPGRIP1L/HNF4A/MET/OXTR/CSMD1/CCDC39/CLDN1/FA2H/WNT7B/PITX1/HNF1A/ERBB4/BAAT/CIT/XDH/SERPINB5/ISL1/SOX9/E2F7/SLC6A3/FOXN1/IHH/HAMP/IGF2/WNT10A/STRA6/EDAR/TNFSF11 |
| GO:0048880 | sensory system development | 38/951 | 389/18800 | 8.33E-05 | 0.006134 | RP1/TMEM231/BMPR1B/SOX2/DZANK1/NPHP1/SLC44A4/GRHL2/RPGRIP1L/MDM1/B9D1/IFT172/AQP5/CDK20/CC2D2A/SDK1/WNT7B/SLITRK6/BIRC7/FOXN4/PROM1/RET/SLC17A8/GRHL3/TULP1/ISL1/SOX9/WNT16/TFAP2B/SLC4A5/NTRK2/SLC6A3/IHH/USH1C/STRA6/TMEM132E/RORB/ADAMTS18 |
| GO:0042490 | mechanoreceptor differentiation | 12/951 | 65/18800 | 8.82E-05 | 0.006391 | WHRN/SLC44A4/IFT88/LRTOMT/IFT27/SLITRK6/USH1G/HEY2/CTHRC1/NTRK2/USH1C/TMEM132E |
| GO:0009913 | epidermal cell differentiation | 26/951 | 230/18800 | 0.000108 | 0.007697 | TP63/CDH3/GRHL1/KRT4/WHRN/KRT80/SLC44A4/GRHL2/LRTOMT/KRT17/FA2H/INTU/SLITRK6/OVOL2/KRT5/PTCH2/SFN/IVL/HEY2/WNT16/FOXN1/USH1C/SFRP4/CNFN/TMEM132E/KRT6A |
| GO:0031503 | protein-containing complex localization | 20/951 | 155/18800 | 0.000111 | 0.007697 | TTC26/IFT46/LCA5L/MAK/DYNC2H1/IFT88/RABL2B/TTC21A/TTC30B/IFT172/IFT81/TRAF3IP1/IFT27/TTC30A/CNIH2/WDR35/BBS12/LCA5/SHISA6/NPTX1 |
| GO:0048839 | inner ear development | 23/951 | 192/18800 | 0.000111 | 0.007697 | SIX1/SIX4/EYA1/STOX1/WHRN/SOX2/SLC44A4/IFT88/RPGRIP1L/LRTOMT/IFT27/LGR5/SLITRK6/FZD3/USH1G/SLC17A8/HEY2/CTHRC1/GRHL3/SOX9/KCNK2/USH1C/ATP6V1B1 |
| GO:0060042 | retina morphogenesis in camera-type eye | 11/951 | 57/18800 | 0.000114 | 0.007744 | RP1/RPGRIP1L/SDK1/FOXN4/PROM1/SOX9/TFAP2B/NTRK2/IHH/USH1C/RORB |
| GO:0060221 | retinal rod cell differentiation | 5/951 | 11/18800 | 0.000117 | 0.007875 | RP1/RPGRIP1L/SOX9/NTRK2/RORB |
| GO:0001942 | hair follicle development | 14/951 | 89/18800 | 0.000144 | 0.009534 | TP63/CDH3/KRT17/LGR5/FUZ/INTU/FERMT1/FZD3/SOX21/NGFR/SOX9/FOXN1/WNT10A/EDAR |
| GO:0050891 | multicellular organismal water homeostasis | 11/951 | 59/18800 | 0.000157 | 0.010252 | TP63/GRHL1/TRPV4/MET/CLDN1/FA2H/SCNN1G/SFN/GRHL3/TFAP2B/ATP6V1B1 |
| GO:0061436 | establishment of skin barrier | 7/951 | 25/18800 | 0.000179 | 0.011484 | TP63/GRHL1/MET/CLDN1/FA2H/SFN/GRHL3 |
| GO:0003407 | neural retina development | 12/951 | 70/18800 | 0.000185 | 0.01171 | RP1/RPGRIP1L/SDK1/FOXN4/PROM1/SLC17A8/SOX9/TFAP2B/NTRK2/IHH/USH1C/RORB |
| GO:0042303 | molting cycle | 16/951 | 114/18800 | 0.000198 | 0.012199 | TP63/CDH3/KRT17/LGR5/FA2H/FUZ/INTU/FERMT1/FZD3/PTCH2/SOX21/NGFR/SOX9/FOXN1/WNT10A/EDAR |
| GO:0042633 | hair cycle | 16/951 | 114/18800 | 0.000198 | 0.012199 | TP63/CDH3/KRT17/LGR5/FA2H/FUZ/INTU/FERMT1/FZD3/PTCH2/SOX21/NGFR/SOX9/FOXN1/WNT10A/EDAR |
| GO:0022404 | molting cycle process | 14/951 | 92/18800 | 0.000207 | 0.012415 | TP63/CDH3/KRT17/LGR5/FUZ/INTU/FERMT1/FZD3/SOX21/NGFR/SOX9/FOXN1/WNT10A/EDAR |
| GO:0022405 | hair cycle process | 14/951 | 92/18800 | 0.000207 | 0.012415 | TP63/CDH3/KRT17/LGR5/FUZ/INTU/FERMT1/FZD3/SOX21/NGFR/SOX9/FOXN1/WNT10A/EDAR |
| GO:0008589 | regulation of smoothened signaling pathway | 13/951 | 82/18800 | 0.000227 | 0.013492 | FOXA1/GAS8/DYNC2H1/RPGRIP1L/IFT172/CDK20/IFT81/TCTN1/DCDC2/FUZ/INTU/PTCH2/IHH |
| GO:0098773 | skin epidermis development | 14/951 | 93/18800 | 0.000232 | 0.013587 | TP63/CDH3/KRT17/LGR5/FUZ/INTU/FERMT1/FZD3/SOX21/NGFR/SOX9/FOXN1/WNT10A/EDAR |
| GO:0048705 | skeletal system morphogenesis | 25/951 | 228/18800 | 0.000235 | 0.013587 | SIX1/HOXB4/BARX2/SIX4/EYA1/HOXC4/BMPR1B/SPEF2/HOXB3/TRPV4/FGFR3/TMEM107/ATG9B/GRHL2/HOXB2/FUZ/IRX5/DSCAML1/GREM1/SOX9/HOXB1/COMP/IHH/ALX1/SFRP4 |
| GO:0045494 | photoreceptor cell maintenance | 9/951 | 43/18800 | 0.000249 | 0.01419 | RP1/MAK/BBS12/PROM1/LCA5/USH1G/TULP1/USH1C/RDH12 |
| GO:0043583 | ear development | 24/951 | 219/18800 | 0.000313 | 0.017654 | SIX1/SIX4/EYA1/STOX1/WHRN/SOX2/SLC44A4/IFT88/RPGRIP1L/LRTOMT/IFT27/LGR5/SLITRK6/FZD3/USH1G/SLC17A8/HEY2/CTHRC1/GRHL3/SOX9/KCNK2/USH1C/STRA6/ATP6V1B1 |
| GO:0043010 | camera-type eye development | 32/951 | 330/18800 | 0.000336 | 0.018689 | RP1/TMEM231/BMPR1B/NPHP1/GRHL2/RPGRIP1L/MDM1/B9D1/IFT172/AQP5/CDK20/CC2D2A/SDK1/WNT7B/SLITRK6/BIRC7/FOXN4/PROM1/RET/SLC17A8/GRHL3/TULP1/SOX9/WNT16/TFAP2B/SLC4A5/NTRK2/SLC6A3/IHH/USH1C/STRA6/RORB |
| GO:0060428 | lung epithelium development | 8/951 | 36/18800 | 0.000355 | 0.019542 | FOXJ1/FOXA1/GRHL2/AGR2/WNT7B/SPDEF/SOX9/STRA6 |
| GO:1902017 | regulation of cilium assembly | 11/951 | 65/18800 | 0.000381 | 0.02072 | MNS1/RP1/KIF24/ZMYND10/MAK/IFT88/MAPK15/DCDC2/FUZ/MCIDAS/SAXO1 |
| GO:0010817 | regulation of hormone levels | 43/951 | 496/18800 | 0.00042 | 0.022551 | INHBB/SPP1/CHST9/PCSK1N/FOXA1/WNT4/TRPV4/GIPR/BAIAP3/SRI/HNF4A/RFX3/SRD5A2/PCSK4/PLA2G7/ILDR1/ADH6/BCO2/CYP11A1/CYP2W1/DIO2/CRYM/TRPV6/HNF1A/CYP27C1/UCN3/ADH1C/ADRA2A/GHRL/DIO1/HSD17B2/ISL1/TFAP2B/CLTRN/TTR/ABAT/GDF9/TNFSF11/HTR2C/SPINK1/RDH12/ADH7/FAM3D |
| GO:0030104 | water homeostasis | 11/951 | 66/18800 | 0.000437 | 0.023193 | TP63/GRHL1/TRPV4/MET/CLDN1/FA2H/SCNN1G/SFN/GRHL3/TFAP2B/ATP6V1B1 |
| GO:0001654 | eye development | 35/951 | 379/18800 | 0.000453 | 0.023772 | RP1/TMEM231/BMPR1B/SOX2/DZANK1/NPHP1/GRHL2/RPGRIP1L/MDM1/B9D1/IFT172/AQP5/CDK20/CC2D2A/SDK1/WNT7B/SLITRK6/BIRC7/FOXN4/PROM1/RET/SLC17A8/GRHL3/TULP1/SOX9/WNT16/TFAP2B/SLC4A5/NTRK2/SLC6A3/IHH/USH1C/STRA6/RORB/ADAMTS18 |
| GO:0033561 | regulation of water loss via skin | 7/951 | 29/18800 | 0.000486 | 0.02464 | TP63/GRHL1/MET/CLDN1/FA2H/SFN/GRHL3 |
| GO:0060295 | regulation of cilium movement involved in cell motility | 7/951 | 29/18800 | 0.000486 | 0.02464 | GAS2L2/CFAP45/TTLL6/CFAP69/KIF9/CFAP206/EPPIN |
| GO:1902019 | regulation of cilium-dependent cell motility | 7/951 | 29/18800 | 0.000486 | 0.02464 | GAS2L2/CFAP45/TTLL6/CFAP69/KIF9/CFAP206/EPPIN |
| GO:0060041 | retina development in camera-type eye | 18/951 | 149/18800 | 0.000539 | 0.027062 | RP1/BMPR1B/NPHP1/RPGRIP1L/MDM1/SDK1/FOXN4/PROM1/RET/SLC17A8/TULP1/SOX9/TFAP2B/SLC4A5/NTRK2/IHH/USH1C/RORB |
| GO:0150063 | visual system development | 35/951 | 383/18800 | 0.000549 | 0.02723 | RP1/TMEM231/BMPR1B/SOX2/DZANK1/NPHP1/GRHL2/RPGRIP1L/MDM1/B9D1/IFT172/AQP5/CDK20/CC2D2A/SDK1/WNT7B/SLITRK6/BIRC7/FOXN4/PROM1/RET/SLC17A8/GRHL3/TULP1/SOX9/WNT16/TFAP2B/SLC4A5/NTRK2/SLC6A3/IHH/USH1C/STRA6/RORB/ADAMTS18 |
| GO:0021591 | ventricular system development | 7/951 | 30/18800 | 0.000606 | 0.029097 | NME5/AK8/DPCD/DNAH5/HYDIN/RPGRIP1L/KIF27 |
| GO:0030277 | maintenance of gastrointestinal epithelium | 6/951 | 22/18800 | 0.000612 | 0.029097 | VSIG1/INAVA/MUC4/SOX9/TFF3/MUC2 |
| GO:0060487 | lung epithelial cell differentiation | 6/951 | 22/18800 | 0.000612 | 0.029097 | FOXJ1/FOXA1/GRHL2/AGR2/SPDEF/SOX9 |
| GO:0060972 | left/right pattern formation | 6/951 | 22/18800 | 0.000612 | 0.029097 | FOXJ1/MNS1/PIFO/DPCD/DNAAF1/IFT172 |
| GO:0042472 | inner ear morphogenesis | 14/951 | 103/18800 | 0.000675 | 0.031769 | SIX1/SIX4/EYA1/STOX1/WHRN/SLC44A4/SLITRK6/FZD3/USH1G/CTHRC1/GRHL3/SOX9/USH1C/ATP6V1B1 |
| GO:0021675 | nerve development | 12/951 | 81/18800 | 0.000737 | 0.03433 | SIX1/SIX4/HOXB3/HOXB2/SLITRK6/NTF3/RET/NPTX1/NGFR/NKX2-2/ISL1/HOXB1 |
| GO:0001841 | neural tube formation | 14/951 | 104/18800 | 0.000744 | 0.03433 | GDF7/GRHL2/IFT172/CDK20/TCTN1/CC2D2A/FUZ/FZD3/SALL4/OVOL2/RGMA/LRP2/CTHRC1/GRHL3 |
| GO:0060479 | lung cell differentiation | 6/951 | 23/18800 | 0.000792 | 0.036155 | FOXJ1/FOXA1/GRHL2/AGR2/SPDEF/SOX9 |
| GO:0021915 | neural tube development | 18/951 | 154/18800 | 0.0008 | 0.036155 | FOXA1/DZIP1L/GDF7/TMEM107/GRHL2/RPGRIP1L/IFT172/CDK20/TCTN1/CC2D2A/FUZ/FZD3/SALL4/OVOL2/RGMA/LRP2/CTHRC1/GRHL3 |
| GO:0048704 | embryonic skeletal system morphogenesis | 13/951 | 94/18800 | 0.000879 | 0.039357 | SIX1/HOXB4/SIX4/EYA1/HOXC4/HOXB3/GRHL2/HOXB2/FUZ/IRX5/DSCAML1/HOXB1/ALX1 |
| GO:0010669 | epithelial structure maintenance | 7/951 | 32/18800 | 0.000917 | 0.04067 | VSIG1/ILDR1/INAVA/MUC4/SOX9/TFF3/MUC2 |
| GO:0120254 | olefinic compound metabolic process | 18/951 | 157/18800 | 0.001003 | 0.044005 | WNT4/CYP2J2/CYP2F1/FAAH2/GRIN1/SRD5A2/ADH6/GSTA1/BCO2/CYP11A1/CYP27C1/CYP2A13/ADH1C/ALOX15/TTR/CYP2G1P/RDH12/ADH7 |
| GO:0001964 | startle response | 6/951 | 24/18800 | 0.001012 | 0.044005 | GLRB/CSMD1/SLITRK6/PENK/SLC6A3/DRD1 |
| GO:0046530 | photoreceptor cell differentiation | 10/951 | 62/18800 | 0.001025 | 0.044169 | RP1/DZANK1/RPGRIP1L/PROM1/TULP1/SOX9/NTRK2/IHH/USH1C/RORB |

| **Supplemental Table 4b GO enrichment analysis (biological process, BP) of down-regulated genes** | | | | | | |
| --- | --- | --- | --- | --- | --- | --- |
| **ID** | **Description** | **Gene**  **Ratio** | **Bg**  **Ratio** | **pvalue** | **p.adjust** | **geneID** |
| GO:0032103 | positive regulation of response to external stimulus | 39/449 | 442/18800 | 2.18E-12 | 8.97E-09 | LPL/CADM1/SEMA5A/IL1RL1/FPR2/FABP4/OASL/PGC/KLRD1/CCL4/NLRC4/AGTR1/IL1B/SASH1/TLR8/NLRP12/S100A12/BMP6/SH2D1B/ALOX5AP/AGER/FFAR2/S100A8/MNDA/EDN1/THBD/LRRK2/ACE/TNF/GPR4/LILRA2/TXK/LILRA5/ARG1/SERPINE1/CPB2/IFNG/CX3CR1/NKG7 |
| GO:0050727 | regulation of inflammatory response | 35/449 | 394/18800 | 2.56E-11 | 5.26E-08 | LPL/EDNRB/IL1RL1/FPR2/FFAR4/FABP4/CASP12/AGTR1/FOXF1/MEFV/FGR/IL1B/NLRP12/S100A12/ALOX5AP/BST1/AGER/CDH5/FFAR2/S100A8/GGT1/CST7/GPER1/LRRK2/ACE/TNF/GPR4/TEK/LILRA5/CALCRL/CASP5/SERPINE1/IFNG/NKG7/NCF1 |
| GO:0031349 | positive regulation of defense response | 29/449 | 289/18800 | 7.66E-11 | 1.05E-07 | LPL/CADM1/IL1RL1/FPR2/FABP4/PGC/KLRD1/NLRC4/AGTR1/IL1B/TLR8/NLRP12/S100A12/SH2D1B/ALOX5AP/FFAR2/S100A8/MNDA/LRRK2/ACE/TNF/GPR4/LILRA2/TXK/LILRA5/ARG1/SERPINE1/IFNG/NKG7 |
| GO:0051480 | regulation of cytosolic calcium ion concentration | 31/449 | 356/18800 | 5.96E-10 | 6.13E-07 | UBASH3B/EDNRA/TRPV2/EDNRB/MCOLN3/KCNK3/SLC24A4/FPR2/TRPC3/FFAR4/HAP1/AGTR1/GRIA1/ADCY8/P2RX7/RAMP3/CXCR2/ADRA1D/MCOLN2/NOS1/FPR1/P2RY1/F2RL3/CD52/EDN1/GPER1/GPR4/PLA2G1B/GPR17/CXCR1/CX3CR1 |
| GO:0001819 | positive regulation of cytokine production | 36/449 | 475/18800 | 1.10E-09 | 8.45E-07 | LPL/CADM1/FLT4/IL1RL1/SPN/HEG1/NLRC4/SLC11A1/MEFV/FGR/IL1A/TBX21/P2RX7/IL17D/IL1B/TLR8/NLRP12/NOS2/MCOLN2/ALOX15B/AGER/IL18R1/FFAR2/MNDA/LILRB2/CLEC4E/LRRK2/TNF/LILRA2/PLA2G1B/PTPN22/TXK/LILRA5/SERPINE1/IFNG/ORM1 |
| GO:0007204 | positive regulation of cytosolic calcium ion concentration | 29/449 | 325/18800 | 1.23E-09 | 8.45E-07 | UBASH3B/EDNRA/TRPV2/EDNRB/MCOLN3/SLC24A4/FPR2/TRPC3/FFAR4/HAP1/AGTR1/ADCY8/P2RX7/RAMP3/CXCR2/ADRA1D/MCOLN2/NOS1/FPR1/P2RY1/F2RL3/CD52/EDN1/GPER1/GPR4/PLA2G1B/GPR17/CXCR1/CX3CR1 |
| GO:0032731 | positive regulation of interleukin-1 beta production | 13/449 | 62/18800 | 1.98E-09 | 1.17E-06 | LPL/NLRC4/MEFV/P2RX7/TLR8/NLRP12/AGER/MNDA/TNF/LILRA2/LILRA5/IFNG/ORM1 |
| GO:0072503 | cellular divalent inorganic cation homeostasis | 36/449 | 494/18800 | 3.14E-09 | 1.62E-06 | UBASH3B/EDNRA/TRPV2/EDNRB/MCOLN3/KCNK3/SLC24A4/FPR2/TRPC3/FFAR4/SLC39A8/HAP1/AGTR1/SLC11A1/GRIA1/ADCY8/P2RX7/RAMP3/CXCR2/ADRA1D/MCOLN2/NOS1/STC2/FPR1/P2RY1/CDH5/F2RL3/S100A8/CD52/EDN1/GPER1/GPR4/PLA2G1B/GPR17/CXCR1/CX3CR1 |
| GO:0001906 | cell killing | 21/449 | 185/18800 | 3.65E-09 | 1.67E-06 | CADM1/CD5L/TREM1/KLRD1/IL7R/EMP2/UNC13D/P2RX7/FCGR3A/NOS2/S100A12/PRF1/GNLY/AGER/GZMB/ICAM1/ARG1/GZMM/IFNG/CX3CR1/NKG7 |
| GO:0003018 | vascular process in circulatory system | 25/449 | 263/18800 | 4.87E-09 | 2.00E-06 | EDNRA/EDNRB/ITGA1/AGTR1/GJA5/SLC2A3/MFSD2A/SLC22A8/NPR1/CLDN5/ADRA1D/NOS1/ADRB2/BMP6/P2RY1/AGER/CDH5/EDN1/SLC6A4/GPER1/SLC5A1/ACE/TNF/GPR4/TEK |
| GO:0050729 | positive regulation of inflammatory response | 18/449 | 145/18800 | 1.17E-08 | 4.38E-06 | LPL/IL1RL1/FABP4/AGTR1/IL1B/NLRP12/S100A12/ALOX5AP/FFAR2/S100A8/LRRK2/ACE/TNF/GPR4/LILRA5/SERPINE1/IFNG/NKG7 |
| GO:0001909 | leukocyte mediated cytotoxicity | 17/449 | 131/18800 | 1.52E-08 | 5.08E-06 | CADM1/TREM1/KLRD1/IL7R/EMP2/UNC13D/P2RX7/FCGR3A/NOS2/PRF1/AGER/GZMB/ICAM1/ARG1/GZMM/CX3CR1/NKG7 |
| GO:0032732 | positive regulation of interleukin-1 production | 13/449 | 73/18800 | 1.62E-08 | 5.08E-06 | LPL/NLRC4/MEFV/P2RX7/TLR8/NLRP12/AGER/MNDA/TNF/LILRA2/LILRA5/IFNG/ORM1 |
| GO:0006874 | cellular calcium ion homeostasis | 33/449 | 456/18800 | 1.73E-08 | 5.08E-06 | UBASH3B/EDNRA/TRPV2/EDNRB/MCOLN3/KCNK3/SLC24A4/FPR2/TRPC3/FFAR4/HAP1/AGTR1/GRIA1/ADCY8/P2RX7/RAMP3/CXCR2/ADRA1D/MCOLN2/NOS1/STC2/FPR1/P2RY1/CDH5/F2RL3/CD52/EDN1/GPER1/GPR4/PLA2G1B/GPR17/CXCR1/CX3CR1 |
| GO:0034472 | snRNA 3'-end processing | 9/449 | 31/18800 | 2.95E-08 | 8.09E-06 | CT45A8/CT45A7/CT45A2/CT45A3/CT45A9/CT45A6/CT45A5/CT45A1/CT45A10 |
| GO:0055074 | calcium ion homeostasis | 33/449 | 468/18800 | 3.23E-08 | 8.30E-06 | UBASH3B/EDNRA/TRPV2/EDNRB/MCOLN3/KCNK3/SLC24A4/FPR2/TRPC3/FFAR4/HAP1/AGTR1/GRIA1/ADCY8/P2RX7/RAMP3/CXCR2/ADRA1D/MCOLN2/NOS1/STC2/FPR1/P2RY1/CDH5/F2RL3/CD52/EDN1/GPER1/GPR4/PLA2G1B/GPR17/CXCR1/CX3CR1 |
| GO:0032611 | interleukin-1 beta production | 15/449 | 110/18800 | 5.49E-08 | 1.26E-05 | LPL/FFAR4/NLRC4/MEFV/P2RX7/TLR8/NLRP12/AGER/MNDA/TNF/LILRA2/LILRA5/IFNG/CX3CR1/ORM1 |
| GO:0032651 | regulation of interleukin-1 beta production | 15/449 | 110/18800 | 5.49E-08 | 1.26E-05 | LPL/FFAR4/NLRC4/MEFV/P2RX7/TLR8/NLRP12/AGER/MNDA/TNF/LILRA2/LILRA5/IFNG/CX3CR1/ORM1 |
| GO:0032612 | interleukin-1 production | 16/449 | 129/18800 | 7.73E-08 | 1.59E-05 | LPL/FFAR4/NLRC4/MEFV/P2RX7/TLR8/NLRP12/AGER/MNDA/TNF/LILRA2/LILRA5/IFNG/IL1R2/CX3CR1/ORM1 |
| GO:0032652 | regulation of interleukin-1 production | 16/449 | 129/18800 | 7.73E-08 | 1.59E-05 | LPL/FFAR4/NLRC4/MEFV/P2RX7/TLR8/NLRP12/AGER/MNDA/TNF/LILRA2/LILRA5/IFNG/IL1R2/CX3CR1/ORM1 |
| GO:0003158 | endothelium development | 16/449 | 133/18800 | 1.19E-07 | 2.32E-05 | EDNRA/EDNRB/NOTCH4/HEG1/DLL4/GJA5/IL1B/ACVRL1/CLDN5/ROBO4/BMP6/CDH5/TMEM100/TNF/ICAM1/COL22A1 |
| GO:0016180 | snRNA processing | 9/449 | 36/18800 | 1.24E-07 | 2.32E-05 | CT45A8/CT45A7/CT45A2/CT45A3/CT45A9/CT45A6/CT45A5/CT45A1/CT45A10 |
| GO:0032496 | response to lipopolysaccharide | 26/449 | 333/18800 | 1.35E-07 | 2.41E-05 | EDNRB/HPGD/SMAD6/SLC11A1/IL1A/P2RX7/IL1B/SASH1/CSF2RB/NOS2/NOS1/BMP6/MRC1/S100A8/CAMP/EDN1/LILRB2/THBD/ACE/TNF/LILRA2/PTPN22/ARG1/SERPINE1/ALPL/CX3CR1 |
| GO:0007200 | phospholipase C-activating G protein-coupled receptor signaling pathway | 14/449 | 104/18800 | 1.80E-07 | 2.94E-05 | EDNRB/FPR2/FFAR4/AGTR1/CXCR2/ADRA1D/FPR1/P2RY1/F2RL3/EDN1/GPR4/GPR17/CHRM1/CX3CR1 |
| GO:0050900 | leukocyte migration | 28/449 | 384/18800 | 1.84E-07 | 2.94E-05 | EDNRB/TREM1/FPR2/SPN/ITGA1/CCL4/SELPLG/IL1A/TBX21/IL1B/MYO1G/CXCR2/NLRP12/MCOLN2/S100A12/PREX1/BST1/AGER/FFAR2/S100A8/EDN1/TNF/PLA2G1B/ICAM1/CXCR1/SERPINE1/CD300H/CX3CR1 |
| GO:0043270 | positive regulation of ion transport | 23/449 | 273/18800 | 1.86E-07 | 2.94E-05 | EDNRA/TRPV2/TRPC3/CCL4/HAP1/ARC/IL1A/P2RX7/RAMP3/IL1B/NOS1/ADRB2/P2RY1/STAC/F2RL3/EDN1/SLC6A4/GPER1/LILRA2/PLA2G1B/LILRA5/CHRM1/IFNG |
| GO:0001885 | endothelial cell development | 11/449 | 64/18800 | 3.04E-07 | 4.62E-05 | EDNRA/EDNRB/NOTCH4/HEG1/IL1B/CLDN5/ROBO4/CDH5/TNF/ICAM1/COL22A1 |
| GO:1990266 | neutrophil migration | 15/449 | 128/18800 | 4.17E-07 | 6.13E-05 | TREM1/ITGA1/CCL4/IL1A/IL1B/CXCR2/MCOLN2/S100A12/PREX1/BST1/S100A8/EDN1/PLA2G1B/CXCR1/CD300H |
| GO:0002237 | response to molecule of bacterial origin | 26/449 | 354/18800 | 4.42E-07 | 6.14E-05 | EDNRB/HPGD/SMAD6/SLC11A1/IL1A/P2RX7/IL1B/SASH1/CSF2RB/NOS2/NOS1/BMP6/MRC1/S100A8/CAMP/EDN1/LILRB2/THBD/ACE/TNF/LILRA2/PTPN22/ARG1/SERPINE1/ALPL/CX3CR1 |
| GO:0007188 | adenylate cyclase-modulating G protein-coupled receptor signaling pathway | 21/449 | 244/18800 | 4.48E-07 | 6.14E-05 | ADGRE1/EDNRA/VIPR1/FPR2/ADGRL2/ADCY8/ADGRE3/RAMP3/ADRA1D/ADRB2/ADGRG2/FPR1/S1PR5/P2RY1/EDN1/GPER1/PSAPL1/GPR4/GHRHR/CHRM1/CALCRL |
| GO:0032755 | positive regulation of interleukin-6 production | 13/449 | 97/18800 | 5.21E-07 | 6.91E-05 | LPL/IL1A/P2RX7/IL17D/IL1B/TLR8/NOS2/AGER/LILRB2/TNF/LILRA2/LILRA5/IFNG |
| GO:0002443 | leukocyte mediated immunity | 30/449 | 457/18800 | 6.37E-07 | 8.18E-05 | CADM1/TREM1/KLRD1/IL7R/C1QA/EMP2/UNC13D/FOXF1/FGR/TBX21/P2RX7/IL1B/MYO1G/FCGR3A/TLR8/CSF2RB/NOS2/SH2D1B/PRF1/AGER/IL18R1/GZMB/ACE/TNF/PLA2G1B/ICAM1/ARG1/GZMM/CX3CR1/NKG7 |
| GO:0097529 | myeloid leukocyte migration | 20/449 | 229/18800 | 6.67E-07 | 8.32E-05 | EDNRB/TREM1/FPR2/ITGA1/CCL4/IL1A/IL1B/CXCR2/MCOLN2/S100A12/PREX1/BST1/AGER/S100A8/EDN1/PLA2G1B/CXCR1/SERPINE1/CD300H/CX3CR1 |
| GO:0045446 | endothelial cell differentiation | 14/449 | 117/18800 | 7.85E-07 | 9.49E-05 | EDNRA/EDNRB/NOTCH4/HEG1/IL1B/ACVRL1/CLDN5/ROBO4/BMP6/CDH5/TMEM100/TNF/ICAM1/COL22A1 |
| GO:0003007 | heart morphogenesis | 21/449 | 254/18800 | 8.66E-07 | 0.000102 | EDNRA/HEYL/TBX5/TBX3/HEG1/DLL4/FOXF1/TTN/SMAD6/NDRG4/GJA5/ACVRL1/CLDN5/TGFBR3/TNNC1/TMEM100/EDN1/MYBPC3/TEK/TCAP/MSX1 |
| GO:0030856 | regulation of epithelial cell differentiation | 16/449 | 157/18800 | 1.16E-06 | 0.00013 | NOTCH4/TBX3/CDKN2B/KLF7/IL1A/IL1B/ACVRL1/CLDN5/ALOX15B/BMP6/CDH5/TMEM100/PRLR/TNF/SERPINE1/IFNG |
| GO:0019932 | second-messenger-mediated signaling | 23/449 | 303/18800 | 1.17E-06 | 0.00013 | EDNRB/SLC24A4/FPR2/FFAR4/GUCY1A2/CCL4/AGTR1/P2RX7/NPR1/CXCR2/NOS2/MYOZ1/NOS1/SAMD14/FPR1/BST1/TMEM100/EDN1/LRRK2/TNF/GHRHR/CXCR1/CX3CR1 |
| GO:1901342 | regulation of vasculature development | 25/449 | 351/18800 | 1.29E-06 | 0.00014 | SEMA5A/BMPER/COL4A3/CEMIP2/COL4A2/EMP2/IL1A/IL1B/ACVRL1/NPR1/SASH1/CLDN5/SEMA6A/JCAD/HYAL1/ANGPTL7/FUT1/CDH5/ANGPT4/CAMP/TNF/GPR4/TEK/SERPINE1/CX3CR1 |
| GO:0002697 | regulation of immune effector process | 25/449 | 353/18800 | 1.43E-06 | 0.000143 | CADM1/COLEC10/CD5L/PGC/KLRD1/IL7R/UNC13D/FOXF1/FGR/TBX21/P2RX7/IL1B/NOS2/SH2D1B/AGER/IL18R1/FFAR2/FCN3/TNF/ICAM1/PTPN22/ARG1/IFNG/CX3CR1/NCF1 |
| GO:0035296 | regulation of tube diameter | 15/449 | 141/18800 | 1.46E-06 | 0.000143 | EDNRA/EDNRB/ITGA1/AGTR1/GJA5/NPR1/ADRA1D/NOS1/ADRB2/P2RY1/EDN1/SLC6A4/GPER1/ACE/TNF |
| GO:0097746 | blood vessel diameter maintenance | 15/449 | 141/18800 | 1.46E-06 | 0.000143 | EDNRA/EDNRB/ITGA1/AGTR1/GJA5/NPR1/ADRA1D/NOS1/ADRB2/P2RY1/EDN1/SLC6A4/GPER1/ACE/TNF |
| GO:0030593 | neutrophil chemotaxis | 13/449 | 106/18800 | 1.46E-06 | 0.000143 | TREM1/ITGA1/CCL4/IL1B/CXCR2/S100A12/PREX1/BST1/S100A8/EDN1/PLA2G1B/CXCR1/CD300H |
| GO:0035150 | regulation of tube size | 15/449 | 142/18800 | 1.59E-06 | 0.000152 | EDNRA/EDNRB/ITGA1/AGTR1/GJA5/NPR1/ADRA1D/NOS1/ADRB2/P2RY1/EDN1/SLC6A4/GPER1/ACE/TNF |
| GO:0060326 | cell chemotaxis | 23/449 | 315/18800 | 2.26E-06 | 0.000212 | SEMA5A/EDNRB/TREM1/PLXNB3/FPR2/ITGA1/BIN2/CCL4/AGTR1/IL1B/CXCR2/S100A12/PREX1/BST1/FFAR2/S100A8/EDN1/PLA2G1B/CXCR1/SERPINE1/HBEGF/CD300H/CX3CR1 |
| GO:0043628 | ncRNA 3'-end processing | 9/449 | 50/18800 | 2.45E-06 | 0.000224 | CT45A8/CT45A7/CT45A2/CT45A3/CT45A9/CT45A6/CT45A5/CT45A1/CT45A10 |
| GO:0002456 | T cell mediated immunity | 13/449 | 112/18800 | 2.75E-06 | 0.000243 | KLRD1/IL7R/EMP2/TBX21/P2RX7/IL1B/MYO1G/PRF1/AGER/IL18R1/ICAM1/ARG1/GZMM |
| GO:0043410 | positive regulation of MAPK cascade | 30/449 | 491/18800 | 2.78E-06 | 0.000243 | FLT4/BMPER/FPR2/FFAR4/ITGA1/CCL4/WNT7A/NDRG4/IL1A/P2RX7/RAMP3/IL1B/SASH1/ADRA1D/S100A12/ADRB2/JCAD/P2RY1/AGER/EDN1/GPER1/LRRK2/TNF/PLA2G1B/GPBAR1/ICAM1/TEK/PTPN22/LILRA5/NCF1 |
| GO:1904659 | glucose transmembrane transport | 13/449 | 113/18800 | 3.04E-06 | 0.00026 | EDNRA/ENPP1/FFAR4/SLC2A6/SLC2A3/IL1B/EDN1/SLC5A4/SLC5A1/ACE/TNF/PLA2G1B/SLC2A14 |
| GO:0045765 | regulation of angiogenesis | 24/449 | 345/18800 | 3.17E-06 | 0.000266 | SEMA5A/BMPER/COL4A3/CEMIP2/COL4A2/EMP2/IL1A/IL1B/ACVRL1/NPR1/SASH1/CLDN5/SEMA6A/JCAD/HYAL1/FUT1/CDH5/ANGPT4/CAMP/TNF/GPR4/TEK/SERPINE1/CX3CR1 |
| GO:0002274 | myeloid leukocyte activation | 19/449 | 232/18800 | 3.30E-06 | 0.000272 | IL1RL1/FPR2/C1QA/UNC13D/FOXF1/SLC11A1/FGR/CXCR2/S100A12/PREX1/AGER/CAMP/CST7/LRRK2/TNF/LILRA2/IFNG/CLEC4D/CX3CR1 |
| GO:0015732 | prostaglandin transport | 6/449 | 19/18800 | 3.74E-06 | 0.000301 | IL1A/P2RX7/SLCO2A1/IL1B/NOS2/EDN1 |
| GO:0032635 | interleukin-6 production | 16/449 | 172/18800 | 3.88E-06 | 0.000301 | LPL/IL1A/P2RX7/IL17D/IL1B/TLR8/NLRP12/NOS2/AGER/LILRB2/TNF/LILRA2/PTPN22/LILRA5/IFNG/ORM1 |
| GO:0032675 | regulation of interleukin-6 production | 16/449 | 172/18800 | 3.88E-06 | 0.000301 | LPL/IL1A/P2RX7/IL17D/IL1B/TLR8/NLRP12/NOS2/AGER/LILRB2/TNF/LILRA2/PTPN22/LILRA5/IFNG/ORM1 |
| GO:0060284 | regulation of cell development | 30/449 | 500/18800 | 3.99E-06 | 0.000304 | SEMA5A/TRPV2/EDNRB/LRP4/PLXNB3/HEYL/CLDN18/SPRY4/CDKN2B/DLL4/HAP1/WNT7A/UNC13D/IL1A/IL1B/CLDN5/SEMA6A/PREX1/CDH5/TRIM58/TNR/SEMA5B/GPER1/LRRK2/ACE/TNF/SEMA3G/IFNG/CX3CR1/ZNF488 |
| GO:0097530 | granulocyte migration | 15/449 | 154/18800 | 4.41E-06 | 0.000323 | TREM1/ITGA1/CCL4/IL1A/IL1B/CXCR2/MCOLN2/S100A12/PREX1/BST1/S100A8/EDN1/PLA2G1B/CXCR1/CD300H |
| GO:0023061 | signal release | 28/449 | 451/18800 | 4.43E-06 | 0.000323 | STX11/EDNRB/RIMS4/FFAR4/TBX3/LIN7A/KLF7/WNT7A/AGTR1/IL1A/ADCY8/P2RX7/IL1B/NOS2/BMP6/P2RY1/FFAR2/ILDR2/RPH3A/EDN1/SLC6A4/SYN2/GPER1/LRRK2/TNF/GHRHR/OTOF/IFNG |
| GO:0008645 | hexose transmembrane transport | 13/449 | 117/18800 | 4.49E-06 | 0.000323 | EDNRA/ENPP1/FFAR4/SLC2A6/SLC2A3/IL1B/EDN1/SLC5A4/SLC5A1/ACE/TNF/PLA2G1B/SLC2A14 |
| GO:0031341 | regulation of cell killing | 12/449 | 100/18800 | 4.67E-06 | 0.000323 | CADM1/CD5L/KLRD1/IL7R/P2RX7/NOS2/PRF1/AGER/ICAM1/ARG1/IFNG/CX3CR1 |
| GO:0006968 | cellular defense response | 9/449 | 54/18800 | 4.78E-06 | 0.000323 | CD5L/SPN/CXCR2/PRF1/GNLY/MNDA/LILRB2/CX3CR1/NCF1 |
| GO:0061900 | glial cell activation | 9/449 | 54/18800 | 4.78E-06 | 0.000323 | FPR2/C1QA/IL1B/AGER/CST7/LRRK2/TNF/IFNG/CX3CR1 |
| GO:0071715 | icosanoid transport | 9/449 | 54/18800 | 4.78E-06 | 0.000323 | PLA2G4F/IL1A/P2RX7/SLCO2A1/IL1B/NOS2/EDN1/ACE/PLA2G1B |
| GO:0015749 | monosaccharide transmembrane transport | 13/449 | 119/18800 | 5.43E-06 | 0.00036 | EDNRA/ENPP1/FFAR4/SLC2A6/SLC2A3/IL1B/EDN1/SLC5A4/SLC5A1/ACE/TNF/PLA2G1B/SLC2A14 |
| GO:0014002 | astrocyte development | 8/449 | 43/18800 | 6.92E-06 | 0.000452 | LAMC3/FPR2/C1QA/IL1B/AGER/S100A8/TNF/IFNG |
| GO:0002699 | positive regulation of immune effector process | 19/449 | 248/18800 | 8.69E-06 | 0.000559 | CADM1/COLEC10/PGC/KLRD1/UNC13D/FOXF1/FGR/TBX21/P2RX7/IL1B/NOS2/SH2D1B/IL18R1/FFAR2/FCN3/TNF/PTPN22/ARG1/IFNG |
| GO:0150076 | neuroinflammatory response | 10/449 | 73/18800 | 8.84E-06 | 0.00056 | FPR2/C1QA/ADCY8/IL1B/AGER/CST7/LRRK2/TNF/IFNG/CX3CR1 |
| GO:0098801 | regulation of renal system process | 7/449 | 33/18800 | 1.06E-05 | 0.000658 | EDNRB/EMP2/AGTR1/GJA5/NPR1/EDN1/ACE |
| GO:0032309 | icosanoid secretion | 8/449 | 46/18800 | 1.17E-05 | 0.000718 | PLA2G4F/IL1A/P2RX7/IL1B/NOS2/EDN1/ACE/PLA2G1B |
| GO:0033674 | positive regulation of kinase activity | 28/449 | 476/18800 | 1.21E-05 | 0.000722 | GPRC5D/FLT4/FPR2/TAL1/NRG3/EMP2/SLC11A1/FGR/ADCY8/P2RX7/IL1B/SASH1/S100A12/ADRB2/AGER/ANGPT4/PRLR/EDN1/LRRK2/ACE/TNF/PLA2G1B/TEK/LTK/LILRA5/IFNG/HBEGF/NCF1 |
| GO:0071621 | granulocyte chemotaxis | 13/449 | 128/18800 | 1.21E-05 | 0.000722 | TREM1/ITGA1/CCL4/IL1B/CXCR2/S100A12/PREX1/BST1/S100A8/EDN1/PLA2G1B/CXCR1/CD300H |
| GO:0007171 | activation of transmembrane receptor protein tyrosine kinase activity | 5/449 | 14/18800 | 1.27E-05 | 0.000748 | TAL1/NRG3/ADRB2/ANGPT4/PRLR |
| GO:0001774 | microglial cell activation | 8/449 | 47/18800 | 1.38E-05 | 0.000789 | FPR2/C1QA/AGER/CST7/LRRK2/TNF/IFNG/CX3CR1 |
| GO:0045601 | regulation of endothelial cell differentiation | 8/449 | 47/18800 | 1.38E-05 | 0.000789 | NOTCH4/IL1B/ACVRL1/CLDN5/BMP6/CDH5/TMEM100/TNF |
| GO:0003015 | heart process | 19/449 | 257/18800 | 1.44E-05 | 0.000812 | APLN/EDNRA/EDNRB/TBX5/EPAS1/TTN/GJA5/RAMP3/ADRA1D/NOS1/TNNC1/EDN1/DSC2/MYBPC3/ACE/TNF/TCAP/HBEGF/MYL7 |
| GO:0032102 | negative regulation of response to external stimulus | 26/449 | 429/18800 | 1.48E-05 | 0.000824 | UBASH3B/SEMA5A/FPR2/FFAR4/KLRD1/FOXF1/MEFV/FGR/NLRP12/SEMA6A/MYOZ1/CDH5/TNR/SEMA5B/EDN1/CST7/THBD/GPER1/TNF/LILRA2/TEK/ARG1/CALCRL/SEMA3G/SERPINE1/CPB2 |
| GO:0032757 | positive regulation of interleukin-8 production | 9/449 | 62/18800 | 1.54E-05 | 0.000843 | IL17D/IL1B/TLR8/NOS2/FFAR2/TNF/LILRA2/PLA2G1B/SERPINE1 |
| GO:0034219 | carbohydrate transmembrane transport | 13/449 | 131/18800 | 1.56E-05 | 0.000843 | EDNRA/ENPP1/FFAR4/SLC2A6/SLC2A3/IL1B/EDN1/SLC5A4/SLC5A1/ACE/TNF/PLA2G1B/SLC2A14 |
| GO:0030595 | leukocyte chemotaxis | 18/449 | 236/18800 | 1.59E-05 | 0.000848 | EDNRB/TREM1/FPR2/ITGA1/CCL4/IL1B/CXCR2/S100A12/PREX1/BST1/FFAR2/S100A8/EDN1/PLA2G1B/CXCR1/SERPINE1/CD300H/CX3CR1 |
| GO:0061028 | establishment of endothelial barrier | 8/449 | 48/18800 | 1.62E-05 | 0.000856 | EDNRA/EDNRB/IL1B/CLDN5/ROBO4/CDH5/TNF/ICAM1 |
| GO:0048143 | astrocyte activation | 6/449 | 24/18800 | 1.68E-05 | 0.000873 | FPR2/C1QA/IL1B/AGER/TNF/IFNG |
| GO:0021782 | glial cell development | 12/449 | 115/18800 | 1.99E-05 | 0.001006 | MYRF/LAMC3/FPR2/WASF3/C1QA/IL1B/ARHGEF10/AGER/S100A8/TNF/IFNG/ZNF488 |
| GO:0070371 | ERK1 and ERK2 cascade | 22/449 | 335/18800 | 2.00E-05 | 0.001006 | FLT4/BMPER/FPR2/FFAR4/SPRY4/CCL4/NDRG4/IL1A/RAMP3/IL1B/NLRP12/SEMA6A/P2RY1/AGER/EDN1/GPER1/TNF/GPBAR1/ICAM1/DUSP6/TEK/PTPN22 |
| GO:0016073 | snRNA metabolic process | 9/449 | 64/18800 | 2.01E-05 | 0.001006 | CT45A8/CT45A7/CT45A2/CT45A3/CT45A9/CT45A6/CT45A5/CT45A1/CT45A10 |
| GO:0070372 | regulation of ERK1 and ERK2 cascade | 21/449 | 311/18800 | 2.04E-05 | 0.001012 | FLT4/BMPER/FPR2/FFAR4/SPRY4/CCL4/NDRG4/IL1A/RAMP3/IL1B/NLRP12/SEMA6A/P2RY1/AGER/GPER1/TNF/GPBAR1/ICAM1/DUSP6/TEK/PTPN22 |
| GO:0032602 | chemokine production | 11/449 | 98/18800 | 2.21E-05 | 0.001072 | LPL/IL1RL1/TREM1/MEFV/IL1B/MCOLN2/ALOX15B/AGER/FFAR2/TNF/IFNG |
| GO:0002269 | leukocyte activation involved in inflammatory response | 8/449 | 50/18800 | 2.21E-05 | 0.001072 | FPR2/C1QA/AGER/CST7/LRRK2/TNF/IFNG/CX3CR1 |
| GO:0008643 | carbohydrate transport | 14/449 | 156/18800 | 2.33E-05 | 0.001114 | EDNRA/ENPP1/FFAR4/SLC2A6/SLC2A3/MFSD2A/IL1B/EDN1/SLC5A4/SLC5A1/ACE/TNF/PLA2G1B/SLC2A14 |
| GO:0042742 | defense response to bacterium | 23/449 | 364/18800 | 2.40E-05 | 0.001136 | TREM1/FPR2/SPN/PGC/IL7R/NLRC4/SLC11A1/FGR/GBP4/P2RX7/NOS2/S100A12/GNLY/CFP/S100A8/CAMP/CLEC4E/TNF/PLA2G1B/SERPINE1/GBP7/CLEC4D/HP |
| GO:0002449 | lymphocyte mediated immunity | 23/449 | 365/18800 | 2.51E-05 | 0.001173 | CADM1/KLRD1/IL7R/C1QA/EMP2/UNC13D/TBX21/P2RX7/IL1B/MYO1G/FCGR3A/TLR8/CSF2RB/SH2D1B/PRF1/AGER/IL18R1/GZMB/TNF/ICAM1/ARG1/GZMM/NKG7 |
| GO:0031649 | heat generation | 5/449 | 16/18800 | 2.67E-05 | 0.001234 | EDNRB/IL1A/IL1B/ADRB2/TNF |
| GO:0031644 | regulation of nervous system process | 13/449 | 138/18800 | 2.72E-05 | 0.001244 | MYRF/EDNRB/WASF3/WNT7A/IL1A/ADRB2/TMEM100/TNR/EDN1/CST7/LRRK2/TNF/ZNF488 |
| GO:0051928 | positive regulation of calcium ion transport | 12/449 | 119/18800 | 2.81E-05 | 0.001268 | TRPV2/TRPC3/CCL4/HAP1/P2RX7/RAMP3/STAC/F2RL3/GPER1/LILRA2/PLA2G1B/LILRA5 |
| GO:0002833 | positive regulation of response to biotic stimulus | 15/449 | 180/18800 | 2.89E-05 | 0.00129 | CADM1/FPR2/OASL/PGC/KLRD1/NLRC4/SASH1/TLR8/BMP6/SH2D1B/FFAR2/MNDA/LILRA2/TXK/ARG1 |
| GO:0060047 | heart contraction | 18/449 | 247/18800 | 2.92E-05 | 0.00129 | APLN/EDNRA/EDNRB/TBX5/EPAS1/TTN/GJA5/ADRA1D/NOS1/TNNC1/EDN1/DSC2/MYBPC3/ACE/TNF/TCAP/HBEGF/MYL7 |
| GO:0019722 | calcium-mediated signaling | 16/449 | 202/18800 | 2.95E-05 | 0.00129 | EDNRB/SLC24A4/FPR2/CCL4/AGTR1/P2RX7/CXCR2/MYOZ1/SAMD14/BST1/TMEM100/EDN1/LRRK2/TNF/CXCR1/CX3CR1 |
| GO:0006816 | calcium ion transport | 25/449 | 424/18800 | 3.41E-05 | 0.00147 | UBASH3B/EDNRA/TRPV2/EDNRB/MCOLN3/SLC24A4/TRPC3/GPM6A/CCL4/HAP1/P2RX7/RAMP3/MCOLN2/NOS1/STC2/STAC/F2RL3/EDN1/LILRB2/GPER1/ACE/LILRA2/PLA2G1B/LILRA5/CALCRL |
| GO:0008217 | regulation of blood pressure | 15/449 | 183/18800 | 3.50E-05 | 0.00147 | APLN/EDNRA/EDNRB/EMP2/AGTR1/GJA5/ACVRL1/NPR1/ADRA1D/NOS2/NOS1/ADRB2/EDN1/ACE/TNF |
| GO:0045766 | positive regulation of angiogenesis | 15/449 | 183/18800 | 3.50E-05 | 0.00147 | SEMA5A/BMPER/IL1A/IL1B/ACVRL1/SASH1/JCAD/HYAL1/FUT1/CDH5/ANGPT4/CAMP/TEK/SERPINE1/CX3CR1 |
| GO:1904018 | positive regulation of vasculature development | 15/449 | 183/18800 | 3.50E-05 | 0.00147 | SEMA5A/BMPER/IL1A/IL1B/ACVRL1/SASH1/JCAD/HYAL1/FUT1/CDH5/ANGPT4/CAMP/TEK/SERPINE1/CX3CR1 |
| GO:0032310 | prostaglandin secretion | 5/449 | 17/18800 | 3.71E-05 | 0.001525 | IL1A/P2RX7/IL1B/NOS2/EDN1 |
| GO:0038065 | collagen-activated signaling pathway | 5/449 | 17/18800 | 3.71E-05 | 0.001525 | UBASH3B/COL4A3/COL4A2/OSCAR/COL4A1 |
| GO:0051047 | positive regulation of secretion | 20/449 | 300/18800 | 3.84E-05 | 0.001564 | EDNRB/FFAR4/UNC13D/TTN/FGR/IL1A/ADCY8/P2RX7/IL1B/NPR1/BMP6/P2RY1/S100A8/EDN1/SLC6A4/GPER1/PLA2G1B/GHRHR/CPB2/IFNG |
| GO:0032722 | positive regulation of chemokine production | 9/449 | 70/18800 | 4.17E-05 | 0.001683 | LPL/IL1RL1/IL1B/MCOLN2/ALOX15B/AGER/FFAR2/TNF/IFNG |
| GO:0019221 | cytokine-mediated signaling pathway | 27/449 | 486/18800 | 4.68E-05 | 0.001851 | IL1RL1/OASL/CLDN18/IL7R/CCL4/IL1A/IL1B/CXCR2/CSF2RB/LILRA6/LILRA1/IL18R1/PRLR/EDN1/LILRB2/IL18RAP/TNF/LILRA2/GPR17/CXCR1/TXK/LILRA5/ARG1/IFNG/IL1R2/CX3CR1/LILRB3 |
| GO:0050805 | negative regulation of synaptic transmission | 9/449 | 71/18800 | 4.68E-05 | 0.001851 | GRIA1/ARC/ADCY8/IL1B/AGER/TNR/LILRB2/SLC6A4/LRRK2 |
| GO:0090066 | regulation of anatomical structure size | 27/449 | 487/18800 | 4.84E-05 | 0.001898 | SEMA5A/EDNRA/TRPV2/EDNRB/ITGA1/IL7R/WNT7A/AGTR1/GJA5/P2RX7/NPR1/ADRA1D/SEMA6A/NOS1/PLEKHH2/ADRB2/TMSB15A/P2RY1/TNR/SEMA5B/EDN1/SLC6A4/GPER1/LRRK2/ACE/TNF/SEMA3G |
| GO:0003206 | cardiac chamber morphogenesis | 12/449 | 126/18800 | 4.95E-05 | 0.001904 | EDNRA/HEYL/TBX5/TBX3/HEG1/DLL4/FOXF1/SMAD6/TGFBR3/TNNC1/MYBPC3/TEK |
| GO:0003231 | cardiac ventricle development | 12/449 | 126/18800 | 4.95E-05 | 0.001904 | EDNRA/HEYL/TBX5/TBX3/HEG1/DLL4/FOXF1/SMAD6/GJA5/TGFBR3/TNNC1/MYBPC3 |
| GO:0003229 | ventricular cardiac muscle tissue development | 8/449 | 56/18800 | 5.17E-05 | 0.001969 | EDNRA/TBX5/TBX3/HEG1/DLL4/TGFBR3/TNNC1/MYBPC3 |
| GO:0003208 | cardiac ventricle morphogenesis | 9/449 | 72/18800 | 5.24E-05 | 0.001977 | EDNRA/HEYL/TBX5/HEG1/DLL4/FOXF1/TGFBR3/TNNC1/MYBPC3 |
| GO:0007156 | homophilic cell adhesion via plasma membrane adhesion molecules | 14/449 | 168/18800 | 5.30E-05 | 0.001983 | CDH19/CADM1/PLXNB3/CNTN6/ROBO4/PCDHA4/CDH5/DSC2/PCDH12/PCDHA10/CDH16/PCDHGB3/PCDHGA11/PCDHGB4 |
| GO:0002703 | regulation of leukocyte mediated immunity | 17/449 | 236/18800 | 5.60E-05 | 0.002075 | CADM1/KLRD1/IL7R/UNC13D/FOXF1/FGR/TBX21/P2RX7/IL1B/NOS2/SH2D1B/AGER/IL18R1/TNF/ICAM1/ARG1/CX3CR1 |
| GO:0071526 | semaphorin-plexin signaling pathway | 7/449 | 43/18800 | 6.48E-05 | 0.002358 | SEMA5A/EDNRA/PLXNB3/SEMA6A/SEMA5B/EDN1/SEMA3G |
| GO:0140353 | lipid export from cell | 7/449 | 43/18800 | 6.48E-05 | 0.002358 | AGTR1/IL1A/P2RX7/IL1B/NOS2/BMP6/EDN1 |
| GO:0001935 | endothelial cell proliferation | 15/449 | 194/18800 | 6.86E-05 | 0.002474 | SEMA5A/APLN/FLT4/BMPER/COL4A3/PLXNB3/DLL4/AGTR1/ACVRL1/BMP6/JCAD/FUT1/TNF/TEK/ARG1 |
| GO:0010876 | lipid localization | 25/449 | 446/18800 | 7.72E-05 | 0.002762 | LPL/PITPNM2/PLA2G4F/ENPP1/STARD4/FABP4/APOBR/AGTR1/MFSD2A/IL1A/P2RX7/SLCO2A1/IL1B/MSR1/NOS2/BMP6/ABCA3/FFAR2/RBP2/SFTPA1/EDN1/ACE/TNF/PLA2G1B/ENPP7 |
| GO:0070374 | positive regulation of ERK1 and ERK2 cascade | 16/449 | 220/18800 | 8.23E-05 | 0.002917 | FLT4/BMPER/FPR2/FFAR4/CCL4/NDRG4/IL1A/RAMP3/P2RY1/AGER/GPER1/TNF/GPBAR1/ICAM1/TEK/PTPN22 |
| GO:0002526 | acute inflammatory response | 11/449 | 113/18800 | 8.32E-05 | 0.002925 | EDNRB/TREM1/IL1A/IL1B/FCGR3A/ALOX5AP/FFAR2/S100A8/TNF/ORM1/HP |
| GO:0055008 | cardiac muscle tissue morphogenesis | 8/449 | 60/18800 | 8.57E-05 | 0.002987 | EDNRA/HEG1/DLL4/TTN/TGFBR3/TNNC1/MYBPC3/TCAP |
| GO:0035813 | regulation of renal sodium excretion | 5/449 | 20/18800 | 8.76E-05 | 0.003005 | EDNRB/AGTR1/NPR1/EDN1/ACE |
| GO:0045860 | positive regulation of protein kinase activity | 23/449 | 396/18800 | 8.77E-05 | 0.003005 | GPRC5D/TAL1/NRG3/EMP2/SLC11A1/ADCY8/P2RX7/IL1B/SASH1/S100A12/ADRB2/AGER/ANGPT4/PRLR/EDN1/LRRK2/ACE/TNF/PLA2G1B/LILRA5/IFNG/HBEGF/NCF1 |
| GO:0002460 | adaptive immune response based on somatic recombination of immune receptors built from immunoglobulin superfamily domains | 22/449 | 370/18800 | 8.86E-05 | 0.003011 | IL1RL1/SPN/KLRD1/IL7R/C1QA/EMP2/UNC13D/SLC11A1/TBX21/P2RX7/IL1B/MYO1G/FCGR3A/TLR8/CSF2RB/PRF1/AGER/IL18R1/TNF/ICAM1/ARG1/GZMM |
| GO:0007189 | adenylate cyclase-activating G protein-coupled receptor signaling pathway | 13/449 | 155/18800 | 9.13E-05 | 0.003077 | ADGRE1/ADGRL2/ADCY8/ADGRE3/RAMP3/ADRA1D/ADRB2/ADGRG2/S1PR5/GPER1/GPR4/GHRHR/CALCRL |
| GO:0001660 | fever generation | 4/449 | 11/18800 | 9.27E-05 | 0.003096 | EDNRB/IL1A/IL1B/TNF |
| GO:0001659 | temperature homeostasis | 14/449 | 177/18800 | 9.33E-05 | 0.003096 | TRPV2/EDNRB/ACADL/FFAR4/FABP4/EPAS1/IL1A/IL1B/ADRB2/IL18R1/SCD/PRLR/TNF/ALPL |
| GO:0001936 | regulation of endothelial cell proliferation | 14/449 | 178/18800 | 9.91E-05 | 0.003262 | SEMA5A/APLN/FLT4/COL4A3/PLXNB3/DLL4/AGTR1/ACVRL1/BMP6/JCAD/FUT1/TNF/TEK/ARG1 |
| GO:0032642 | regulation of chemokine production | 10/449 | 97/18800 | 0.000108 | 0.003512 | LPL/IL1RL1/MEFV/IL1B/MCOLN2/ALOX15B/AGER/FFAR2/TNF/IFNG |
| GO:0048708 | astrocyte differentiation | 9/449 | 79/18800 | 0.000109 | 0.003541 | LAMC3/FPR2/TAL1/C1QA/IL1B/AGER/S100A8/TNF/IFNG |
| GO:0015718 | monocarboxylic acid transport | 11/449 | 117/18800 | 0.000114 | 0.003638 | PLA2G4F/IL1A/P2RX7/SLCO2A1/IL1B/NOS2/EDN1/ACE/TNF/PLA2G1B/SLC6A12 |
| GO:0002064 | epithelial cell development | 15/449 | 203/18800 | 0.000114 | 0.003638 | EDNRA/EDNRB/NOTCH4/HEG1/WNT7A/IL1A/IL1B/CLDN5/ROBO4/BMP6/CDH5/TNF/GPR4/ICAM1/COL22A1 |
| GO:0045940 | positive regulation of steroid metabolic process | 6/449 | 33/18800 | 0.000115 | 0.003638 | STARD4/AGTR1/IL1A/BMP6/TNF/IFNG |
| GO:0003197 | endocardial cushion development | 7/449 | 47/18800 | 0.000116 | 0.003657 | HEYL/TBX5/TBX3/FOXF1/ACVRL1/TMEM100/MSX1 |
| GO:0010721 | negative regulation of cell development | 14/449 | 181/18800 | 0.000118 | 0.003691 | SEMA5A/EDNRB/LRP4/CLDN18/SPRY4/CDKN2B/WNT7A/IL1A/IL1B/SEMA6A/TNR/SEMA5B/TNF/SEMA3G |
| GO:0051924 | regulation of calcium ion transport | 17/449 | 251/18800 | 0.000119 | 0.003693 | UBASH3B/TRPV2/TRPC3/CCL4/HAP1/P2RX7/RAMP3/NOS1/STC2/STAC/F2RL3/LILRB2/GPER1/ACE/LILRA2/PLA2G1B/LILRA5 |
| GO:0015908 | fatty acid transport | 10/449 | 99/18800 | 0.000128 | 0.003921 | PLA2G4F/FABP4/MFSD2A/IL1A/P2RX7/IL1B/RBP2/EDN1/ACE/PLA2G1B |
| GO:0042311 | vasodilation | 7/449 | 48/18800 | 0.000134 | 0.00407 | EDNRB/ITGA1/GJA5/NOS1/ADRB2/GPER1/TNF |
| GO:0007159 | leukocyte cell-cell adhesion | 22/449 | 381/18800 | 0.000135 | 0.004087 | SIRPB1/PAG1/SPN/SLC39A8/IL7R/SELPLG/IL1A/TBX21/IL1B/OLR1/AGER/S100A8/ILDR2/LRRC32/LILRB2/TNF/ICAM1/PTPN22/ARG1/IFNG/CX3CR1/CLEC4M |
| GO:0038063 | collagen-activated tyrosine kinase receptor signaling pathway | 4/449 | 12/18800 | 0.000136 | 0.004095 | UBASH3B/COL4A3/COL4A2/COL4A1 |
| GO:0034341 | response to interferon-gamma | 12/449 | 140/18800 | 0.000137 | 0.004095 | CCL4/SLC11A1/MEFV/GBP4/NOS2/MRC1/EDN1/TNF/TXK/ARG1/IFNG/GBP7 |
| GO:0050678 | regulation of epithelial cell proliferation | 22/449 | 382/18800 | 0.00014 | 0.004125 | SEMA5A/APLN/EDNRB/FLT4/COL4A3/PLXNB3/CDKN2B/DLL4/WNT7A/AGTR1/ACVRL1/TGFBR3/BMP6/JCAD/HYAL1/FUT1/ODAM/TNF/GPBAR1/TEK/ARG1/CPB2 |
| GO:0098742 | cell-cell adhesion via plasma-membrane adhesion molecules | 18/449 | 279/18800 | 0.00014 | 0.004125 | CDH19/CADM1/PLXNB3/CLDN18/CNTN6/CLDN5/ROBO4/PCDHA4/CDH5/SLITRK2/DSC2/PCDH12/ICAM1/PCDHA10/CDH16/PCDHGB3/PCDHGA11/PCDHGB4 |
| GO:0035812 | renal sodium excretion | 5/449 | 22/18800 | 0.000143 | 0.004171 | EDNRB/AGTR1/NPR1/EDN1/ACE |
| GO:0007162 | negative regulation of cell adhesion | 19/449 | 305/18800 | 0.000146 | 0.004225 | UBASH3B/SEMA5A/PLXNB3/NOTCH4/PAG1/SPN/SPRY4/TBX21/ACVRL1/SEMA6A/BMP6/ILDR2/TNR/LRRC32/LILRB2/PTPN22/ARG1/FAM107A/SERPINE1 |
| GO:0031343 | positive regulation of cell killing | 8/449 | 65/18800 | 0.000152 | 0.004352 | CADM1/CD5L/KLRD1/P2RX7/NOS2/PRF1/ARG1/IFNG |
| GO:0072577 | endothelial cell apoptotic process | 8/449 | 65/18800 | 0.000152 | 0.004352 | SEMA5A/COL4A3/CDH5/GPER1/TNF/ICAM1/TEK/SERPINE1 |
| GO:0051960 | regulation of nervous system development | 24/449 | 440/18800 | 0.000162 | 0.004559 | MYRF/SEMA5A/TRPV2/LRP4/PLXNB3/HEYL/CDKN2B/DLL4/HAP1/WASF3/WNT7A/IL1B/SEMA6A/TNR/SLITRK2/SEMA5B/CST7/GPER1/ACE/TNF/SEMA3G/IFNG/CX3CR1/ZNF488 |
| GO:0010737 | protein kinase A signaling | 6/449 | 35/18800 | 0.000162 | 0.004559 | EDNRA/TTN/RAMP3/ADRB2/EDN1/LRRK2 |
| GO:0032677 | regulation of interleukin-8 production | 10/449 | 102/18800 | 0.000164 | 0.004587 | IL17D/IL1B/TLR8/NOS2/FFAR2/TNF/LILRA2/PLA2G1B/PTPN22/SERPINE1 |
| GO:0015711 | organic anion transport | 21/449 | 361/18800 | 0.000173 | 0.004795 | SLC25A18/PLA2G4F/SLC39A8/SLC2A6/SLC22A10/SLC2A3/MFSD2A/IL1A/P2RX7/SLCO2A1/IL1B/NOS2/EDN1/ACE/TNF/FOLR3/SLCO4A1/PLA2G1B/CA4/SLC6A12/SLC2A14 |
| GO:0001913 | T cell mediated cytotoxicity | 7/449 | 50/18800 | 0.000174 | 0.004801 | KLRD1/IL7R/EMP2/P2RX7/PRF1/AGER/GZMM |
| GO:0050679 | positive regulation of epithelial cell proliferation | 15/449 | 211/18800 | 0.000175 | 0.004803 | SEMA5A/APLN/FLT4/PLXNB3/WNT7A/AGTR1/ACVRL1/BMP6/JCAD/HYAL1/ODAM/TNF/GPBAR1/TEK/ARG1 |
| GO:0032637 | interleukin-8 production | 10/449 | 103/18800 | 0.000178 | 0.004816 | IL17D/IL1B/TLR8/NOS2/FFAR2/TNF/LILRA2/PLA2G1B/PTPN22/SERPINE1 |
| GO:0050673 | epithelial cell proliferation | 24/449 | 443/18800 | 0.000179 | 0.004816 | SEMA5A/APLN/EDNRB/SDR16C5/FLT4/BMPER/COL4A3/PLXNB3/CDKN2B/DLL4/WNT7A/AGTR1/ACVRL1/TGFBR3/BMP6/JCAD/HYAL1/FUT1/ODAM/TNF/GPBAR1/TEK/ARG1/CPB2 |
| GO:0010893 | positive regulation of steroid biosynthetic process | 5/449 | 23/18800 | 0.000179 | 0.004816 | STARD4/IL1A/BMP6/TNF/IFNG |
| GO:1901224 | positive regulation of NIK/NF-kappaB signaling | 8/449 | 67/18800 | 0.000189 | 0.005044 | RTKN2/IL1B/SASH1/NLRP12/AGER/IL18R1/EDN1/TNF |
| GO:0001910 | regulation of leukocyte mediated cytotoxicity | 9/449 | 85/18800 | 0.000193 | 0.005067 | CADM1/KLRD1/IL7R/P2RX7/NOS2/AGER/ICAM1/ARG1/CX3CR1 |
| GO:0003205 | cardiac chamber development | 13/449 | 167/18800 | 0.000193 | 0.005067 | EDNRA/HEYL/TBX5/TBX3/HEG1/DLL4/FOXF1/SMAD6/GJA5/TGFBR3/TNNC1/MYBPC3/TEK |
| GO:1900452 | regulation of long-term synaptic depression | 4/449 | 13/18800 | 0.000193 | 0.005067 | ARC/ADCY8/AGER/LILRB2 |
| GO:0062013 | positive regulation of small molecule metabolic process | 12/449 | 146/18800 | 0.000204 | 0.005313 | GPD1/STARD4/P2RX7/IL1B/NOS2/NOS1/BMP6/P2RY1/GPER1/TNF/IFNG/GUCA2A |
| GO:0044062 | regulation of excretion | 5/449 | 24/18800 | 0.000222 | 0.00574 | EDNRB/AGTR1/NPR1/EDN1/ACE |
| GO:0042116 | macrophage activation | 10/449 | 106/18800 | 0.000225 | 0.005776 | IL1RL1/FPR2/C1QA/SLC11A1/AGER/CST7/LRRK2/TNF/IFNG/CX3CR1 |
| GO:0050866 | negative regulation of cell activation | 15/449 | 216/18800 | 0.000226 | 0.005776 | UBASH3B/PAG1/SPN/FOXF1/FGR/TBX21/ILDR2/MNDA/LRRC32/LILRB2/CST7/THBD/GPER1/PTPN22/ARG1 |
| GO:0071222 | cellular response to lipopolysaccharide | 15/449 | 217/18800 | 0.000238 | 0.006035 | EDNRB/IL1A/IL1B/SASH1/NOS2/BMP6/MRC1/CAMP/LILRB2/TNF/LILRA2/PTPN22/ARG1/SERPINE1/CX3CR1 |
| GO:0060402 | calcium ion transport into cytosol | 13/449 | 171/18800 | 0.000244 | 0.006154 | UBASH3B/TRPV2/MCOLN3/SLC24A4/TRPC3/HAP1/P2RX7/RAMP3/MCOLN2/NOS1/F2RL3/GPER1/PLA2G1B |
| GO:0050832 | defense response to fungus | 7/449 | 53/18800 | 0.000252 | 0.00633 | S100A12/GNLY/S100A8/CLEC4E/ARG1/CLEC4D/CX3CR1 |
| GO:0032306 | regulation of prostaglandin secretion | 4/449 | 14/18800 | 0.000266 | 0.006583 | IL1A/P2RX7/IL1B/EDN1 |
| GO:0032308 | positive regulation of prostaglandin secretion | 4/449 | 14/18800 | 0.000266 | 0.006583 | IL1A/P2RX7/IL1B/EDN1 |
| GO:0048762 | mesenchymal cell differentiation | 16/449 | 244/18800 | 0.000271 | 0.006676 | RANBP3L/SEMA5A/EDNRA/EDNRB/NOTCH4/HEYL/TBX5/TBX3/IL1B/TGFBR3/SEMA6A/TMEM100/SEMA5B/EDN1/SEMA3G/MSX1 |
| GO:0050766 | positive regulation of phagocytosis | 8/449 | 71/18800 | 0.000284 | 0.006874 | SIRPB1/COLEC10/FPR2/SLC11A1/IL1B/FCN3/TNF/IFNG |
| GO:0045926 | negative regulation of growth | 16/449 | 245/18800 | 0.000284 | 0.006874 | SEMA5A/ENPP1/TBX5/ACVRL1/NPR1/SEMA6A/MYOZ1/ALOX15B/ADRB2/HYAL1/STC2/TNR/SEMA5B/SLC6A4/SEMA3G/MSX1 |
| GO:0010524 | positive regulation of calcium ion transport into cytosol | 7/449 | 54/18800 | 0.000284 | 0.006874 | TRPC3/HAP1/P2RX7/RAMP3/F2RL3/GPER1/PLA2G1B |
| GO:0071695 | anatomical structure maturation | 16/449 | 246/18800 | 0.000297 | 0.007105 | EDNRA/EDNRB/ADAMTS7/SLC24A4/TAL1/GLDN/EPAS1/C1QA/ACVRL1/CDH5/TRIM58/EDN1/LRRK2/PHOSPHO1/GHRHR/CX3CR1 |
| GO:0010959 | regulation of metal ion transport | 22/449 | 403/18800 | 0.000297 | 0.007105 | UBASH3B/TRPV2/TRPC3/CCL4/HAP1/HECW2/P2RX7/RAMP3/NOS1/ADRB2/STC2/STAC/F2RL3/LILRB2/GPER1/DPP6/ACE/LILRA2/PLA2G1B/LILRA5/KCNIP1/IFNG |
| GO:0045834 | positive regulation of lipid metabolic process | 12/449 | 153/18800 | 0.000315 | 0.007491 | STARD4/FPR2/AGTR1/MFSD2A/FGR/IL1A/IL1B/BMP6/TNF/TEK/IFNG/ENPP7 |
| GO:0071674 | mononuclear cell migration | 14/449 | 199/18800 | 0.000318 | 0.007515 | FPR2/SPN/CCL4/TBX21/MYO1G/CXCR2/NLRP12/S100A12/AGER/TNF/ICAM1/CXCR1/SERPINE1/CX3CR1 |
| GO:0001938 | positive regulation of endothelial cell proliferation | 10/449 | 111/18800 | 0.000328 | 0.007665 | SEMA5A/APLN/FLT4/PLXNB3/AGTR1/ACVRL1/BMP6/JCAD/TEK/ARG1 |
| GO:0048640 | negative regulation of developmental growth | 10/449 | 111/18800 | 0.000328 | 0.007665 | SEMA5A/TBX5/SEMA6A/MYOZ1/ADRB2/STC2/TNR/SEMA5B/SLC6A4/SEMA3G |
| GO:0042832 | defense response to protozoan | 5/449 | 26/18800 | 0.00033 | 0.007672 | SLC11A1/GBP4/ARG1/GBP7/NKG7 |
| GO:0001655 | urogenital system development | 20/449 | 352/18800 | 0.000332 | 0.00768 | EDNRA/COL4A4/EDNRB/LRP4/BMPER/COL4A3/HEYL/HPGD/AGTR1/FOXF1/SMAD6/ALOX15B/BMP6/PRLR/COL4A1/LRRK2/PSAPL1/ACE/GPR4/TEK |
| GO:1903532 | positive regulation of secretion by cell | 17/449 | 274/18800 | 0.000337 | 0.007751 | FFAR4/UNC13D/TTN/FGR/IL1A/ADCY8/P2RX7/IL1B/BMP6/P2RY1/EDN1/SLC6A4/GPER1/PLA2G1B/GHRHR/CPB2/IFNG |
| GO:0015833 | peptide transport | 16/449 | 249/18800 | 0.00034 | 0.007766 | CA2/FFAR4/DISP1/KLF7/ADCY8/IL1B/NOS2/FFAR2/S100A8/ILDR2/EDN1/GPER1/TNF/GHRHR/IFNG/CLEC4M |
| GO:0045123 | cellular extravasation | 8/449 | 73/18800 | 0.000344 | 0.007766 | SPN/ITGA1/SELPLG/BST1/AGER/TNF/ICAM1/CX3CR1 |
| GO:0060415 | muscle tissue morphogenesis | 8/449 | 73/18800 | 0.000344 | 0.007766 | EDNRA/HEG1/DLL4/TTN/TGFBR3/TNNC1/MYBPC3/TCAP |
| GO:0060485 | mesenchyme development | 18/449 | 301/18800 | 0.000356 | 0.007983 | RANBP3L/SEMA5A/EDNRA/EDNRB/NOTCH4/HEYL/TBX5/TBX3/FOXF1/IL1B/ACVRL1/TGFBR3/SEMA6A/TMEM100/SEMA5B/EDN1/SEMA3G/MSX1 |
| GO:0055078 | sodium ion homeostasis | 7/449 | 56/18800 | 0.000357 | 0.007983 | EDNRA/EDNRB/AGTR1/IL1A/NPR1/EDN1/ACE |
| GO:0030324 | lung development | 13/449 | 179/18800 | 0.00038 | 0.008455 | FLT4/TBX5/HEG1/EPAS1/PKDCC/FOXF1/HSD11B1/ABCA3/CRISPLD2/ACE/TNF/RSPO2/ARG1 |
| GO:0060541 | respiratory system development | 14/449 | 203/18800 | 0.000389 | 0.008601 | FLT4/TBX5/HEG1/DISP1/EPAS1/PKDCC/FOXF1/HSD11B1/ABCA3/CRISPLD2/ACE/TNF/RSPO2/ARG1 |
| GO:0010817 | regulation of hormone levels | 25/449 | 496/18800 | 0.000394 | 0.008601 | EDNRB/SDR16C5/FFAR4/TBX3/DISP1/KLF7/CYP3A5/AGTR1/ADCY8/IL1B/NOS2/BMP6/STC2/P2RY1/FFAR2/SULT1B1/ILDR2/EDN1/GPER1/AWAT2/ACE/TNF/SLCO4A1/GHRHR/IFNG |
| GO:0001562 | response to protozoan | 5/449 | 27/18800 | 0.000397 | 0.008601 | SLC11A1/GBP4/ARG1/GBP7/NKG7 |
| GO:0009065 | glutamine family amino acid catabolic process | 5/449 | 27/18800 | 0.000397 | 0.008601 | PRODH/ASRGL1/NOS2/NOS1/ARG1 |
| GO:0097254 | renal tubular secretion | 5/449 | 27/18800 | 0.000397 | 0.008601 | EDNRB/AGTR1/NPR1/EDN1/ACE |
| GO:1905954 | positive regulation of lipid localization | 9/449 | 94/18800 | 0.000413 | 0.008885 | LPL/IL1A/P2RX7/IL1B/MSR1/BMP6/ABCA3/EDN1/ENPP7 |
| GO:0071219 | cellular response to molecule of bacterial origin | 15/449 | 229/18800 | 0.000422 | 0.009047 | EDNRB/IL1A/IL1B/SASH1/NOS2/BMP6/MRC1/CAMP/LILRB2/TNF/LILRA2/PTPN22/ARG1/SERPINE1/CX3CR1 |
| GO:0050890 | cognition | 18/449 | 306/18800 | 0.000433 | 0.009223 | AFF2/SHROOM4/NDRG4/MFSD2A/GRIA1/ARC/ADCY8/CLDN5/LRRN4/AGER/MGAT3/TNR/LILRB2/SLC6A4/TNF/CHRM1/FAM107A/CX3CR1 |
| GO:0003073 | regulation of systemic arterial blood pressure | 9/449 | 95/18800 | 0.000446 | 0.009463 | EDNRB/EMP2/AGTR1/GJA5/ADRA1D/ADRB2/EDN1/ACE/TNF |
| GO:0030198 | extracellular matrix organization | 18/449 | 307/18800 | 0.00045 | 0.009488 | COL4A4/ADAMTS7/COL4A3/SLC39A8/COL4A2/COL12A1/TNXB/SPOCK2/FOXF1/ANGPTL7/CRISPLD2/MMP25/TNR/COL4A1/TNF/COL22A1/CPB2/MIA |
| GO:0050767 | regulation of neurogenesis | 20/449 | 361/18800 | 0.000459 | 0.009562 | SEMA5A/TRPV2/LRP4/PLXNB3/HEYL/CDKN2B/DLL4/HAP1/WNT7A/IL1B/SEMA6A/TNR/SEMA5B/GPER1/ACE/TNF/SEMA3G/IFNG/CX3CR1/ZNF488 |
| GO:0017014 | protein nitrosylation | 4/449 | 16/18800 | 0.000465 | 0.009562 | NOS2/NOS1/S100A8/ACE |
| GO:0018119 | peptidyl-cysteine S-nitrosylation | 4/449 | 16/18800 | 0.000465 | 0.009562 | NOS2/NOS1/S100A8/ACE |
| GO:0043062 | extracellular structure organization | 18/449 | 308/18800 | 0.000467 | 0.009562 | COL4A4/ADAMTS7/COL4A3/SLC39A8/COL4A2/COL12A1/TNXB/SPOCK2/FOXF1/ANGPTL7/CRISPLD2/MMP25/TNR/COL4A1/TNF/COL22A1/CPB2/MIA |
| GO:0030323 | respiratory tube development | 13/449 | 183/18800 | 0.00047 | 0.009562 | FLT4/TBX5/HEG1/EPAS1/PKDCC/FOXF1/HSD11B1/ABCA3/CRISPLD2/ACE/TNF/RSPO2/ARG1 |
| GO:0006836 | neurotransmitter transport | 14/449 | 207/18800 | 0.000473 | 0.009562 | STX11/RIMS4/LIN7A/WNT7A/P2RX7/NOS1/P2RY1/RPH3A/SLC6A4/SYN2/GPER1/LRRK2/OTOF/SLC6A12 |
| GO:0010955 | negative regulation of protein processing | 5/449 | 28/18800 | 0.000474 | 0.009562 | CST7/LRRK2/SERPINE1/CPB2/IL1R2 |
| GO:0035116 | embryonic hindlimb morphogenesis | 5/449 | 28/18800 | 0.000474 | 0.009562 | AFF3/TBX3/WNT7A/RSPO2/MSX1 |
| GO:1903318 | negative regulation of protein maturation | 5/449 | 28/18800 | 0.000474 | 0.009562 | CST7/LRRK2/SERPINE1/CPB2/IL1R2 |
| GO:0001755 | neural crest cell migration | 7/449 | 59/18800 | 0.000494 | 0.009864 | SEMA5A/EDNRA/EDNRB/SEMA6A/SEMA5B/EDN1/SEMA3G |
| GO:2000351 | regulation of endothelial cell apoptotic process | 7/449 | 59/18800 | 0.000494 | 0.009864 | SEMA5A/CDH5/GPER1/TNF/ICAM1/TEK/SERPINE1 |
| GO:0006869 | lipid transport | 21/449 | 391/18800 | 0.000502 | 0.009965 | PITPNM2/PLA2G4F/STARD4/FABP4/APOBR/AGTR1/MFSD2A/IL1A/P2RX7/SLCO2A1/IL1B/MSR1/NOS2/BMP6/ABCA3/RBP2/SFTPA1/EDN1/ACE/PLA2G1B/ENPP7 |
| GO:0045229 | external encapsulating structure organization | 18/449 | 310/18800 | 0.000505 | 0.009978 | COL4A4/ADAMTS7/COL4A3/SLC39A8/COL4A2/COL12A1/TNXB/SPOCK2/FOXF1/ANGPTL7/CRISPLD2/MMP25/TNR/COL4A1/TNF/COL22A1/CPB2/MIA |
| GO:0003009 | skeletal muscle contraction | 6/449 | 43/18800 | 0.000517 | 0.010182 | SYNM/TNNC1/STAC/TNF/TCAP/TNNT1 |
| GO:0048738 | cardiac muscle tissue development | 15/449 | 234/18800 | 0.00053 | 0.010372 | EDNRA/TBX5/TBX3/HEG1/DLL4/TTN/NDRG4/GJA5/TGFBR3/TNNC1/EDN1/MYBPC3/TCAP/MSX1/MYL7 |
| GO:0071346 | cellular response to interferon-gamma | 10/449 | 118/18800 | 0.000535 | 0.01038 | CCL4/GBP4/NOS2/MRC1/EDN1/TNF/TXK/ARG1/IFNG/GBP7 |
| GO:1904019 | epithelial cell apoptotic process | 10/449 | 118/18800 | 0.000535 | 0.01038 | SEMA5A/EDNRA/COL4A3/CDH5/GPER1/TNF/PLA2G1B/ICAM1/TEK/SERPINE1 |
| GO:0010827 | regulation of glucose transmembrane transport | 8/449 | 78/18800 | 0.00054 | 0.010436 | EDNRA/ENPP1/FFAR4/IL1B/EDN1/ACE/TNF/PLA2G1B |
| GO:0034767 | positive regulation of ion transmembrane transport | 12/449 | 163/18800 | 0.000559 | 0.010701 | EDNRA/HAP1/ARC/P2RX7/RAMP3/NOS1/ADRB2/STAC/F2RL3/EDN1/GPER1/IFNG |
| GO:0120162 | positive regulation of cold-induced thermogenesis | 9/449 | 98/18800 | 0.000561 | 0.010701 | TRPV2/ACADL/FFAR4/FABP4/EPAS1/ADRB2/SCD/PRLR/ALPL |
| GO:0002825 | regulation of T-helper 1 type immune response | 5/449 | 29/18800 | 0.000562 | 0.010701 | IL1RL1/SLC11A1/TBX21/IL1B/IL18R1 |
| GO:0042886 | amide transport | 17/449 | 287/18800 | 0.000573 | 0.010856 | CA2/FFAR4/DISP1/KLF7/ADCY8/IL1B/NOS2/FFAR2/S100A8/ILDR2/EDN1/GPER1/TNF/FOLR3/GHRHR/IFNG/CLEC4M |
| GO:0045981 | positive regulation of nucleotide metabolic process | 6/449 | 44/18800 | 0.000587 | 0.011007 | GPD1/P2RX7/NOS2/NOS1/IFNG/GUCA2A |
| GO:1900544 | positive regulation of purine nucleotide metabolic process | 6/449 | 44/18800 | 0.000587 | 0.011007 | GPD1/P2RX7/NOS2/NOS1/IFNG/GUCA2A |
| GO:0016264 | gap junction assembly | 4/449 | 17/18800 | 0.000597 | 0.011007 | TBX5/GJA5/IL1B/ACE |
| GO:0042481 | regulation of odontogenesis | 4/449 | 17/18800 | 0.000597 | 0.011007 | EDN1/SP6/RSPO2/MSX1 |
| GO:1901550 | regulation of endothelial cell development | 4/449 | 17/18800 | 0.000597 | 0.011007 | IL1B/CLDN5/CDH5/TNF |
| GO:1903140 | regulation of establishment of endothelial barrier | 4/449 | 17/18800 | 0.000597 | 0.011007 | IL1B/CLDN5/CDH5/TNF |
| GO:0050764 | regulation of phagocytosis | 9/449 | 99/18800 | 0.000605 | 0.011105 | SIRPB1/COLEC10/FPR2/SLC11A1/FGR/IL1B/FCN3/TNF/IFNG |
| GO:0002683 | negative regulation of immune system process | 22/449 | 425/18800 | 0.00061 | 0.011147 | UBASH3B/IL1RL1/PAG1/SPN/CLDN18/KLRD1/IL7R/FOXF1/FGR/TBX21/IL17D/ILDR2/MNDA/LRRC32/LILRB2/CST7/GPER1/TNF/PTPN22/ARG1/CX3CR1/LILRB3 |
| GO:0001505 | regulation of neurotransmitter levels | 14/449 | 213/18800 | 0.000629 | 0.011444 | STX11/RIMS4/LIN7A/WNT7A/P2RX7/NOS1/P2RY1/RPH3A/SLC6A4/SYN2/GPER1/LRRK2/OTOF/SLC6A12 |
| GO:0003151 | outflow tract morphogenesis | 8/449 | 80/18800 | 0.000641 | 0.011512 | EDNRA/HEYL/TBX3/SMAD6/GJA5/CLDN5/TGFBR3/EDN1 |
| GO:0048644 | muscle organ morphogenesis | 8/449 | 80/18800 | 0.000641 | 0.011512 | EDNRA/HEG1/DLL4/TTN/TGFBR3/TNNC1/MYBPC3/TCAP |
| GO:0009914 | hormone transport | 17/449 | 290/18800 | 0.000644 | 0.011512 | FFAR4/TBX3/KLF7/AGTR1/ADCY8/IL1B/NOS2/BMP6/P2RY1/FFAR2/ILDR2/EDN1/GPER1/TNF/SLCO4A1/GHRHR/IFNG |
| GO:0010632 | regulation of epithelial cell migration | 17/449 | 290/18800 | 0.000644 | 0.011512 | SEMA5A/FLT4/BMPER/DLL4/WNT7A/EMP2/ACVRL1/SASH1/JCAD/HYAL1/FUT1/ANGPT4/EDN1/TNF/TEK/IFNG/HBEGF |
| GO:0038094 | Fc-gamma receptor signaling pathway | 5/449 | 30/18800 | 0.000661 | 0.011622 | FGR/MYO1G/FCGR3A/CLEC4E/CLEC4D |
| GO:0060292 | long-term synaptic depression | 5/449 | 30/18800 | 0.000661 | 0.011622 | GRIA1/ARC/ADCY8/AGER/LILRB2 |
| GO:0042088 | T-helper 1 type immune response | 6/449 | 45/18800 | 0.000664 | 0.011622 | IL1RL1/SPN/SLC11A1/TBX21/IL1B/IL18R1 |
| GO:1900271 | regulation of long-term synaptic potentiation | 6/449 | 45/18800 | 0.000664 | 0.011622 | ARC/ADCY8/AGER/LILRB2/FAM107A/CX3CR1 |
| GO:2000273 | positive regulation of signaling receptor activity | 6/449 | 45/18800 | 0.000664 | 0.011622 | ARC/ADRB2/EDN1/IFNG/HBEGF/NCF1 |
| GO:0042063 | gliogenesis | 17/449 | 291/18800 | 0.000669 | 0.011624 | MYRF/LAMC3/FPR2/TAL1/CDKN2B/WASF3/C1QA/IL1B/ARHGEF10/P2RY1/AGER/S100A8/TNF/CHRM1/IFNG/CX3CR1/ZNF488 |
| GO:0060401 | cytosolic calcium ion transport | 13/449 | 190/18800 | 0.00067 | 0.011624 | UBASH3B/TRPV2/MCOLN3/SLC24A4/TRPC3/HAP1/P2RX7/RAMP3/MCOLN2/NOS1/F2RL3/GPER1/PLA2G1B |
| GO:0007269 | neurotransmitter secretion | 11/449 | 145/18800 | 0.000733 | 0.012621 | STX11/RIMS4/LIN7A/WNT7A/P2RX7/P2RY1/RPH3A/SYN2/GPER1/LRRK2/OTOF |
| GO:0099643 | signal release from synapse | 11/449 | 145/18800 | 0.000733 | 0.012621 | STX11/RIMS4/LIN7A/WNT7A/P2RX7/P2RY1/RPH3A/SYN2/GPER1/LRRK2/OTOF |
| GO:0001974 | blood vessel remodeling | 6/449 | 46/18800 | 0.000748 | 0.012691 | EDNRA/FLT4/DLL4/EPAS1/ACVRL1/ACE |
| GO:0031640 | killing of cells of another organism | 6/449 | 46/18800 | 0.000748 | 0.012691 | P2RX7/NOS2/S100A12/PRF1/GNLY/IFNG |
| GO:0002827 | positive regulation of T-helper 1 type immune response | 4/449 | 18/18800 | 0.000753 | 0.012691 | SLC11A1/TBX21/IL1B/IL18R1 |
| GO:0030730 | sequestering of triglyceride | 4/449 | 18/18800 | 0.000753 | 0.012691 | LPL/ENPP1/IL1B/TNF |
| GO:0031643 | positive regulation of myelination | 4/449 | 18/18800 | 0.000753 | 0.012691 | MYRF/WASF3/CST7/ZNF488 |
| GO:0042310 | vasoconstriction | 8/449 | 82/18800 | 0.000756 | 0.012696 | EDNRA/EDNRB/AGTR1/GJA5/ADRA1D/EDN1/SLC6A4/ACE |
| GO:0035115 | embryonic forelimb morphogenesis | 5/449 | 31/18800 | 0.000773 | 0.012909 | TBX5/TBX3/WNT7A/RSPO2/MSX1 |
| GO:0002695 | negative regulation of leukocyte activation | 13/449 | 193/18800 | 0.000775 | 0.012909 | PAG1/SPN/FOXF1/FGR/TBX21/ILDR2/MNDA/LRRC32/LILRB2/CST7/GPER1/PTPN22/ARG1 |
| GO:0010522 | regulation of calcium ion transport into cytosol | 9/449 | 103/18800 | 0.000807 | 0.013323 | UBASH3B/TRPC3/HAP1/P2RX7/RAMP3/NOS1/F2RL3/GPER1/PLA2G1B |
| GO:0032760 | positive regulation of tumor necrosis factor production | 9/449 | 103/18800 | 0.000807 | 0.013323 | LPL/SPN/IL1A/AGER/LRRK2/LILRA2/LILRA5/IFNG/ORM1 |
| GO:0009620 | response to fungus | 7/449 | 64/18800 | 0.000812 | 0.013364 | S100A12/GNLY/S100A8/CLEC4E/ARG1/CLEC4D/CX3CR1 |
| GO:0002831 | regulation of response to biotic stimulus | 19/449 | 351/18800 | 0.000834 | 0.013665 | CADM1/FPR2/OASL/PGC/KLRD1/NLRC4/FGR/IL1B/SASH1/TLR8/BMP6/SH2D1B/FFAR2/MNDA/LILRA2/PTPN22/TXK/ARG1/NCF1 |
| GO:0045776 | negative regulation of blood pressure | 6/449 | 47/18800 | 0.000841 | 0.013671 | APLN/GJA5/NOS2/NOS1/ADRB2/TNF |
| GO:0055010 | ventricular cardiac muscle tissue morphogenesis | 6/449 | 47/18800 | 0.000841 | 0.013671 | EDNRA/HEG1/DLL4/TGFBR3/TNNC1/MYBPC3 |
| GO:0002444 | myeloid leukocyte mediated immunity | 9/449 | 104/18800 | 0.000865 | 0.014002 | TREM1/UNC13D/FOXF1/FGR/FCGR3A/ACE/PLA2G1B/ARG1/CX3CR1 |
| GO:1904064 | positive regulation of cation transmembrane transport | 11/449 | 148/18800 | 0.000869 | 0.014018 | EDNRA/HAP1/ARC/RAMP3/NOS1/ADRB2/STAC/F2RL3/EDN1/GPER1/IFNG |
| GO:0051482 | positive regulation of cytosolic calcium ion concentration involved in phospholipase C-activating G protein-coupled signaling pathway | 5/449 | 32/18800 | 0.000898 | 0.014379 | AGTR1/F2RL3/EDN1/GPR4/GPR17 |
| GO:1901889 | negative regulation of cell junction assembly | 5/449 | 32/18800 | 0.000898 | 0.014379 | IL1B/ACVRL1/ACE/TNF/FAM107A |
| GO:0007043 | cell-cell junction assembly | 11/449 | 149/18800 | 0.000919 | 0.014646 | CDH19/TBX5/HEG1/CLDN18/GJA5/IL1B/CLDN5/CDH5/ACE/TNF/GPBAR1 |
| GO:0010744 | positive regulation of macrophage derived foam cell differentiation | 4/449 | 19/18800 | 0.000936 | 0.014676 | LPL/AGTR1/MSR1/ALOX15B |
| GO:0035743 | CD4-positive, alpha-beta T cell cytokine production | 4/449 | 19/18800 | 0.000936 | 0.014676 | TBX21/IL1B/IL18R1/ARG1 |
| GO:0003044 | regulation of systemic arterial blood pressure mediated by a chemical signal | 6/449 | 48/18800 | 0.000942 | 0.014676 | EDNRB/AGTR1/ADRA1D/ADRB2/EDN1/ACE |
| GO:0006953 | acute-phase response | 6/449 | 48/18800 | 0.000942 | 0.014676 | EDNRB/IL1A/IL1B/TNF/ORM1/HP |
| GO:0043114 | regulation of vascular permeability | 6/449 | 48/18800 | 0.000942 | 0.014676 | NPR1/CLDN5/BMP6/CDH5/GPR4/TEK |
| GO:0060976 | coronary vasculature development | 6/449 | 48/18800 | 0.000942 | 0.014676 | APLN/TBX5/SMAD6/TGFBR3/GPER1/ACE |
| GO:0010469 | regulation of signaling receptor activity | 12/449 | 173/18800 | 0.000947 | 0.014693 | SLC24A4/ARC/RAMP3/ADRB2/P2RY1/EDN1/TNF/DKK2/SERPINE1/IFNG/HBEGF/NCF1 |
| GO:0046889 | positive regulation of lipid biosynthetic process | 8/449 | 85/18800 | 0.00096 | 0.014809 | STARD4/MFSD2A/IL1A/IL1B/BMP6/TNF/IFNG/ENPP7 |
| GO:0014706 | striated muscle tissue development | 15/449 | 248/18800 | 0.000961 | 0.014809 | EDNRA/TBX5/TBX3/HEG1/DLL4/TTN/NDRG4/GJA5/TGFBR3/TNNC1/EDN1/MYBPC3/TCAP/MSX1/MYL7 |
| GO:0032835 | glomerulus development | 7/449 | 66/18800 | 0.000978 | 0.014948 | EDNRA/COL4A4/EDNRB/COL4A3/HEYL/GPR4/TEK |
| GO:0071677 | positive regulation of mononuclear cell migration | 7/449 | 66/18800 | 0.000978 | 0.014948 | FPR2/SPN/CCL4/AGER/TNF/SERPINE1/CX3CR1 |
| GO:0010634 | positive regulation of epithelial cell migration | 12/449 | 174/18800 | 0.000995 | 0.015107 | SEMA5A/FLT4/WNT7A/SASH1/JCAD/HYAL1/FUT1/ANGPT4/EDN1/TEK/IFNG/HBEGF |
| GO:0048771 | tissue remodeling | 12/449 | 174/18800 | 0.000995 | 0.015107 | UBASH3B/EDNRA/FLT4/CLDN18/DLL4/EPAS1/IL1A/P2RX7/ACVRL1/ADRB2/ACE/ARG1 |
| GO:0006887 | exocytosis | 19/449 | 357/18800 | 0.001019 | 0.015403 | STX11/RIMS4/SEPTIN4/LIN7A/HAP1/WNT7A/UNC13D/FOXF1/FGR/P2RX7/LGI3/MYO1G/P2RY1/STXBP6/SYN2/LRRK2/OTOF/IFNG/NKG7 |
| GO:0060317 | cardiac epithelial to mesenchymal transition | 5/449 | 33/18800 | 0.001038 | 0.015645 | HEYL/TBX3/TGFBR3/TMEM100/MSX1 |
| GO:0002253 | activation of immune response | 20/449 | 386/18800 | 0.001052 | 0.015792 | COLEC10/CD5L/FPR2/MASP1/KLRD1/NLRC4/C1QA/FGR/IL1B/MYO1G/FPR1/CFP/FFAR2/MNDA/FCN3/BTNL9/LILRA2/PTPN22/TXK/BTNL8 |
| GO:1903557 | positive regulation of tumor necrosis factor superfamily cytokine production | 9/449 | 107/18800 | 0.00106 | 0.015854 | LPL/SPN/IL1A/AGER/LRRK2/LILRA2/LILRA5/IFNG/ORM1 |
| GO:1901343 | negative regulation of vasculature development | 11/449 | 152/18800 | 0.001082 | 0.016122 | COL4A3/COL4A2/NPR1/CLDN5/SEMA6A/ANGPTL7/ANGPT4/TNF/GPR4/TEK/SERPINE1 |
| GO:0009743 | response to carbohydrate | 14/449 | 226/18800 | 0.001118 | 0.016608 | LPL/COL4A3/KLF7/IL1A/ADCY8/IL1B/AGER/ILDR2/GPER1/ACE/ICAM1/GHRHR/CALCRL/CPB2 |
| GO:0042476 | odontogenesis | 10/449 | 130/18800 | 0.001136 | 0.016809 | LRP4/SLC24A4/EDN1/SP6/ODAM/RSPO2/SOSTDC1/SERPINE1/MSX1/ALPL |
| GO:0006525 | arginine metabolic process | 4/449 | 20/18800 | 0.001148 | 0.016921 | SLC39A8/NOS2/NOS1/ARG1 |
| GO:0031281 | positive regulation of cyclase activity | 6/449 | 50/18800 | 0.001172 | 0.017214 | EDNRA/NOS2/NOS1/ADRB2/GHRHR/GUCA2A |
| GO:2000352 | negative regulation of endothelial cell apoptotic process | 5/449 | 34/18800 | 0.001194 | 0.017327 | SEMA5A/CDH5/ICAM1/TEK/SERPINE1 |
| GO:0051092 | positive regulation of NF-kappaB transcription factor activity | 11/449 | 154/18800 | 0.001203 | 0.017327 | RTKN2/NLRC4/IL1B/S100A12/AGER/IL18R1/S100A8/IL18RAP/TNF/PLA2G1B/CX3CR1 |
| GO:0097553 | calcium ion transmembrane import into cytosol | 11/449 | 154/18800 | 0.001203 | 0.017327 | UBASH3B/TRPV2/MCOLN3/SLC24A4/HAP1/P2RX7/RAMP3/MCOLN2/NOS1/F2RL3/GPER1 |
| GO:0002709 | regulation of T cell mediated immunity | 8/449 | 88/18800 | 0.001205 | 0.017327 | KLRD1/IL7R/TBX21/P2RX7/IL1B/AGER/IL18R1/ARG1 |
| GO:0014031 | mesenchymal cell development | 8/449 | 88/18800 | 0.001205 | 0.017327 | SEMA5A/EDNRA/EDNRB/HEYL/SEMA6A/SEMA5B/EDN1/SEMA3G |
| GO:0048864 | stem cell development | 8/449 | 88/18800 | 0.001205 | 0.017327 | SEMA5A/EDNRA/EDNRB/WNT7A/SEMA6A/SEMA5B/EDN1/SEMA3G |
| GO:0003012 | muscle system process | 22/449 | 449/18800 | 0.001246 | 0.017812 | EDNRA/EDNRB/TRPC3/TBX3/SYNM/TTN/GJA5/IL1B/MYOZ1/NOS1/TNNC1/ADRB2/P2RY1/STAC/EDN1/DSC2/GPER1/MYBPC3/TNF/TCAP/CALCRL/TNNT1 |
| GO:0046879 | hormone secretion | 16/449 | 281/18800 | 0.001247 | 0.017812 | FFAR4/TBX3/KLF7/AGTR1/ADCY8/IL1B/NOS2/BMP6/P2RY1/FFAR2/ILDR2/EDN1/GPER1/TNF/GHRHR/IFNG |
| GO:1905952 | regulation of lipid localization | 11/449 | 155/18800 | 0.001268 | 0.018047 | LPL/AGTR1/IL1A/P2RX7/IL1B/MSR1/BMP6/ABCA3/EDN1/TNF/ENPP7 |
| GO:0032370 | positive regulation of lipid transport | 7/449 | 69/18800 | 0.001274 | 0.018069 | IL1A/P2RX7/IL1B/BMP6/ABCA3/EDN1/ENPP7 |
| GO:1901222 | regulation of NIK/NF-kappaB signaling | 9/449 | 110/18800 | 0.00129 | 0.018226 | RTKN2/IL1B/SASH1/NLRP12/AGER/IL18R1/EDN1/TNF/PTPN22 |
| GO:0051349 | positive regulation of lyase activity | 6/449 | 51/18800 | 0.001302 | 0.018334 | EDNRA/NOS2/NOS1/ADRB2/GHRHR/GUCA2A |
| GO:0001667 | ameboidal-type cell migration | 23/449 | 480/18800 | 0.001306 | 0.018334 | SEMA5A/EDNRA/EDNRB/FLT4/BMPER/DLL4/WNT7A/EMP2/ACVRL1/SASH1/SEMA6A/JCAD/HYAL1/FUT1/CDH5/ANGPT4/SEMA5B/EDN1/TNF/TEK/SEMA3G/IFNG/HBEGF |
| GO:0071216 | cellular response to biotic stimulus | 15/449 | 256/18800 | 0.001321 | 0.018431 | EDNRB/IL1A/IL1B/SASH1/NOS2/BMP6/MRC1/CAMP/LILRB2/TNF/LILRA2/PTPN22/ARG1/SERPINE1/CX3CR1 |
| GO:0010594 | regulation of endothelial cell migration | 14/449 | 230/18800 | 0.001322 | 0.018431 | SEMA5A/FLT4/BMPER/DLL4/WNT7A/EMP2/ACVRL1/SASH1/JCAD/FUT1/ANGPT4/EDN1/TNF/TEK |
| GO:0090130 | tissue migration | 19/449 | 366/18800 | 0.001361 | 0.018789 | SEMA5A/FLT4/BMPER/DLL4/WNT7A/EMP2/FOXF1/ACVRL1/SASH1/JCAD/HYAL1/FUT1/CDH5/ANGPT4/EDN1/TNF/TEK/IFNG/HBEGF |
| GO:0001569 | branching involved in blood vessel morphogenesis | 5/449 | 35/18800 | 0.001366 | 0.018789 | EDNRA/NOTCH4/DLL4/COL4A1/EDN1 |
| GO:0031646 | positive regulation of nervous system process | 5/449 | 35/18800 | 0.001366 | 0.018789 | MYRF/WASF3/TNR/CST7/ZNF488 |
| GO:0035137 | hindlimb morphogenesis | 5/449 | 35/18800 | 0.001366 | 0.018789 | AFF3/TBX3/WNT7A/RSPO2/MSX1 |
| GO:0045088 | regulation of innate immune response | 14/449 | 231/18800 | 0.001377 | 0.018821 | CADM1/FPR2/KLRD1/NLRC4/FGR/TLR8/SH2D1B/FFAR2/MNDA/LILRA2/PTPN22/TXK/ARG1/NCF1 |
| GO:0046883 | regulation of hormone secretion | 14/449 | 231/18800 | 0.001377 | 0.018821 | FFAR4/KLF7/AGTR1/ADCY8/IL1B/NOS2/BMP6/P2RY1/FFAR2/EDN1/GPER1/TNF/GHRHR/IFNG |
| GO:0007585 | respiratory gaseous exchange by respiratory system | 7/449 | 70/18800 | 0.001387 | 0.018825 | EDNRA/FLT4/SFTPC/CSF2RB/TNNC1/SFTPA1/EDN1 |
| GO:0032305 | positive regulation of icosanoid secretion | 4/449 | 21/18800 | 0.001391 | 0.018825 | IL1A/P2RX7/IL1B/EDN1 |
| GO:0060973 | cell migration involved in heart development | 4/449 | 21/18800 | 0.001391 | 0.018825 | EDNRA/TBX5/NDRG4/EDN1 |
| GO:0042045 | epithelial fluid transport | 3/449 | 10/18800 | 0.001433 | 0.019152 | EDNRB/EDN1/SLC5A1 |
| GO:0051918 | negative regulation of fibrinolysis | 3/449 | 10/18800 | 0.001433 | 0.019152 | THBD/SERPINE1/CPB2 |
| GO:0071389 | cellular response to mineralocorticoid stimulus | 3/449 | 10/18800 | 0.001433 | 0.019152 | EDN1/GPER1/ACE |
| GO:0072001 | renal system development | 17/449 | 312/18800 | 0.001434 | 0.019152 | EDNRA/COL4A4/EDNRB/LRP4/BMPER/COL4A3/HEYL/HPGD/AGTR1/FOXF1/SMAD6/BMP6/COL4A1/LRRK2/ACE/GPR4/TEK |
| GO:0060021 | roof of mouth development | 8/449 | 91/18800 | 0.001497 | 0.019932 | TBX3/WNT7A/PKDCC/CLDN5/TGFBR3/MMP25/LRRC32/MSX1 |
| GO:2001259 | positive regulation of cation channel activity | 7/449 | 71/18800 | 0.001508 | 0.019997 | EDNRA/HAP1/ARC/ADRB2/STAC/EDN1/IFNG |
| GO:0030879 | mammary gland development | 10/449 | 135/18800 | 0.001512 | 0.019997 | APLN/NOTCH4/TBX3/NRG3/FOXF1/PRLR/GHRHR/ARG1/SOSTDC1/MSX1 |
| GO:0043405 | regulation of MAP kinase activity | 12/449 | 183/18800 | 0.001535 | 0.0201 | TNXB/SPRY4/P2RX7/IL1B/SASH1/S100A12/AGER/EDN1/LRRK2/TNF/PLA2G1B/PTPN22 |
| GO:0090087 | regulation of peptide transport | 12/449 | 183/18800 | 0.001535 | 0.0201 | CA2/FFAR4/KLF7/ADCY8/IL1B/NOS2/FFAR2/S100A8/GPER1/TNF/GHRHR/IFNG |
| GO:0070588 | calcium ion transmembrane transport | 17/449 | 314/18800 | 0.001536 | 0.0201 | UBASH3B/EDNRA/TRPV2/EDNRB/MCOLN3/SLC24A4/TRPC3/GPM6A/HAP1/P2RX7/RAMP3/MCOLN2/NOS1/STAC/F2RL3/EDN1/GPER1 |
| GO:1903522 | regulation of blood circulation | 15/449 | 260/18800 | 0.001539 | 0.0201 | APLN/EDNRA/EDNRB/TBX5/EPAS1/AGTR1/GJA5/ADRA1D/NOS1/AGER/EDN1/DSC2/ACE/TNF/HBEGF |
| GO:0072132 | mesenchyme morphogenesis | 6/449 | 53/18800 | 0.001594 | 0.020715 | HEYL/TBX3/FOXF1/ACVRL1/TMEM100/MSX1 |
| GO:0009581 | detection of external stimulus | 10/449 | 136/18800 | 0.001598 | 0.020715 | SLC24A4/RS1/TRPC3/FOXF1/TTN/CABP4/SEMA5B/TNF/TCAP/RGR |
| GO:0009953 | dorsal/ventral pattern formation | 8/449 | 92/18800 | 0.001607 | 0.020715 | LRP4/DISP1/DLL4/WNT7A/SMAD6/ACVRL1/EDN1/SOSTDC1 |
| GO:1903035 | negative regulation of response to wounding | 8/449 | 92/18800 | 0.001607 | 0.020715 | UBASH3B/MYOZ1/TNR/EDN1/THBD/TNF/SERPINE1/CPB2 |
| GO:0050804 | modulation of chemical synaptic transmission | 21/449 | 429/18800 | 0.001613 | 0.020729 | CA2/RIMS4/HAP1/NRG3/WNT7A/GRIA1/ARC/ADCY8/IL1B/ADRB2/P2RY1/AGER/TNR/EDN1/LILRB2/SLC6A4/GPER1/LRRK2/TNF/FAM107A/CX3CR1 |
| GO:0006801 | superoxide metabolic process | 7/449 | 72/18800 | 0.001636 | 0.020968 | FPR2/NOS2/PREX1/BST1/EDN1/NCF1B/NCF1 |
| GO:0099177 | regulation of trans-synaptic signaling | 21/449 | 430/18800 | 0.001659 | 0.021185 | CA2/RIMS4/HAP1/NRG3/WNT7A/GRIA1/ARC/ADCY8/IL1B/ADRB2/P2RY1/AGER/TNR/EDN1/LILRB2/SLC6A4/GPER1/LRRK2/TNF/FAM107A/CX3CR1 |
| GO:0010829 | negative regulation of glucose transmembrane transport | 4/449 | 22/18800 | 0.001669 | 0.021185 | ENPP1/IL1B/ACE/TNF |
| GO:0032303 | regulation of icosanoid secretion | 4/449 | 22/18800 | 0.001669 | 0.021185 | IL1A/P2RX7/IL1B/EDN1 |
| GO:0006959 | humoral immune response | 17/449 | 317/18800 | 0.001699 | 0.021498 | COLEC10/CD5L/TREM1/MASP1/PGC/C1QA/SLC11A1/IL1B/S100A12/BST1/GNLY/CFP/CAMP/FCN3/TNF/PLA2G1B/IFNG |
| GO:0050806 | positive regulation of synaptic transmission | 11/449 | 161/18800 | 0.00172 | 0.0217 | CA2/HAP1/ARC/ADCY8/AGER/TNR/LILRB2/GPER1/TNF/FAM107A/CX3CR1 |
| GO:0048863 | stem cell differentiation | 13/449 | 211/18800 | 0.001744 | 0.02193 | SEMA5A/EDNRA/EDNRB/TBX5/TAL1/GPM6A/WNT7A/SEMA6A/SEMA5B/EDN1/ACE/SEMA3G/MSX1 |
| GO:1901652 | response to peptide | 23/449 | 491/18800 | 0.001751 | 0.021953 | LPL/CA2/EDNRA/EDNRB/ENPP1/SLC24A4/FPR2/AGTR1/ADCY8/RAMP3/IL1B/TGFBR3/ADRB2/STC2/AGER/EDN1/GPER1/TNF/PLA2G1B/ICAM1/PTPN22/GHRHR/ARG1 |
| GO:0002040 | sprouting angiogenesis | 12/449 | 186/18800 | 0.001761 | 0.021982 | SEMA5A/FLT4/BMPER/ESM1/CEMIP2/DLL4/AGTR1/ACVRL1/SEMA6A/JCAD/FUT1/TEK |
| GO:0003203 | endocardial cushion morphogenesis | 5/449 | 37/18800 | 0.001764 | 0.021982 | HEYL/TBX3/ACVRL1/TMEM100/MSX1 |
| GO:0003281 | ventricular septum development | 7/449 | 73/18800 | 0.001774 | 0.022038 | HEYL/TBX5/TBX3/HEG1/SMAD6/GJA5/TGFBR3 |
| GO:0002705 | positive regulation of leukocyte mediated immunity | 10/449 | 138/18800 | 0.001782 | 0.022079 | CADM1/KLRD1/TBX21/P2RX7/IL1B/NOS2/SH2D1B/IL18R1/TNF/ARG1 |
| GO:1904035 | regulation of epithelial cell apoptotic process | 8/449 | 94/18800 | 0.001844 | 0.022773 | SEMA5A/CDH5/GPER1/TNF/PLA2G1B/ICAM1/TEK/SERPINE1 |
| GO:0043406 | positive regulation of MAP kinase activity | 9/449 | 116/18800 | 0.001868 | 0.023009 | P2RX7/IL1B/SASH1/S100A12/AGER/EDN1/LRRK2/TNF/PLA2G1B |
| GO:0034764 | positive regulation of transmembrane transport | 13/449 | 213/18800 | 0.001895 | 0.023141 | CA2/EDNRA/HAP1/ARC/P2RX7/RAMP3/NOS1/ADRB2/STAC/F2RL3/EDN1/GPER1/IFNG |
| GO:0002043 | blood vessel endothelial cell proliferation involved in sprouting angiogenesis | 6/449 | 55/18800 | 0.001934 | 0.023141 | SEMA5A/BMPER/DLL4/AGTR1/ACVRL1/JCAD |
| GO:1904645 | response to amyloid-beta | 6/449 | 55/18800 | 0.001934 | 0.023141 | FPR2/RAMP3/ADRB2/AGER/TNF/ICAM1 |
| GO:0006936 | muscle contraction | 18/449 | 349/18800 | 0.001935 | 0.023141 | EDNRA/EDNRB/TBX3/SYNM/TTN/GJA5/NOS1/TNNC1/ADRB2/STAC/EDN1/DSC2/GPER1/MYBPC3/TNF/TCAP/CALCRL/TNNT1 |
| GO:0001886 | endothelial cell morphogenesis | 3/449 | 11/18800 | 0.001935 | 0.023141 | NOTCH4/HEG1/COL22A1 |
| GO:0003096 | renal sodium ion transport | 3/449 | 11/18800 | 0.001935 | 0.023141 | EDNRA/EDNRB/EDN1 |
| GO:0006527 | arginine catabolic process | 3/449 | 11/18800 | 0.001935 | 0.023141 | NOS2/NOS1/ARG1 |
| GO:0019372 | lipoxygenase pathway | 3/449 | 11/18800 | 0.001935 | 0.023141 | HPGD/ALOX15B/ALOX5AP |
| GO:0031284 | positive regulation of guanylate cyclase activity | 3/449 | 11/18800 | 0.001935 | 0.023141 | NOS2/NOS1/GUCA2A |
| GO:0086100 | endothelin receptor signaling pathway | 3/449 | 11/18800 | 0.001935 | 0.023141 | EDNRA/EDNRB/EDN1 |
| GO:0031123 | RNA 3'-end processing | 9/449 | 117/18800 | 0.001983 | 0.023506 | CT45A8/CT45A7/CT45A2/CT45A3/CT45A9/CT45A6/CT45A5/CT45A1/CT45A10 |
| GO:0045089 | positive regulation of innate immune response | 10/449 | 140/18800 | 0.001983 | 0.023506 | CADM1/FPR2/KLRD1/NLRC4/TLR8/SH2D1B/FFAR2/MNDA/LILRA2/TXK |
| GO:0050768 | negative regulation of neurogenesis | 10/449 | 140/18800 | 0.001983 | 0.023506 | SEMA5A/LRP4/CDKN2B/WNT7A/IL1B/SEMA6A/TNR/SEMA5B/TNF/SEMA3G |
| GO:0035136 | forelimb morphogenesis | 5/449 | 38/18800 | 0.001992 | 0.02354 | TBX5/TBX3/WNT7A/RSPO2/MSX1 |
| GO:0002228 | natural killer cell mediated immunity | 7/449 | 75/18800 | 0.002074 | 0.024308 | CADM1/KLRD1/UNC13D/FCGR3A/SH2D1B/GZMB/NKG7 |
| GO:0006809 | nitric oxide biosynthetic process | 7/449 | 75/18800 | 0.002074 | 0.024308 | IL1B/NOS2/NOS1/EDN1/TNF/IFNG/CX3CR1 |
| GO:0050848 | regulation of calcium-mediated signaling | 7/449 | 75/18800 | 0.002074 | 0.024308 | SLC24A4/CCL4/MYOZ1/BST1/TMEM100/LRRK2/TNF |
| GO:0002687 | positive regulation of leukocyte migration | 10/449 | 141/18800 | 0.00209 | 0.024424 | FPR2/SPN/CCL4/IL1A/AGER/EDN1/TNF/ICAM1/SERPINE1/CX3CR1 |
| GO:0021700 | developmental maturation | 16/449 | 296/18800 | 0.002121 | 0.024604 | EDNRA/EDNRB/ADAMTS7/SLC24A4/TAL1/GLDN/EPAS1/C1QA/ACVRL1/CDH5/TRIM58/EDN1/LRRK2/PHOSPHO1/GHRHR/CX3CR1 |
| GO:0050879 | multicellular organismal movement | 6/449 | 56/18800 | 0.002124 | 0.024604 | SYNM/TNNC1/STAC/TNF/TCAP/TNNT1 |
| GO:0050881 | musculoskeletal movement | 6/449 | 56/18800 | 0.002124 | 0.024604 | SYNM/TNNC1/STAC/TNF/TCAP/TNNT1 |
| GO:0009410 | response to xenobiotic stimulus | 20/449 | 411/18800 | 0.00221 | 0.025485 | LPL/KCNK3/CYP3A5/P2RX7/IL1B/NOS2/S100A12/NOS1/SULT1A2/ABCA3/SULT1B1/EDN1/SLC6A4/APOBEC3A/ACE/TNF/DUSP6/ARG1/DPEP1/CPB2 |
| GO:0050878 | regulation of body fluid levels | 19/449 | 382/18800 | 0.002212 | 0.025485 | UBASH3B/APLN/EDNRB/HEG1/EMP2/GJA5/NPR1/P2RY1/F2RL3/PRLR/NFE2/EDN1/THBD/GHRHR/TXK/CHRM1/MPIG6B/SERPINE1/CPB2 |
| GO:0010810 | regulation of cell-substrate adhesion | 13/449 | 217/18800 | 0.002232 | 0.025647 | SPRY4/EMP2/SPOCK2/UNC13D/FOXF1/COL26A1/ACVRL1/PREX1/BST1/FUT1/TEK/FAM107A/SERPINE1 |
| GO:0061045 | negative regulation of wound healing | 7/449 | 76/18800 | 0.002239 | 0.025651 | UBASH3B/MYOZ1/EDN1/THBD/TNF/SERPINE1/CPB2 |
| GO:0002532 | production of molecular mediator involved in inflammatory response | 8/449 | 97/18800 | 0.00225 | 0.025711 | MEFV/IL17D/NOS2/ALOX5AP/TNF/SERPINE1/IL1R2/NCF1 |
| GO:0010001 | glial cell differentiation | 13/449 | 218/18800 | 0.002324 | 0.026434 | MYRF/LAMC3/FPR2/TAL1/WASF3/C1QA/IL1B/ARHGEF10/AGER/S100A8/TNF/IFNG/ZNF488 |
| GO:0046456 | icosanoid biosynthetic process | 6/449 | 57/18800 | 0.002327 | 0.026434 | PLA2G4F/IL1B/ALOX5AP/GGT1/EDN1/PLA2G1B |
| GO:0019835 | cytolysis | 4/449 | 24/18800 | 0.002335 | 0.026451 | PRF1/GZMB/GZMH/GZMM |
| GO:0032729 | positive regulation of interferon-gamma production | 7/449 | 77/18800 | 0.002413 | 0.027194 | SLC11A1/IL1B/TLR8/IL18R1/TNF/PTPN22/TXK |
| GO:0051339 | regulation of lyase activity | 7/449 | 77/18800 | 0.002413 | 0.027194 | EDNRA/EDNRB/NOS2/NOS1/ADRB2/GHRHR/GUCA2A |
| GO:0006690 | icosanoid metabolic process | 9/449 | 121/18800 | 0.002495 | 0.02789 | DPEP2/PLA2G4F/HPGD/IL1B/ALOX15B/ALOX5AP/GGT1/EDN1/PLA2G1B |
| GO:0030282 | bone mineralization | 9/449 | 121/18800 | 0.002495 | 0.02789 | ENPP1/PKDCC/FGR/P2RX7/ADRB2/BMP6/PHOSPHO1/RSPO2/ALPL |
| GO:0048675 | axon extension | 9/449 | 121/18800 | 0.002495 | 0.02789 | SEMA5A/EDNRA/TRPV2/SEMA6A/TNR/SEMA5B/EDN1/RAPH1/SEMA3G |
| GO:0048638 | regulation of developmental growth | 17/449 | 329/18800 | 0.002505 | 0.027927 | SEMA5A/TRPV2/TBX5/MFSD2A/TGFBR3/SEMA6A/MYOZ1/ADRB2/STC2/SPAAR/TNR/SEMA5B/EDN1/SLC6A4/DUSP6/GHRHR/SEMA3G |
| GO:0001867 | complement activation, lectin pathway | 3/449 | 12/18800 | 0.002535 | 0.02803 | COLEC10/MASP1/FCN3 |
| GO:0031650 | regulation of heat generation | 3/449 | 12/18800 | 0.002535 | 0.02803 | EDNRB/IL1B/TNF |
| GO:1900272 | negative regulation of long-term synaptic potentiation | 3/449 | 12/18800 | 0.002535 | 0.02803 | AGER/FAM107A/CX3CR1 |
| GO:0010631 | epithelial cell migration | 18/449 | 358/18800 | 0.002547 | 0.028083 | SEMA5A/FLT4/BMPER/DLL4/WNT7A/EMP2/ACVRL1/SASH1/JCAD/HYAL1/FUT1/CDH5/ANGPT4/EDN1/TNF/TEK/IFNG/HBEGF |
| GO:0019233 | sensory perception of pain | 8/449 | 99/18800 | 0.002558 | 0.028136 | EDNRB/IL1A/P2RX7/P2RY1/TMEM100/EDN1/ACE/TNF |
| GO:0051961 | negative regulation of nervous system development | 10/449 | 145/18800 | 0.002566 | 0.028146 | SEMA5A/LRP4/CDKN2B/WNT7A/IL1B/SEMA6A/TNR/SEMA5B/TNF/SEMA3G |
| GO:0009064 | glutamine family amino acid metabolic process | 7/449 | 78/18800 | 0.002598 | 0.028343 | SLC39A8/PRODH/ASRGL1/NOS2/NOS1/GGT1/ARG1 |
| GO:0031279 | regulation of cyclase activity | 7/449 | 78/18800 | 0.002598 | 0.028343 | EDNRA/EDNRB/NOS2/NOS1/ADRB2/GHRHR/GUCA2A |
| GO:0031214 | biomineral tissue development | 11/449 | 170/18800 | 0.002637 | 0.028691 | ENPP1/SLC24A4/PKDCC/FGR/P2RX7/ADRB2/BMP6/ODAM/PHOSPHO1/RSPO2/ALPL |
| GO:0051235 | maintenance of location | 17/449 | 331/18800 | 0.002667 | 0.028941 | LPL/UBASH3B/MCOLN3/ENPP1/STARD4/HAP1/P2RX7/IL1B/MSR1/MCOLN2/NOS1/TMSB15A/FFAR2/F2RL3/S100A8/GPER1/TNF |
| GO:0106106 | cold-induced thermogenesis | 10/449 | 146/18800 | 0.002698 | 0.029123 | TRPV2/ACADL/FFAR4/FABP4/EPAS1/ADRB2/IL18R1/SCD/PRLR/ALPL |
| GO:0120161 | regulation of cold-induced thermogenesis | 10/449 | 146/18800 | 0.002698 | 0.029123 | TRPV2/ACADL/FFAR4/FABP4/EPAS1/ADRB2/IL18R1/SCD/PRLR/ALPL |
| GO:0003071 | renal system process involved in regulation of systemic arterial blood pressure | 4/449 | 25/18800 | 0.002727 | 0.029213 | EDNRB/EMP2/AGTR1/GJA5 |
| GO:0035821 | modulation of process of another organism | 4/449 | 25/18800 | 0.002727 | 0.029213 | P2RX7/NOS2/PRF1/IFNG |
| GO:2000193 | positive regulation of fatty acid transport | 4/449 | 25/18800 | 0.002727 | 0.029213 | IL1A/P2RX7/IL1B/EDN1 |
| GO:0090132 | epithelium migration | 18/449 | 361/18800 | 0.002784 | 0.02961 | SEMA5A/FLT4/BMPER/DLL4/WNT7A/EMP2/ACVRL1/SASH1/JCAD/HYAL1/FUT1/CDH5/ANGPT4/EDN1/TNF/TEK/IFNG/HBEGF |
| GO:1901653 | cellular response to peptide | 18/449 | 361/18800 | 0.002784 | 0.02961 | LPL/CA2/EDNRA/ENPP1/FPR2/AGTR1/ADCY8/IL1B/ADRB2/AGER/EDN1/GPER1/TNF/PLA2G1B/ICAM1/PTPN22/GHRHR/ARG1 |
| GO:0046323 | glucose import | 7/449 | 79/18800 | 0.002793 | 0.02961 | ENPP1/SLC2A3/SLC5A1/ACE/TNF/PLA2G1B/SLC2A14 |
| GO:0048844 | artery morphogenesis | 7/449 | 79/18800 | 0.002793 | 0.02961 | EDNRA/HPGD/DLL4/FOXF1/GJA5/ACVRL1/EDN1 |
| GO:0042119 | neutrophil activation | 5/449 | 41/18800 | 0.002805 | 0.029655 | CXCR2/PREX1/CAMP/TNF/LILRA2 |
| GO:0110148 | biomineralization | 11/449 | 172/18800 | 0.002886 | 0.03044 | ENPP1/SLC24A4/PKDCC/FGR/P2RX7/ADRB2/BMP6/ODAM/PHOSPHO1/RSPO2/ALPL |
| GO:0001818 | negative regulation of cytokine production | 18/449 | 363/18800 | 0.002952 | 0.03105 | IL1RL1/FFAR4/SLC11A1/MEFV/TBX21/TLR8/NLRP12/AGER/LRRC32/TNF/PTPN22/LILRA5/ARG1/IFNG/IL1R2/GBP7/CX3CR1/ORM1 |
| GO:0001570 | vasculogenesis | 7/449 | 80/18800 | 0.003 | 0.031401 | TBX5/HEG1/WNT7A/EMP2/FOXF1/TGFBR3/TMEM100 |
| GO:0002822 | regulation of adaptive immune response based on somatic recombination of immune receptors built from immunoglobulin superfamily domains | 11/449 | 173/18800 | 0.003018 | 0.031401 | IL1RL1/KLRD1/IL7R/SLC11A1/TBX21/P2RX7/IL1B/AGER/IL18R1/TNF/ARG1 |
| GO:0071772 | response to BMP | 11/449 | 173/18800 | 0.003018 | 0.031401 | ADAMTS7/BMPER/HEYL/SMAD6/ACVRL1/TGFBR3/BMP6/CDH5/TMEM100/SOSTDC1/MSX1 |
| GO:0071773 | cellular response to BMP stimulus | 11/449 | 173/18800 | 0.003018 | 0.031401 | ADAMTS7/BMPER/HEYL/SMAD6/ACVRL1/TGFBR3/BMP6/CDH5/TMEM100/SOSTDC1/MSX1 |
| GO:0043506 | regulation of JUN kinase activity | 6/449 | 60/18800 | 0.003023 | 0.031401 | TNXB/SASH1/AGER/EDN1/TNF/PTPN22 |
| GO:0043542 | endothelial cell migration | 15/449 | 279/18800 | 0.003034 | 0.031404 | SEMA5A/FLT4/BMPER/DLL4/WNT7A/EMP2/ACVRL1/SASH1/JCAD/FUT1/CDH5/ANGPT4/EDN1/TNF/TEK |
| GO:0031589 | cell-substrate adhesion | 18/449 | 364/18800 | 0.003039 | 0.031404 | LAMC3/TNXB/ITGA1/SPRY4/EMP2/SPOCK2/UNC13D/FOXF1/SMAD6/COL26A1/ACVRL1/MYO1G/PREX1/BST1/FUT1/TEK/FAM107A/SERPINE1 |
| GO:0002790 | peptide secretion | 13/449 | 225/18800 | 0.003054 | 0.031484 | FFAR4/KLF7/ADCY8/IL1B/NOS2/FFAR2/S100A8/ILDR2/EDN1/GPER1/TNF/GHRHR/IFNG |
| GO:0022408 | negative regulation of cell-cell adhesion | 12/449 | 199/18800 | 0.003081 | 0.031676 | UBASH3B/NOTCH4/PAG1/SPN/TBX21/BMP6/ILDR2/TNR/LRRC32/LILRB2/PTPN22/ARG1 |
| GO:0016525 | negative regulation of angiogenesis | 10/449 | 149/18800 | 0.003125 | 0.032055 | COL4A3/COL4A2/NPR1/CLDN5/SEMA6A/ANGPT4/TNF/GPR4/TEK/SERPINE1 |
| GO:0062009 | secondary palate development | 4/449 | 26/18800 | 0.003163 | 0.032364 | WNT7A/TGFBR3/MMP25/LRRC32 |
| GO:0034284 | response to monosaccharide | 12/449 | 200/18800 | 0.003209 | 0.032746 | LPL/COL4A3/KLF7/IL1A/ADCY8/AGER/ILDR2/GPER1/ACE/ICAM1/GHRHR/CPB2 |
| GO:0046209 | nitric oxide metabolic process | 7/449 | 81/18800 | 0.003217 | 0.032756 | IL1B/NOS2/NOS1/EDN1/TNF/IFNG/CX3CR1 |
| GO:0035810 | positive regulation of urine volume | 3/449 | 13/18800 | 0.003238 | 0.032799 | EDNRB/NPR1/EDN1 |
| GO:0035815 | positive regulation of renal sodium excretion | 3/449 | 13/18800 | 0.003238 | 0.032799 | EDNRB/NPR1/EDN1 |
| GO:0035335 | peptidyl-tyrosine dephosphorylation | 8/449 | 103/18800 | 0.003273 | 0.033065 | UBASH3B/PTPN5/PALD1/PTPRB/PTPRQ/DUSP6/DUSP8/PTPN22 |
| GO:0034113 | heterotypic cell-cell adhesion | 6/449 | 61/18800 | 0.003287 | 0.033065 | GLDN/IL1B/AGER/LILRB2/DSC2/TNF |
| GO:0002706 | regulation of lymphocyte mediated immunity | 11/449 | 175/18800 | 0.003296 | 0.033065 | CADM1/KLRD1/IL7R/TBX21/P2RX7/IL1B/SH2D1B/AGER/IL18R1/TNF/ARG1 |
| GO:0046890 | regulation of lipid biosynthetic process | 11/449 | 175/18800 | 0.003296 | 0.033065 | ACADL/STARD4/MFSD2A/IL1A/IL1B/BMP6/ABCA3/GPER1/TNF/IFNG/ENPP7 |
| GO:0006909 | phagocytosis | 16/449 | 310/18800 | 0.00335 | 0.033523 | SIRPB1/COLEC10/FPR2/BIN2/UNC13D/SLC11A1/FGR/P2RX7/IL1B/MYO1G/MSR1/SFTPA1/FCN3/TNF/CEACAM4/IFNG |
| GO:2000181 | negative regulation of blood vessel morphogenesis | 10/449 | 151/18800 | 0.003439 | 0.03433 | COL4A3/COL4A2/NPR1/CLDN5/SEMA6A/ANGPT4/TNF/GPR4/TEK/SERPINE1 |
| GO:2001057 | reactive nitrogen species metabolic process | 7/449 | 82/18800 | 0.003447 | 0.03433 | IL1B/NOS2/NOS1/EDN1/TNF/IFNG/CX3CR1 |
| GO:0016339 | calcium-dependent cell-cell adhesion via plasma membrane cell adhesion molecules | 5/449 | 43/18800 | 0.003465 | 0.034341 | CDH19/CDH5/PCDH12/CDH16/PCDHGB4 |
| GO:0045214 | sarcomere organization | 5/449 | 43/18800 | 0.003465 | 0.034341 | TTN/MYOZ1/EDN1/TCAP/TNNT1 |
| GO:0050830 | defense response to Gram-positive bacterium | 8/449 | 104/18800 | 0.003473 | 0.034341 | IL7R/FGR/GBP4/P2RX7/CAMP/TNF/PLA2G1B/GBP7 |
| GO:0031663 | lipopolysaccharide-mediated signaling pathway | 6/449 | 62/18800 | 0.003567 | 0.035185 | IL1B/SASH1/BMP6/TNF/LILRA2/PTPN22 |
| GO:0042060 | wound healing | 20/449 | 429/18800 | 0.003594 | 0.035363 | UBASH3B/NOTCH4/WNT7A/SLC11A1/IL1A/ACVRL1/MYOZ1/P2RY1/F2RL3/S100A8/EDN1/THBD/ODAM/TNF/GPR4/TXK/MPIG6B/SERPINE1/CPB2/HBEGF |
| GO:0002675 | positive regulation of acute inflammatory response | 4/449 | 27/18800 | 0.003644 | 0.035435 | IL1B/ALOX5AP/FFAR2/TNF |
| GO:0002710 | negative regulation of T cell mediated immunity | 4/449 | 27/18800 | 0.003644 | 0.035435 | KLRD1/IL7R/TBX21/ARG1 |
| GO:0003171 | atrioventricular valve development | 4/449 | 27/18800 | 0.003644 | 0.035435 | HEYL/TBX5/SMAD6/GJA5 |
| GO:0007263 | nitric oxide mediated signal transduction | 4/449 | 27/18800 | 0.003644 | 0.035435 | GUCY1A2/NOS2/NOS1/FPR1 |
| GO:0048843 | negative regulation of axon extension involved in axon guidance | 4/449 | 27/18800 | 0.003644 | 0.035435 | SEMA5A/SEMA6A/SEMA5B/SEMA3G |
| GO:0034308 | primary alcohol metabolic process | 8/449 | 105/18800 | 0.003683 | 0.035703 | EDNRB/SDR16C5/GPD1/CYP3A5/SULT1A2/BMP6/SULT1B1/AWAT2 |
| GO:0014032 | neural crest cell development | 7/449 | 83/18800 | 0.003689 | 0.035703 | SEMA5A/EDNRA/EDNRB/SEMA6A/SEMA5B/EDN1/SEMA3G |
| GO:0002366 | leukocyte activation involved in immune response | 15/449 | 285/18800 | 0.0037 | 0.035727 | SPN/UNC13D/FOXF1/SLC11A1/FGR/TBX21/SH2D1B/IL18R1/CLEC4E/LILRA2/ICAM1/IFNG/CLEC4D/CX3CR1/NKG7 |
| GO:0006941 | striated muscle contraction | 11/449 | 178/18800 | 0.003751 | 0.036131 | SYNM/TTN/GJA5/NOS1/TNNC1/STAC/DSC2/MYBPC3/TNF/TCAP/TNNT1 |
| GO:0017157 | regulation of exocytosis | 12/449 | 204/18800 | 0.003764 | 0.036171 | RIMS4/SEPTIN4/HAP1/WNT7A/UNC13D/FOXF1/FGR/LGI3/P2RY1/STXBP6/LRRK2/IFNG |
| GO:0071900 | regulation of protein serine/threonine kinase activity | 18/449 | 372/18800 | 0.003816 | 0.036561 | PKIA/HEG1/TNXB/SPRY4/CDKN2B/P2RX7/IL1B/SASH1/S100A12/ADRB2/AGER/EDN1/LRRK2/TNF/PLA2G1B/PTPN22/IFNG/SERTAD1 |
| GO:0001666 | response to hypoxia | 15/449 | 286/18800 | 0.003822 | 0.036561 | EDNRA/KCNK3/EPAS1/IL1A/ACVRL1/TGFBR3/NOS2/NOS1/STC2/AGER/ANGPT4/EDN1/SLC6A4/ACE/TNF |
| GO:0030517 | negative regulation of axon extension | 5/449 | 44/18800 | 0.003834 | 0.036588 | SEMA5A/SEMA6A/TNR/SEMA5B/SEMA3G |
| GO:0051341 | regulation of oxidoreductase activity | 8/449 | 106/18800 | 0.003903 | 0.037076 | AGTR1/IL1A/IL1B/EDN1/LRRK2/TNF/IFNG/HP |
| GO:0060840 | artery development | 8/449 | 106/18800 | 0.003903 | 0.037076 | EDNRA/HPGD/DLL4/FOXF1/SMAD6/GJA5/ACVRL1/EDN1 |
| GO:0045216 | cell-cell junction organization | 12/449 | 205/18800 | 0.003914 | 0.037095 | CDH19/TBX5/HEG1/CLDN18/GJA5/IL1B/CLDN5/BMP6/CDH5/ACE/TNF/GPBAR1 |
| GO:0018108 | peptidyl-tyrosine phosphorylation | 18/449 | 373/18800 | 0.003924 | 0.037098 | LRP4/FLT4/PKDCC/TTN/FGR/BMP6/BST1/ANGPT4/PRLR/ACE/TNF/TEK/LTK/TXK/LILRA5/IFNG/HBEGF/NCF1 |
| GO:0031282 | regulation of guanylate cyclase activity | 3/449 | 14/18800 | 0.004048 | 0.038101 | NOS2/NOS1/GUCA2A |
| GO:0051917 | regulation of fibrinolysis | 3/449 | 14/18800 | 0.004048 | 0.038101 | THBD/SERPINE1/CPB2 |
| GO:0002791 | regulation of peptide secretion | 11/449 | 180/18800 | 0.004081 | 0.038326 | FFAR4/KLF7/ADCY8/IL1B/NOS2/FFAR2/S100A8/GPER1/TNF/GHRHR/IFNG |
| GO:0003272 | endocardial cushion formation | 4/449 | 28/18800 | 0.004173 | 0.038959 | HEYL/TBX3/TMEM100/MSX1 |
| GO:0031638 | zymogen activation | 6/449 | 64/18800 | 0.004181 | 0.038959 | CD5L/NLRC4/GGT1/SERPINE1/CPB2/HP |
| GO:0050771 | negative regulation of axonogenesis | 6/449 | 64/18800 | 0.004181 | 0.038959 | SEMA5A/LRP4/SEMA6A/TNR/SEMA5B/SEMA3G |
| GO:0002263 | cell activation involved in immune response | 15/449 | 289/18800 | 0.004208 | 0.038959 | SPN/UNC13D/FOXF1/SLC11A1/FGR/TBX21/SH2D1B/IL18R1/CLEC4E/LILRA2/ICAM1/IFNG/CLEC4D/CX3CR1/NKG7 |
| GO:0050886 | endocrine process | 7/449 | 85/18800 | 0.004212 | 0.038959 | EDNRB/TBX3/AGTR1/IL1B/BMP6/EDN1/ACE |
| GO:0042551 | neuron maturation | 5/449 | 45/18800 | 0.00423 | 0.038959 | EDNRA/EDNRB/GLDN/C1QA/LRRK2 |
| GO:0051180 | vitamin transport | 5/449 | 45/18800 | 0.00423 | 0.038959 | SLC2A6/SLC2A3/SLC19A3/FOLR3/SLC2A14 |
| GO:1904646 | cellular response to amyloid-beta | 5/449 | 45/18800 | 0.00423 | 0.038959 | FPR2/ADRB2/AGER/TNF/ICAM1 |
| GO:0006022 | aminoglycan metabolic process | 9/449 | 131/18800 | 0.004244 | 0.038959 | EDNRA/EDNRB/CEMIP2/B3GNT8/SPOCK2/IL1B/GALNT5/HYAL1/CHST1 |
| GO:0010595 | positive regulation of endothelial cell migration | 9/449 | 131/18800 | 0.004244 | 0.038959 | SEMA5A/FLT4/WNT7A/SASH1/JCAD/FUT1/ANGPT4/EDN1/TEK |
| GO:0018212 | peptidyl-tyrosine modification | 18/449 | 376/18800 | 0.004262 | 0.039042 | LRP4/FLT4/PKDCC/TTN/FGR/BMP6/BST1/ANGPT4/PRLR/ACE/TNF/TEK/LTK/TXK/LILRA5/IFNG/HBEGF/NCF1 |
| GO:0032640 | tumor necrosis factor production | 11/449 | 182/18800 | 0.004434 | 0.040351 | LPL/SPN/IL1A/AGER/LRRK2/LILRA2/PTPN22/LILRA5/IFNG/CX3CR1/ORM1 |
| GO:0032680 | regulation of tumor necrosis factor production | 11/449 | 182/18800 | 0.004434 | 0.040351 | LPL/SPN/IL1A/AGER/LRRK2/LILRA2/PTPN22/LILRA5/IFNG/CX3CR1/ORM1 |
| GO:0050728 | negative regulation of inflammatory response | 11/449 | 182/18800 | 0.004434 | 0.040351 | FPR2/FFAR4/FOXF1/MEFV/FGR/NLRP12/CDH5/CST7/GPER1/TEK/CALCRL |
| GO:0019915 | lipid storage | 7/449 | 86/18800 | 0.004493 | 0.040704 | LPL/ENPP1/STARD4/IL1B/MSR1/FFAR2/TNF |
| GO:1900542 | regulation of purine nucleotide metabolic process | 7/449 | 86/18800 | 0.004493 | 0.040704 | GPD1/SLC2A6/P2RX7/NOS2/NOS1/IFNG/GUCA2A |
| GO:0030858 | positive regulation of epithelial cell differentiation | 6/449 | 65/18800 | 0.004515 | 0.040705 | CDKN2B/ACVRL1/ALOX15B/BMP6/TMEM100/SERPINE1 |
| GO:0070613 | regulation of protein processing | 6/449 | 65/18800 | 0.004515 | 0.040705 | NLRC4/CST7/LRRK2/SERPINE1/CPB2/IL1R2 |
| GO:0031960 | response to corticosteroid | 10/449 | 157/18800 | 0.004533 | 0.040705 | BMP6/ABCA3/EDN1/GPER1/ACE/TNF/GHRHR/ARG1/FAM107A/ALPL |
| GO:0055067 | monovalent inorganic cation homeostasis | 10/449 | 157/18800 | 0.004533 | 0.040705 | CA2/EDNRA/EDNRB/AGTR1/SLC11A1/IL1A/NPR1/EDN1/LRRK2/ACE |
| GO:1900744 | regulation of p38MAPK cascade | 5/449 | 46/18800 | 0.004655 | 0.041712 | IL1B/SASH1/AGER/PTPN22/NCF1 |
| GO:0060389 | pathway-restricted SMAD protein phosphorylation | 6/449 | 66/18800 | 0.004869 | 0.043536 | BMPER/SMAD6/ACVRL1/TGFBR3/BMP6/INHBC |
| GO:0008016 | regulation of heart contraction | 12/449 | 211/18800 | 0.00492 | 0.043598 | APLN/EDNRA/EDNRB/TBX5/EPAS1/GJA5/ADRA1D/NOS1/EDN1/DSC2/TNF/HBEGF |
| GO:1990845 | adaptive thermogenesis | 10/449 | 159/18800 | 0.004952 | 0.043598 | TRPV2/ACADL/FFAR4/FABP4/EPAS1/ADRB2/IL18R1/SCD/PRLR/ALPL |
| GO:0003214 | cardiac left ventricle morphogenesis | 3/449 | 15/18800 | 0.004971 | 0.043598 | EDNRA/TBX5/FOXF1 |
| GO:0032725 | positive regulation of granulocyte macrophage colony-stimulating factor production | 3/449 | 15/18800 | 0.004971 | 0.043598 | IL17D/IL1B/LILRA2 |
| GO:0034116 | positive regulation of heterotypic cell-cell adhesion | 3/449 | 15/18800 | 0.004971 | 0.043598 | IL1B/AGER/TNF |
| GO:0043129 | surfactant homeostasis | 3/449 | 15/18800 | 0.004971 | 0.043598 | EPAS1/NAPSA/ABCA3 |
| GO:0071639 | positive regulation of monocyte chemotactic protein-1 production | 3/449 | 15/18800 | 0.004971 | 0.043598 | IL1B/MCOLN2/AGER |
| GO:0086069 | bundle of His cell to Purkinje myocyte communication | 3/449 | 15/18800 | 0.004971 | 0.043598 | TBX5/GJA5/DSC2 |
| GO:1902287 | semaphorin-plexin signaling pathway involved in axon guidance | 3/449 | 15/18800 | 0.004971 | 0.043598 | EDNRA/PLXNB3/EDN1 |
| GO:0006140 | regulation of nucleotide metabolic process | 7/449 | 88/18800 | 0.005098 | 0.044284 | GPD1/SLC2A6/P2RX7/NOS2/NOS1/IFNG/GUCA2A |
| GO:1903510 | mucopolysaccharide metabolic process | 7/449 | 88/18800 | 0.005098 | 0.044284 | EDNRA/EDNRB/CEMIP2/SPOCK2/IL1B/HYAL1/CHST1 |
| GO:0002673 | regulation of acute inflammatory response | 5/449 | 47/18800 | 0.005109 | 0.044284 | EDNRB/IL1B/ALOX5AP/FFAR2/TNF |
| GO:0036230 | granulocyte activation | 5/449 | 47/18800 | 0.005109 | 0.044284 | CXCR2/PREX1/CAMP/TNF/LILRA2 |
| GO:0050919 | negative chemotaxis | 5/449 | 47/18800 | 0.005109 | 0.044284 | SEMA5A/NRG3/SEMA6A/SEMA5B/SEMA3G |
| GO:0070482 | response to oxygen levels | 16/449 | 324/18800 | 0.005114 | 0.044284 | EDNRA/KCNK3/GUCY1A2/EPAS1/IL1A/ACVRL1/TGFBR3/NOS2/NOS1/STC2/AGER/ANGPT4/EDN1/SLC6A4/ACE/TNF |
| GO:0002704 | negative regulation of leukocyte mediated immunity | 6/449 | 67/18800 | 0.005243 | 0.045207 | KLRD1/IL7R/FOXF1/TBX21/ARG1/CX3CR1 |
| GO:0009583 | detection of light stimulus | 6/449 | 67/18800 | 0.005243 | 0.045207 | SLC24A4/RS1/TRPC3/CABP4/SEMA5B/RGR |
| GO:0001945 | lymph vessel development | 4/449 | 30/18800 | 0.005379 | 0.045997 | FLT4/HEG1/ACVRL1/TMEM204 |
| GO:0033198 | response to ATP | 4/449 | 30/18800 | 0.005379 | 0.045997 | TRPC3/P2RX7/IL1B/P2RY1 |
| GO:0060251 | regulation of glial cell proliferation | 4/449 | 30/18800 | 0.005379 | 0.045997 | CDKN2B/IL1B/TNF/CHRM1 |
| GO:0010720 | positive regulation of cell development | 15/449 | 297/18800 | 0.005395 | 0.045997 | SEMA5A/TRPV2/PLXNB3/HAP1/UNC13D/IL1B/CLDN5/PREX1/CDH5/GPER1/ACE/TNF/IFNG/CX3CR1/ZNF488 |
| GO:0051250 | negative regulation of lymphocyte activation | 10/449 | 161/18800 | 0.005402 | 0.045997 | PAG1/SPN/FGR/TBX21/ILDR2/MNDA/LRRC32/LILRB2/PTPN22/ARG1 |
| GO:0001558 | regulation of cell growth | 19/449 | 415/18800 | 0.005419 | 0.045997 | SEMA5A/TRPV2/ENPP1/NRG3/AGTR1/MFSD2A/ACVRL1/NPR1/SEMA6A/HYAL1/S100A8/TNR/SEMA5B/EDN1/FAM107A/SEMA3G/MSX1/HBEGF/PAPPA2 |
| GO:0071706 | tumor necrosis factor superfamily cytokine production | 11/449 | 187/18800 | 0.005424 | 0.045997 | LPL/SPN/IL1A/AGER/LRRK2/LILRA2/PTPN22/LILRA5/IFNG/CX3CR1/ORM1 |
| GO:1903555 | regulation of tumor necrosis factor superfamily cytokine production | 11/449 | 187/18800 | 0.005424 | 0.045997 | LPL/SPN/IL1A/AGER/LRRK2/LILRA2/PTPN22/LILRA5/IFNG/CX3CR1/ORM1 |
| GO:0030195 | negative regulation of blood coagulation | 5/449 | 48/18800 | 0.005593 | 0.04724 | UBASH3B/EDN1/THBD/SERPINE1/CPB2 |
| GO:0031641 | regulation of myelination | 5/449 | 48/18800 | 0.005593 | 0.04724 | MYRF/WASF3/CST7/TNF/ZNF488 |
| GO:1903317 | regulation of protein maturation | 6/449 | 68/18800 | 0.005637 | 0.047352 | NLRC4/CST7/LRRK2/SERPINE1/CPB2/IL1R2 |
| GO:0001837 | epithelial to mesenchymal transition | 10/449 | 162/18800 | 0.005639 | 0.047352 | EDNRA/NOTCH4/HEYL/TBX5/TBX3/IL1B/TGFBR3/TMEM100/EDN1/MSX1 |
| GO:0002819 | regulation of adaptive immune response | 11/449 | 188/18800 | 0.005641 | 0.047352 | IL1RL1/KLRD1/IL7R/SLC11A1/TBX21/P2RX7/IL1B/AGER/IL18R1/TNF/ARG1 |
| GO:0036293 | response to decreased oxygen levels | 15/449 | 299/18800 | 0.00573 | 0.047969 | EDNRA/KCNK3/EPAS1/IL1A/ACVRL1/TGFBR3/NOS2/NOS1/STC2/AGER/ANGPT4/EDN1/SLC6A4/ACE/TNF |
| GO:0002768 | immune response-regulating cell surface receptor signaling pathway | 16/449 | 328/18800 | 0.005738 | 0.047969 | FPR2/KLRD1/FGR/MYO1G/FCGR3A/FPR1/FFAR2/MNDA/LILRB2/CLEC4E/BTNL9/LILRA2/PTPN22/TXK/BTNL8/CLEC4D |
| GO:0042475 | odontogenesis of dentin-containing tooth | 7/449 | 90/18800 | 0.005762 | 0.048073 | LRP4/SLC24A4/ODAM/RSPO2/SOSTDC1/SERPINE1/MSX1 |
| GO:0016485 | protein processing | 13/449 | 243/18800 | 0.005818 | 0.048443 | MYRF/CD5L/DISP1/NLRC4/P2RX7/GGT1/CST7/LRRK2/ACE/SERPINE1/CPB2/IL1R2/HP |
| GO:0009749 | response to glucose | 11/449 | 189/18800 | 0.005865 | 0.048736 | LPL/COL4A3/KLF7/ADCY8/AGER/ILDR2/GPER1/ACE/ICAM1/GHRHR/CPB2 |
| GO:0032943 | mononuclear cell proliferation | 15/449 | 300/18800 | 0.005904 | 0.048959 | SPN/IL7R/SLC11A1/IL1A/P2RX7/IL1B/FCGR3A/BST1/AGER/MNDA/LRRC32/LILRB2/ACE/PTPN22/ARG1 |
| GO:0006828 | manganese ion transport | 3/449 | 16/18800 | 0.006011 | 0.049154 | TRPC3/SLC39A8/SLC11A1 |
| GO:0008228 | opsonization | 3/449 | 16/18800 | 0.006011 | 0.049154 | COLEC10/SFTPA1/FCN3 |
| GO:0009642 | response to light intensity | 3/449 | 16/18800 | 0.006011 | 0.049154 | SLC24A4/RS1/RGS9 |
| GO:0045779 | negative regulation of bone resorption | 3/449 | 16/18800 | 0.006011 | 0.049154 | UBASH3B/CLDN18/P2RX7 |
| GO:0051044 | positive regulation of membrane protein ectodomain proteolysis | 3/449 | 16/18800 | 0.006011 | 0.049154 | IL1B/TNF/IFNG |
| GO:0060841 | venous blood vessel development | 3/449 | 16/18800 | 0.006011 | 0.049154 | HEG1/FOXF1/ACVRL1 |
| GO:1902285 | semaphorin-plexin signaling pathway involved in neuron projection guidance | 3/449 | 16/18800 | 0.006011 | 0.049154 | EDNRA/PLXNB3/EDN1 |
| GO:0003014 | renal system process | 8/449 | 114/18800 | 0.006046 | 0.049339 | EDNRA/EDNRB/EMP2/AGTR1/GJA5/NPR1/EDN1/ACE |
| GO:0061384 | heart trabecula morphogenesis | 4/449 | 31/18800 | 0.006062 | 0.049359 | HEG1/DLL4/TGFBR3/TEK |
| GO:0070661 | leukocyte proliferation | 16/449 | 330/18800 | 0.006072 | 0.049359 | SPN/IL7R/SLC11A1/IL1A/P2RX7/IL1B/FCGR3A/CSF2RB/BST1/AGER/MNDA/LRRC32/LILRB2/ACE/PTPN22/ARG1 |
| GO:0007602 | phototransduction | 5/449 | 49/18800 | 0.00611 | 0.049466 | SLC24A4/RS1/TRPC3/CABP4/RGR |
| GO:1900047 | negative regulation of hemostasis | 5/449 | 49/18800 | 0.00611 | 0.049466 | UBASH3B/EDN1/THBD/SERPINE1/CPB2 |
| GO:0034329 | cell junction assembly | 19/449 | 420/18800 | 0.006138 | 0.049597 | CDH19/LRP4/TBX5/HEG1/GPM6A/CLDN18/WNT7A/SPOCK2/GJA5/IL1B/ACVRL1/CLDN5/CDH5/SLITRK2/ACE/TNF/GPBAR1/TEK/FAM107A |
