# Supplementary material for: Integrative analysis of bulk and single-cell RNA sequencing reveals the gene expression profile and the critical signaling pathways of type II CPAM
Source: Cell Biosci. 2024 Jul 18;14:94. doi: 10.1186/s13578-024-01276-8 (PMC11264590; doi:10.1186/s13578-024-01276-8)
Supplement: Supplementary file 17 — Supplementary Material 17: Supplemental Table 11 Inference and analysis of CPAM-associated epithelial cells communication from single-cell data using CellChat. [file 13578_2024_1276_MOESM17_ESM.docx]

**Supplemental Table 5a GO enrichment analysis (molecular function, MF) of up-regulated genes**

| **ID** | **Description** | **Gene**  **Ratio** | **Bg Ratio** | ***P*-value** | **p.adjust** | **Gene ID** |
| --- | --- | --- | --- | --- | --- | --- |
| GO:0003777 | microtubule motor activity | 21/1006 | 67/18410 | 2.94E-11 | 2.26E-08 | DNAH6/DNAI2/KIF24/KIF19/DNAL4/DNAH5/DNAH11/KIF21A/DYNLRB2/KIF6/DNAH7/DNAH9/DYNC2H1/DNAH12/DNAH10/DNAH2/DNAH3/KIF9/KIF3A/DNAH1/KIF27 |
| GO:0015631 | tubulin binding | 53/1006 | 376/18410 | 2.12E-10 | 7.05E-08 | DNAL1/GAS2L2/CETN2/EFHC2/STMND1/PACRG/MAPRE3/RP1/KIF24/MAP6/SPAG8/TOGARAM2/KIF19/PIFO/SPEF1/SAXO2/C9orf24/TTLL6/TTLL9/DCDC1/TRPV4/KIF21A/EFHC1/KIF6/CFAP157/GAS8/SPAG6/DLEC1/CCDC181/PPP1R42/MAP1A/CCDC170/MDM1/STRBP/CCDC187/TPPP3/IFT81/TRAF3IP1/REEP2/SPATA4/EML6/B9D2/FAM161A/AGBL4/KATNAL2/KIF9/FAM83D/HSPH1/KIF3A/KIF27/KIF26B/SAXO1/MTUS2 |
| GO:0008569 | minus-end-directed microtubule motor activity | 11/1006 | 18/18410 | 2.75E-10 | 7.05E-08 | DNAH6/DNAH5/DNAH11/DNAH7/DNAH9/DYNC2H1/DNAH12/DNAH10/DNAH2/DNAH3/DNAH1 |
| GO:0051959 | dynein light intermediate chain binding | 11/1006 | 27/18410 | 7.13E-08 | 1.37E-05 | DNAH6/DNAL4/DNAH5/DNAH11/DNAH7/DNAH9/DYNC2H1/DNAH10/DNAH2/DNAH3/DNAH1 |
| GO:0003774 | cytoskeletal motor activity | 22/1006 | 111/18410 | 1.21E-07 | 1.87E-05 | DNAH6/DNAI2/DNAI1/KIF24/KIF19/DNAL4/DNAH5/DNAH11/KIF21A/DYNLRB2/KIF6/DNAH7/DNAH9/DYNC2H1/DNAH12/DNAH10/DNAH2/DNAH3/KIF9/KIF3A/DNAH1/KIF27 |
| GO:0045505 | dynein intermediate chain binding | 12/1006 | 36/18410 | 2.45E-07 | 3.15E-05 | DNAH6/DNAL4/DNAH5/DNAH11/DYNLRB2/DNAH7/DNAH9/DYNC2H1/DNAH10/DNAH2/DNAH3/DNAH1 |
| GO:0008017 | microtubule binding | 37/1006 | 272/18410 | 2.86E-07 | 3.15E-05 | GAS2L2/CETN2/MAPRE3/RP1/KIF24/MAP6/SPAG8/TOGARAM2/KIF19/SPEF1/SAXO2/DCDC1/TRPV4/KIF21A/KIF6/CFAP157/GAS8/SPAG6/CCDC181/MAP1A/CCDC170/MDM1/STRBP/CCDC187/TRAF3IP1/REEP2/SPATA4/EML6/FAM161A/KATNAL2/KIF9/FAM83D/KIF3A/KIF27/KIF26B/SAXO1/MTUS2 |
| GO:0004550 | nucleoside diphosphate kinase activity | 6/1006 | 18/18410 | 0.000276 | 0.026549 | NME5/AK8/AK7/NME7/AK9/NME9 |

**Supplemental Table 5b GO enrichment analysis (molecular function, MF) of down-regulated genes**

| **ID** | **Description** | **Gene**  **Ratio** | **Bg Ratio** | ***P*-value** | **p.adjust** | **Gene ID** |
| --- | --- | --- | --- | --- | --- | --- |
| GO:0140375 | immune receptor activity | 21/445 | 148/18410 | 6.90E-11 | 4.27E-08 | IL1RL1/FPR2/KLRD1/IL7R/FCGR3A/CXCR2/CSF2RB/LILRA6/FPR1/LILRA1/IL18R1/PRLR/LILRB2/IL18RAP/LILRA2/GPR17/CXCR1/LILRA5/IL1R2/CX3CR1/LILRB3 |
| GO:0032396 | inhibitory MHC class I receptor activity | 6/445 | 12/18410 | 1.58E-07 | 4.88E-05 | LILRA6/LILRA1/LILRB2/LILRA2/LILRA5/LILRB3 |
| GO:0032393 | MHC class I receptor activity | 6/445 | 17/18410 | 1.90E-06 | 0.000393 | LILRA6/LILRA1/LILRB2/LILRA2/LILRA5/LILRB3 |
| GO:0008528 | G protein-coupled peptide receptor activity | 15/445 | 148/18410 | 3.10E-06 | 0.00048 | EDNRA/EDNRB/VIPR1/FPR2/AGTR1/RAMP3/NPR1/CXCR2/FPR1/F2RL3/GPR17/GHRHR/CXCR1/CALCRL/CX3CR1 |
| GO:0001653 | peptide receptor activity | 15/445 | 154/18410 | 5.08E-06 | 0.000629 | EDNRA/EDNRB/VIPR1/FPR2/AGTR1/RAMP3/NPR1/CXCR2/FPR1/F2RL3/GPR17/GHRHR/CXCR1/CALCRL/CX3CR1 |
| GO:0030246 | carbohydrate binding | 20/445 | 270/18410 | 9.93E-06 | 0.001024 | LGALSL/COLEC10/ENPP1/SIGLEC11/KLRD1/SIGLEC5/HK3/KLRG2/ADGRL2/SLC2A3/GALNT5/MRC1/OLR1/P3H2/SFTPA1/FCN3/CLEC4E/ITLN2/CLEC4D/CLEC4M |
| GO:0004896 | cytokine receptor activity | 11/445 | 97/18410 | 2.23E-05 | 0.001975 | IL1RL1/IL7R/CXCR2/CSF2RB/IL18R1/PRLR/IL18RAP/GPR17/CXCR1/IL1R2/CX3CR1 |
| GO:0055056 | D-glucose transmembrane transporter activity | 4/445 | 11/18410 | 9.71E-05 | 0.007515 | SLC2A6/SLC2A3/SLC5A1/SLC2A14 |
| GO:0005355 | glucose transmembrane transporter activity | 5/445 | 22/18410 | 0.000151 | 0.010155 | SLC2A6/SLC2A3/SLC5A4/SLC5A1/SLC2A14 |
| GO:0019955 | cytokine binding | 12/445 | 141/18410 | 0.000164 | 0.010155 | IL1RL1/ACVRL1/CXCR2/TGFBR3/IL18R1/PRLR/LRRC32/CXCR1/TCAP/SOSTDC1/IL1R2/CX3CR1 |
| GO:0015149 | hexose transmembrane transporter activity | 5/445 | 23/18410 | 0.000189 | 0.010202 | SLC2A6/SLC2A3/SLC5A4/SLC5A1/SLC2A14 |
| GO:0030546 | signaling receptor activator activity | 26/445 | 496/18410 | 0.000198 | 0.010202 | SEMA5A/APLN/COLEC10/IL32/TAL1/CCL4/NRG3/WNT7A/IL1A/IL17D/IL1B/SEMA6A/SECTM1/BMP6/STC2/ANGPT4/SEMA5B/EDN1/TNF/SEMA3G/MIA/IFNG/INHBC/HBEGF/ENHO/GUCA2A |
| GO:0015145 | monosaccharide transmembrane transporter activity | 5/445 | 25/18410 | 0.000287 | 0.013536 | SLC2A6/SLC2A3/SLC5A4/SLC5A1/SLC2A14 |
| GO:0031406 | carboxylic acid binding | 13/445 | 173/18410 | 0.000306 | 0.013536 | FFAR4/FABP4/SIGLEC11/SIGLEC5/NOS2/PADI4/NOS1/ALOX5AP/S100A8/RBP2/P3H2/FOLR3/PLA2G1B |
| GO:0016805 | dipeptidase activity | 4/445 | 15/18410 | 0.000372 | 0.015351 | DPEP3/DPEP2/ACE/DPEP1 |
| GO:0051119 | sugar transmembrane transporter activity | 5/445 | 27/18410 | 0.00042 | 0.0155 | SLC2A6/SLC2A3/SLC5A4/SLC5A1/SLC2A14 |
| GO:0050135 | NAD(P)+ nucleosidase activity | 4/445 | 16/18410 | 0.000487 | 0.0155 | IL1RL1/BST1/IL18R1/IL18RAP |
| GO:0061809 | NAD+ nucleotidase, cyclic ADP-ribose generating | 4/445 | 16/18410 | 0.000487 | 0.0155 | IL1RL1/BST1/IL18R1/IL18RAP |
| GO:0003953 | NAD+ nucleosidase activity | 5/445 | 28/18410 | 0.000501 | 0.0155 | IL1RL1/TLR8/BST1/IL18R1/IL18RAP |
| GO:0045499 | chemorepellent activity | 5/445 | 28/18410 | 0.000501 | 0.0155 | SEMA5A/NRG3/SEMA6A/SEMA5B/SEMA3G |
| GO:0038024 | cargo receptor activity | 8/445 | 79/18410 | 0.000637 | 0.018767 | CD5L/ENPP1/FPR2/APOBR/MSR1/MRC1/OLR1/AGER |
| GO:0001540 | amyloid-beta binding | 8/445 | 81/18410 | 0.000753 | 0.02118 | FPR2/C1QA/GRIA1/RAMP3/MSR1/ADRB2/AGER/LILRB2 |
| GO:0098631 | cell adhesion mediator activity | 7/445 | 64/18410 | 0.000871 | 0.022554 | PLXNB3/ITGA1/CNTN6/GLDN/ROBO4/STXBP6/DSC2 |
| GO:0048018 | receptor ligand activity | 24/445 | 489/18410 | 0.000874 | 0.022554 | SEMA5A/APLN/COLEC10/IL32/CCL4/NRG3/WNT7A/IL1A/IL17D/IL1B/SEMA6A/SECTM1/BMP6/STC2/SEMA5B/EDN1/TNF/SEMA3G/MIA/IFNG/INHBC/HBEGF/ENHO/GUCA2A |
| GO:0050786 | RAGE receptor binding | 3/445 | 10/18410 | 0.001483 | 0.036488 | S100A12/FPR1/S100A8 |
| GO:0017046 | peptide hormone binding | 6/445 | 52/18410 | 0.001533 | 0.036488 | EDNRB/VIPR1/NPR1/PRLR/GHRHR/CALCRL |
| GO:0048029 | monosaccharide binding | 7/445 | 71/18410 | 0.001614 | 0.036996 | COLEC10/HK3/SLC2A3/MRC1/P3H2/CLEC4D/CLEC4M |
| GO:0005537 | mannose binding | 4/445 | 22/18410 | 0.001744 | 0.037216 | COLEC10/MRC1/CLEC4D/CLEC4M |
| GO:0009975 | cyclase activity | 4/445 | 22/18410 | 0.001744 | 0.037216 | GUCY1A2/ADCY8/NPR1/BST1 |
| GO:0098632 | cell-cell adhesion mediator activity | 6/445 | 54/18410 | 0.001867 | 0.038526 | PLXNB3/CNTN6/GLDN/ROBO4/STXBP6/DSC2 |
| GO:0019838 | growth factor binding | 10/445 | 139/18410 | 0.002051 | 0.038843 | FLT4/ESM1/HAP1/ACVRL1/TGFBR3/LRRC32/COL4A1/GHRHR/IL1R2/FGFBP2 |
| GO:0016849 | phosphorus-oxygen lyase activity | 4/445 | 23/18410 | 0.002071 | 0.038843 | GUCY1A2/ADCY8/NPR1/BST1 |
| GO:0030215 | semaphorin receptor binding | 4/445 | 23/18410 | 0.002071 | 0.038843 | SEMA5A/SEMA6A/SEMA5B/SEMA3G |

**Supplemental Table 5c GO enrichment analysis (cell component, CC) of up-regulated genes**

| **ID** | **Description** | **Gene**  **Ratio** | **Bg Ratio** | ***P*-value** | **p.adjust** | **Gene ID** |
| --- | --- | --- | --- | --- | --- | --- |
| GO:0031514 | motile cilium | 91/1029 | 227/19594 | 1.21E-56 | 5.77E-54 | NME5/AK8/TCTE1/CFAP61/IQCG/TEKT2/SPA17/DRC3/SPAG16/CCDC65/ENKUR/CETN2/DRC1/ZBBX/RSPH1/PACRG/MNS1/DNAI2/RSPH9/TEKT1/DNAI1/CFAP43/CFAP52/DRC7/AKAP3/AK7/IQCA1/TEKT3/CATSPERD/SPACA9/CFAP45/CFAP221/RSPH4A/CFAP58/CFAP73/TTC29/SPEF1/SAXO2/IFT46/TEKT4/CFAP65/SPEF2/DNALI1/TTLL9/ROPN1L/IQCD/DNAH5/DNAJB13/DNAH11/CFAP47/GAS8/NPHP1/DNAH9/ENO4/MAK/DYNC2H1/IFT88/SPAG6/TSGA10/CCDC181/CABCOCO1/CFAP69/SPAG17/FLACC1/CFAP99/CFAP100/CFAP70/CFAP300/CFAP161/DNAH2/IFT172/CCDC39/ROPN1B/IFT81/IQUB/IFT27/CCDC103/SORD/FBXL13/CFAP44/EFCAB2/KIF9/INTU/CATSPERE/TCP11X1/DNAH1/CFAP206/CABYR/SAXO1/TCP11/ROPN1 |
| GO:0005930 | axoneme | 66/1029 | 131/19594 | 3.39E-49 | 8.05E-47 | AK8/CFAP61/DNAH6/DRC3/SPAG16/DNAL1/CCDC65/EFHC2/DRC1/MNS1/DNAI2/RSPH9/RP1/DNAI1/CFAP43/CFAP52/CCDC113/CFAP45/KIF19/CFAP221/RSPH4A/CFAP73/SPEF1/SAXO2/DNALI1/DNAH5/CCDC96/DNAJB13/DNAH11/AKAP14/LCA5L/CFAP54/EFHC1/DNAH7/CCDC40/DZIP1L/GAS8/DNAH9/BBS5/MAK/DYNC2H1/CFAP74/SPAG6/DNAAF1/DNAH10/HYDIN/SPAG17/RPGRIP1L/CFAP100/CFAP70/TTC30B/DNAH2/IFT172/CCDC39/CFAP46/TRAF3IP1/TTC30A/SPATA4/CCDC103/DCDC2/DNAH3/WDR35/LCA5/DNAH1/CFAP206/SAXO1 |
| GO:0097014 | ciliary plasm | 66/1029 | 132/19594 | 6.45E-49 | 1.02E-46 | AK8/CFAP61/DNAH6/DRC3/SPAG16/DNAL1/CCDC65/EFHC2/DRC1/MNS1/DNAI2/RSPH9/RP1/DNAI1/CFAP43/CFAP52/CCDC113/CFAP45/KIF19/CFAP221/RSPH4A/CFAP73/SPEF1/SAXO2/DNALI1/DNAH5/CCDC96/DNAJB13/DNAH11/AKAP14/LCA5L/CFAP54/EFHC1/DNAH7/CCDC40/DZIP1L/GAS8/DNAH9/BBS5/MAK/DYNC2H1/CFAP74/SPAG6/DNAAF1/DNAH10/HYDIN/SPAG17/RPGRIP1L/CFAP100/CFAP70/TTC30B/DNAH2/IFT172/CCDC39/CFAP46/TRAF3IP1/TTC30A/SPATA4/CCDC103/DCDC2/DNAH3/WDR35/LCA5/DNAH1/CFAP206/SAXO1 |
| GO:0032838 | plasma membrane bounded cell projection cytoplasm | 72/1029 | 220/19594 | 6.05E-38 | 7.19E-36 | AK8/CFAP61/DNAH6/DRC3/SPAG16/DNAL1/CCDC65/EFHC2/DRC1/MNS1/DNAI2/RSPH9/RP1/DNAI1/CFAP43/CFAP52/CCDC113/CFAP45/KIF19/CFAP221/RSPH4A/CFAP73/SPEF1/SAXO2/DNALI1/DNAH5/CCDC96/DNAJB13/DNAH11/AKAP14/LCA5L/CFAP54/EFHC1/DNAH7/CCDC40/DZIP1L/GAS8/DNAH9/BBS5/MAK/DYNC2H1/CFAP74/SPAG6/DNAAF1/DNAH10/HYDIN/SPAG17/RPGRIP1L/MAP1A/CFAP100/CFAP70/TTC30B/DNAH2/IFT172/CCDC39/CFAP46/TRAF3IP1/TTC30A/SPATA4/CCDC103/DCDC2/AGBL4/DNAH3/WDR35/AP3B2/RAB27B/LCA5/KIF3A/DNAH1/GRIK2/CFAP206/SAXO1 |
| GO:0097729 | 9+2 motile cilium | 58/1029 | 149/19594 | 1.58E-35 | 1.50E-33 | NME5/AK8/TCTE1/IQCG/SPA17/DRC3/SPAG16/ENKUR/CETN2/RSPH1/PACRG/MNS1/DNAI2/RSPH9/DNAI1/CFAP43/CFAP52/AKAP3/TEKT3/CATSPERD/SPACA9/CFAP45/CFAP221/CFAP58/TTC29/SPEF1/SAXO2/TEKT4/CFAP65/SPEF2/DNALI1/DNAH5/DNAJB13/DNAH11/CFAP47/GAS8/DNAH9/ENO4/IFT88/SPAG6/CCDC181/CABCOCO1/CFAP69/FLACC1/CFAP70/DNAH2/IFT172/CCDC39/IFT81/IFT27/EFCAB2/KIF9/CATSPERE/TCP11X1/DNAH1/CABYR/SAXO1/TCP11 |
| GO:0099568 | cytoplasmic region | 73/1029 | 259/19594 | 1.32E-33 | 1.05E-31 | AK8/CFAP61/DNAH6/DRC3/SPAG16/DNAL1/CCDC65/EFHC2/DRC1/MNS1/DNAI2/RSPH9/RP1/DNAI1/CFAP43/CFAP52/CCDC113/CFAP45/KIF19/CFAP221/RSPH4A/CFAP73/SPEF1/SAXO2/DNALI1/DNAH5/CCDC96/DNAJB13/DNAH11/AKAP14/LCA5L/CFAP54/EFHC1/DNAH7/CCDC40/DZIP1L/GAS8/DNAH9/BBS5/MAK/DYNC2H1/CFAP74/SPAG6/DNAAF1/DNAH10/HYDIN/SPAG17/RPGRIP1L/MAP1A/CFAP100/CFAP70/TTC30B/DNAH2/IFT172/CCDC39/CFAP46/TRAF3IP1/TTC30A/SPATA4/CCDC103/DCDC2/AGBL4/DNAH3/WDR35/AP3B2/RAB27B/LCA5/KIF3A/DNAH1/GRIK2/CFAP206/HAMP/SAXO1 |
| GO:0036126 | sperm flagellum | 47/1029 | 136/19594 | 2.49E-26 | 1.69E-24 | NME5/AK8/TCTE1/IQCG/SPA17/DRC3/SPAG16/ENKUR/RSPH1/PACRG/MNS1/DNAI2/AKAP3/TEKT3/CATSPERD/SPACA9/CFAP221/CFAP58/TTC29/SAXO2/TEKT4/CFAP65/SPEF2/DNALI1/DNAJB13/CFAP47/GAS8/ENO4/IFT88/SPAG6/CCDC181/CABCOCO1/CFAP69/FLACC1/CFAP70/DNAH2/IFT172/IFT81/IFT27/EFCAB2/KIF9/CATSPERE/TCP11X1/DNAH1/CABYR/SAXO1/TCP11 |
| GO:0036064 | ciliary basal body | 50/1029 | 161/19594 | 1.72E-25 | 1.02E-23 | CFAP126/CCDC65/GAS2L2/CETN2/EFHC2/AGBL2/SPACA9/CCDC113/PIFO/TTC26/SAXO2/IFT46/TTLL6/TTLL9/IQCD/FANK1/CCDC96/WHRN/PPP1R32/CFAP157/DZIP1L/GAS8/BBS5/IFT88/RABL2B/BBOF1/RPGRIP1L/MLF1/C5orf49/CFAP100/CCDC170/MAPK15/CFAP70/B9D1/IFT172/C11orf97/IFT81/TRAF3IP1/CEP19/CEP41/B9D2/FAM161A/AGBL4/INTU/WDR35/LCA5/CFAP206/USH1G/SAXO1/CCDC178 |
| GO:0030286 | dynein complex | 21/1029 | 54/19594 | 1.09E-13 | 5.74E-12 | DNAH6/DNAL1/CCDC65/DRC1/DNAI2/DNAI1/DNALI1/DNAL4/DNAH5/DNAH11/DYNLRB2/DNAH7/DNAH9/DYNC2H1/DNAH12/DNAH10/CFAP70/DNAH2/CCDC103/DNAH3/DNAH1 |
| GO:0005874 | microtubule | 63/1029 | 435/19594 | 2.19E-13 | 1.04E-11 | TEKT2/DNAH6/DNAL1/GAS2L2/EFHC2/DNAI2/TUBA4B/TEKT1/MAPRE3/RP1/DNAI1/KIF24/MAP6/TOGARAM2/SPACA9/KIF19/SPEF1/SAXO2/C4orf47/DCDC2B/TTLL6/TTLL9/DNAL4/DNAH5/DCDC1/DNAH11/TRPV4/KIF21A/DYNLRB2/KIF6/DNAH7/GAS8/DNAH9/DYNC2H1/SPAG6/CCDC181/DNAH12/DNAH10/SPAG17/RPGRIP1L/MAP1A/MDM1/TTC30B/DNAH2/TPPP3/NEK2/REEP2/TTC30A/DCDC2/EML6/FAM161A/TUBB4B/DNAH3/KATNAL2/KIF9/HSPH1/KIF3A/DNAH1/KIF27/CFAP206/KIF26B/SAXO1/MTUS2 |
| GO:0005858 | axonemal dynein complex | 14/1029 | 23/19594 | 5.84E-13 | 2.52E-11 | DNAH6/DNAL1/CCDC65/DRC1/DNAI2/DNAI1/DNAH5/DNAH7/DNAH9/CFAP70/DNAH2/CCDC103/DNAH3/DNAH1 |
| GO:0030990 | intraciliary transport particle | 13/1029 | 24/19594 | 3.12E-11 | 1.23E-09 | TTC26/IFT46/IFT22/IFT88/TTC21A/CLUAP1/TTC30B/IFT172/IFT81/TRAF3IP1/IFT27/TTC30A/WDR35 |
| GO:0030992 | intraciliary transport particle B | 11/1029 | 17/19594 | 7.36E-11 | 2.69E-09 | TTC26/IFT46/IFT22/IFT88/CLUAP1/TTC30B/IFT172/IFT81/TRAF3IP1/IFT27/TTC30A |
| GO:0035869 | ciliary transition zone | 20/1029 | 69/19594 | 2.10E-10 | 7.14E-09 | CETN2/RP1/TMEM231/WHRN/TMEM67/NPHP1/TMEM107/MAK/RPGRIP1L/B9D1/TRAF3IP1/TCTN1/CC2D2A/TCTN2/B9D2/UNC119B/FAM161A/TMEM17/LCA5/USH1G |
| GO:0120293 | dynein axonemal particle | 11/1029 | 20/19594 | 8.62E-10 | 2.73E-08 | DNAI2/DNAI1/DNAAF3/DNALI1/ZMYND10/SPAG1/RUVBL1/DNAAF4/RUVBL2/NME9/DNAAF2 |
| GO:0097542 | ciliary tip | 16/1029 | 47/19594 | 9.61E-10 | 2.85E-08 | RP1/TTC26/SPEF1/IFT46/IFT22/DYNLRB2/DYNC2H1/IFT88/CLUAP1/TTC30B/IFT172/IFT81/TRAF3IP1/IFT27/WDR35/KIF3A |
| GO:0005875 | microtubule associated complex | 30/1029 | 160/19594 | 1.03E-09 | 2.88E-08 | DNAH6/DNAL1/CCDC65/DRC1/DNAI2/RP1/DNAI1/KIF19/DNALI1/DNAL4/DNAH5/DNAH11/KIF21A/DYNLRB2/KIF6/DNAH7/DNAH9/DYNC2H1/DNAH12/DNAH10/MAP1A/CFAP70/DNAH2/CCDC103/DNAH3/KIF9/KIF3A/DNAH1/KIF27/KIF26B |
| GO:0036038 | MKS complex | 9/1029 | 13/19594 | 1.73E-09 | 4.58E-08 | TMEM231/TMEM67/TMEM107/B9D1/TCTN1/CC2D2A/TCTN2/B9D2/TMEM17 |
| GO:0002177 | manchette | 9/1029 | 15/19594 | 1.10E-08 | 2.76E-07 | IQCG/PACRG/SPEF2/C9orf24/CCDC181/LRGUK/PPP1R42/MEIG1/STRBP |
| GO:0097228 | sperm principal piece | 11/1029 | 29/19594 | 1.15E-07 | 2.72E-06 | SPA17/ENKUR/AKAP3/CATSPERD/ENO4/SPAG6/IFT172/IFT81/IFT27/EFCAB2/CATSPERE |
| GO:0036157 | outer dynein arm | 7/1029 | 11/19594 | 2.96E-07 | 6.69E-06 | DNAL1/DNAI2/DNAI1/DNAH5/DNAH9/CFAP70/CCDC103 |
| GO:0097730 | non-motile cilium | 24/1029 | 166/19594 | 6.32E-06 | 0.000137 | CETN2/RSPH9/RP1/C4orf47/TTLL6/WHRN/DNAAF4/NPHP1/MAK/IFT88/CERKL/CFAP69/RPGRIP1L/IFTAP/DCDC2/FAM161A/PROM1/LCA5/USH1G/MCHR1/TULP1/CNGA1/DRD1/USH1C |
| GO:0097225 | sperm midpiece | 10/1029 | 38/19594 | 1.88E-05 | 0.000388 | PACRG/AKAP3/CFAP58/CFAP65/SPEF2/CFAP69/IFT172/IFT81/IFT27/TCP11 |
| GO:0005881 | cytoplasmic microtubule | 14/1029 | 75/19594 | 3.10E-05 | 0.000613 | MAPRE3/TOGARAM2/SPACA9/SAXO2/C4orf47/TRPV4/RPGRIP1L/TTC30B/REEP2/TTC30A/FAM161A/CFAP206/SAXO1/MTUS2 |
| GO:0097546 | ciliary base | 10/1029 | 42/19594 | 4.82E-05 | 0.000917 | SPACA9/TTC26/DNALI1/FAM183A/FANK1/IFT88/MOK/C11orf97/TRAF3IP1/CEP126 |
| GO:0045177 | apical part of cell | 42/1029 | 424/19594 | 6.15E-05 | 0.001124 | SPTBN2/CFAP126/CETN2/CEACAM5/PROM2/AQP6/EPB41L4B/SPEF1/ZMYND10/TRPV4/SLC44A4/DYNC2H1/SLC22A4/OXTR/AQP5/SLC23A1/CLDN1/CDH2/ECRG4/FZD3/SCNN1G/LRP2/PROM1/SLC34A3/RAB27B/MUC20/UPK2/ANXA13/ATP4B/P2RY6/SLC4A5/F2RL2/SLC15A1/KCNK2/HAMP/USH1C/ABCB1/SLC4A11/ATP12A/EDAR/ATP6V1B1/AJAP1 |
| GO:0005814 | centriole | 20/1029 | 147/19594 | 8.76E-05 | 0.00154 | CETN2/CCDC146/KIF24/AGBL2/CCDC78/SAXO2/DEUP1/DZIP1L/CEP83/IFT88/RABL2B/TSGA10/MDM1/MAPK15/CEP19/CEP41/AGBL4/WDR90/KIF3A/SAXO1 |
| GO:0016324 | apical plasma membrane | 36/1029 | 358/19594 | 0.000149 | 0.002536 | SPTBN2/CFAP126/CEACAM5/PROM2/AQP6/SPEF1/ZMYND10/TRPV4/SLC44A4/SLC22A4/OXTR/AQP5/SLC23A1/CLDN1/CDH2/ECRG4/FZD3/SCNN1G/LRP2/PROM1/SLC34A3/RAB27B/MUC20/UPK2/ANXA13/ATP4B/P2RY6/SLC4A5/F2RL2/SLC15A1/KCNK2/ABCB1/SLC4A11/ATP12A/ATP6V1B1/AJAP1 |
| GO:0032391 | photoreceptor connecting cilium | 9/1029 | 41/19594 | 0.000224 | 0.003673 | CETN2/RP1/WHRN/NPHP1/MAK/RPGRIP1L/FAM161A/LCA5/USH1G |
| GO:0098878 | neurotransmitter receptor complex | 9/1029 | 45/19594 | 0.00047 | 0.007437 | GRIN3B/GRIK5/GRIN1/CNIH2/SHISA6/GRIK2/HTR3E/VWC2L/GRIA2 |
| GO:0097731 | 9+0 non-motile cilium | 17/1029 | 132/19594 | 0.000554 | 0.008485 | CETN2/RP1/C4orf47/TTLL6/WHRN/NPHP1/MAK/CERKL/RPGRIP1L/IFTAP/FAM161A/PROM1/LCA5/USH1G/TULP1/CNGA1/USH1C |
| GO:0009925 | basal plasma membrane | 26/1029 | 251/19594 | 0.000761 | 0.011219 | CEACAM5/PROM2/SPEF1/VSIG1/KCNJ16/OSCP1/MET/AQP5/SLC23A1/FRMPD2/CLDN8/CLDN1/CDH2/ERBB4/SLC16A12/MUC20/ADRA2A/MTTP/FRMPD2B/P2RY6/SLC4A11/ATP12A/ALPK2/ATP6V1B1/AJAP1/LPO |
| GO:0001669 | acrosomal vesicle | 16/1029 | 124/19594 | 0.000779 | 0.011219 | ENKUR/MORN2/AKAP3/TEKT3/SPAG8/SPACA9/CFAP65/SPAG6/RND2/LRGUK/PCSK4/IQUB/SYT8/TCP11X1/TMEM190/TCP11 |
| GO:0008328 | ionotropic glutamate receptor complex | 8/1029 | 40/19594 | 0.000964 | 0.013469 | GRIN3B/GRIK5/GRIN1/CNIH2/SHISA6/GRIK2/VWC2L/GRIA2 |
| GO:0001917 | photoreceptor inner segment | 10/1029 | 64/19594 | 0.001745 | 0.022818 | RP1/REEP6/WHRN/MAK/CERKL/FAM161A/USH1G/TULP1/USH1C/RDH12 |
| GO:0034702 | ion channel complex | 28/1029 | 294/19594 | 0.00177 | 0.022818 | CLIC6/GRIN3B/CNGA4/CATSPERD/GLRB/KCNJ16/GRIK5/GRIN1/CACNG6/CLCNKA/BEST4/HCN4/CNIH2/CNGA3/CATSPERE/TRPV6/SCNN1G/SHISA6/GRIK2/CACNA1G/LRRC26/HTR3E/GABRP/CNGA1/KCNK2/VWC2L/GABRB3/GRIA2 |
| GO:0060170 | ciliary membrane | 11/1029 | 75/19594 | 0.001777 | 0.022818 | CNGA4/TMEM231/PROM2/TMEM67/BBS5/TCTN2/TMEM17/PROM1/MCHR1/CNGA1/DRD1 |
| GO:0045178 | basal part of cell | 26/1029 | 269/19594 | 0.002064 | 0.025802 | CEACAM5/PROM2/SPEF1/VSIG1/KCNJ16/OSCP1/MET/AQP5/SLC23A1/FRMPD2/CLDN8/CLDN1/CDH2/ERBB4/SLC16A12/MUC20/ADRA2A/MTTP/FRMPD2B/P2RY6/SLC4A11/ATP12A/ALPK2/ATP6V1B1/AJAP1/LPO |
| GO:0005902 | microvillus | 12/1029 | 90/19594 | 0.002574 | 0.031355 | FOXA1/PROM2/SPEF1/ESPN/FMN2/OXTR/AQP5/PROM1/MUC20/MTTP/USH1C/ATP6V1B1 |
| GO:0034703 | cation channel complex | 22/1029 | 221/19594 | 0.003064 | 0.03639 | GRIN3B/CNGA4/CATSPERD/KCNJ16/GRIK5/GRIN1/CACNG6/HCN4/CNIH2/CNGA3/CATSPERE/TRPV6/SCNN1G/SHISA6/GRIK2/CACNA1G/LRRC26/HTR3E/CNGA1/KCNK2/VWC2L/GRIA2 |
| GO:0016323 | basolateral plasma membrane | 22/1029 | 226/19594 | 0.004016 | 0.046526 | CEACAM5/PROM2/SPEF1/VSIG1/KCNJ16/FRMPD2/CLDN8/CLDN1/CDH2/ERBB4/SLC16A12/MUC20/ADRA2A/MTTP/FRMPD2B/P2RY6/SLC4A11/ATP12A/ALPK2/ATP6V1B1/AJAP1/LPO |

**Supplemental Table 5d GO enrichment analysis (cell component, CC) of down-regulated genes**

| **ID** | **Description** | **Gene**  **Ratio** | **Bg Ratio** | ***P*-value** | **p.adjust** | **Gene ID** |
| --- | --- | --- | --- | --- | --- | --- |
| GO:0032039 | integrator complex | 9/468 | 27/19594 | 7.49E-09 | 2.69E-06 | CT45A8/CT45A7/CT45A2/CT45A3/CT45A9/CT45A6/CT45A5/CT45A1/CT45A10 |
| GO:0009897 | external side of plasma membrane | 33/468 | 455/19594 | 1.68E-08 | 3.02E-06 | ADGRE1/IL1RL1/SPN/HEG1/ITGA1/KLRD1/IL7R/P2RX7/FCGR3A/CXCR2/TLR8/CSF2RB/TGFBR3/MSR1/TRGC1/CDH5/PRLR/THBD/CLEC4E/TRGV9/BTNL9/ACE/TNF/FOLR3/ICAM1/FCRL6/TRGV4/CXCR1/CA4/BTNL8/CLEC4D/CX3CR1/CLEC4M |
| GO:0005581 | collagen trimer | 13/468 | 86/19594 | 1.24E-07 | 1.49E-05 | COL4A4/COLEC10/COL4A3/COL4A2/COL12A1/GLDN/C1QA/COL26A1/MSR1/SFTPA1/FCN3/COL4A1/COL22A1 |
| GO:0070820 | tertiary granule | 17/468 | 164/19594 | 4.27E-07 | 3.83E-05 | FPR2/MCEMP1/SIGLEC5/SLC11A1/SLC2A3/ADGRE3/PTPRB/FPR1/CFP/OLR1/CAMP/OSCAR/LILRB2/FOLR3/CLEC4D/ORM1/HP |
| GO:0042581 | specific granule | 16/468 | 160/19594 | 1.51E-06 | 0.000108 | FPR2/MCEMP1/SLC2A3/PTPRB/BST1/CFP/OLR1/CEACAM3/CAMP/MMP25/OSCAR/FOLR3/ARG1/CLEC4D/ORM1/HP |
| GO:0030667 | secretory granule membrane | 22/468 | 312/19594 | 6.65E-06 | 0.000398 | SIRPB1/FPR2/MCEMP1/SIGLEC5/SLC11A1/SLC2A3/ADGRE3/PTPRB/CXCR2/ABCA3/FPR1/PLA1A/BST1/OLR1/LAMP3/CEACAM3/MMP25/LILRB2/CXCR1/CA4/CLEC4D/LILRB3 |
| GO:0044194 | cytolytic granule | 5/468 | 13/19594 | 8.36E-06 | 0.000429 | PRF1/GNLY/GZMB/GZMH/NKG7 |
| GO:0042599 | lamellar body | 5/468 | 17/19594 | 3.71E-05 | 0.001666 | NAPSA/SFTPC/ABCA3/LAMP3/SFTPA1 |
| GO:0070821 | tertiary granule membrane | 9/468 | 73/19594 | 5.87E-05 | 0.00234 | FPR2/MCEMP1/SIGLEC5/SLC11A1/SLC2A3/PTPRB/OLR1/LILRB2/CLEC4D |
| GO:0045178 | basal part of cell | 18/468 | 269/19594 | 8.92E-05 | 0.00316 | CADM1/ENPP1/SHROOM4/HPGD/GKN2/SLC39A8/LIN7A/ITGA1/DISP1/NDRG4/SLC22A8/ADCY8/P2RY1/EDN1/ACE/TEK/CDH16/OTOF |
| GO:0101003 | ficolin-1-rich granule membrane | 8/468 | 61/19594 | 9.68E-05 | 0.00316 | FPR2/SIGLEC5/SLC11A1/SLC2A3/ADGRE3/FPR1/LILRB2/CLEC4D |
| GO:0062023 | collagen-containing extracellular matrix | 23/468 | 429/19594 | 0.000285 | 0.008516 | LAMA3/LAMC3/COL4A4/COL4A3/COL4A2/COL12A1/TNXB/C1QA/FIBCD1/COL26A1/MATN3/ANGPTL7/CFP/ANGPT4/S100A8/TNR/P3H2/FCN3/COL4A1/ANGPTL6/ICAM1/SERPINE1/ORM1 |
| GO:0035579 | specific granule membrane | 9/468 | 91/19594 | 0.000325 | 0.008963 | FPR2/MCEMP1/SLC2A3/PTPRB/BST1/OLR1/CEACAM3/MMP25/CLEC4D |
| GO:0098793 | presynapse | 25/468 | 492/19594 | 0.000353 | 0.009044 | STX11/RIMS4/SEPTIN4/PPFIBP1/SH3GL3/LIN7A/GPM6A/CNTN6/HAP1/WNT7A/KCNA3/CABP4/GRIA1/ADCY8/P2RX7/LGI3/P2RY1/RPH3A/SLC6A4/SYN2/GPER1/LRRK2/CHRM1/OTOF/SLC6A12 |
| GO:0048786 | presynaptic active zone | 8/468 | 75/19594 | 0.000415 | 0.009924 | STX11/RIMS4/PPFIBP1/GPM6A/ADCY8/P2RY1/GPER1/OTOF |
| GO:0035580 | specific granule lumen | 7/468 | 62/19594 | 0.000671 | 0.013891 | CFP/CAMP/OSCAR/FOLR3/ARG1/ORM1/HP |
| GO:0097060 | synaptic membrane | 20/468 | 373/19594 | 0.000696 | 0.013891 | STX11/LRP4/RIMS4/RGS9/LIN7A/GPM6A/CNTN6/KCNA3/GRIA1/ARC/ADCY8/P2RY1/RPH3A/SLC6A4/GPER1/CHRM1/DDN/LRRTM4/OTOF/IQSEC3 |
| GO:0005771 | multivesicular body | 7/468 | 63/19594 | 0.00074 | 0.013891 | NAPSA/SFTPC/ABCA3/SFTPA1/CST7/LRRK2/NSG2 |
| GO:0061702 | inflammasome complex | 4/468 | 18/19594 | 0.000753 | 0.013891 | CASP12/NLRC4/MEFV/CASP5 |
| GO:0031225 | anchored component of membrane | 12/468 | 169/19594 | 0.000774 | 0.013891 | DPEP3/DPEP2/CNTN6/LYPD5/BST1/MMP25/CD52/FOLR3/XPNPEP2/DPEP1/CA4/ALPL |
| GO:0005912 | adherens junction | 12/468 | 172/19594 | 0.000903 | 0.015437 | CDH19/SHROOM1/SHROOM4/LIN7A/SYNM/ALOX15B/JCAD/CCDC85A/TMEM204/CDH5/STXBP6/DSC2 |
| GO:0009925 | basal plasma membrane | 15/468 | 251/19594 | 0.00109 | 0.017784 | CADM1/ENPP1/SHROOM4/HPGD/SLC39A8/LIN7A/DISP1/NDRG4/SLC22A8/ADCY8/P2RY1/ACE/TEK/CDH16/OTOF |
| GO:0098644 | complex of collagen trimers | 4/468 | 22/19594 | 0.00167 | 0.025256 | COL4A4/COL4A3/COL4A2/COL4A1 |
| GO:0101002 | ficolin-1-rich granule | 12/468 | 185/19594 | 0.001688 | 0.025256 | FPR2/BIN2/SIGLEC5/HK3/SLC11A1/SLC2A3/ADGRE3/FPR1/CRISPLD2/MNDA/LILRB2/CLEC4D |
| GO:0098889 | intrinsic component of presynaptic membrane | 7/468 | 74/19594 | 0.001922 | 0.025711 | GPM6A/CNTN6/KCNA3/ADCY8/P2RY1/SLC6A4/CHRM1 |
| GO:1904724 | tertiary granule lumen | 6/468 | 55/19594 | 0.001937 | 0.025711 | CFP/CAMP/OSCAR/FOLR3/ORM1/HP |
| GO:0034774 | secretory granule lumen | 17/468 | 322/19594 | 0.002013 | 0.025711 | BIN2/HK3/UNC13D/FGR/S100A12/CFP/S100A8/CRISPLD2/CAMP/MNDA/OSCAR/FOLR3/ARG1/SERPINE1/ORM1/HP/PRSS57 |
| GO:0005911 | cell-cell junction | 23/468 | 497/19594 | 0.002058 | 0.025711 | CDH19/CADM1/SHROOM1/PDZD2/SHROOM4/HEG1/LIN7A/CLDN18/SYNM/GJA5/P2RX7/CLDN5/ALOX15B/JCAD/MPP3/CCDC85A/TMEM204/CDH5/ILDR2/STXBP6/DSC2/PCDH12/TEK |
| GO:0060205 | cytoplasmic vesicle lumen | 17/468 | 325/19594 | 0.002218 | 0.025711 | BIN2/HK3/UNC13D/FGR/S100A12/CFP/S100A8/CRISPLD2/CAMP/MNDA/OSCAR/FOLR3/ARG1/SERPINE1/ORM1/HP/PRSS57 |
| GO:0045121 | membrane raft | 17/468 | 326/19594 | 0.00229 | 0.025711 | EDNRB/LRP4/PAG1/ITGA1/SELPLG/EMP2/KCNA3/ARC/ADCY8/NOS1/OLR1/TNR/SLC6A4/LRRK2/TNF/ICAM1/TEK |
| GO:0042734 | presynaptic membrane | 10/468 | 143/19594 | 0.002324 | 0.025711 | STX11/GPM6A/CNTN6/KCNA3/ADCY8/P2RY1/SLC6A4/GPER1/CHRM1/OTOF |
| GO:0031983 | vesicle lumen | 17/468 | 327/19594 | 0.002363 | 0.025711 | BIN2/HK3/UNC13D/FGR/S100A12/CFP/S100A8/CRISPLD2/CAMP/MNDA/OSCAR/FOLR3/ARG1/SERPINE1/ORM1/HP/PRSS57 |
| GO:0098857 | membrane microdomain | 17/468 | 327/19594 | 0.002363 | 0.025711 | EDNRB/LRP4/PAG1/ITGA1/SELPLG/EMP2/KCNA3/ARC/ADCY8/NOS1/OLR1/TNR/SLC6A4/LRRK2/TNF/ICAM1/TEK |
| GO:0036379 | myofilament | 4/468 | 25/19594 | 0.002729 | 0.028818 | TNNC1/MYBPC3/MYBPHL/TNNT1 |
| GO:0016323 | basolateral plasma membrane | 13/468 | 226/19594 | 0.003183 | 0.031428 | CADM1/ENPP1/HPGD/SLC39A8/LIN7A/DISP1/NDRG4/SLC22A8/ADCY8/P2RY1/TEK/CDH16/OTOF |
| GO:0001931 | uropod | 3/468 | 13/19594 | 0.003239 | 0.031428 | SPN/SELPLG/BST1 |
| GO:0031254 | cell trailing edge | 3/468 | 13/19594 | 0.003239 | 0.031428 | SPN/SELPLG/BST1 |
| GO:0001891 | phagocytic cup | 4/468 | 28/19594 | 0.004175 | 0.039446 | BIN2/MYO1G/CLEC4E/TNF |
| GO:0098978 | glutamatergic synapse | 16/468 | 319/19594 | 0.004431 | 0.040786 | SH3GL3/RGS9/GPM6A/WASF3/NRG3/WNT7A/KCNA3/GRIA1/ARC/ADCY8/P2RY1/TNR/SLITRK2/SYN2/LRRK2/CHRM1 |
| GO:0048787 | presynaptic active zone membrane | 4/468 | 29/19594 | 0.004753 | 0.042662 | STX11/GPM6A/P2RY1/OTOF |
| GO:0032591 | dendritic spine membrane | 3/468 | 15/19594 | 0.004974 | 0.043549 | GRIA1/GPER1/DDN |
| GO:0099056 | integral component of presynaptic membrane | 6/468 | 67/19594 | 0.005249 | 0.044864 | GPM6A/KCNA3/ADCY8/P2RY1/SLC6A4/CHRM1 |
| GO:0005902 | microvillus | 7/468 | 90/19594 | 0.00577 | 0.048173 | MYO7B/SPN/MYO1G/LRRK2/TEK/DPEP1/ENPP7 |
