# Supplementary material for: Integrative analysis of bulk and single-cell RNA sequencing reveals the gene expression profile and the critical signaling pathways of type II CPAM
Source: Cell Biosci. 2024 Jul 18;14:94. doi: 10.1186/s13578-024-01276-8 (PMC11264590; doi:10.1186/s13578-024-01276-8)
Supplement: Supplementary file 18 — Supplementary Material 18: Supplemental Table 12 Ligands and receptors enriched in PI3K-AKT signaling pathway and epithelial to mesenchymal transition. [file 13578_2024_1276_MOESM18_ESM.docx]

| **Supplemental Table 6. KEGG analysis of differentially expressed genes** | | | | | | |
| --- | --- | --- | --- | --- | --- | --- |
| **ID** | **Description** | **Set**  **Size** | **Enrichment**  **Score** | ***P*-value** | **Adjusted*P*-value** | **Gene** |
| hsa04080 | Neuroactive ligand-receptor interaction | 269 | 0.620613 | 6.26E-16 | 2.10E-13 | HTR2C/SST/UCN3/GRM4/NPBWR1/CRH/NMBR/GALR2/CHRNA1/UCN2/CCKBR/OXTR/GPR156/GRIN3B/GIPR/MCHR1/RLN2/GABRP/RXFP3/GRIK2/ADRA2A/SLURP2/GLRB/BDKRB1/GRIA2/AVPR1A/LPAR3/GRIK5/AGTR2/GLRA2/GABRB3/GHRL/PENK/BDKRB2/GRM7/GRIN1/F2RL2/GALR1/GLRA3/DRD1/GRM5/ADRA1B/MAS1/OPRL1/OXT/CALCB/P2RY6/LYPD6B/ADORA2B/CORT/NMUR2/LPAR4/CALCA/TACR1/NTS/TSHR/DRD5/F2RL1/GPR83/P2RX3/P2RY2/PLG/GPR35/QRFP/VIPR2/CTSG/ADRA2B/ADM2/GABRG3/HTR2A/GABRQ/CHRNA4/PTGER2/NMB/PTGFR/PNOC/RLN1/DRD2/ADCYAP1/P2RX6/LHB/GABRA2/C5AR1/UCN/DRD4/PYY/NPFFR1/LTB4R2/PGRMC1 |
| hsa04814 | Motor proteins | 177 | 0.642325 | 1.36E-12 | 2.28E-10 | DNAI2/DNAH5/DNAH7/DNAI1/DNAH9/DNAH12/KIF19/DNAH6/DYNLRB2/TNNI3/DNAH10/DNAH11/DNAH3/DNAH2/KIF24/MYH1/KIF6/KIF21A/DNALI1/MYH2/MYLPF/TNNT3/DYNC2H1/DNAL1/DNAH1/TUBB8/BICDL2/KIF9/TUBAL3/TUBB4B/KIF27/KIF26B/TUBB3/DNAL4/KIF3A/KIF1A/MYO16/ACTG2/DNAH14/MYH15/DNAH8/KIF2A/TUBB4A/MYO18B/KIF12/MYO1E/DYNLL1/KIF20A/MYO3B/MYH13/KIF17/KIFAP3/DYNC2LI1/DYNLT1/MYO7A/KIF18B/MYO3A/KIF3B/KIF2C |
| hsa00980 | Metabolism of xenobiotics by cytochrome P450 | 62 | 0.746756 | 1.41E-09 | 1.58E-07 | UGT1A6/UGT2A1/ADH7/UGT2B17/CYP2F1/CYP2A13/GSTA3/UGT2B15/GSTA1/GSTA2/UGT1A7/ALDH3B2/ADH6/ALDH3A1/HSD11B1L/ADH1C/ALDH3B1/UGT1A1/AKR7A3/CYP2S1/AKR1C1/AKR7L/MGST1/CYP2A6/CYP2A7/CYP1B1/UGT2B7/GSTM2/CYP2B6/AKR7A2/GSTO2 |
| hsa03010 | Ribosome | 135 | 0.634732 | 6.00E-09 | 3.49E-07 | DNAI2/DNAH5/DNAH7/DNAI1/DNAH9/DNAH12/DNAH6/DNAH10/DNAH11/DNAH3/DNAH2/GRIA2/DNALI1/GRIN1/COX6B2/DNAL1/GRM5/MAPK10/DNAH1/TUBB8/TUBAL3/TUBB4B/TUBB3/DNAL4/IFT57/GPX2/NDUFC2-KCTD14/POLR2I/DNAH14/CREB3L4/DNAH8/TUBB4A/TAF4B/CACNA1B/CASP9/PPIF/GPX8/STX1A/SOD1/VDAC3/NDUFAB1/TUBA3D/NRF1/TUBA1A/COX7A2L/SLC1A2/COX7C/NDUFA8/SEM1/NDUFB3/SLC25A4/CLTA/NDUFA4/NDUFC1/NDUFB5/RB1CC1/SIN3A/GPX7/TBPL1/COX6B1/NDUFB7/HDAC1/POLR2J/ATP5PO/NDUFA5/ATP5F1A/UQCRC1/COX5B/UQCRB/COX5A/SDHC/PSMB5/ATP5PF/KLC3/UQCR11/NDUFS6/DLG4/HDAC2/WIPI1/NDUFA1/ACTR1B/TFAM/POLR2B/POLR2F/PSMB7/GRIA3/NDUFA10/NDUFA13/ATP5MC1/NDUFB4/COX7B/ATP5MC2/UQCRC2/UQCRH/TP53/SDHA/NDUFB8/ATP5F1D/PSMA5/UQCRFS1/COX6A1/KLC4/POLR2H/NDUFB1/ATP5PB/NDUFS7/NDUFA2/PSMD4/ACTR10/UQCR10/NDUFA7/DCTN3/PSMC4/SDHB/ATP5F1B/COX6C/CREB3/PSMC6/NDUFB9/NDUFB10/WIPI2/NDUFB6/SLC25A6/COX8A/MAPK8/SLC1A3/NDUFS1/CASP3/ATP5PD/TRAF2/PSMD14/CREB3L2/SDHD/POLR2E/UQCRQ/COX7A2/CLTC/NDUFS4/NDUFS2/COX4I1/ATP5MC3/AP2B1/NDUFS3/POLR2D/NDUFV3/ULK2/VDAC1/BECN1/ATG2B/NDUFB2/PSMB1/CYC1/ATG14 |
| hsa04060 | Cytokine-cytokine receptor interaction | 244 | 0.55647 | 4.52E-09 | 3.49E-07 | NXF2B/DNAI2/DNAH5/DNAH7/DNAI1/DNAH9/DNAH12/DNAH6/NRG4/NXF2/DNAH10/DNAH11/DNAH3/DNAH2/GRIA2/DNALI1/ERBB4/GRIN1/PFN2/COX6B2/DNAL1/DNAH1/TUBB8/TUBAL3/TUBB4B/TUBB3/DNAL4/NRG2/GPX2/NDUFC2-KCTD14/DNAH14/DNAH8/C9orf72/TUBB4A/CASP9/GPX8/PFN4/CHCHD10/NXT2/NDC1/SOD1/MAP1LC3A/NDUFAB1/NRG1/TUBA3D/MCU/TUBA1A/GLE1/NUP37/ANXA11/COX7A2L/NUP155/RAE1/SLC1A2/COX7C/NDUFA8/PRPH/SEM1/NDUFB3/NDUFA4/HNRNPA1/NDUFC1/ATXN2/NDUFB5/RB1CC1/MAP2K3/GPX7/COX6B1/NUP62/NDUFB7/ATP5PO/NDUFA5/HNRNPA1L2/ATP5F1A/UQCRC1/COX5B/UQCRB/MAP1LC3B2/COX5A/SDHC/PSMB5/ATP5PF/KLC3/UQCR11/NDUFS6/WIPI1/NDUFA1/ACTR1B/NUP133/ANG/ANXA7/HSPA5/CHMP2B/PSMB7/NDUFA10/NUP35/NDUFA13/ATP5MC1/NDUFB4/COX7B/BCL2/ATP5MC2/UQCRC2/UQCRH/TP53/SDHA/NDUFB8/ATP5F1D/NUP88/PSMA5/UQCRFS1/NUP107/COX6A1/KLC4/NDUFB1/ATP5PB/NDUFS7/NUP54/MAP2K6/VCP/NDUFA2/PSMD4/ACTR10/UQCR10/NDUFA7/SIGMAR1/DCTN3/MATR3/PSMC4/XBP1/SDHB/ATP5F1B/COX6C/PSMC6/NDUFB9/NUP153/NDUFB10/WIPI2/NDUFB6/COX8A/SRSF7/NUP93/NDUFS1/BAD/CASP3/ATP5PD/TRAF2/ALS2/PSMD14/TOMM40/RANBP2/NUP85/SDHD/SPG11/UQCRQ/COX7A2/NDUFS4/ALYREF/NDUFS2/COX4I1/ATP5MC3/NDUFS3/NDUFV3/ULK2/UBQLN2/VDAC1/BECN1/ATG2B/NDUFB2/PSMB1/HNRNPA3/CYC1/ATG14 |
| hsa05014 | Amyotrophic lateral sclerosis | 338 | 0.510922 | 6.25E-09 | 3.49E-07 | BMP15/CCL15/BMPR1B/IFNE/CCL11/CXCL6/IL5RA/IL13RA2/INHBB/TNFSF11/GDF15/AMHR2/GDF7/CXCL13/IL7/CXCL1/IL11/IL12A/GDF9/CCR6/CXCL8/GDF6/EDAR/CCR8/EDA2R/TNFRSF13B/IL23R/NGFR/CXCR5/CCL14/CCL13/IL20RA/IL22RA1/CRLF2/IL12B/LIF/IL10/CCL19/ACKR3/BMP4/IL1RL2/TNFRSF13C/CXCL17/TNFRSF17/BMP7/IL31RA/CCL28/CXCL14/CCL16/CNTF/BMP8B/NGF/GDF1/ACVR1B/BMP3/IL17RB/CCL27 |
| hsa05171 | Coronavirus disease - COVID-19 | 208 | 0.557028 | 9.69E-09 | 4.64E-07 | RPL17-C18orf32/RPL36A-HNRNPH2/RPLP0/RPS5/RPL22L1/RPS8/RPSA/MRPS9/RPS6/RPS3A/RPS19/RPL17/RPS4X/MRPS6/RPL34/RPL13A/RPL10A/RPL12/RPL32/RPL26/RPL35A/RPL4/RPS18/RPL41/RPS10/RPLP1/RPL13/RPL38/RPL29/RPL31/RPS17/RPS24/RPL39/RPL5/RPS3/RPS9/RPS15A/RPL36A/RPL27A/RPS27A/RPL7A/RPL3/RPL23/RPS7/RPS14/RPL6/RPS27/RPL8/RPS25/RPL7/RPL19/RPL36/MRPL17/RPL21/RPS21/RPL23A/RPS11/RPS2/RPL14/RPS15/RPL15/RPL11/RPL24/MRPL21/RPL18A/RPL37A/RPL18/MRPL18/RPS10-NUDT3/MRPL13/RPS13/RPS12/RPL35/RPS20/RPS29/RPS16/RPL37/RPL28/RPL22/RPS23/RPL26L1/MRPL3/RPL30/RPL10/MRPL35/MRPS16/MRPL12/MRPL33/MRPL24/MRPS21/RPL27/FAU/RPS27L/RPLP2/MRPS17/RPS28/MRPL4/MRPL11/RSL24D1/MRPS5/RPS26/MRPL23/MRPL2/UBA52/RPL36AL/MRPL27/MRPL9/MRPL28/MRPS2/MRPS7/MRPL1/MRPL30 |
| hsa05016 | Huntington disease | 282 | 0.527744 | 1.18E-08 | 4.93E-07 | C6/IL12A/FGG/CXCL8/MAPK10/MAS1/IL12B/CFB/C4B/C4A/FGA/RPL17-C18orf32/RPL36A-HNRNPH2/PIK3R2/TRAF3/RPLP0/C8A/RPS5/RPL22L1/RPS8/C9/RPSA/RPS6/RPS3A/RPS19/RPL17/RPS4X/RPL34/RPL13A/RPL10A/RPL12/RPL32/C5AR1/RPL26/RPL35A/RPL4/RPS18/RPL41/RPS10/RPLP1/RPL13/C8B/RPL38/RPL29/RPL31/RPS17/RPS24/RPL39/RPL5/RPS3/RPS9/RPS15A/RPL36A/RPL27A/RPS27A/RPL7A/RPL3/RPL23/FOS/RPS7/RPS14/RPL6/RPS27/RPL8/CCL2/RPS25/RPL7/RPL19/RPL36/RPL21/RPS21/RPL23A/RPS11/RPS2/RPL14/RPS15/RPL15/RPL11/RPL24/RPL18A/RPL37A/RPL18/RPS10-NUDT3/RPS13/RPS12/RPL35/RPS20/RPS29/RPS16/RPL37/RPL28/RPL22/RPS23/IKBKE/RPL26L1/RPL30/RPL10/MMP3/RPL27/FAU/RPS27L/STING1/C5/RPLP2/PRKCG/RPS28 |
| hsa04024 | cAMP signaling pathway | 204 | 0.560912 | 2.23E-08 | 6.81E-07 | HTR2C/ATP2B3/CCKBR/OXTR/GRIN3B/MYLK3/MRLN/CALML4/BDKRB1/SMIM6/AVPR1A/SLN/ERBB4/BDKRB2/CACNA1G/GRIN1/NTRK2/RET/DRD1/GRM5/ADRA1B/ADCY2/MET/ADORA2B/FGFR3/FGF16/GNA14/TACR1/GDNF/FGF20/DRD5/ATP2B2/P2RX3/ATP2A2/PDE1A/CACNA1E/ORAI2/PDGFC/CACNA1F/ERBB2/PHKA1/HTR2A/ERBB3/PHKG2/RYR3/ATP2A1/FGF5/CACNA1I/NGF/CACNA1B/PTGFR/TGFA/PPIF/CACNA1A/P2RX6/PLN/CD38/FGF7/CALM1/NFATC4 |
| hsa04020 | Calcium signaling pathway | 235 | 0.549241 | 1.86E-08 | 6.81E-07 | DNAI2/DNAH5/DNAH7/DNAI1/DNAH9/DNAH12/DNAH6/DNAH10/WNT16/DNAH11/DNAH3/DNAH2/CALML4/SPTBN2/GRIA2/WNT8B/DNALI1/GRIN1/SLC6A3/WNT4/COX6B2/DNAL1/WNT10A/GRM5/MAPK10/DNAH1/WNT7B/TUBB8/SNCAIP/TUBAL3/TUBB4B/TUBB3/DNAL4/FZD3/IFT57/GPX2/NDUFC2-KCTD14/ATP2A2/WNT5B/UCHL1/DNAH14/DNAH8/C9orf72/FZD9/TUBB4A/CACNA1F/SEPTIN5/GPR37/RYR3/ATP2A1/CACNA1B/FRAT1/WNT5A/CASP9/PPIF/GPX8/FRAT2/STX1A/PPID/CALM1/RPS27A/SOD1/MAP1LC3A/VDAC3/TRAP1/NDUFAB1/WNT6/CDK5R1/TUBA3D/MCU/CSNK2A3/TUBA1A/ATP2A3/COX7A2L/CALM2/CAMK2D/COX7C/NDUFA8/PRPH/SEM1/NDUFB3/SLC25A4/NDUFA4/NDUFC1/ATXN2/CAMK2B/NDUFB5/RB1CC1/CSNK2A1/MAP2K3/GPX7/COX6B1/NDUFB7/ATP5PO/NDUFA5/LRP5/ATP5F1A/UQCRC1/COX5B/UQCRB/MAP1LC3B2/COX5A/SDHC/PSMB5/ATP5PF/KLC3/UQCR11/PRKCG/NDUFS6/DLG4/WIPI1/NDUFA1/ACTR1B/GRM1/CALML6/CAPN2/HSPA5/CHMP2B/PSMB7/GRIA3/PARK7/NDUFA10/NDUFA13/ATP5MC1/NDUFB4/COX7B/BCL2/ATP5MC2/UQCRC2/UQCRH/SDHA/NDUFB8/ATP5F1D/UBA1/PSMA5/UQCRFS1/COX6A1/KLC4/UBB/NDUFB1/ATP5PB/NDUFS7/FZD6/MAP2K6/VCP/UBA52/NDUFA2/UBE2G1/PSMD4/ACTR10/UQCR10/NDUFA7/SIGMAR1/DCTN3/PSMC4/DVL2/XBP1/CTNNB1/SDHB/ATP5F1B/COX6C/PSMC6/NDUFB9/NDUFB10/WIPI2/NDUFB6/SLC25A6/COX8A/MAPK8/RYR1/NDUFS1/BAD/MFN1/CASP3/ATP5PD/TRAF2/ALS2/PSMD14/TOMM40/CASP7/CALM3/CAPN1/SDHD/CDK5/SPG11/UQCRQ |
| hsa05022 | Pathways of neurodegeneration - multiple diseases | 445 | 0.476597 | 2.21E-08 | 6.81E-07 | NXF2B/NXF2/ZNF473/ZNF98/ZNF273/IL12A/ZNF214/ZNF440/ZNF19/ZNF99/ZNF157/ZNF492/HLA-DRB1/POU2F3/ZNF257/ZNF20/IL12B/ZNF132/ZNF709/ZNF730/ZNF454/ZNF599/ZNF10/ZNF606/ZNF229/ZNF311/ZNF285/ZNF713/ZNF3/ZNF208/NECTIN1/PIK3R2/ZNF607/ZFP69B/ZNF627/ZNF584/ZNF726/ZNF667/TRAF3/ZNF658/ZNF688/ZNF33B/ZNF486/ZNF324B/ZNF749/CASP9/ZNF732/ZNF334/ZNF888/ZNF559-ZNF177/ZNF233/ZNF23/ZNF552/ZNF550/ZNF853/ZNF253/TRADD/ZFP37/ZNF610/ZNF764/ZNF420/ZNF382/ZNF875/ZNF85/CCL2/ZNF681/ZFP90/ZNF782/ZFP14/ZNF468/ZNF551/ZNF786/ZNF619/ZNF554/ZNF490/ZNF860/ZNF43/ZNF230/ZNF768/ZNF264/ZNF254/ZNF780B/ZNF268/ZNF561/ZNF251/ZNF596/ZNF225/ZNF586/ZNF816/ZNF425/ZNF485/ZNF415/ZNF26/IKBKE/ZNF727/ZNF529/ZNF587/ZNF564/POU2F2/ZNF680/ZNF114/ZNF284/ZNF404/ZNF714/ZNF793/ZNF738/ZNF83/ZNF624/ZNF605/ZNF180/ZNF763/ZNF112/ZNF670/EIF2AK1/ZNF84/ZNF543/ZNF510/STING1/C5/ZNF184/ZNF417/ZNF223/ZNF107/AKT2/ZNF799/ZNF544/ZNF320/ZNF616/ZNF717/SRSF9/ZNF432/ZNF829/ZNF267/ZNF135/ZNF777/ZNF525/ZNF286A/BCL2/SRSF8/TP53/ZNF77/ZNF182/ZNF347/ZNF514/ZNF33A/ZNF776/ZNF565/EIF2AK4/ZNF785/ZNF430/RBAK/EIF2B5/ZNF200/ZNF175/ZNF140/ZNF74/ZNF808/ZNF558/ZNF12/ZNF880/IFNGR2/SRPK1/PILRB/SRSF6/HLA-DMA/TAB1/ZNF585A/ZNF226/ZNF91/ZNF14/ZNF736/SRSF7/ZNF891/ZNF439/ZNF836/ZNF846/IRF3/ZNF600/ZNF585B/BAD/MAP3K7/CASP3/SRSF1/ZNF517/TRAF2/ZNF569/ZNF169/ZNF675/ZNF350/HLA-DOB/ZNF527/ZNF354B/ZNF92/C3/ZNF480/ZNF79/IFNAR1/ZNF626/ZNF2/ZNF212/ZNF471/ALYREF/ZNF354A/ZNF441/SYK/ZNF302/ZNF669/ZNF324/ZNF300/ZNF90/ZNF562/ZNF708/ZNF282/ZNF766/ZNF398 |
| hsa05168 | Herpes simplex virus 1 infection | 482 | 0.463161 | 6.18E-08 | 1.72E-06 | KCNK2/SST/ATP2B3/CRH/CNGA4/OXTR/CNGA3/TNNI3/GRIN3B/HCN4/GIPR/HCAR1/CALML4/GRIA2/GHRL/GRIN1/CNGA1/DRD1/MAPK10/SOX9/ADCY2/OXT/CNGB1/TSHR/DRD5/ATP2B2/ATP2A2/CREB3L4/CFTR/CACNA1F/PAK1/VIPR2/PIK3R2/ATP1B1/ATP2A1/PTGER2/PLD1/DRD2/RAC3/ADCYAP1/LIPE/LHB/PLN |
| hsa01240 | Biosynthesis of cofactors | 141 | 0.58427 | 2.33E-07 | 5.99E-06 | UGT1A6/UGT2A1/UGT2B17/GSTA3/UGT2B15/GSTA1/GSTA2/UGT1A7/TNFSF11/ADCY2/KPNA7/FGF16/EPHX3/UGT1A1/FGF20/HSP90AA1/RPS6KA6/PAQR7/CREB3L4/MGST1/CACNA1F/PTGES3/CYP1B1/UGT2B7/PIK3R2/GSTM2/CYP2B6/EPHX2/CDC6/CHRNA4/FGF5/CACNA1B/GSTO2/KLF5/MYC/PAQR8/GSTM4/CACNA1A/FGF7/GSTM1/HSP90AB1/KPNA5/FOS/JAG1 |
| hsa04976 | Bile secretion | 71 | 0.683103 | 3.72E-07 | 8.90E-06 | GNG13/BIRC7/GSTA3/CCNA1/GSTA1/GSTA2/WNT16/IL5RA/CDKN2A/CALML4/ALK/BDKRB1/LPAR3/WNT8B/IL7/BDKRB2/IL12A/WNT4/RET/CXCL8/WNT10A/MAPK10/ADCY2/WNT7B/PTCH2/MET/EGLN3/HEY2/IL23R/IGF2/FZD3/FGFR3/FGF16/LPAR4/IL12B/FGF20/SLC2A1/HSP90AA1/NQO1/WNT5B/MSH3/BMP4/PIM2/GNB3/MGST1/FZD9/CDH1/CTNNA3/TRAF4/ERBB2/DAPK1/GNG4/PIK3R2/MECOM/GSTM2/FGF5/GSTO2/PTGER2/FRAT1/TRAF3/WNT5A/PLD1/CCNA2/RUNX1/TGFA/CASP9/MYC/RAC3/GNG3/GSTM4/GNB5/FRAT2/LAMB4/JAG2/TERT/FGF7/GSTM1/CALM1/GADD45G/HSP90AB1/RARB/COL4A6/FOS/JAG1/TXNRD3/WNT6/MSH2/PGF/TGFB3/GSTT2B/KEAP1/MDM2/GSTP1/GNG12/CALM2/CAMK2D/ESR2/SKP1/FGF9/AR/GSTT2/MMP9/TGFBR1/MSH6/RALGDS/GADD45A/RAD51/TXNRD1 |
| hsa05207 | Chemical carcinogenesis - receptor activation | 177 | 0.560949 | 4.31E-07 | 9.62E-06 | GNG13/CRH/GRIN3B/H2BU1/CALML4/H2AC17/SHC4/GRIN1/SLC6A3/NTRK2/DRD1/SLC18A1/H2AW/SLC18A2/H3C15/H3C14/ADORA2B/H4C15/H2BC5/H2BC18/H4C14/H3C8/H2BC9/GNB3/CREB3L4/H2AC8/GNG4/SLC29A3/H2BC11/H4C11/MACROH2A2/DRD2/GNG3/H4C12/GNB5/H2BC15/FOSB/H3C6/CALM1 |
| hsa05200 | Pathways in cancer | 492 | 0.450353 | 4.66E-07 | 9.76E-06 | GNG13/SPP1/IBSP/EFNA2/COMP/MYB/LPAR3/IL7/ERBB4/NTRK2/RET/NTF3/MET/COL9A2/NGFR/EREG/IGF2/FGFR3/FGF16/LPAR4/GDNF/FGF20/MAGI2/ITGB8/HSP90AA1/PRKAA2/CD19/NR4A1/COL9A3/THBS4/GNB3/PDGFC/CREB3L4/ERBB2/GNG4/ITGA11/PIK3R2/ITGA7/ERBB3/ANGPT2/FGF5/AREG/NGF/ITGB4/PPP2R2C/TGFA/PHLPP1/CASP9/MYC/GNG3/GNB5/RPS6/PIK3R6/INSR/LAMB4/ARTN/SGK2/EPHA2/FGF7/HSP90AB1/EFNA5/THBS2/NTF4 |
| hsa05034 | Alcoholism | 148 | 0.580914 | 7.37E-07 | 1.45E-05 | UGT1A6/UGT2A1/BAAT/UGT2B17/UGT2B15/UGT1A7/ABCB1/ABCB11/ADCY2/SLC4A5/UGT1A1/SLC2A1/SLC10A1/CFTR/ABCC3/UGT2B7/ATP1B1/ABCB4/SLC51A/SLC27A5 |
| hsa00982 | Drug metabolism - cytochrome P450 | 57 | 0.720306 | 7.88E-07 | 1.47E-05 | UGT1A6/UGT2A1/ADH7/UGT2B17/CYP2W1/UGT2B15/UGT1A7/ADH6/RDH12/CYP27C1/ADH1C/UGT1A1/ALDH1A1/CYP2S1/DHRS3/CYP2A6/CYP2A7/PNPLA4/DHRS9/UGT2B7/CYP2B6/CYP2C8 |
| hsa04151 | PI3K-Akt signaling pathway | 319 | 0.473737 | 1.47E-06 | 2.60E-05 | UGT1A6/UGT2A1/UGT2B17/UGT2B15/AK8/AK7/MAT1A/UGT1A7/AK9/RDH12/NME7/PSAT1/TDO2/PPOX/ALPG/ALPP/UGT1A1/NQO1/ADSS1/AK5/GCLM/UGDH/DHRS3/AK4/GCLC/UGT2B7/PHOSPHO2/SPR/ALDH1B1/NME1-NME2/NME1/RFK/MTHFD2L/MPI/COQ7/COX10/PANK1/COQ3/ADSS2/PDXP/NME6/NADK2/EARS2/MMAB/DHFR2/EPRS1/PMM1/BCO1/COASY/PTS/HAAO/COX15/UMPS/COQ5/SHMT2/AKR1A1/HSD17B6/CAD/HMBS/GMPPA/MTHFD2/LIAS/NFS1/PMM2/ADSL/FLAD1/DHODH/CTPS2/NMNAT1/AK6/GMPPB |
| hsa00830 | Retinol metabolism | 53 | 0.716977 | 2.53E-06 | 4.24E-05 | UGT1A6/UGT2A1/ADH7/UGT2B17/GSTA3/UGT2B15/GSTA1/GSTA2/UGT1A7/ALDH3B2/ADH6/ALDH3A1/ADH1C/ALDH3B1/UGT1A1/MGST1/CYP2A6/CYP2A7/UGT2B7/GSTM2/CYP2B6/GSTO2/CYP2C8/GSTM4/FMO3 |
| hsa05204 | Chemical carcinogenesis - DNA adducts | 54 | 0.698878 | 3.23E-06 | 4.92E-05 | UGT1A6/UGT2A1/UGT2B17/CYP2A13/GSTA3/UGT2B15/GSTA1/GSTA2/UGT1A7/NAT1/HSD11B1L/NAT2/UGT1A1/MGST1/CYP2A6/CYP2A7/CYP1B1/UGT2B7/GSTM2/GSTO2/CYP2C8/GSTM4/AKR1C2 |
| hsa00230 | Purine metabolism | 122 | 0.575348 | 3.17E-06 | 4.92E-05 | UGT1A6/UGT2A1/UGT2B17/GSTA3/UGT2B15/GSTA1/GSTA2/UGT1A7/XDH/NAT1/NME7/NAT2/UPP2/UGT1A1/MGST1/CYP2A6/CYP2A7/UGT2B7/GSTM2/GSTO2/NME1-NME2/GSTM4/NME1/HPRT1/IMPDH2/GSTM1/RRM2/CYP2E1/NME6/RRM2B/TYMP/GSTT2B/GSTP1/TK1 |
| hsa00983 | Drug metabolism - other enzymes | 67 | 0.681776 | 6.11E-06 | 8.53E-05 | GUCY2F/AMPD1/AK8/AK7/GDA/XDH/PDE11A/AMPD3/AK9/ADCY2/NT5E/GUCY2C/ENTPD3/NME7/ENPP3/ADSS1/PDE6B/AK5/PDE1A/ENTPD2/AK4/NT5C1B/FHIT/PAICS/NPR2/NME1-NME2/NME1/PAPSS1/HPRT1/ADK/PRPS1/IMPDH2/NT5M/PDE2A/ADSS2/RRM2/PFAS/NME6/RRM2B/PRPS2 |
| hsa04950 | Maturity onset diabetes of the young | 18 | 0.831518 | 6.00E-06 | 8.53E-05 | NKX2-2/HNF1A/HNF4A/HNF4G/NEUROD1/BHLHA15/NKX6-1/PAX6/FOXA2/GCK/FOXA3 |
| hsa04110 | Cell cycle | 157 | 0.521824 | 7.81E-06 | 0.000101 | HOXB1/ISL1/BMPR1B/SOX2/WNT16/INHBB/DUSP9/OTX1/WNT8B/WNT4/WNT10A/WNT7B/FZD3/FGFR3/PAX6/ESRRB/LIF/WNT5B/BMP4/FZD9/PIK3R2/WNT5A/DLX5/ACVR1B/SMAD1/MYC |
| hsa05012 | Parkinson disease | 241 | 0.496038 | 7.61E-06 | 0.000101 | GNG13/EFNA2/PAK5/CALML4/PLA2G2D/PLA2G4E/SHC4/GRIN1/NTRK2/MAPK10/PAK6/NTF3/MET/RASAL1/NGFR/KSR2/IGF2/FGFR3/FGF16/FGF20/SYNGAP1/PLA2G12B/GNB3/PDGFC/PAK1/GNG4/PIK3R2/PLA2G4B/ANGPT2/FGF5/NGF/PLD1/PLA2G6/TGFA/RAC3/GNG3/RASGRF2/GNB5/INSR/EPHA2/FGF7/CALM1/EFNA5/NTF4/PLA2G10 |
| hsa04934 | Cushing syndrome | 147 | 0.546267 | 1.42E-05 | 0.000176 | KCNK2/CRH/WNT16/CYP11A1/CDKN2A/WNT8B/PDE11A/CACNA1G/WNT4/WNT10A/ADCY2/WNT7B/STAR/FZD3/WDR5B/RASD1/NR4A1/WNT5B/CREB3L4/FZD9/CACNA1F/CACNA1I/WNT5A |
| hsa04972 | Pancreatic secretion | 84 | 0.614718 | 1.65E-05 | 0.00019 | EFNA2/CACNG6/DUSP9/PLA2G4E/ERBB4/CACNA1G/NTRK2/RET/MAPK10/MAPK8IP1/NTF3/MET/NGFR/EREG/IGF2/FGFR3/FGF16/FLNC/GDNF/FGF20/RPS6KA6/NR4A1/CACNG8/CACNA1E/HSPA1L/PDGFC/CACNA1F/ERBB2/FLNB/PAK1/MECOM/ERBB3/PLA2G4B/ANGPT2/FGF5/AREG/CACNA1I/NGF/CACNA1B/TGFA/MYC/RAC3/RASGRF2/CACNA1A/INSR/DUSP10/ARTN/HSPB1/DUSP5/EPHA2/FGF7/GADD45G/EFNA5/NTF4/TRADD/DUSP1/FOS/JMJD7-PLA2G4B/PGF/MAP3K13/TGFB3/EFNA4/GNG12/FGF9/MAPK8IP2/TGFBR1/EFNA3/PSPN/GADD45A |
| hsa04550 | Signaling pathways regulating pluripotency of stem cells | 134 | 0.562871 | 1.62E-05 | 0.00019 | CALML4/SLC6A3/COX6B2/DRD1/MAPK10/SLC18A1/TUBB8/SLC18A2/SNCAIP/TUBAL3/TUBB4B/TUBB3/NDUFC2-KCTD14/SLC39A6/UCHL1/TUBB4A/SEPTIN5/GPR37/RYR3/SLC39A5/DRD2/CASP9/PPIF/CALM1/SLC11A2/RPS27A/DUSP1/SOD1/VDAC3/TRAP1/NDUFAB1/TUBA3D/MCU/TUBA1A/KEAP1/TXN2/COX7A2L/SLC39A11/CALM2/CAMK2D/COX7C/NDUFA8/SEM1/NDUFB3/SLC25A4/NDUFA4/NDUFC1/CAMK2B/NDUFB5/COX6B1/NDUFB7/ATP5PO/NDUFA5/ATP5F1A/UQCRC1/COX5B/UQCRB/COX5A/SDHC/PSMB5/ATP5PF/KLC3/UQCR11/NDUFS6/NFE2L2/NDUFA1/CALML6/HSPA5/PSMB7/PARK7/NDUFA10/NDUFA13/ATP5MC1/NDUFB4/COX7B/ATP5MC2/UQCRC2/UQCRH/TP53/SDHA/NDUFB8/ATP5F1D/UBA1/PSMA5/UQCRFS1/COX6A1/KLC4/UBB/NDUFB1/ATP5PB/NDUFS7/UBA52/NDUFA2/UBE2G1/PSMD4/UQCR10/SLC39A10/NDUFA7/PSMC4/XBP1/SDHB/ATP5F1B/COX6C/PSMC6/NDUFB9/NDUFB10/NDUFB6/SLC25A6/COX8A/MAPK8/ADCY5/NDUFS1/MFN1/CASP3/ATP5PD/PSMD14/CALM3/SDHD/UQCRQ/COX7A2/NDUFS4/NDUFS2/COX4I1/ATP5MC3/NDUFS3/NDUFV3/VDAC1/MAOB/NDUFB2/PSMB1/CYC1 |
| hsa00190 | Oxidative phosphorylation | 116 | 0.564508 | 1.75E-05 | 0.000196 | GSTA3/GSTA1/GSTA2/WNT16/CDKN2A/WNT8B/SHC4/WNT4/WNT10A/WNT7B/MET/IGF2/FZD3/NQO1/WNT5B/MGST1/FZD9/PIK3R2/GSTM2/GSTO2/FRAT1/WNT5A/TGFA/MYC/GSTM4/FRAT2/SMARCB1/TERT/GSTM1/GADD45G/TXNRD3/WNT6/TGFB3/GSTT2B/KEAP1/GSTP1/GSTT2/TGFBR1/SMARCA4/GADD45A/TXNRD1/CDKN1A/ACTL6A/DPF1/LRP5/SMAD2/SMARCC1/PRKCG/MGST2/NFE2L2/AKT2 |
| hsa01232 | Nucleotide metabolism | 82 | 0.620353 | 1.85E-05 | 0.000198 | ATP2B3/CLCA2/CLCA4/AMY1B/AMY1C/AMY1A/PLA2G2D/CPB1/ADCY2/CELA2A/CPA3/RAB27B/ATP2B2/PLA2G12B/ATP2A2/CFTR/CEL/KCNMA1/ATP1B1/ATP2A1/AMY2B/CD38/AMY2A/PLA2G10 |
| hsa04010 | MAPK signaling pathway | 288 | 0.46109 | 1.89E-05 | 0.000198 | UGT1A6/UGT2A1/UGT2B17/SRD5A2/UGT2B15/UGT1A7/CYP11A1/HSD17B2/LRTOMT/HSD11B1L/HSD17B3/UGT1A1/AKR1C1/CYP1B1/UGT2B7/HSD17B11/CYP17A1/AKR1C2/HSD17B8/CYP2E1/CYP21A2/SULT2B1 |
| hsa04726 | Serotonergic synapse | 103 | 0.567041 | 1.97E-05 | 0.0002 | MEP1A/COL10A1/COL17A1/SLC15A1/COL11A1/CPB1/COL28A1/COL7A1/CELA2A/COL9A2/CPA3/KCNK5/COL9A3/PGA5/COL25A1/COL21A1/SLC7A9/ATP1B1/KCNN4/SLC36A4/SLC3A1/COL8A2/COL15A1/SLC16A10 |
| hsa05208 | Chemical carcinogenesis - reactive oxygen species | 203 | 0.503776 | 2.80E-05 | 0.000276 | CYP2F1/GSTA3/GSTA1/GSTA2/LPO/COX6B2/MAPK10/MET/EPHX3/NDUFC2-KCTD14/NQO1/AKR1C1/MGST1/CYP1B1/PIK3R2/GSTM2/EPHX2/GSTO2/PLD1/GSTM4/PPIF/AKR1C2/GSTM1/SOD1/VDAC3/FOS/CYP2E1/NDUFAB1/GSTT2B/KEAP1/PRKCD/COX7A2L/COX7C/GSTT2/NDUFA8/NDUFB3/SLC25A4/NDUFA4/NDUFC1/NDUFB5/COX6B1/ACP1/NDUFB7/ATP5PO/NDUFA5/ATP5F1A/UQCRC1/COX5B/UQCRB/COX5A/SDHC/EPHX4/ATP5PF/UQCR11/AKR1C3/NDUFS6/AKR1A1/MGST2/NFE2L2/NDUFA1/AKT2/NDUFA10/NDUFA13/ATP5MC1/NDUFB4/COX7B/ATP5MC2/UQCRC2/UQCRH/SDHA/NDUFB8/ATP5F1D/UQCRFS1/COX6A1/CBR1/NDUFB1/ATP5PB/NDUFS7/NDUFA2/EPHX1/UQCR10/NDUFA7/SDHB/ATP5F1B/COX6C/NDUFB9/NDUFB10/NDUFB6/SLC25A6/COX8A/MAPK8/ABL1/NDUFS1/BAD/ATP5PD/MGST3/SDHD/UQCRQ/COX7A2/PLD2/NDUFS4/NDUFS2/COX4I1/ATP5MC3/NDUFS3/NDUFV3 |
| hsa04014 | Ras signaling pathway | 222 | 0.497308 | 3.06E-05 | 0.000293 | CCNA1/CDKN2A/TRIP13/SFN/CDT1/MAD2L1/WEE1/CCNB1/TTK/CDC14A/ESCO2/ANAPC4/CDC20/CDC14B/CDC6/MCM2/CCNA2/CDK7/MCM4/MYC/E2F5/CCNB2/BUB1B/GADD45G/CDK1/ATR/ORC6/SMC1B/TGFB3/MDM2/ORC4/CHEK1/ORC3/SKP1/KNL1/PLK1/AURKB/GADD45A/CDKN1A/TFDP2/PCNA/SMC3/PPP2R1A/HDAC1/ANAPC10/RBL1/ANAPC16/SMAD2/DBF4B/CDC16/HDAC2/DBF4/BUB1/YWHAE/SMC1A/TFDP1/ANAPC13/ANAPC1/CUL1/ANAPC5/CDC26/CDC25A/TP53/ORC5/WEE2/CDKN1B/SGO1 |
| hsa04974 | Protein digestion and absorption | 89 | 0.588171 | 3.28E-05 | 0.000305 | AMPD1/AK8/AK7/GDA/XDH/AMPD3/AK9/NT5E/ENTPD3/NME7/UPP2/ENPP3/ADSS1/AK5/ENTPD2/AK4/NT5C1B/NME1-NME2/NME1/HPRT1/ADK/IMPDH2/NT5M/ADSS2/RRM2/TYMS/NME6/RRM2B/TYMP/TK1/DCTPP1 |
| hsa05225 | Hepatocellular carcinoma | 162 | 0.521048 | 5.14E-05 | 0.000466 | ATP4B/ATP12A/ATP6V0D2/ATP6V1B1/COX6B2/NDUFC2-KCTD14/ATP6V1C2/LHPP/ATP6V1D/COX10/NDUFAB1/COX7A2L/COX7C/NDUFA8/NDUFB3/NDUFA4/NDUFC1/NDUFB5/COX6B1/NDUFB7/ATP5PO/NDUFA5/COX15/ATP6V0E2/ATP5F1A/UQCRC1/COX5B/UQCRB/COX5A/SDHC/ATP5PF/UQCR11/NDUFS6/NDUFA1/ATP5ME/PPA2/NDUFA10/NDUFA13/ATP5MC1/ATP6V0A4/NDUFB4/COX7B/ATP5MC2/UQCRC2/UQCRH/SDHA/NDUFB8/ATP5F1D/UQCRFS1/COX6A1/NDUFB1/ATP5PB/NDUFS7/COX11/NDUFA2/ATP5MG/UQCR10/NDUFA7/SDHB/ATP5F1B/ATP6V1E2/COX6C/ATP6V1H/NDUFB9/NDUFB10/NDUFB6/COX8A/NDUFS1/ATP5PD/ATP5MF/SDHD/UQCRQ/COX7A2/ATP6AP1/NDUFS4/NDUFS2/COX4I1/ATP5MC3/NDUFS3/NDUFV3/ATP6V1G1/NDUFB2/CYC1/ATP6V1F/NDUFS5/ATP6V1G2/NDUFS8/UQCRHL/ATP6V1C1/NDUFA11 |
| hsa00140 | Steroid hormone biosynthesis | 44 | 0.680732 | 5.69E-05 | 0.000472 | GNG13/GRM4/GRIN3B/GRIK2/SLC17A8/GRIA2/GRIK5/PLA2G4E/GRM7/SLC1A6/GRIN1/GRM5/ADCY2/GLS2/GNB3/GNG4/SHANK2/PLA2G4B/PLD1/GNG3/HOMER2/GNB5/CACNA1A |
| hsa00860 | Porphyrin metabolism | 32 | 0.72038 | 5.75E-05 | 0.000472 | EFNA2/BMPR1B/PAK5/NGEF/EFNB3/LRRC4/GDF7/EPHA7/SRGAP3/WNT4/PAK6/RGMA/MET/FZD3/WNT5B/NTN1/BMP7/SEMA6B/PAK1/PLXNB1/PIK3R2/SEMA4B/WNT5A/EPHB2/RAC3/PLXNB2/EPHB3/SEMA4G/EPHA2/NFATC4/RND1/EFNA5/PLXNC1/ENAH/SLIT1/NTN3/EFNA4/CAMK2D/UNC5A/EFNA3/EPHB1/SSH3/CAMK2B |
| hsa01200 | Carbon metabolism | 106 | 0.561026 | 5.39E-05 | 0.000472 | CST1/ATP2B3/AQP5/AMY1B/AMY1C/CALML4/AMY1A/LPO/TRPV6/ADRA1B/ADCY2/ATP2B2/KCNMA1/ATP1B1/KCNN4/RYR3/CST5/AMY2B/CD38/CALM1/AMY2A/PRH2 |
| hsa05226 | Gastric cancer | 138 | 0.536172 | 5.78E-05 | 0.000472 | HTR3E/GNG13/HTR2C/ALOX15/CYP2J2/GABRB3/PLA2G4E/CYP4X1/SLC18A1/SLC18A2/TPH1/GNB3/CACNA1F/GNG4/HTR2A/PLA2G4B/CACNA1B/CYP2C8/GNG3/GNB5/CACNA1A |
| hsa04970 | Salivary secretion | 79 | 0.61501 | 5.94E-05 | 0.000473 | CDX2/MUC2/WNT16/ABCB1/WNT8B/SHC4/WNT4/WNT10A/WNT7B/MET/CDH17/FZD3/FGF16/FGF20/WNT5B/FZD9/CDH1/CTNNA3/ERBB2/PIK3R2/FGF5/FRAT1/WNT5A/MYC/FRAT2/TERT/FGF7/GADD45G/RARB |
| hsa04724 | Glutamatergic synapse | 111 | 0.558956 | 6.07E-05 | 0.000473 | VTCN1/CLDN8/CLDN16/CDH3/IGSF11/CLDN10/LRRC4/CLDN9/CDH2/CLDN1/SLITRK6/CLDN19/CLDN3/HLA-DRB1/CLDN20/ITGB8/CD8B2/CLDN4/NRXN1/CNTN2/CNTNAP2/CDH1/NECTIN1/PTPRF/NEGR1/CTLA4 |
| hsa05206 | MicroRNAs in cancer | 174 | 0.49829 | 6.62E-05 | 0.000504 | UGT1A6/UGT2A1/UGT2B17/UGT2B15/UGT1A7/UGT1A1/UGDH/UGT2B7/ALDH1B1 |
| hsa05010 | Alzheimer disease | 354 | 0.433108 | 7.88E-05 | 0.000587 | SDS/ENO4/OGDHL/PSPH/PSAT1/AGXT/ALDOB/PHGDH/GPT2/FBP2/GCK/IDH1/TKFC/HKDC1/PC/RPEL1/AMT/DLAT/IDH2/PFKP/PRPS1/GOT1/ACSS1/ENO2/SDSL/MCEE/PRPS2/MDH1/HK1/ALDOC/GOT2/IDNK/ACO2/SDHC/IDH3A/SHMT2/ACOX3/SUCLA2/ECHS1/MMUT/ENO1/GPI/SUCLG2/SUCLG1/SDHA/PCCB/ESD/MDH2/PGLS/SHMT1/ALDH6A1/PKM/SDHB |
| hsa04514 | Cell adhesion molecules | 149 | 0.51834 | 0.000102 | 0.000724 | CLDN8/CLDN16/IGSF5/CLDN10/CLDN9/TJP3/CLDN1/EPB41L4B/MAPK10/CLDN19/TUBAL3/CLDN3/CLDN20/PRKAA2/CLDN4/MARVELD3/CFTR/ERBB2/ACTR3B/RUNX1/PPP2R2C/CLDN6/AMOT/CD1A/YBX3/PRKAB1/MYH11/MICALL2/MPDZ/TUBA3D/TUBA1A/NEDD4L/CLDN12/MYH14/CGN/EZR/DLG3/PCNA/PARD6G/HSPA4/MARVELD2/SLC9A3R1/PPP2R1A/ACTR3C/LLGL2 |
| hsa04062 | Chemokine signaling pathway | 183 | 0.493703 | 0.000102 | 0.000724 | RNVU1-3/NCBP2L/PRPF40B/HSPA1L/RNVU1-1/PPIL1/RNU2-1/CDC5L/LSM5/PRPF4/SRSF10/PPIE/EIF4A3/LSM7/SF3B2/SNRPD2/HNRNPA1/THOC3/PRPF19/CHERP/HNRNPA1L2/PRPF6/HSPA8/SNRPC/LSM4/SF3B4/SF3B5/HNRNPC/SNRPD1/HNRNPM/SYF2/PRPF31/SNRPA/AQR/SRSF9/SF3B3/ZMAT2/SRSF8/SF3A1/SF3B6/RBMX/PPIH/SNW1/CWC15/SNRPD3/TCERG1/SNRNP40/SNU13/TRA2A/XAB2/SNRPF/TXNL4A/BUD31/SNRPE/PUF60/SRSF6/BCAS2/PRPF18/SRSF7/PLRG1/SNRPB/CCDC12/DHX16/HNRNPU/SRSF1/DDX42/DDX23/MAGOH/U2AF2/SNRPG/HNRNPK/CRNKL1/SF3A3/LSM3/WBP11/ALYREF/SNRNP200/CTNNBL1/PRPF8/HNRNPA3/SNRPA1/PRPF40A/EFTUD2/CDC40/HSPA1B/SNRPB2/NCBP2/NCBP1 |
| hsa00040 | Pentose and glucuronate interconversions | 23 | 0.748515 | 0.000113 | 0.000767 | SIX1/PROM1/EYA1/CCNA1/SIX4/WNT16/SLC45A3/BAIAP3/HMGA2/MLF1/CXCL8/HOXA10/MET/H3C15/H3C14/NGFR/H3C8/PAX5/FUT8/NR4A3/BMP2K/CCNA2/RUNX1/SMAD1/MYC/PLAT/MLLT1/H3C6/GADD45G/HOXA9/MLLT3/MDM2/SS18/MMP9/H3-5/GADD45A/PER2/SIN3A/CDKN1A/H3-3A/BCL6/HDAC1/CEBPA/H3-3B/MMP3/SUPT3H/PBX3/HDAC2/TCF3/GRIA3/FLT3/ATF1/MITF/TP53/TMPRSS2/TAF15/KDM6A/PBX1/IGF1R/CDKN1B/MEN1/DDB2 |
| hsa00010 | Glycolysis / Gluconeogenesis | 60 | 0.613783 | 0.000115 | 0.000767 | HTR2C/CALML4/CYP2J2/BDKRB1/TRPV4/PLA2G4E/BDKRB2/MAPK10/ADCY2/F2RL1/ASIC4/P2RY2/ASIC2/PIK3R2/HTR2A/PLA2G4B/NGF/PTGER2/PLA2G6/ASIC1/ASIC3 |
| hsa05202 | Transcriptional misregulation in cancer | 170 | 0.497465 | 0.000112 | 0.000767 | HTR3E/GNG13/GRM4/TRPM5/HCN4/CALHM1/SCNN1G/KCNK5/P2RX3/PDE1A/GNB3/ENTPD2/SCNN1B/ASIC2/TAS2R20/CACNA1A/GABRA2/SCN3A |
| hsa00053 | Ascorbate and aldarate metabolism | 18 | 0.781317 | 0.000127 | 0.000831 | GNG13/CCL15/CCL11/CXCL6/CXCL13/CXCL1/SHC4/CCR6/CXCL8/ADCY2/CCR8/CXCR5/CCL14/CCL13/CCL19/GNB3/CCL28/PAK1/GNG4/CXCL14/PIK3R2/CCL16/CCL27/RAC3/GNG3/GNB5/PIK3R6/CCL22 |
| hsa04750 | Inflammatory mediator regulation of TRP channels | 92 | 0.562139 | 0.000141 | 0.000908 | SDS/ENO4/MAT1A/PSPH/PSAT1/ALDOB/PHGDH/GPT2/PYCR3/IDH1/ASL/PC/ALDH18A1/PYCR1/RPEL1/IDH2/PFKP/ASS1/PRPS1/GOT1/ENO2/ARG2/SDSL/CTH/PRPS2/ABHD14A-ACY1/ALDOC/GOT2/ACO2/PYCR2/IDH3A/SHMT2 |
| hsa04061 | Viral protein interaction with cytokine and cytokine receptor | 90 | 0.551833 | 0.00015 | 0.000946 | SERPINB5/CYP24A1/TP63/EFNA2/MIR205/CDKN2A/SLC45A3/ABCB1/HMGA2/SHC4/MET/FZD3/FGFR3/GLS2/MIR200B/HOXD10/SOX4/MIR200A/ERBB2/IGF2BP1/CYP1B1/PIK3R2/ERBB3/MMP16/PDCD4/MYC/TRIM71/ST14/BMF/SLC7A1/EFNA5 |
| hsa04530 | Tight junction | 158 | 0.499826 | 0.000161 | 0.001 | WNT16/CALML4/IRS4/WNT8B/GRIN1/WNT4/COX6B2/PSENEN/WNT10A/GRM5/MAPK10/WNT7B/TUBB8/TUBAL3/APOE/TUBB4B/TUBB3/FZD3/NDUFC2-KCTD14/ATP2A2/WNT5B/SLC39A6/FZD9/TUBB4A/CACNA1F/PIK3R2/RYR3/ATP2A1/BACE2/SLC39A5/FRAT1/APH1B/WNT5A/CASP9/PPIF/FRAT2/PPID/INSR/CALM1/SLC11A2/VDAC3/NDUFAB1/WNT6/CDK5R1/APBB1/TUBA3D/MCU/CSNK2A3/TUBA1A/ATP2A3/COX7A2L/SLC39A11/CALM2/COX7C/NDUFA8/SEM1/NDUFB3/SLC25A4/NDUFA4/NDUFC1/NDUFB5/RB1CC1/CSNK2A1/COX6B1/NDUFB7/ATP5PO/NDUFA5/LRP5/ATP5F1A/UQCRC1/COX5B/UQCRB/COX5A/SDHC/PSMB5/ATP5PF/KLC3/UQCR11/NDUFS6/WIPI1/NDUFA1/CALML6/AKT2/CAPN2/IDE/PSMB7/NDUFA10/NDUFA13/ATP5MC1/NDUFB4/COX7B/ATP5MC2/UQCRC2/UQCRH/SDHA/NDUFB8/ATP5F1D/PSMA5/UQCRFS1/COX6A1/KLC4/NDUFB1/ATP5PB/NDUFS7/FZD6/NDUFA2/PSMD4/UQCR10/SLC39A10/NDUFA7/PSMC4/DVL2/XBP1/CTNNB1/SDHB/ATP5F1B/COX6C/PSMC6/NDUFB9/NDUFB10/WIPI2/NDUFB6/SLC25A6/COX8A/MAPK8/NDUFS1/BAD/CASP3/ATP5PD/TRAF2/PSMD14/CASP7/CALM3/CAPN1/SDHD/CDK5/UQCRQ/COX7A2/RTN3/NDUFS4/NDUFS2/COX4I1/ATP5MC3/NDUFS3/NDUFV3/ULK2/VDAC1/BECN1/NAE1/CSNK1A1/ATG2B/NDUFB2/PSMB1/WNT11/CYC1/ATG14/NDUFS5/HSD17B10/CSNK2B/GAPDH/NDUFS8/UQCRHL/NDUFA11/PIK3CB/PSMD8/SLC39A9 |
| hsa04360 | Axon guidance | 175 | 0.501971 | 0.000186 | 0.001131 | KCNK2/ATP4B/SST/KCNJ16/CCKBR/MYLK3/CALML4/ADCY2/CFTR/SLC26A7/ATP1B1 |
| hsa04390 | Hippo signaling pathway | 153 | 0.49037 | 0.000201 | 0.001205 | GSTA3/GSTA1/GSTA2/GGT6/CHAC1/GPX2/GCLM/MGST1/GGT5/IDH1/GCLC/GSTM2/GSTO2/GSTM4/IDH2/GPX8/GPX4/GGCT/GSTM1/GSR/RRM2/RRM2B/GSTT2B/NAT8/GSTP1/GSTT2/GPX7/ODC1/HPGDS/MGST2/GGT7/RRM1/SMS/TXNDC12 |
| hsa01524 | Platinum drug resistance | 71 | 0.588717 | 0.000211 | 0.00122 | GRIN3B/GABRP/SLC17A8/GRIA2/GABRB3/GRIN1/GABRG3/GABRQ/CHRNA4/CACNA1B/CACNA1A/GABRA2 |
| hsa04115 | p53 signaling pathway | 73 | 0.577767 | 0.00021 | 0.00122 | SERPINB5/TP73/CDKN2A/TP53AIP1/SFN/STEAP3/PERP/RPRM/CCNB1/ZNF385A/CASP9/CCNB2/SESN3/GADD45G/CDK1/ATR/RRM2/RRM2B/MDM2/SESN1/CHEK1/GTSE1/GADD45A/CDKN1A |
| hsa04012 | ErbB signaling pathway | 84 | 0.571525 | 0.000248 | 0.001408 | UGT1A6/UGT2A1/CP/UGT2B17/UGT2B15/UGT1A7/PPOX/UGT1A1 |
| hsa04714 | Thermogenesis | 210 | 0.468066 | 0.000258 | 0.001442 | ADH7/ENO4/ALDH3B2/ADH6/ALDH3A1/ADH1C/ALDH3B1/ALDOB/FBP2/GCK/HKDC1/ALDH1B1/DLAT/LDHAL6A/PFKP/LDHB/GALM/ACSS1/ENO2/LDHAL6B |
| hsa05230 | Central carbon metabolism in cancer | 66 | 0.586759 | 0.000279 | 0.001532 | CCL15/CCL11/CXCL6/CXCL13/CXCL1/CCR6/CXCL8/CCR8/CXCR5/CCL14/CCL13/IL20RA/IL22RA1/IL10/CCL19/ACKR3/CCL28/CXCL14/CCL16/CCL27/CCL22/CXCL2 |
| hsa04146 | Peroxisome | 80 | 0.557361 | 0.000295 | 0.001591 | CR2/C6/BDKRB1/SERPINB2/BDKRB2/F2RL2/FGG/F7/CFB/C4B/SERPINA5/PLG/C4A/CLU/PROC/FGA/C8A/CFI/C9/C4BPB/PLAT/CR1L/C5AR1/KLKB1/C8B/CFHR1/F8/CFH |
| hsa01230 | Biosynthesis of amino acids | 67 | 0.622081 | 0.000306 | 0.001612 | COX6B2/ADCY2/PRKAA2/NDUFC2-KCTD14/RPS6KA6/CREB3L4/BMP8B/PLIN1/NDUFAF6/RPS6/LIPE/SMARCB1/PRDM16/SLC25A29/COX10/PRKAB1/NDUFAB1/BMP8A/COX7A2L/CPT1B/NDUFAF8/COX7C/NPPA/NDUFA8/NDUFAF3/SMARCA4/KDM1A/NDUFB3/NDUFA4/NDUFC1/NDUFB5/MAP2K3/COX6B1/ACTL6A/NDUFAF2/DPF1/NDUFB7/ATP5PO/NDUFA5/COX15/ATP5F1A/ADCY10/UQCRC1/COX5B/UQCRB/COX5A/SDHC/ATP5PF/UQCR11/COA6/ADRB3/SMARCC1/NDUFS6/NDUFA1/ATP5ME/COA3/COX14/NDUFA10/NDUFA13/ATP5MC1/NDUFAF1/COX19/NDUFB4/KLB/MLST8/COX7B/ATP5MC2/UQCRC2/UQCRH/COA5/SDHA/NDUFB8/ATP5F1D/ZNF516/UQCRFS1/COX6A1/NDUFB1/ATP5PB/NDUFS7/COX11/COX16/NDUFA2/ATP5MG/UQCR10/NDUFA7/SDHB/ATP5F1B/COX6C/CREB3/COX20/SMARCA2/NDUFB9/NDUFB10/NDUFB6/COX8A/KDM3A/ADCY5/NDUFS1/COA1/ATP5PD/ATP5MF/CREB3L2/SDHD/UQCRQ/COX7A2/COA4/NDUFS4/NDUFS2/COX4I1/ATP5MC3/NDUFS3/NDUFV3/NDUFB2/CYC1/NDUFS5/RPTOR/RPS6KB1/NDUFS8/UQCRHL/NDUFA11 |
| hsa04610 | Complement and coagulation cascades | 77 | 0.589801 | 0.000308 | 0.001612 | PAK5/NRG4/SHC4/ERBB4/MAPK10/PAK6/EREG/NRG2/BTC/ERBB2/PAK1/PIK3R2/ERBB3/AREG/TGFA/MYC |
| hsa04742 | Taste transduction | 51 | 0.655128 | 0.000338 | 0.001743 | B3GNT3/GCNT3/ST6GALNAC1/GALNT15/C1GALT1C1L/B3GNT6/ST6GALNAC2/GALNT3/GALNTL6/GALNT16/GALNT6/GALNT12/GALNT7 |
| hsa00480 | Glutathione metabolism | 53 | 0.639478 | 0.000393 | 0.00197 | C6/H2BU1/H2AC17/H2AW/H3C15/H3C14/H4C15/HLA-DRB1/H2BC5/H2BC18/H4C14/H3C8/H2BC9/IL10/C4B/C4A/H2AC8/CTSG/H2BC11/H4C11/C8A/MACROH2A2/C9/H4C12/H2BC15/H3C6/C8B/H2BC6/H4-16/SSB/H2AB3/H2BC4 |
| hsa04072 | Phospholipase D signaling pathway | 143 | 0.488028 | 0.000394 | 0.00197 | BMPR1B/SOX2/TP73/WNT16/GDF7/WNT8B/WNT4/WNT10A/GDF6/WNT7B/FZD3/WNT5B/BMP4/RASSF6/CRB1/BMP7/FZD9/CDH1/CTNNA3/BMP8B/AREG/WNT5A/SMAD1/PPP2R2C/CRB2/WWC1/MYC/AMOT/WNT6/BMP8A/FRMD6/WTIP/TGFB3/SAV1/DLG5/DLG3/TGFBR1 |
| hsa04350 | TGF-beta signaling pathway | 103 | 0.519137 | 0.000426 | 0.002099 | CLDN8/WIPF3/CLDN16/CLDN10/CLDN9/CLDN1/CXCL8/MAPK10/TUBB8/CLDN19/TUBAL3/TUBB4B/CLDN3/TUBB3/LPAR4/CLDN20/BAIAP2L1/CLDN4/TUBB4A/PAK1/ACTR3B/MYO1E/TLR5/CASP9/CLDN6/TRADD/RPS3/FOS/MYH11/TUBA3D/TMBIM6/TUBA1A/CLDN12/MYO5C/MYH14/EZR/MYO1D |
| hsa03040 | Spliceosome | 134 | 0.5152 | 0.000479 | 0.002328 | CENPS/FANCF/POLN/CENPS-CORT/RMI1/RPA3/EME1/BRIP1/UBE2T/BLM/FANCB/ATR/RMI2/ERCC1/RAD51/FANCD2/RAD51C/POLH/RPA2/PMS2/MLH1/BRCA1/RPA1/FANCI/TOP3A/FAN1/ATRIP/PALB2/FANCL/TELO2/TOP3B/POLI/REV3L/ERCC4/USP1/FANCG |
| hsa03013 | Nucleocytoplasmic transport | 107 | 0.520153 | 0.000508 | 0.002433 | PTPRZ1/IGSF5/ATP6V0D2/CXCL1/ATP6V1B1/CXCL8/MAPK10/MET/ATP6V1C2/PAK1 |
| hsa05203 | Viral carcinogenesis | 181 | 0.46244 | 0.000522 | 0.002465 | IFNE/IL5RA/IL13RA2/IL7/IL11/IL12A/GFAP/IL23R/IL20RA/IL22RA1/CRLF2/IL12B/LIF/IL10/IL22RA2/IL31RA/PIK3R2/SOCS7/CNTF/SOCS6/MYC/PIAS3/CTF1 |
| hsa04630 | JAK-STAT signaling pathway | 132 | 0.496735 | 0.000562 | 0.002616 | C6/GRIN3B/GRIN1/COX6B2/MAPK10/TUBB8/TUBAL3/TUBB4B/TUBB3/NDUFC2-KCTD14/HSPA1L/CREB3L4/TUBB4A/CACNA1F/PIK3R2/RYR3/CACNA1B/C8A/CASP9/STIP1/C9/PPIF/C8B/EGR1/SOD1/VDAC3/NDUFAB1/TUBA3D/MCU/CSNK2A3/TUBA1A/PRKCD/GRIN3A/COX7A2L/COX7C/NDUFA8/SEM1/NDUFB3/SLC25A4/NDUFA4/NDUFC1/NDUFB5/CSNK2A1/NCAM1/COX6B1/NDUFB7/ATP5PO/NDUFA5/ATP5F1A/UQCRC1/COX5B/UQCRB/HSPA8/COX5A/SDHC/PSMB5/ATP5PF/KLC3/UQCR11/C5/NDUFS6/NDUFA1/HSPA5/PSMB7/NDUFA10/NDUFA13/ATP5MC1/NDUFB4/COX7B/ATF6B/ATP5MC2/UQCRC2/UQCRH/SDHA/NDUFB8/ATP5F1D/PSMA5/UQCRFS1/COX6A1/KLC4/NDUFB1/ATP5PB/NDUFS7/NDUFA2/PSMD4/UQCR10/NDUFA7/PSMC4/SDHB/ATP5F1B/COX6C/CREB3/PSMC6/NDUFB9/NDUFB10/NDUFB6/SLC25A6/COX8A/MAPK8/RYR1/NDUFS1/BAD/CASP3/ATP5PD/PSMD14/CREB3L2/SDHD/UQCRQ/COX7A2/NDUFS4/NDUFS2/COX4I1/ATP5MC3/NDUFS3/NDUFV3/VDAC1/NDUFB2/PSMB1/CYC1/NDUFS5/CSNK2B/NDUFS8/UQCRHL/NDUFA11/PIK3CB/PSMD8 |
| hsa03460 | Fanconi anemia pathway | 54 | 0.600988 | 0.000583 | 0.002676 | SDS/MAT1A/TAT/PSAT1/PHGDH/GCLM/GCLC/TST/LDHAL6A/LDHB/GOT1/LDHAL6B/SDSL/CTH/AHCY/BHMT2/MDH1/GOT2/MTAP |
| hsa04727 | GABAergic synapse | 75 | 0.545273 | 0.000662 | 0.002956 | GSTA3/GSTA1/GSTA2/CDKN2A/MSH3/MGST1/TOP2A/ERBB2/PIK3R2/GSTM2/GSTO2/CASP9/GSTM4/GSTM1/MSH2/GSTT2B/MDM2/GSTP1/ERCC1/GSTT2/MSH6/CDKN1A/TOP2B/POLH/MLH1/BRCA1/MGST2/AKT2/BCL2/TP53/BIRC5 |
| hsa04141 | Protein processing in endoplasmic reticulum | 167 | 0.468729 | 0.000653 | 0.002956 | UGT1A6/UGT2A1/UGT2B17/UGT2B15/UGT1A7/SORD/UGT1A1/UGDH/UGT2B7/RPEL1 |
| hsa05033 | Nicotine addiction | 28 | 0.70495 | 0.000703 | 0.003097 | NXF2B/NXF2/KPNA7/NCBP2L/IPO4/IPO11/NXT2/NDC1/KPNA5/EEF1A1/GLE1/NUP37/EIF4A3/NUP155/RAE1/SUMO4/THOC7/THOC3/NUP62/IPO13/AAAS/NUP133/UPF2/XPO5/UPF3B/NUP35/SNUPN/NUP88/IPO5/NUP107/PHAX/XPO1/NUP54/UPF3A/CSE1L/SAP18/KPNA1/SENP2/NUP153/NUP93/RAN/UPF1/MAGOH/RANBP2/NUP85/RNPS1/ALYREF/AHCTF1/TPR/SUMO1/EEF1A2/NXT1/NCBP2/NCBP1/SUMO2/DDX19B/XPOT/NUP98/SEC13/RANGAP1/UBE2I/NUP214/NUP210L/THOC1/IPO7/SRRM1/MAGOHB/DDX19A/THOC5 |
| hsa04310 | Wnt signaling pathway | 164 | 0.478397 | 0.000741 | 0.003223 | ABCB5/ABCA13/ABCA12/ABCB1/ABCD2/ABCB11/ABCB6/ABCC5/CFTR/ABCC3/ABCC8/ABCB4/ABCA5/ABCB9 |
| hsa05020 | Prion disease | 251 | 0.432712 | 0.000806 | 0.00346 | BAAT/SLC27A2/XDH/ABCD2/AGXT/ECI2/MPV17L/PEX6/PRDX5/PEX11G/IDH1/EPHX2/NUDT7/AMACR/IDH2/HACL1/PEX12/EHHADH/SLC25A17/SOD1/PEX7/PEX11A/PAOX/ABCD3/CROT/MVK/ACOT8/DECR2/PEX10/PEX16/NUDT13/ACOX3/HMGCL/NUDT12/PEX5/PEX13/DHRS4 |
| hsa04512 | ECM-receptor interaction | 88 | 0.53623 | 0.000864 | 0.003663 | BMP15/CYP11A1/CYP2J2/PLA2G4E/HSD17B2/ADCY2/STAR/CYP1B1/PLA2G4B/CYP17A1/LHB/INSR |
| hsa03420 | Nucleotide excision repair | 62 | 0.560149 | 0.000917 | 0.003839 | SPP1/IBSP/MEPE/COMP/DMP1/GP5/COL9A2/ITGB8/COL9A3/THBS4/HMMR/SV2B/ITGA11/ITGA7/ITGB4/FREM2/LAMB4/THBS2/GP9/COL4A6 |
| hsa04927 | Cortisol synthesis and secretion | 60 | 0.568201 | 0.00093 | 0.003846 | GRM4/PLPP2/DGKB/AVPR1A/LPAR3/PLA2G4E/SHC4/GRM7/CXCL8/GRM5/ADCY2/MS4A2/LPAR4/PDGFC/PIK3R2/PLA2G4B/AGPAT5/PTGFR/PLD1/DGKI/PIK3R6/DNM1/INSR |
| hsa02010 | ABC transporters | 45 | 0.616883 | 0.000963 | 0.003933 | CCNA1/H2BU1/CDKN2A/ATP6V0D2/CCR8/H4C15/H2BC5/H2BC18/H4C14/H2BC9/SCIN/CREB3L4/CDC20/PIK3R2/TRAF3/H2BC11/CCNA2/H4C11/H4C12/H2BC15/TRADD/CDK1/H2BC6/VDAC3/H4-16/GTF2E2/H2BC4/MDM2/CHEK1/GTF2H3/CDKN1A/TBPL1/CCR4/POLB/H2BC21/DDB1/HDAC1/RBL1/GSN/HDAC2/EGR3/YWHAE/DNAJA3/RANBP1/KAT2A/ATF6B/CHD4/SND1/TP53/CDKN1B/SNW1/CCNE2/PKM/HDAC11/GTF2A2/GTF2B/CREB3/IRF3/BAD/CASP3/TRAF2/MRPS18B/CREB3L2/YWHAQ/HNRNPK/CDK4/SCRIB/C3/UBE3A |
| hsa05032 | Morphine addiction | 78 | 0.540161 | 0.000975 | 0.003937 | TNNI3/COX6B2/MAPK10/SLC2A1/NDUFC2-KCTD14/ATP2A2/PIK3R2/ATP2A1/MPC2/PPIF/CMA1/INSR/GFPT1/PLN/GSR/VDAC3/NDUFAB1/TGFB3/ATP2A3/PRKCD/COX7A2L/CAMK2D/CPT1B/COX7C/NDUFA8/MMP9/TGFBR1/NDUFB3/SLC25A4/NDUFA4/NDUFC1/CAMK2B/NDUFB5/COL3A1/COX6B1/NDUFB7/ATP5PO/NDUFA5/ATP5F1A/UQCRC1/COX5B/UQCRB/PDK2/COX5A/SDHC/SMAD2/ATP5PF/UQCR11/PRKCG/NDUFS6/SLC2A4/NDUFA1/AKT2/NDUFA10/NDUFA13/ATP5MC1/NDUFB4/COX7B/ATP5MC2/UQCRC2/UQCRH/SDHA/NDUFB8/ATP5F1D/UQCRFS1/COX6A1/NDUFB1/ATP5PB/NDUFS7/NDUFA2/UQCR10/NDUFA7/CD36/SDHB/ATP5F1B/PARP1/COX6C/NDUFB9/NDUFB10/NDUFB6/SLC25A6/COX8A/MAPK8/GFPT2/NDUFS1/ATP5PD/AGT/SDHD/UQCRQ/COX7A2/NDUFS4/NDUFS2/COX4I1/ATP5MC3/NDUFS3/NDUFV3/SMAD3/VDAC1/NDUFB2/CYC1/NDUFS5/PDHB/GAPDH/NDUFS8/UQCRHL/NDUFA11/PIK3CB/MAPK9/NDUFA6/COL1A1/PIK3R3/NDUFV2/ATP5F1C/NDUFV1 |
| hsa04971 | Gastric acid secretion | 69 | 0.587657 | 0.001098 | 0.004379 | HSPA4L/MAPK10/DERL3/FBXO2/HSPH1/HSPBP1/HSP90AA1/HSPA1L/TUSC3/DNAJC10/TRAM1L1/PDIA4/HSP90AB1/FBXO6/YOD1/DNAJC5B/TMEM258/RNF5/SSR4/LMAN1/SKP1/SEC23B/SEC24D/UBE2D1/STT3A/HSPA8/MARCHF6/UBXN4/NFE2L2/OSTC/CAPN2/HSPA5/ERLEC1/CUL1/ATF6B/BCL2/DNAJA1/ERO1B/PDIA6/NSFL1C/HSP90B1/DNAJA2/MBTPS1/HERPUD1/MBTPS2/VCP/UBE2G1/MAN1B1/DAD1/XBP1/DNAJC5/DDOST/SEC61B/MAPK8/MOGS/SEC63/PRKCSH/TRAF2/RAD23B/LMAN2/DNAJB2/UBXN6/BAG1/CAPN1/SEC31A/CANX/UBXN1/SEC24B/DERL2/SEC61G/UBQLN2/PPP1R15A/SEC24C/SSR3/UBE2D3/SAR1B/HSPA1B/SIL1/UBE2D4/MAPK9/SEC13/UBE2G2/STUB1/DNAJC3/ERN1/TRAM1/HYOU1 |
| hsa04721 | Synaptic vesicle cycle | 71 | 0.557471 | 0.001123 | 0.004426 | ADH7/ALDH3B2/ADH6/LRTOMT/ALDH3A1/TAT/ADH1C/ALDH3B1/DCT/GOT1/MIF/TPO |
| hsa05322 | Systemic lupus erythematosus | 99 | 0.529454 | 0.001281 | 0.00499 | GUCY2F/RHO/CALML4/GUCA1A/CNGA1/CNGB1/PDE6B/GNAT2 |
| hsa00350 | Tyrosine metabolism | 34 | 0.639281 | 0.001309 | 0.005039 | GNG13/OR51B5/CNGA4/CNGA3/CALML4/OR56B4/OR7C1/CNGB1/OR10H5/OR3A3/OR52D1/PDE1A |
| hsa04913 | Ovarian steroidogenesis | 44 | 0.612215 | 0.001324 | 0.00504 | RET/MET/FGFR3/GLS2/SLC2A1/SLC7A5/GCK/SCO2/ERBB2/IDH1/PIK3R2/PDK1/HKDC1/MYC/IDH2/LDHAL6A/PFKP/SIRT3/LDHB |
| hsa05231 | Choline metabolism in cancer | 97 | 0.511022 | 0.001386 | 0.005215 | BMPR1B/HAMP/GREM1/INHBB/AMHR2/GDF7/GDF6/RGMA/HJV/BMP4/BMP7/BMP8B/ACVR1B/TGIF1/SMAD1/MYC/E2F5/GREM2/IGSF1/BAMBI/ACVR1/BMP8A/ACVR2B/ZFYVE16/TGFB3/SKP1/LEFTY2/NBL1/TGFBR1/LTBP1/SIN3A/THSD4/PPP2R1A/HDAC1/RBL1/RGMB/SMAD2/INHBE/HDAC2/ACVR1C/FBN1/TFDP1/CUL1 |
| hsa04744 | Phototransduction | 22 | 0.704007 | 0.001418 | 0.005277 | BIRC7/CBLC/KLHL13/FBXO2/TRIM32/AIRE/MID1/ANAPC4/CDC20/PIAS3/TRIM37/UBE2C/RPS27A/CUL7/FBXO4/NEDD4L/KEAP1/MDM2/DET1/ERCC8/SKP1/UBE2D1/PRPF19/DDB1/ANAPC10/ANAPC16/UBOX5/BRCA1/CDC16/BTRC/UBE2O/UBE2H/ANAPC13/ANAPC1/ELOB/CUL1/ANAPC5/CDC26/UBE2E3/UBA1/CUL3/UBB/SMURF1/DDB2/FANCL/FZR1/UBA52/ELOC/UBE2G1/UBE2M/MAP3K1/KLHL9/WWP2/ANAPC7/MGRN1/UBA3/UBE3A/PIAS4/UBE2Z/UBE3B/CUL2/UBE2E1/ANAPC15/UBE2D3/UBE2D4/CDC34/FBXW8/SAE1/ANAPC11/UBE2G2/STUB1/TRIP12/UBE2I/SKP2 |
| hsa05130 | Pathogenic Escherichia coli infection | 188 | 0.460819 | 0.001474 | 0.005425 | MMP7/WNT16/LGR5/WNT8B/CCN4/WNT4/WNT10A/MAPK10/RUVBL1/WNT7B/SFRP4/CXXC4/FZD3/RNF43/SERPINF1/APCDD1L/CCDC88C/WNT5B/CBY1/FZD9/FRAT1/WNT5A/FRZB/MYC/RAC3/FRAT2/BAMBI/VANGL1/RSPO1/NFATC4/SFRP1/APCDD1/FOSL1/WNT6 |
| hsa00512 | Mucin type O-glycan biosynthesis | 35 | 0.658487 | 0.001522 | 0.005541 | GSTA3/BMPR1B/GSTA1/GSTA2/CALML4/TRPV4/MAPK10/HSP90AA1/PRKAA2/NQO1/BMP4/MGST1/PIK3R2/GSTM2/GSTO2/RAC3/GSTM4/PLAT/ASS1/ACVR1/GSTM1/CALM1/HSP90AB1/DUSP1/FOS/CCL2/GPC1/ACVR2B/GSTT2B/KEAP1/GSTP1/TXN2/CALM2/GSTT2/MMP9/SUMO4/VCAM1 |
| hsa04120 | Ubiquitin mediated proteolysis | 140 | 0.483323 | 0.001643 | 0.005919 | GNG13/CALML4/GRIA2/LRTOMT/SLC6A3/DRD1/MAPK10/SLC18A1/SLC18A2/DRD5/GNB3/CREB3L4/GNG4/CACNA1B/PPP2R2C/DRD2/GNG3/GNB5/CACNA1A/DRD4/CALM1/FOS/GNG12/CALM2/CAMK2D/CAMK2B/PPP2R1A/PRKCG/DDC/PPP2R3B/GNG7/CALML6/AKT2/GRIA3/ATF6B |
| hsa05418 | Fluid shear stress and atherosclerosis | 134 | 0.484346 | 0.002005 | 0.007086 | CETN2/POLR2I/MNAT1/POLD2/RPA3/LIG1/CDK7/GTF2H5/POLD3/RFC5/RFC3/UVSSA/ERCC8/ERCC1/GTF2H3/PCNA/ERCC5/POLE2/DDB1/POLR2J/RPA2/POLE3/RPA1/ERCC2/XPC/RFC4/POLR2B/POLR2F/POLR2H/DDB2/ERCC4/RAD23B/POLR2E/ERCC3/ERCC6/RPA4/POLR2D |
| hsa04914 | Progesterone-mediated oocyte maturation | 98 | 0.489637 | 0.00201 | 0.007086 | UROC1/AOC1/ALDH3B2/ALDH3A1/ALDH3B1/HNMT/HDC/ALDH1B1 |
| hsa05415 | Diabetic cardiomyopathy | 182 | 0.454781 | 0.002039 | 0.007115 | SPDYE4/CPEB1/CALML4/ADCY2/MAD2L1/FBXO43/RPS6KA6/REC8/CCNB1/ANAPC4/CDC20/CCNB2/CALM1/CDK1/STAG3/AURKA/SMC1B/CALM2/CAMK2D/SKP1/AR/PLK1/CAMK2B/SMC3/SPDYE16/PPP2R1A/ANAPC10/ANAPC16/CDC16/CALML6/BUB1/YWHAE/BTRC/SMC1A/ANAPC13/ANAPC1/SPDYE5/CUL1/ANAPC5/CDC26/IGF1R/SGO1 |
| hsa03015 | mRNA surveillance pathway | 93 | 0.506386 | 0.002091 | 0.007222 | PLPP2/SLC44A5/SLC22A4/SLC44A4/DGKB/PLA2G4E/PCYT1B/MAPK10/SLC5A7/PDGFC/PIK3R2/PLA2G4B/PLD1/DGKI/RAC3/SLC22A3/SLC44A3/FOS/JMJD7-PLA2G4B |
| hsa00340 | Histidine metabolism | 22 | 0.692104 | 0.002242 | 0.007454 | GNG13/GABRP/GABRB3/ADCY2/ABAT/GLS2/GAD1/GNB3/CACNA1F/GNG4/GABRG3/GABRQ/CACNA1B/GNG3/GNB5/CACNA1A/GABRA2 |
| hsa04114 | Oocyte meiosis | 123 | 0.485141 | 0.002208 | 0.007454 | NXF2B/NXF2/WDR3/RPP38/REXO5/RPP40/FBL/NXT2/FCF1/GNL3/GAR1/NOB1/CSNK2A3/NHP2/NOP58/DKC1/REXO2/RPP25L/SNORD3B-2/MDN1/PWP2/CSNK2A1/BMS1/NOP56/NVL/UTP18/RIOK1/UTP14A/WDR43/NAT10/EFL1/XPO1/HEATR1/GTPBP4/AK6/SNU13/WDR75/EIF6/UTP4/GNL2/POP4/SNORD3B-1/RAN/MPHOSPH10/RPP30/XRN2/POP7/RRP7A/DROSHA/POP5/NOL6/UTP6/EMG1/CSNK2B/TBL3/SPATA5 |
| hsa04728 | Dopaminergic synapse | 127 | 0.480931 | 0.002225 | 0.007454 | PLPP2/PLA2G2D/DGKB/PLA2G4E/PCYT1B/CDS1/ETNK2/PLA2G12B/PLPP4/PLPP5/ETNK1/PCYT2/PLA2G4B/AGPAT5/PLD1/PLA2G6/DGKI/MBOAT1/PLA2G10/JMJD7-PLA2G4B/PLAAT2 |
| hsa04928 | Parathyroid hormone synthesis, secretion and action | 100 | 0.488806 | 0.002247 | 0.007454 | CKMT1A/AOC1/CKMT1B/GAMT/AZIN2/AGMAT/PYCR3/P4HA3/CKM/ALDH18A1/PYCR1/ALDH1B1/CKMT2/CKB/GOT1/L3HYPDH/ARG2/OAT/GOT2/P4HA1/ODC1/PYCR2/ALDH7A1/HOGA1/P4HA2/GATM/SMS |
| hsa04740 | Olfactory transduction | 67 | 0.579853 | 0.002335 | 0.007668 | KCNK2/CYP11A1/NR0B1/CACNA1G/ADCY2/STAR/NR4A1/CREB3L4/CACNA1F/CACNA1I/CYP17A1 |
| hsa05120 | Epithelial cell signaling in Helicobacter pylori infection | 69 | 0.561522 | 0.002581 | 0.008234 | GNG13/GABRP/PDE11A/GABRB3/DRD1/ADCY2/PDE1A/GNB3/GNG4/GABRG3/GABRQ/CACNA1B/GNG3/GNB5/CACNA1A/GABRA2 |
| hsa04340 | Hedgehog signaling pathway | 54 | 0.570716 | 0.002564 | 0.008234 | AK8/AK7/ALPG/ALPP/AK5/AK4 |
| hsa04510 | Focal adhesion | 200 | 0.435047 | 0.002547 | 0.008234 | NXF2B/NXF2/MSI2/NCBP2L/BCL2L2-PABPN1/PABPC1L/MSI1/PPP2R2C/NXT2/CSTF3/GLE1/PABPC4/EIF4A3/PABPC1/PAPOLA/PABPC3/CSTF2T/GSPT2/PPP2R1A/HBS1L/PPP2R3B/UPF2/CPSF3/UPF3B/CPSF6/CSTF1/SMG7/UPF3A/SYMPK/NUDT21/SAP18/PPP2R5E/RNMT/RNGTT/UPF1/MAGOH/PPP2R2B/RNPS1/ALYREF/PPP2R2A/DAZAP1/SSU72/CPSF1/PCF11/WDR33/NXT1/NCBP2/SMG6/NCBP1/FIP1L1/DDX19B/PPP2R3A |
| hsa05146 | Amoebiasis | 98 | 0.481433 | 0.002811 | 0.008883 | SPP1/IBSP/PAK5/COMP/MYLK3/MYLPF/SHC4/MAPK10/PAK6/MET/COL9A2/FLNC/ITGB8/COL9A3/THBS4/PDGFC/ERBB2/FLNB/PAK1/ITGA11/PIK3R2/ITGA7/ITGB4/RAC3 |
| hsa04657 | IL-17 signaling pathway | 79 | 0.527598 | 0.002843 | 0.008902 | ADPRS/TDP1/NEIL1/POLD2/LIG1/XRCC1/POLD3/RFC5/RFC3/SMUG1/PCNA/POLL/POLB/POLE2/LIG3/POLE3/FEN1/POLG2/NEIL2/RFC4/APEX1/PNKP/PARP2/PARP1/APTX/PARP3/UNG |
| hsa03018 | RNA degradation | 77 | 0.538691 | 0.002985 | 0.00926 | SLC6A2/SLC17A8/ATP6V0D2/SLC1A6/SLC6A3/ATP6V1B1/SLC18A1/SLC18A2/SLC6A7/ATP6V1C2/CACNA1B/STX1A/CACNA1A/DNM1/ATP6V1D/SLC6A9/RAB3A/UNC13A/SLC1A2/CLTA/CPLX3/ATP6V0E2/SNAP25/STX1B |
| hsa00270 | Cysteine and methionine metabolism | 48 | 0.60501 | 0.003196 | 0.009734 | GNG13/GABRP/SLC17A8/GRIA2/GABRB3/GRM5/MAPK10/ADCY2/NDUFC2-KCTD14/GNB3/CACNA1F/GNG4/GABRG3/GABRQ/CACNA1B/GNG3/GNB5/CACNA1A/GABRA2/ABHD6/NDUFAB1/FAAH/GNG12/NDUFA8/NDUFB3/NDUFA4/GABRE/NDUFC1/NDUFB5/NDUFB7/NDUFA5/PRKCG/NDUFS6/NDUFA1/GNG7/GRM1/GRIA3/NDUFA10/NDUFA13/NDUFB4/NDUFB8/KCNJ3/NDUFB1/NDUFS7/NDUFA2/NDUFA7/NDUFB9/NDUFB10/NDUFB6/MAPK8/ADCY5/NDUFS1 |
| hsa00650 | Butanoate metabolism | 24 | 0.651261 | 0.003187 | 0.009734 | TTR/SLC5A5/LRP2/ADCY2/SLC26A4/ALB/TSHR/GPX2/TG/CREB3L4/ATP1B1/GPX8/IYD/PDIA4/TPO/GSR |
| hsa03440 | Homologous recombination | 41 | 0.58894 | 0.003252 | 0.009815 | HHATL/LRP2/PTCH2/IHH/KIF3A/IQCE/EFCAB7/SCUBE2/HHAT/CDON |
| hsa03008 | Ribosome biogenesis in eukaryotes | 86 | 0.526148 | 0.003308 | 0.009893 | POLD2/RPA3/MCM2/LIG1/MCM4/RNASEH2A/PRIM1/POLD3/RFC5/RFC3/PRIM2/RNASEH2B/PCNA/POLE2/RPA2/POLE3/SSBP1/FEN1/RPA1/RFC4 |
| hsa04261 | Adrenergic signaling in cardiomyocytes | 141 | 0.458347 | 0.003352 | 0.009938 | GRIN3B/GRIA2/GRIN1/SLC6A3/DRD1/SLC18A1/SLC18A2/CREB3L4/DRD2/FOSB/CDK5R1/GRIN3A/GRM2 |
| hsa00730 | Thiamine metabolism | 14 | 0.740342 | 0.00354 | 0.010403 | ENO4/BTG4/TOB1/PABPC1L/HSPD1/WDR61/PFKP/DCP1B/ENO2/EXOSC2/LSM5/PNLDC1/PABPC4/EXOSC5/LSM7/PABPC1/PABPC3/EDC3/EXOSC4/LSM4/ZCCHC7/EXOSC1/HSPA9/EXOSC3/CNOT3/LSM1/ENO1/CNOT1/CNOT9/BTG2/MTREX/DHX36/PNPT1/CNOT10/DIS3L/PARN/PAN2/SKIV2L/DIS3/XRN2/C1D/LSM3/CNOT6/EXOSC7/EXOSC8/PAN3/EDC4/CNOT7 |
| hsa04918 | Thyroid hormone synthesis | 68 | 0.546568 | 0.003683 | 0.010711 | H2AC17/RUVBL1/H2AW/RUVBL2/BCL7A/MORF4L2/H2AC8/INO80B/MACROH2A2/ACTR6/MEAF6/SMARCB1/YEATS4/H2AB3/SS18/SMARCA4/KAT5/ACTL6A/DPF1/EPC2/HDAC1/SMARCA1/CHD3/POLE3/SMARCC1/MTA3/MORF4L1/HDAC2/H2AJ/H2AB2/CHD4/BRD8/EP400/MACROH2A1/NFRKB/ZNHIT1/SMARCA2/TRRAP/BCL7C/RBBP4/PBRM1/DMAP1/ACTR5/H2AX/BAZ1A/TFPT/VPS72/BAZ1B/MTA1/H2AC6/BPTF/MRGBP/INO80/H2AC18/H2AC19/ARID1B/INO80C/INO80E/SMARCE1/CDK2AP2/ACTR8 |
| hsa05030 | Cocaine addiction | 47 | 0.571115 | 0.003709 | 0.010711 | CALML4/ADCY2/SIK1/SLC2A1/PRKAA2/CREB3L4/FBP2/GCK/PHKA1/PHKG2/LDHAL6A/PFKP/LDHB/CALM1/PRKAB1/PPP4R3B/LDHAL6B/CALM2/CAMK2D/CPT1B/CAMK2B/PFKFB1/CALML6/AKT2/PRMT1/G6PC3 |
| hsa04932 | Non-alcoholic fatty liver disease | 144 | 0.449588 | 0.00384 | 0.010994 | GRIN3B/SPTBN2/GRIA2/SLC1A6/GRIN1/MAPK10/FGF14/ATP2A2/PIK3R2/ATP2A1/OMA1/PPIF/CACNA1A/KCNC3/TWNK/VDAC3/ATXN10/MCU/ATP2A3/KCND3/GRIN3A/SEM1/SLC25A4/ATXN2/RB1CC1/VLDLR/RELN/TBPL1/KAT5/NOP56/PSMB5/PRKCG/WIPI1/GRM1/AKT2/PSMB7/GRIA3/PSMA5/PSMD4/PSMC4/XBP1/GTF2B/PSMC6/WIPI2/SLC25A6/MAPK8/RYR1/TRAF2/PSMD14/PUM2/OPA1/AFG3L2/ULK2/VDAC1/BECN1/ATG2B/PSMB1/ATG14 |
| hsa05224 | Breast cancer | 137 | 0.462584 | 0.003904 | 0.011085 | MMP13/CCL11/CXCL6/MAPK15/CXCL1/CXCL8/MAPK10/HSP90AA1/TRAF4/TRAF3/IL17RB/CXCL2/FOSB/HSP90AB1/TRADD/FOS/FOSL1/CCL2/MMP9/IKBKE/TRAF3IP2 |
| hsa04978 | Mineral absorption | 53 | 0.567305 | 0.00402 | 0.011317 | ATP2B3/MYLK3/ADRA2A/CALML4/IRS4/BDKRB2/CNGA1/ADRA1B/ADCY2/CNGB1/ATP2B2/ATP2A2/CREB3L4/CACNA1F/KCNMA1/ADRA2B/ATP1B1/ATP2A1/NPR2/KCNMB1/PPIF/PIK3R6/INSR/PLN/MEF2B/CALM1/NFATC4/PDE2A |
| hsa00534 | Glycosaminoglycan biosynthesis - heparan sulfate / heparin | 23 | 0.667268 | 0.004156 | 0.01147 | GNG13/MMP13/RLN2/RXFP3/SHC4/MAPK10/ADCY2/GNB3/CREB3L4/GNG4/PIK3R2/RLN1/GNG3/GNB5 |
| hsa03410 | Base excision repair | 44 | 0.584809 | 0.004177 | 0.01147 | ALOX15/CYP2J2/PLA2G2D/PLA2G4E/PLA2G12B/PTGR1/PTGES3/GGT5/PTGES/CYP2B6/PLA2G4B/EPHX2/PTGR2/CYP2C8/PLA2G6/GPX4/PLA2G10/CYP2U1/CBR3/CYP2E1/JMJD7-PLA2G4B/PLAAT2 |
| hsa05017 | Spinocerebellar ataxia | 134 | 0.470259 | 0.004169 | 0.01147 | KCNK2/ATP2B3/CACNG6/TNNI3/CALML4/AGTR2/ADRA1B/ADCY2/KCNE1/ATP2B2/PPP1R1A/CACNG8/ATP2A2/CREB3L4/CACNA1F/ATP1B1/ATP2A1/PPP2R2C/PIK3R6/PLN/CALM1 |
| hsa03430 | Mismatch repair | 23 | 0.666068 | 0.004271 | 0.011633 | ADH7/ACSBG1/ADH6/ADH1C/ECI2/HADH/ALDH1B1/EHHADH/CYP2U1/ACADSB/ECI1/CPT1B/ACADM/ALDH7A1/ACOX3/GCDH/ECHS1 |
| hsa04922 | Glucagon signaling pathway | 92 | 0.495411 | 0.004336 | 0.011714 | WNT16/TNFSF11/WNT8B/SHC4/WNT4/WNT10A/WNT7B/HEY2/FZD3/FGF16/FGF20/WNT5B/FZD9/ERBB2/PIK3R2/FGF5/FRAT1/WNT5A/MYC/FRAT2/JAG2/FGF7/GADD45G/FOS/JAG1/WNT6 |
| hsa04936 | Alcoholic liver disease | 122 | 0.459701 | 0.00438 | 0.011739 | ATP2B3/TRPV6/SLC34A3/STEAP1/STEAP2/ATP2B2/MT1F/SLC46A1/ATP1B1 |
| hsa04926 | Relaxin signaling pathway | 124 | 0.458698 | 0.004577 | 0.012092 | ALOX15/CYP2J2/PLA2G2D/PLA2G4E/PLA2G12B/PLA2G4B/CYP2C8/PLA2G6/PLA2G10/CYP2E1/JMJD7-PLA2G4B/PLAAT2 |
| hsa04150 | mTOR signaling pathway | 151 | 0.433366 | 0.004584 | 0.012092 | SPDYE4/CCNA1/CPEB1/MAPK10/ADCY2/MAD2L1/HSP90AA1/RPS6KA6/CCNB1/ANAPC4/PIK3R2/CCNA2/CCNB2/HSP90AB1/CDK1/AURKA/PLK1/SPDYE16/ANAPC10/ANAPC16/CDC16/AKT2/BUB1/KIF22/ANAPC13/ANAPC1/SPDYE5/ANAPC5/CDC26/CDC25A |
| hsa04371 | Apelin signaling pathway | 131 | 0.453691 | 0.004728 | 0.012375 | RAD51B/RAD54L/POLD2/XRCC2/RPA3/EME1/BRIP1/ABRAXAS1/RAD50/BLM/BRCC3/POLD3/BABAM2/SEM1/RAD51/RAD51C/RPA2/SSBP1/BRCA1/RBBP8/RPA1/TOP3A/RAD52/PALB2/TOP3B/RAD54B |
| hsa04540 | Gap junction | 84 | 0.504515 | 0.004872 | 0.012554 | BDH1/ABAT/ACSM1/GAD1/ALDH5A1/ACSM2A/HADH/OXCT2/ACSM2B/EHHADH/HMGCS2 |
| hsa04218 | Cellular senescence | 151 | 0.432298 | 0.004857 | 0.012554 | SDS/PSPH/PSAT1/AGXT/GAMT/PHGDH/GCAT/AMT/SRR/CHDH/SDSL/CTH |
| hsa04723 | Retrograde endocannabinoid signaling | 128 | 0.471625 | 0.004984 | 0.012746 | CHST9/MGAT4D/B4GALNT4/B4GALNT3/CHST8/FUT8/TUSC3/ALG1/TMEM258/ALG13/MGAT4C/STT3A/OSTC/ALG3/MAN1B1/B4GALT2/DAD1/ALG11/DDOST/B4GALT1 |
| hsa00564 | Glycerophospholipid metabolism | 94 | 0.501333 | 0.00573 | 0.014541 | ADH7/ADH6/ADH1C/ACYP1/PC/ALDH1B1/DLAT/LDHAL6A/LDHB/ACSS1/LDHAL6B |
| hsa05217 | Basal cell carcinoma | 62 | 0.51094 | 0.00609 | 0.01534 | KRT17/KRT15/KRT40/CALML4/KRT14/SHC4/ADCY2/HSP90AA1/HSPA1L/CREB3L4/PIK3R2/KRT23/TGFA/CALM1/HSP90AB1/FOS/FKBP4/PRKCD/CALM2/ESR2/MMP9 |
| hsa00330 | Arginine and proline metabolism | 48 | 0.57748 | 0.006212 | 0.01553 | HTR2C/DRD1/GRM5/ADCY2/TUBB8/TUBAL3/TUBB4B/TUBB3/PDGFC/TUBB4A/HTR2A/DRD2 |
| hsa00590 | Arachidonic acid metabolism | 55 | 0.5604 | 0.006687 | 0.016374 | ADH7/ADH6/CXCL1/IL12A/CXCL8/MAPK10/ADH1C/IL12B/PRKAA2/C4B/C4A/TRAF3/ALDH1B1/C5AR1/CXCL2/TRADD/PRKAB1/SCD5/CYP2E1/CPT1B/IKBKE/CXCL3/MAP2K3/LY96/ACADM/C5/ALDH7A1/ACOX3/AKT2 |
| hsa00071 | Fatty acid degradation | 43 | 0.575646 | 0.006733 | 0.016374 | COX6B2/CXCL8/MAPK10/PRKAA2/NDUFC2-KCTD14/PIK3R2/INSR/PRKAB1/FOS/CYP2E1/NDUFAB1/COX7A2L/COX7C/NDUFA8/NDUFB3/NDUFA4/NDUFC1/NDUFB5/COX6B1/NDUFB7/NDUFA5/UQCRC1/COX5B/UQCRB/CEBPA/COX5A/SDHC/UQCR11/NDUFS6/MLXIPL/NDUFA1/AKT2/NDUFA10/NDUFA13/NDUFB4/COX7B/UQCRC2/UQCRH/SDHA/IL6R/NDUFB8/UQCRFS1/COX6A1/NDUFB1/NDUFS7/NDUFA2/UQCR10/NDUFA7/NR1H3/XBP1/SDHB/COX6C/NDUFB9/ADIPOR1/NDUFB10/NDUFB6/COX8A/MAPK8/NDUFS1/CASP3/TRAF2/CASP7/SDHD/UQCRQ/COX7A2/NDUFS4/NDUFS2/COX4I1/NDUFS3/NDUFV3/LEP/MLXIP/NDUFB2/CYC1/NDUFS5/NDUFS8/UQCRHL/NDUFA11/PIK3CB/MAPK9/NDUFA6/PIK3R3/NDUFV2 |
| hsa04713 | Circadian entrainment | 91 | 0.479768 | 0.006745 | 0.016374 | NAT8L/AGXT/ABAT/GLS2/ADSS1/GAD1/GPT2/ALDH5A1/ASL/ASS1/GFPT1/GOT1/ADSS2/PPAT/GOT2/NIT2/CAD/IL4I1/ADSL |
| hsa04022 | cGMP-PKG signaling pathway | 155 | 0.44406 | 0.006689 | 0.016374 | GNG13/SPP1/MYLK3/CALML4/ADCY2/PRKAA2/GNB3/CDH1/GNG4/RYR3/PLIN1/GNG3/GNB5/RPS6/LIPE/PIK3R6/PLAT/MEF2B/CALM1/EGR1/MAP1LC3A/PRKAB1/JAG1/NRF1/GNG12/CALM2/TGFBR1/ADCY10/MAP1LC3B2/SMAD2/GNG7/TFAM/CALML6/SLC8A3/AKT2 |
| hsa00250 | Alanine, aspartate and glutamate metabolism | 34 | 0.590177 | 0.006915 | 0.016666 | WNT16/WNT8B/WNT4/ATP6V1B1/WNT10A/WNT7B/FZD3/PRKAA2/RPS6KA6/WNT5B/ATP6V1C2/SLC7A5/FZD9/DEPTOR/PIK3R2/WNT5A/RRAGD/WDR24/RPS6/INSR/ATP6V1D/MIOS |
| hsa03030 | DNA replication | 36 | 0.597998 | 0.007242 | 0.017205 | CHIT1/UGDH/GCK/NANS/UAP1/HKDC1/GFPT1/GALK2/MPI/CYB5R1/PMM1/HK1/NPL/GNE/GMDS/GMPPA/GPI/PMM2/GNPDA2/FCSK/GMPPB/GNPDA1/CMAS/GFPT2 |
| hsa00565 | Ether lipid metabolism | 46 | 0.564689 | 0.00724 | 0.017205 | CCNA1/CDKN2A/CALML4/TRPV4/CXCL8/CCNB1/ZFP36L1/PIK3R2/RAD9A/CCNA2/HIPK1/MYC/E2F5/PPID/RAD50/CCNB2/CALM1/NFATC4/GADD45G/CDK1/ATR/VDAC3/MCU/MYBL2/TGFB3/MDM2/CHEK1/CALM2/TGFBR1/TRAF3IP2/SLC25A4/GADD45A/MAP2K3/CDKN1A/RBL1/SMAD2/CALML6/AKT2/CAPN2/BTRC/NFATC2/CDC25A/TP53/ZFP36L2/LIN9/RRAS2 |
| hsa00591 | Linoleic acid metabolism | 28 | 0.624768 | 0.008008 | 0.018893 | GNG13/CALML4/GRIA2/CACNA1G/GRIN1/ADCY2/RASD1/GNB3/GNG4/RYR3/CACNA1I/GNG3/GNB5/ADCYAP1/CALM1/FOS/GNG12/CALM2/CAMK2D/PER2/CAMK2B/ADCY10/PRKCG/GNG7/RPS6KA5/CALML6 |
| hsa05031 | Amphetamine addiction | 64 | 0.540303 | 0.008311 | 0.019201 | PARS2/WARS2/IARS1/EARS2/FARS2/PSTK/TARS2/IARS2/FARSB/LARS2/EPRS1/FARSA/QRSL1/MTFMT/VARS2/NARS1/AARS2/GATB/QARS1/LARS1/NARS2/VARS1/RARS1/SEPSECS/DARS2/AARS1/SARS2/DARS1 |
| hsa00513 | Various types of N-glycan biosynthesis | 42 | 0.567633 | 0.0083 | 0.019201 | MYLK3/CALML4/PLA2G2D/AVPR1A/PLA2G4E/ADRA1B/ADCY2/CALCB/ADORA2B/CALCA/PLA2G12B/RAMP1/ACTG2/CACNA1F/KCNMA1/ADM2/PLA2G4B/NPR2/PLA2G6/KCNMB1/CALM1/PLA2G10/MYH11/JMJD7-PLA2G4B/PRKCD/MYH14/CALM2/NPPA |
| hsa04670 | Leukocyte transendothelial migration | 107 | 0.458247 | 0.008203 | 0.019201 | OGDHL/AGXT/GPT2/IDH1/DLAT/IDH2/GOT1/ABHD14A-ACY1/GOT2/ACO2/IDH3A/DBT/BCKDHB |
| hsa00620 | Pyruvate metabolism | 43 | 0.565158 | 0.008456 | 0.019403 | MMP13/CYP24A1/TNFSF11/SLC34A3/CASR/NR4A2/ADCY2/PTHLH/CREB3L4/CYP27B1/MMP16/PLD1 |
| hsa00260 | Glycine, serine and threonine metabolism | 37 | 0.592721 | 0.008727 | 0.019755 | SERPINB3/MUC2/CXCL1/IL12A/CXCL8/GNA14/IL12B/IL10/CTSG/PIK3R2/C8A/C9/LAMB4/HSPB1/CXCL2/C8B/RAB7B/CD1A/ARG2/COL4A6 |
| hsa05150 | Staphylococcus aureus infection | 64 | 0.538403 | 0.008724 | 0.019755 | SPP1/IBSP/CCNA1/COMP/WNT16/HES2/ATP6V0D2/WNT8B/WNT4/ATP6V1B1/WNT10A/WNT7B/COL9A2/HEY2/FZD3/ITGB8/COL9A3/THBS4/WNT5B/ATP6V1C2/CREB3L4/FZD9/HES6/ITGA11/PIK3R2/ITGA7/TRAF3/WNT5A/CCNA2/ITGB4/PPP2R2C/LAMB4/ATP6V1D/TERT/THBS2/TRADD/COL4A6/ATR/JAG1/WNT6 |
| hsa04915 | Estrogen signaling pathway | 114 | 0.473361 | 0.008949 | 0.020121 | GRIN3B/CALML4/GRIA2/GRIN1/SLC6A3/DRD1/SLC18A1/SLC18A2/CREB3L4/STX1A/FOSB/CALM1/FOS/GRIN3A/CALM2/CAMK2D/CAMK2B/HDAC1/PRKCG/DDC/HDAC2/CALML6/GRIA3/ATF6B |
| hsa04270 | Vascular smooth muscle contraction | 124 | 0.441897 | 0.009013 | 0.020128 | PLA2G7/PLPP2/PLA2G2D/PLA2G4E/PLA2G12B/GDPD1/PLA2G4B/PLD1/ENPP6/PLA2G6/PAFAH1B3/PLA2G10/JMJD7-PLA2G4B/PLAAT2/UGT8/CEPT1/ENPP2 |
| hsa04916 | Melanogenesis | 96 | 0.462948 | 0.009135 | 0.020267 | WDR5B/CBX8/L3MBTL2/L3MBTL1/ASXL3/SFMBT1/SCML2/PHC1/FBRS/LCOR/SKP1/AURKB/UBE2D1/L3MBTL4/HDAC1/ASXL2/USP45/EZH2/HDAC2/EED/TFDP1/KDM2B/RING1/AEBP2/PCGF1/PCGF6/CBX6/FBRSL1/MGA/SFMBT2/PCGF2/RBBP4/BCORL1/CBX3/RNF2/CSNK2B/USP16/UBE2D3/UBE2D4/SCMH1/SCML1/BMI1/OGT/WDR5/FOXK2/E2F6/RBBP7/SUZ12/UBE2D2/MBD6/BAP1/PHC3/CBX2 |
| hsa00500 | Starch and sucrose metabolism | 32 | 0.605033 | 0.009219 | 0.020317 | HS3ST2/HS6ST2/HS3ST5/HS3ST1/HS3ST3B1/HS6ST3/HS6ST1 |
| hsa04930 | Type II diabetes mellitus | 41 | 0.559754 | 0.009453 | 0.020563 | MSH3/EXO1/POLD2/RPA3/LIG1/MSH2/POLD3/RFC5/RFC3/MSH6/PCNA/RPA2/PMS2/MLH1/SSBP1/RPA1/RFC4 |
| hsa05165 | Human papillomavirus infection | 308 | 0.384943 | 0.009445 | 0.020563 | KRT17/KRT15/KRT40/KRT14/FGG/HLA-DRB1/IL10/CFB/C4B/PLG/C4A/KRT23/CFI/C5AR1 |
| hsa04064 | NF-kappa B signaling pathway | 101 | 0.466429 | 0.01036 | 0.022392 | CR2/IL5RA/GP5/IL7/CD24/IL11/DNTT/MS4A1/HLA-DRB1/CD19/CD8B2 |
| hsa00561 | Glycerolipid metabolism | 54 | 0.528858 | 0.010883 | 0.02337 | TAF1L/MNAT1/TAF4B/CDK7/TAF6/GTF2H5/GTF2E2/GTF2IRD1/GTF2F1/TAF7/GTF2H3/TBPL1/GTF2I/TAF9/ERCC2/TAF1/TAF15/GTF2F2/TAF12/GTF2A2/GTF2B |
| hsa04640 | Hematopoietic cell lineage | 92 | 0.472565 | 0.011322 | 0.024159 | AK9/NT5E/ENTPD3/NME7/UPP2/ENPP3/NT5C1B/NME1-NME2/NME1/NT5M/RRM2/TYMS/NME6/RRM2B/TYMP/TK1/DCTPP1/DPYS/UMPS/CAD/RRM1/DHODH/CTPS2/NUDT2/ENTPD6/UCK1/NT5C3B/NME2/NME3/DCTD/DTYMK/NT5C |
| hsa05205 | Proteoglycans in cancer | 200 | 0.409048 | 0.011543 | 0.024473 | WNT16/WNT8B/ERBB4/WNT4/WNT10A/WNT7B/MET/IHH/IGF2/FZD3/IL12B/FLNC/WNT5B/TWIST1/HOXD10/HPSE/FZD9/ERBB2/FLNB/PAK1/PIK3R2/ERBB3/WNT5A/PDCD4/HPSE2/MYC/RPS6/TWIST2/WNT6/TFAP4/GPC1/MDM2/EZR/CAMK2D/ANK2/MMP9/ANK3/CAMK2B/CDKN1A/EIF4B/SDC4/SMAD2/GPC3/PRKCG/SMO |
| hsa04672 | Intestinal immune network for IgA production | 40 | 0.574524 | 0.011875 | 0.024853 | WNT16/WNT8B/WNT4/WNT10A/WNT7B/PTCH2/FZD3/WNT5B/BMP4/FZD9/WNT5A |
| hsa03022 | Basal transcription factors | 42 | 0.553227 | 0.011947 | 0.024853 | PIGR/TNFRSF13B/HLA-DRB1/IL10/TNFRSF13C/TNFRSF17/AICDA/CCL28 |
| hsa04925 | Aldosterone synthesis and secretion | 91 | 0.4665 | 0.012019 | 0.024853 | ATP2B3/CYP11A1/CALML4/NR4A2/CACNA1G/ADCY2/STAR/ATP2B2/NR4A1/CREB3L4/CACNA1F/ATP1B1/CACNA1I/LIPE/CALM1/PDE2A/CYP21A2/CALM2/CAMK2D/NPPA/CAMK2B |
| hsa04810 | Regulation of actin cytoskeleton | 215 | 0.393828 | 0.011881 | 0.024853 | CLDN8/CLDN16/CLDN10/CLDN9/MYLPF/CLDN1/CLDN19/CLDN3/CLDN20/CLDN4/CTNNA3/PIK3R2 |
| hsa00970 | Aminoacyl-tRNA biosynthesis | 44 | 0.561429 | 0.01236 | 0.025243 | ABAT/HADH/OXCT2/ALDH1B1/EHHADH/HMGCS2/HIBADH/MCEE/ACADSB/ACAD8/ACADM/ALDH7A1/DBT/BCKDHB/HMGCL/ECHS1/ACSF3/MMUT/IL4I1/PCCB/ALDH6A1/ACAT1/AACS/ACADS/PCCA/IVD/HSD17B10/HMGCS1/BCKDHA/HADHA/ACAA1/HIBCH/MCCC1/ACAT2/MCCC2/OXCT1/ALDH3A2 |
| hsa04917 | Prolactin signaling pathway | 66 | 0.497118 | 0.012433 | 0.025243 | ABCB11/LRP2/APOE/STAR/LPA/CIDEB/SOAT2/PLTP/SOAT1/VDAC3/ABCG8/LIPA |
| hsa04921 | Oxytocin signaling pathway | 142 | 0.42493 | 0.012298 | 0.025243 | TNFSF11/EDARADD/CXCL1/CXCL8/EDAR/EDA2R/CCL13/CCL19/TNFRSF13C/CARD14/BLNK/TRAF3/CXCL2/TNFRSF11A/GADD45G/TRADD/CSNK2A3/GADD45A/VCAM1/CSNK2A1/CXCL3/LY96/CCL21 |
| hsa00120 | Primary bile acid biosynthesis | 15 | 0.68192 | 0.012931 | 0.026095 | B3GNT3/B3GALT5/FUT2/B3GNT5/B3GALT1/B3GALT2/ABO/B4GALT4/A4GALT |
| hsa03320 | PPAR signaling pathway | 66 | 0.494999 | 0.013255 | 0.02659 | NXF2B/TMPRSS4/NXF2/IL12A/CXCL8/TPSD1/KPNA7/TPSAB1/BCL2L2-PABPN1/HLA-DRB1/IL12B/TPSB2/PLG/PIK3R2/TRAF3/CASP9 |
| hsa00520 | Amino sugar and nucleotide sugar metabolism | 48 | 0.544376 | 0.013766 | 0.027305 | AQP2/DYNC2H1/CREB3L4/DYNLL1/DYNC2LI1 |
| hsa00603 | Glycosphingolipid biosynthesis - globo and isoglobo series | 14 | 0.697906 | 0.013812 | 0.027305 | PLPP2/DGKB/MOGAT2/PLPP4/PLPP5/CEL/DGAT2/TKFC/AGPAT5/ALDH1B1/DGKI/GK/MBOAT1 |
| hsa00240 | Pyrimidine metabolism | 55 | 0.532057 | 0.013856 | 0.027305 | ATG9B/IRS4/MAPK10/PRKAA2/C9orf72/VMP1/DEPTOR/DAPK1/PIK3R2/RRAGD/PRAP1/TAX1BP1/RAB7B/IGBP1/RPS27A/MAP1LC3A/PRKCD/RRAGA/RB1CC1/ATG10/GABARAP/MAP1LC3B2/SUPT20H/MTMR3/WIPI1/AKT2/ATG4C/MLST8/BCL2/STK11/NBR1/IGF1R/RRAS2/VAMP8/WDR45B/UBB/EIF2AK4/PLEKHM1/UBA52/VPS33A/WIPI2/VPS41/MAPK8/ATG16L1/BAD/VPS11/MAP3K7/VPS16/ATG5/ULK2/CTSB/BECN1/GABARAPL2/ATG2B/STX7/ATG14/RPTOR/ATG4A/RPS6KB1/PIK3CB/CTSL/MAPK9/PIK3C3/DDIT4/HMGB1/PIK3R3/ERN1/BNIP3/RAF1/TSC1/RRAGB |
| hsa00430 | Taurine and hypotaurine metabolism | 15 | 0.675914 | 0.014381 | 0.028173 | IRS4/CACNA1G/MAPK10/CACNA1E/GCK/ABCC8/PIK3R2/CACNA1B/HKDC1/CACNA1A/INSR |
| hsa00280 | Valine, leucine and isoleucine degradation | 47 | 0.53316 | 0.014767 | 0.028761 | CP/ALOX15/STEAP3/GCLM/GCLC/GPX4/SLC11A2/MAP1LC3A/VDAC3 |
| hsa04979 | Cholesterol metabolism | 47 | 0.532134 | 0.015198 | 0.02926 | OXTR/CACNG6/MYLK3/CALML4/PLA2G4E/ADCY2/OXT/PRKAA2/CACNG8/KCNJ14/CACNA1F/PLA2G4B/RYR3/NPR2/PIK3R6/CD38/CALM1/NFATC4/KCNJ2/PRKAB1/FOS/TRPM2/JMJD7-PLA2G4B/CALM2/CAMK2D/NPPA/CAMK2B/CDKN1A/EEF2K/CACNB3/PRKCG/EEF2/CALML6 |
| hsa00310 | Lysine degradation | 60 | 0.491431 | 0.015173 | 0.02926 | AMY1B/AMY1C/AMY1A/GYG2/PGM2L1/ENPP3/GCK/HKDC1/AMY2B/AMY2A |
| hsa04962 | Vasopressin-regulated water reabsorption | 42 | 0.543729 | 0.016037 | 0.0307 | UPK3A/CDKN2A/CXCL8/FGFR3/CDH1/ERBB2/DAPK1/MYC/TYMP/MDM2/MMP9/CDKN1A |
| hsa04216 | Ferroptosis | 40 | 0.557191 | 0.016315 | 0.031054 | CYP24A1/SQLE/CEL/SOAT2/CYP27B1/SOAT1/LIPA/TM7SF2/HSD17B7/NSDHL |
| hsa00600 | Sphingolipid metabolism | 51 | 0.525458 | 0.016799 | 0.031795 | SST/IRS4/GHRL/SHC4/MAPK10/ADCY2/MRAP2/CREB3L4/CACNA1F/PIK3R2 |
| hsa05219 | Bladder cancer | 40 | 0.552369 | 0.017869 | 0.033629 | WNT16/CALML4/WNT8B/WNT4/WNT10A/ADCY2/WNT7B/FZD3/WNT5B/CREB3L4/FZD9/DCT/WNT5A |
| hsa05132 | Salmonella infection | 244 | 0.380467 | 0.018021 | 0.033727 | ATG9B/MAPK10/TOMM20L/MTX2/TAX1BP1/RAB7B/RPS27A/MAP1LC3A/TOMM70/CSNK2A3/FUNDC1/MTX3/MTX1/CCZ1B/CSNK2A1/CCZ1/GABARAP/MAP1LC3B2/NLRX1/USP8/SAMM50/MITF/TP53/NBR1/RRAS2/UBB/SMURF1/TOMM7/TOMM20/VCP/UBA52/PHB2/FIS1/RHOT2/ARIH1/MAPK8/FKBP8/MFN1/TRAF2/RABGEF1/TOMM40/OPA1/BECN1/GABARAPL2/MUL1/MON1A/CSNK2B/BNIP3L/MAPK9/MARCHF5/CITED2/PGAM5/USP30/BNIP3 |
| hsa04935 | Growth hormone synthesis, secretion and action | 112 | 0.442137 | 0.018131 | 0.033744 | CCNA1/HNF4A/IRS4/PRKAA2/CREB3L4/FBP2/CFTR/PIK3R2/CCNA2/PPP2R2C/LIPE/PFKP/INSR/PRKAB1/SCD5/CPT1B/HMGCR/STRADA/RAB11B/PFKFB1/EEF2K/PPP2R1A/PPP2R3B/EEF2/SLC2A4/AKT2/G6PC3/STK11/CAB39L/IGF1R/PPP2R5E/CD36/CREB3/ADIPOR1/ELAVL1/MAP3K7/PPP2R2B/CREB3L2 |
| hsa04152 | AMPK signaling pathway | 116 | 0.436521 | 0.018275 | 0.033824 | CALML4/IRS4/SHC4/MAPK10/PRKAA2/FBP2/GCK/PHKA1/PIK3R2/PTPRF/PHKG2/HKDC1/RPS6/LIPE/INSR/PPP1R3B/CALM1/PRKAB1/PRKAR1A/PPP1R3F/PRKAR2A/CALM2/HK1/PPP1R3D/SLC2A4/CALML6/AKT2/G6PC3 |
| hsa04975 | Fat digestion and absorption | 32 | 0.581375 | 0.019219 | 0.035309 | RORB/PRKAA2/NPAS2/RORC/CRY2/PRKAB1/NR1D1/SKP1/CRY1/PER2/BHLHE40/BHLHE41/BTRC/CUL1/DBP/NR1D2 |
| hsa05323 | Rheumatoid arthritis | 84 | 0.465636 | 0.019288 | 0.035309 | PAK5/C6/MYLK3/BDKRB1/MYLPF/BDKRB2/PFN2/PAK6/FGFR3/FGF16/LPAR4/FGF20/ITGB8/SCIN/PDGFC/PAK1/ACTR3B/ITGA11/PIK3R2/ITGA7/FGF5/ITGB4/C8A/C9/RAC3/PFN4/FGF7/ARHGEF4/C8B/IQGAP3/MYH11/ENAH/MYH14/EZR/GNG12/FGF9 |
| hsa04215 | Apoptosis - multiple species | 31 | 0.568956 | 0.020422 | 0.037182 | BAAT/CH25H/CYP39A1/AMACR/SLC27A5/CYP8B1/CYP46A1/ACOT8 |
| hsa00100 | Steroid biosynthesis | 20 | 0.647181 | 0.021356 | 0.038463 | TNFSF11/SHC4/ELF5/MAPK10/GCK/PIK3R2/SOCS7/SOCS6/CYP17A1/LHB/TNFRSF11A/FOS |
| hsa05160 | Hepatitis C | 138 | 0.413763 | 0.021293 | 0.038463 | CDKN2A/ALK/RET/MET/ERBB2/FHIT/PIK3R2/TGFA/CASP9/GADD45G/RARB |
| hsa00601 | Glycosphingolipid biosynthesis - lacto and neolacto series | 25 | 0.604964 | 0.02209 | 0.039574 | FABP6/SLC27A2/ACSBG1/SLC27A6/FABP7/AQP7/PLTP/PLIN1/SLC27A5/GK/EHHADH/HMGCS2/CYP8B1/SCD5 |
| hsa05223 | Non-small cell lung cancer | 72 | 0.477074 | 0.022401 | 0.039916 | BBOX1/KMT5C/SMYD3/MECOM/HADH/ALDH1B1/SMYD2/PRDM16/EHHADH/HYKK/KMT5A/SETDB2/EZH2/ALDH7A1/GCDH/ECHS1/NSD3/SUV39H2/AADAT/SUV39H1/ACAT1/EHMT2/SETD2/PIPOX/SETMAR/KMT5B/SETD1B/HADHA/SETD1A/TMLHE/SETDB1/ACAT2 |
| hsa05164 | Influenza A | 148 | 0.422524 | 0.02269 | 0.040218 | PLPP2/PLA2G2D/MOGAT2/MTTP/PLA2G12B/CEL/DGAT2 |
| hsa04720 | Long-term potentiation | 63 | 0.484298 | 0.024602 | 0.043378 | CCL11/MS4A2/HLA-DRB1/IL10 |
| hsa05310 | Asthma | 21 | 0.61572 | 0.025414 | 0.044575 | ATP4B/ATP6V0D2/ATP6V1B1/ATP6V1C2/ATP6V1D/CLCNKB |
| hsa04140 | Autophagy - animal | 138 | 0.411005 | 0.025737 | 0.044905 | CALML4/GRIA2/GRIN1/GRM5/RPS6KA6/PPP1R1A |
| hsa04910 | Insulin signaling pathway | 128 | 0.431707 | 0.026155 | 0.045399 | BAAT/GGT6/GAD1/GGT5/FMO3 |
| hsa05417 | Lipid and atherosclerosis | 194 | 0.388118 | 0.02868 | 0.049524 | CXCL6/TNFSF11/ATP6V0D2/CXCL1/IL11/ATP6V1B1/CXCL8/HLA-DRB1/ATP6V1C2/CTSK/CTLA4/ATP6V1D/CXCL2/TNFRSF11A/FOS/CCL2/TGFB3 |
| hsa03020 | RNA polymerase | 31 | 0.556299 | 0.02885 | 0.049562 | core_enrichment |
| hsa00020 | Citrate cycle (TCA cycle) | 29 | 0.566346 | 0.029031 | 0.049619 | HTR2C/SST/UCN3/GRM4/NPBWR1/CRH/NMBR/GALR2/CHRNA1/UCN2/CCKBR/OXTR/GPR156/GRIN3B/GIPR/MCHR1/RLN2/GABRP/RXFP3/GRIK2/ADRA2A/SLURP2/GLRB/BDKRB1/GRIA2/AVPR1A/LPAR3/GRIK5/AGTR2/GLRA2/GABRB3/GHRL/PENK/BDKRB2/GRM7/GRIN1/F2RL2/GALR1/GLRA3/DRD1/GRM5/ADRA1B/MAS1/OPRL1/OXT/CALCB/P2RY6/LYPD6B/ADORA2B/CORT/NMUR2/LPAR4/CALCA/TACR1/NTS/TSHR/DRD5/F2RL1/GPR83/P2RX3/P2RY2/PLG/GPR35/QRFP/VIPR2/CTSG/ADRA2B/ADM2/GABRG3/HTR2A/GABRQ/CHRNA4/PTGER2/NMB/PTGFR/PNOC/RLN1/DRD2/ADCYAP1/P2RX6/LHB/GABRA2/C5AR1/UCN/DRD4/PYY/NPFFR1/LTB4R2/PGRMC1 |
